# Eye structure shapes neuron function in *Drosophila* motion vision

**DOI:** 10.1038/s41586-025-09276-5

**Published:** 2025-07-23

**Authors:** Arthur Zhao, Eyal Gruntman, Aljoscha Nern, Nirmala Iyer, Edward M. Rogers, Sanna Koskela, Igor Siwanowicz, Marisa Dreher, Miriam A. Flynn, Connor Laughland, Henrique Ludwig, Alexander Thomson, Cullen Moran, Bruck Gezahegn, Davi D. Bock, Michael B. Reiser

**Affiliations:** 1 HHMI Janelia Research Campus, Ashburn, VA USA; 2 Department of Neurological Sciences, University of Vermont, Burlington, VT USA

**Keywords:** Sensory processing, Navigation

## Abstract

Many animals use vision to navigate their environment. The pattern of changes that self-motion induces in the visual scene, referred to as optic flow^1^, is first estimated in local patches by directionally selective neurons^2–4^. However, how arrays of directionally selective neurons, each responsive to motion in a preferred direction at specific retinal positions, are organized to support robust decoding of optic flow by downstream circuits is unclear. Understanding this global organization requires mapping fine, local features of neurons across an animal’s field of view^3^. In *Drosophila*, the asymmetrical dendrites of the T4 and T5 directionally selective neurons establish their preferred direction, which makes it possible to predict directional tuning from anatomy^4,5^. Here we show that the organization of the compound eye shapes the systematic variation in the preferred directions of directionally selective neurons across the entire visual field. To estimate the preferred directions across the visual field, we reconstructed hundreds of T4 neurons in an electron-microscopy volume of the full adult fly brain^6^, and discovered unexpectedly stereotypical dendritic arborizations. We then used whole-head micro-computed-tomography scans to map the viewing directions of all compound eye facets, and found a non-uniform sampling of visual space that explains the spatial variation in preferred directions. Our findings show that the global organization of the directionally selective neurons’ preferred directions is determined mainly by the fly’s compound eye, revealing the intimate connections between eye structure, functional properties of neurons and locomotion control.

## Abstract (web summary)

In *Drosophila*, the physical structure of the eye has a key role in the directional tuning of motion-sensitive neurons, showing how navigational behaviour is tightly associated with anatomy.

## Main

**Fig. 1:**
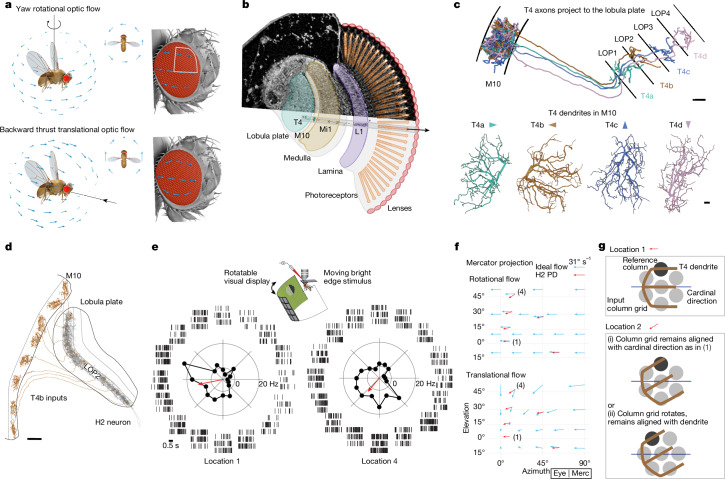
Non-cardinal direction preference by directionally selective neurons. **a**, Ideal optic-flow fields induced by yaw rotation or backward translation, on the right eye of a model fly. The local flow structure is similar near the eye’s equator, but differs away from it. **b**, Columnar architecture of the fly’s compound eye. Top, micro-computed tomography (µCT) cross-section showing visual-system neuropils. Bottom, schematic with representative EM-reconstructed connected, columnar neurons. Arrow shows facet (ommatidium) viewing direction; grey rectangle represents the corresponding column. **c**, Direction-selective T4 cells, of which there are four types, receive inputs in medulla layer M10 and project to one of lobula plate layers LOP1–LOP4. Each T4 neuron’s PD (arrowheads) opposes the primary dendritic orientation^4^. Scale bars, 5 μm (top); 1 μm (bottom). **d**, EM reconstruction of a wide-field H2 neuron (complete morphology in Extended Data Fig. 1b) dendrite receiving T4b inputs across lobula plate layer LOP2. Scale bar, 10 μm. **e**, Electrophysiological recordings of H2 responding to bright moving edges. Local PDs of an example H2 neuron (cell 2 from Supplementary Data 1; additional recordings in Extended Data Fig. 1) were recorded with whole-cell patch clamp. Raster plots show spiking activity to local edge motion in 16 directions at 2 retinal locations. Polar plots show average response rates (50 ms pre-stimulus baseline subtracted, negative responses indicate suppression from baseline). Red arrows indicate the local PD as the vector sum of responses. Inset, experimental set-up. **f**, Ideal optic-flow fields (yaw rotation, backward translational; 31° maximum retinal slip, matching moving edge speed) overlaid with averaged H2 local PDs from the responses of seven cells to bright and dark edges (see also Extended Data Fig. 1d). The plotted area corresponds to the white outlined area in **a**. The coordinate system and representation of spatial data are indicated with the boxed label ‘Eye | Merc’ for eye coordinates in the Mercator projection (complete key in Extended Data Fig. 1d). **g**, Two potential mechanisms for different T4 PD at location 4: (i) location-dependent dendritic sampling of the input column grid; or (ii) consistent dendritic orientation (with respect to local input columnar grid) with non-uniform mapping of visual space.

By moving through an environment, seeing animals can determine the physical layout and estimate their path using visual motion detection^1^ (Fig. 1a), analogous to solving the structure from motion problem in computer vision^7^. However, biological vision does not provide perfect geometrical measurements. Instead, the global structure is estimated using arrays of directionally selective neurons that report relative motion in small regions of the scene. Insects are highly skilled at rapid flight manoeuvres that depend on optic flow—the global structure of visual motion^8,9^. Research in *Drosophila* has elucidated key aspects of the circuits that compute motion detection and the visual control of navigation. Nevertheless, the intervening logic by which local motion detectors are spatially organized for reliable, behaviourally relevant estimation of optic flow remains unclear.

**Extended Data Fig. 1:**
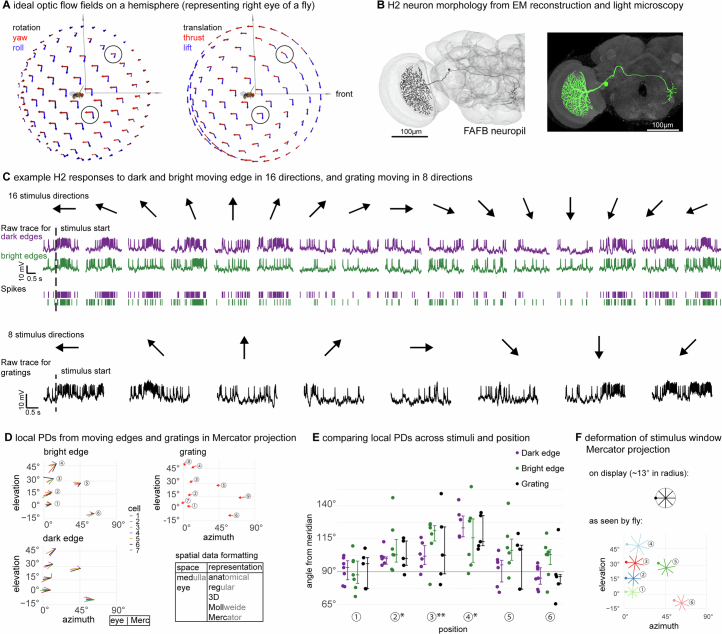
Global optic-flow patterns and H2 neuron results. Related to Fig. 1. **a**, Left: two optic-flow fields on a hemisphere (representing the fly’s right eye) induced by yaw (red) and roll (blue) rotations. Right: flow fields induced by thrust (red) and lift (blue) translations. The local red and blue flow vectors are orthogonal near the centre of the eye, but form skewed yet different angles near the boundary. **b**, EM reconstruction (in the FAFB^6^ volume) and light-microscopy image of a single H2 neuron (morphology consistent across 3 independent images of this driver line). **c**, H2 responses to dark and bright moving edges along 16 directions and to moving gratings along eight directions, at location 4 in Fig. 1f. **d**, Local H2 PDs for 7 individual flies measured in response to moving bright and dark edges at 6 retinal locations (left, individual cell responses to all 16 directions of bright and dark moving edges in Supplementary Data 1), and mean local H2 PDs for moving grating stimuli at 9 retinal locations (stimulus protocols detailed in Methods). Locations 2–6 correspond to those in Fig. 1f and **e**, and include 7 flies from experiment set #2 (8 directions); locations 7–9 include 5 flies from experiment set #1 (16 directions); location 1 includes flies from both experiment set #1 and experiment set #2. The retinal positions are combined across flies based on our procedure for aligning each fly’s head (uncertainty in the stimulus positions across all cells and positions, standard deviation <3°). Inset: key for plotting conventions of spatial data. **e**, Angular distribution of H2 local PDs with respect to the forward direction at six visual locations, represented as median +/- quartiles, from recordings of 7 cells at position 1, 2, and 6, and 6 cells at position 3, 4, and 5. Colour encodes stimulus types. Two-way ANONA without interaction found a statistically significant difference in the average angle by location (F(5) = 3.43, p < 0.01) but not by stimulus type (F(2) = 2.63, p = 0.08). Combining dark and bright moving edge data, one-sample Two-sided Wilcoxon test found significant differences (from 0) at locations 2 (p < 0.013), 3 (p < 0.0034), and 4 (p < 0.034). Three data points outside the plotting range are not shown. **f**, We simulate how 8 points regularly spaced on a circle, representing the distance travelled by a moving edge stimulus, will appear to the fly at 6 locations. The apparent expansion of these points (e.g., location 4) is due to the Mercator projection. Comparing distant positions, the vectors at position 4 (average ~11.6°) are ~7% smaller than those at position 1 (average ~12.7°). The angular distortions are accounted for in determining the local PDs of H2, but we do not correct the minimal changes in the speed of the stimulus. During the experiment, the display is repositioned, which further minimizes the distortions of the stimuli at higher elevations (see Methods).

A fruit fly eye comprises around 750 columnar units called ommatidia, which are arranged on an approximate hemisphere to maximize the field of view^10^. Each ommatidium houses photoreceptors and collects light from a small area of visual space^10,11^. Along the motion pathway, columnar neurons, such as L1 and Mi1, receive, modify and transmit photoreceptor signals, preserving retinotopy^4,12,13^ (Fig. 1b). T4 neurons are the local ON-directionally selective cells^14,15^ that are sensitive to bright edge movement (analogous T5 neurons are the OFF-directionally selective cells^5,16–18^). T4 neurons integrate columnar inputs along their dendrites, and the principal anatomical orientation of these dendrites corresponds to the neuron’s preferred direction (PD) of motion^4,5^ (Fig. 1c). There are four types of T4 neuron, each with a distinct dendritic orientation, and an axon terminal projecting to one of four layers in the lobula plate^2,19^. These neurons are best understood near the centre of the eye, where the PDs of each type align with one of four orthogonal, cardinal directions (forwards, backwards, up and down)^2,4^. It is unclear how well this relationship holds for T4 neurons away from the centre. Indeed, owing to the spherical geometry of the compound eye, the PDs cannot be globally aligned with the cardinal directions while also maintaining orthogonality between types (Extended Data Fig. 1a). Because wide-field neurons in the lobula plate integrate from large ensembles of T4 neurons^19,20^, the directional tuning of T4 neurons across the eye shapes global optic-flow processing.

## Non-cardinal local direction tuning

To survey the directional preference of T4 neurons across visual space, we measured the local PDs of H2 (refs. ^19,21^). This large neuron serves as a nearly perfect proxy for the inputs into the second layer of the lobula plate (Fig. 1d and Extended Data Fig. 1b). H2 receives about 85% of its optic-lobe inputs from T4b and T5b neurons, with around 90% of T4b and T5b neurons connecting to H2 (ref. ^22^). Furthermore, as a wide-field spiking neuron, H2 allows for high-signal, low-noise measurements of the local PDs across the field of view without the confound of multi-layer inputs seen in the VS cells of the lobula plate^23^. We used whole-cell electrophysiology to record the responses of H2 to bright and dark edges moving in 16 directions at several locations on the eye (Fig. 1e,f). We find that H2’s PD is aligned with cardinal, back-to-front motion near the eye’s equator, as previously reported^21,24,25^. However, at more dorso-frontal locations, the PD shows a prominent downward component (Fig. 1e,f), consistently across flies and stimuli (Extended Data Fig. 1c–e). The moving bright and dark edge stimuli isolate the contributions of T4 and T5 neurons^2,15,26^. Because H2 responses do not significantly differ between these stimuli (Extended Data Fig. 1c,e), the local variations in T4b and T5b PDs are likely to be matched across the field of view. The anatomical analysis in this study will focus on T4 neurons, but the mechanism we find extends to T5 neurons (see ‘Discussion’).

Notably, the local PDs do not resemble a purely rotational flow field (as expected for H2 from blowflies^25^) or a purely translational one (Fig. 1f). This shift in the local PDs of H2 implies that T4 neurons are not globally tuned to cardinal motion directions, a prediction that agrees well with a previous imaging study of T4 and T5 axons^27^. But what causes T4 cells to change their directional preference across the eye remains unclear. Two parsimonious mechanisms could account for how T4 dendrites are differentially oriented with respect to each other at different retinotopic locations (Fig. 1g; location 1 versus location 4). Either T4 dendritic orientations vary with respect to their retinotopic inputs throughout the eye (scenario (i) in Fig. 1g), or T4 dendrites use a conserved integration strategy, but the representation of space by the array of input neurons is non-uniform across the eye (scenario (ii) in Fig. 1g; note the rotation of the column grid). To distinguish between these two hypotheses, we reconstructed the morphology of hundreds of T4 neurons to determine the spatial integration pattern in the medulla. We then generated a high-resolution map detailing the spatial sampling by each ommatidium in the eye. Combining these datasets, we map the PDs of T4 neurons into visual space, thereby revealing the mechanism that underlies the non-cardinal motion sensitivity. Finally, our global analysis of the fly eye reveals principal axes of body movements whose corresponding optic flow is measured most sensitively by the visual system.

## Conserved T4 arbor shape across the eye

**Fig. 2:**
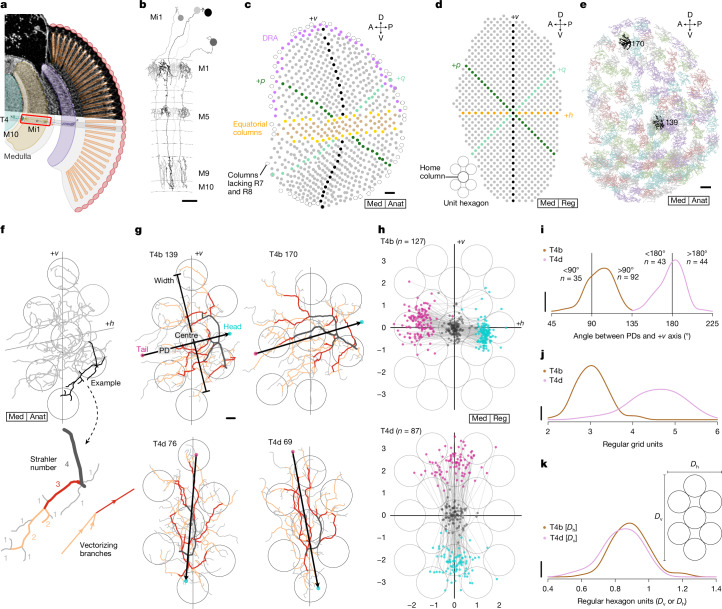
EM reconstruction of T4 dendrites across the eye reveals a stereotypical pattern of arborization. **a**, Schematic of the *Drosophila* visual system, highlighting Mi1 and T4 cells in a column. **b**, EM reconstruction (FAFB dataset^6^) of four Mi1 cells arborizing in medulla layers M1, M5 and M9–M10. Scale bar, 10 μm. **c**, Medulla columns identified by the centres of mass of Mi1 cells in M10. Magenta dots, dorsal rim area (DRA) columns^56^; middle band, equatorial region with seven (yellow) or eight (tan) photoreceptors in corresponding lamina cartridges (Extended Data Fig. 2b); black dots, central meridian dividing columns into approximately equal halves; empty circles, medulla columns lacking R7 and R8 inputs, presumably with no corresponding ommatidia^57^. Scale bar, 10 μm. All boxed keys are defined in Extended Data Fig. 1d. **d**, Medulla columns mapped onto a 2D regular grid (‘Med | Reg’) with orthogonal +*h* and +*v* axes defined by equatorial region and central meridian. The +*p* and +*q* axes are shown for consistency with previous work^31,58^. **e**, Dendritic arbors of 176 T4b cells in M10. The two highlighted examples are shown in **g**. Scale bar, 10 μm. **f**, An example T4b dendrite (T4b 139). Bolded branch (top) colour-coded by Strahler number (bottom). Each branch is represented as a vector, and the dendrite’s anatomical PD is defined as the vector sum of all Strahler number 2 and 3 branches. **g**, Example T4b and T4d dendrites, with PD and width indicated. Branches coloured by Strahler number (>3 in black). The seven circles represent the home column and six nearest neighbours. Scale bar, 1 μm. **h**, PDs mapped to regular grid using 19 neighbouring columns (Methods). Tail, centre and head of each PD vector indicated as in **g**. **i**, Distribution of angles between T4 PDs and the +*v* axis. **j**, PD amplitude distributions in regular grid units for T4b and T4d neurons (two-sided Wilcoxon rank test, *P* = 2.2 × 10^−16^). **k**, PD amplitudes normalized by respective hexagon length units (defined in inset; two-sided Wilcoxon rank test, *P* = 0.015). The scale bars for **i**–**k** span from zero to the height of the uniform distribution.

**Extended Data Fig. 2:**
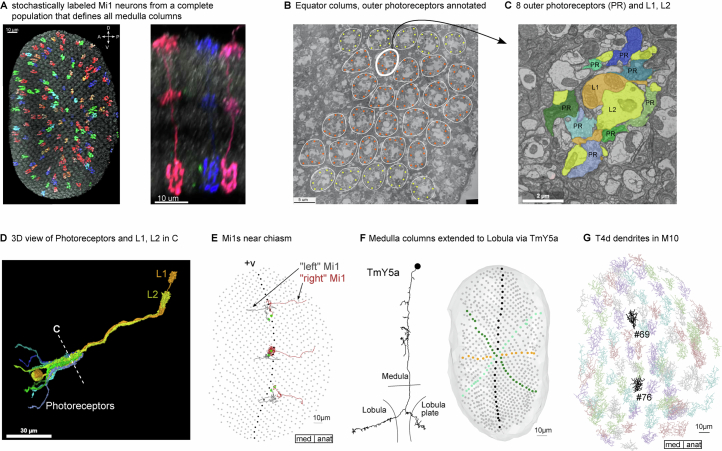
Anatomical considerations for mapping visual neurons throughout the medulla. Related to Fig. 2, **a**, Left: light microscopy of stochastically labelled Mi1 cells in the background of nc82 staining from a single substack showing the M10 layer of the medulla. Right: side view of stochastically labelled Mi1 cells (3 out of 4 columns have visible cells). **b**, A cross-section of the equatorial region in the lamina (from one eye of the FAFB EM volume), identified by cartridges (white contour) with 8 (orange) or fewer (yellow) outer photoreceptors. All equatorial columns corresponding to annotated lamina cartridges are indicated in Fig. 2c. **c**, A zoomed-in view of a single cartridge showing L1/2 cells and eight photoreceptor cells. L1 cells receive input from photoreceptor cells (R1-6) and output to Mi1 cells. **d**, 3D rendering of the same cartridge. **e**, Chiasm medulla columns (green) with corresponding Mi1 cells at three vertical locations identified by the twisting of R7 and R8 photoreceptor axons (not shown). For comparison, the central meridian is indicated in black. **f**, Extension of medulla column map to the lobula via interpolation of the positions of 63 reconstructed TmY5a neurons. **g**, The dendritic arbors of 114 reconstructed T4d cells in M10. The highlighted neurons are the examples in Fig. 2g.

To compare the arborization patterns of T4 neurons across the entire medulla, we manually reconstructed all 779 Mi1 neurons on the right side of the full adult fly brain (FAFB) volume^6^ to establish a neuroanatomical coordinate system. Mi1 neurons are columnar cells that are a major input to T4 neurons^4,15^ (Fig. 2a,b and Extended Data Fig. 2a). Their reconstruction was essential for propagating retinotopic coordinates from the more regular, distal layers of the medulla to layer M10, where Mi1 neurons synapse onto T4 dendrites. All Mi1 neurons in M10 (Fig. 2c) were then mapped into a two-dimensional (2D) regular grid with the orthogonal +*h* and +*v* axes (Fig. 2d). Because the rows of Mi1 neurons are not generally straight (Fig. 2c), capturing the global grid structure (Fig. 2d) enables the direct comparison of T4 neurons’ arborization patterns across the eye. Two special rows serve as global landmarks: the ‘equator’ (Fig. 2d, in orange) is derived from the equatorial region in Fig. 2c, which is located via the corresponding lamina cartridges with additional photoreceptors (Methods and Extended Data Fig. 2b–d); and the ‘central meridian’ (Fig. 2d, in black) divides the points into roughly equal halves and coincides well with the first optic chiasm (Extended Data Fig. 2e). This regular grid mapping required access to the complete medulla and lamina neuropils in the electron microscopy (EM) volume, and further tracing of columnar neurons can extend this coordinate system into deeper neuropils, such as the lobula (Extended Data Fig. 2f).

**Extended Data Fig. 3:**
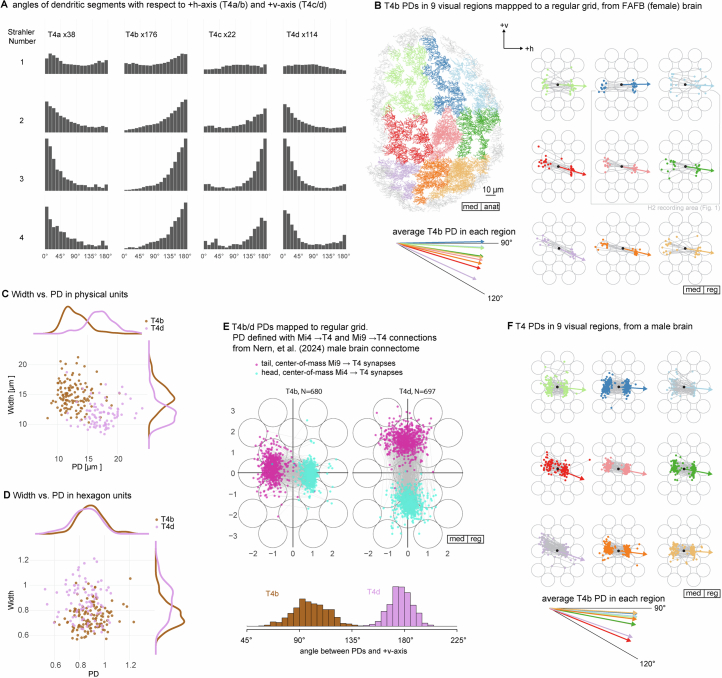
Analysis of the dendritic morphology of T4 neurons. Related to Fig. 2. **a**, The angular distribution of dendritic segments, treating each segment as a vector and calculating the angle formed with respect to the grid axes. For T4a/b, the angle was computed with respect to the +h axis, normalized by solid angle, and grouped by Strahler number. For T4c/d, the angle was computed with respect to the -v-axis. We use Strahler number = 2 and 3 branches to compute the PD vector because these segments are abundant and have more consistent directions, while level 1 branches are nearly non-directional **b**, T4b PD vectors mapped to a regular grid for cells in 9 visual regions, whose divisions in the eye coordinates (using the result from Fig. 4) are azimuth = [−15°, 35°, 70°, 150°] and elevation = [−70°, −10°, 20°, 90]. The arrow in each region originates from the centre of the central circle and denotes the mean PD direction. The bottom left plot compares each region’s vector mean of the PD directions. **c**, Dendrite width vs. PD length in the medulla’s raw physical units [µm]. **d**, Width vs. PD length: T4b PDs and T4d widths are normalized by the horizontal hexagon unit *D*_h_, while T4b widths and T4d PD lengths are normalized by the vertical hexagon unit *D*_v_ (Fig. 2k inset). **e**, As an alternative method, we used the centres of mass of synaptic connections Mi4 → T4 and Mi9 → T4 from a new male optic lobe EM dataset^22^ of all T4b and T4d neurons (excluding those close to the medulla’s boundary) to define the PDs. The results are consistent with Fig. 2h. **f**, Partition analysis shows a small downward PD bias in the ventral posterior visual area, consistent with data from the female brain in **b**.

Because the orientation of T4 dendrites corresponds to their PD (Fig. 1c), we reconstructed the complete dendritic morphology of 176 horizontal-motion-sensitive T4b cells (Fig. 2e) and 114 vertical-motion-sensitive T4d cells (Extended Data Fig. 2g). We applied branching analysis developed for river networks^28^ to each T4 neuron’s dendritic tree to capture its primary orientation (Methods, Fig. 2f and Extended Data Fig. 3a) as an anatomical PD estimate. This estimate yields a PD vector, represented as an arrow going through the dendrite’s centre of mass, with a length corresponding to the spatial extent of the dendrite along the PD (Fig. 2g).

Although the dendritic tree of each T4 neuron is idiosyncratic in its fine features, many conserved characteristics, such as the size and dominant branch orientation, suggest that these neurons are more stereotyped than would be expected from visual inspection of their morphology. To examine potential stereotypy, we transformed all T4 PD vectors into the regular grid of Mi1 neurons (Fig. 2d,h), using kernel regression that maintains the spatial relationships between each PD and its neighbouring Mi1 neurons (excluding boundary T4 neurons; Methods). Once transformed, the PD vectors for both T4 types show high similarity. First, the centres of mass for all T4 dendrites fall within a ‘home’ column. Second, the heads and tails of the PD vectors are each localized to a small area (the standard deviation of the head and tail positions is less than half the inter-column distance). Third, the dendrites of both types roughly span a single unit hexagon (one home + six nearest columns). The PD vectors of T4b and T4d neurons are aligned mainly with the +*h* and −*v* axes, respectively (Fig. 2i). The population of T4b neurons has a broader angular distribution and a downward bias (greater than 90°), which is accounted for mostly by neurons in the posterior-ventral regions of the eye (Extended Data Fig. 3b; note that the +*h* axis points towards the posterior medulla, corresponding to anterior on the eye because of the optic chiasm). We measured a modest but consistent spatial bias in a second EM dataset^22^ (Extended Data Fig. 3e,f), suggesting that this small offset in T4b—but not T4d—PDs is unlikely to be a technical limitation of either dataset. Instead, it might reflect some developmental effects, potentially serving a functional role, but it cannot explain the H2 data (see also Fig. 4c, inset). The PD vector lengths between the types are notably different (Fig. 2j and Extended Data Fig. 3c). However, the unit hexagon is anisotropic, because its height is greater than its width (Fig. 2d, inset); we therefore defined a new unit distance, the ‘hexagon unit’, as the edge-to-edge span: three horizontal columns (*D*_h_) for T4b and five vertical columns (*D*_v_) for T4d (Fig. 2k, inset). When we normalize the PD length by these unit distances for each type separately, we find that T4b neurons and T4d neurons are now highly overlapping (Fig. 2k and Extended Data Fig. 3d). Because we identified the T4 types on the basis of lobula plate layer innervation, the marked within-type similarity of the PDs does not support further divisions based on morphology^19,27,29^. Our analysis thus reveals that T4 neurons share a universal sampling strategy—throughout the eye, they innervate a unit hexagon of columns while establishing a PD by aligning their dendrites mostly in one direction, parallel to either the horizontal or the vertical axes of the hexagonal grid.

## Non-uniform sampling in the fly compound eye

**Fig. 3:**
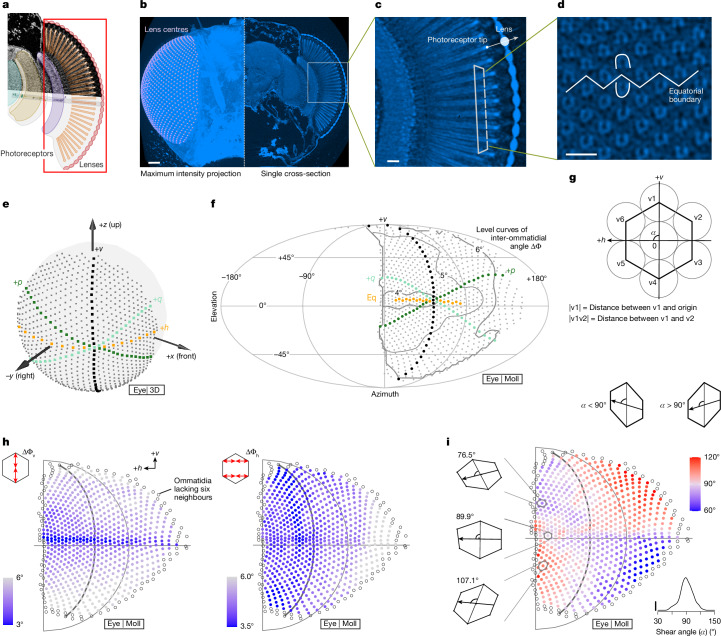
Non-uniform sampling of visual space established by µCT of the *Drosophila* eye. **a**, Schematic of the *Drosophila* visual system, highlighting the retina. **b**, Maximal intensity projection of a whole fly head scanned with µCT. Lenses are labelled with white spheres in the left half. Scale bar, 50 μm. **c**, Magnified cross-section (boxed region in **b**) showing lenses and photoreceptors, with example tip–lens pair defining the viewing direction of that ommatidium. Scale bar, 10 μm. **d**, Magnification of boxed region in **c**. Photoreceptors in each ommatidium are arranged in an ‘n’ or ‘u’ shape above or below the equator^30^. Scale bar, 20 μm. **e**, Right eye ommatidia directions represented by points [*x*, *y*, *z*] on a unit sphere. The +*h* row is based on **d**, and +*v* divides approximately equal halves. **f**, Mollweide projection of three-dimensional (3D) ommatidia directions (‘Eye | Moll’) and inter-ommatidial angles (ΔΦ, averaged over six neighbours). Contour lines show iso-levels. **g**, A unit hexagon with seven columns (home column and surrounding six), illustrating the conventions used to characterize the geometry of the eye’s viewing directions. The +*h* axis is the line from the centre of two right neighbours to two left neighbours, and the +*v* axis is the line from bottom neighbour to top. Shear angle *α* is the angle between +*h* and +*v* axes. Inter-ommatidial angles include six-neighbour ΔΦ = mean(|v1|, |v2|, |v3|, |v4|, |v5|, |v6|), vertical-neighbour ΔΦ_v_ = mean(|v1|, |v4|), horizontal-neighbour ΔΦ_h_ = mean(|v2v6|, |v3v5|). Using small-angle approximation, angles are computed using the Euclidean distance ($| \cdot |$) of points on the unit sphere in **e**. **h**, Spatial distribution of ΔΦ_v_ and ΔΦ_h_. Points represent ommatidia directions as in **f**. **i**, Distribution of shear angles across the right eye, with three example unit hexagons from the same vertical grid line, each aligned to the meridian line through its home column. Inset histogram shows all shear angles. Vertical scale bar corresponds to a uniform distribution. In **h**,**i**, points lacking a complete neighbour set are empty circles. Points not matched to medulla columns (Fig. 4a) are not plotted.

**Extended Data Fig. 4:**
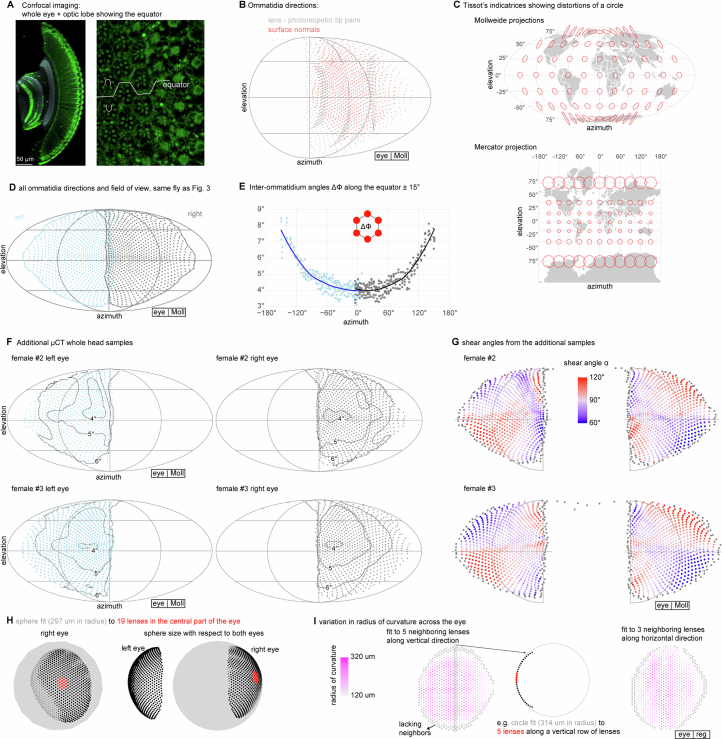
Ommatidia viewing directions in *Drosophila* compound eye maps. Related to Fig. 3. **a**, Left: a high-resolution confocal image showing autofluorescence from ommatidia (green, retina) and GFP-labelled Mi1 neurons (green, medulla). Lamina and medulla neuropils are visible (grey) due to nc82 antibody staining. Right: a different cross-section showing the arrangement of individual photoreceptors near the equator. Six dots (R1–R6) arranged as an “n” or “u” shape can be readily seen in each ommatidium. The 7th smaller dot (R7 + R8) in the centre is also often visible. Image is representative of >10 imaged samples. **b**, Comparison of ommatidia directions defined by lens-photoreceptor tip pairs (grey, used in this study) and by surface normal (red). The surface normal is a typical approximation for the viewing direction, but this estimate differs substantially from that based on the high-resolution structure of each ommatidium. Two corresponding rows are connected with grey lines to illustrate the differences in different eye regions. Notably, these differences are small near the centre of the eye and very large towards the front of the eye. **c**, Comparison of Mollweide vs. Mercator projections. Red circles show the amount of distortion (Tissot’s indicatrix) with respect to an infinitesimal circle on a sphere. See Methods for a discussion of our choice. **d**, Ommatidia directions and field of views (contours) for both eyes of the same fly as in Fig. 3. **e**, Six-neighbour inter-ommatidial angle (ΔΦ) along the equator (+/−15° elevation) for this same fly. **f**, Ommatidia directions and ΔΦ for two additional female flies. **g**, Shear angles for these two female flies, plotted as in Fig. 3i. **h**, Least squares spherical fit to the central 19 lenses shows the centre of the eye is flatter than the edges. **i**, The least-square circle fits to neighbouring lenses in vertical and horizontal directions, showing increasing curvature near the eye’s periphery.

Having established that the PD of T4 neurons is governed by a simple local rule that is conserved throughout most of the medulla (strong evidence against hypothesis (i) in Fig. 1g), understanding the global PD organization now reduces to understanding how visual space, sampled by the compound eye, maps onto the array of medulla columns (required to evaluate hypothesis (ii) in Fig. 1g). Because the EM volume did not contain the eye, we imaged whole heads of female flies with approximately the same number of ommatidia to match the EM dataset. We first tried confocal imaging (Extended Data Fig. 4a), but ultimately used micro-computed tomography (µCT; Fig. 3a–c). The isotropic, approximately 1-µm resolution of the µCT data allowed us to define the viewing direction of each ommatidium (as the vector connecting the ‘tip’ of the photoreceptors to the centre of each corneal lens; Fig. 3b,c and Extended Data Fig. 4b) and to locate the eye’s equator (using the chirality of the photoreceptor arrangement^30^; Fig. 3d).

**Fig. 4:**
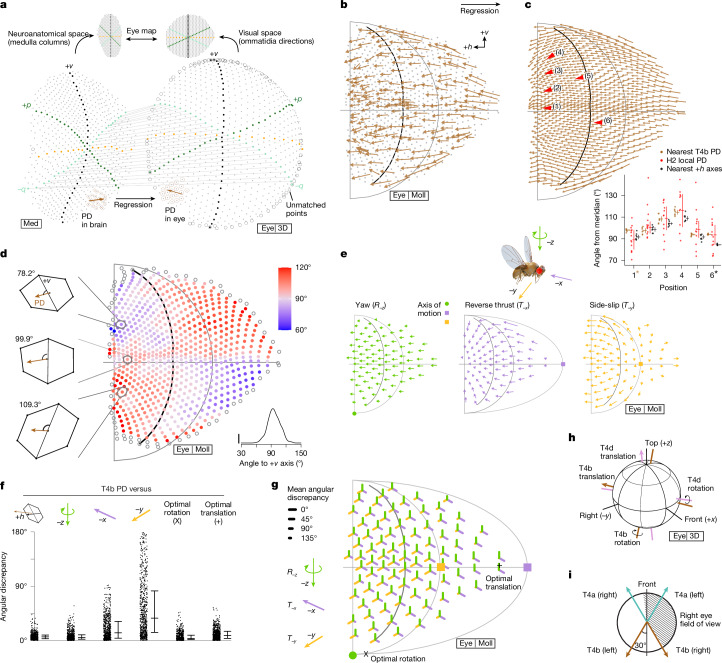
Mapping neuroanatomical space into visual space explains the global organization of directionally selective neuron PDs. **a**, Eye map: one-to-one mapping between medulla columns from EM reconstruction and ommatidia directions from µCT head scan by regular grid mapping. Empty circles show unmatched (peripheral) columns. A T4b PD vector is mapped from the medulla to visual space by applying kernel regression on neighbouring columns (highlighted in brown). **b**, Mapping 176 reconstructed T4b PDs to visual space. Arrow size reflects 1–99% range of dendrite’s span along individual PDs. Example vector from **a** is bolded. **c**, T4b PD field constructed by kernel regression from **b**, assigning one T4b PD vector to each ommatidia direction (length rescaled by 50%). For comparison, average PDs recorded from H2 neurons are replotted from Fig. 1f as red arrowheads. Inset compares H2 local PDs with T4b PDs and +*h* axes (*n* = 7 cells, median ± quartiles); all comparisons are non-significant (*P* > 0.05), except T4b PDs at location 1 (*P* = 0.006), and +*h* axes at location 6 (*P* = 0.04), two-sided *t*-test (asterisks indicate *P* < 0.05). **d**, Angular difference between T4b PD field and +*v* axes. This PD field structure matches ommatidial shearing features (Fig. 3i). **e**, Ideal, cardinal optic-flow fields induced by yaw rotation, reverse thrust and side-slip. Ommatidia directions downsampled by 9×. **f**, Angular differences between T4b PD field, +*h* axis, three cardinal self-motion optic-flow fields and optimized self-motion flow fields (Extended Data Fig. 6e), represented as median ± quartiles. **g**, Spatial distribution of angular differences with three cardinal self-movement optic-flow fields, represented as three line segments (colour-matched to cardinal movements, length proportional to angular difference). ‘X’ and ‘+’ indicate optimal rotation and translation axes, respectively. **h**, Optimal rotation and translation axes for T4b and T4d PD fields in the fly’s eye coordinates. **i**, Top view of optimal translation axes for T4a and T4b in both eyes at the equator.

**Extended Data Fig. 5:**
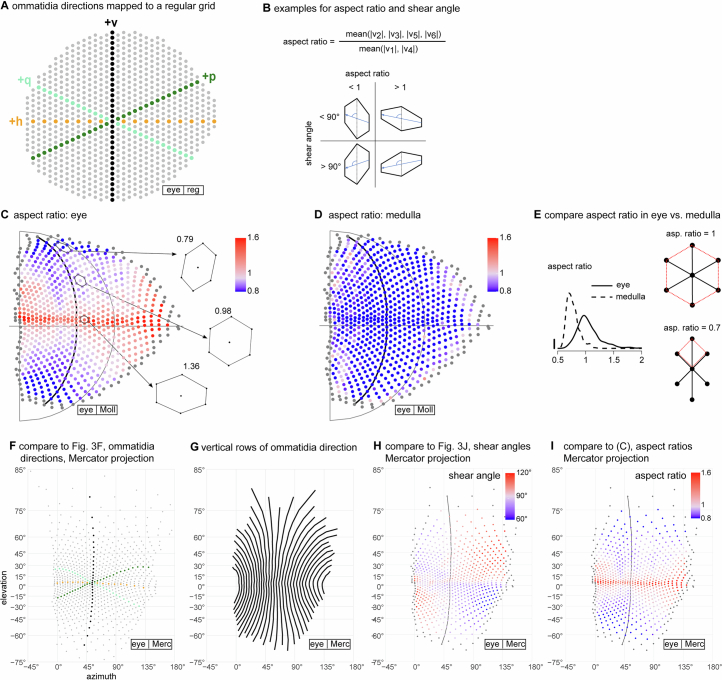
Quantification of the ommatidial viewing direction grids. Related to. Fig. 3. **a**, Ommatidia directions mapped onto a regular hexagonal grid. **b**, Definitions of aspect ratio and shear angle for a unit hexagon with examples. **c**, Aspect ratios calculated from ommatidia directions. **d**, Aspect ratios calculated from medulla columns. **e**, Distributions of aspect ratio for ommatidia and medulla columns. A comparison of the aspect ratios for a regular hexagonal grid and a regular square grid shows that the arrangement of ommatidia directions is more hexagonal. In contrast, the arrangement of medulla columns is more square-like (hence the different choices of regular grids in **a** and Fig. 2d). **f**, Fig. 3f replotted using Mercator projection. **g**, Vertical rows of ommatidia directions given by the grid structure, using Mercator projection. **h**, Fig. 3i replotted using Mercator projection. **i**, The aspect ratio map in **c** replotted using Mercator projection.

We represent the ommatidia directions in eye coordinates as points on a unit sphere ([*x*, *y*, *z*]; Fig. 3e) or in a 2D ([azimuth, elevation]) geographical projection (Mollweide projection, Fig. 3f; Mercator projection, Extended Data Fig. 5f–j; comparison in Extended Data Fig. 4c). The field of view spans from directly above to −70° in elevation, and in azimuth, from less than 10° into the opposite hemisphere in front to around 155° behind, and is quite consistent across maps of ommatidia directions produced from three different females (Extended Data Fig. 4d,f). These maps show a binocular overlap zone of less than 20° and a posterior blind spot of around 50°, in excellent agreement with previous optical measurements of a *Drosophila* eye^31^. These zones are adjustable, because flies can coordinately redirect all ommatidia by up to about 15° using two muscles attached to the retina^32^. The ommatidia directions are well described by a hexagonal grid that we then aligned to the medulla column grid using the equator (+*h*) and central meridian (+*v*) as global landmarks (Extended Data Fig. 5a and Fig. 4a).

The hexagonal arrangement is a dense spatial packing that maximizes the eye’s resolving power^10^. However, many unit hexagons are irregular, as illustrated by the inter-ommatidial angle (ΔΦ) and the shear angle (*α*; Fig. 3g). ΔΦ is smallest at the front near the equator and increases in size away from this region (Fig. 3f and Extended Data Fig. 4e), which is consistent with a previous scanning-EM measurement in *Drosophila*^33^. When calculated separately for vertical (ΔΦ_v_) and horizontal (ΔΦ_h_) neighbours (Fig. 3h), we find that the vertical visual acuity is highest (smallest ΔΦ_v_) along the equator (a typical feature of flying insect eyes^34,35^, not previously reported for *Drosophila melanogaster*^31^). The horizontal acuity is highest in the frontal part of the eye, although the effect of photoreceptor pooling (neural superposition^11^) on these acuity differences is unclear. These acuity differences are consistent with the changes in the aspect ratio of the unit hexagons across the eye (Extended Data Fig. 5b–e). Furthermore, the shear angle of the hexagons systematically changes, with the most regular hexagons (*α* ≈ 90°) found near the equator and the central meridian and sheared hexagons with *α* < 90° in the fronto-dorsal and posterior-ventral quadrants, and *α* > 90° in the other quadrants (Fig. 3i). The eye’s surface is approximately spherical, with a central region that is relatively flatter than the periphery (Extended Data Fig. 4h,i). This µCT scan of the full fly head provides a detailed description of how the compound eye samples visual space. Our analysis reveals an irregular arrangement of ommatidia directions with spatially varying aspect ratios, inter-ommatidial angles and shear angles that shape the inputs to visual pathways. We next sought to ascertain whether this non-uniform sampling could explain the global structure of T4 PDs.

## Eye–brain mapping explains global tuning

**Extended Data Fig. 6:**
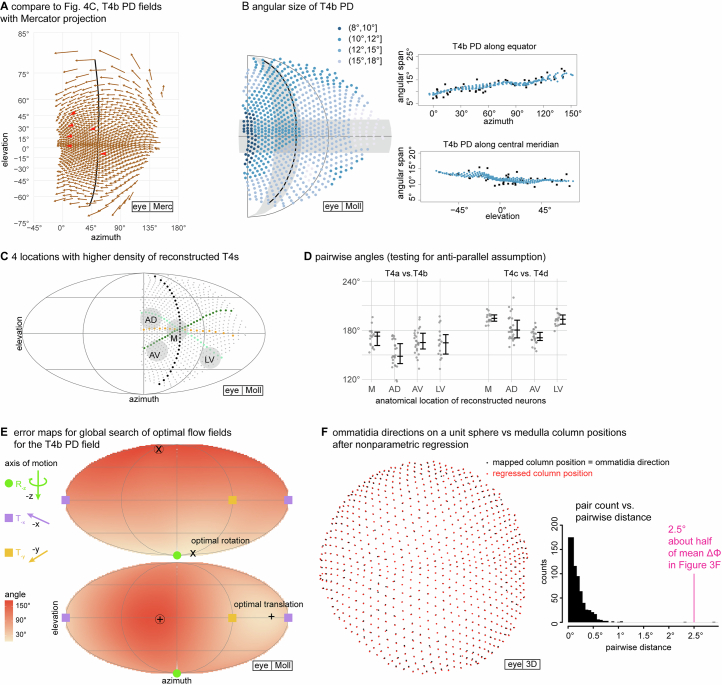
Further quantification of T4b PD distribution. Related to Fig. 4. **a**, Fig. 4c replotted using Mercator projection. **b**, Visual angles subtended by T4b PD vectors (i.e., angular size). Scatter plots show the reconstructed T4b PDs (black dots, also in Fig. 4b and interpolated ones (blue dots, also in Fig. 4c) along the equator (+/−15° horizontal shaded band) and the central meridian (+/−15° vertical shaded crescent). Most T4b PDs span between 10° and 15°, but there are almost twofold differences found across the eye, with larger spans towards the rear and smaller spans near the front. **c**, We reconstructed all T4 types (16 ~ 20 cells) in an early pilot study at each of these four locations. **d**, We first mapped these T4 neurons’ PD vectors to the regular grid in Fig. 2h. Then, we computed the angles between all T4a vs T4b pairs at each location, represented here as median +/- quartiles. Similarly, for T4c vs T4d. **e**, Global search for optimal optic-flow fields yielded these error maps, showing the average angular differences between the T4b PD field and the optic-flow field induced by a rotation (left) or translation (right) along that direction (see Methods: ‘Ideal optic-flow fields’). Symbols “+” and “X” denote the axes of translational and rotational motion with minimal angular difference, respectively. Symbols ⨁ and ⨂ denote those with maximal differences (minimum and maximum are antipodal). **f**, Having established a 1-to-1 matching between two point sets in Fig. 4a, we use non-parametric regression to map new points from one space to the other. As a test of this method, we computed the pairwise distance from the regressed position of a medulla column to its matched ommatidia direction. ‘Perfect’ regression would yield zero pairwise distances, but perfect regression is impossible because the deformation is not affine. Nevertheless, these pairwise distances are a small fraction (nearly all <10%) of the inter-ommatidial angles.

**Extended Data Fig. 7:**
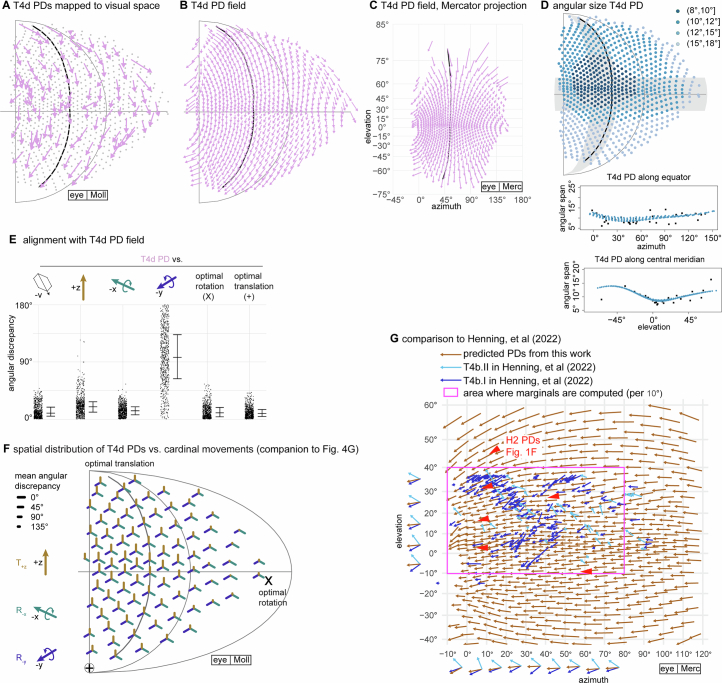
T4d neuron analysis. Related to Fig. 4. **a**, T4d PDs mapped to eye coordinates. **b**, Interpolated T4d PD field (arrows are rescaled to 50% of length in **a**). **c**, The T4d PD field from **b** replotted using Mercator projection. **d**, Visual angles subtended by T4d PD vectors (i.e., angular size). Scatter plots show the reconstructed T4d PDs (black dots, also in **a**) and interpolated ones (blue dots, also in **b**) along the equator (+/−15° horizontal shaded band) and the central meridian (+/−15° vertical shaded crescent). **e**, Angular differences between T4d PD field, -v-axis, three cardinal self-motion optic-flow fields (lift, leftward roll, and upward pitch), and optimized self-motion flow fields, represented as median +/- quartiles. **f**, Spatial distribution of angular differences between T4b PD field and the three cardinal self-motion optic-flow fields. **g**, Comparison of our anatomically predicted T4b PD field (brown arrows) and the H2 local PDs (red arrowheads) from the current study to the measured T4b PDs in a recent study^27^ that subdivided these measurements into two candidate subtypes. To compare all T4b PD measurements, we compute the vector means for each type within the magenta box in 10° strips (horizontal and vertical) and plot these along the margins. In many retinal positions, we find reasonable agreement between the T4b anatomical PDs and the measured T4b.I data (e.g., the discrepancy could be due to global head alignment differences). The T4b.II data do not match the T4b anatomical PDs at any location.

We now had all the data required to map T4 PDs from their neuronal coordinates into the visual coordinates of the eye. We used the regular grids established for medulla columns (Fig. 2d) and ommatidia directions (Extended Data Fig. 5a) to construct a one-to-one mapping between them, matching from the centre outward (Fig. 4a and Supplementary Videos 1 and 2). We used kernel regression to transform the T4b PDs into eye coordinates (Fig. 4b and Extended Data Fig. 6f). Finally, the T4b PDs were estimated, using kernel regression from data in Fig. 4b, for all ommatidia directions (Fig. 4c, Extended Data Fig. 6a,b and T4d in Extended Data Fig. 7a–d). Because T4a–T4b and T4c–T4d are approximately anti-parallel (Extended Data Fig. 6c,d), these estimates directly extend to all T4 types. The stereotypical alignment of T4b PDs in the medulla (Fig. 2h) suggests that the PD field in eye coordinates should follow the ommatidia shearing (Fig. 3i), which is indeed the case (Fig. 4d). The T4b PDs are well aligned with the spatially registered H2 responses (red arrowheads in Fig. 4c; only position 1 shows a significant difference between the electrophysiological data and the anatomical measurement; two-sided *t*-test). Notably, both show a downward component in dorso-frontal PDs, which, in our anatomical analysis, could only have originated from the non-uniform sampling of visual space. H2 responses also align well with the local +*h*-axis orientations of the eye’s hexagonal grid (Fig. 4c, inset; only position 6 shows a significant difference). This strong concordance confirms that the eye’s non-uniform sampling pattern is the main determinant of H2’s directional tuning. The global pattern of the T4b PD field has features of a translational optic-flow field (Fig. 1a,f) that can be readily seen in the Mercator projection comparing the PD field with the eye coordinate parallel lines of constant elevation (Extended Data Fig. 6a). Because T4b provides substantial input to H2 (ref. ^19^), this agreement provides strong evidence for mechanism (ii) in Fig. 1g, and validates our anatomy-based PD prediction and mapping into visual coordinates. Together, these results show that the non-uniform sampling of the eye powerfully shapes the organization of PDs available for optic-flow processing.

We next investigated whether the T4b PD field (Fig. 4c) is optimized for the optic flow induced by cardinal motion along body axes (Fig. 4e), as has been found in mouse directionally selective neurons^3^. The distribution of angular differences between the T4b PD field, the eye’s +*h* axis and several cardinal optic-flow fields shows that the PDs are best aligned with the eye axis, yaw rotation and reverse thrust (Fig. 4f). By contrast, there is a large spread in the differences between the PD field and side-slip optic flow, suggesting substantial regional variation. The spatial distribution in Fig. 4g can be interpreted as predictions for how T4b neurons at any location on the eye would respond to the three cardinal flow fields. This map shows that T4b PDs in the central eye agree well with all three flow fields, whereas side-slip flow matches only the PDs in the anterior eye (the analysis of T4d in Extended Data Fig. 7e,f shows that roll rotation is the best-matched canonical flow field). Consequently, all neurons that integrate from most of a lobula plate layer, like H2 (Extended Data Fig. 1b), will inherit this eye-derived sensitivity. However, by selectively integrating from regional patches, lobula plate neurons can encode diverse optic-flow features, providing an expansive set of motion patterns for behavioural control.

We wanted to establish which body- or head-movement-generated flow fields the T4b population was maximally sensitive to. We searched and found the optimal rotation axis (by minimizing angular differences; Methods) quite close to the yaw axis and a translation axis between reverse thrust and side-slip, near the posterior boundary of the eye’s field of view (rightmost distributions in Fig. 4f, locations denoted with symbols in Fig. 4g and and complete error map in Extended Data Fig. 6e). The comparison between the optimal axes for T4b and T4d (Extended Data Fig. 7f) PDs reveals a strong agreement. Specifically, the body and head yaw axis aligns with the optimal translation axis of T4d neurons, whereas the non-canonical optimal translation axis of T4b neurons aligns with the optimal rotation axis of T4d neurons (Fig. 4h)—a noteworthy difference from estimated motion sensitivities in the mouse retina^3^. Because optic flow is a direct consequence of movement, these axes of maximal motion sensitivity are likely to be fundamental for controlling body and head movements. Notably, the optimal translation axes for the left and right T4a populations are near to the eye’s equator and approximately ±30° from the midline (Fig. 4I), aligned with the retinal positions where flies trigger goal-directed body turns towards objects^36,37^, within the zone of the eye’s highest acuity (Fig. 3f). Recent calcium imaging experiments in H2 and HS neurons (receiving both T4a and T5a inputs) confirm this prediction; the neurons showed maximal responses to a non-canonical translation axis between thrust and side-slip^38^. Moreover, we note a marked resemblance between the optimal translation axes for T4a and T4b (Fig. 4I) and the tuning of optic-flow-sensitive inputs to the central complex^39^, from which the transformations between body-centred and world-centred coordinates are built^40^. This unexpected correspondence of maximal motion sensitivities reveals a deep link between the eye structure and the coordinate systems organizing goal-directed navigation in the central brain.

## Discussion

Our analysis of the eye-derived pattern of spatial integration by T4 directionally selective neurons unifies two perspectives on fly motion vision: work that has provided insight into the local circuit mechanism for computing directional selectivity in *Drosophila*^14,15,17,41^, and groundbreaking work in larger flies on the sensing of global patterns of optic flow by wide-field lobula plate neurons^9,20,24,25^. Our study thus reconciles several previous findings. Behavioural studies using precise, localized visual stimulation described maximal responses to motion directions aligned with rows on the eye^42,43^, and work in larger flies noted that the local PDs of several lobula plate tangential neurons^44^ reflected the orientation of the hexagonal grid in frontal eye regions^45^. A study of the looming-sensitive LPLC2 cells in *Drosophila* found that these neurons were most sensitive to non-cardinal, diagonal movement directions in the dorso-frontal eye regions, and that LPi interneurons had shifting PDs across the field of view^46^. Work in *Drosophila* found systematic local PD changes in HS and VS neurons^47^, and another study confirmed our predicted non-canonical optimal translation axis with H2 and HS cell recordings^38^. Finally, a study identified T4 and T5 axonal responses that resembled a translation-like pattern, with smoothly varying PDs across lobula plate layers^27^. Our study provides a mechanistic explanation for all of these observations—the missing link between the arrangement of eye facets and the local PDs measured in the lobula plate is the universal sampling rule we discovered for T4 neurons (Fig. 2) that adheres closely to the coordinate system of the eye. Because ON and OFF edges evoke identical local PDs (Extended Data Fig. 1e) in H2, it is expected that T5 neurons exhibit local PD changes that match those of T4 neurons, and these local changes are derived from the eye-to-medulla mapping, where the columnar inputs of T5 neurons are organized^5^. On the basis of our anatomical analysis of the dendritic orientation of T4 neuron types across two EM datasets (Fig. 2 and Extended Data Fig. 3), we find no evidence for additional subtypes of T4 neurons. Furthermore, our analysis, grounded entirely in anatomical measurements, shows good quantitative agreement with H2’s PD tuning (Fig. 4c) without the previously proposed subdivision of T4b neurons^27^ (compared in Extended Data Fig. 7g).

Our analysis of global optic-flow patterns (Fig. 4 and Extended Data Figs. 6 and 7) provides a simple explanation for the observation that HS and VS cell responses simultaneously represent information about both self-rotations and self-translations^48,49^. Together with recent work describing the complete set of lobula plate tangential neurons in *Drosophila*^23^, a clear understanding of optic-flow processing emerges, showing that accurate decoding of self-motion-induced optic flow requires integrating signals across large regions of the fly’s field of view. This detection relies on the layer-specific flow fields mapped in the present study, with most downstream tangential neurons integrating their T4 and T5 inputs over large areas of single layers, underscoring their relevance as functional units for optic flow in the fly brain.

The computation of directional selectivity depends on asymmetrical wiring in the dendrites of T4 and T5 neurons. Each major presynaptic neuron type connects preferentially to T4 and T5 neurons at distinct locations along their dendrite^5^, but the developmental mechanisms that establish this wiring asymmetry are unknown^50^. Our discovery of universal sampling by T4 dendrites—each cell integrating from a unit hexagon of medulla columns—suggests that the core developmental mechanisms are similar across the medulla (and the lobula for T5 neurons) and all types, acting together with a process that established the type-specific dendritic orientation. In support of this proposal, RNA-sequencing studies have shown that all eight T4 and T5 types are very similar transcriptionally, including during development^50–52^. The discovery that all T4 neurons are similar in the appropriate reference frame greatly simplifies the scope of the required explanatory mechanism.

Arthropods with compound eyes, which comprise the majority of described animal species, show a considerable diversity of anatomical specializations, reflecting their diverse visual ecology^35^. Because many features of optic-lobe anatomy—including key cell types involved in motion vision—are conserved across flies^53^, and comparable neurons and brain regions are found across arthropods^54^, the insights uncovered in *Drosophila* might be broadly applicable. Extrapolating from our work, we wonder whether detailed eye maps would make strong predictions about the motion directions sensed by the animal, and thus its behaviour and natural history. This correspondence between the structure of the sensory system and an animal’s behavioural repertoire^55^ underlines the fact that neural computations cannot be considered in isolation, because evolution jointly sculpts the function of the nervous system and the structure of the body.

## Methods

### Anatomical data

#### EM reconstruction

All reconstructions in this manuscript are from a serial-section transmission EM volume of a female *D. melanogaster* FAFB^6^. Following established practices^59^, we manually reconstructed neuron skeletons in the CATMAID environment^60^ (in which 27 laboratories were collaboratively building connectomes for specific circuits, mostly outside of the optic lobe). We also used two auto-segmentations of the same dataset, FAFB-FFN1^61^ and FlyWire^62^, to quickly examine many auto-segmented fragments for neurons of interest. Once a fragment of interest was found, it was imported to CATMAID, followed by manual tracing and identity confirmation.

For the data reported here, we identified and reconstructed a total of 780 Mi1, 38 T4a, 176 T4b, 22 T4c, 114 T4d, 63 TmY5a and one H2 cells. All the columnar neurons could be reliably matched to well-established morphology from Golgi-stained neurons^13^. This reconstruction is based on approximately 1.35 million manually placed nodes. (1) Mi1: we traced the main branches of the M5 and M9–M10 arbors such that the centres of mass of the arbors formed a visually identifiable grid. We used the auto-segmentation to accelerate the search for Mi1 cells wherever there appeared to be a missing point in the grid. After an extensive process, we believed that we had found all of the Mi1 cells in the right optic lobe (Fig. 2c,d). One Mi1 near the neuropil boundary was omitted in later analysis because its centre of mass was clearly ‘off the grid’ established by neighbouring Mi1 cells, despite a complete arbor morphology. (2) T4: we traced their axon terminals in the lobula plate for type identification (each type innervates a specific depth in the lobula plate^19^) and manually reconstructed their complete dendritic morphology to determine their anatomical PD. To sample T4 morphology across the whole eye with a reasonable amount of time and effort, we focused on the T4b (Fig. 2e) and T4d (Extended Data Fig. 2g) types with sufficient density to allow us to interpolate the PDs at each column position. In addition, we chose four locations on the eye: medial (M), anterior dorsal (AD), anterior ventral (AV) and lateral ventral (LV), where we reconstructed about three to four sets of T4 cells and confirmed that the PDs were mostly anti-parallel between T4a and T4b, as well as between T4c and T4d (Extended Data Fig. 6c,d). (3) TmY5a: we searched for cells along the equator and central meridian of the medulla and traced out their main branches to be able to extend (with further interpolation) the columnar structure of the medulla to the lobula (Extended Data Fig. 2f). (4) H2: the neuron was found during a survey^23^ of the LPTCs in the right side of the FAFB brain and was completely reconstructed, including all fine branches in the lobula plate (Fig. 1d and Extended Data Fig. 1b).

In addition, we identified several lamina monopolar cells and photoreceptor cells. (5) Lamina cells, mainly L1, L2, L3 and outer photoreceptor cells (R1–R6), were reconstructed, often making some use of auto-segmented data, to allow for their identification. This helped us to locate the equatorial columns in the medulla that have different numbers of photoreceptor inputs in the corresponding lamina cartridge (Fig. 2c and Extended Data Fig. 2b–d). (6) Inner photoreceptor cells R7 and R8: we searched for R7 and R8 cells throughout the eye, at first as part of a focused study on the targets of these photoreceptors^56^. We extended these reconstructions to complete the medulla map in Fig. 2. We searched for R7 and R8 cells corresponding to each Mi1 cell near the boundary of the medulla. Mi1 cells in columns lacking inner photoreceptors were identified and excluded from further analysis (Fig. 2c). Furthermore, we reconstructed several cells near the central meridian and used the shape of their axons to determine the location of the chiasm (Extended Data Fig. 2e).

#### Generation and imaging of split-GAL4 driver lines

We used the split-GAL4 driver lines SS00809 (ref. ^15^) and SS01010 to drive reporter expression in Mi1 and H2 neurons, respectively. Driver lines and representative images of their expression patterns are available at https://splitgal4.janelia.org/. SS01010 (newly reported here; 32A11-p65ADZp in attP40; 81E05-ZpGdbd in attP2) was identified and constructed using previously described methods and hemidriver lines^63,64^. We used MCFO^65^ for multicolour stochastic labelling. Sample preparation and imaging, performed by the Janelia FlyLight Project Team, were as in previous studies^64,65^. Detailed protocols are available online (https://www.janelia.org/project-team/flylight/protocols under ‘IHC - MCFO’). The antibodies used were as follows: mouse nc82 (1:30; Developmental Studies Hybridoma Bank, nc82-s), rat anti-Flag (DYKDDDDK epitope tag) (1:200; Novus Biologicals, NBP1-06712), rabbit anti-HA tag (1:300; Cell Signal Technologies, 3724S), Cy2 goat anti-mouse (1:600; Jackson ImmunoResearch, 115-225-166), ATTO647N goat anti-rat (1:300; Rockland, 612-156-120) and AF594 donkey anti-rabbit (1:500; Jackson ImmunoResearch, 711-585-152). Images were acquired on Zeiss LSM 710 or 780 confocal microscopes with 63×/1.4 NA objectives at 0.19 × 0.19 × 0.38 μm^3^ voxel size. The reoriented views in Extended Data Fig. 1b and Extended Data Fig. 2a were displayed using VVDviewer (https://github.com/JaneliaSciComp/VVDViewer). This involved manual editing to exclude labelling outside of approximately medulla layers M9–M10 (Extended Data Fig. 2a) or to show only a single H2 neuron (Extended Data Fig. 1b).

### Confocal imaging of a whole fly eye

#### Sample preparation

Flies of the following genotype, *w;19F01-LexA(su(Hw)attP5)/ pJFRC22-10XUAS-IVS-myr::tdt(attP40); Rh3-Gal4/ pJFRC19-13XLexAop2-IVS-myr::GFP(attP2)*, were anaesthetized with CO_2_ and briefly washed with 70% ethanol. Heads were isolated, their proboscis removed under 2% paraformaldehyde, phosphate-buffered saline (PBS) and 0.1% Triton X-100 (PBS-T), and fixed in this solution overnight at 4 °C. After washing with PBS-T, the heads were bisected along the midline with fine scissors and incubated in PBS with 1% Triton X-100, 3% normal goat serum, 0.5% dimethyl sulfoxide and escin (0.05 mg ml^−1^, Sigma-Aldrich, E1378) containing chicken anti-GFP (1:500; Abcam, ab13970), mouse anti-nc82 (1:50; Developmental Studies Hybridoma Bank) and rabbit anti-DsRed (1:1,000; Takara Bio, 632496) at room temperature with agitation for two days. After a series of three washes (1 h each) in PBS-T, the sections were incubated for another 24 h in the above buffer containing secondary antibodies: Alexa Fluor 488 goat anti-chicken (1:1,000; Thermo Fisher Scientific, A11039), Alexa Fluor 633 goat anti-mouse (1:1,000; Thermo Fisher Scientific, A21050) and Alexa Fluor 568 goat anti-rabbit (1:1,000; Thermo Fisher Scientific, A11011). The samples were then washed four times (one hour each) in PBS and 1% Triton, and post-fixed for four hours in PBS-T and 2% paraformaldehyde. To avoid artefacts caused by osmotic shrinkage of soft tissue, samples were gradually dehydrated in glycerol (2–80%) and then ethanol (20–100%)^66^ and mounted in methyl salicylate (Sigma-Aldrich, M6752) for imaging.

#### Imaging and rendering

Serial optical sections were obtained at 1-µm intervals on a Zeiss 710 confocal microscope with an LD-LCI 25×/0.8 NA objective using 488-nm, 560-nm and 630-nm lasers, respectively. The image in Extended Data Fig. 4a is a reoriented substack projection, processed in Imaris v.10.1 (Oxford Instruments), in which the red channel (560-nm laser) is not shown.

### µCT imaging of whole fly heads

µCT is an X-ray imaging technique similar to medical CT scanning, but with much higher resolution that makes it more suitable for small samples^67^. A 3D data volume set is reconstructed from a series of 2D X-ray images of the physical sample at different angles. The advantage of this method for determining the ommatidia directions (Fig. 3) is that internal details of the eye, such as individual rhabdoms, distinguishable ‘tips’ of the photoreceptors at the boundary between the pseudocone and the neural retina^68^, and the chirality of the outer photoreceptors, can be resolved across the entire intact fly head with isotropic resolution, which is an essential requirement for preserving the geometry of the eye.

#### Sample preparation

On the basis of previously published fixation and staining protocols for a variety of biological models^69^, we undertook extensive testing of fixatives and stains in addition to mounting and immobilizing steps for µCT scanning. The fixatives tested were Bouin’s fluid, alcoholic Bouin’s and 70% ethanol. We tested staining with phosphotungstic acid in water and in ethanol; phosphomolybdic acid in water and in ethanol; Lugol’s iodine solution; and 1% iodine metal dissolved in 100% ethanol. Various combinations of fixatives and stains and variations in times for each were tried. Drying the samples using hexamethyldisilazane did not yield images with the resolution achievable with critical-point-dried samples^69^. Fixing and staining in ethanol-based solutions followed by critical-point drying produced good contrast with excellent reproducibility but unfortunately introduced a lot of sample shrinkage. We eventually decided to omit the critical-point drying step and to directly scan the samples in an aqueous environment. Extra care was taken to immobilize the head to achieve the desired resolution.

Six- to seven-day-old female *D. melanogaster* flies were anaesthetized with CO_2_ and immersed in 70% ethanol. We kept the thorax and abdomen intact and glued to the head, and subsequently used it as an anchor to stabilize the head in an aqueous environment. We confirmed that no glue got on to the head region. The mouthpart and legs were removed to allow for fixative absorption. Samples were fixed in 70% ethanol at room temperature overnight in a 1.5-ml Eppendorf tube with rotation. The ethanol was then replaced with a staining solution of 0.5% phosphotungstic acid in 70% ethanol. Samples remained in the staining solution at room temperature for 7–14 days with rotation.

#### Imaging and reconstruction

The samples were scanned with a Zeiss Xradia Versa XRM 510 μCT scanner. The scanning was done at a voltage of 40 kV and current of 72 µA (power 2.9 W) at 20× magnification with 20-s exposures and a total of 1,601 projections. Images had a pixel size of around 1 µm with camera binning at 2 and reconstruction binning at 1. The Zeiss XRM reconstruction software was used to generate TIFF stacks of the tomographs. Image segmentation and annotation (lenses and photoreceptor tips) were done in Imaris v.10.1 (Oxford Instruments).

### Whole-cell recordings of labelled H2 neurons

#### Electrophysiology

All of the flies used in electrophysiological recordings were from a single genotype: *pJFRC28-10XUAS-IVS-GFP-p10* (ref. ^70^) in attP2 crossed to the H2 driver line SS01010 (see ‘Generation and imaging of split-GAL4 driver lines’). Flies were reared under a 16-h light–8-h dark light cycle at 24 °C. To perform the recordings, two-to-three-day-old female *D. melanogaster* flies were anaesthetized on ice and glued to a custom-built PEEK platform, with their heads tilted down, using a UV cured glue (Loctite 3972) and a high-power UV-curing LED system (Thorlabs CS2010). To reduce brain motion, the two front legs were removed, the proboscis was folded and glued in its socket and muscle 16 (ref. ^71^) was removed from between the antennae. The cuticle was removed from the posterior part of the head capsule using a hypodermic needle (BD PrecisionGlide 26 g × 1/2 in.) and fine forceps. Manual peeling of the perineural sheath using forceps seemed to damage the recording stability. Therefore, the sheath was removed using collagenase (following a previously described method^72^). To prevent contamination, the pipette holder was replaced after collagenase application.

The brain was continuously perfused with an extracellular saline containing 103 mM NaCl, 3 mM KCl, 1.5 mM CaCl_2_.2H_2_O, 4 mM MgCl_2_.6H_2_O, 1 mM NaH_2_PO_4_.H_2_O, 26 mM NaHCO_3_, 5 mM *N*-Tris(hydroxymethyl)-methyl-2-aminoethane-sulfonic acid, 10 mM glucose and 10 mM trehalose, with the osmolarity adjusted to 275 mOsm and bubbled with carbogen throughout the experiment. Patch clamp electrodes were pulled (Sutter P97), pressure polished (ALA CPM2) and filled with an intracellular saline containing 140 mM Kasp, 10 mM HEPES, 1 mM EGTA, 1 mM KCl, 0.1 mM CaCl_2_, 4 mM MgATP, 0.5 mM NaGTP and 5 mM glutathione^73^. Alexa 594 hydrazide (250 μM) was added to the intracellular saline before each experiment to reach a final osmolarity of 265 mOsm, with a pH of 7.3.

Recordings were obtained using a Sutter SOM microscope with a 60× water-immersion objective (60× Nikon CFI APO NIR Objective, 1.0 NA, 2.8-mm WD). Contrast was generated using oblique illumination from an 850-nm LED connected to a light guide positioned behind the fly’s head. Images were acquired using μManager^74^ to allow for automatic contrast adjustment. All recordings were obtained from the left side of the brain. To block visual input from the contralateral side, the right eye was painted with miniature paint (MSP Bones grey primer followed by dragon black). Current clamp recordings were sampled at 20 kHz and low-pass-filtered at 10 kHz using an Axon multiClamp 700B amplifier (National Instrument PCIe-7842R LX50 Multifunction RIO board) and custom LabView (2013 v.13.0.1f2; National Instruments) and MATLAB (MathWorks) software.

#### Visual stimuli

The display used to present visual stimuli to the fly during H2 recordings was a G4 LED arena^75^ configured with a manual rotation axis. The arena covered slightly more than one-half of a cylinder (240° in azimuth and around 50° in elevation) of the fly’s visual field, with the diameter of each pixel subtending about 1.25° on the fly eye. With the limitations of the mounting platform, the microscope objective and access for visually guided electrophysiology, it is not possible to deliver visual stimuli to the fly’s complete field of view. To access the cell body of H2, the head must be pitched downwards. In this configuration, the frontal and dorsal regions are the most natural eye regions to stimulate. To mitigate stimulus distortion caused by the cylindrical arena (and thus better approximate a spherical display), we rotated the arena (by 30°) once during each recording to present stimuli in the equatorial and more dorsal part of the fly’s visual field. In Extended Data Fig. 1d, positions 1, 2, 6, 7 and 9 are presented with one arena rotation angle, and positions 3, 4, 5 and 8 with a second arena position.

Because it was most important to examine variation in local PDs along the elevation in the frontal part of the visual space (see the ideal flow fields in Fig. 1f), we oriented the fly to have the largest visible extent in this region. Visual stimuli were generated using custom-written MATLAB code. We performed two sets of experiments (five flies in set 1 and seven flies in set 2) using the following stimulus protocols.

Experiment set 1

1. Moving grating: square wave gratings with a constant spatial frequency (7 pixels ON, 7 pixels OFF) moving at 1.78 Hz (40-ms steps) were presented in an approximately 26° (21 pixels in diameter) circular window over an intermediate-intensity background. Gratings were presented for three full cycles (1.68 s) with three repetitions at 16 orientations.
2. Moving bars: bright and dark moving bars were presented in both preferred and non-preferred directions for H2 cells (back to front and front to back, respectively). The H2 responses to these trials are not shown.

Experiment set 2

1. Moving grating: same as above, except that gratings were presented for five full cycles (2.8 s) with three repetitions at eight orientations.
2. Moving edges: bright and dark moving edges were presented in the same circular window as above on an intermediate-intensity background. Edges moving at 40-ms steps (around 31° s^−1^) were presented in 16 orientations to accurately measure the local PD of each cell. Stimuli were presented with three repetitions for each condition.
3. Moving bars: bright and dark moving bars were presented in both preferred and non-preferred directions for H2 cells (back to front and front to back, respectively). The H2 responses to these trials are not shown.

Figure 1e shows the response of an example H2 cell (cell 2 in Extended Data Fig. 1d from experiment set 2) to bright moving edges, and the red arrows in Figs. 1f and 4c and Extended Data Figs. 6a and 7g are responses averaged over all seven H2 cells in experiment set 2 for both bright and dark moving edges. The responses from cell 7 at locations 3, 4 and 5 were excluded owing to the declining quality of the recording.

Extended Data Fig. 1c shows the responses from recorded H2 cell 2 in experiment set 2. The grating responses (bottom) show 2 s of the response after the stimulus start.

Extended Data Fig. 1d plots the responses of individual cells: bright and dark edge responses are from the seven recorded cells in experiment set 2; grating responses, locations 2–6 include seven flies from experiment set 2; locations 7–9 include five flies from experiment set 1; location 1 includes flies from both sets.

Extended Data Fig. 1e compares the responses from the seven recorded cells in experiment set 2 and their responses are further detailed in Supplementary Data 1.

The local PD for the H2 cells was determined using the responses to the 16 directions, averaged for both moving edge stimuli. Spikes were extracted from the recorded data and summed per trial, then averaged across repeated presentations. The polar plots (Fig. 1e) represent these averages (relative to baseline firing rate), and the vector sum over all 16 directions is represented by the red arrows in Fig. 1e,f. The subthreshold responses of H2 can also be used to determine the local PD of the neuron, showing good agreement with the directions based on the neuron’s spiking responses (not shown).

#### Determining head orientation

A camera (Point Grey Flea3 with an 8X CompactTL telecentric lens with in-line illumination, Edmund Optics) was aligned to a platform holder using a custom-made target. This allowed us to adjust the camera and platform holder such that when the holder is centred in the camera’s view, both yaw and roll angles are zero. Next, after the fly was glued to the platform, but before the dissection, images were taken from the front to check for the yaw and roll angles of head orientation. If the deviation of the head away from a ‘straight ahead’ orientation was more than 2°, then that fly was discarded. Finally, to measure pitch angle, the holder was rotated ±90°, and images of the fly’s eye were taken on both sides. Head orientation was then measured as previously described^76^. We found that flies were consistently positioned with very similar orientations, such that we could combine the data across flies to produce the summary local PD plots for H2 recordings (Fig. 1f and Extended Data Fig. 1d).

#### Determining the stimuli in the compound eye reference frame

The positions and directions of the visual stimuli are programmed in the LED coordinates of the G4 display. We first transformed the LED coordinates to the lab coordinates using the dimension and rotation angle of the arena. The arena was set at two different rotation angles to maximize the coverage of the fly’s visual field. Then, using the head orientation measurement, we performed another transformation to the compound eye reference frame (Fig. 1f). These transformations map each stimulus into spherical coordinates in the fly eye reference frame, where the subsequent vector operations to determine the local PDs are performed.

Presenting stimuli on an idealized spherical display while recording from neurons in the fly brain is impractical, but it is important to account for any differences. This cylinder–sphere mismatch between the cylindrical LED arena and the spherical compound eye reference frame introduces both scale and angular distortions to the visual stimuli. The largest distortion should occur between positions with the largest elevation difference; for example, between positions 1 and 4. To visualize this distortion, we mapped eight (angularly) uniformly distributed equal-length vectors (representing the motion travelled by, for example, a moving edge stimulus) to six stimulus locations on our display and plotted them (on a Mercator projection) as the fly would observe each (Extended Data Fig. 1f). The difference between positions 1 and 4 seems to be mainly an overall rotation. Because the local PD of H2 is calculated from the neuron’s spike rate and the stimulus’s moving direction (already accounting for this distortion), the crucial feature is that we are uniformly sampling all directions. Consequently, an overall apparent rotation of the stimulus set does not affect the result. Second, the apparent expansion of the vectors at positions 3, 4 and 5 is due to the Mercator projection (chosen for this visualization because it preserves angles); in fact, the vectors closer to the equator appear larger to the flies. Because we rotate the arena, the difference between positions 3 and 4 is comparable to that between positions 1 and 2, which is minimal. To characterize the variation in stimulus speed due to this geometric distortion, we computed the average stimulus amplitudes (Extended Data Fig. 1f) at two extreme positions: position 1 (12.7° on average) and position 4 (11.6° on average), which showed a change of around 7%. We did not correct the stimulus velocity because the velocity differences are small, and we selected our stimulus speed in a regime in which T4 and T5 neurons have broad speed tuning^15,16^.

### Data analysis

#### Mapping medulla columns

We based our map of medulla columns on the principal columnar cell type Mi1 that is found as one per column. Mi1 neurons resemble columns, with processes that do not spread far from the main ‘trunk’ of the neuron. They have a stereotypical pattern of arborization in medulla layers M1, M5 and M9–M10. For each Mi1 cell, we calculated the centres of mass of its arbors in both M5 and M10, and used them as column markers (Fig. 2b,c). The medulla columns do not form a perfectly regular grid—the column arrangement is squeezed along the anterior–posterior direction, and the dorsal and ventral portions shift towards the anterior. Nevertheless, we were able to map all column positions onto a regular grid by visual inspection (Fig. 2d). This was much clearer based on the positions of the M5 column markers, which are more regular and were used as the basis for our grid assignment. We compared the whole cells (across layers) in a neighbourhood for occasional ambiguous cases to confirm our assignment. We then propagated the grid assignment to M10 column markers and used them throughout the paper, because T4 cells received inputs in layer M10.

Establishing a global reference that could be used to compare the medulla map (Fig. 2c) to the eye map (Fig. 3f) was essential, and so we endeavoured to find the ‘equator’ of the eye in both the EM and the μCT dataset. Lamina cartridges in the equatorial region receive more outer photoreceptor inputs (seven or eight compared with the usual six)^11,77^. We traced hundreds of lamina monopolar cells (L1 or L3 cells), with at least one input to each of around 100 Mi1 cells near the equator region, and counted the number of photoreceptor cells in each corresponding lamina cartridge (Extended Data Fig. 2b–d). This allowed us to locate the equatorial region of the medulla (Fig. 2c). The equator in μCT is identified by the chirality of the outer photoreceptors (Fig. 3d). We further identified the ‘central meridian, +*v*’ row, which is roughly the vertical midline. There is some ambiguity in defining +*h* as the equator in Fig. 2d, because there are four rows of ommatidia with eight photoreceptors (points in tan). We opted for one of the middle two rows that intersects with +*v*. We also identified the chiasm region on the basis of the twisting of R7 and R8 photoreceptor cells (Extended Data Fig. 2e), which very nearly aligned with the central meridian.

#### T4 PD

Strahler number (SN) was first developed in hydrology to define the hierarchy of tributaries of a river^28^, and has since been adapted to analyse the branching pattern of a tree graph (Fig. 2f). A dendrite of a neuron can be considered as a tree graph. The smallest branches (leaves of a tree) are assigned with SN = 1. When two branches of SN = *a* and SN = *b* merge into a larger branch, the latter is assigned with SN = max(*a*, *b*) if *a* ≠ *b*, or with SN = *a* + 1 if *a* = b.

We used SN = {2, 3} branches to define the PD because they are the most consistently directional (Extended Data Fig. 3a). SN = 1 branches have a relatively flat angular distribution, so their inclusion would only add noise, rather than signal, to our PD estimate (which we confirmed in preliminary analysis). Furthermore, the scale of the SN = 1 branches (see examples in Fig. 2g or the gallery of reconstructed neurons in the Supplementary Data) is much smaller than the columns, and they are dominated by the ‘last mile’ of neuronal connectivity within the very dense columns and do not contribute to the neuron ‘backbone’.

Most T4 cells we reconstructed have few SN = 4 branches (which are also directional, but too few to be relied on) and rarely have SN = 5 branches. A 3D vector represents each branch. Vector sums are calculated for all SN = {2, 3} branches, which define the directions of the PD vectors (Fig. 2f). We also assigned an amplitude to the PD in addition to its direction. To generate a mass distribution for each T4 dendrite, we resampled the neuron’s skeleton to position the nodes roughly equidistantly (not so after manual tracing). Then, all dendrite nodes were projected onto the PD axis. We define the length of the PD vector using a robust estimator, the distance between the 1st and 99th percentiles of this distribution. The width is a segment orthogonal to the PD vector, with its length similarly defined as PD and without a direction (Fig. 2g).

#### Mapping T4 PDs into the regular grid in the medulla and the eye coordinates using kernel regression

Kernel regression is a type of non-parametric regression, often used when the relationship between the independent and the dependent variables does not follow a specific form. It computes a locally weighted estimation, in which the weights are given by the data themselves. In our case, we used a Gaussian kernel as the weighting function. More specifically, given a set of points *P* in space *A* (for example, medulla columns in anatomical space), a second set of points *Q* in space *B* (for example, ommatidia directions in visual space) and a one-to-one mapping between *P* and *Q*, one can map a new point (for example, a T4 PD vector) in *A* to a location in *B* on the basis of its relationships with respect to *P*, with more weight given to closer neighbours.

We used this method to map PDs from local medulla space to a regular grid in Fig. 2h and to map PDs from medulla space to visual space in Fig. 4b. We verify the accuracy of the regression method with a test described at the end of this section. For mapping to a regular grid, we defined a 2D reference grid with 19 points, which represented the home column (+1) and the second (+6) and third (+12) closest neighbouring columns in a hexagonal grid. For a given T4 neuron, we searched for the same set of neighbouring medulla columns. We flattened these columns and the T4’s PD locally by projecting them onto a 2D plane given by principal component analysis; that is, the plane is perpendicular to the third principal axis. Finally, we used kernel regression to map the PD from the locally flattened 2D medulla space to the 2D reference grid. The difference in mapping to the visual space (Fig. 4b and Extended Data Fig. 7a) is that the regression is from the locally flattened 2D medulla space to a unit sphere in 3D (the space of ommatidia directions).

Kernel regression can also be used as an interpolation method. This method is equivalent to mapping from a space to its scalar or vector field; that is, assigning a value to a new location on the basis of existing values in a neighbourhood. This is how we calculated the PD fields in Fig. 4c and Extended Data Fig. 7b.

In practice, we used the np package^78^ in R, particularly the npregbw function, which determines the width of the Gaussian kernel. Most parameters of the npregbw function were set to default except that: (1) we used the local-linear estimator, regtype = ‘ll’, which we determined performs better near boundaries; (2) we used fixed bandwidth, bwtype= ‘fixed’, for interpolation and the adaptive nearest neighbour method, bwtype= ‘adaptive_nn’, for mapping between two different spaces (for example, from medulla to ommatidia).

Extended Data Fig. 6f quantifies the kernel regression by comparing the medulla columns regressed from the medulla space to the ommatidia space versus their matched positions. The perfect regression would yield no spatial discrepancies between these positions, and the observed residuals are quite small compared with the inter-ommatidial angle. Because PD vectors are defined in reference to the medulla columns, the regression method will project them to visual space with high accuracy. Further details can be found in our GitHub repository and the np package manual.

#### Ommatidia directions

We analysed the µCT volumes in Imaris v.10.1 (Oxford Instruments). We separately segmented out a volume that contained all the lenses and one that contained all the photoreceptor tips. We then used the ‘spot detection’ (based on contrast) algorithm in Imaris to locate the centres of individual lenses and photoreceptor tips, and quality controlled by visual inspection and manual editing. The lens positions are highly regular and can be readily mapped onto a regular hexagonal grid (Extended Data Fig. 5a, directly comparable to the medulla grid in Fig. 2d). With our optimized µCT data, it is also straightforward to match all individual lenses to all individual photoreceptor ‘tips’ in a one-to-one manner, and consequently to compute the ommatidia viewing directions. These directional vectors can be represented as points on a unit sphere (Fig. 3e). We then performed a locally weighted smoothing for points with at least five neighbours: the position of the point itself accounts for 50%, and the average position of its six neighbours accounts for the remaining 50%. This gentle smoothing only affects the positions in the bulk of the eye, leaving the boundary points alone.

Assuming left–right symmetry, we used the lens positions from both eyes to define the visual field’s frontal midline (sagittal plane). Together with the equator, identified by the inversion in the chirality of the outer photoreceptors (Fig. 3c,d), we could then define an eye coordinate system for the fly’s visual space—represented for one eye in Fig. 3e,f. Note that the *z* = 0 plane (*z* is ‘up’ in Fig. 3e) in the coordinate system is defined by lens positions, hence the ‘equator’ ommatidia directions do not necessarily lie in this plane (more easily seen in Fig. 3f). In addition, we defined the ‘central meridian’ line of points (+*v* in Fig. 2e,f and Extended Data Fig. 5a) that divides the whole grid into roughly equal halves. Because this definition is based on the grid structure, this central meridian does not lie on a geographical meridian line in the eye coordinates.

#### Eye map: one-to-one mapping between medulla columns and ommatidia directions

With both medulla columns and ommatidia directions mapped to a regular grid (Fig. 2d and Extended Data Fig. 5a) and equators and central meridians defined, it is straightforward to match these two point sets, starting from the centre outwards. Because the medulla columns are from a fly imaged with EM and ommatidia directions from a different fly imaged with µCT, we do not expect these two point sets to match exactly. Still, we endeavoured to use flies with a very similar total number of ommatidia (and of the same genotype). By matching the points from the centre outwards and relying on anatomical features such as the equator, we minimize the column receptive field discrepancies, especially in the eye’s interior. By construction, this approach yields a more accurate alignment in the interior of the eye and medulla rather than on the boundary of each point set, and is better suited for our purpose of mapping the global organization of T4 PDs. Nonetheless, we minimize the boundary effects by adding auxiliary points along the grid beyond the boundary points, and using them for regressing the original boundary points. The matching at the boundary is somewhat complicated by the existence of medulla columns with no inner photoreceptor (R7 or R8) inputs^57^ (Fig. 2c). In the eye map in Fig. 4a, we noted unmatched points with empty circles, all of which lie on the boundaries (which is why the ommatidia directions in Fig. 3f contain additional points). For these reasons, we expect our alignment to be accurate in the eye’s interior, but there are limits to how accurately the medulla columns and ommatidia directions along the boundary of each dataset—from two separate flies—can be aligned. We also noted the boundary points that did not have enough neighbours for computing the inter-ommatidial angles, the shear angles or the aspect ratios in Fig. 3h,i and Extended Data Fig. 5c,d. Of note, our main discoveries about the universal sampling of medulla columns (Fig. 2), and the strong relationship between T4 PDs and the shear angle of ommatidia hexagons (comparing Fig. 3i with Fig. 4d) are well supported by the anatomy of the bulk of the eye and do not depend on perfect matching across datasets or the particular fly used to construct the eye map (Extended Data Fig. 4f,g).

#### Grid convention: regular versus irregular, and hexagonal versus square

Facet lenses of the fly’s eye are arranged in an almost regular hexagonal grid. However, the medulla columns are squeezed along the anterior–posterior direction and more closely resemble a square grid tilted at 45° (Extended Data Fig. 5e). This difference can also be seen by comparing the aspect ratios (Extended Data Fig. 5c,d). To preserve these anatomical features, we mapped the medulla columns and T4 PDs onto a regular square grid (tilted by 45°; see, for example, Fig. 2d,h) and the ommatidia directions onto a regular hexagonal grid (Extended Data Fig. 5a).

#### Mercator and Mollweide projections

For presenting spherical data, the Mercator projection is more common, but we prefer the Mollweide projection because it produces smaller distortion near the poles, whereas the Mercator projection has singularities at the poles. The Mollweide projection thus provides a more intuitive representation of spatial coverage. On the other hand, the Mercator projection preserves the angular relationships (conformal) and is more convenient for reading out angular distributions, which is why we use it for presenting the H2 data (Fig. 1f and Extended Data Fig. 1e,f). Otherwise, we present the Mollweide projections in the main figures and provide the Mercator version for some plots (Extended Data Figs. 5f–j, 6a and 7c,g). See Extended Data Fig. 4c for a comparison between these two projections.

#### Ideal optic-flow fields

Following the classic framework for the geometry of optic flow^79^, we calculate the optic-flow field for a spherical sampling of visual space under the assumption that all objects are at an equal distance from the fly (only relevant for translational movements). With ommatidia directions represented by unit vectors in 3D, the optic-flow field induced by translation is computed as the component of the inverse of the translation vector (because motion and optic flow are ‘opposite’) perpendicular to the ommatidia directions (also known as a vector rejection). The flow field induced by rotation is computed as the cross product between the ommatidia directions and the rotation vector. Because the motion perceived by the fly would be the opposite of the induced motion, the flow field is the reverse of the ones described above (Fig. 4e). The angles between T4 PDs and ideal optic-flow fields at each ommatidia direction are computed for subsequent comparisons between various optic-flow fields (Fig. 4f,g and Extended Data Fig. 7e,f).

We performed a grid search to determine the optimal axis of movement (minimal average errors) for a given PD field. We defined 10,356 axes on the unit sphere (roughly 1° sampling) and generated optic-flow fields induced by translations and rotations along these axes. We compared all of these optic-flow fields and the PD fields for T4b and T4d to determine the axes with minimal average angular differences (Extended Data Fig. 6e). These are the optimal axes in Fig. 4f–i and Extended Data Figs. 6e and 7f.

### Data analysis and plotting conventions

All histograms are smoothed as a kernel density estimation. To set the scale of each histogram plot, we show a scale bar on the left-hand side that spans from zero at the bottom to the height of a uniform distribution.

All 2D projections (Mollweide or Mercator) are such that the right half (azimuth > 0) represents the right-side visual field of the fly (looking from inside out). The medulla grid and the ommatidia grid are left–right flipped because of the optic chiasm. The top half (elevation > 0) represents the dorsal visual field. The boxed label in the lower right corner of each plot of mapped points indicates the space (med or eye) and representation (anat, 3D, Moll or Merc) used (anat is short for anatomical, indicating that the data are shown in the ‘native’ coordinates of the anatomical dataset).

Animations (Supplementary Videos 1 and 2) were created in Blender (v.4.2)^80^ and using the Python package NAVis (v.1.10.0)^81^.

### Reporting summary

Further information on research design is available in the Nature Portfolio Reporting Summary linked to this article.

## Online content

Any methods, additional references, Nature Portfolio reporting summaries, source data, extended data, supplementary information, acknowledgements, peer review information; details of author contributions and competing interests; and statements of data and code availability are available at 10.1038/s41586-025-09276-5.

## Supplementary information

Supplementary Data 1 H2 directional tuning from individual recorded cells. The angular tuning of the n=7 recorded H2 cells, whose summarized preferred direction (PD) tuning is plotted in Fig. 1f, Extended Data Fig. 1d,e. and Fig. 4c, plotted for each cell and each recorded position. Bright and Dark edge responses to 16 directions of motion are plotted separately as green and magenta, respectively, and the PD, as the vector sum of the responses to each stimulus type, is shown with the larger dot. The black circle indicates the baseline firing rate, and the scale is indicated for each row.

Reporting Summary

Supplementary Data 2–5 Galleries of T4 neurons with PDs. All T4 neurons reconstructed in the FAFB dataset: 38 T4a, 176 T4b, 22 T4c, 114 T4d, are plotted similarly as in Fig. 2g. Using the eye map established in Fig. 4a, we include the position (elevation and azimuth angles) in the eye coordinate. The angle between T4’s PD and the local meridian line is computed instead of using the +v-axis as the reference, as in Fig. 2g. The meridian line is defined as the direction line going from the south pole to the north pole in the eye reference frame (often close to the +v-axis). The cell and surrounding columns are also aligned such that the vertical direction in the plot coincides with the meridian direction. A summary of the Strahler number analysis for each cell is included.

Supplementary Video 1 Summary of *Drosophila* eye map, enabling the projection of the compound eye’s visual space into the neural circuits of the optic lobe. Whole-head µCT scan with overlaid EM-reconstructed neurons, showing the columnar structure of the compound eye and optic lobe. Lens-photoreceptor tip pairs determined ommatidia directions. Medulla columns were defined as the Mi1 cells’ arbor in layer M10. Finally, we established an eye map: a 1-to-1 mapping between ommatidia directions and medulla columns.

Supplementary Video 2 Illustration of how the dendritic orientation of T4 neurons facilitates motion detection in different directions. There are four types of T4 cells, innervating four distinct layers in the lobula plate. A T4 cell’s preferred direction (PD) is computed based on its dendritic arborization pattern. PDs can be mapped to eye coordinates using the eye map defined in Supplementary Video 1. In the central eye region, the four T4 types are well aligned with directions of motion in the four cardinal directions (forward, backward, up, and down).

## Data Availability

EM-reconstructed neurons in the FAFB dataset are available from the public CATMAID server: https://fafb.catmaid.virtualflybrain.org/. FAFB-FFN1 automatic segmentation can be accessed at https://fafb-ffn1.storage.googleapis.com/landing.html. FAFB-Flywire automatic segmentation can be accessed at https://flywire.ai. The male brain optic-lobe dataset can be accessed at https://neuprint.janelia.org/?dataset=optic-lobe:v1.1. Flylight images for the split-GAL4 line used are available on the FlyLight website: https://splitgal4.janelia.org/cgi-bin/splitgal4.cgi. The electrophysiological recordings are available at 10.25378/janelia.28462100.v1. µCT and confocal stack data are available at 10.25378/janelia.29111339.v1.

## Code availability

Analysis and plotting code are available on the accompanying GitHub repository: https://github.com/reiserlab/eyemap_T4. Our data analysis used these software packages: MATLAB (MathWorks), R^82^, RStudio (Posit Software) and the primary R packages natverse (v.0.2.4)^83^, tidyverse (v.2.0.0)^84^ and np (v0.67-17)^78^. We refer readers to the above-mentioned GitHub repository for detailed information on all software packages used in the analysis.
