# Supplementary material for: Eye structure shapes neuron function in Drosophila motion vision
Source: Nature. 2025 Jul 23;646(8083):135–42. doi: 10.1038/s41586-025-09276-5 (PMC12488493; doi:10.1038/s41586-025-09276-5)

nature portfolio

https://doi.org/10.1038/s41586-025-09276-5

Supplementary information

Eye structure shapes neuron function in *Drosophila* motion vision

In the format provided by the
authors and unedited

H2 directional tuning from individual recorded cells
(summarized in ED Fig. 1D)

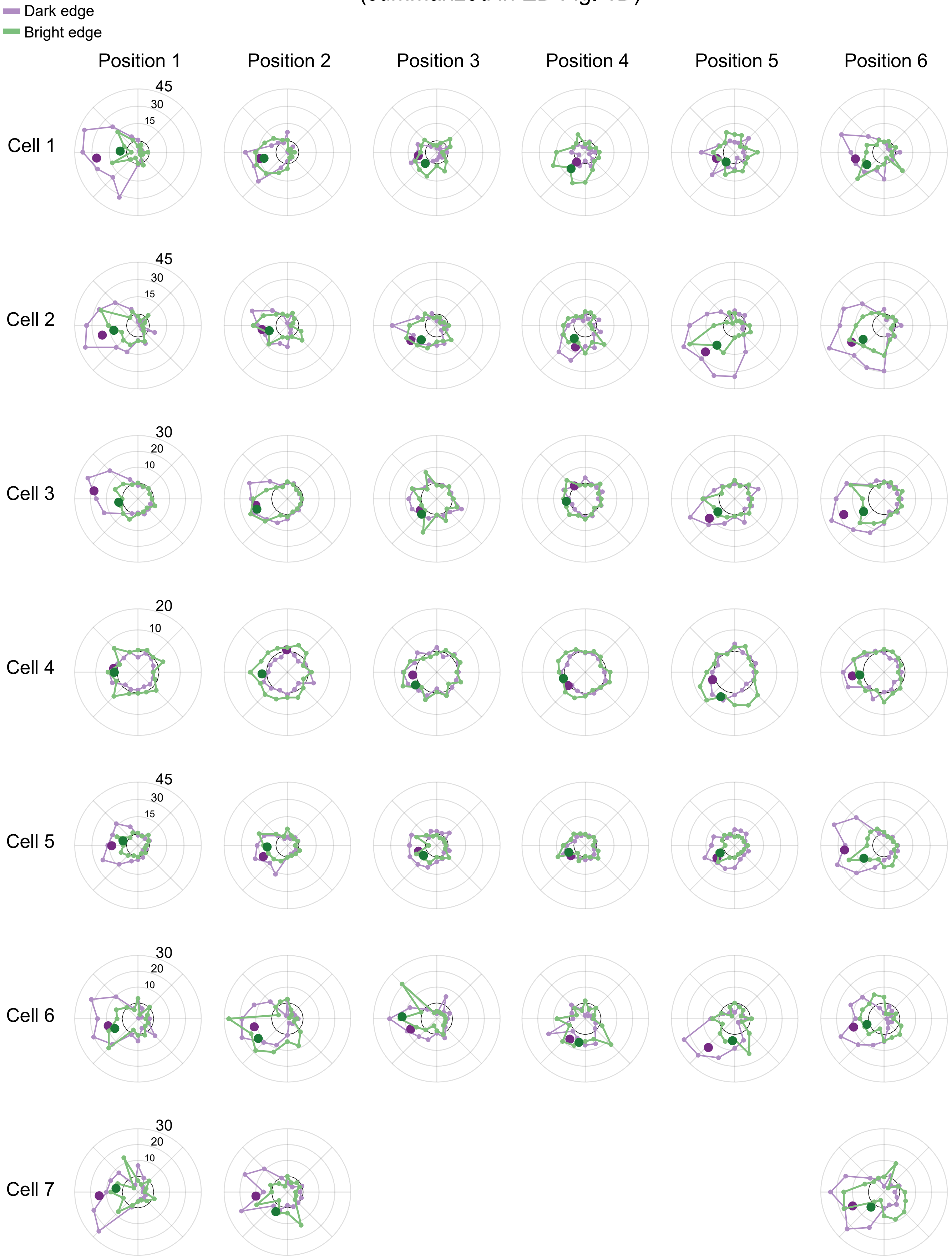

Supplement: Supplementary file 1 — H2 directional tuning from individual recorded cells. The angular tuning of the n=7 recorded H2 cells, whose summarized preferred direction (PD) tuning is plotted in Fig. 1f, Extended Data Fig. 1d,e. and Fig. 4c, plotted for each cell and each recorded position. Bright and Dark edge responses to 16 directions of motion are plotted separately as green and magenta, respectively, and the PD, as the vector sum of the responses to each stimulus type, is shown with the larger dot. The black circle indicates the baseline firing rate, and the scale is indicated for each row. [file 41586_2025_9276_MOESM1_ESM.pdf]
