# Supplementary material for: Eye structure shapes neuron function in Drosophila motion vision
Source: Nature. 2025 Jul 23;646(8083):135–42. doi: 10.1038/s41586-025-09276-5 (PMC12488493; doi:10.1038/s41586-025-09276-5)
Supplement: Supplementary file 2 — Reporting Summary [file 41586_2025_9276_MOESM2_ESM.pdf]

nature portfolio

Corresponding author(s): Michael B. Reiser

Last updated by author(s): May 29, 2025

Reporting Summary

Nature Portfolio wishes to improve the reproducibility of the work that we publish. This form provides structure for consistency and transparency in reporting. For further information on Nature Portfolio policies, see our Editorial Policies and the Editorial Policy Checklist.

Statistics

For all statistical analyses, confirm that the following items are present in the figure legend, table legend, main text, or Methods section.

n/a	Confirmed	
☐	☒	The exact sample size (*n*) for each experimental group/condition, given as a discrete number and unit of measurement
☐	☒	A statement on whether measurements were taken from distinct samples or whether the same sample was measured repeatedly
☐	☒	The statistical test(s) used AND whether they are one- or two-sided<br>*Only common tests should be described solely by name; describe more complex techniques in the Methods section.*
☒	☐	A description of all covariates tested
☒	☐	A description of any assumptions or corrections, such as tests of normality and adjustment for multiple comparisons
☐	☒	A full description of the statistical parameters including central tendency (e.g. means) or other basic estimates (e.g. regression coefficient) AND variation (e.g. standard deviation) or associated estimates of uncertainty (e.g. confidence intervals)
☐	☒	For null hypothesis testing, the test statistic (e.g. *F*, *t*, *r*) with confidence intervals, effect sizes, degrees of freedom and *P* value noted<br>*Give P values as exact values whenever suitable.*
☒	☐	For Bayesian analysis, information on the choice of priors and Markov chain Monte Carlo settings
☒	☐	For hierarchical and complex designs, identification of the appropriate level for tests and full reporting of outcomes
☒	☐	Estimates of effect sizes (e.g. Cohen's *d*, Pearson's *r*), indicating how they were calculated

Our web collection on statistics for biologists contains articles on many of the points above.

Software and code

Policy information about availability of computer code

Data collection	Manual EM reconstruction was carried out in a CATMAID (https://catmaid.readthedocs.io/en/stable/) environment. Split-Gal4 driver line images and whole fly eye images were acquired on Zeiss LSM 710 or 780 confocal microscope with accompanying Zeiss software. Micro-CT images of whole fly heads were acquired with Zeiss Xradia Versa XRM500 microCT scanner and the accompanying Zeiss XRM reconstruction software. Whole cell recordings of labeled H2 neurons were sampled with LabView (2013 v.13.0.1f2; National Instruments. Neurons visualized for recordings using Micro-Manager (v2.0.0).
Data analysis	EM reconstruction data was analyzed with custom code in R (4.4.1) and RStudio (2024.12.1 Build 563), using mainly the following R packages: natverse (v0.2.4), tidyverse (v2.0.0), and np (v0.67-17). Light microscopy stacks were processed and displayed using VVDviewer (https://github.com/JaneliaSciComp/VVDViewer) and Imaris v10.1 (Oxford Instruments). Micro-CT stacks were segmented and annotated in Imaris v10.1. Animations were created in Blender and also using a Python package navis. Electrophysiological data were analyzed and plotted in Matlab (Mathworks, 2024 version). Analysis and plotting code are available on the accompanying GitHub repository: https://github.com/reiserlab/eyemap_T4.

For manuscripts utilizing custom algorithms or software that are central to the research but not yet described in published literature, software must be made available to editors and reviewers. We strongly encourage code deposition in a community repository (e.g. GitHub). See the Nature Portfolio guidelines for submitting code & software for further information.

Data

Policy information about availability of data

All manuscripts must include a data availability statement. This statement should provide the following information, where applicable:

- Accession codes, unique identifiers, or web links for publicly available datasets
- A description of any restrictions on data availability
- For clinical datasets or third party data, please ensure that the statement adheres to our policy

EM reconstructed neurons in the FAFB dataset available from the public CATMAID server: https://catmaid.virtualflybrain.org. FAFB-FFN1 automatic segmentation accessed via https://fafb-ffn1.storage.googleapis.com/landing.html. FAFB-Flywire automatic segmentation accessed via https://flywire.ai. Male Brain Optic lobe data set accessed via https://neuprint.janelia.org/?dataset=optic-lobe:v1.1. Flylight images for the plit-GAL4 line used are available on the FlyLight website: https://splitgal4.janelia.org/cgi-bin/splitgal4.cgi. The electrophysiological recordings are available: https://doi.org/10.25378/janelia.28462100.v1. µCT and confocal stack available from: https://doi.org/10.25378/janelia.29111339.v1.

Research involving human participants, their data, or biological material

Policy information about studies with human participants or human data. See also policy information about sex, gender (identity/presentation), and sexual orientation and race, ethnicity and racism.

Reporting on sex and gender	N/A
Reporting on race, ethnicity, or other socially relevant groupings	N/A
Population characteristics	N/A
Recruitment	N/A
Ethics oversight	N/A

Note that full information on the approval of the study protocol must also be provided in the manuscript.

Field-specific reporting

Please select the one below that is the best fit for your research. If you are not sure, read the appropriate sections before making your selection.

☒ Life sciences ☐ Behavioural & social sciences ☐ Ecological, evolutionary & environmental sciences

For a reference copy of the document with all sections, see nature.com/documents/nr-reporting-summary-flat.pdf

Life sciences study design

All studies must disclose on these points even when the disclosure is negative.

Sample size	No sample size calculation was performed. There is only one full brain EM volume (female) and a male brain optic lobe dataset available at the time of this study. However, we have reconstructed >1000 neurons in FAFB, thereby sampling the diversity of cell morphology within this single EM volume. Micro-CT is a novel but rather expensive technique for imaging small-volume samples. We were able to acquire high-quality scans of 3 flies that have approximately the same number of facet lenses as the fly imaged with EM. As we show in the manuscript, we did not observe any substantial variation, in the critical eye measurements we use for our analyses and conclusions, between these 3 independent scans. The electrophysiology experiments include 12 H2 cells, each from a different fly.
Data exclusions	There is only one reconstructed Mi1 neurons that's excluded from the analyses. We stated the reason in the method section: "One Mi1 near the neuropil boundary was omitted in later analysis because its center-of-mass was clearly “off the grid” established by neighboring Mi1 cells despite a complete arbor morphology."<br>For electrophysiological experiments, 2 cell recordings were excluded from the analysis due to visual display malfunction that caused excessive noise (flickers that induced strong responses).
Replication	All major experimental findings were successfully replicated across independent preparations. Within the FAFB data set, our conclusions are drawn based on hundreds of cells (that were reconstructed independently by more than 6 people). Three Micro-CT scans of different individual animals show very similar results. An independent replication of the distribution of T4 PDs based on dendritic morphology was carried out using the data from the male brain optic lobe. H2 directional tuning was successfully recorded from 12 individual flies across two experimental sets (5 flies in set #1, 7 flies in set #2) using different stimulus protocols.
Randomization	This is not relevant to this study since there is no grouping of samples.
Blinding	Not relevant since there is no grouping.

Reporting for specific materials, systems and methods

We require information from authors about some types of materials, experimental systems and methods used in many studies. Here, indicate whether each material, system or method listed is relevant to your study. If you are not sure if a list item applies to your research, read the appropriate section before selecting a response.

Materials & experimental systems

n/a	Involved in the study
☐	☒ Antibodies
☒	☐ Eukaryotic cell lines
☒	☐ Palaeontology and archaeology
☐	☒ Animals and other organisms
☒	☐ Clinical data
☒	☐ Dual use research of concern
☒	☐ Plants

Methods

n/a	Involved in the study
☒	☐ ChIP-seq
☒	☐ Flow cytometry
☒	☐ MRI-based neuroimaging

Antibodies

Antibodies used

nc82 – Mouse α-bruchpilot, Developmental Studies Hybridoma Bank. # 82-s;
Rat α-FLAG Tag (DYKDDDDK Epitope Tag). Novus Biologicals. # NBP1-06712;
Rabbit α-HA Tag. Cell Signal Technologies. # 3724S;
Cy2 Goat α-Mouse, Jackson Immuno Research. # 115-225-166;
ATTO647N Goat α-Rat, Rockland. # 612-156-120;
AF594 Donkey α-Rabbit, Jackson Immuno Research. # 711-585-152;
chicken anti-GFP, Abcam #ab 13970;
rabbit anti-DsRed, TaKaRa Bio USA, #632496;
Alexa Fluor 488 goat anti-chicken, Thermo Fisher #A11039;
Alexa Fluor 633 goat anti-mouse, Thermo Fisher #A21050;
Alexa Fluor 568 goat anti-rabbit, Thermo Fisher #A11011

Validation

All antibodies used in this study have been extensively used and validated previously (https://www.janelia.org/project-team/flylight/protocols), but some additional details are provided:
nc82 – Mouse α-bruchpilot, Buchner E, Neuron 49.6 (2006 Mar 16): 833-44.
Rat α-FLAG Tag (DYKDDDDK Epitope Tag), see "https://www.novusbio.com/primary-antibodies/dykddddk-epitope-tag?host=Rat'
Rabbit α-HA Tag. "This antibody has been validated using SimpleChIP® Enzymatic Chromatin IP Kits." from supplier website.
rabbit anti-RFP, see "https://www.takarabio.com/documents/Certificate%20of%20Analysis/632496/632496-101717.pdf"
chicken anti-GFP, see "https://www.abcam.com/GFP-antibody-ab13970.html"

Animals and other research organisms

Policy information about studies involving animals; ARRIVE guidelines recommended for reporting animal research, and Sex and Gender in Research

Laboratory animals	female drosophila melanogaster, wild type, and split-GAL4 driver lines SS00809 and SS01010
Wild animals	no wild animal involved
Reporting on sex	female flies used (and documented) in all experiments since the EM data is acquired on a female fly; comparison data from the male optic lobe data set, clearly stated as such.
Field-collected samples	no field collection involved
Ethics oversight	no ethics oversight required.

Note that full information on the approval of the study protocol must also be provided in the manuscript.

Plants

Seed stocks	N/A
Novel plant genotypes	N/A
Authentication	N/A
