# Supplementary material for: Eye structure shapes neuron function in Drosophila motion vision
Source: Nature. 2025 Jul 23;646(8083):135–42. doi: 10.1038/s41586-025-09276-5 (PMC12488493; doi:10.1038/s41586-025-09276-5)
Supplement: Supplementary file 3 — Galleries of T4 neurons with PDs. All T4 neurons reconstructed in the FAFB dataset: 38 T4a, 176 T4b, 22 T4c, 114 T4d, are plotted similarly as in Fig. 2g. Using the eye map established in Fig. 4a, we include the position (elevation and azimuth angles) in the eye coordinate. The angle between T4’s PD and the local meridian line is computed instead of using the +v-axis as the reference, as in Fig. 2g. The meridian line is defined as the direction line going from the south pole to the north pole in the eye reference frame (often close to the +v-axis). The cell and surrounding columns are also aligned such that the vertical direction in the plot coincides with the meridian direction. A summary of the Strahler number analysis for each cell is included. [file 41586_2025_9276_MOESM3_ESM.zip › gallery_T4a.pdf]

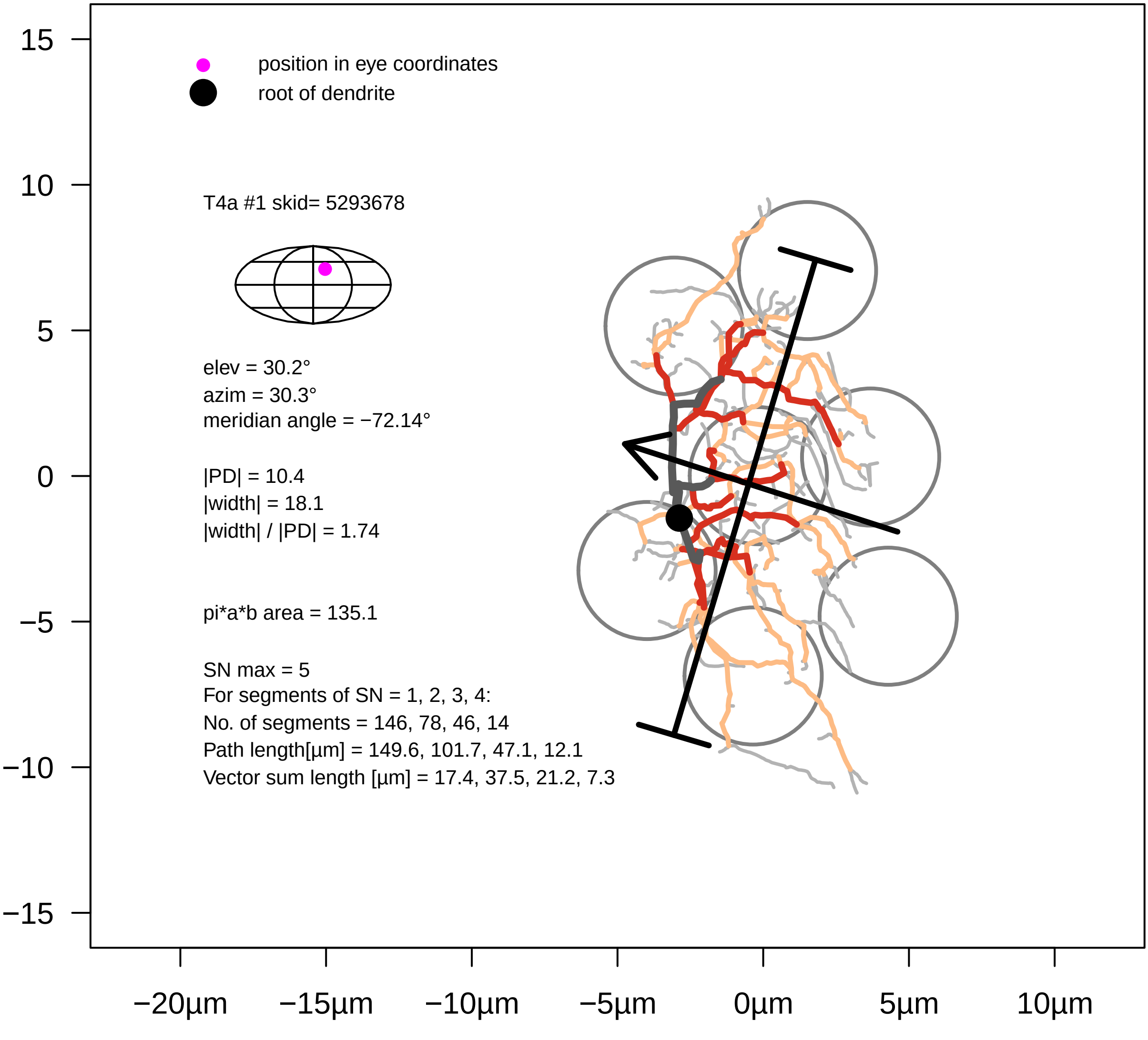

15
10
5
0
−5
−10
−15
−20µm
−15µm
−10µm
−5µm
0µm
5µm
10µm
position in eye coordinates
root of dendrite
T4a #1 skid= 5293678
elev = 30.2°
azim = 30.3°
meridian angle = −72.14°
|PD| = 10.4
|width| = 18.1
|width| / |PD| = 1.74
pi*a*b area = 135.1
SN max = 5
For segments of SN = 1, 2, 3, 4:
No. of segments = 146, 78, 46, 14
Path length[µm] = 149.6, 101.7, 47.1, 12.1
Vector sum length [µm] = 17.4, 37.5, 21.2, 7.3

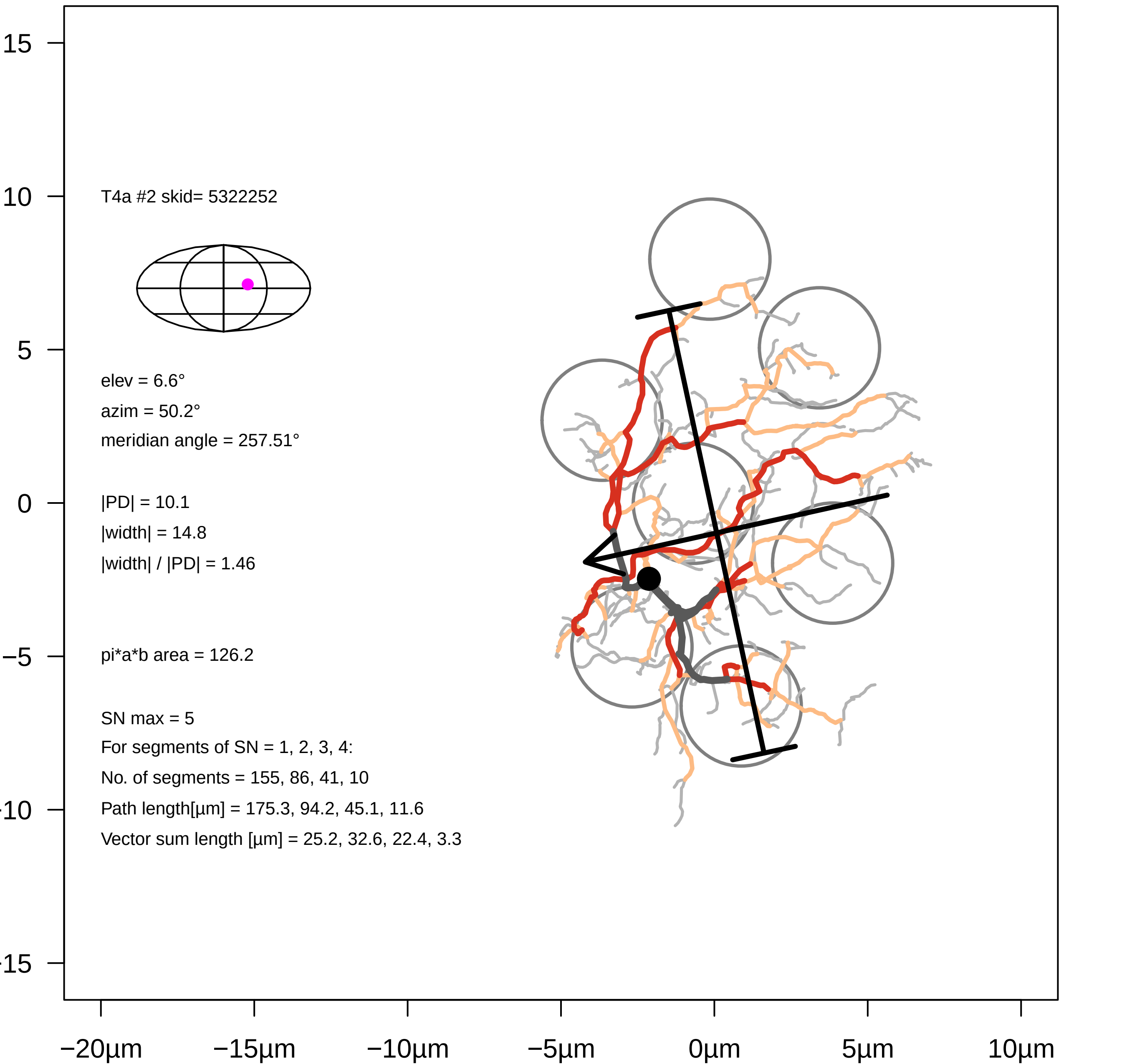
15
10
5
0
−5
-10
-15
−20µm
−15µm
−10µm
−5µm
0µm
5µm
10µm
T4a #2 skid= 5322252
elev = 6.6°
azim = 50.2°
meridian angle = 257.51°
|PD| = 10.1
|width| = 14.8
|width| / |PD| = 1.46
pi*a*b area = 126.2
SN max = 5
For segments of SN = 1, 2, 3, 4:
No. of segments = 155, 86, 41, 10
Path length[µm] = 175.3, 94.2, 45.1, 11.6
Vector sum length [µm] = 25.2, 32.6, 22.4, 3.3

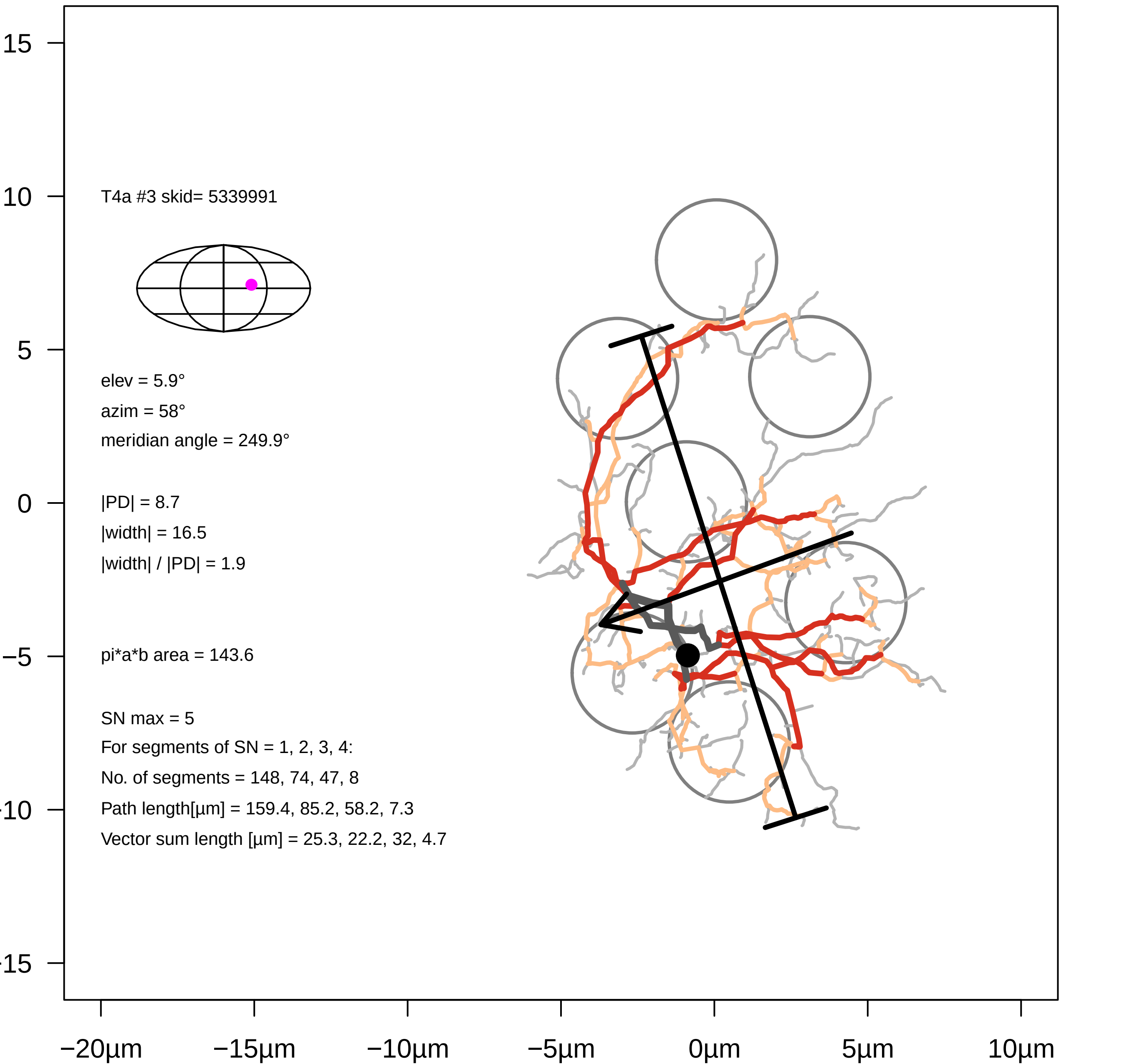
T4a #3 skid= 5339991
elev = 5.9°
azim = 58°
meridian angle = 249.9°
|PD| = 8.7
|width| = 16.5
|width| / |PD| = 1.9
pi*a*b area = 143.6
SN max = 5
For segments of SN = 1, 2, 3, 4:
No. of segments = 148, 74, 47, 8
Path length[µm] = 159.4, 85.2, 58.2, 7.3
Vector sum length [µm] = 25.3, 22.2, 32, 4.7
15
10
5
0
−5
−10
−15
−20µm
−15µm
−10µm
−5µm
0µm
5µm
10µm

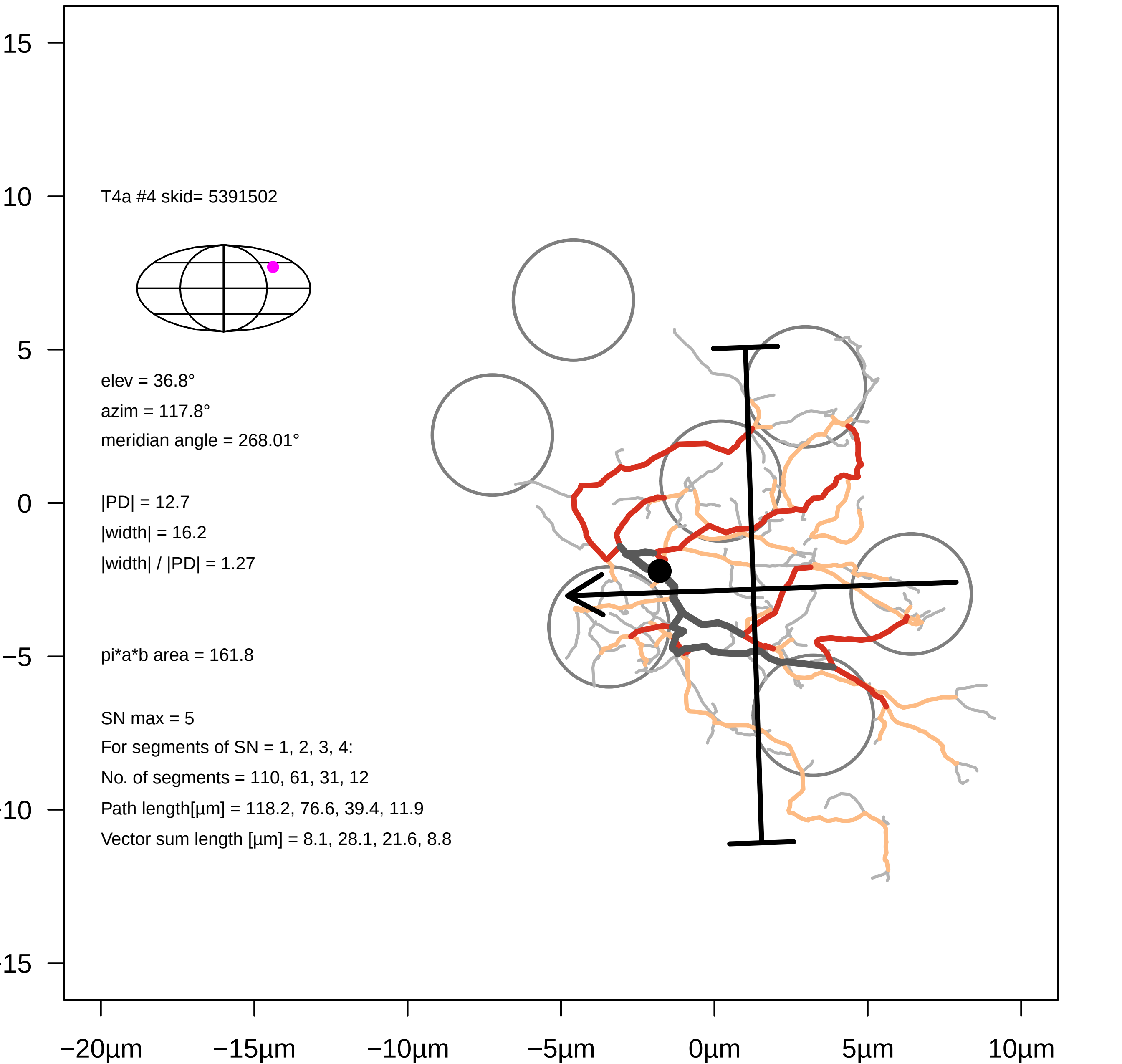

T4a #4 skid= 5391502
elev = 36.8°
azim = 117.8°
meridian angle = 268.01°
|PD| = 12.7
|width| = 16.2
|width| / |PD| = 1.27
pi*a*b area = 161.8
SN max = 5
For segments of SN = 1, 2, 3, 4:
No. of segments = 110, 61, 31, 12
Path length[µm] = 118.2, 76.6, 39.4, 11.9
Vector sum length [µm] = 8.1, 28.1, 21.6, 8.8
15
10
5
0
−5
−10
−15
−20µm
−15µm
−10µm
−5µm
0µm
5µm
10µm

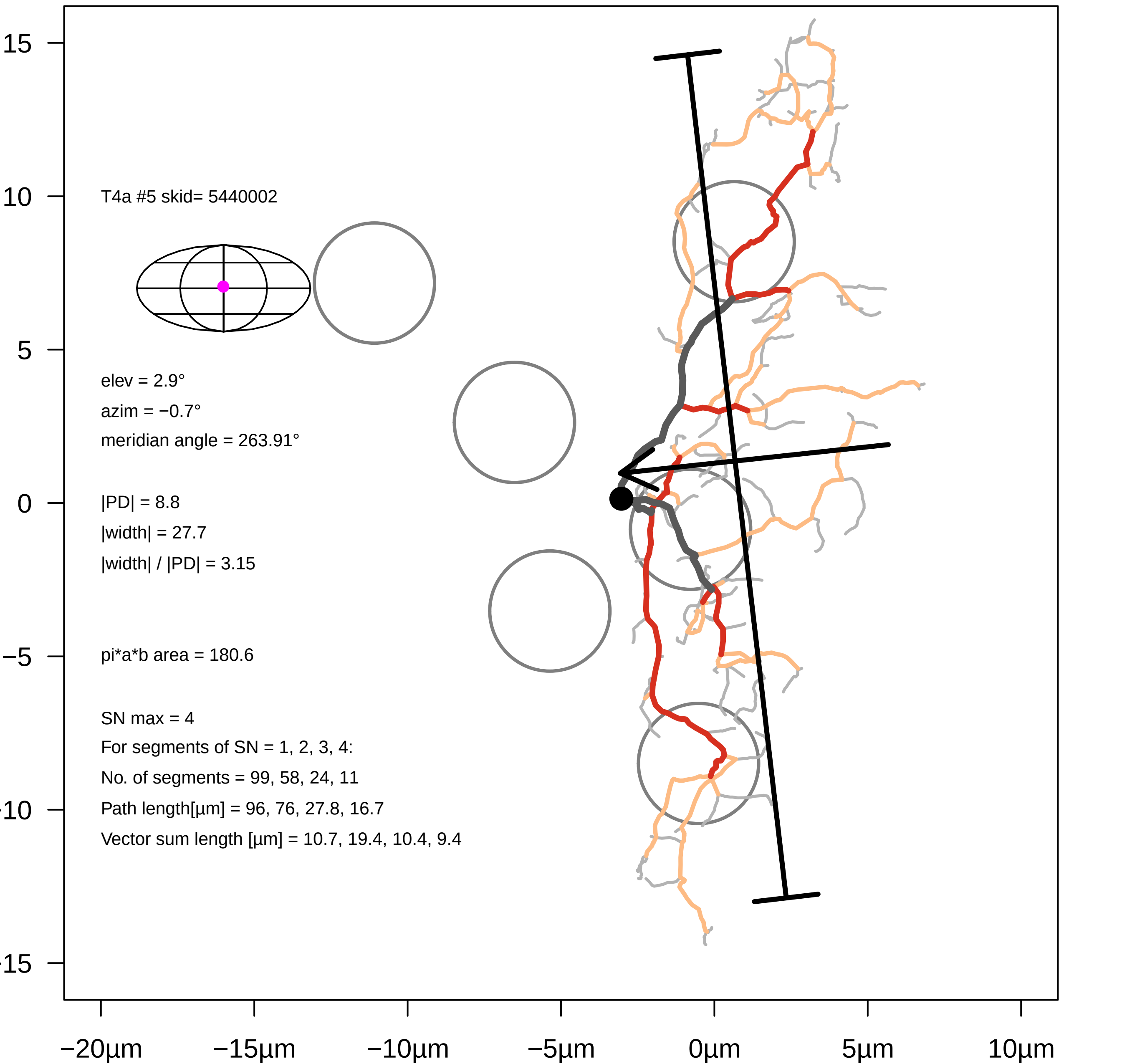

15
10
5
0
−5
-10
-15
−20µm
−15µm
−10µm
−5µm
0µm
5µm
10µm
T4a #5 skid= 5440002
elev = 2.9°
azim = −0.7°
meridian angle = 263.91°
|PD| = 8.8
|width| = 27.7
|width| / |PD| = 3.15
pi*a*b area = 180.6
SN max = 4
For segments of SN = 1, 2, 3, 4:
No. of segments = 99, 58, 24, 11
Path length[µm] = 96, 76, 27.8, 16.7
Vector sum length [µm] = 10.7, 19.4, 10.4, 9.4

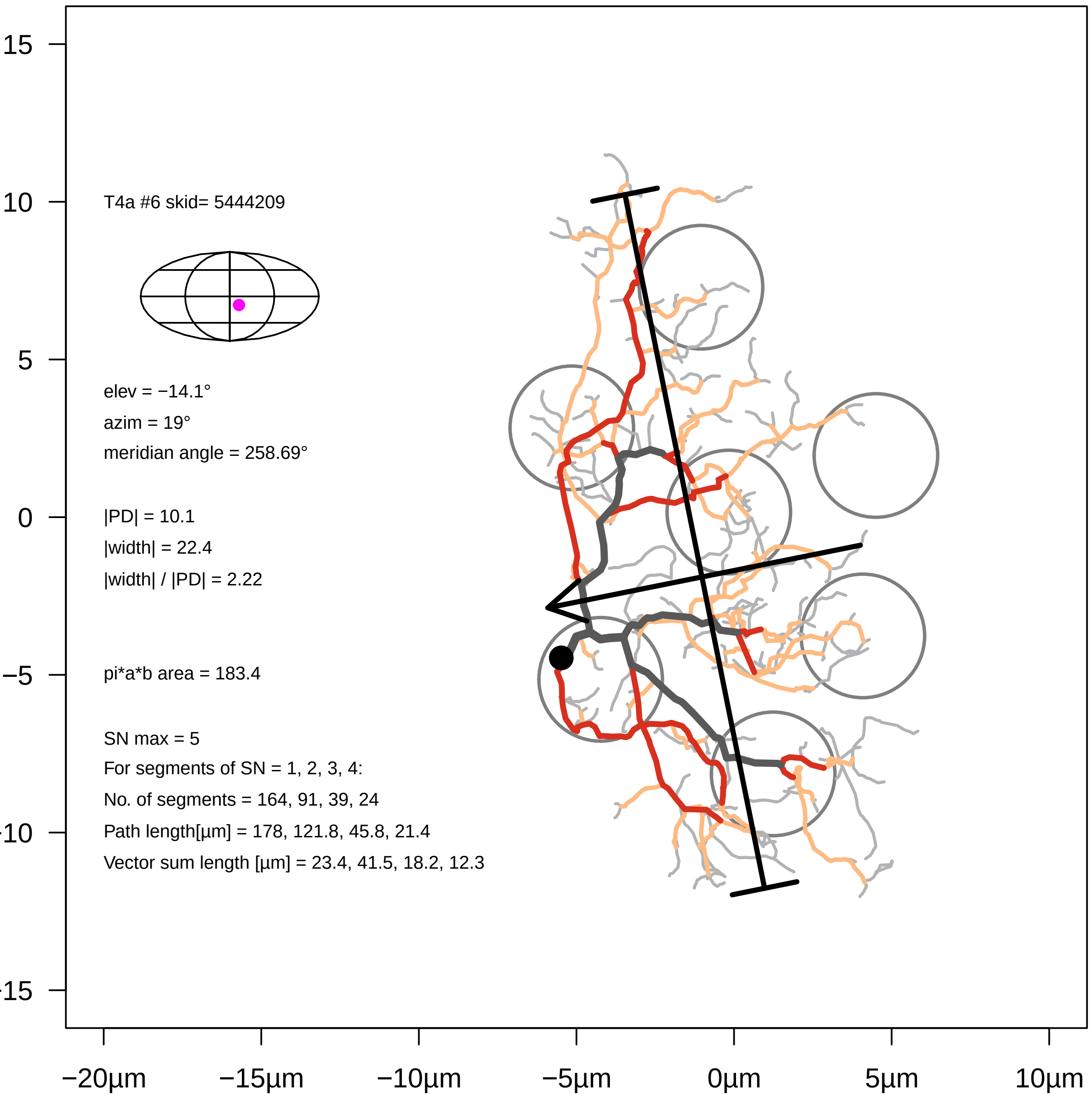

T4a #6 skid= 5444209
elev = −14.1°
azim = 19°
meridian angle = 258.69°
|PD| = 10.1
|width| = 22.4
|width| / |PD| = 2.22
pi*a*b area = 183.4
SN max = 5
For segments of SN = 1, 2, 3, 4:
No. of segments = 164, 91, 39, 24
Path length[µm] = 178, 121.8, 45.8, 21.4
Vector sum length [µm] = 23.4, 41.5, 18.2, 12.3
15
10
5
0
−5
−10
−15
−20µm
−15µm
−10µm
−5µm
0µm
5µm
10µm

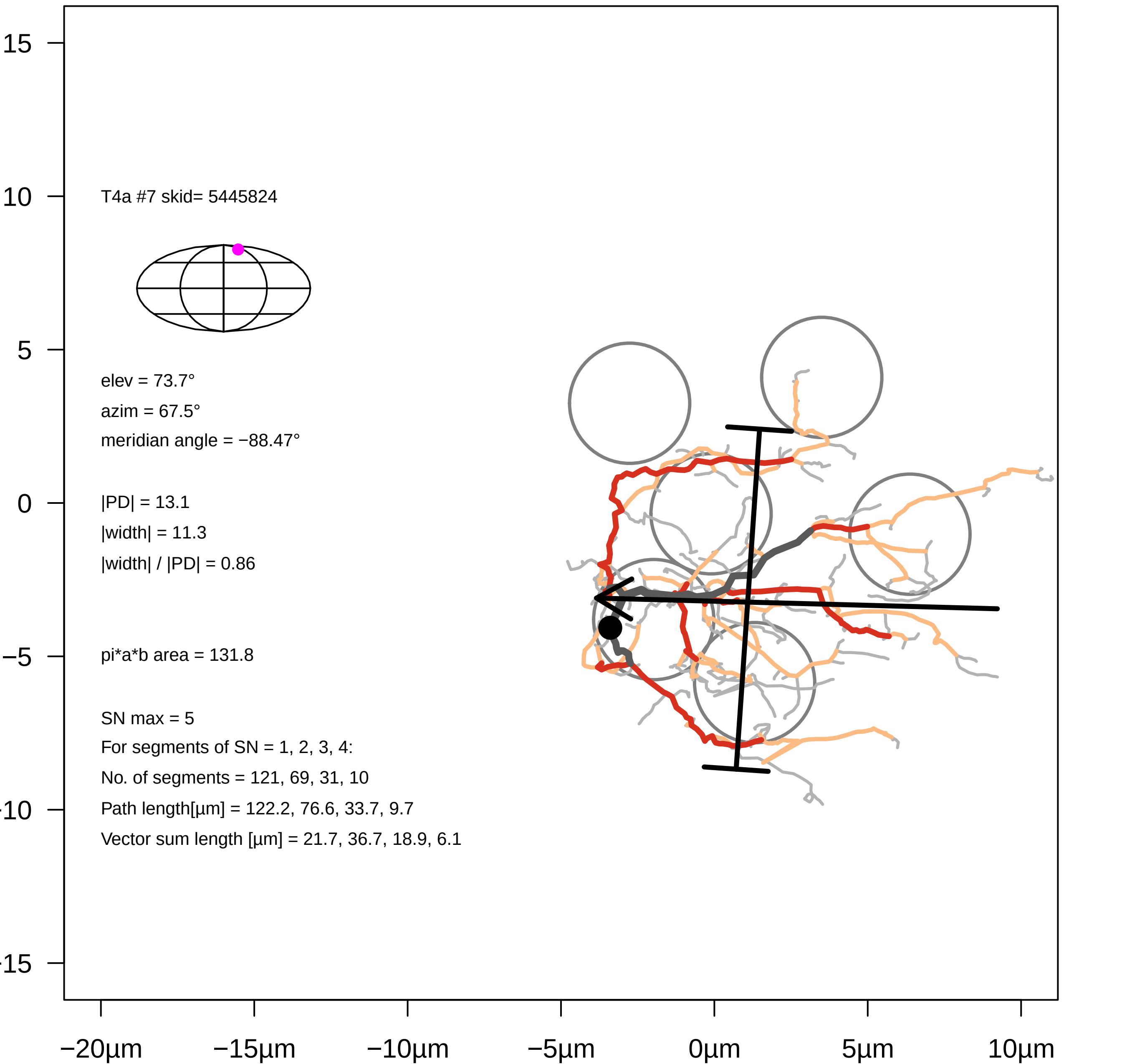
T4a #7 skid= 5445824
elev = 73.7°
azim = 67.5°
meridian angle = −88.47°
|PD| = 13.1
|width| = 11.3
|width| / |PD| = 0.86
pi*a*b area = 131.8
SN max = 5
For segments of SN = 1, 2, 3, 4:
No. of segments = 121, 69, 31, 10
Path length[µm] = 122.2, 76.6, 33.7, 9.7
Vector sum length [µm] = 21.7, 36.7, 18.9, 6.1
15
10
5
0
−5
−10
−15
−20µm
−15µm
−10µm
−5µm
0µm
5µm
10µm

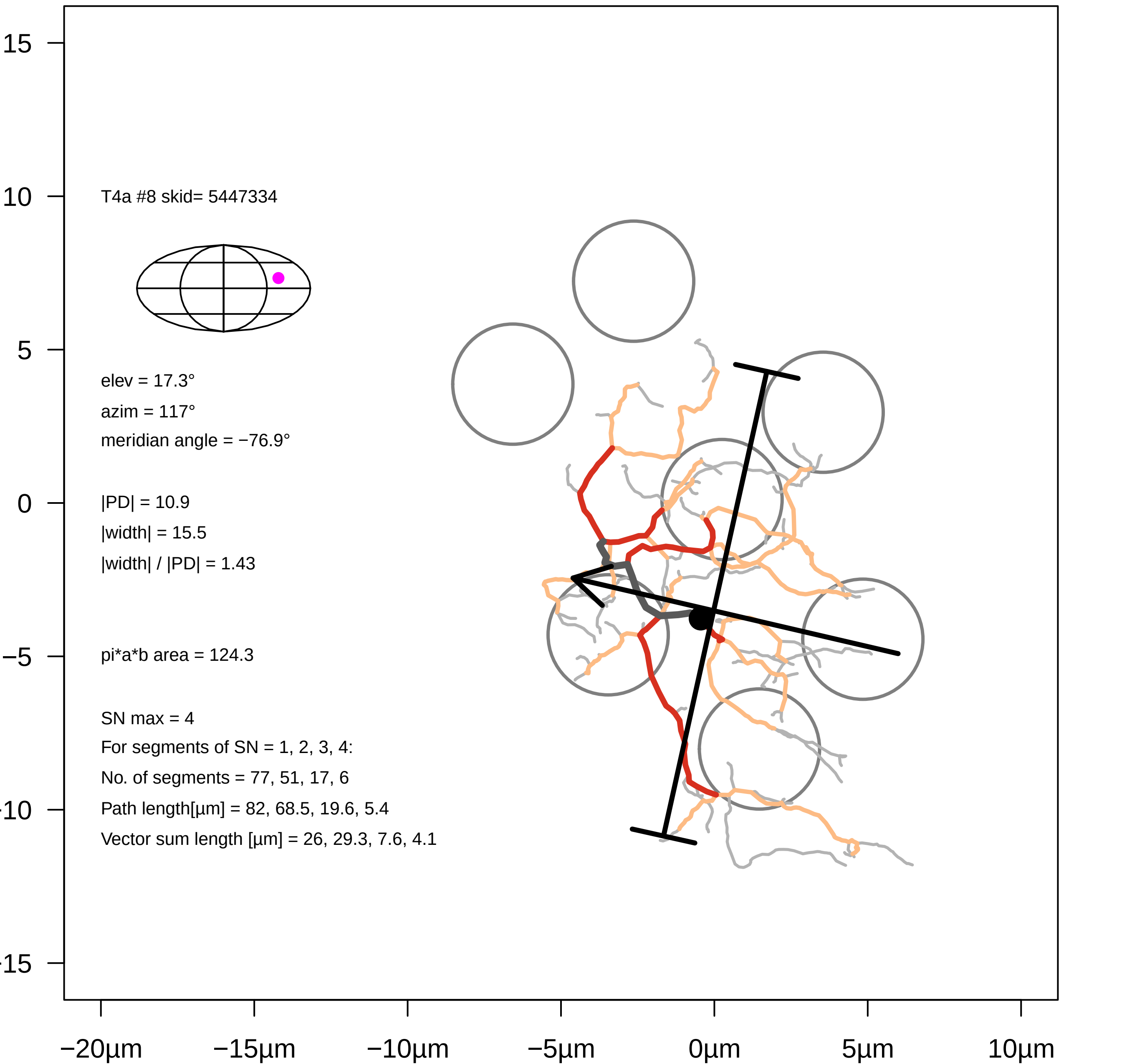

T4a #8 skid= 5447334
elev = 17.3°
azim = 117°
meridian angle = −76.9°
|PD| = 10.9
|width| = 15.5
|width| / |PD| = 1.43
pi*a*b area = 124.3
SN max = 4
For segments of SN = 1, 2, 3, 4:
No. of segments = 77, 51, 17, 6
Path length[µm] = 82, 68.5, 19.6, 5.4
Vector sum length [µm] = 26, 29.3, 7.6, 4.1
15
10
5
0
−5
−10
−15
−20µm
−15µm
−10µm
−5µm
0µm
5µm
10µm

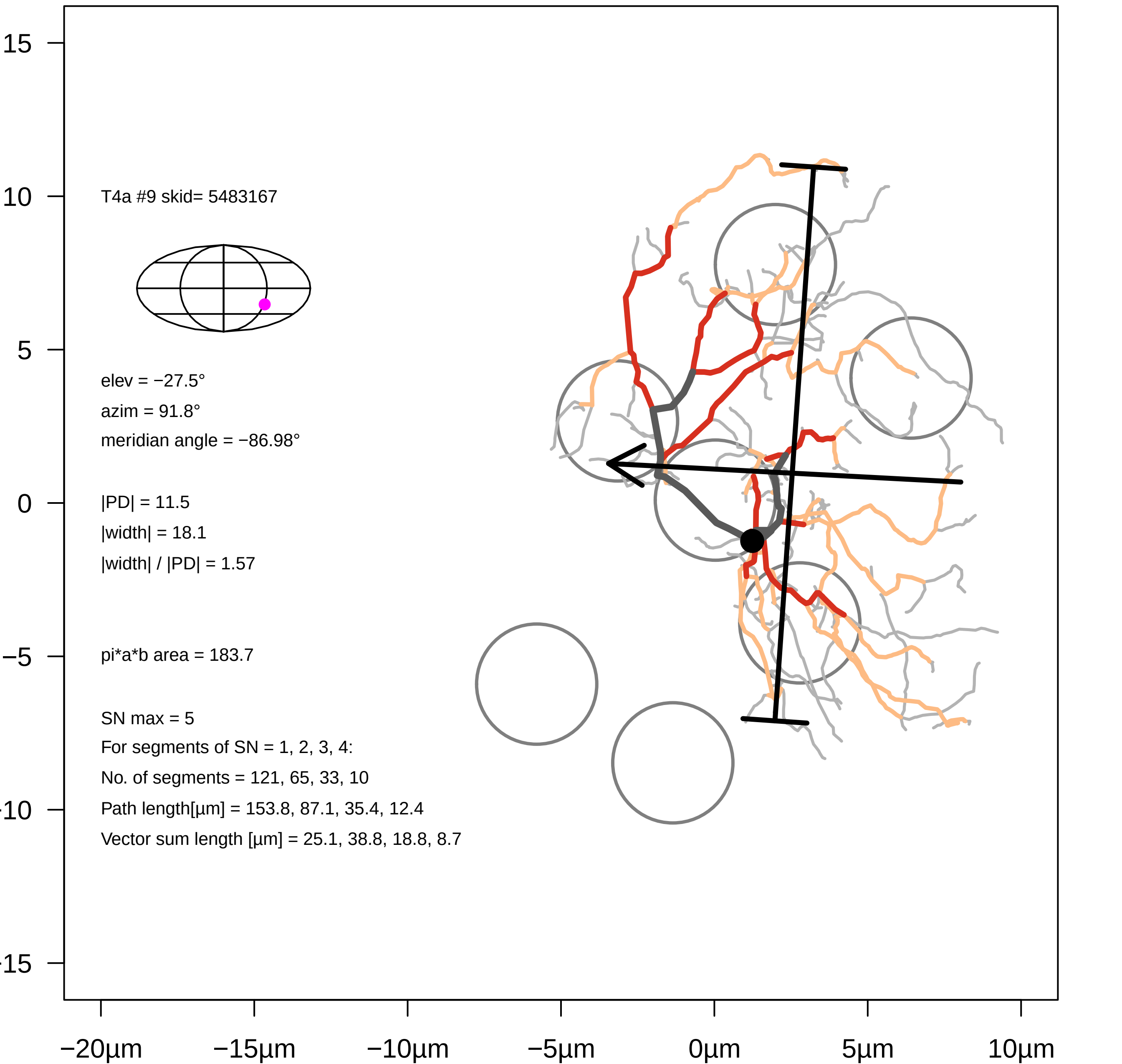
T4a #9 skid= 5483167
elev = −27.5°
azim = 91.8°
meridian angle = −86.98°
|PD| = 11.5
|width| = 18.1
|width| / |PD| = 1.57
pi*a*b area = 183.7
SN max = 5
For segments of SN = 1, 2, 3, 4:
No. of segments = 121, 65, 33, 10
Path length[µm] = 153.8, 87.1, 35.4, 12.4
Vector sum length [µm] = 25.1, 38.8, 18.8, 8.7
15
10
5
0
−5
−20µm
−15µm
−10µm
−5µm
0µm
5µm
10µm

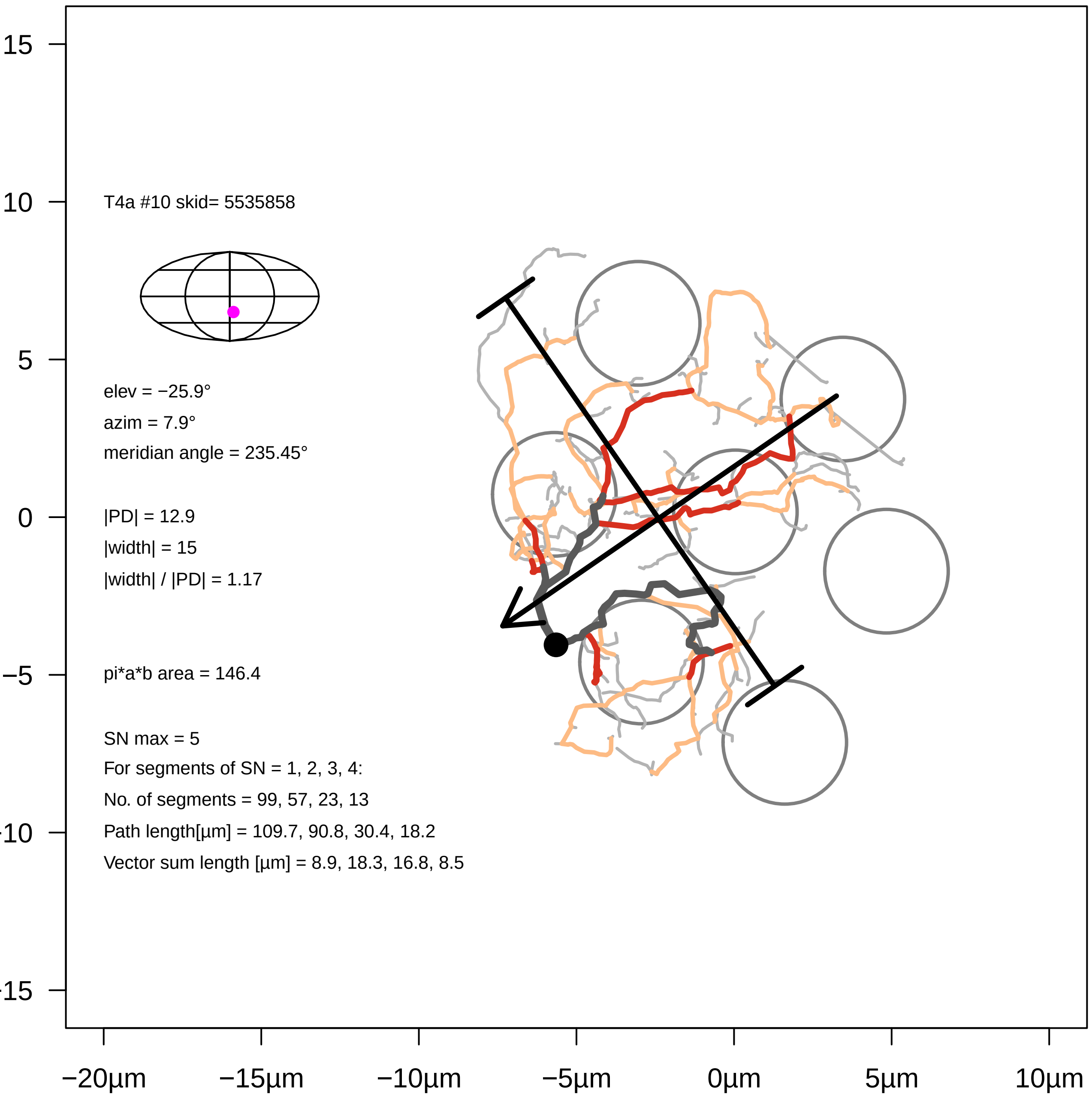

T4a #10 skid= 5535858
elev = −25.9°
azim = 7.9°
meridian angle = 235.45°
|PD| = 12.9
|width| = 15
|width| / |PD| = 1.17
pi*a*b area = 146.4
SN max = 5
For segments of SN = 1, 2, 3, 4:
No. of segments = 99, 57, 23, 13
Path length[µm] = 109.7, 90.8, 30.4, 18.2
Vector sum length [µm] = 8.9, 18.3, 16.8, 8.5
15
10
5
0
−5
−10
−15
−20µm
−15µm
−10µm
−5µm
0µm
5µm
10µm

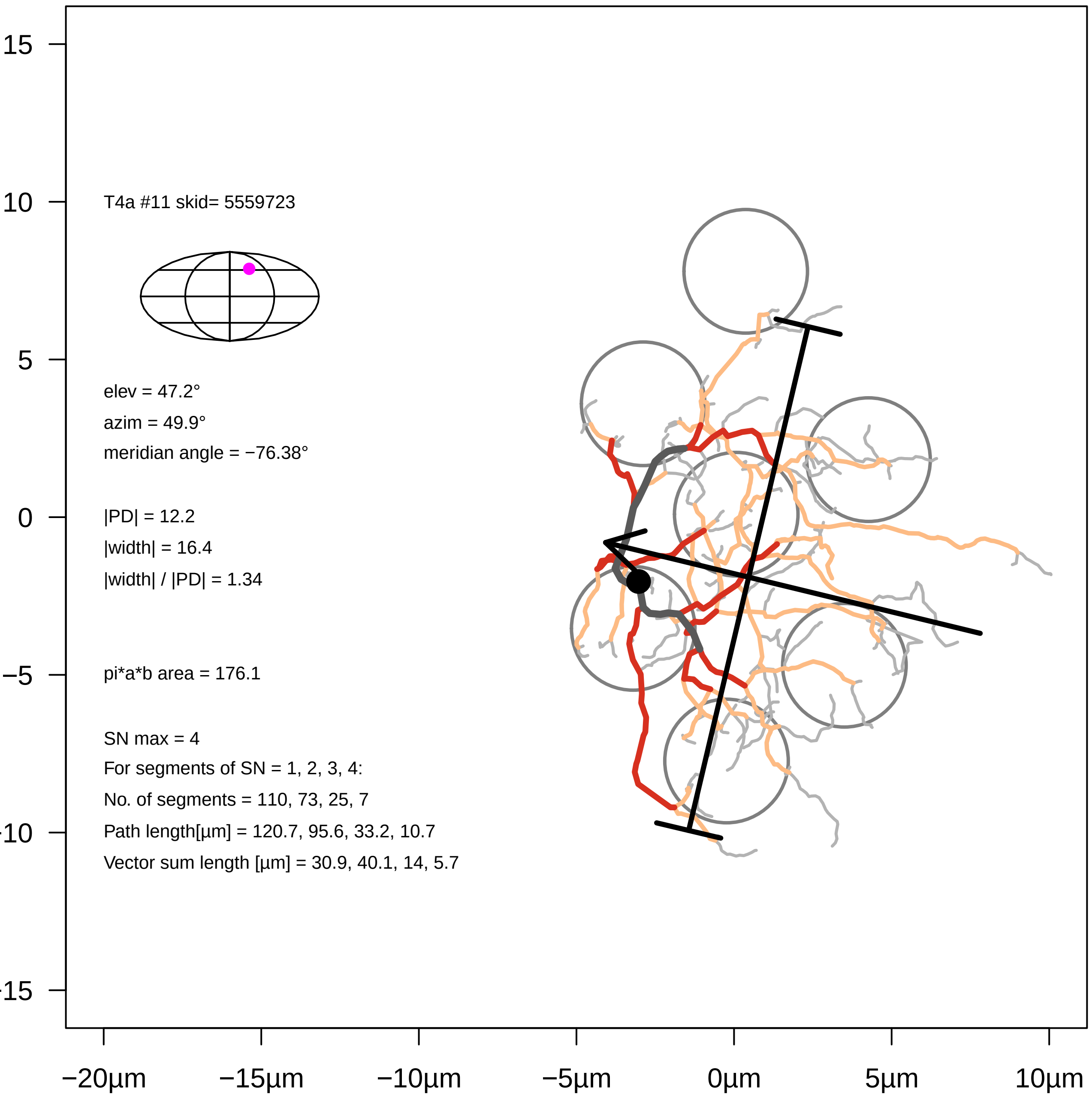

T4a #11 skid= 5559723
elev = 47.2°
azim = 49.9°
meridian angle = −76.38°
|PD| = 12.2
|width| = 16.4
|width| / |PD| = 1.34
pi*a*b area = 176.1
SN max = 4
For segments of SN = 1, 2, 3, 4:
No. of segments = 110, 73, 25, 7
Path length[µm] = 120.7, 95.6, 33.2, 10.7
Vector sum length [µm] = 30.9, 40.1, 14, 5.7
15
10
5
0
−5
−10
−15
−20µm
−15µm
−10µm
−5µm
0µm
5µm
10µm

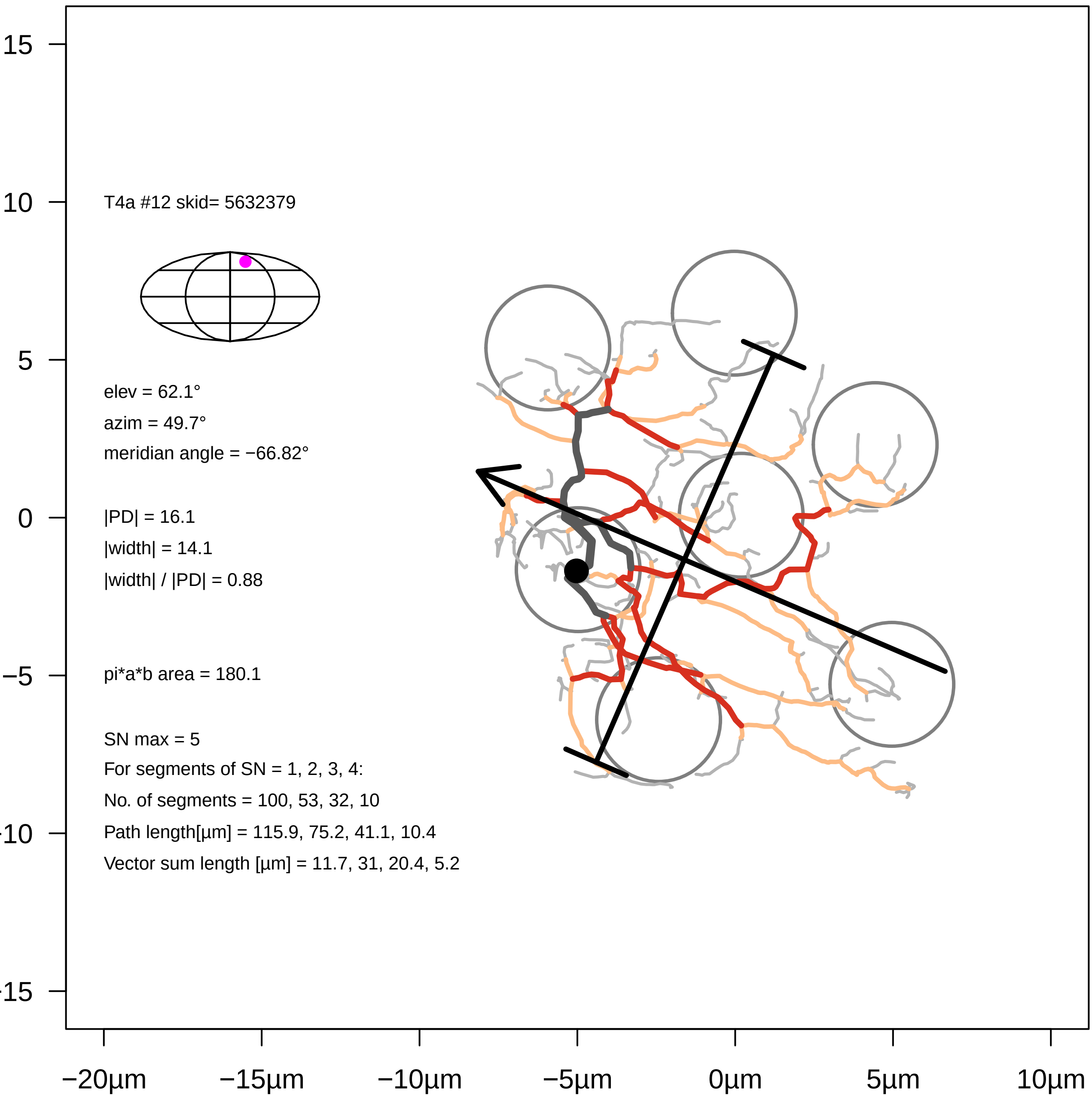

T4a #12 skid= 5632379
elev = 62.1°
azim = 49.7°
meridian angle = −66.82°
|PD| = 16.1
|width| = 14.1
|width| / |PD| = 0.88
pi*a*b area = 180.1
SN max = 5
For segments of SN = 1, 2, 3, 4:
No. of segments = 100, 53, 32, 10
Path length[µm] = 115.9, 75.2, 41.1, 10.4
Vector sum length [µm] = 11.7, 31, 20.4, 5.2
15
10
5
0
−5
−10
−15
−20µm
−15µm
−10µm
−5µm
0µm
5µm
10µm

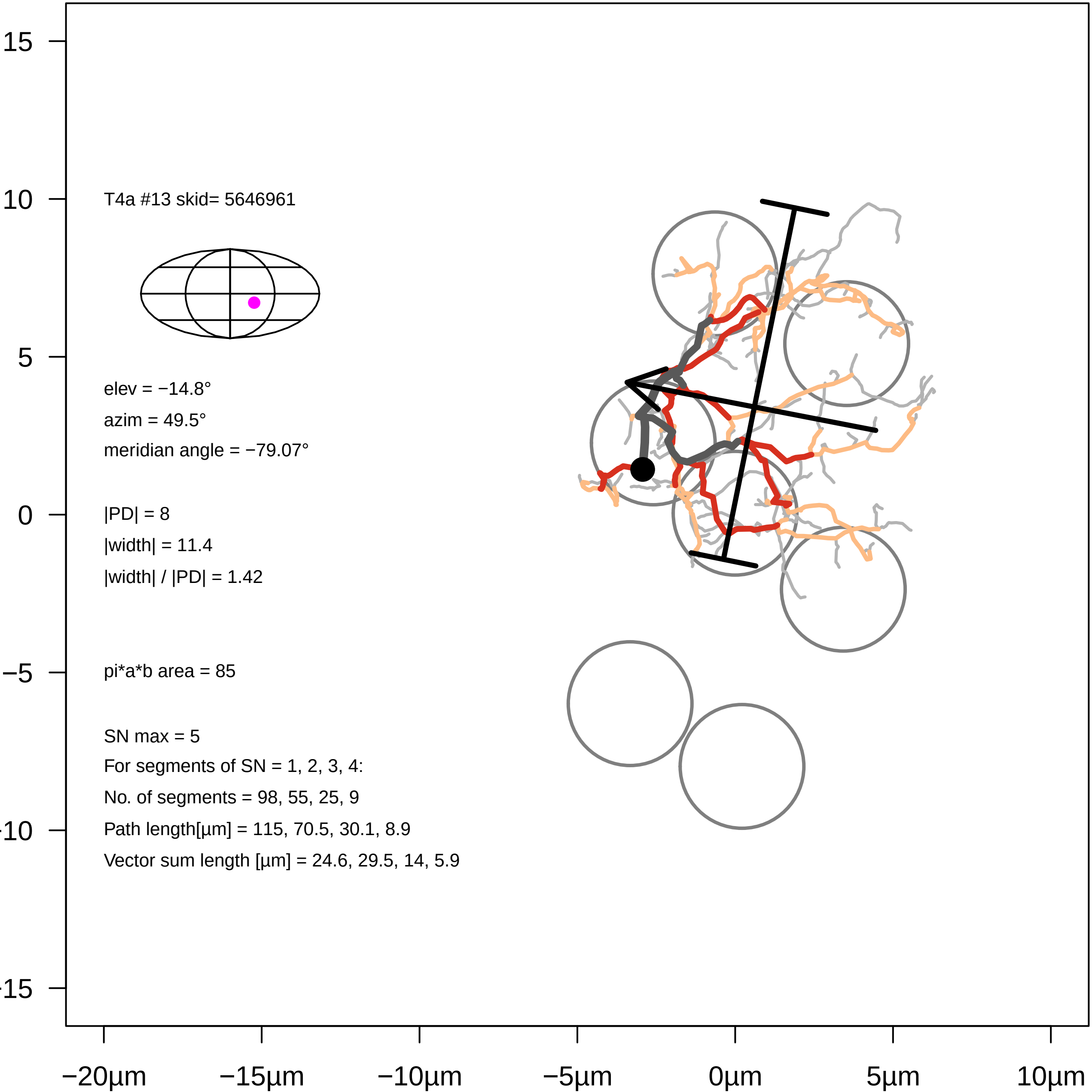

15
10
5
0
−5
−10
−15
−20µm
−15µm
−10µm
−5µm
0µm
5µm
10µm
T4a #13 skid= 5646961
elev = −14.8°
azim = 49.5°
meridian angle = −79.07°
|PD| = 8
|width| = 11.4
|width| / |PD| = 1.42
pi*a*b area = 85
SN max = 5
For segments of SN = 1, 2, 3, 4:
No. of segments = 98, 55, 25, 9
Path length[µm] = 115, 70.5, 30.1, 8.9
Vector sum length [µm] = 24.6, 29.5, 14, 5.9

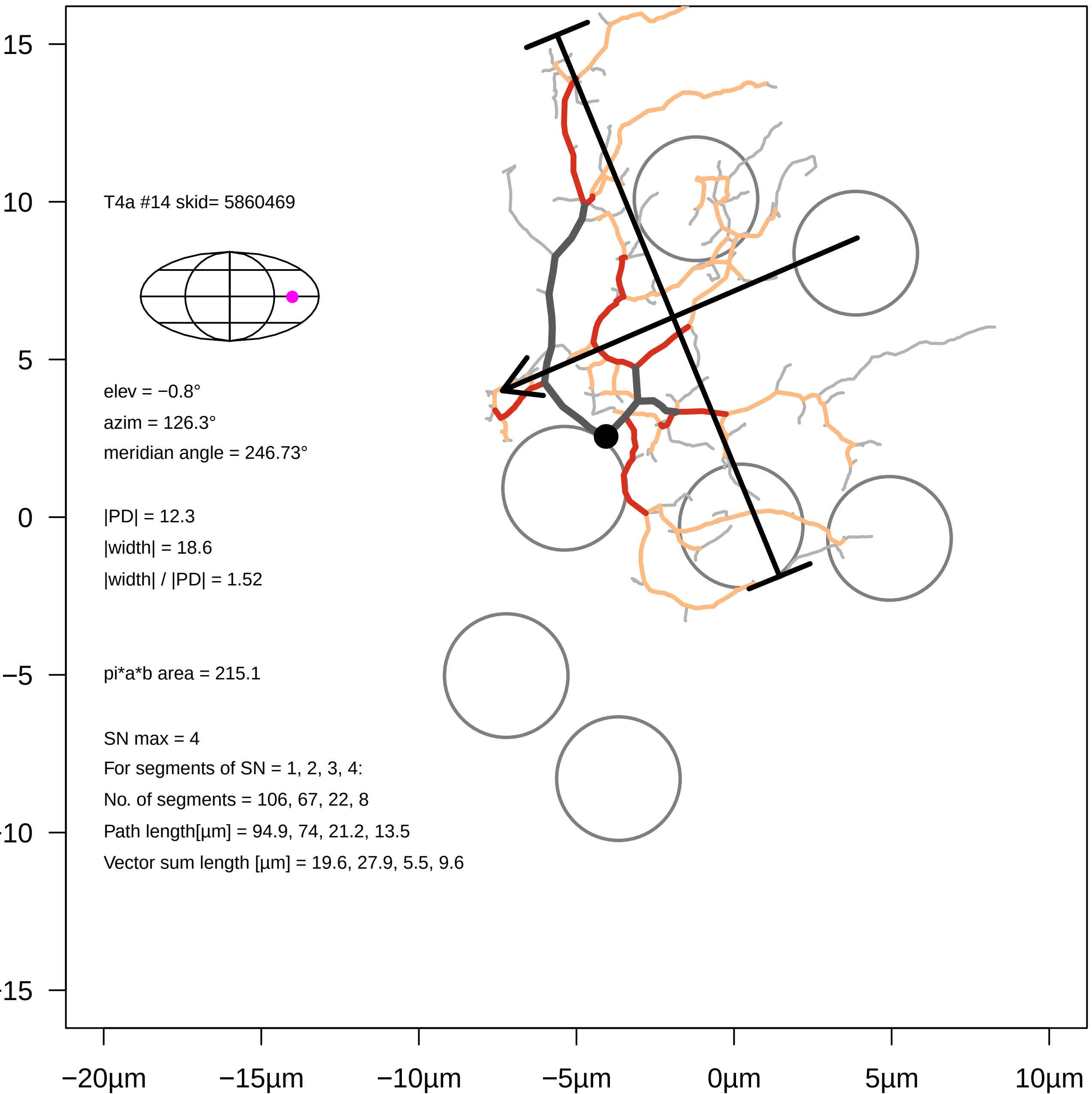

T4a #14 skid= 5860469
elev = −0.8°
azim = 126.3°
meridian angle = 246.73°
|PD| = 12.3
|width| = 18.6
|width| / |PD| = 1.52
pi*a*b area = 215.1
SN max = 4
For segments of SN = 1, 2, 3, 4:
No. of segments = 106, 67, 22, 8
Path length[µm] = 94.9, 74, 21.2, 13.5
Vector sum length [µm] = 19.6, 27.9, 5.5, 9.6
15
10
5
0
−5
-10
-15
−20µm
−15µm
−10µm
−5µm
0µm
5µm
10µm

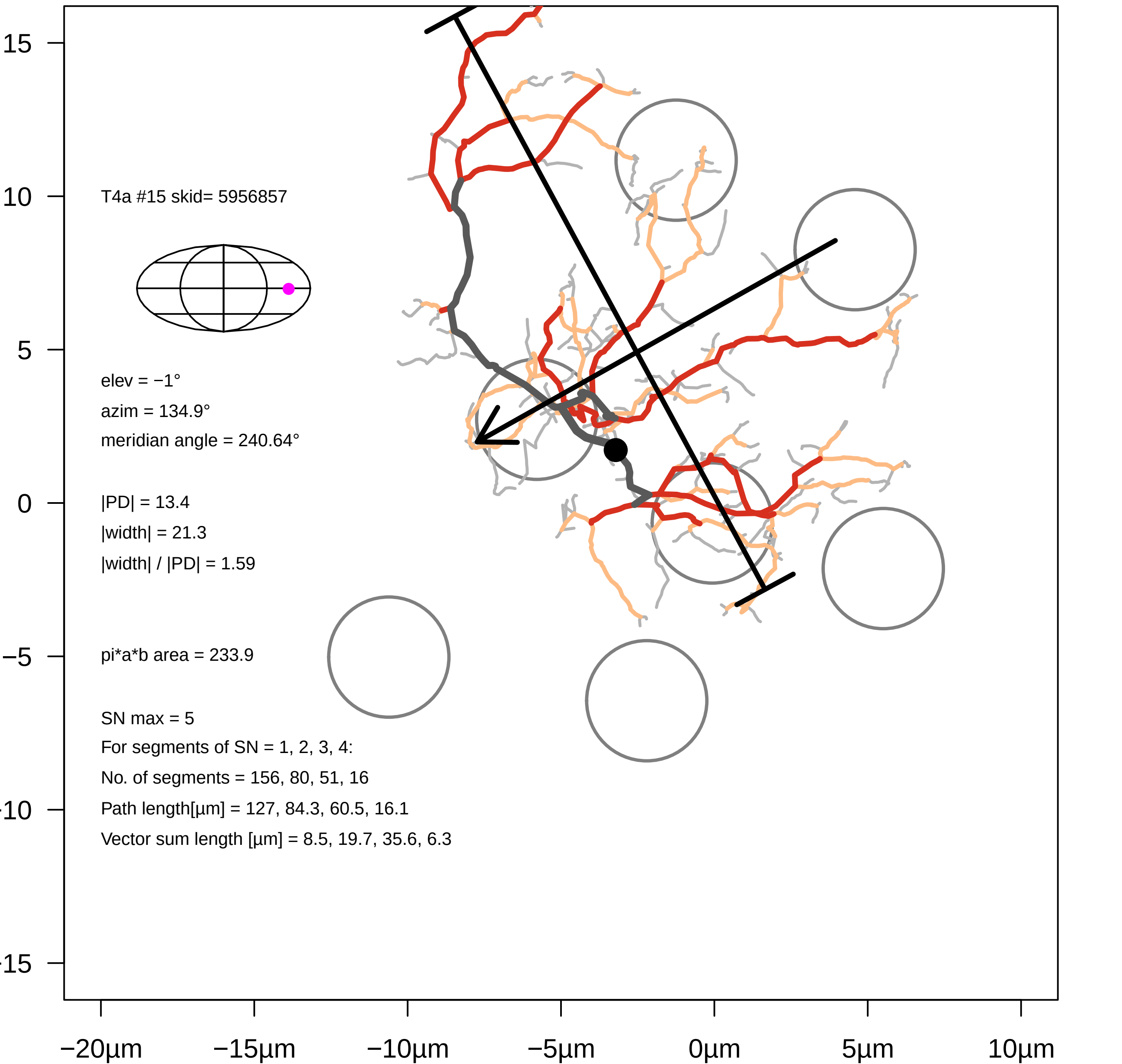
T4a #15 skid= 5956857
elev = −1°
azim = 134.9°
meridian angle = 240.64°
|PD| = 13.4
|width| = 21.3
|width| / |PD| = 1.59
pi*a*b area = 233.9
SN max = 5
For segments of SN = 1, 2, 3, 4:
No. of segments = 156, 80, 51, 16
Path length[µm] = 127, 84.3, 60.5, 16.1
Vector sum length [µm] = 8.5, 19.7, 35.6, 6.3
15
10
5
0
−5
-10
-15
−20µm
−15µm
−10µm
−5µm
0µm
5µm
10µm

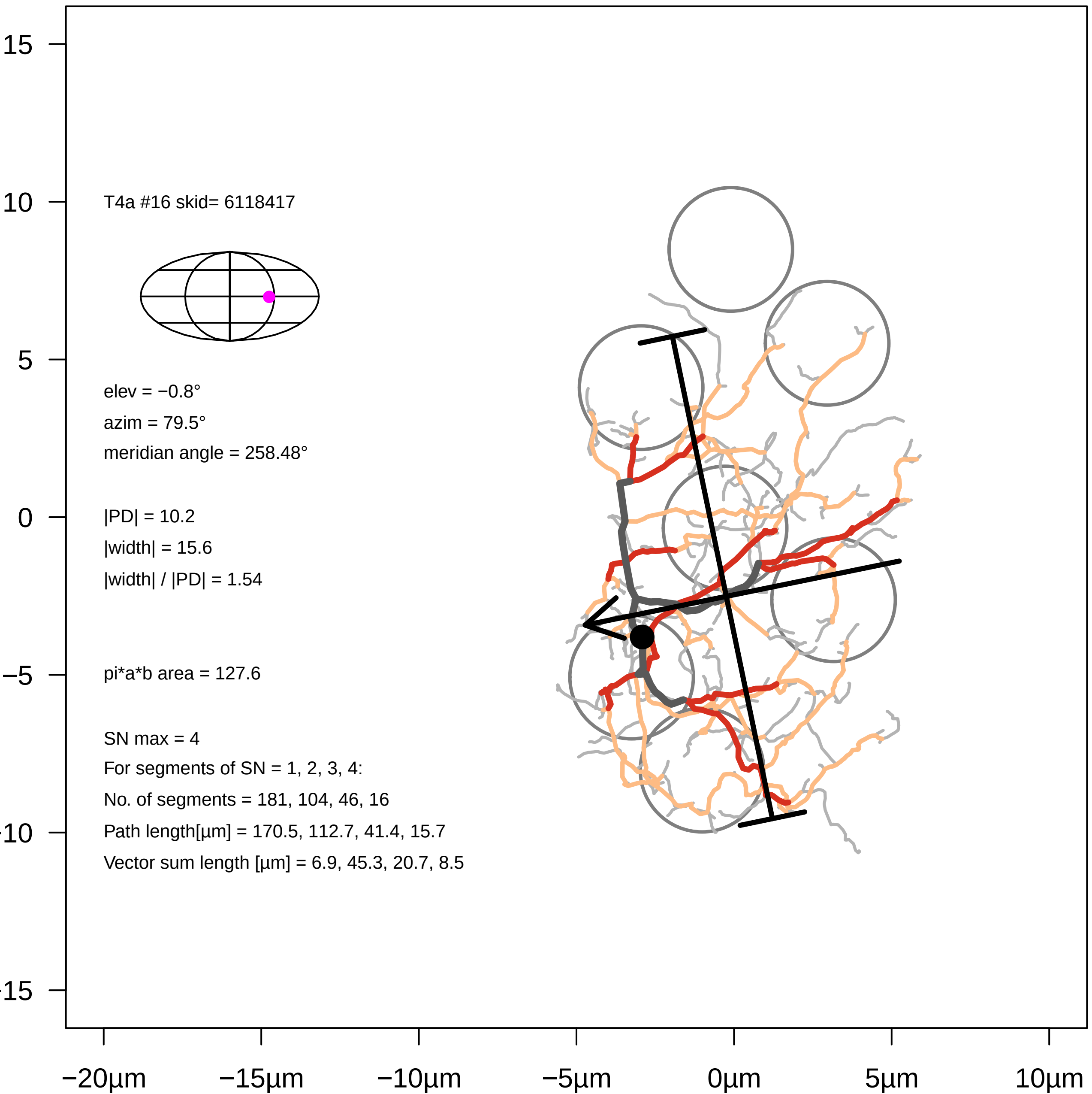

T4a #16 skid= 6118417
elev = −0.8°
azim = 79.5°
meridian angle = 258.48°
|PD| = 10.2
|width| = 15.6
|width| / |PD| = 1.54
pi*a*b area = 127.6
SN max = 4
For segments of SN = 1, 2, 3, 4:
No. of segments = 181, 104, 46, 16
Path length[µm] = 170.5, 112.7, 41.4, 15.7
Vector sum length [µm] = 6.9, 45.3, 20.7, 8.5
15
10
5
0
−5
−10
−15
−20µm
−15µm
−10µm
−5µm
0µm
5µm
10µm

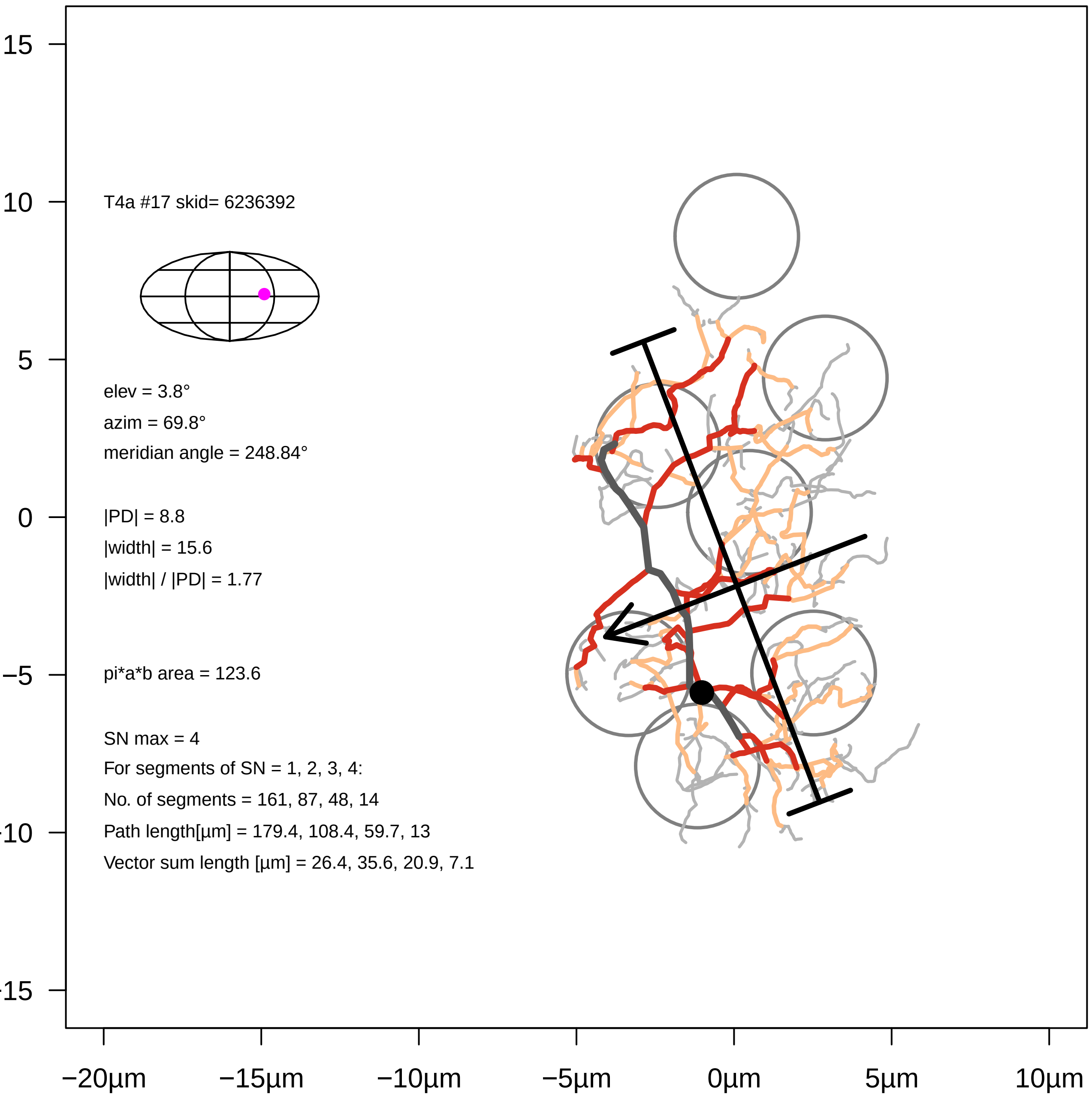

T4a #17 skid= 6236392
elev = 3.8°
azim = 69.8°
meridian angle = 248.84°
|PD| = 8.8
|width| = 15.6
|width| / |PD| = 1.77
pi*a*b area = 123.6
SN max = 4
For segments of SN = 1, 2, 3, 4:
No. of segments = 161, 87, 48, 14
Path length[µm] = 179.4, 108.4, 59.7, 13
Vector sum length [µm] = 26.4, 35.6, 20.9, 7.1
15
10
5
0
−5
−10
−15
−20µm
−15µm
−10µm
−5µm
0µm
5µm
10µm

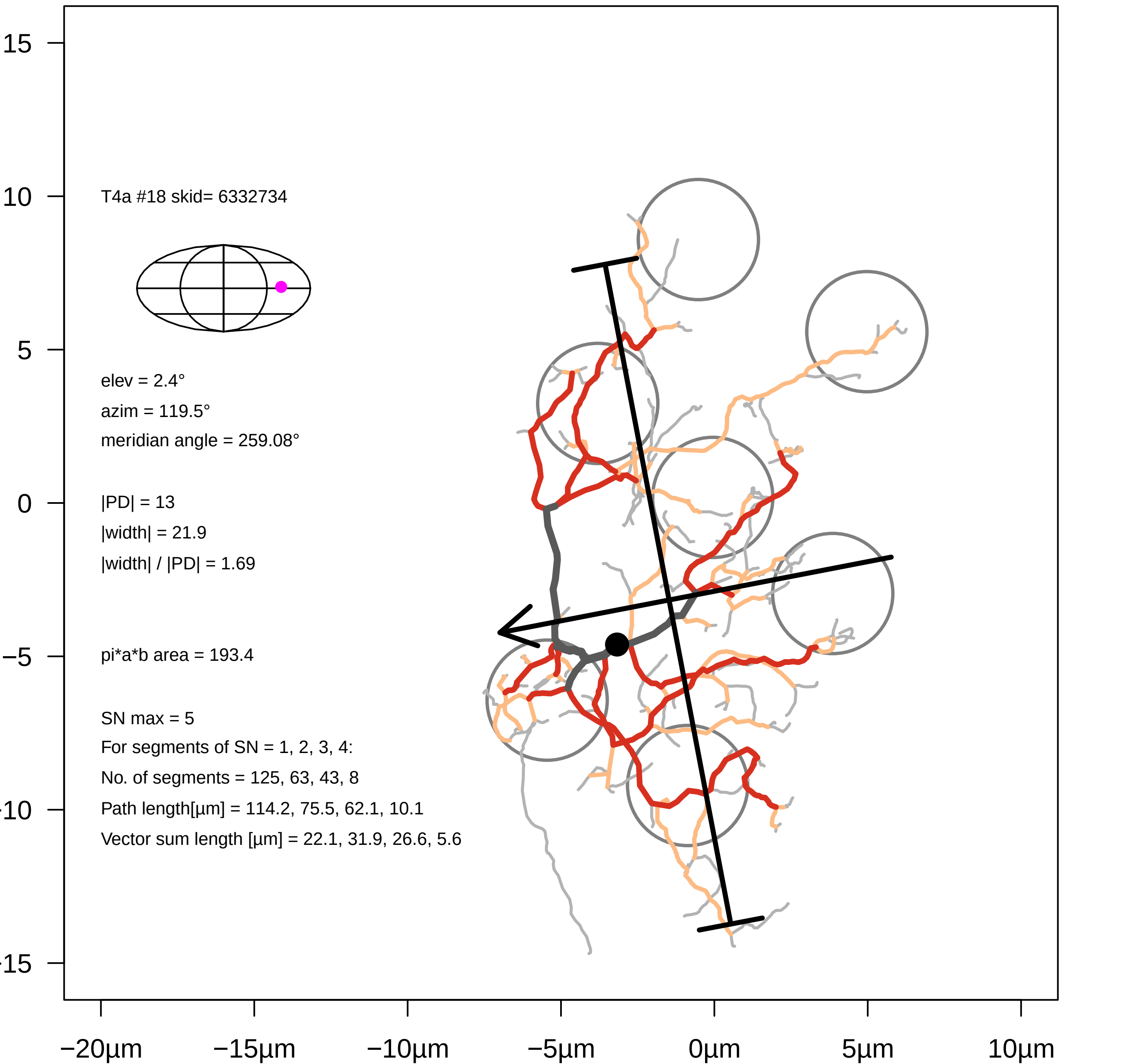
T4a #18 skid= 6332734
elev = 2.4°
azim = 119.5°
meridian angle = 259.08°
|PD| = 13
|width| = 21.9
|width| / |PD| = 1.69
pi*a*b area = 193.4
SN max = 5
For segments of SN = 1, 2, 3, 4:
No. of segments = 125, 63, 43, 8
Path length[µm] = 114.2, 75.5, 62.1, 10.1
Vector sum length [µm] = 22.1, 31.9, 26.6, 5.6
15
10
5
0
−5
−10
−15
−20µm
−15µm
−10µm
−5µm
0µm
5µm
10µm

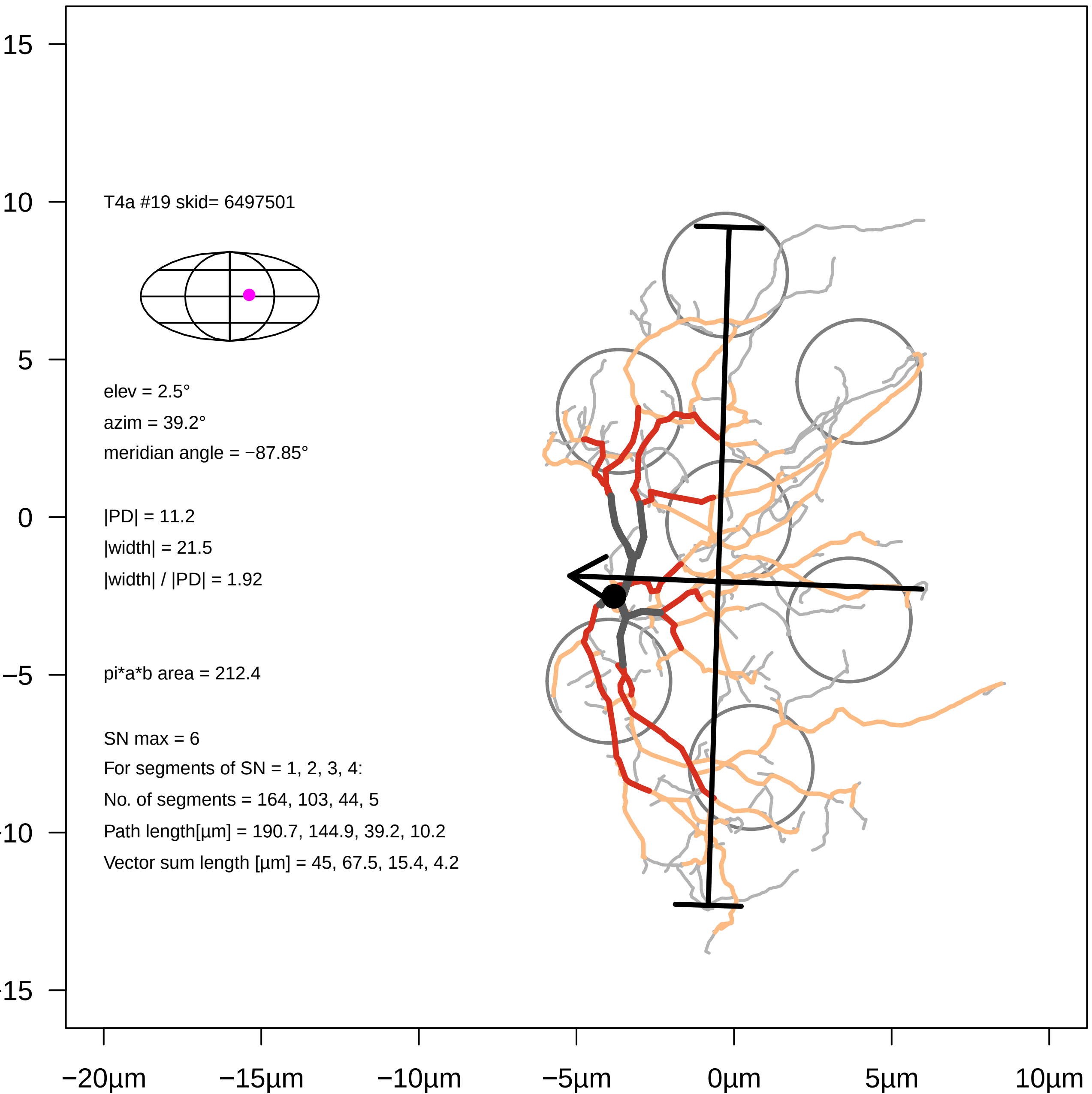
T4a #19 skid= 6497501
elev = 2.5°
azim = 39.2°
meridian angle = −87.85°
|PD| = 11.2
|width| = 21.5
|width| / |PD| = 1.92
pi*a*b area = 212.4
SN max = 6
For segments of SN = 1, 2, 3, 4:
No. of segments = 164, 103, 44, 5
Path length[µm] = 190.7, 144.9, 39.2, 10.2
Vector sum length [µm] = 45, 67.5, 15.4, 4.2
15
10
5
0
−5
-10
-15
−20µm
−15µm
−10µm
−5µm
0µm
5µm
10µm

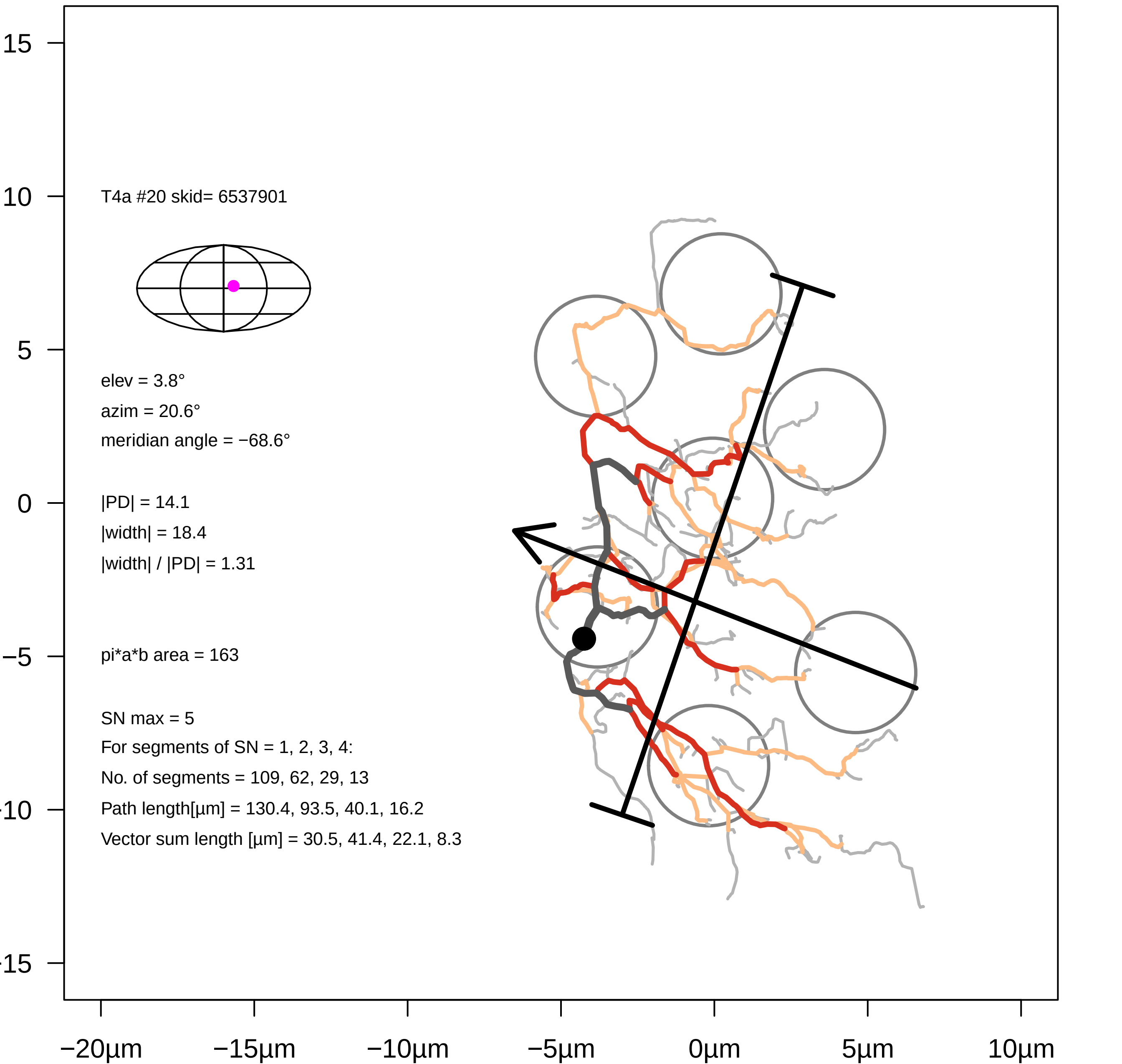
T4a #20 skid= 6537901
elev = 3.8°
azim = 20.6°
meridian angle = −68.6°
|PD| = 14.1
|width| = 18.4
|width| / |PD| = 1.31
pi*a*b area = 163
SN max = 5
For segments of SN = 1, 2, 3, 4:
No. of segments = 109, 62, 29, 13
Path length[µm] = 130.4, 93.5, 40.1, 16.2
Vector sum length [µm] = 30.5, 41.4, 22.1, 8.3
15
10
5
0
−5
−10
−15
−20µm
−15µm
−10µm
−5µm
0µm
5µm
10µm

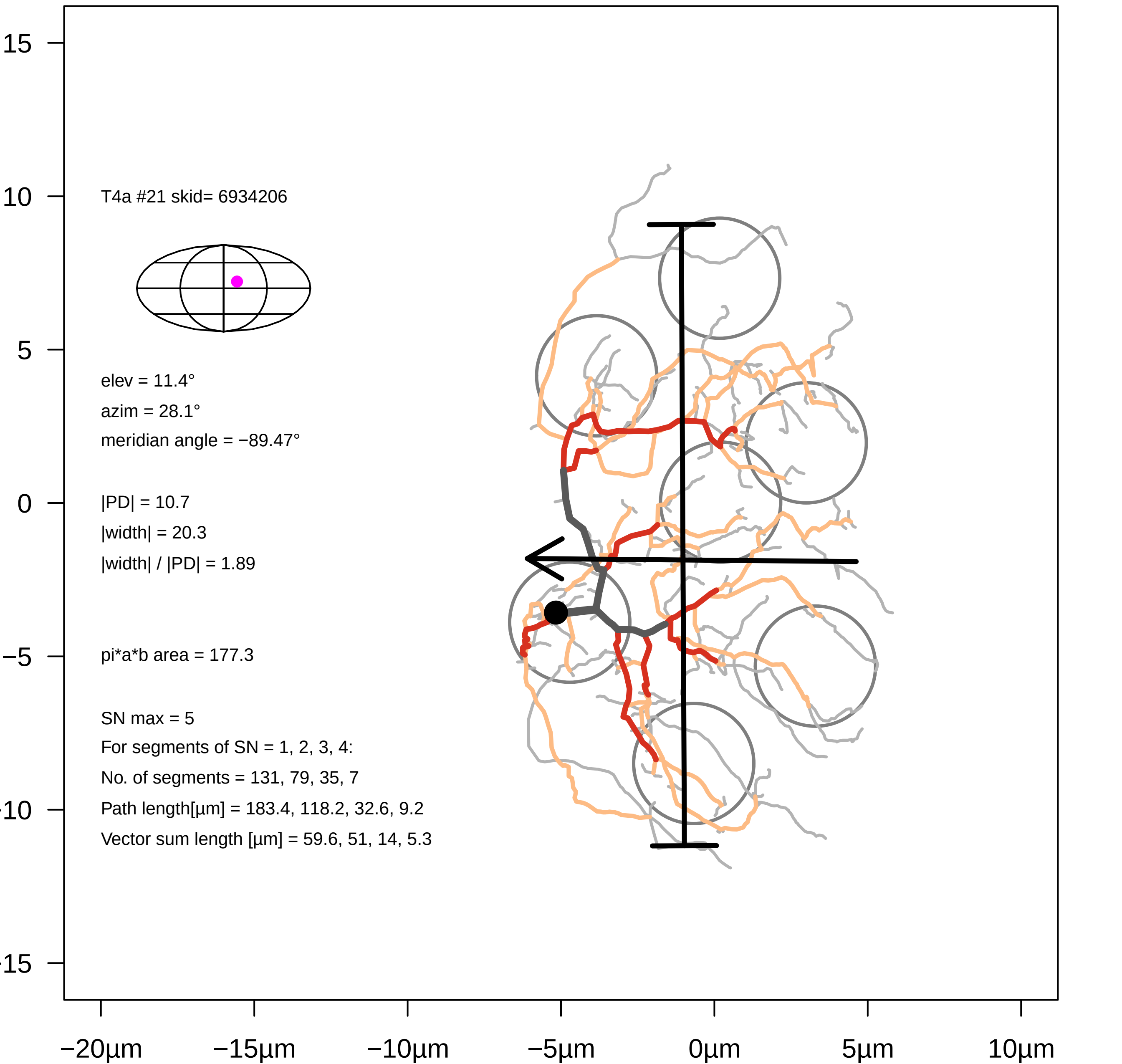

T4a #21 skid= 6934206
elev = 11.4°
azim = 28.1°
meridian angle = −89.47°
|PD| = 10.7
|width| = 20.3
|width| / |PD| = 1.89
pi*a*b area = 177.3
SN max = 5
For segments of SN = 1, 2, 3, 4:
No. of segments = 131, 79, 35, 7
Path length[µm] = 183.4, 118.2, 32.6, 9.2
Vector sum length [µm] = 59.6, 51, 14, 5.3
15
10
5
0
−5
−10
−15
−20µm
−15µm
−10µm
−5µm
0µm
5µm
10µm

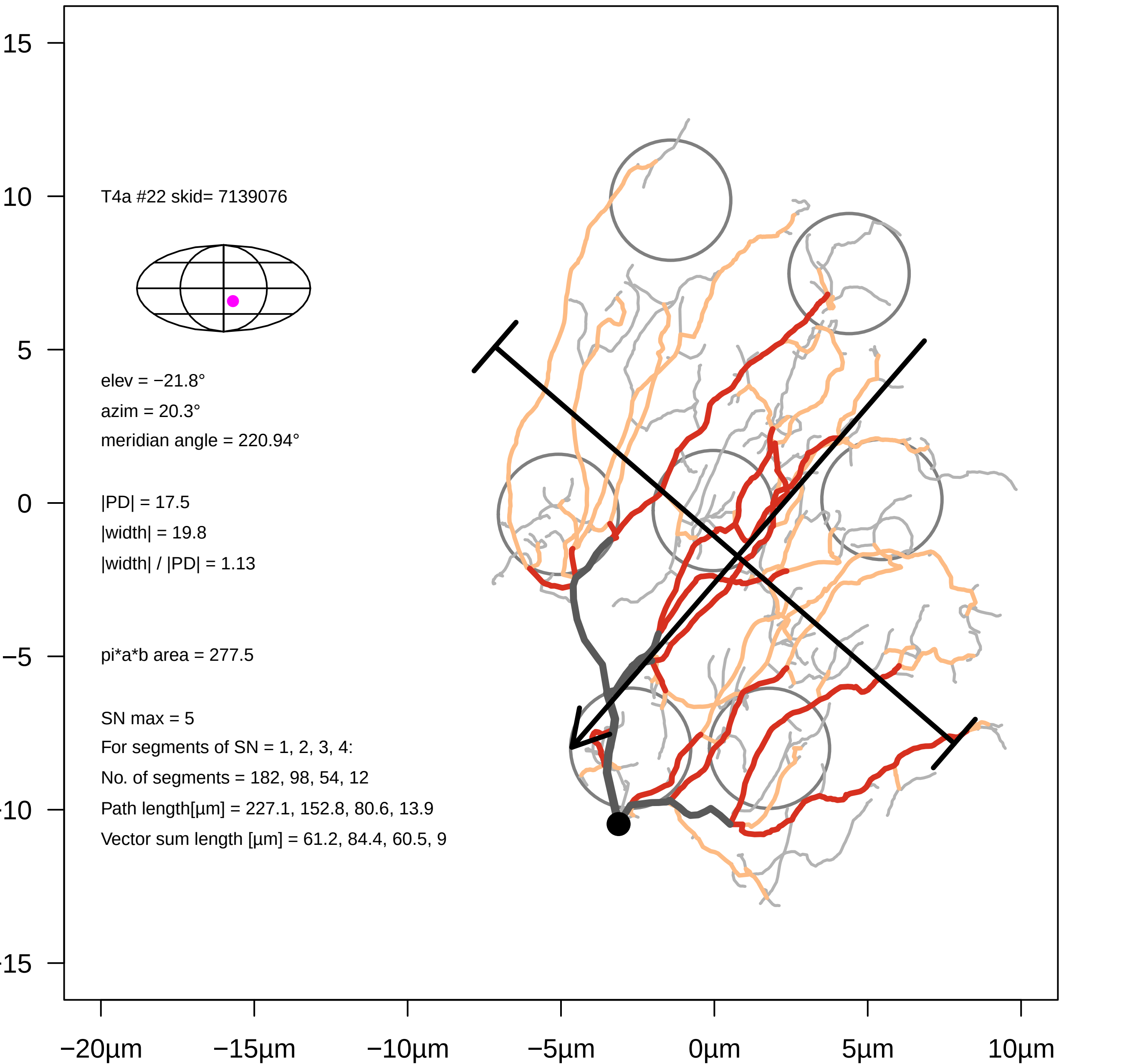

15
10
5
0
−5
−10
−15
−20µm
−15µm
−10µm
−5µm
0µm
5µm
10µm
T4a #22 skid= 7139076
elev = −21.8°
azim = 20.3°
meridian angle = 220.94°
|PD| = 17.5
|width| = 19.8
|width| / |PD| = 1.13
pi*a*b area = 277.5
SN max = 5
For segments of SN = 1, 2, 3, 4:
No. of segments = 182, 98, 54, 12
Path length[µm] = 227.1, 152.8, 80.6, 13.9
Vector sum length [µm] = 61.2, 84.4, 60.5, 9

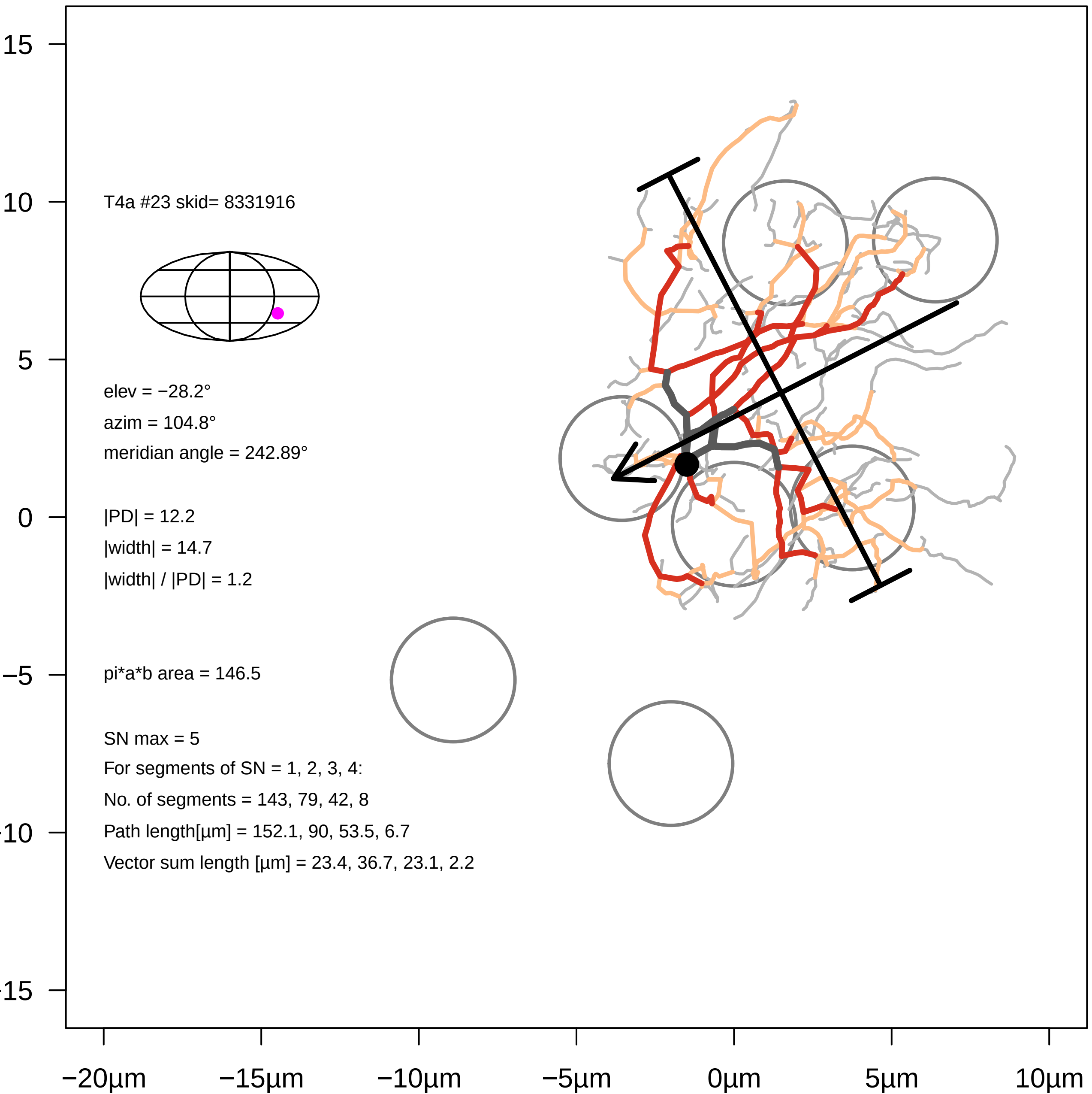

T4a #23 skid= 8331916
elev = −28.2°
azim = 104.8°
meridian angle = 242.89°
|PD| = 12.2
|width| = 14.7
|width| / |PD| = 1.2
pi*a*b area = 146.5
SN max = 5
For segments of SN = 1, 2, 3, 4:
No. of segments = 143, 79, 42, 8
Path length[µm] = 152.1, 90, 53.5, 6.7
Vector sum length [µm] = 23.4, 36.7, 23.1, 2.2
15
10
5
0
−5
-10
-15
−20µm
−15µm
−10µm
−5µm
0µm
5µm
10µm

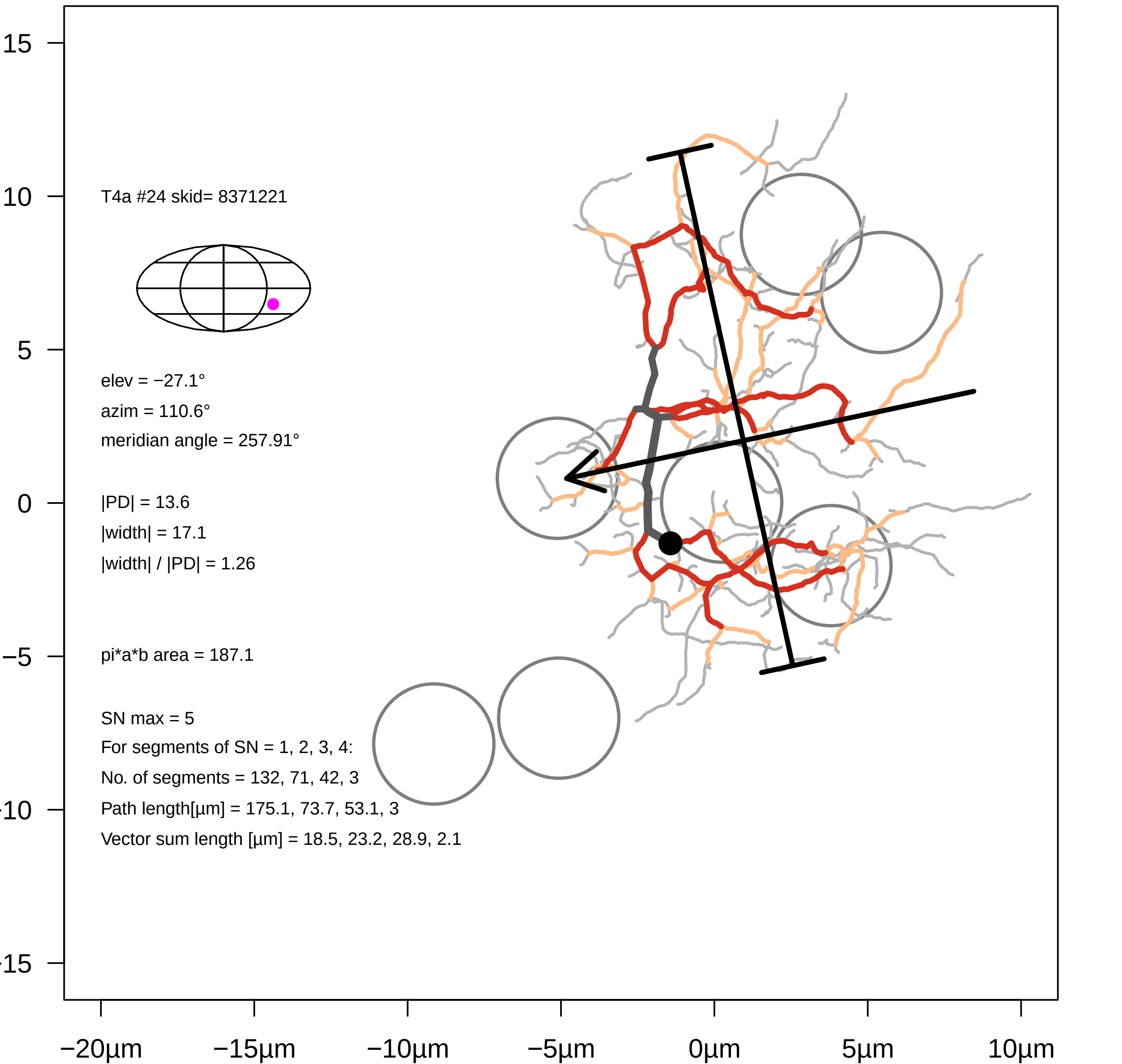

T4a #24 skid= 8371221
elev = −27.1°
azim = 110.6°
meridian angle = 257.91°
|PD| = 13.6
|width| = 17.1
|width| / |PD| = 1.26
pi*a*b area = 187.1
SN max = 5
For segments of SN = 1, 2, 3, 4:
No. of segments = 132, 71, 42, 3
Path length[µm] = 175.1, 73.7, 53.1, 3
Vector sum length [µm] = 18.5, 23.2, 28.9, 2.1
15
10
5
0
−5
−10
−15
−20µm
−15µm
−10µm
−5µm
0µm
5µm
10µm

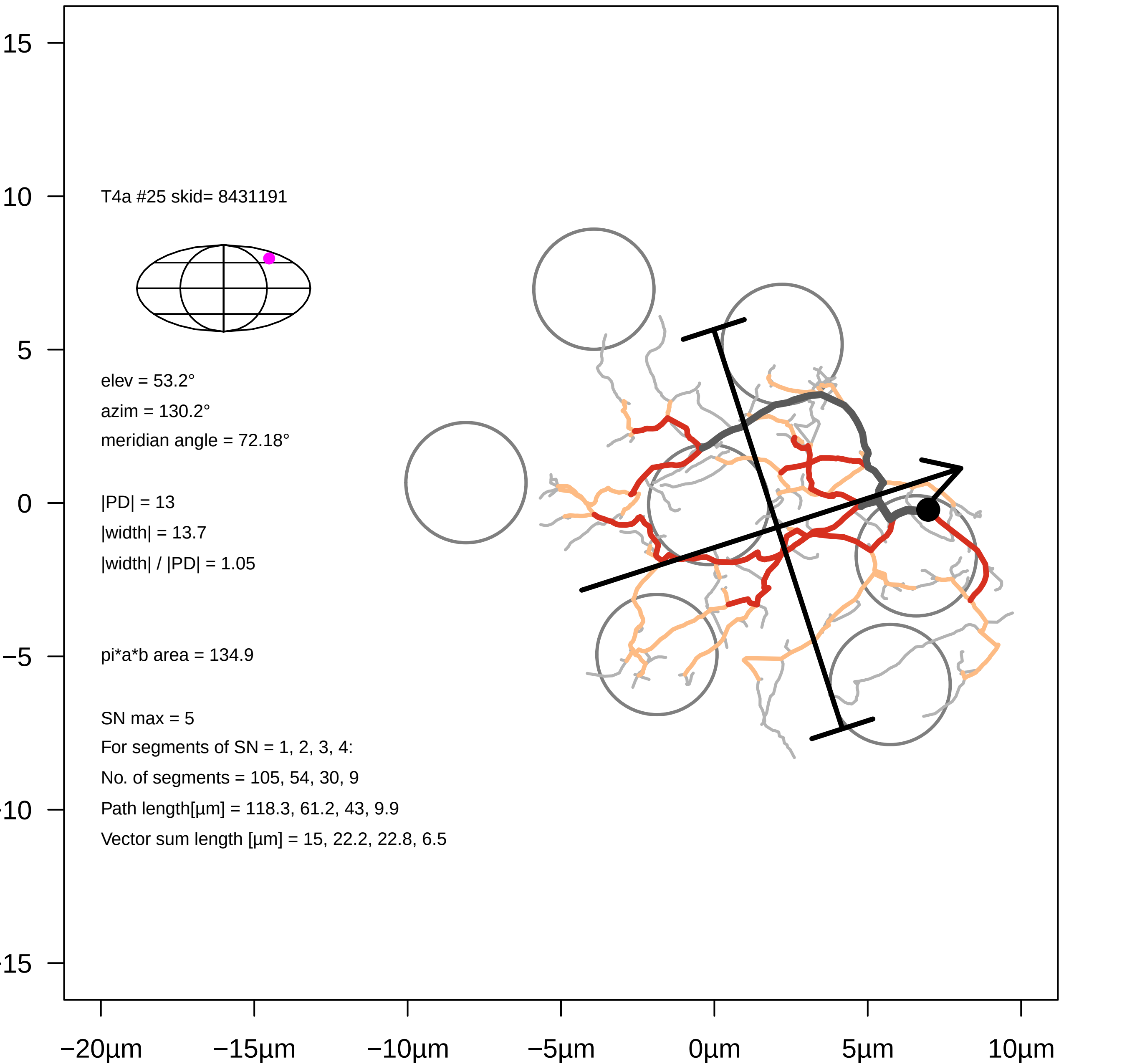
T4a #25 skid= 8431191
elev = 53.2°
azim = 130.2°
meridian angle = 72.18°
|PD| = 13
|width| = 13.7
|width| / |PD| = 1.05
pi*a*b area = 134.9
SN max = 5
For segments of SN = 1, 2, 3, 4:
No. of segments = 105, 54, 30, 9
Path length[µm] = 118.3, 61.2, 43, 9.9
Vector sum length [µm] = 15, 22.2, 22.8, 6.5
15
10
5
0
−5
-10
-15
−20µm
−15µm
−10µm
−5µm
0µm
5µm
10µm

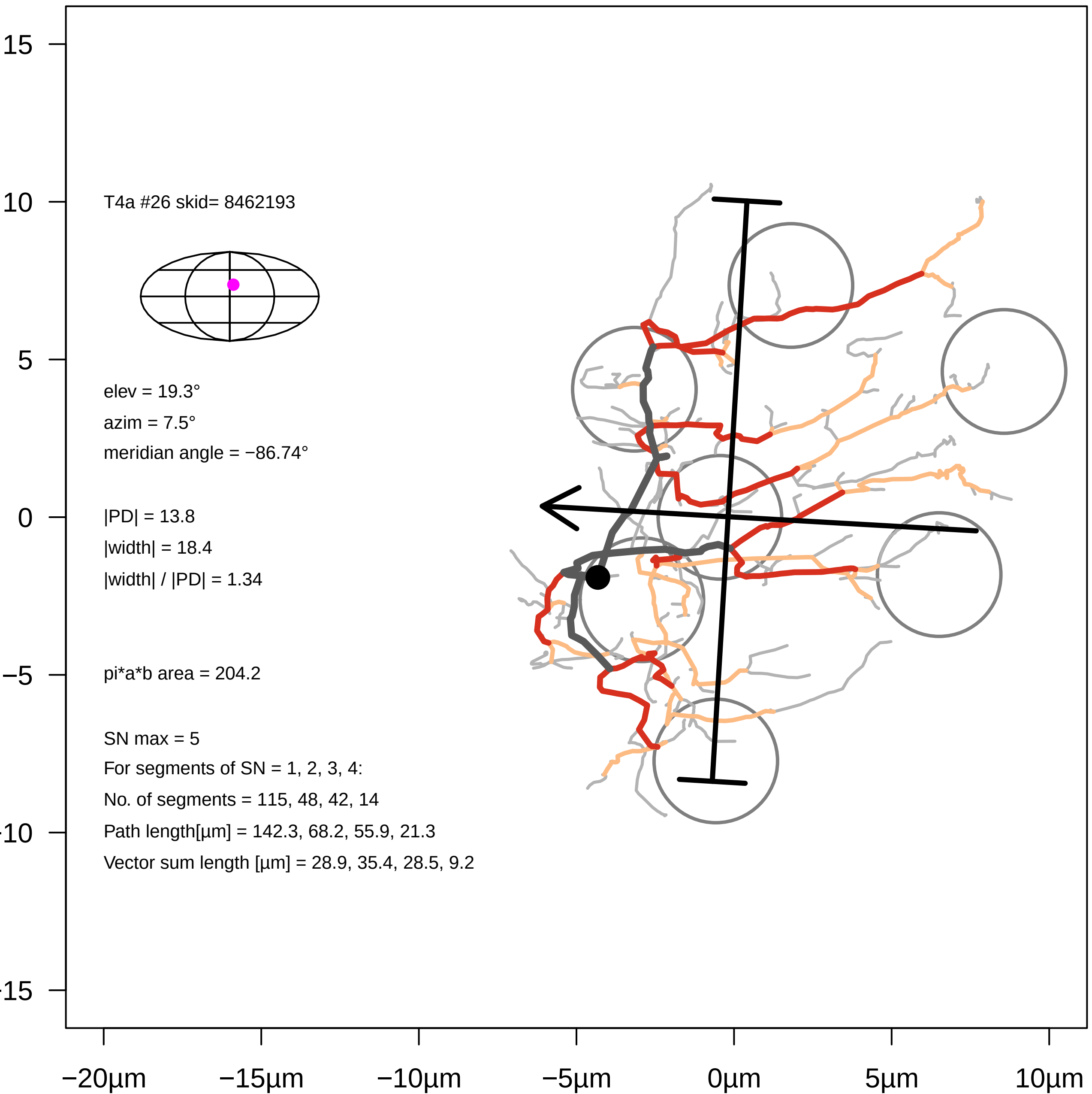
T4a #26 skid= 8462193
elev = 19.3°
azim = 7.5°
meridian angle = −86.74°
|PD| = 13.8
|width| = 18.4
|width| / |PD| = 1.34
pi*a*b area = 204.2
SN max = 5
For segments of SN = 1, 2, 3, 4:
No. of segments = 115, 48, 42, 14
Path length[µm] = 142.3, 68.2, 55.9, 21.3
Vector sum length [µm] = 28.9, 35.4, 28.5, 9.2
15
10
5
0
−5
−10
−15
−20µm
−15µm
−10µm
−5µm
0µm
5µm
10µm

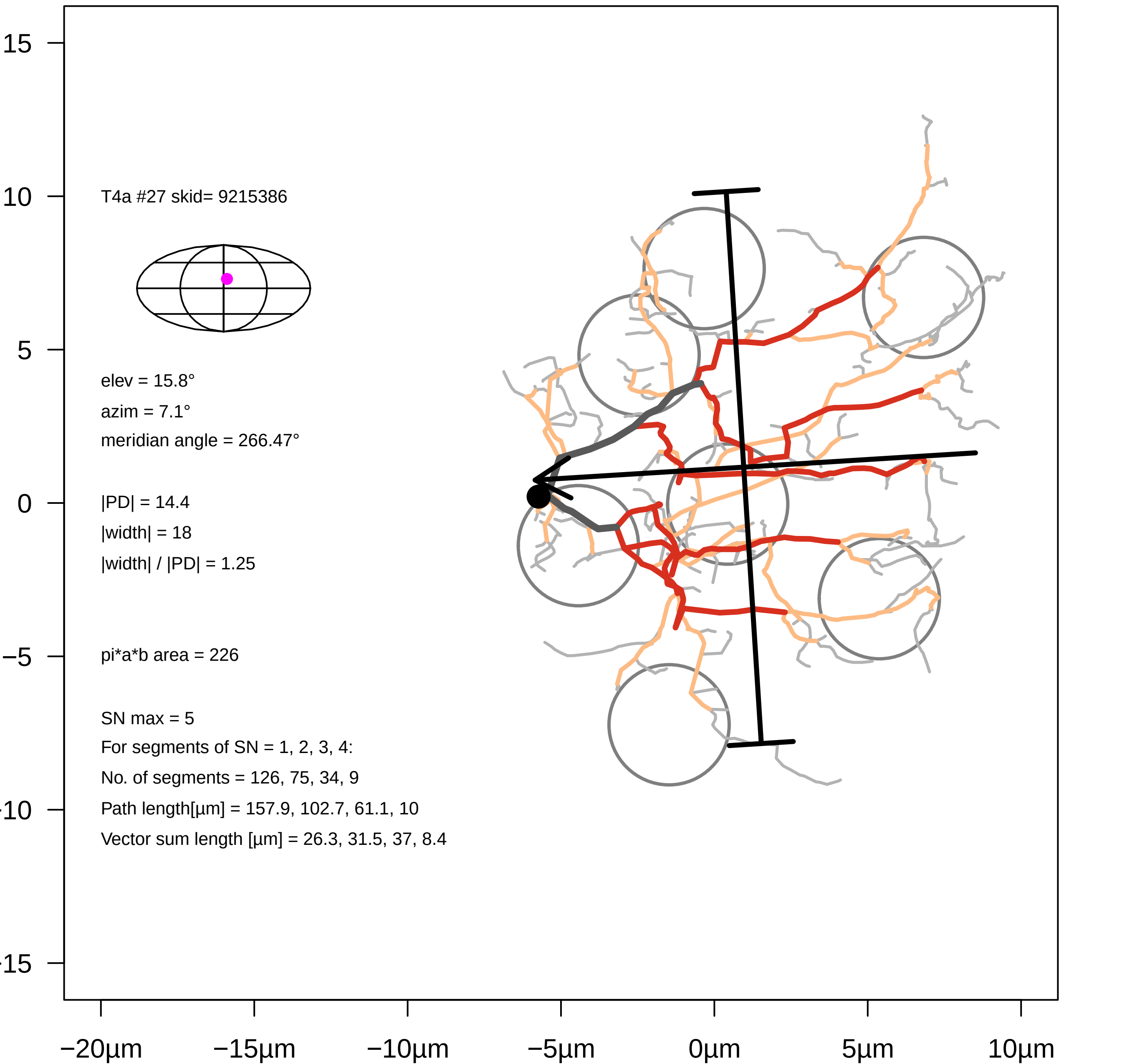
T4a #27 skid= 9215386
elev = 15.8°
azim = 7.1°
meridian angle = 266.47°
|PD| = 14.4
|width| = 18
|width| / |PD| = 1.25
pi*a*b area = 226
SN max = 5
For segments of SN = 1, 2, 3, 4:
No. of segments = 126, 75, 34, 9
Path length[µm] = 157.9, 102.7, 61.1, 10
Vector sum length [µm] = 26.3, 31.5, 37, 8.4
15
10
5
0
−5
−10
−15
−20µm
−15µm
−10µm
−5µm
0µm
5µm
10µm

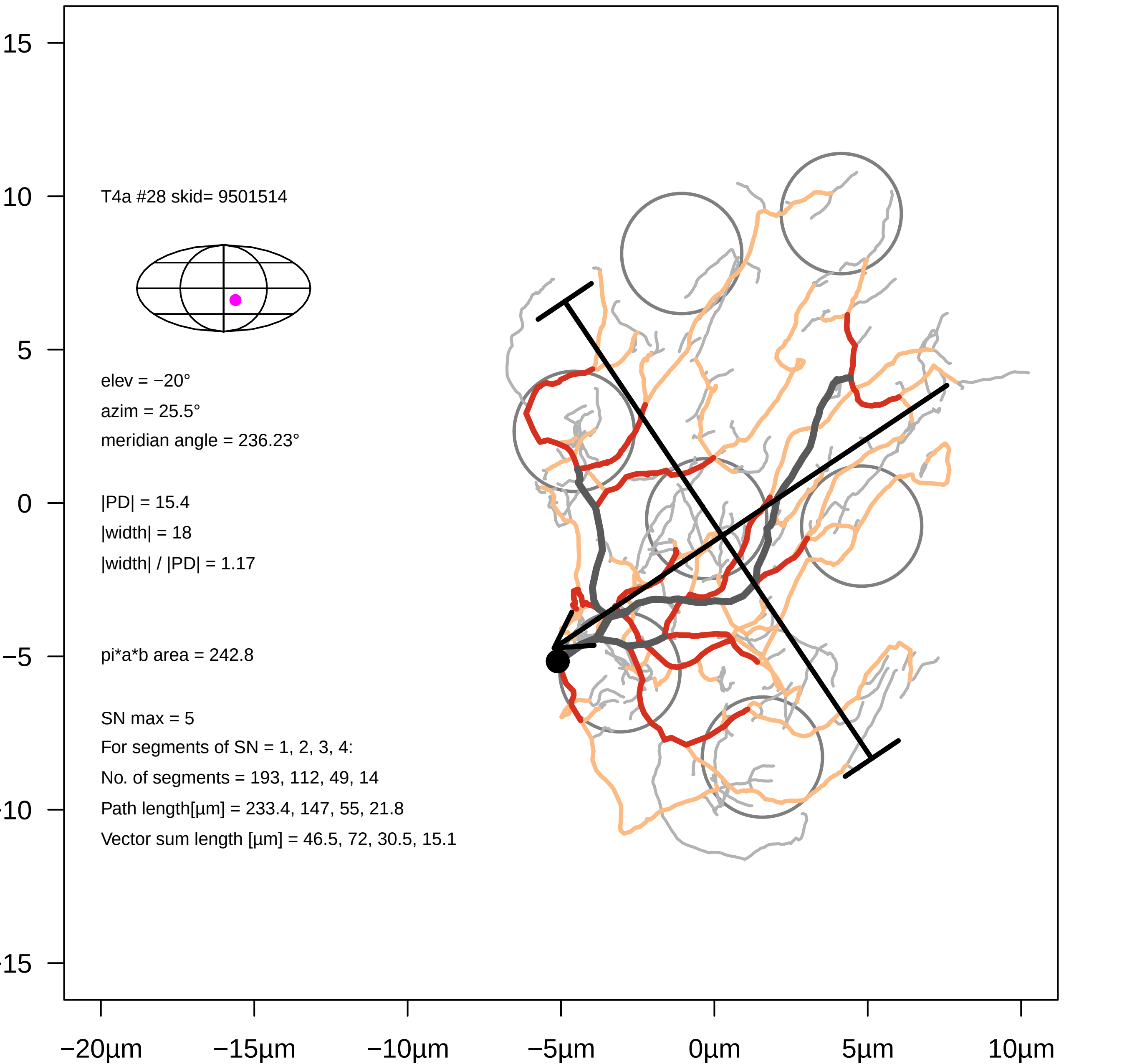

T4a #28 skid= 9501514
elev = −20°
azim = 25.5°
meridian angle = 236.23°
|PD| = 15.4
|width| = 18
|width| / |PD| = 1.17
pi*a*b area = 242.8
SN max = 5
For segments of SN = 1, 2, 3, 4:
No. of segments = 193, 112, 49, 14
Path length[µm] = 233.4, 147, 55, 21.8
Vector sum length [µm] = 46.5, 72, 30.5, 15.1
15
10
5
0
−5
−10
−15
−20µm
−15µm
−10µm
−5µm
0µm
5µm
10µm

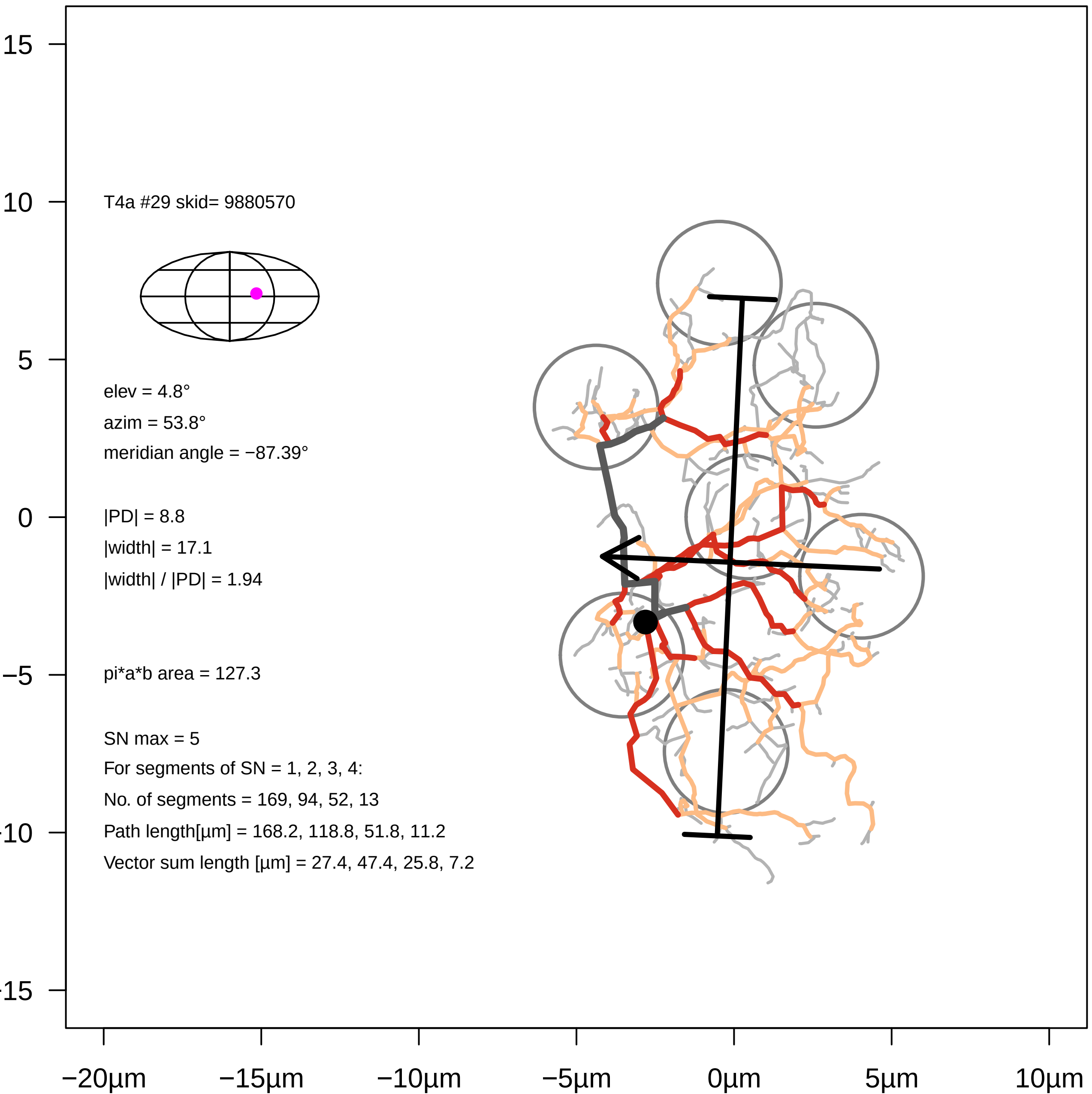

T4a #29 skid= 9880570
elev = 4.8°
azim = 53.8°
meridian angle = −87.39°
|PD| = 8.8
|width| = 17.1
|width| / |PD| = 1.94
pi*a*b area = 127.3
SN max = 5
For segments of SN = 1, 2, 3, 4:
No. of segments = 169, 94, 52, 13
Path length[µm] = 168.2, 118.8, 51.8, 11.2
Vector sum length [µm] = 27.4, 47.4, 25.8, 7.2
15
10
5
0
−5
−10
−15
−20µm
−15µm
−10µm
−5µm
0µm
5µm
10µm

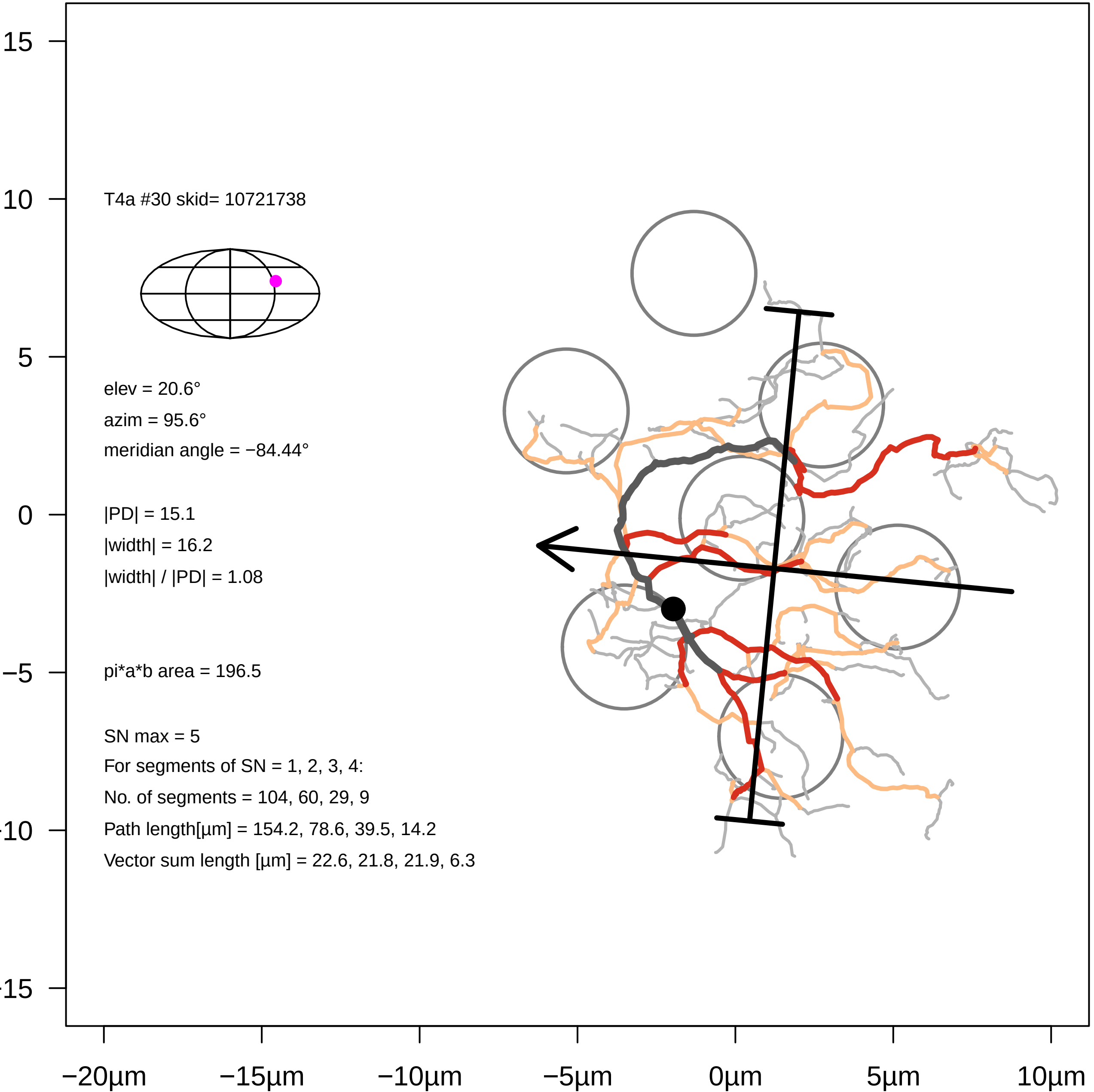
T4a #30 skid= 10721738
elev = 20.6°
azim = 95.6°
meridian angle = −84.44°
|PD| = 15.1
|width| = 16.2
|width| / |PD| = 1.08
pi*a*b area = 196.5
SN max = 5
For segments of SN = 1, 2, 3, 4:
No. of segments = 104, 60, 29, 9
Path length[µm] = 154.2, 78.6, 39.5, 14.2
Vector sum length [µm] = 22.6, 21.8, 21.9, 6.3
15
10
5
0
−5
−10
−15
−20µm
−15µm
−10µm
−5µm
0µm
5µm
10µm

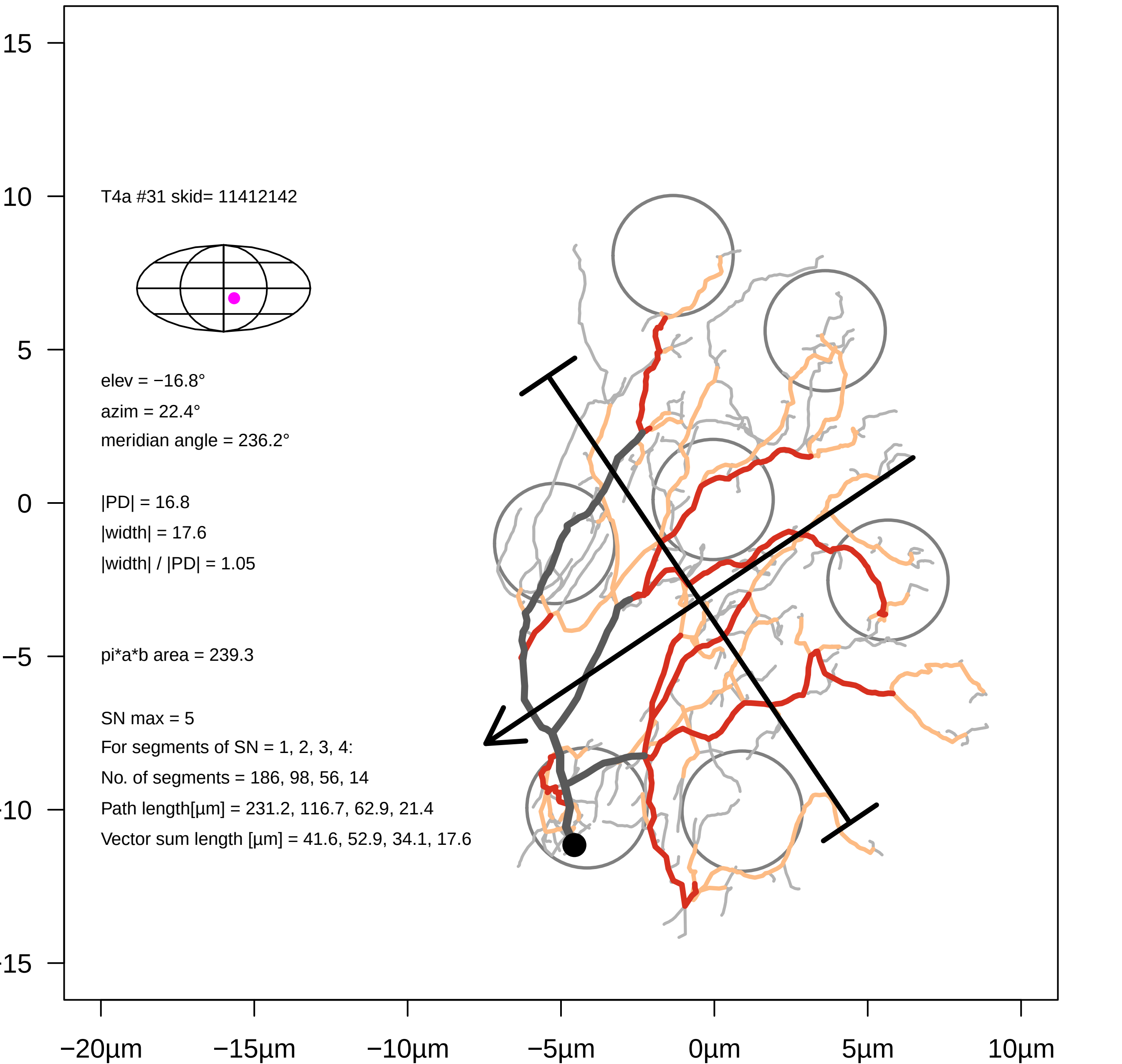
T4a #31 skid= 11412142
elev = −16.8°
azim = 22.4°
meridian angle = 236.2°
|PD| = 16.8
|width| = 17.6
|width| / |PD| = 1.05
pi*a*b area = 239.3
SN max = 5
For segments of SN = 1, 2, 3, 4:
No. of segments = 186, 98, 56, 14
Path length[µm] = 231.2, 116.7, 62.9, 21.4
Vector sum length [µm] = 41.6, 52.9, 34.1, 17.6
15
10
5
0
−5
−10
−15
−20µm
−15µm
−10µm
−5µm
0µm
5µm
10µm

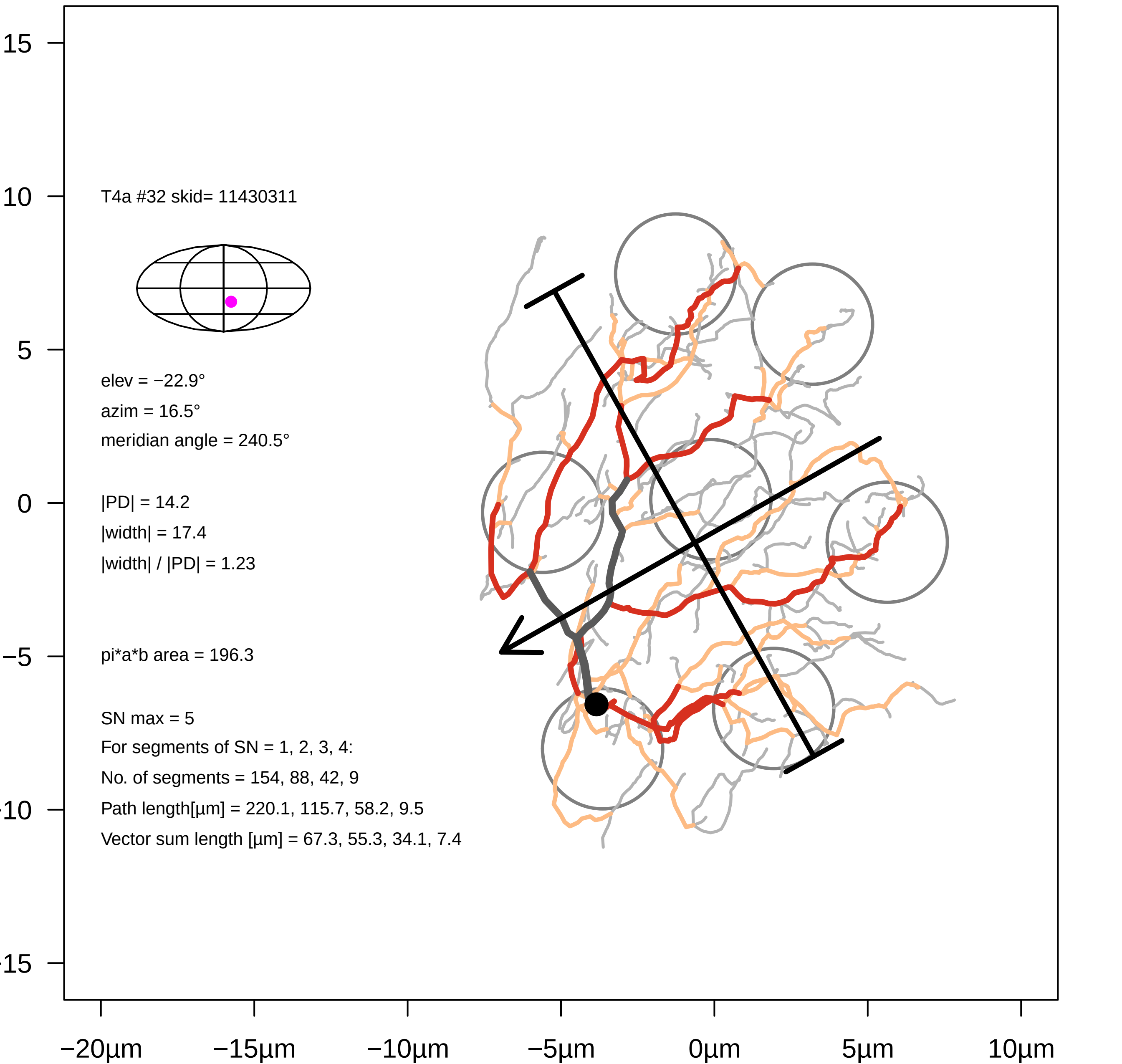
T4a #32 skid= 11430311
elev = −22.9°
azim = 16.5°
meridian angle = 240.5°
|PD| = 14.2
|width| = 17.4
|width| / |PD| = 1.23
pi*a*b area = 196.3
SN max = 5
For segments of SN = 1, 2, 3, 4:
No. of segments = 154, 88, 42, 9
Path length[µm] = 220.1, 115.7, 58.2, 9.5
Vector sum length [µm] = 67.3, 55.3, 34.1, 7.4
15
10
5
0
−5
−20µm
−15µm
−10µm
−5µm
0µm
5µm
10µm

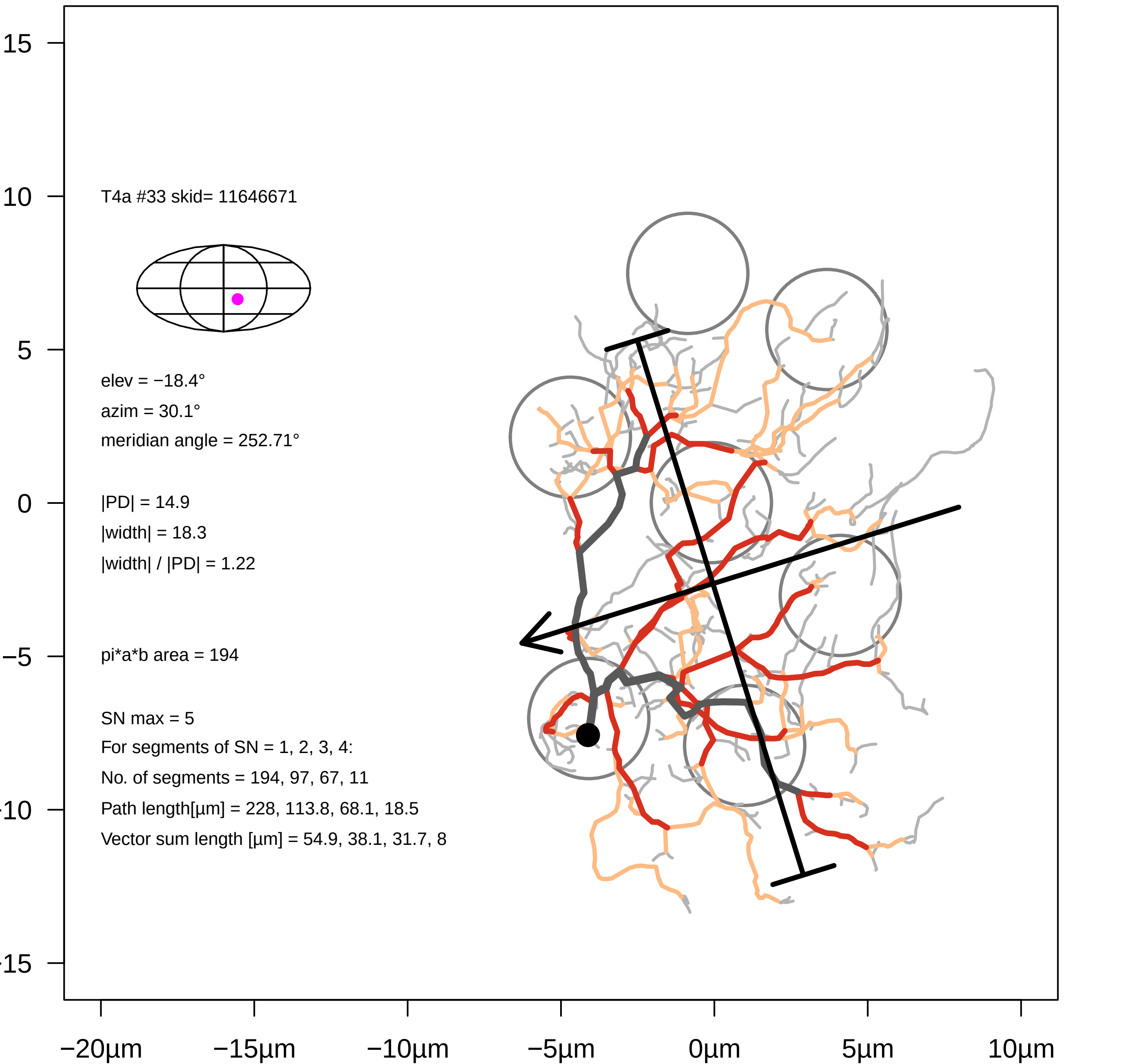

T4a #33 skid= 11646671
elev = −18.4°
azim = 30.1°
meridian angle = 252.71°
|PD| = 14.9
|width| = 18.3
|width| / |PD| = 1.22
pi*a*b area = 194
SN max = 5
For segments of SN = 1, 2, 3, 4:
No. of segments = 194, 97, 67, 11
Path length[µm] = 228, 113.8, 68.1, 18.5
Vector sum length [µm] = 54.9, 38.1, 31.7, 8
15
10
5
0
−5
-10
-15
−20µm
−15µm
−10µm
−5µm
0µm
5µm
10µm

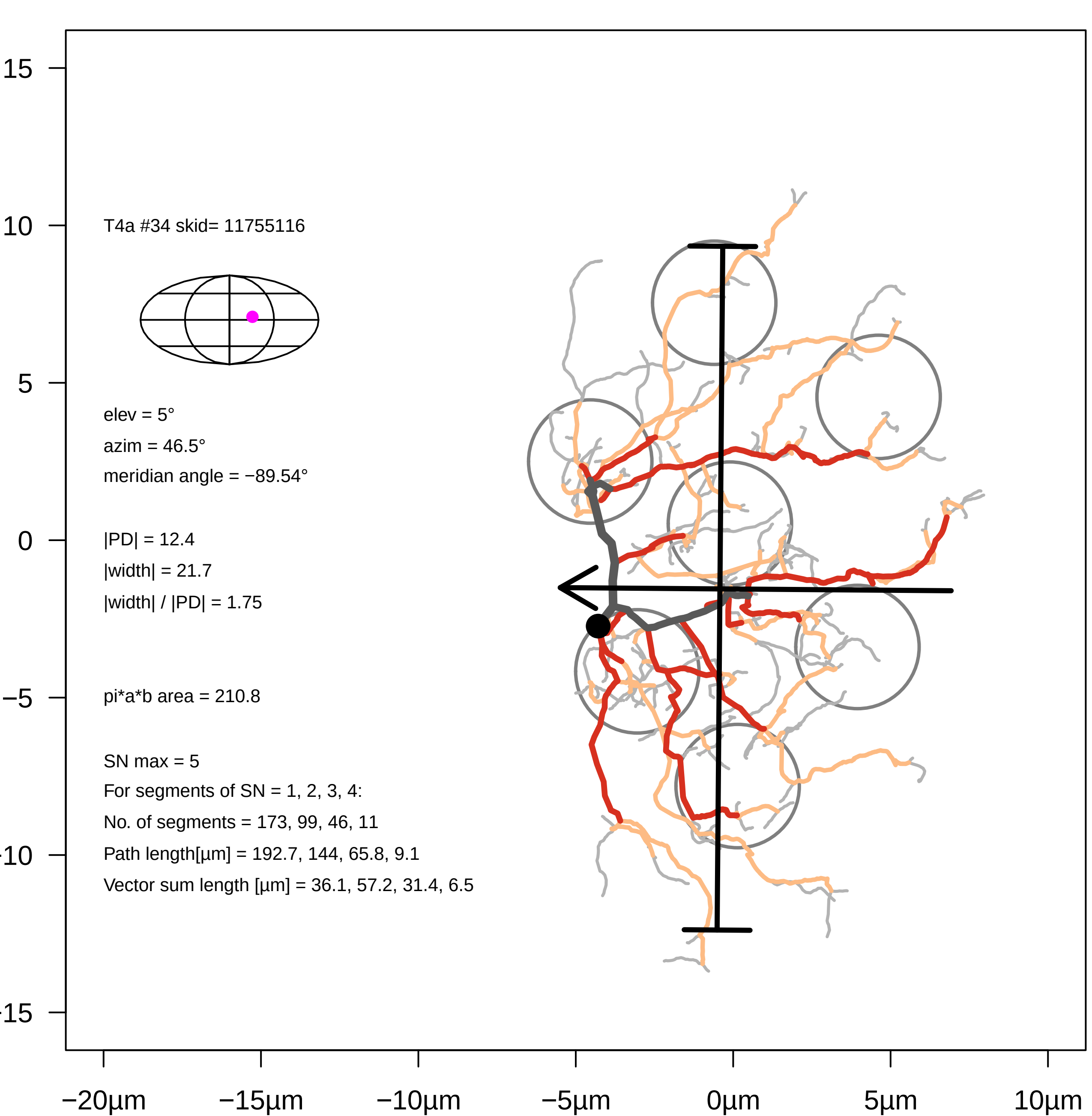
T4a #34 skid= 11755116
elev = 5°
azim = 46.5°
meridian angle = −89.54°
|PD| = 12.4
|width| = 21.7
|width| / |PD| = 1.75
pi*a*b area = 210.8
SN max = 5
For segments of SN = 1, 2, 3, 4:
No. of segments = 173, 99, 46, 11
Path length[µm] = 192.7, 144, 65.8, 9.1
Vector sum length [µm] = 36.1, 57.2, 31.4, 6.5
15
10
5
0
−5
−10
−15
−20µm
−15µm
−10µm
−5µm
0µm
5µm
10µm

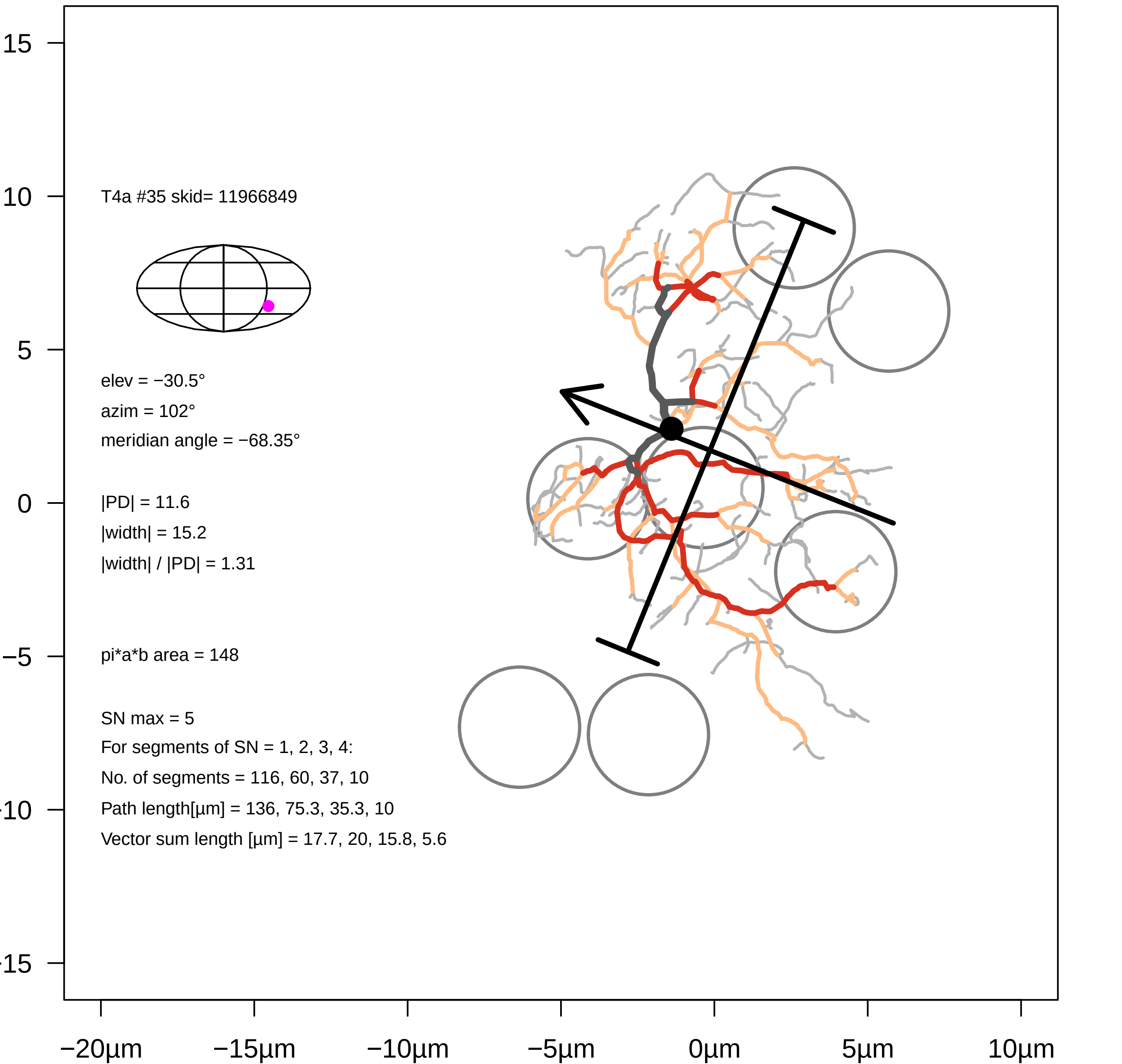

15
10
5
0
−5
10
15
T4a #35 skid= 11966849
elev = −30.5°
azim = 102°
meridian angle = −68.35°
|PD| = 11.6
|width| = 15.2
|width| / |PD| = 1.31
pi*a*b area = 148
SN max = 5
For segments of SN = 1, 2, 3, 4:
No. of segments = 116, 60, 37, 10
Path length[µm] = 136, 75.3, 35.3, 10
Vector sum length [µm] = 17.7, 20, 15.8, 5.6
−20µm
−15µm
−10µm
−5µm
0µm
5µm
10µm

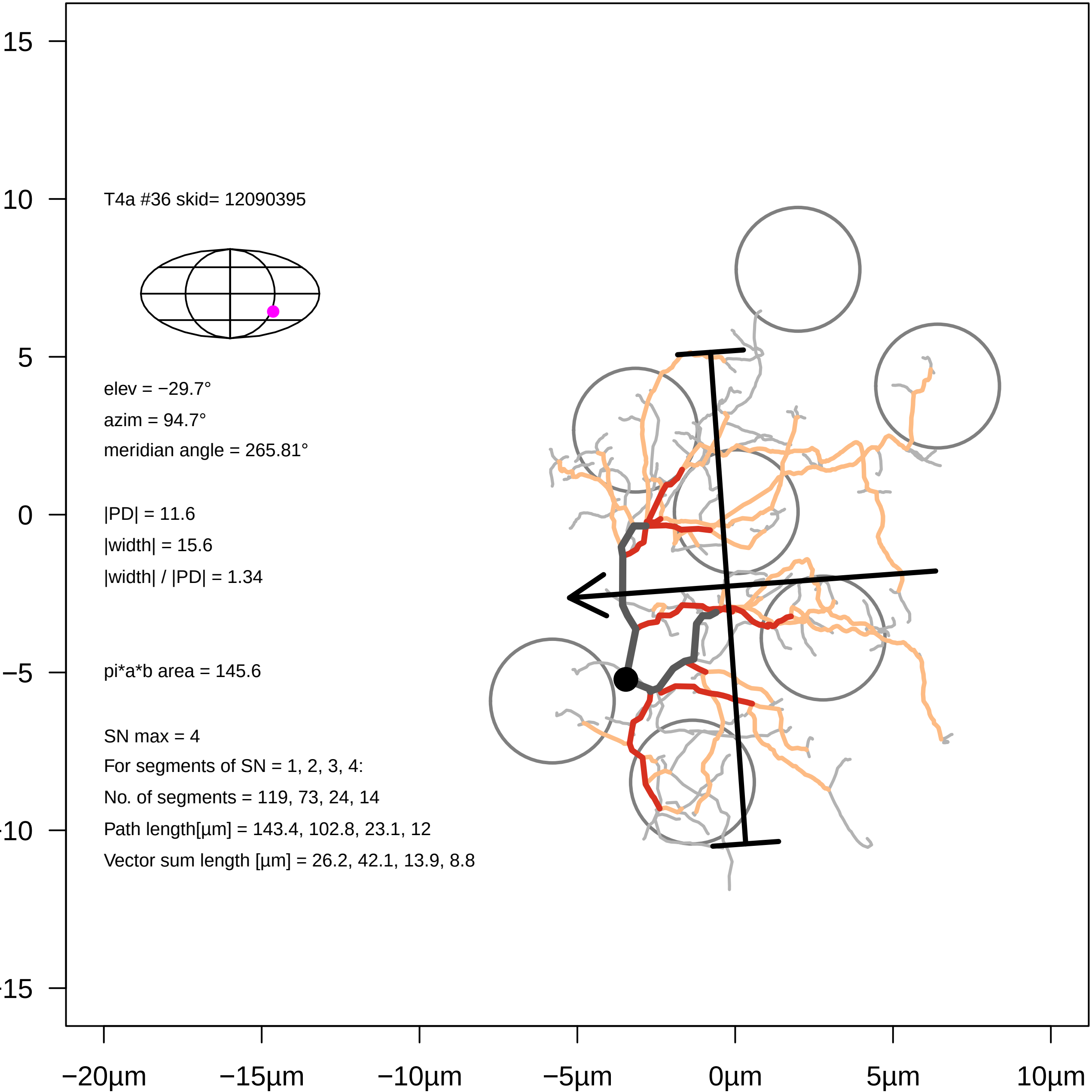
15
10
5
0
−5
−10
−15
−20µm
−15µm
−10µm
−5µm
0µm
5µm
10µm
T4a #36 skid= 12090395
elev = −29.7°
azim = 94.7°
meridian angle = 265.81°
|PD| = 11.6
|width| = 15.6
|width| / |PD| = 1.34
pi*a*b area = 145.6
SN max = 4
For segments of SN = 1, 2, 3, 4:
No. of segments = 119, 73, 24, 14
Path length[µm] = 143.4, 102.8, 23.1, 12
Vector sum length [µm] = 26.2, 42.1, 13.9, 8.8

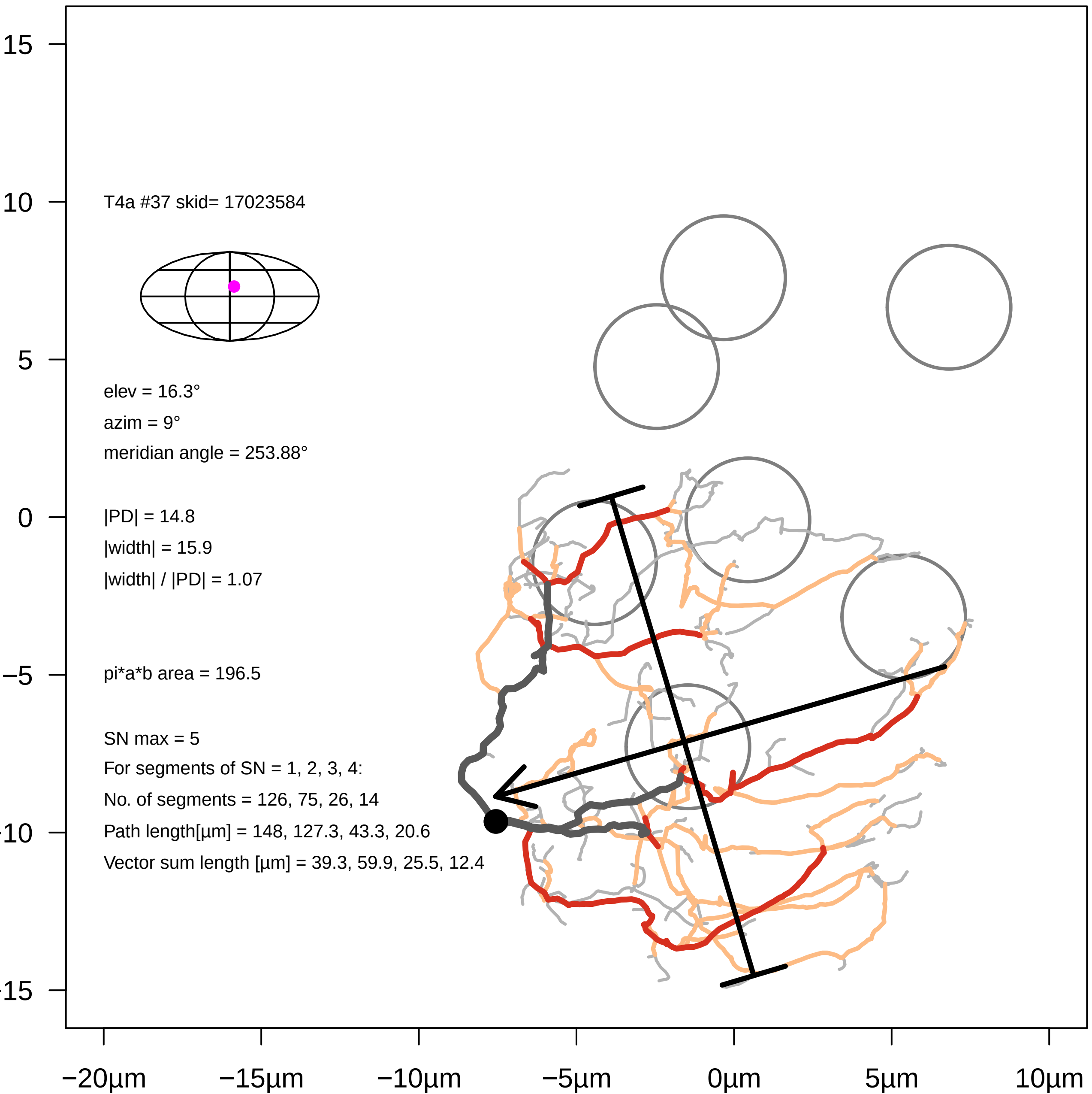

T4a #37 skid= 17023584
elev = 16.3°
azim = 9°
meridian angle = 253.88°
|PD| = 14.8
|width| = 15.9
|width| / |PD| = 1.07
pi*a*b area = 196.5
SN max = 5
For segments of SN = 1, 2, 3, 4:
No. of segments = 126, 75, 26, 14
Path length[µm] = 148, 127.3, 43.3, 20.6
Vector sum length [µm] = 39.3, 59.9, 25.5, 12.4
15
10
5
0
−5
−10
−15
−20µm
−15µm
−10µm
−5µm
0µm
5µm
10µm

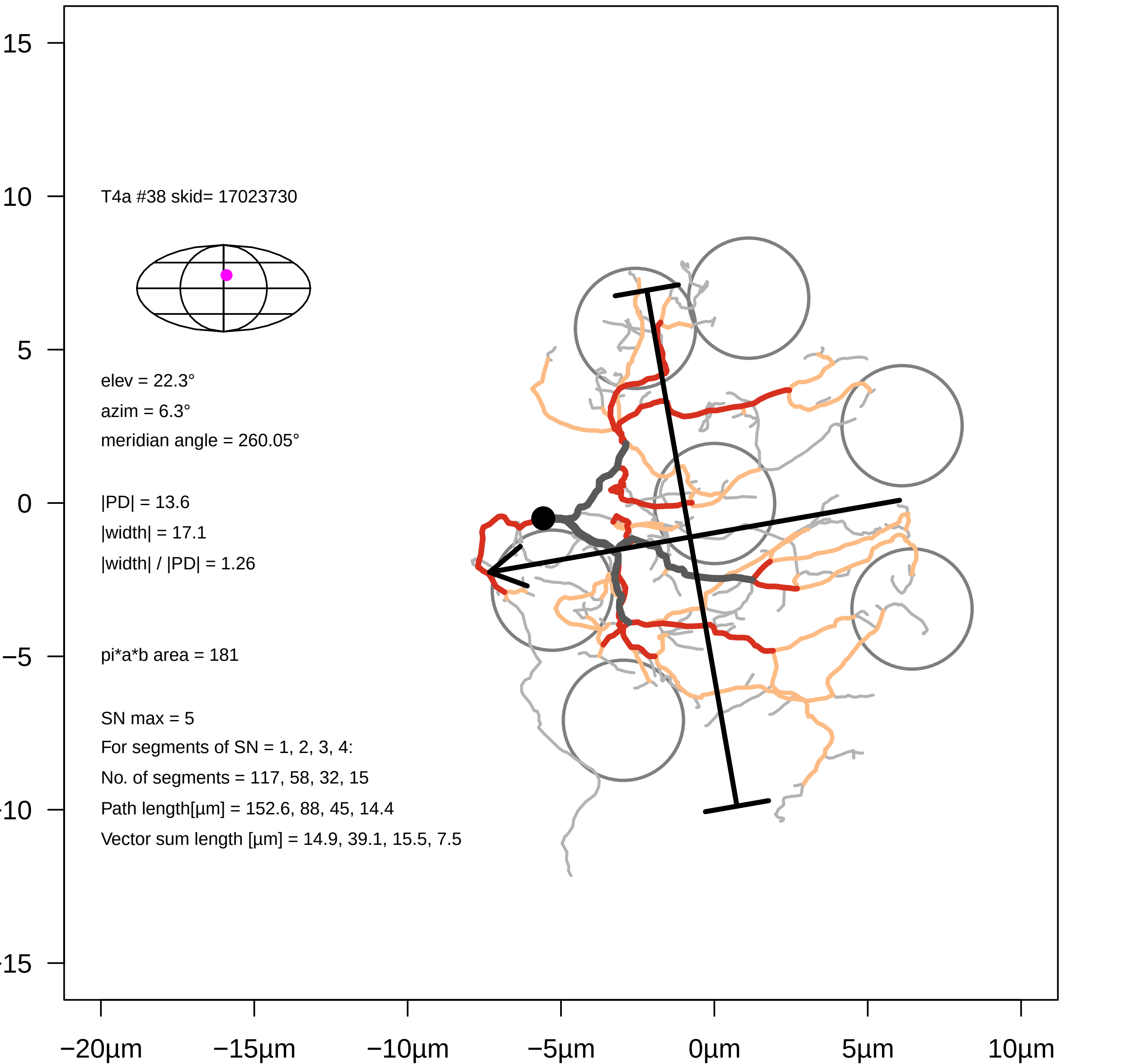
T4a #38 skid= 17023730
elev = 22.3°
azim = 6.3°
meridian angle = 260.05°
|PD| = 13.6
|width| = 17.1
|width| / |PD| = 1.26
pi*a*b area = 181
SN max = 5
For segments of SN = 1, 2, 3, 4:
No. of segments = 117, 58, 32, 15
Path length[µm] = 152.6, 88, 45, 14.4
Vector sum length [µm] = 14.9, 39.1, 15.5, 7.5
15
10
5
0
−5
−10
−15
−20µm
−15µm
−10µm
−5µm
0µm
5µm
10µm
