# Supplementary figures and images for: Eye structure shapes neuron function in Drosophila motion vision
Source: Nature. 2025 Jul 23;646(8083):135–42. doi: 10.1038/s41586-025-09276-5 (PMC12488493; doi:10.1038/s41586-025-09276-5)

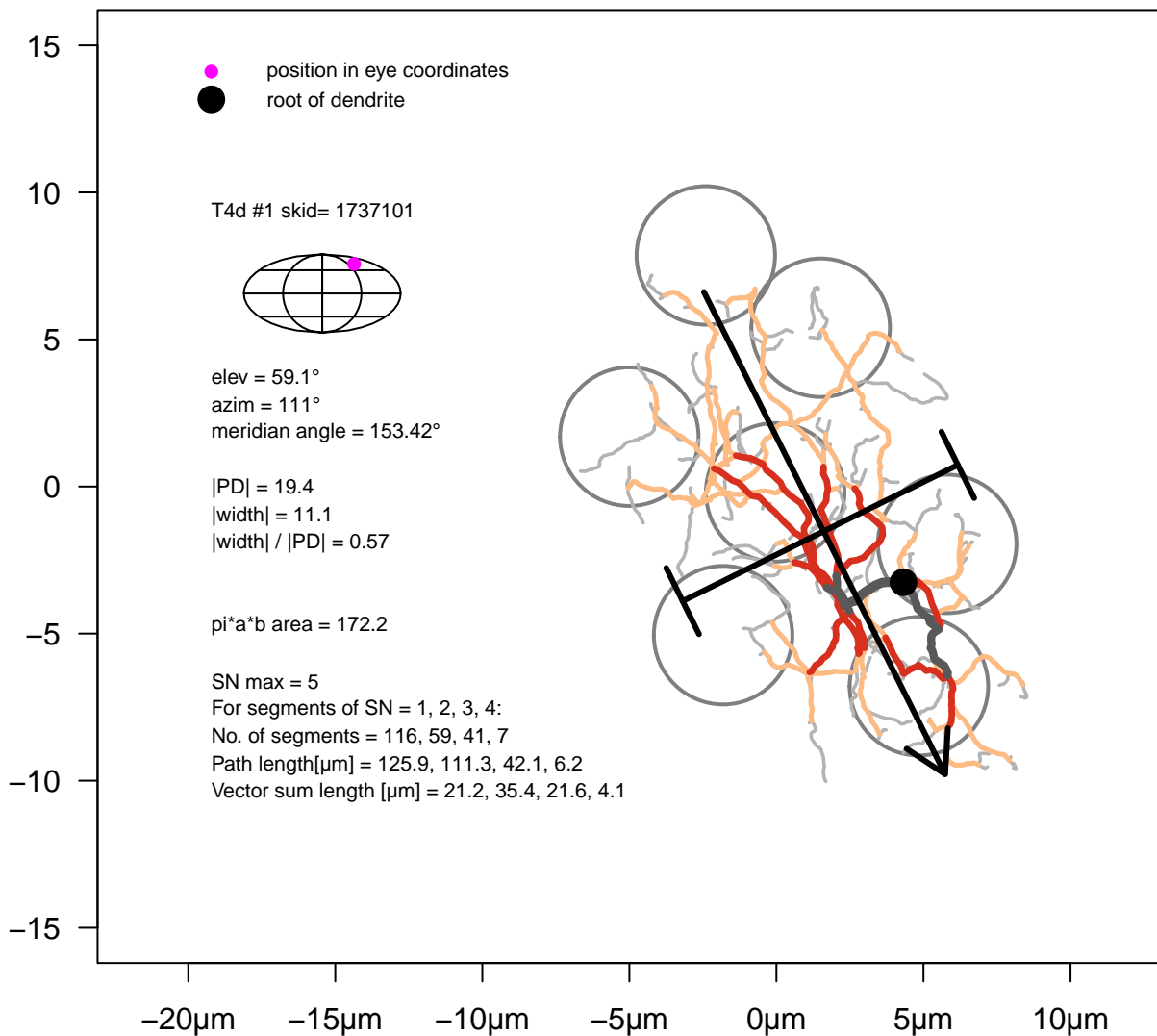

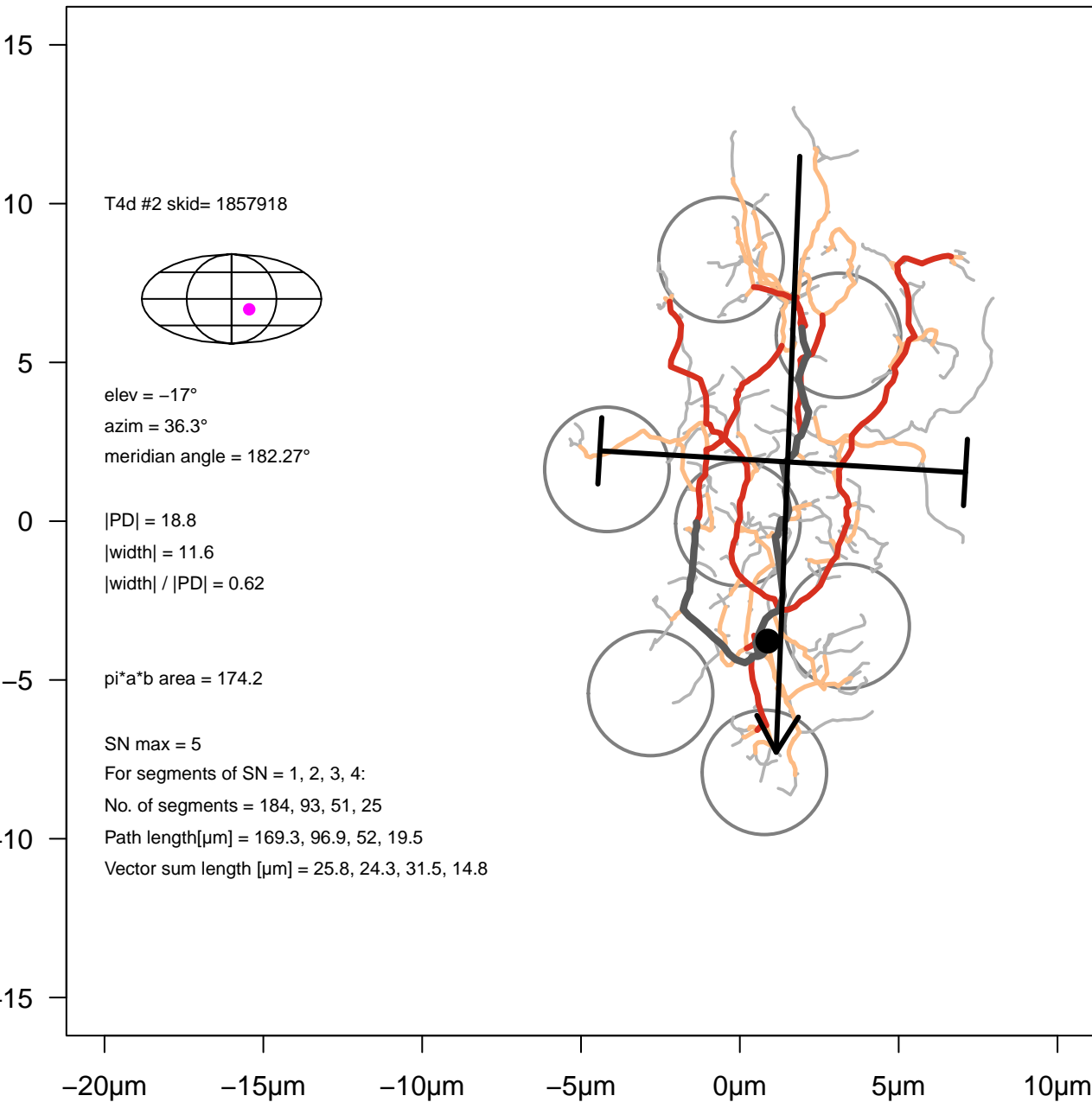

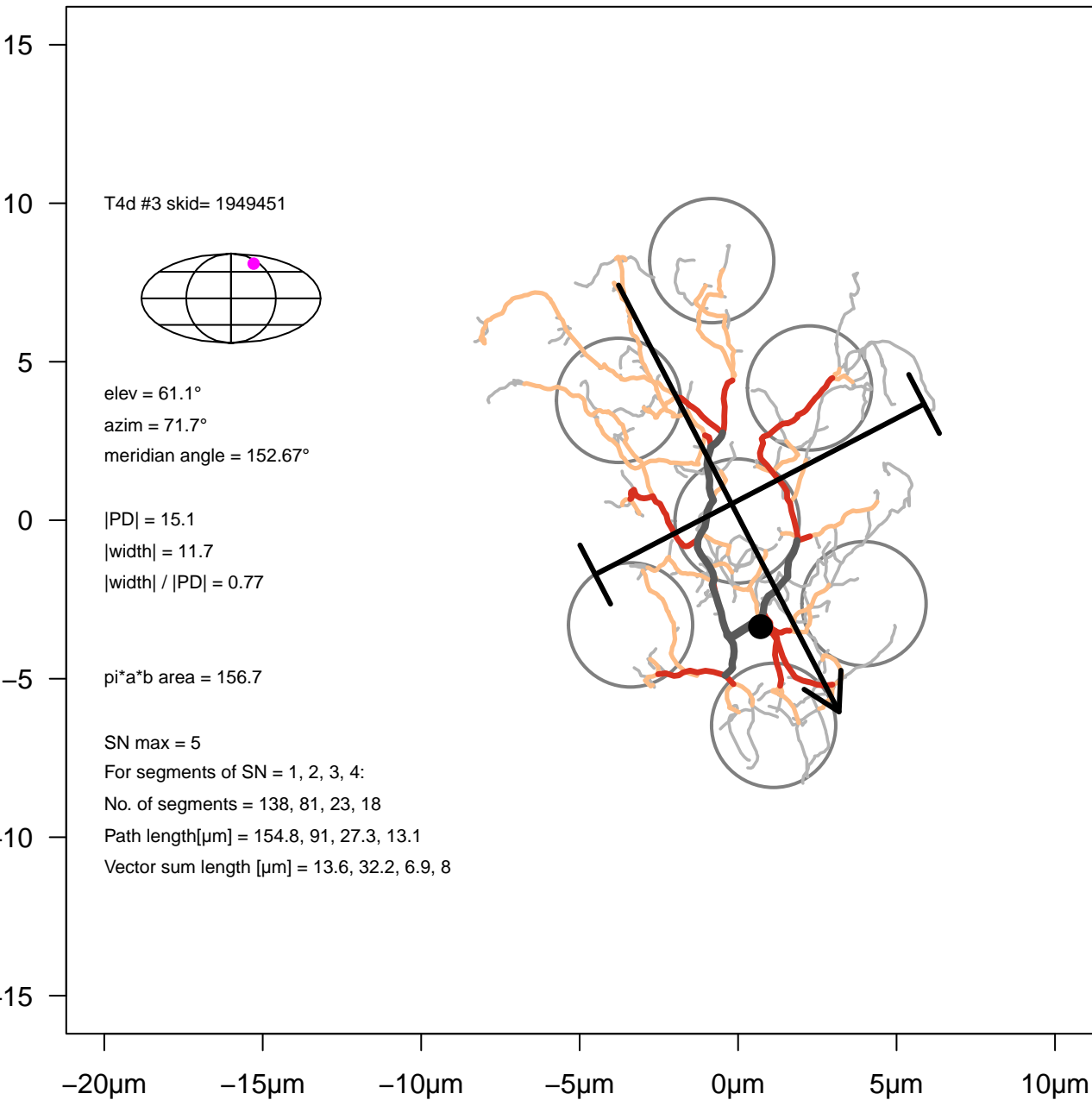

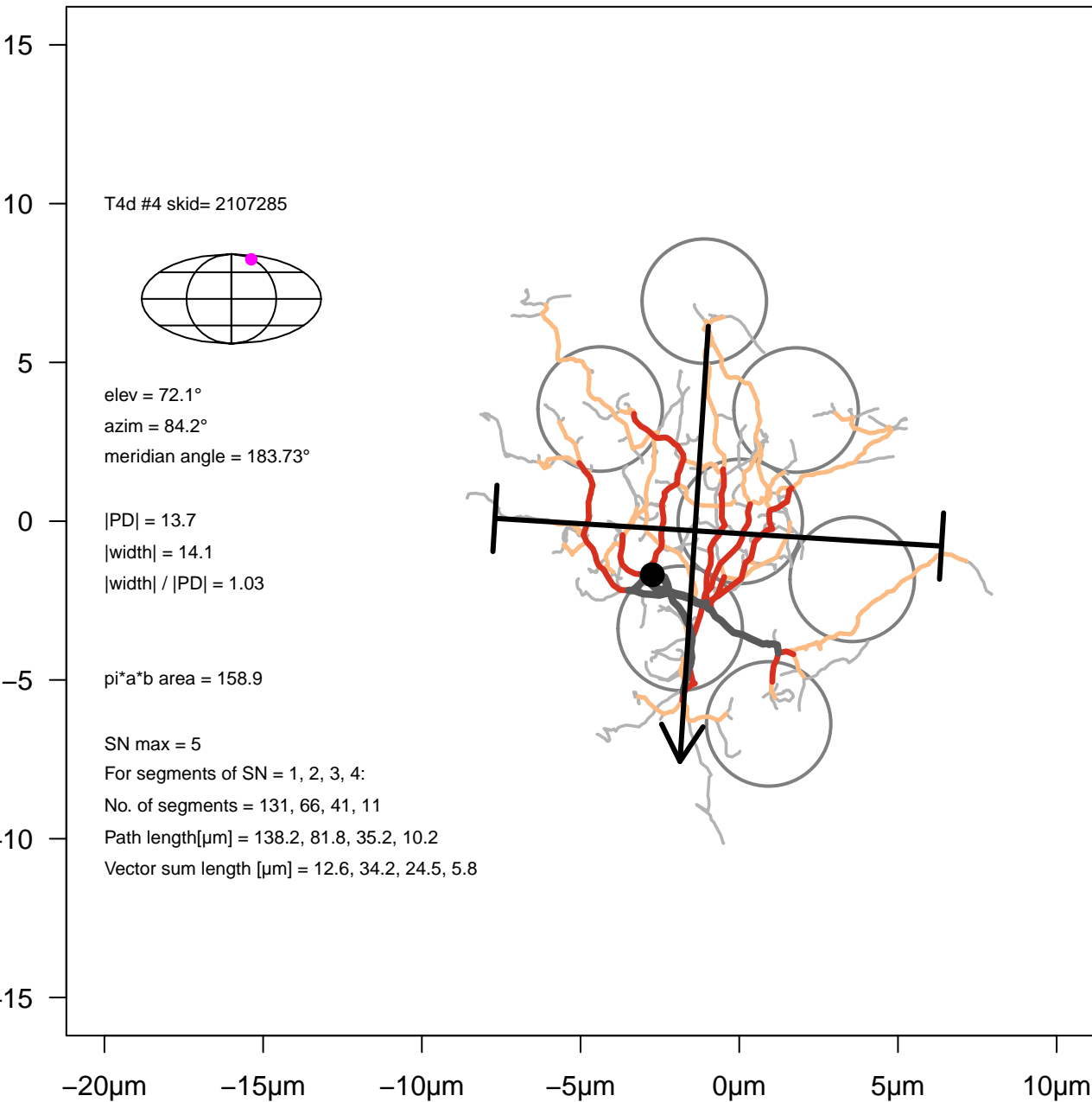

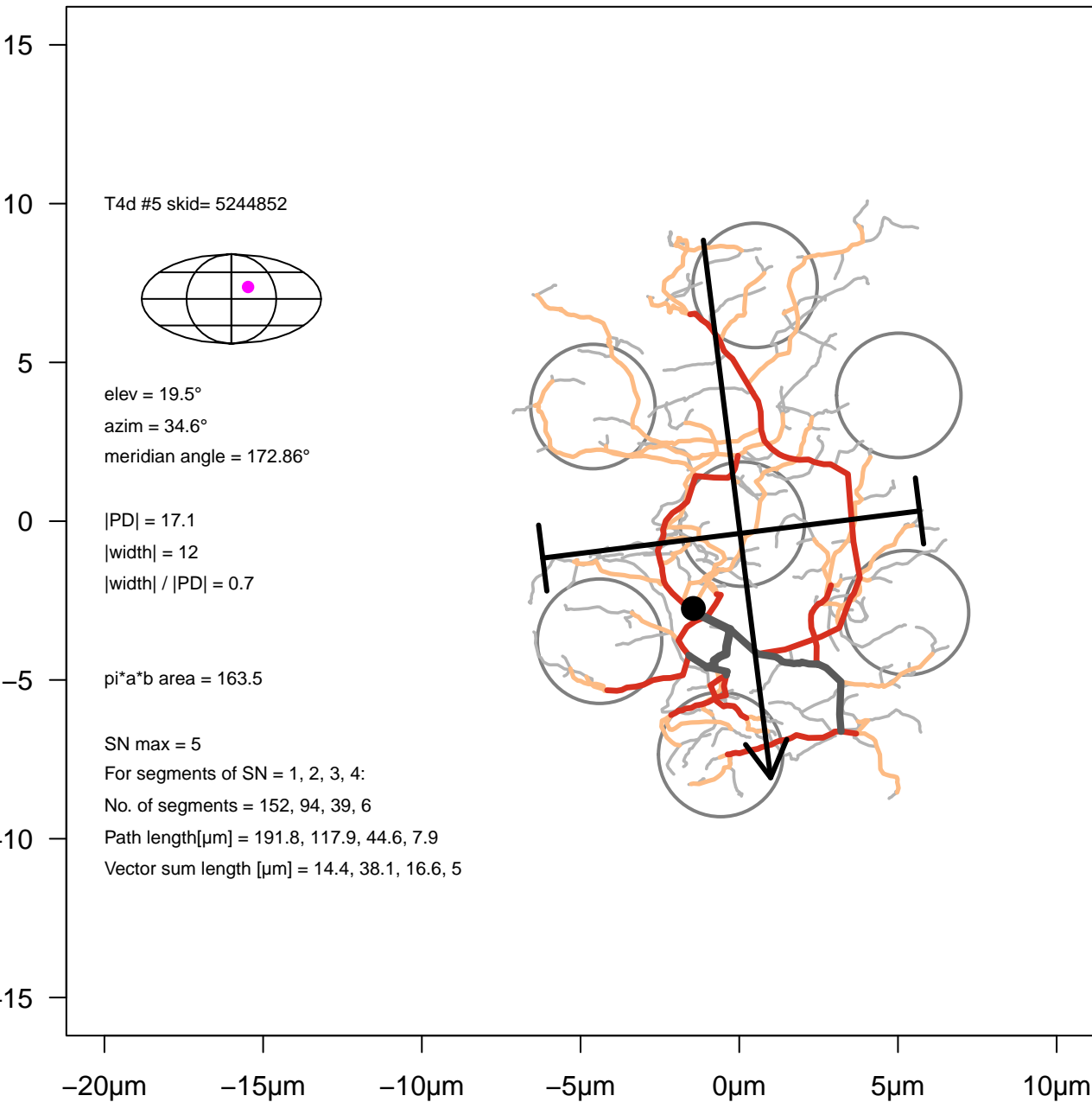

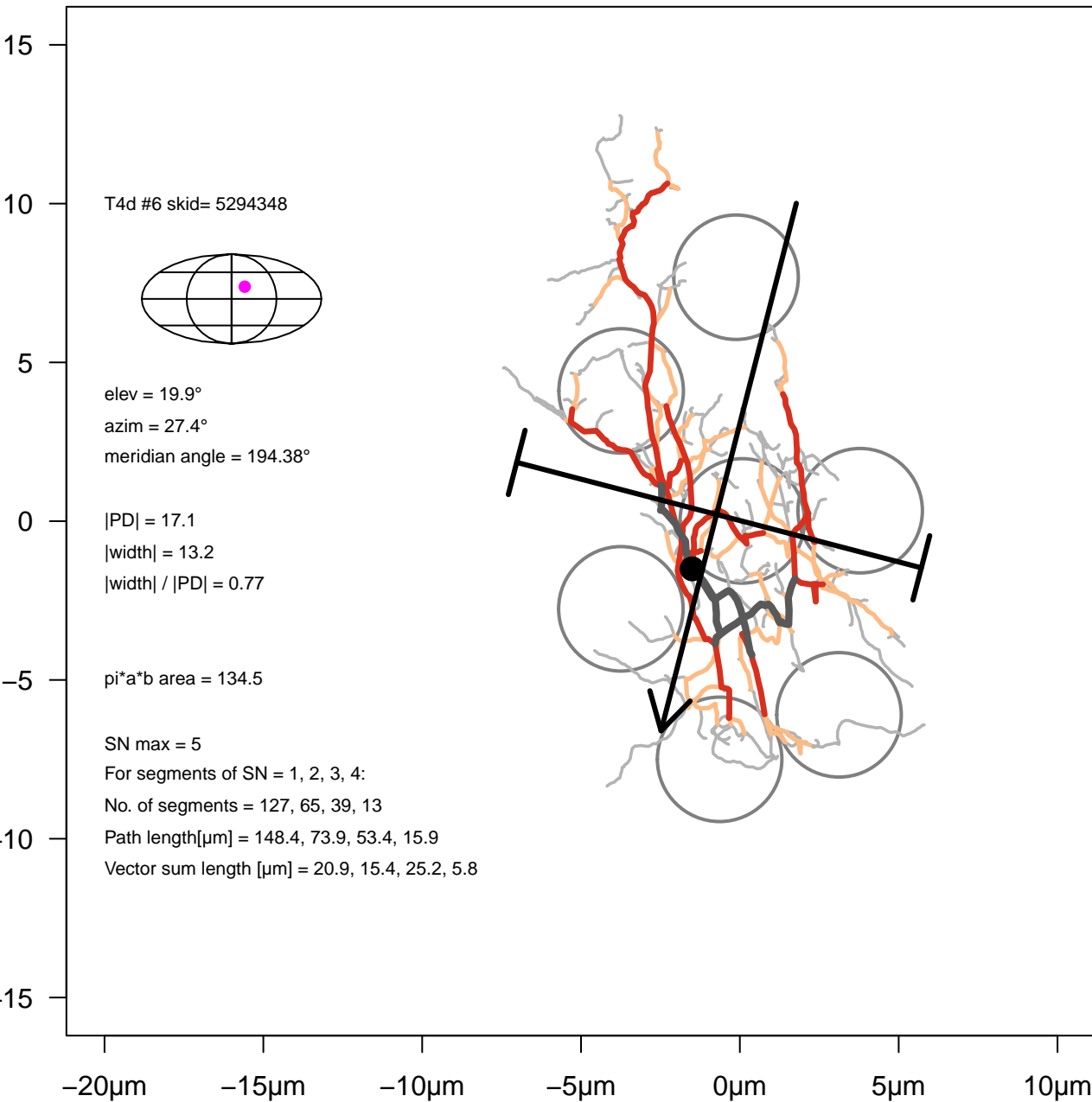

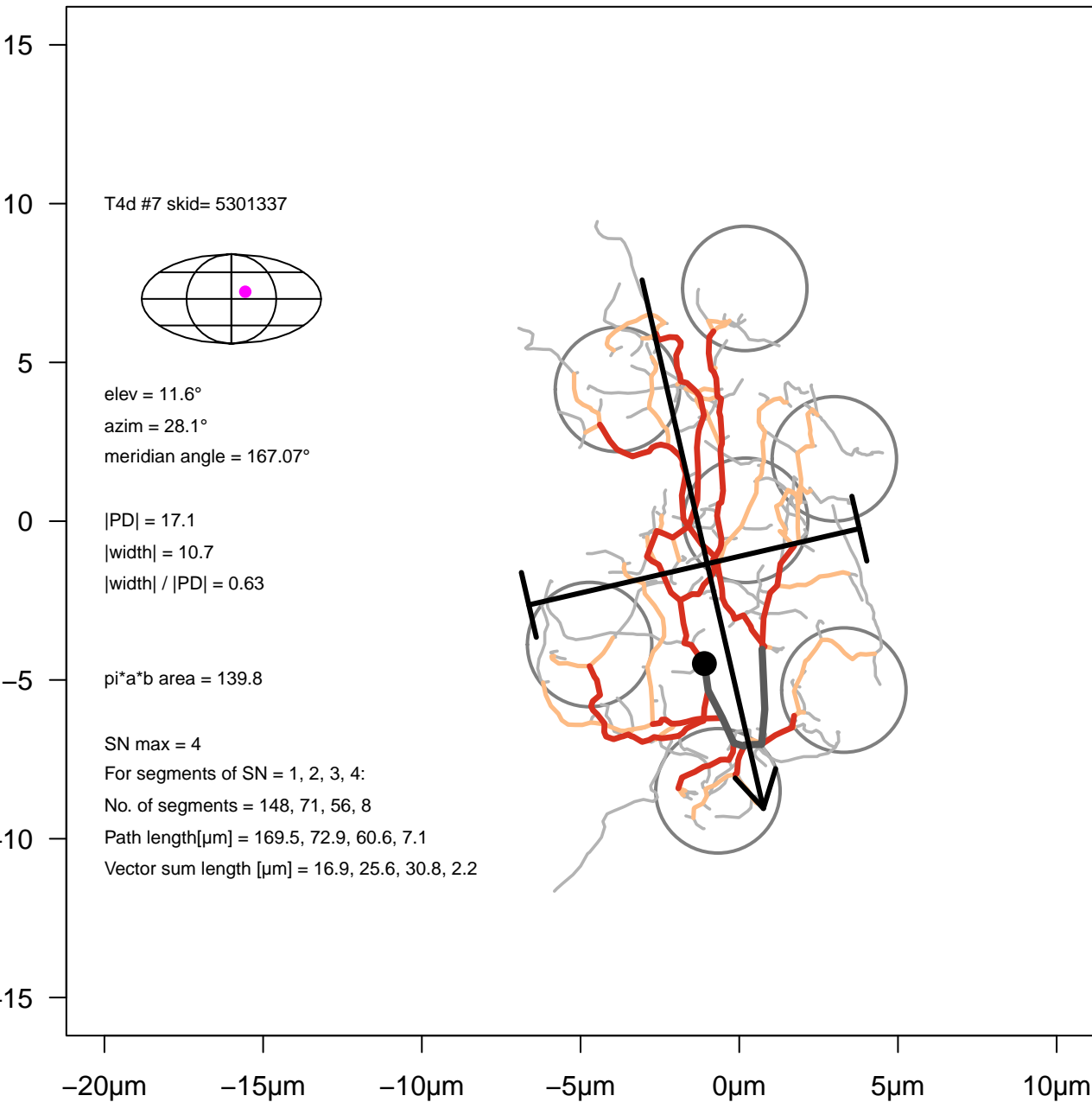

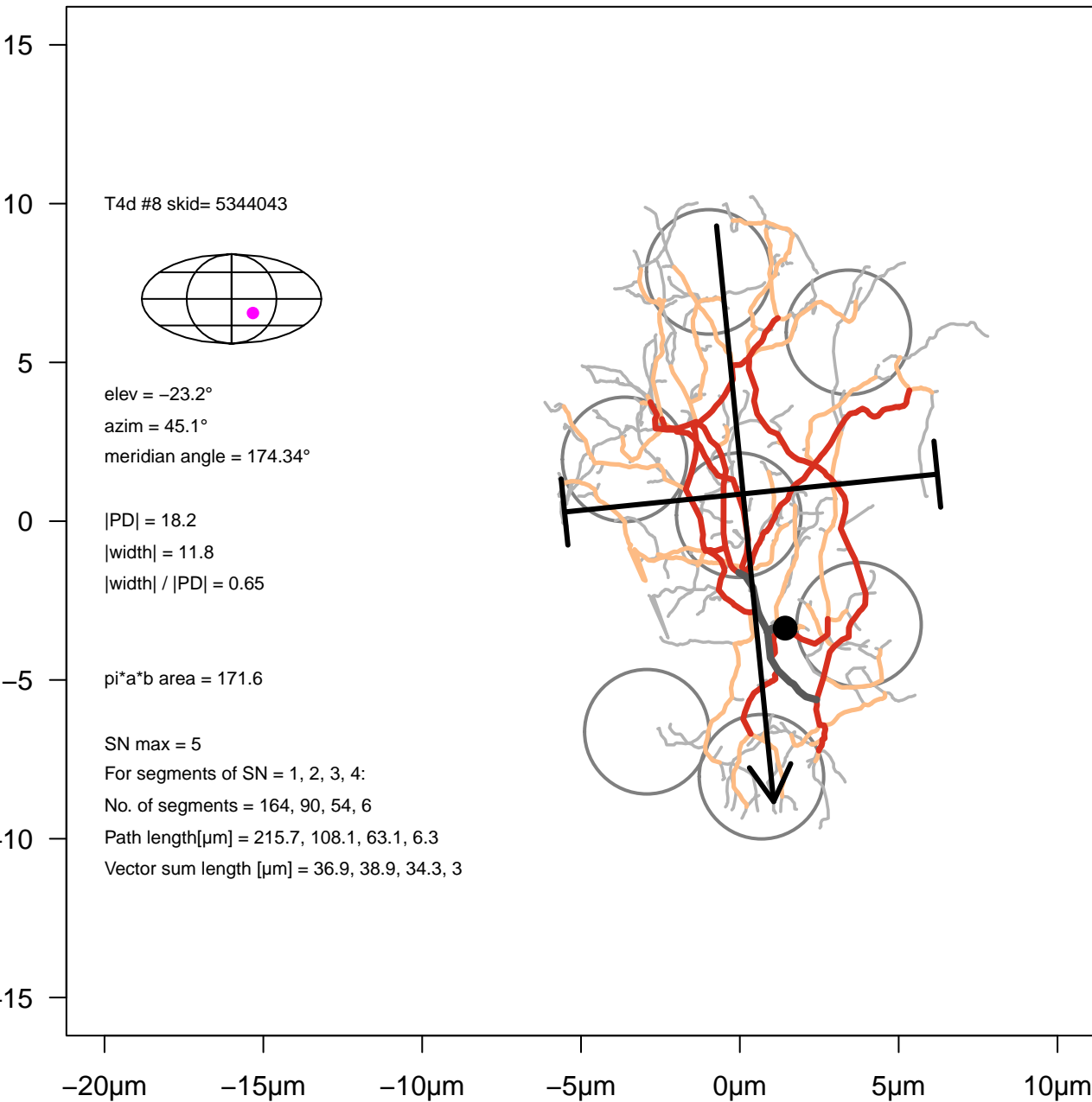

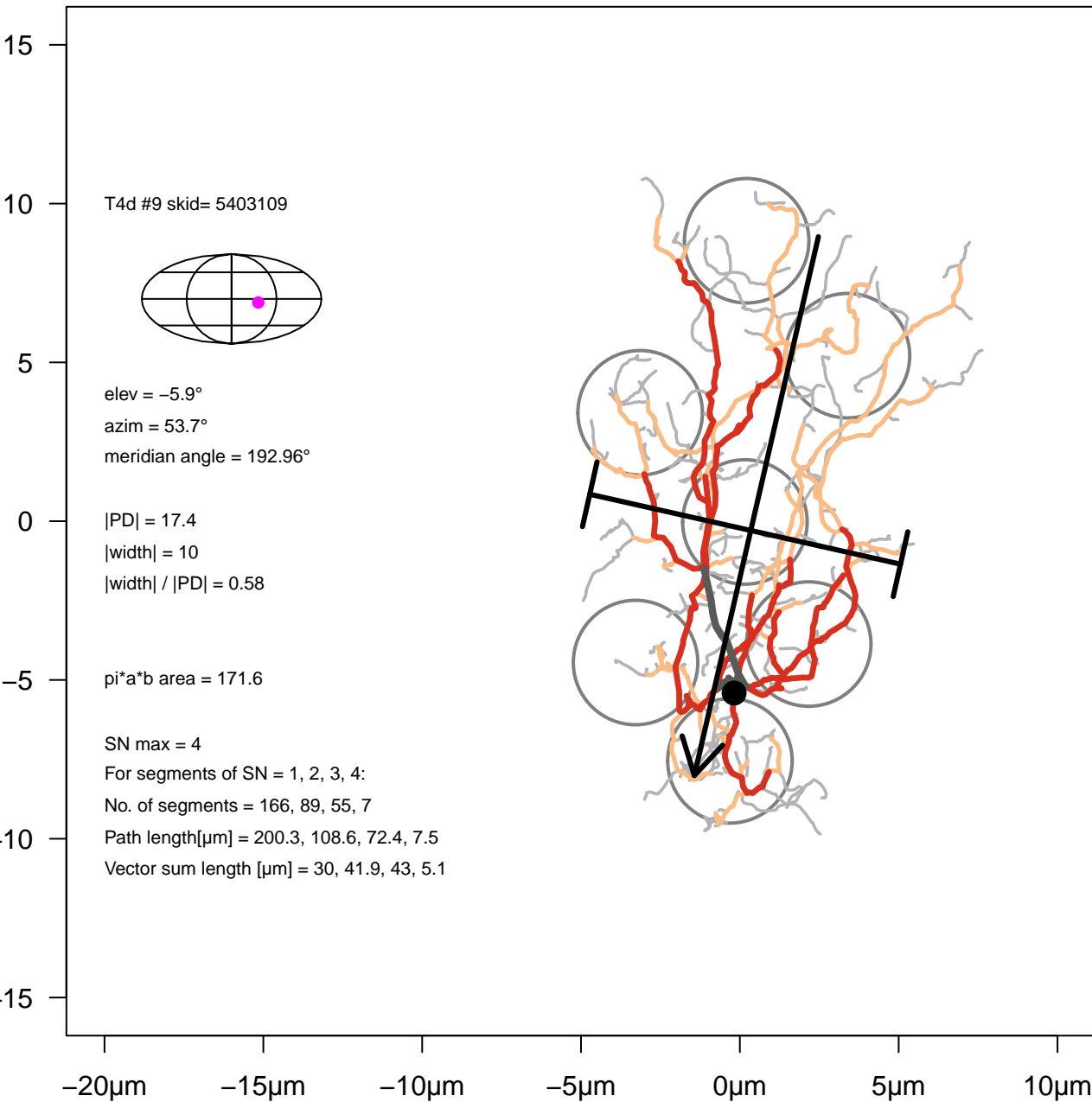

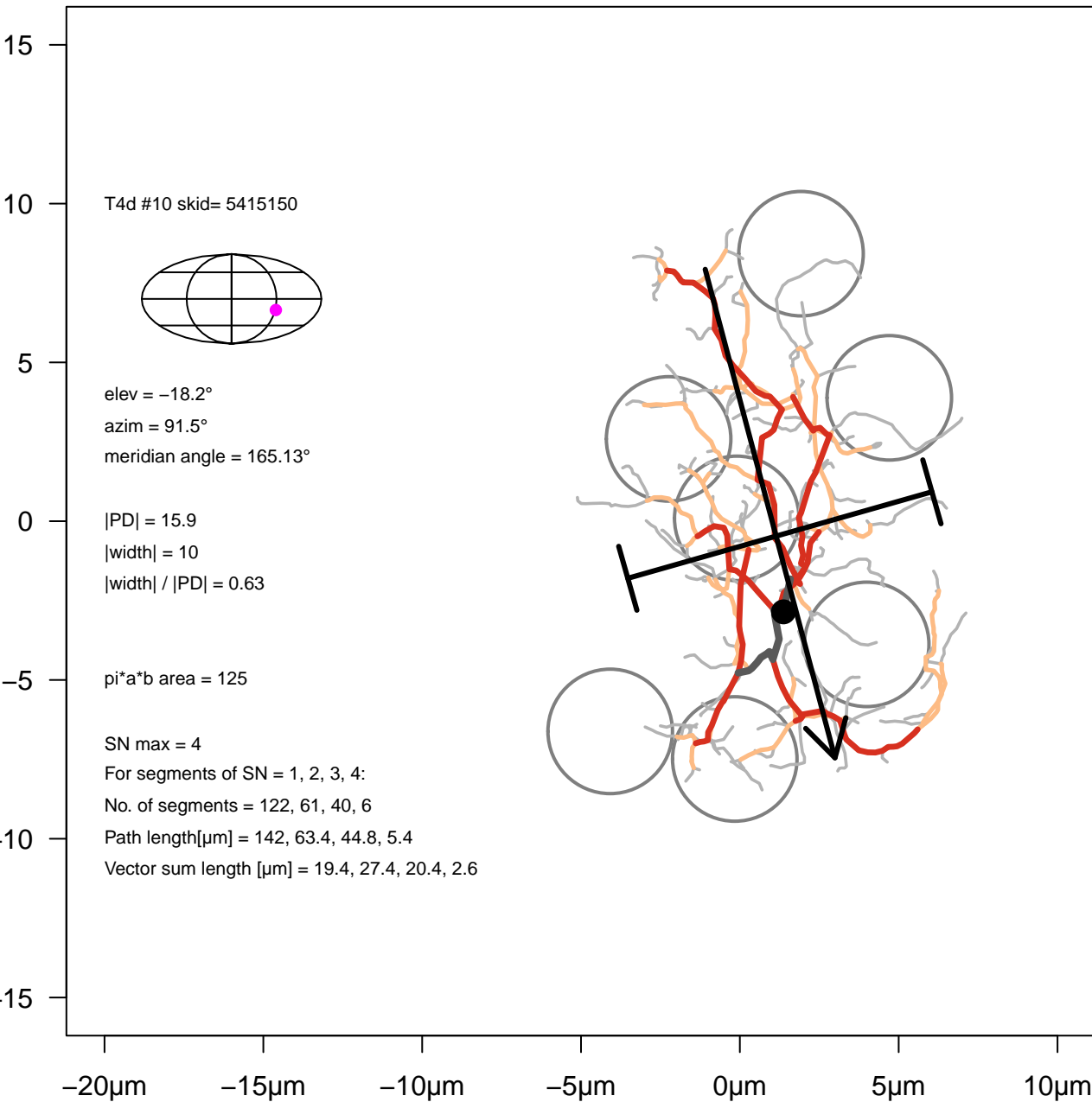

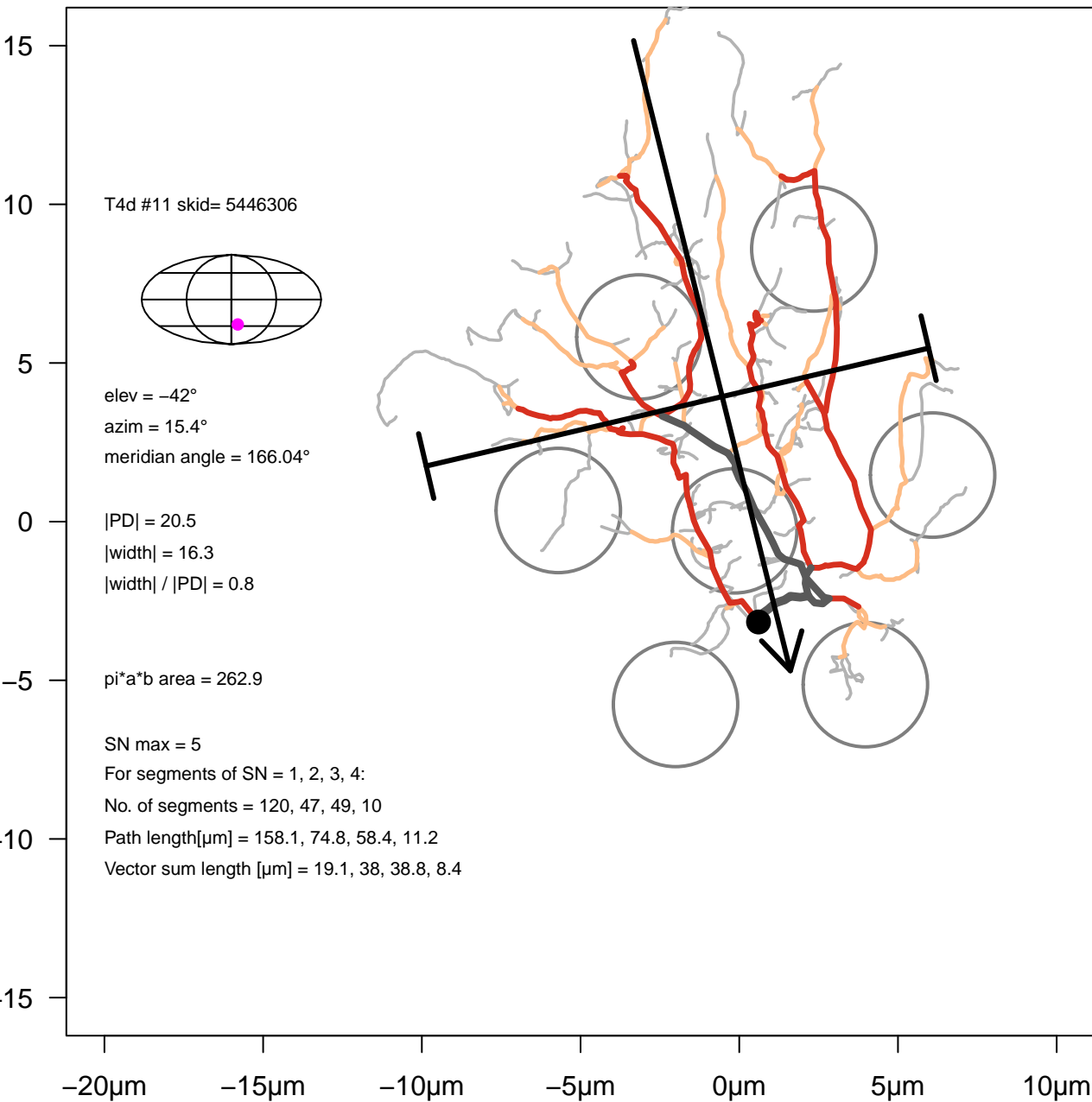

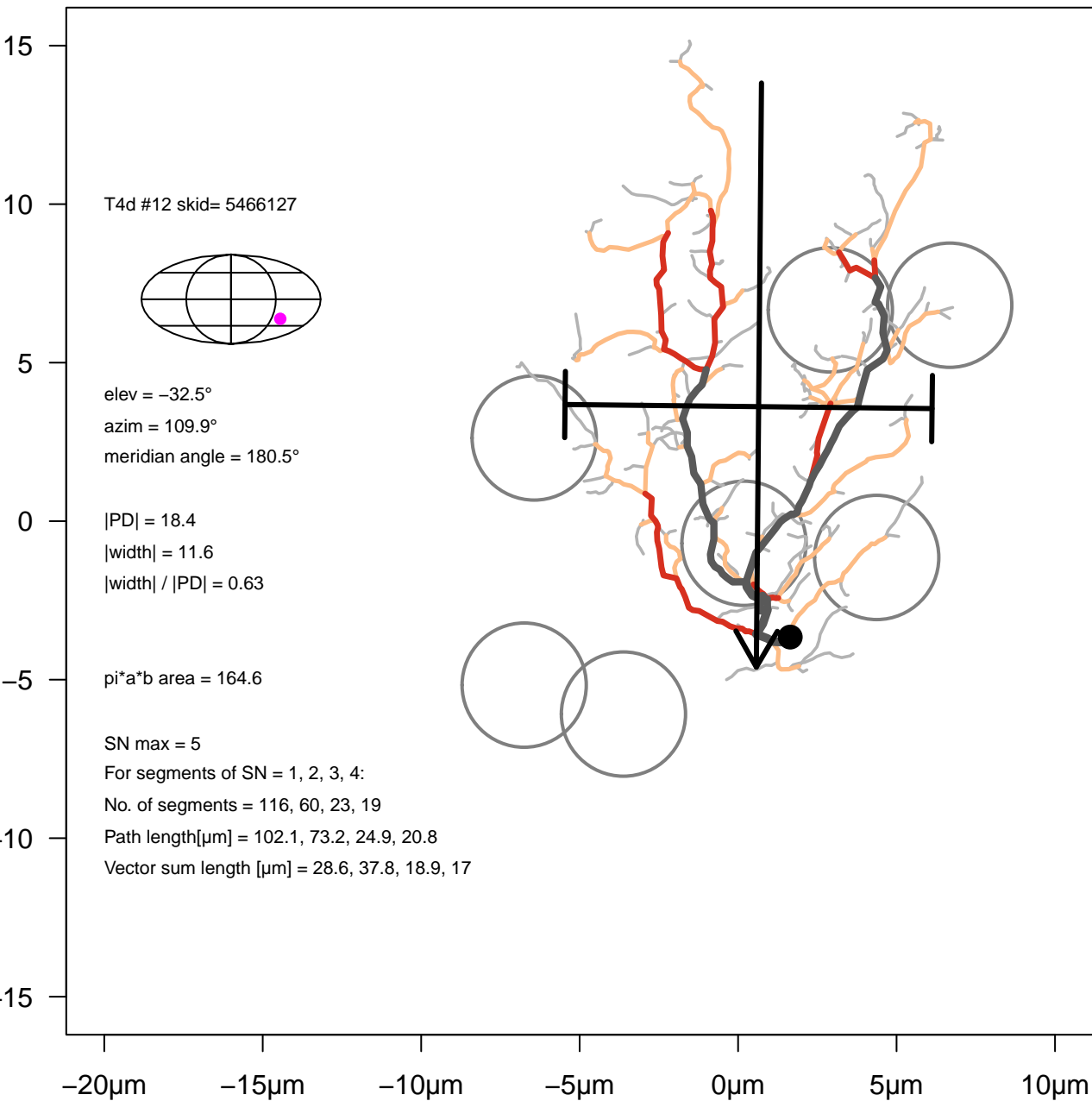

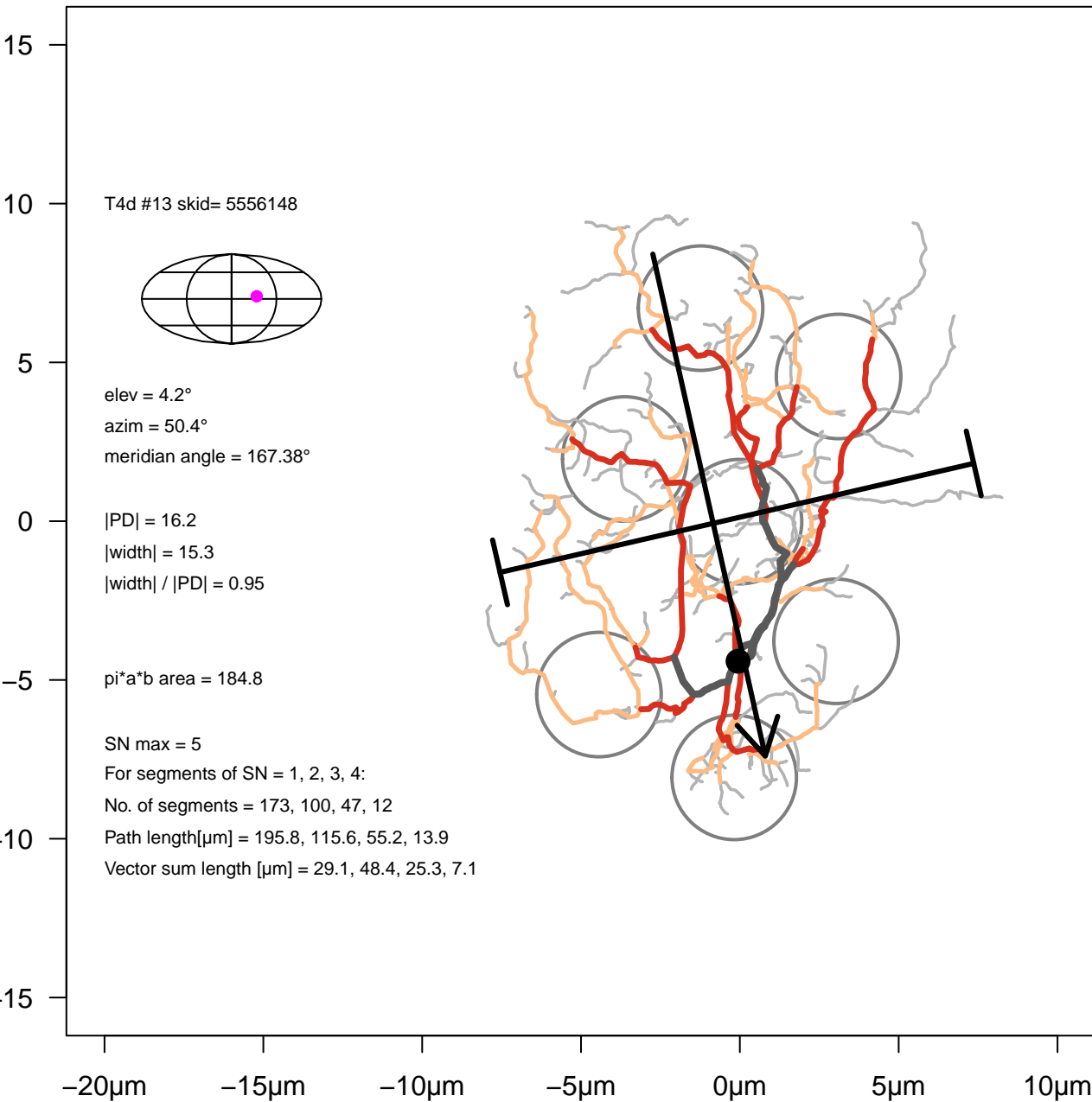

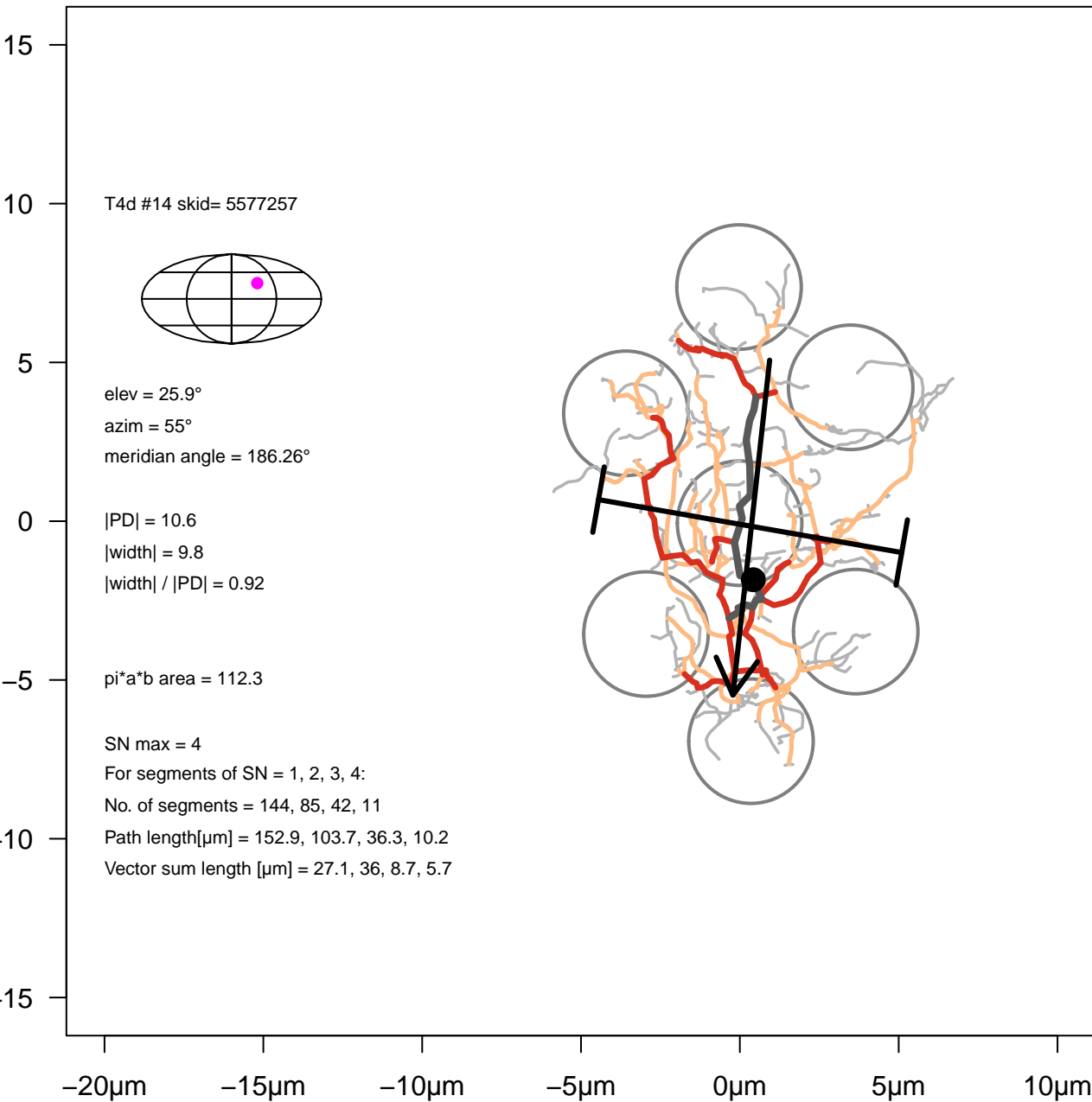

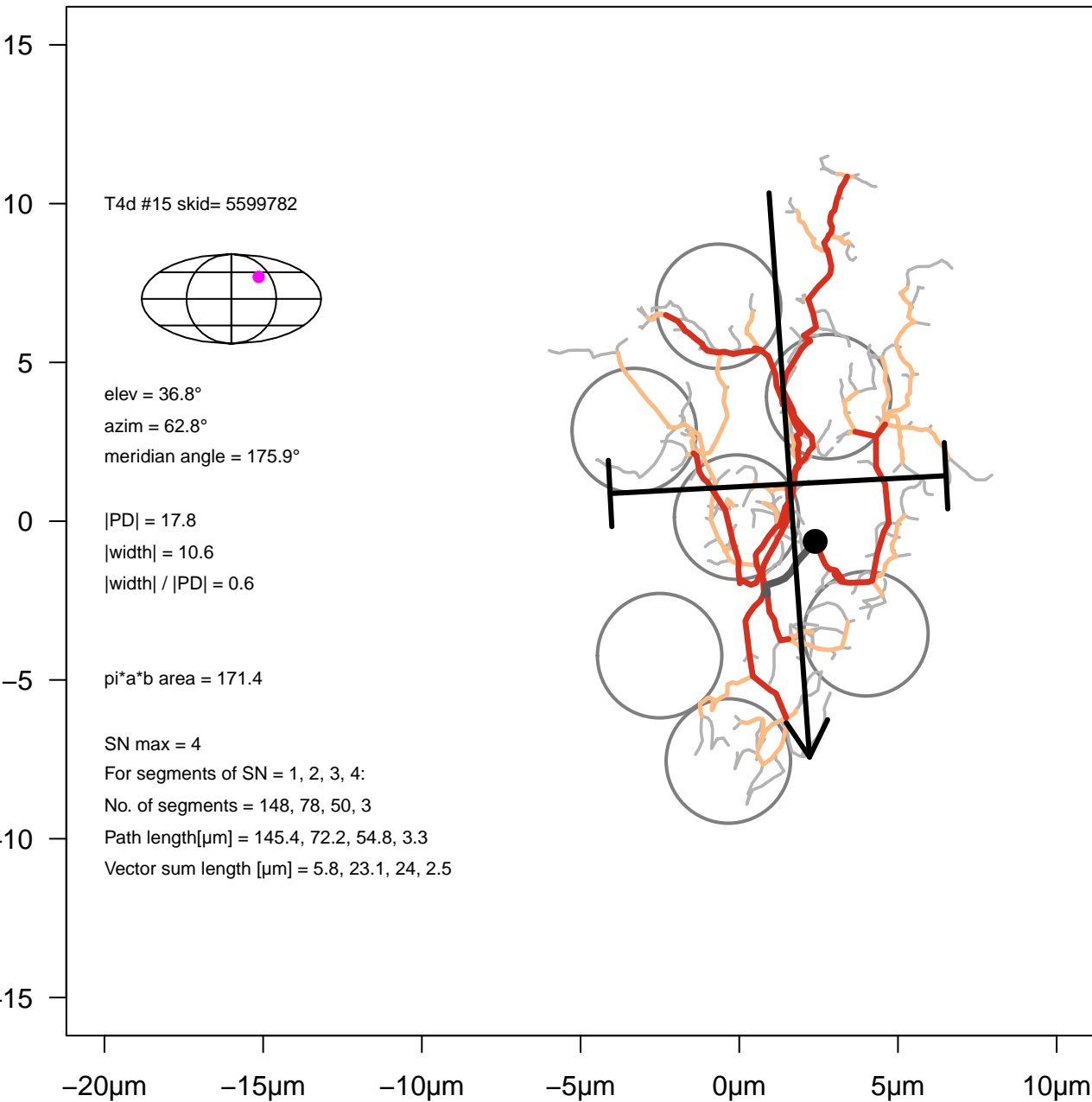

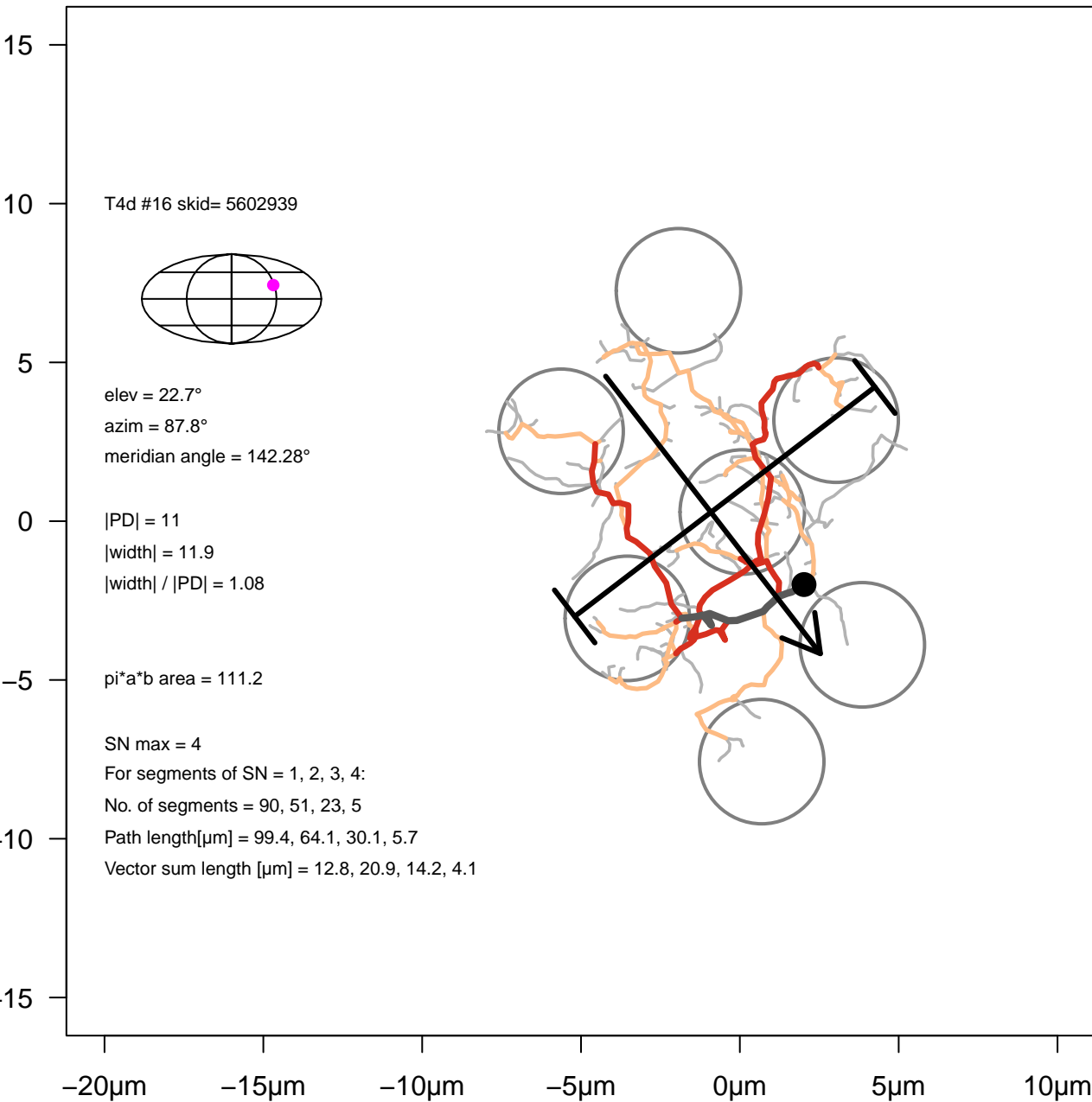

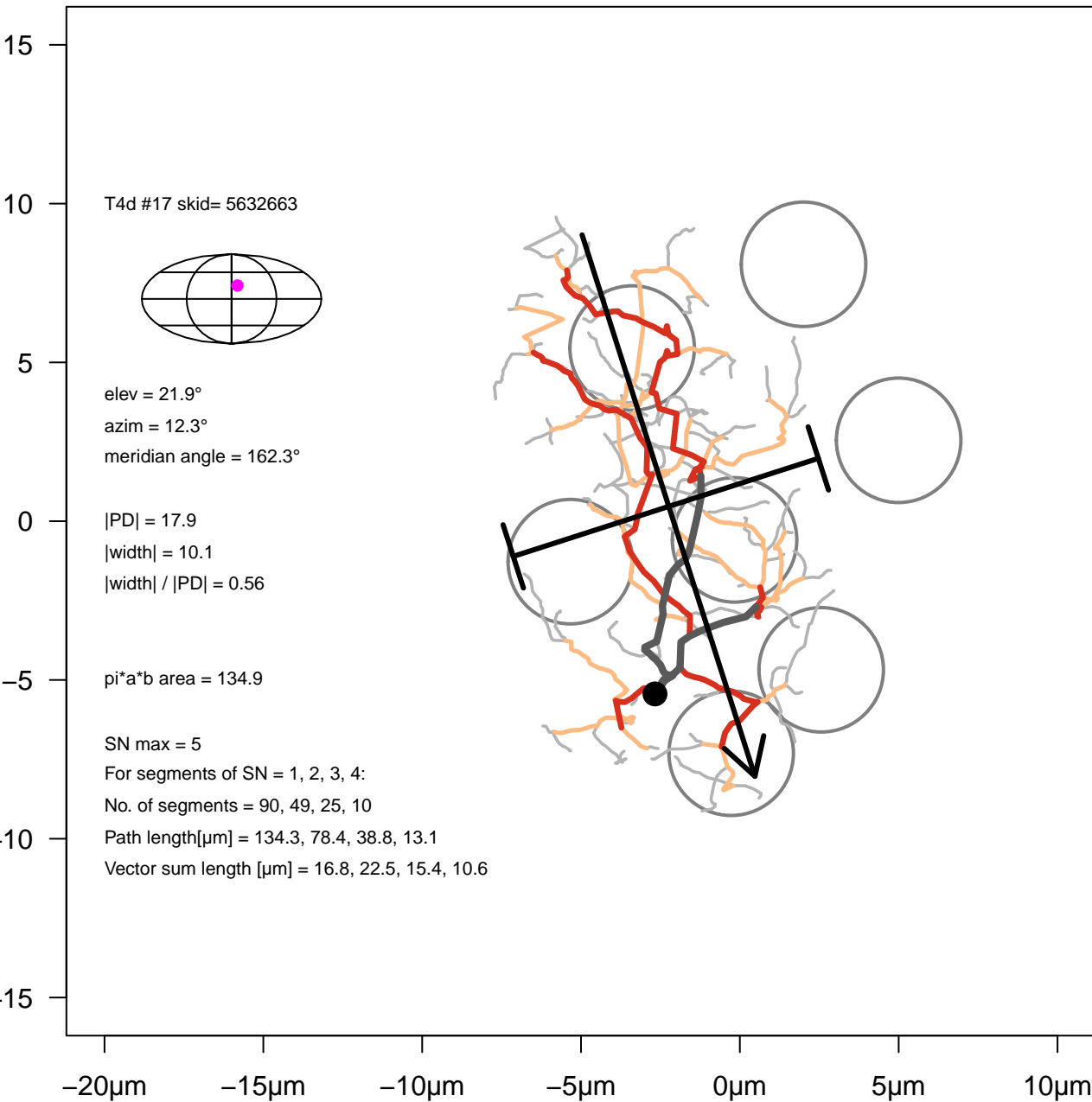

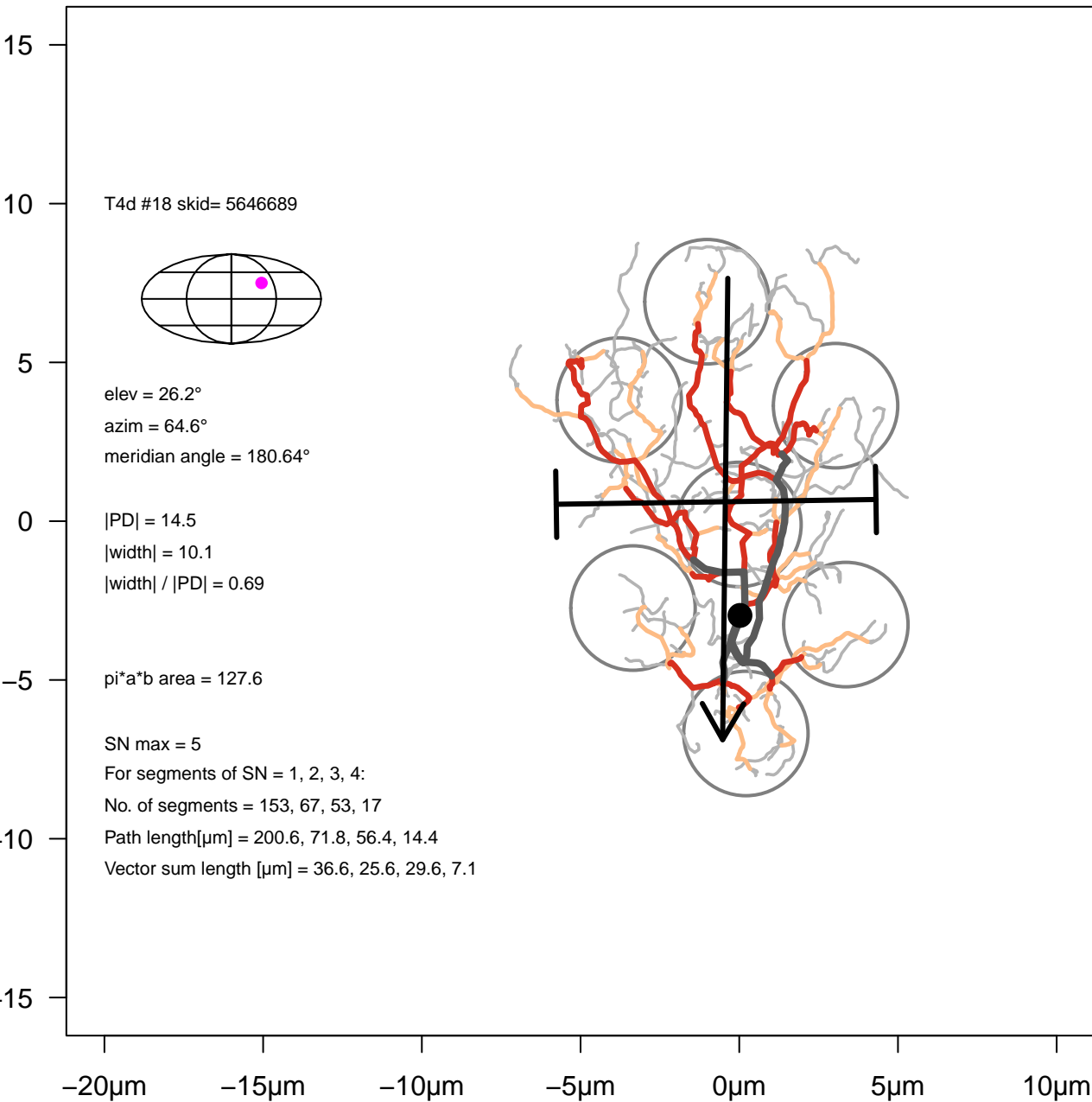

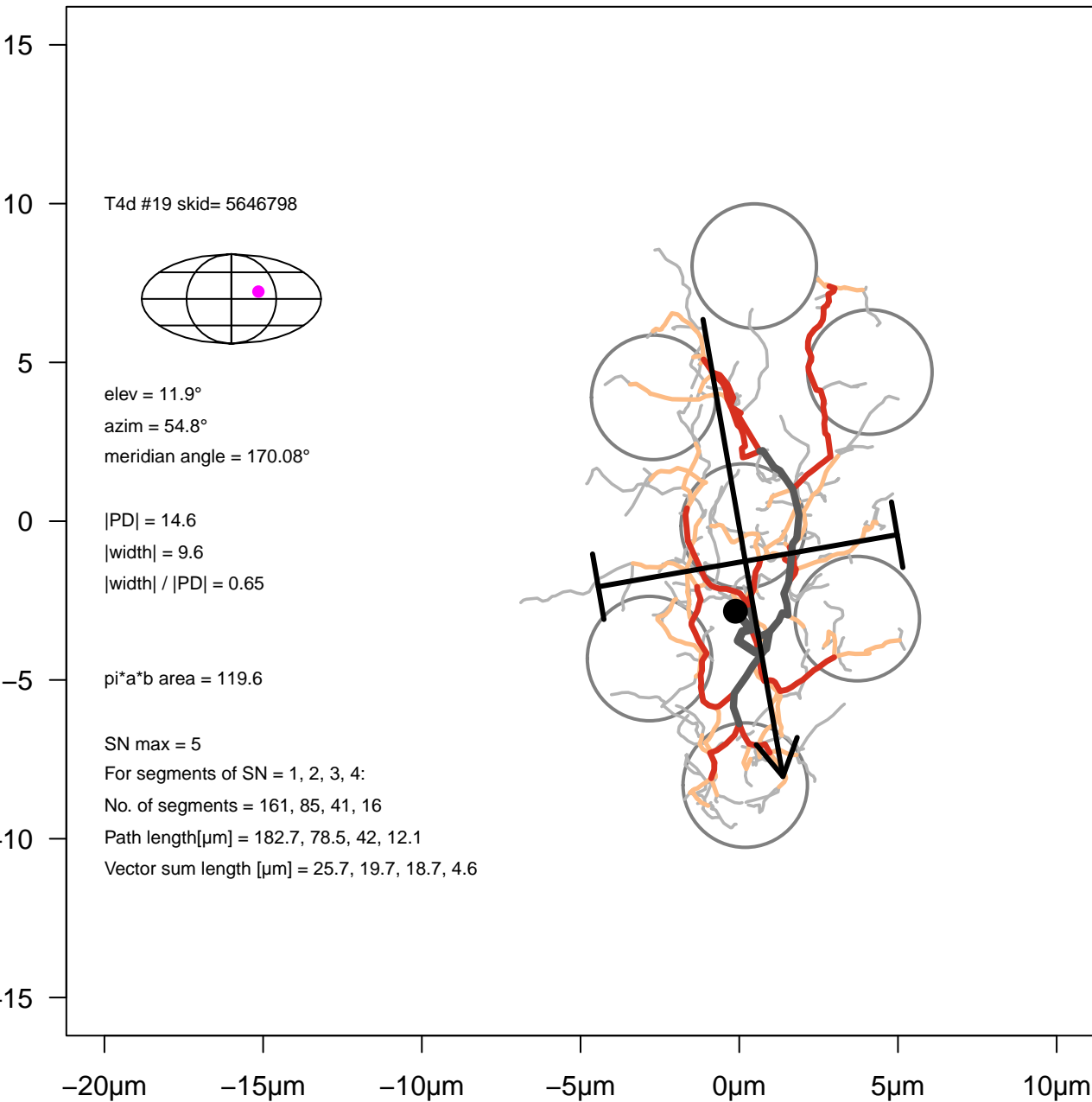

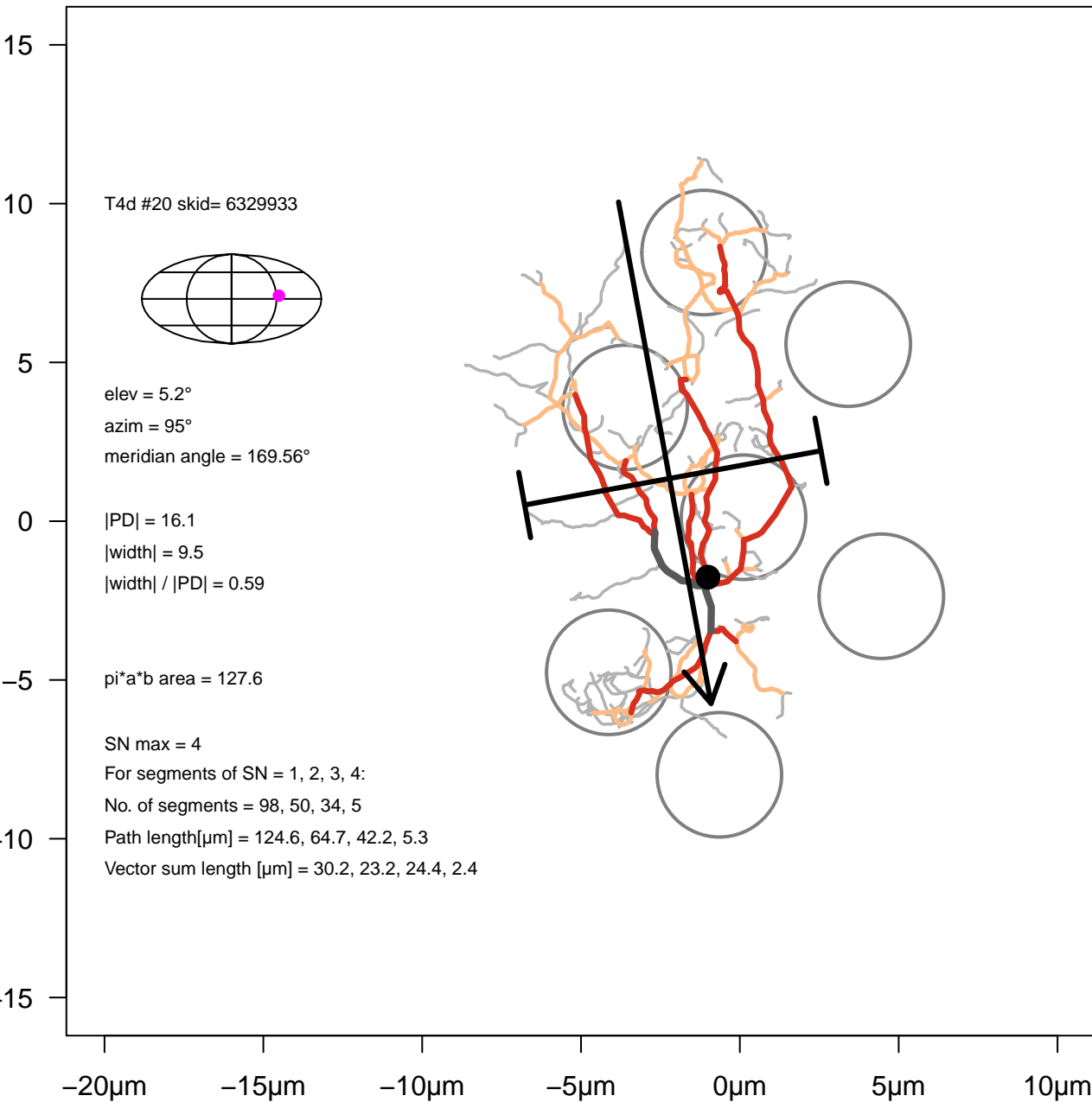

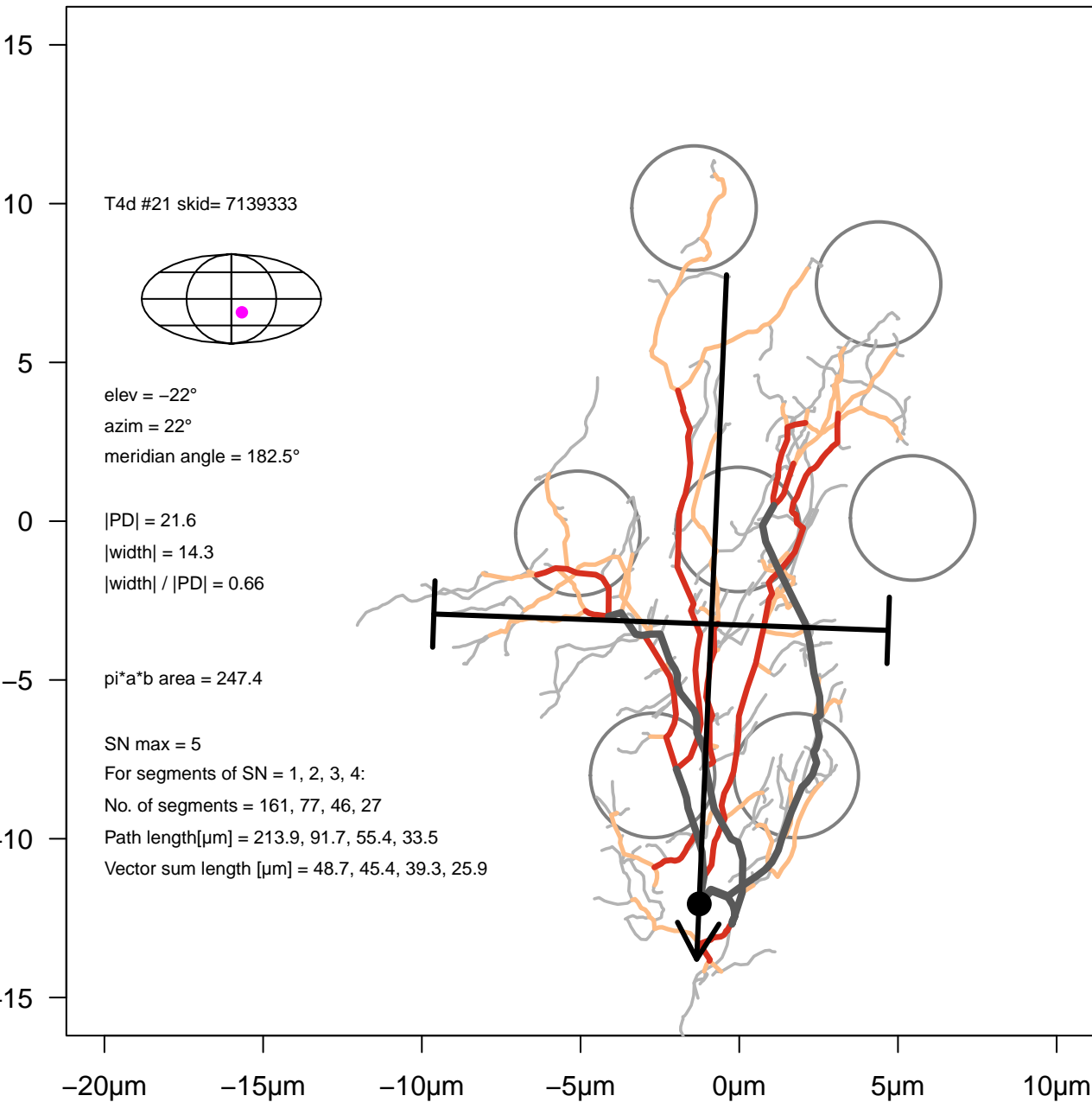

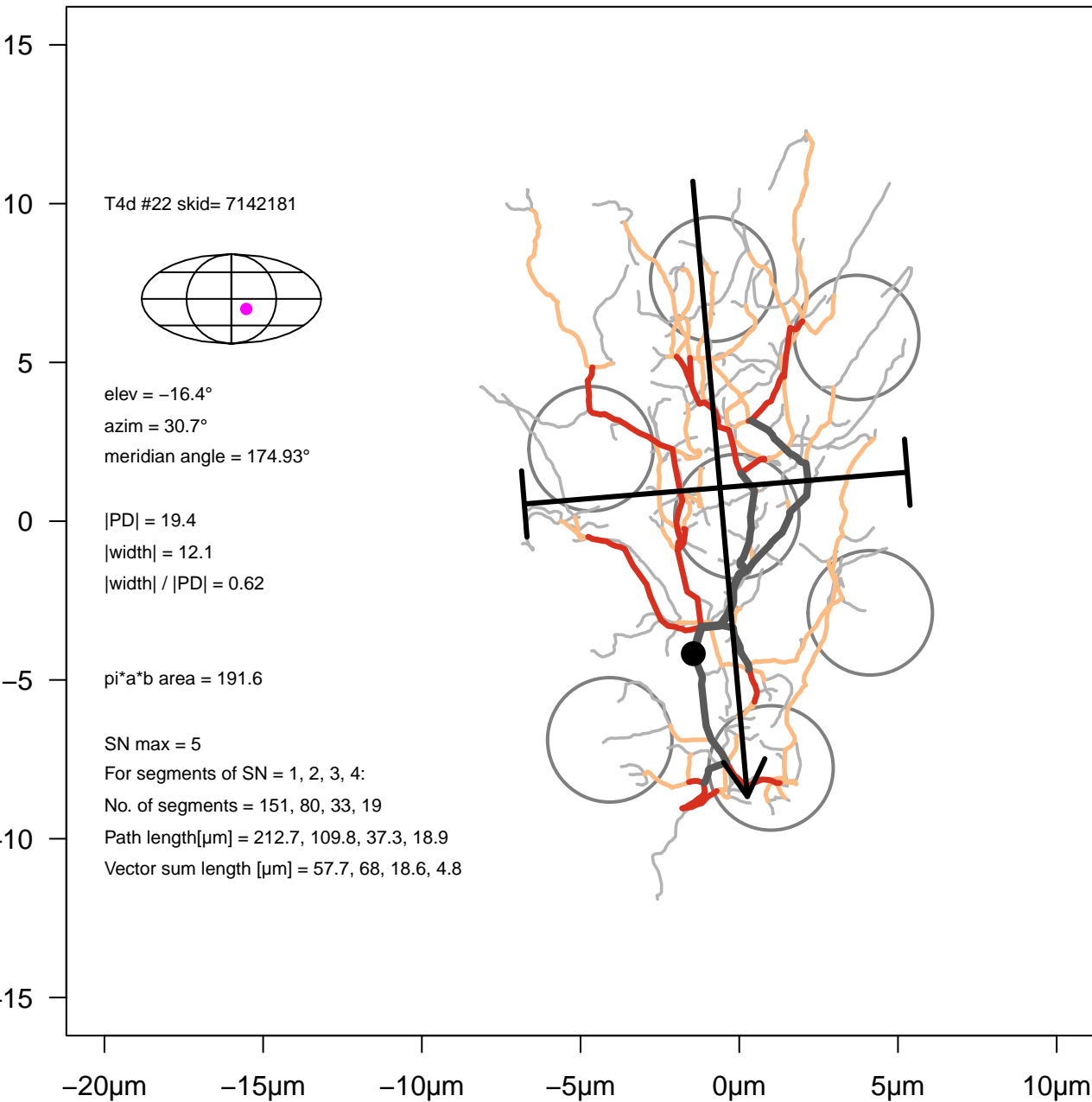

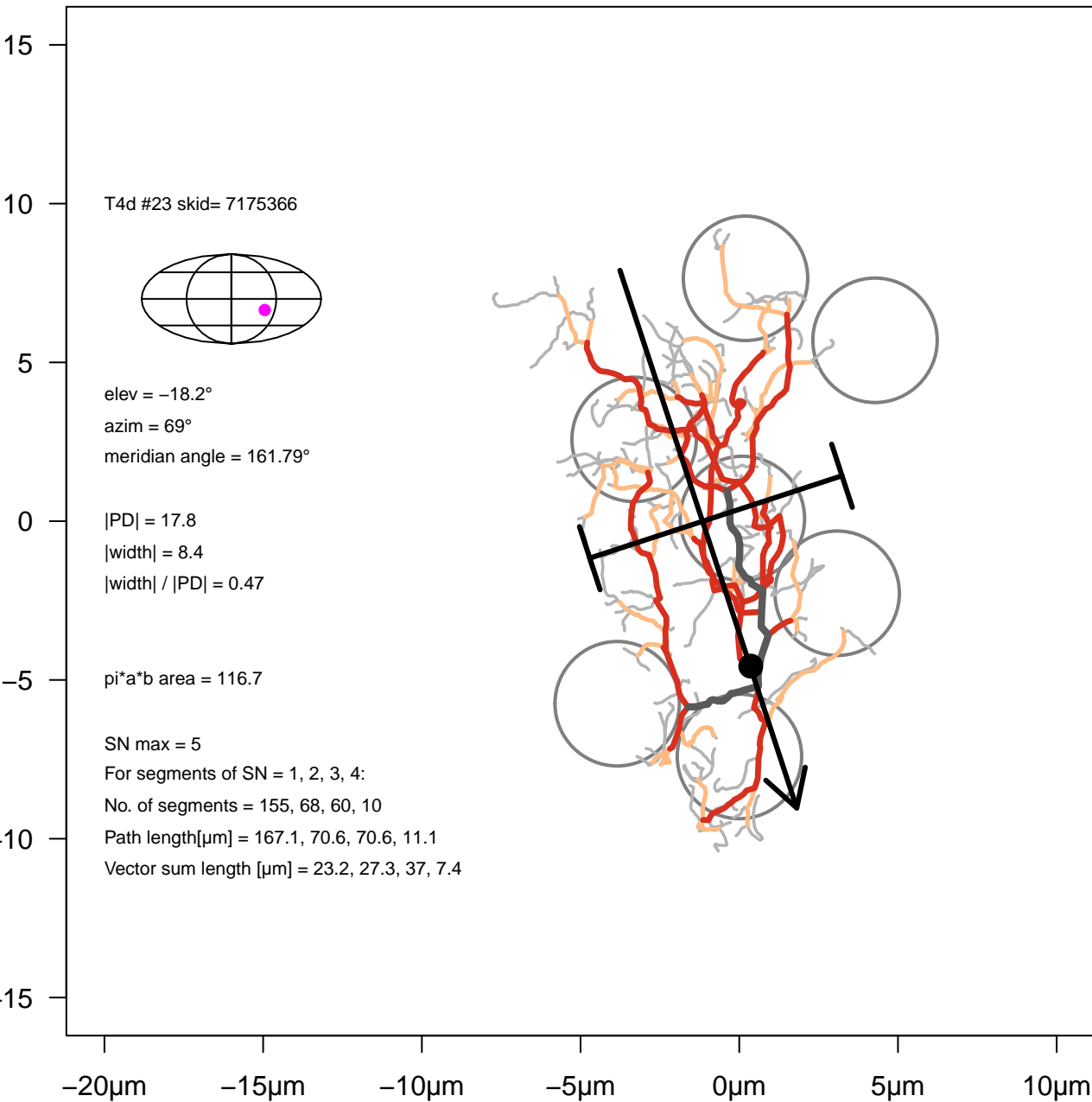

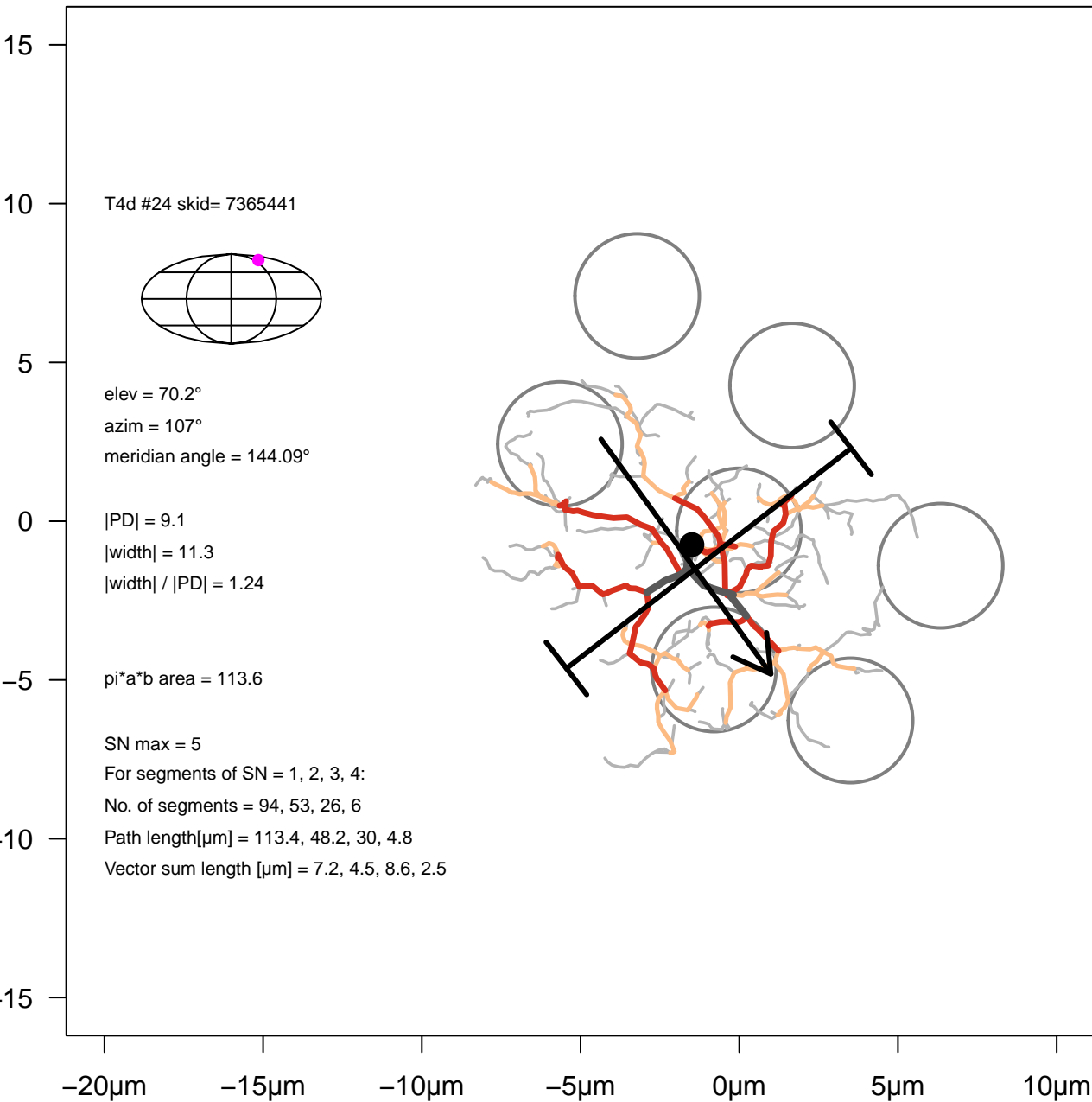

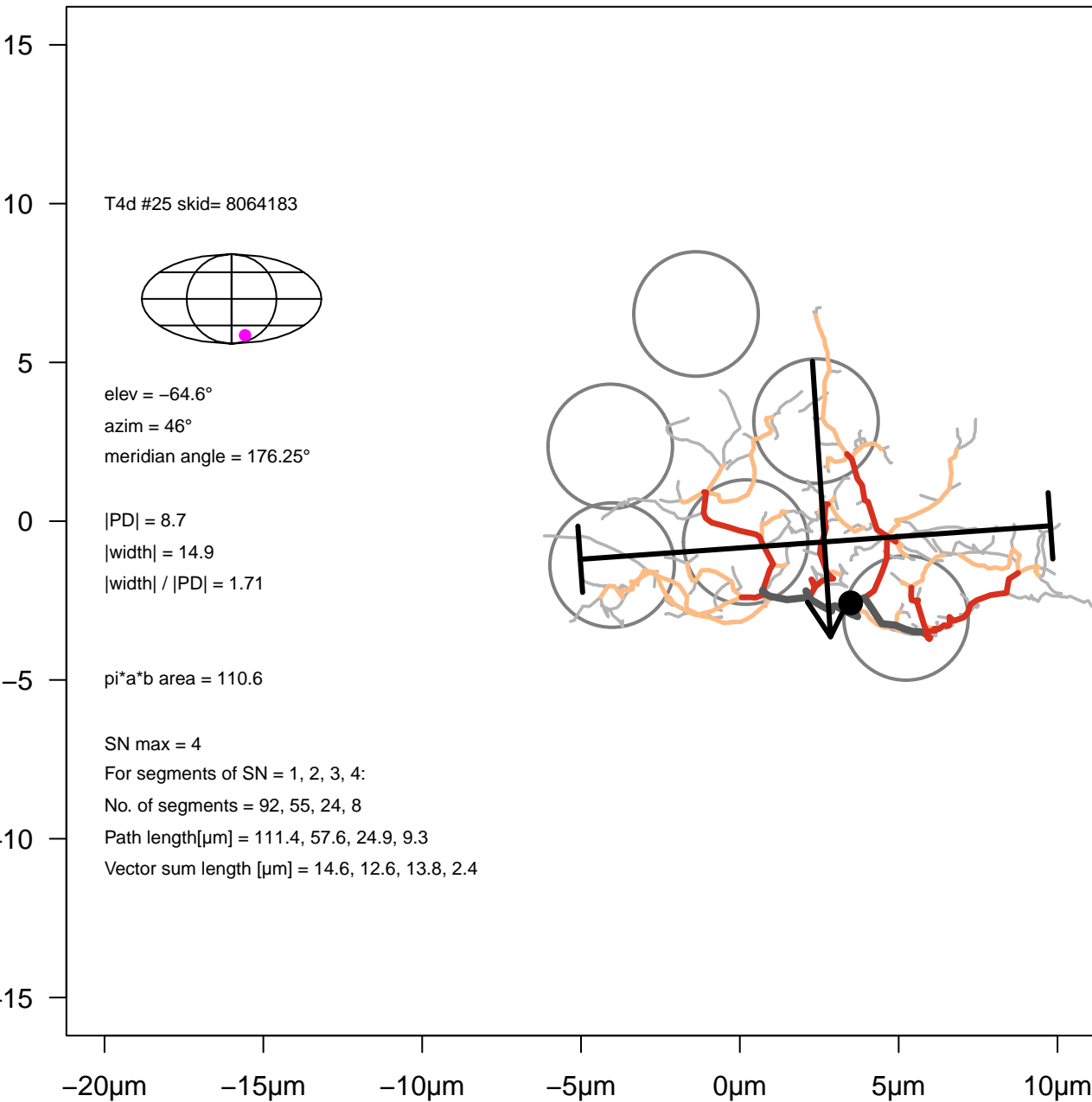

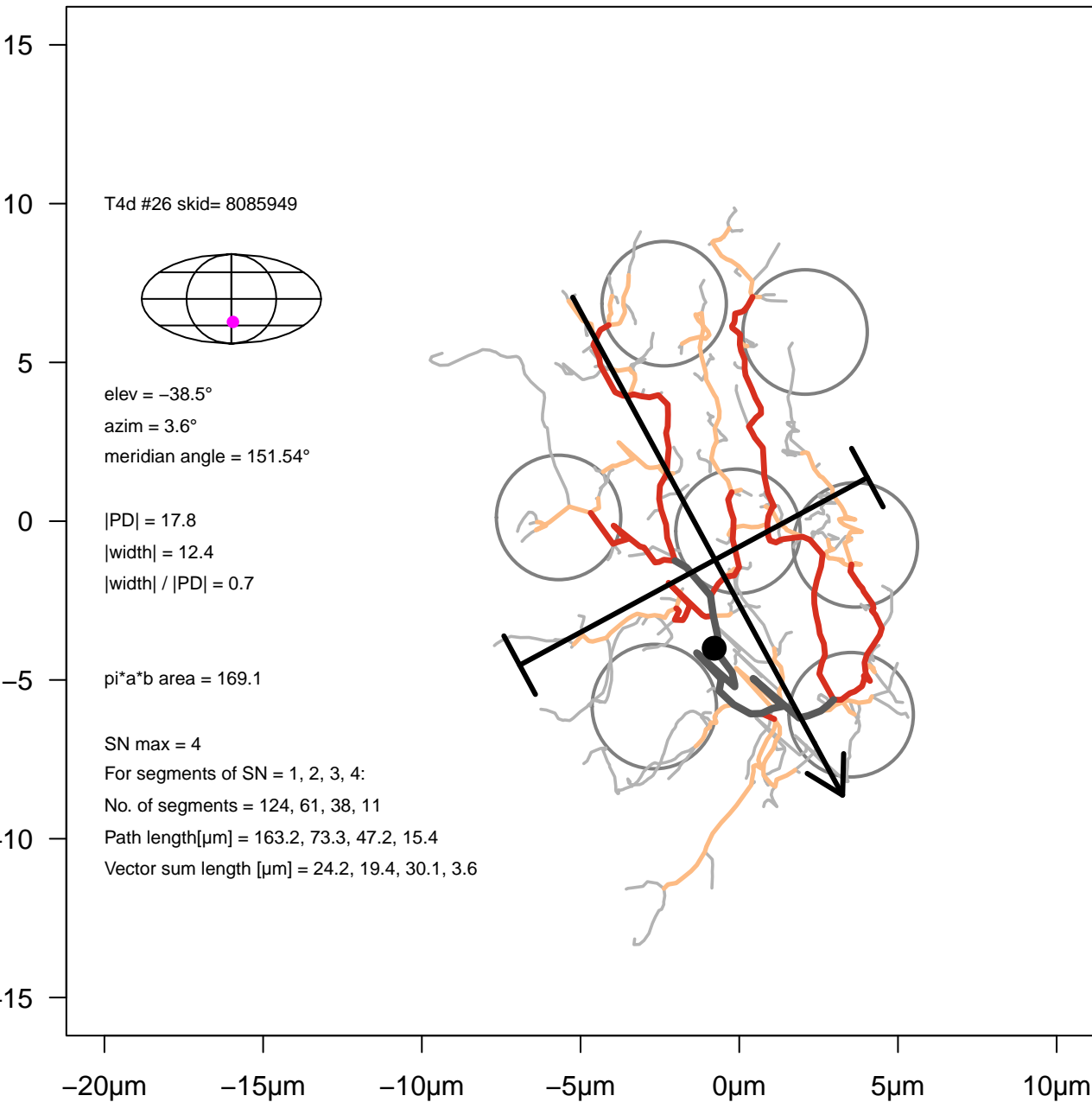

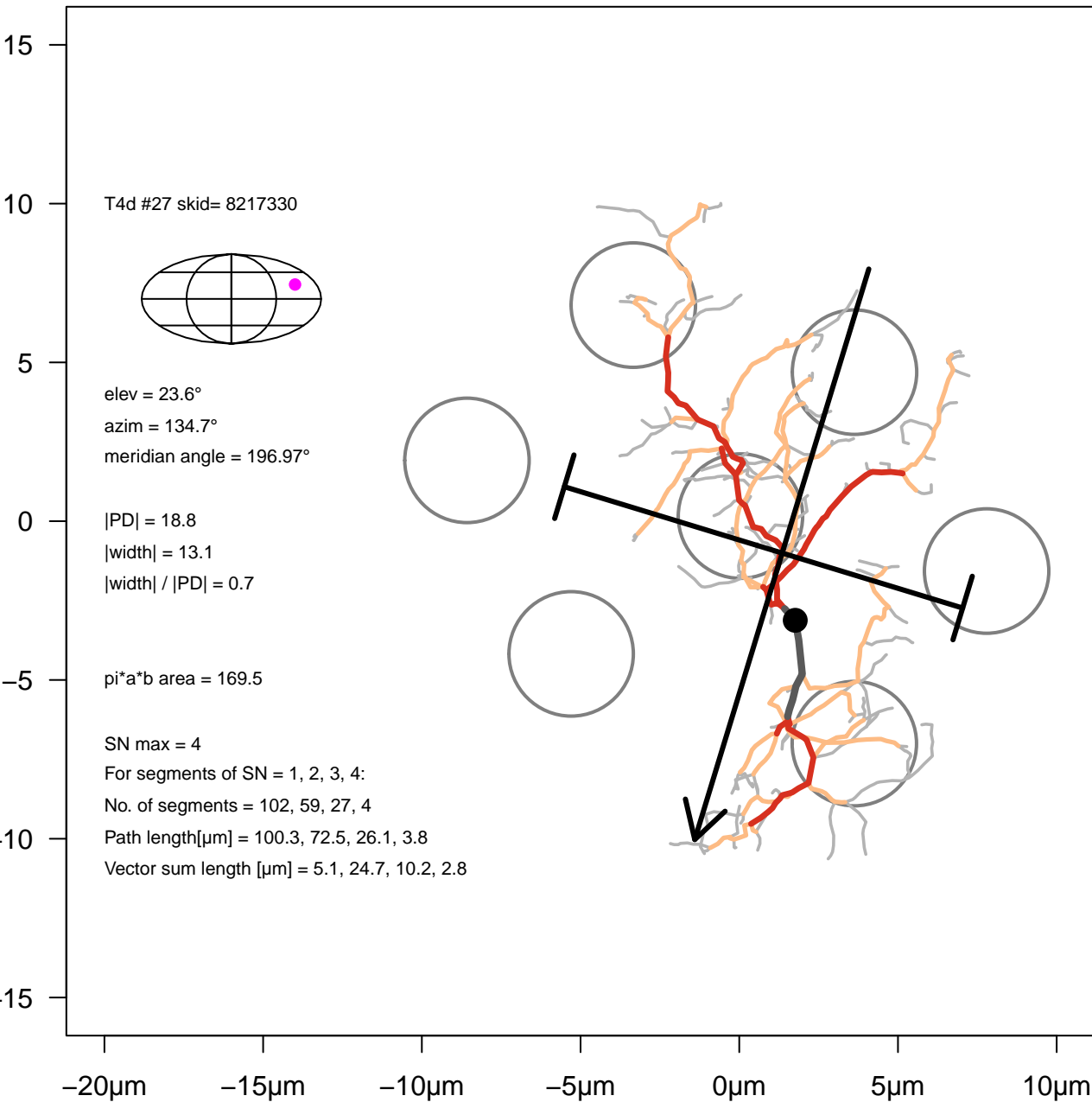

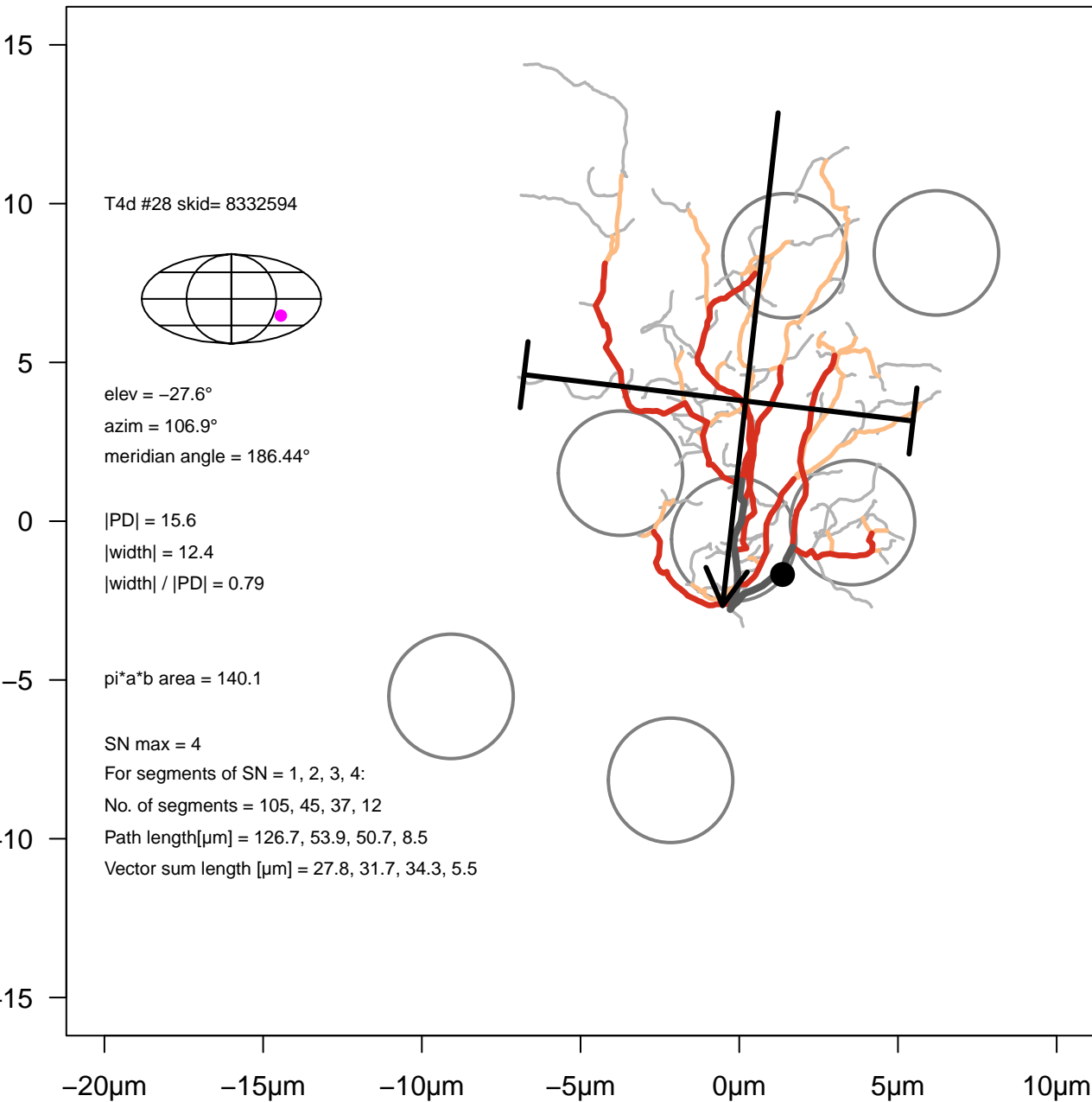

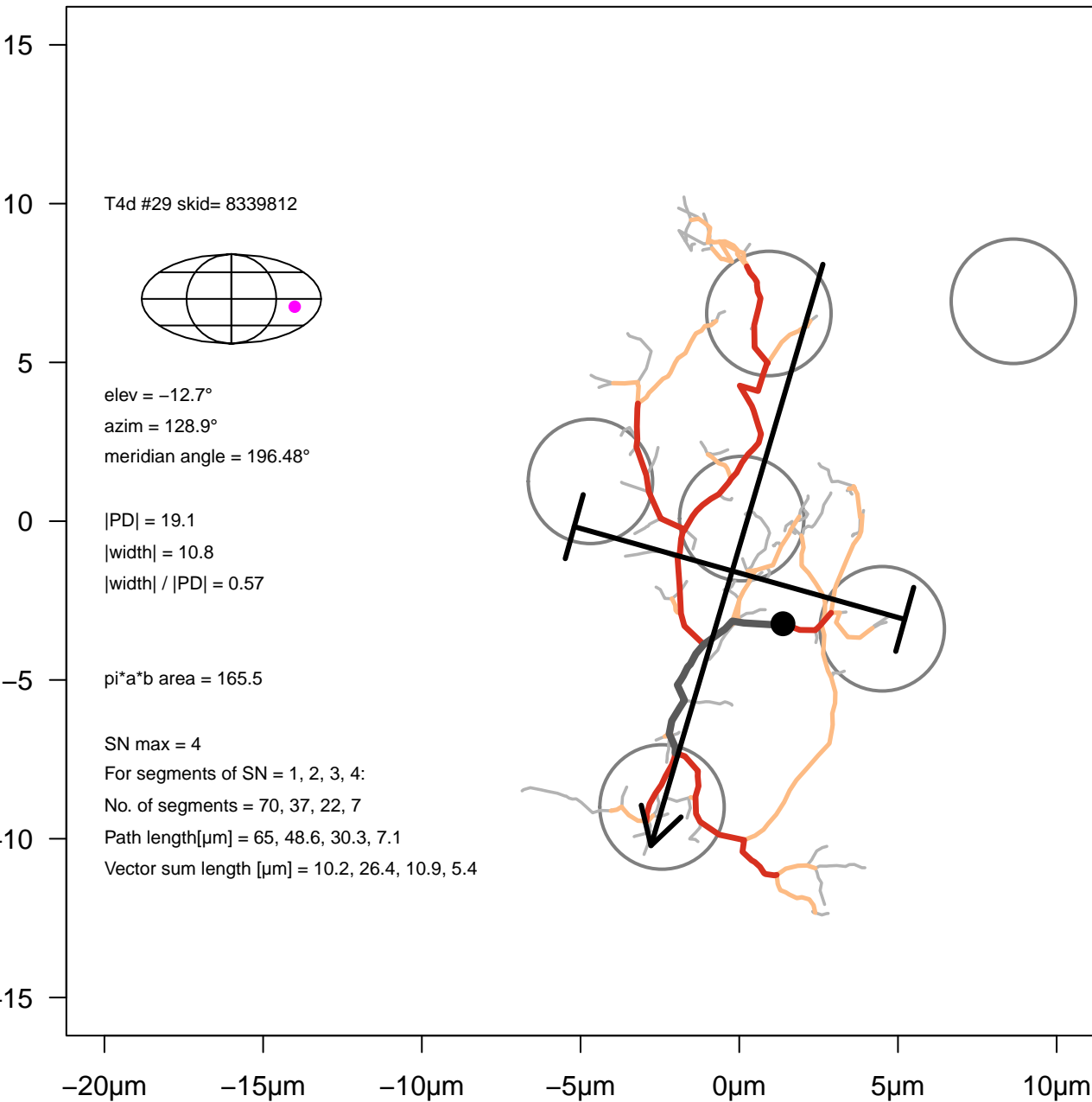

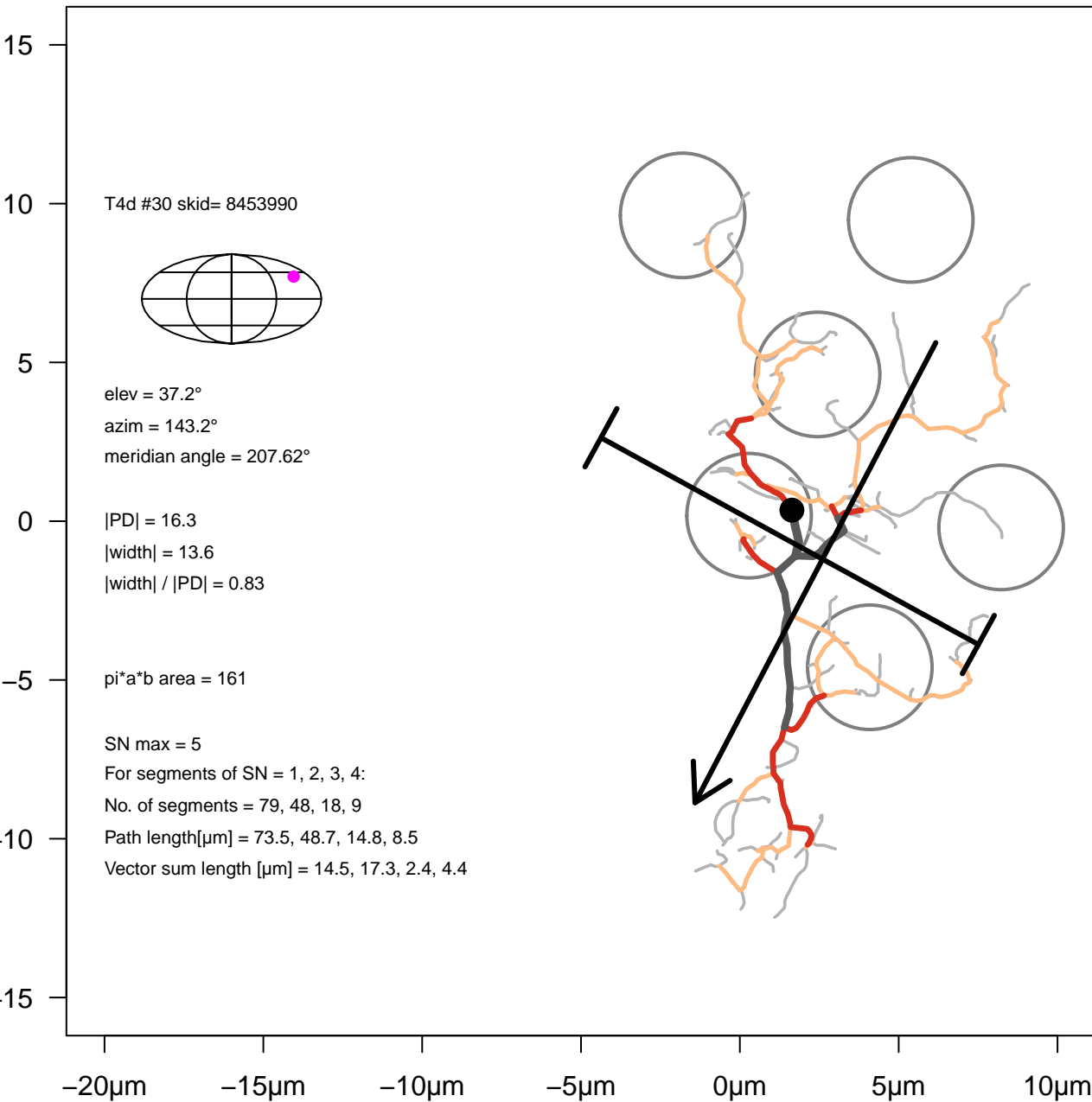

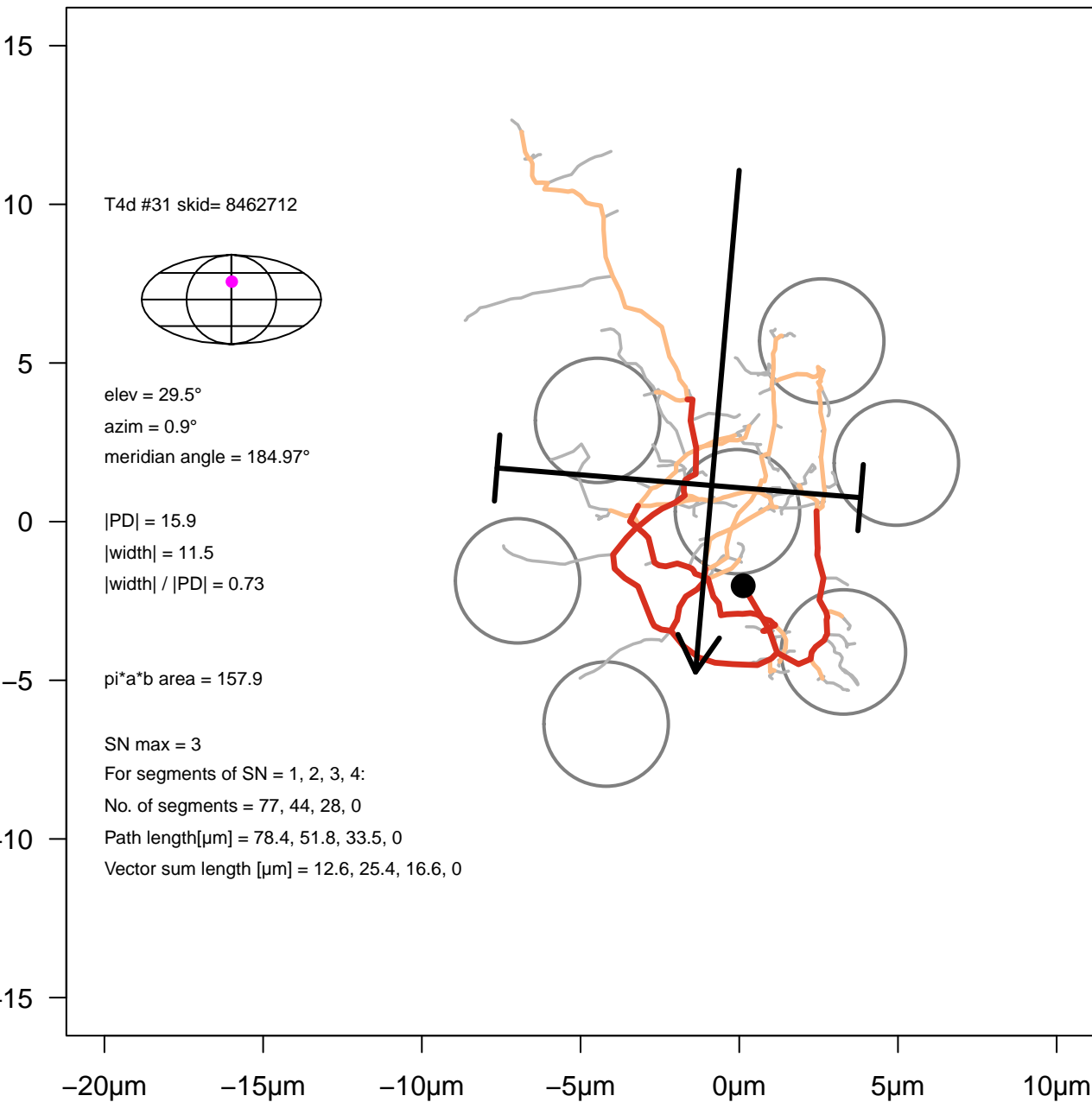

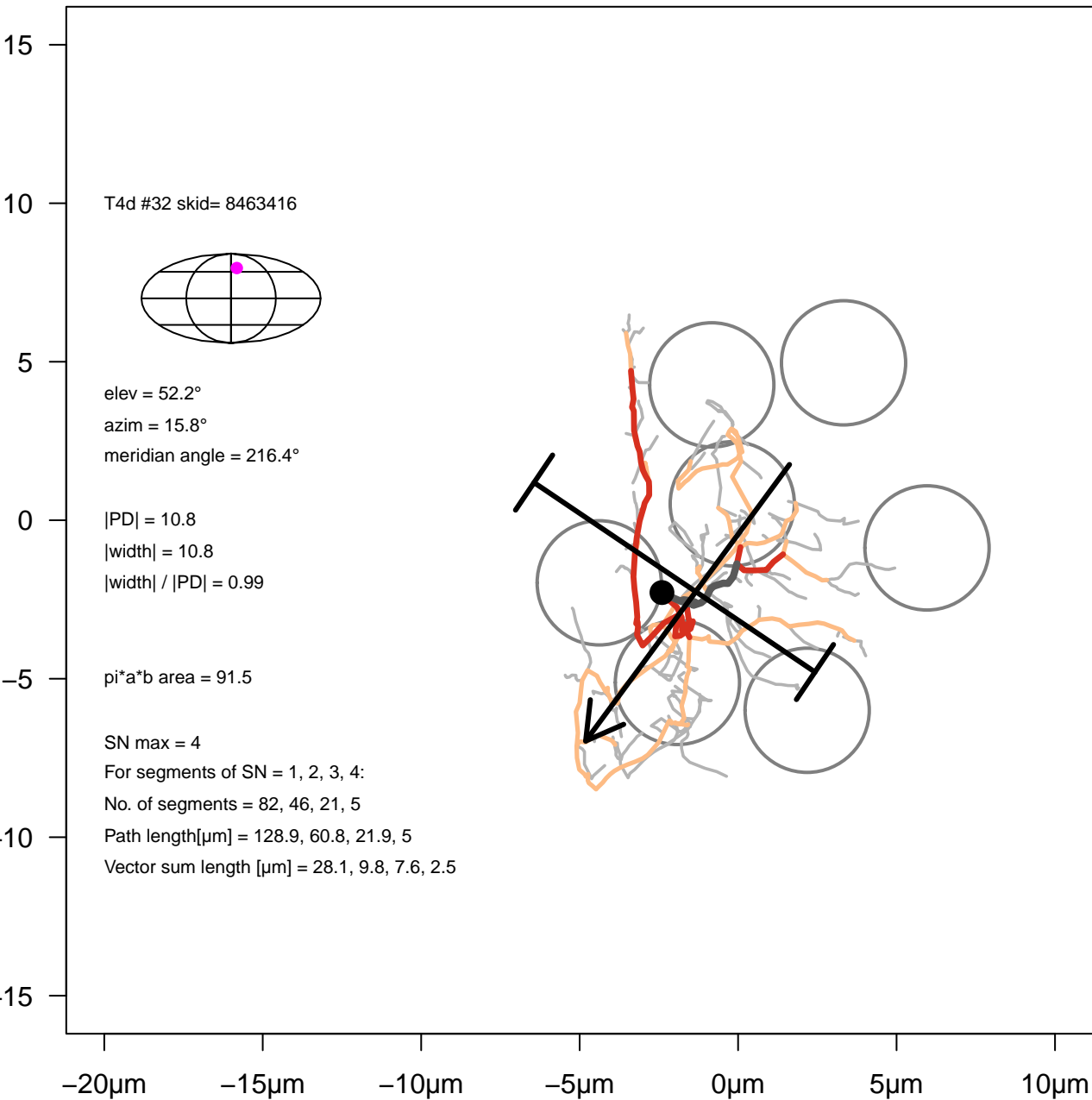

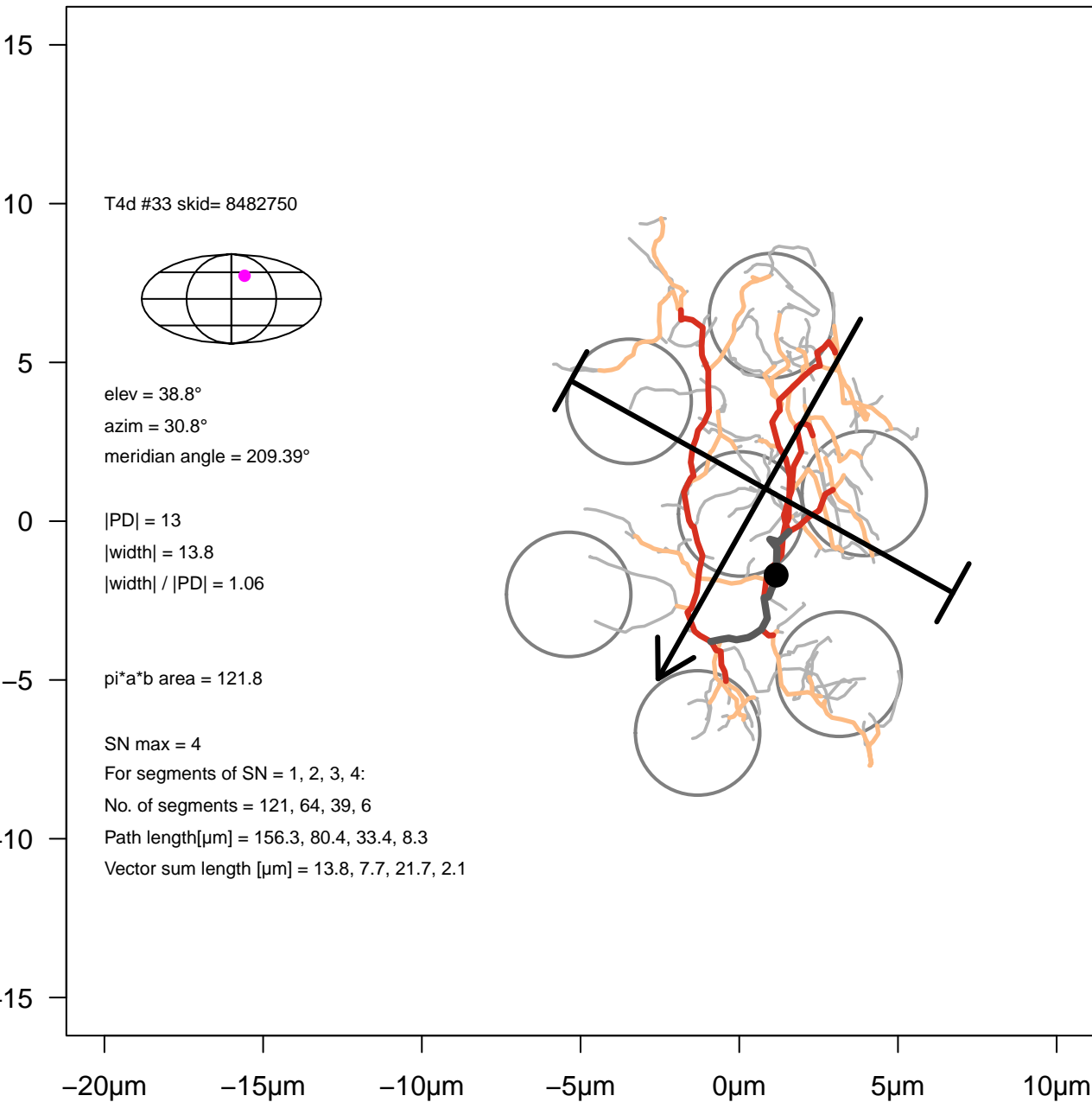

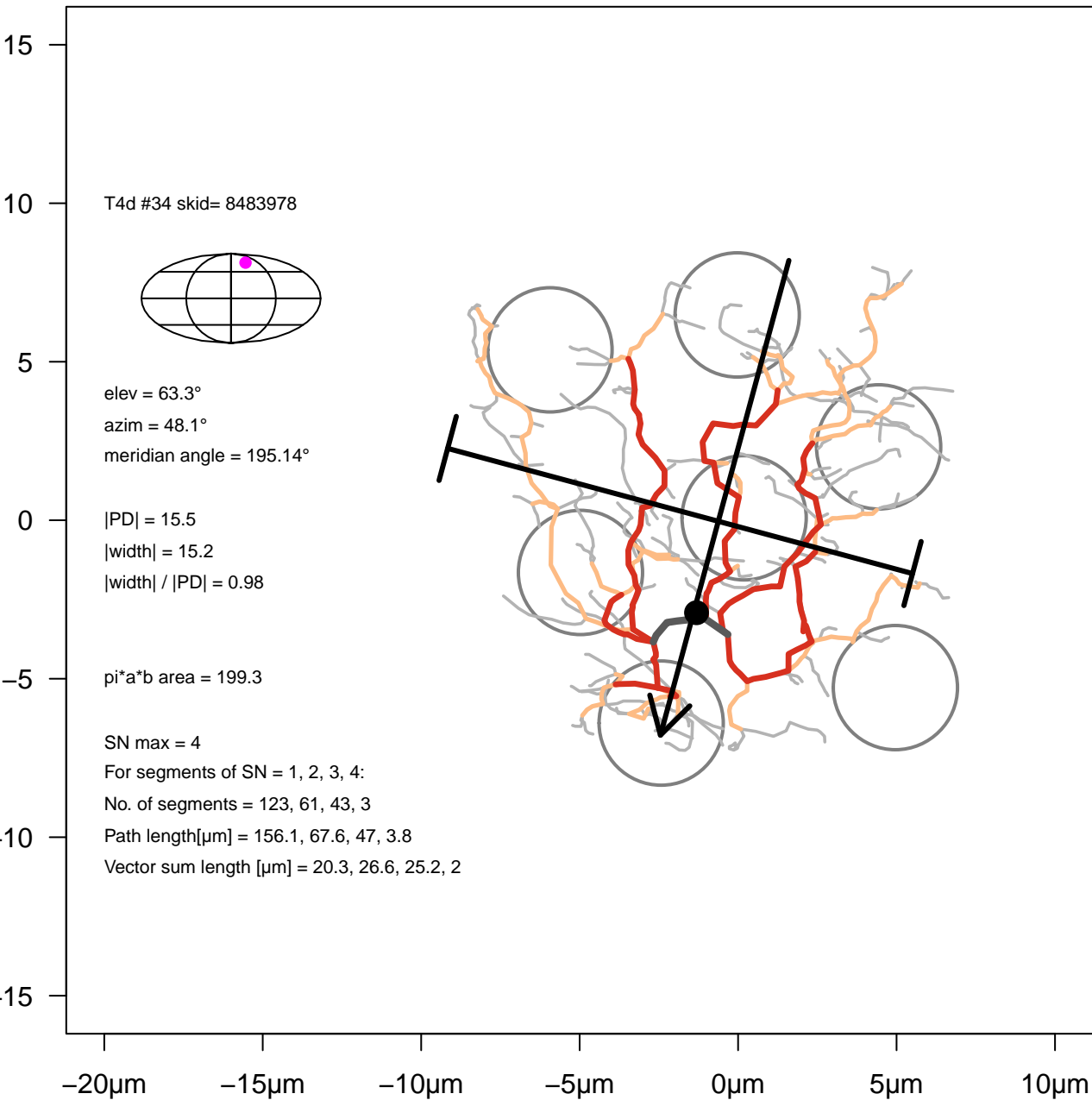

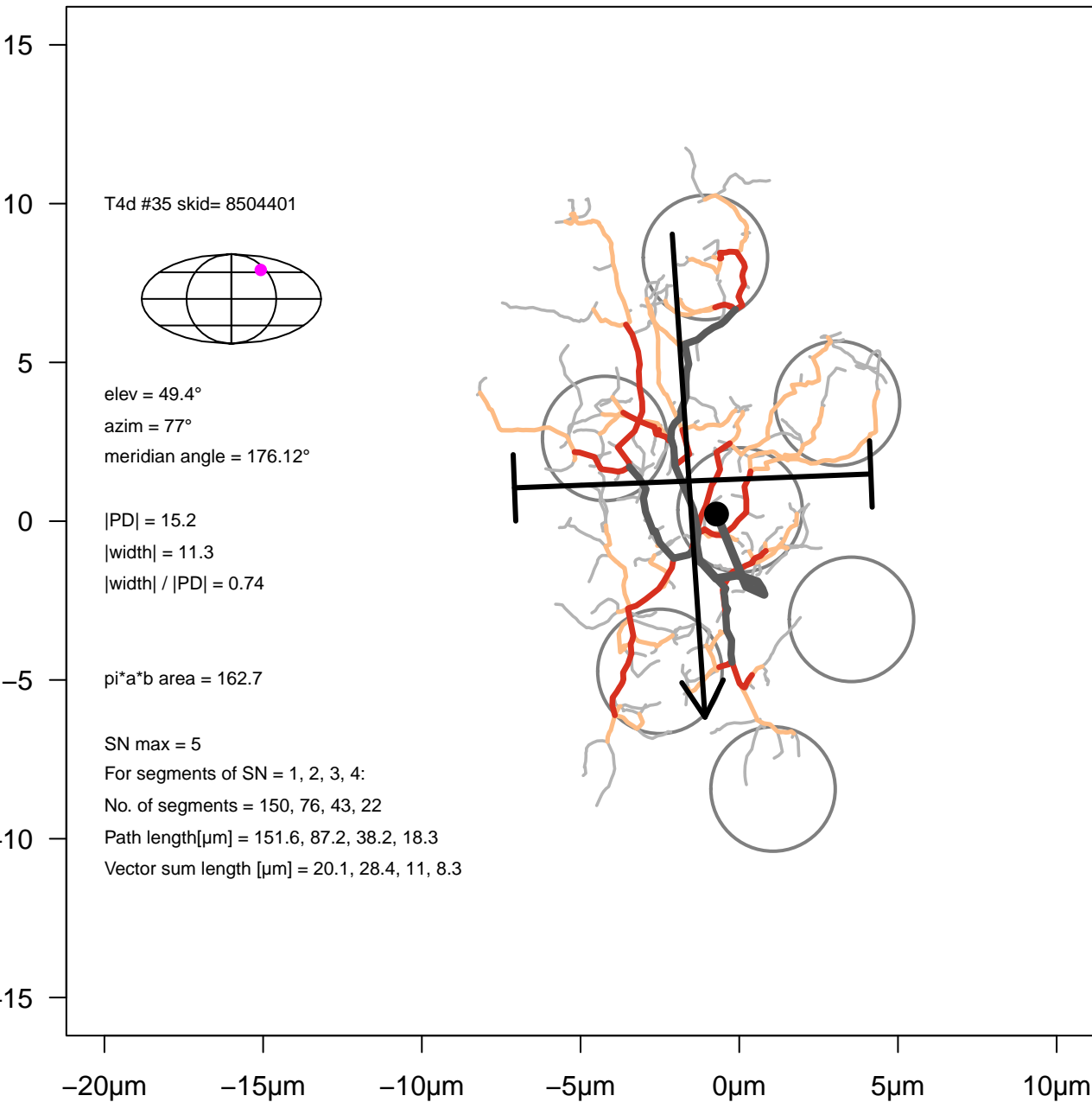

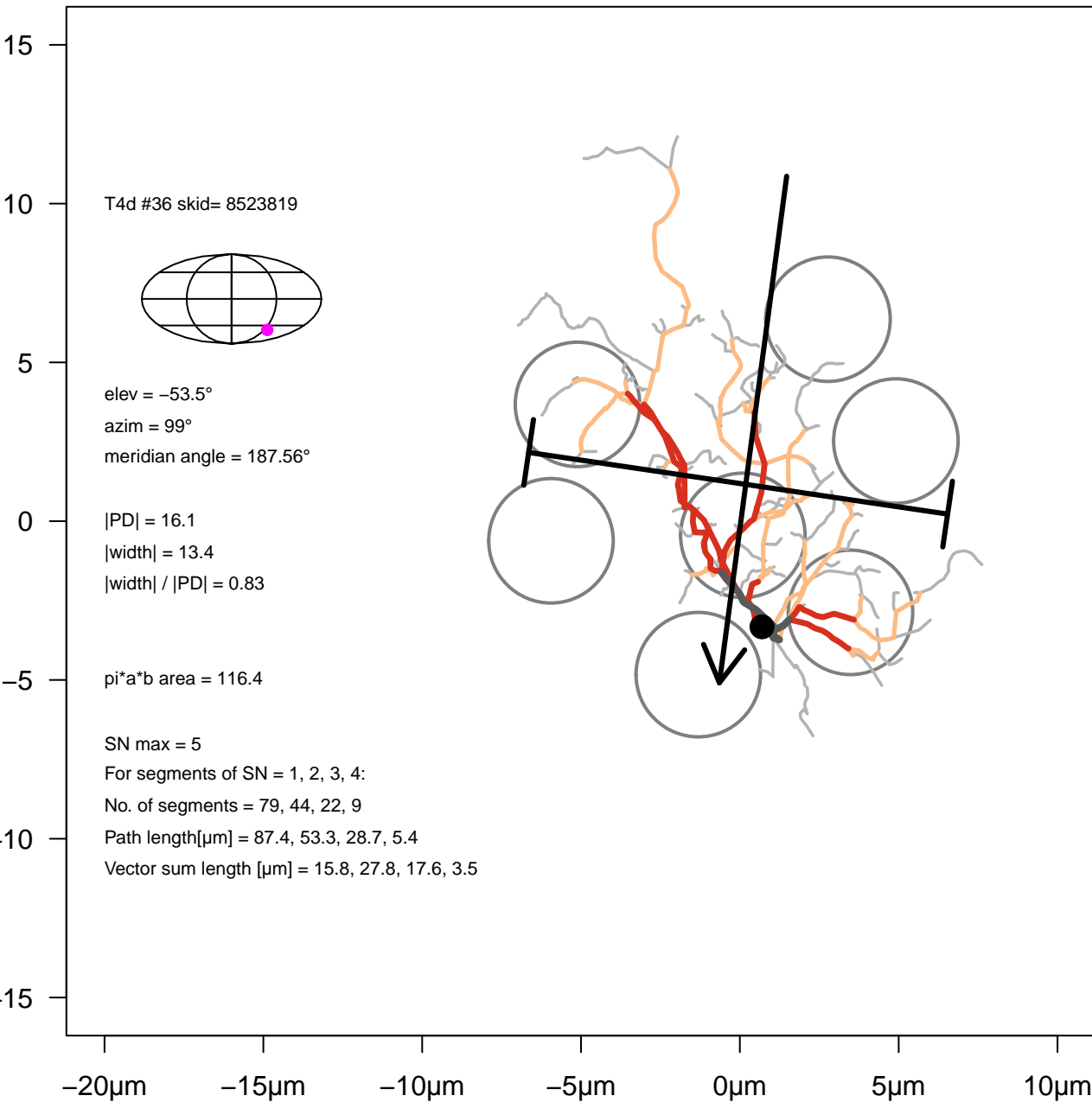

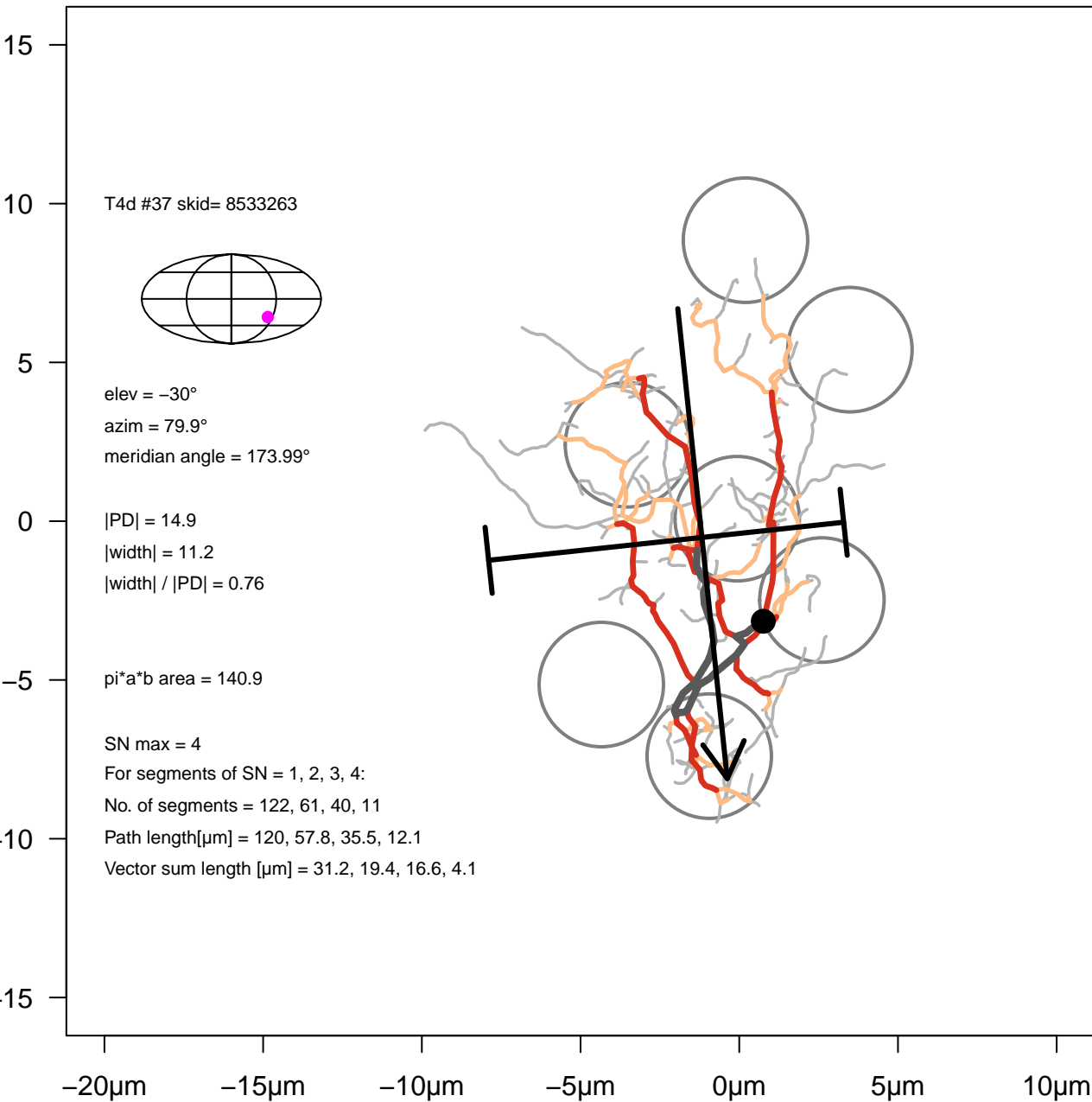

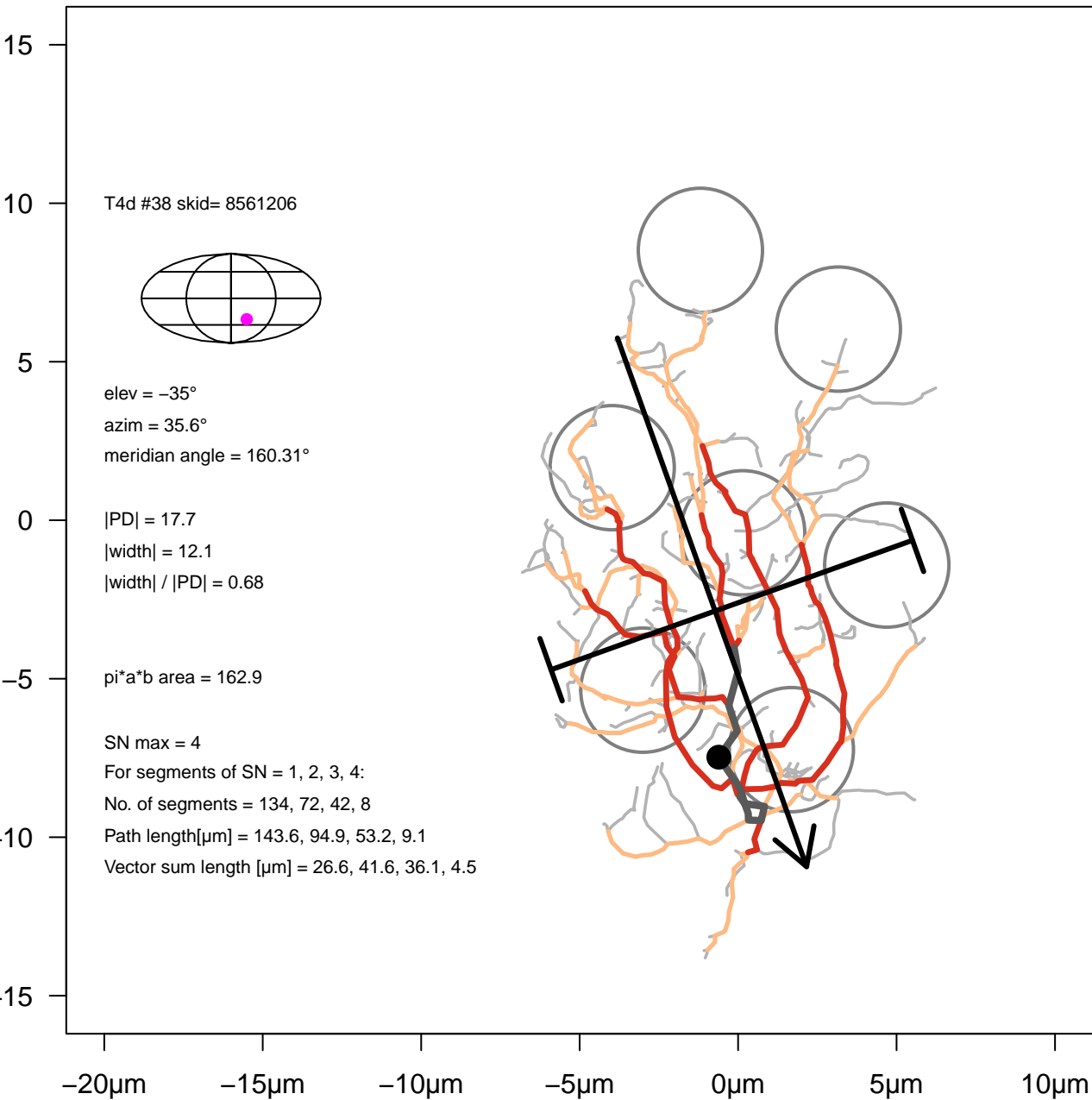

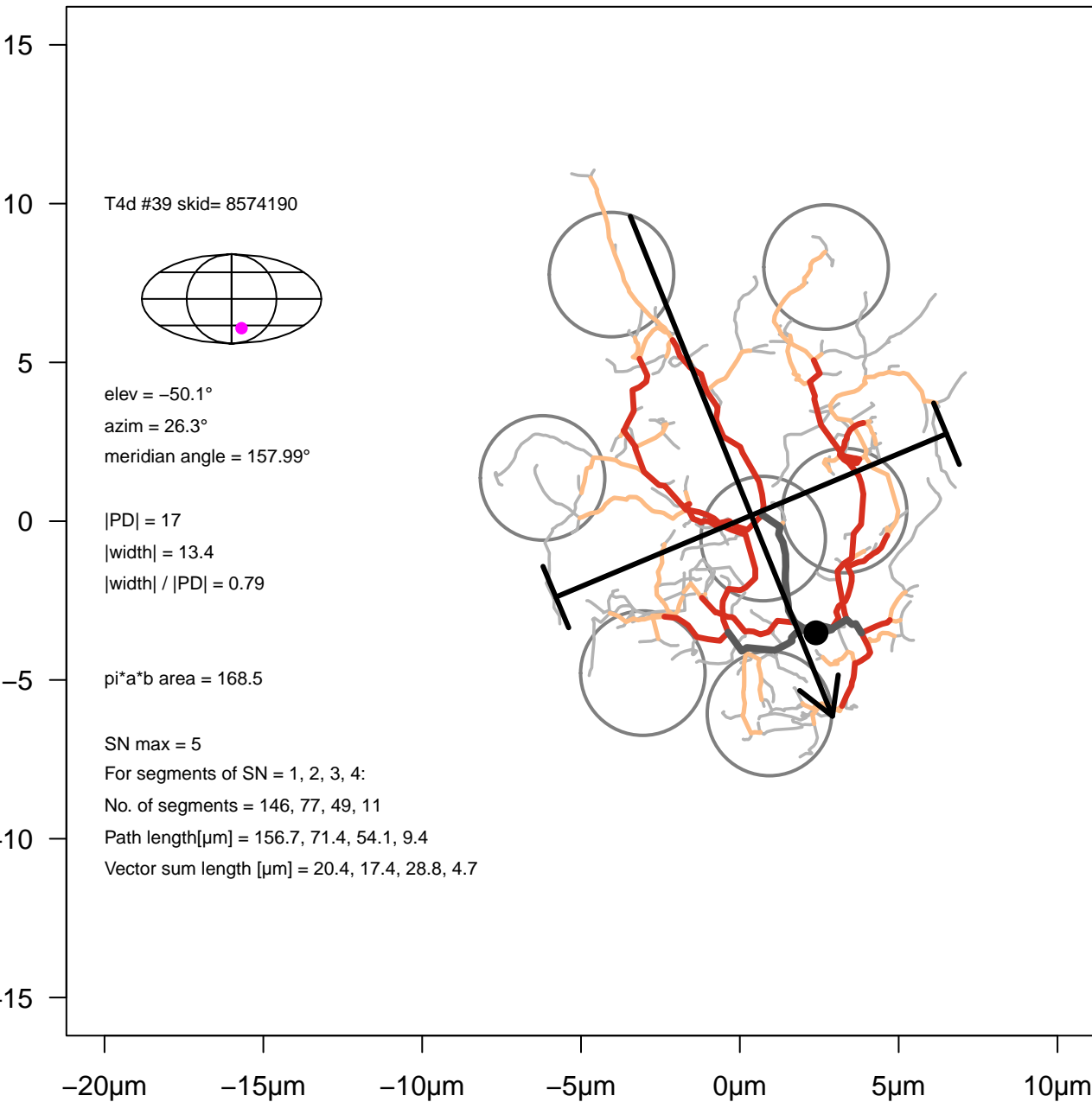

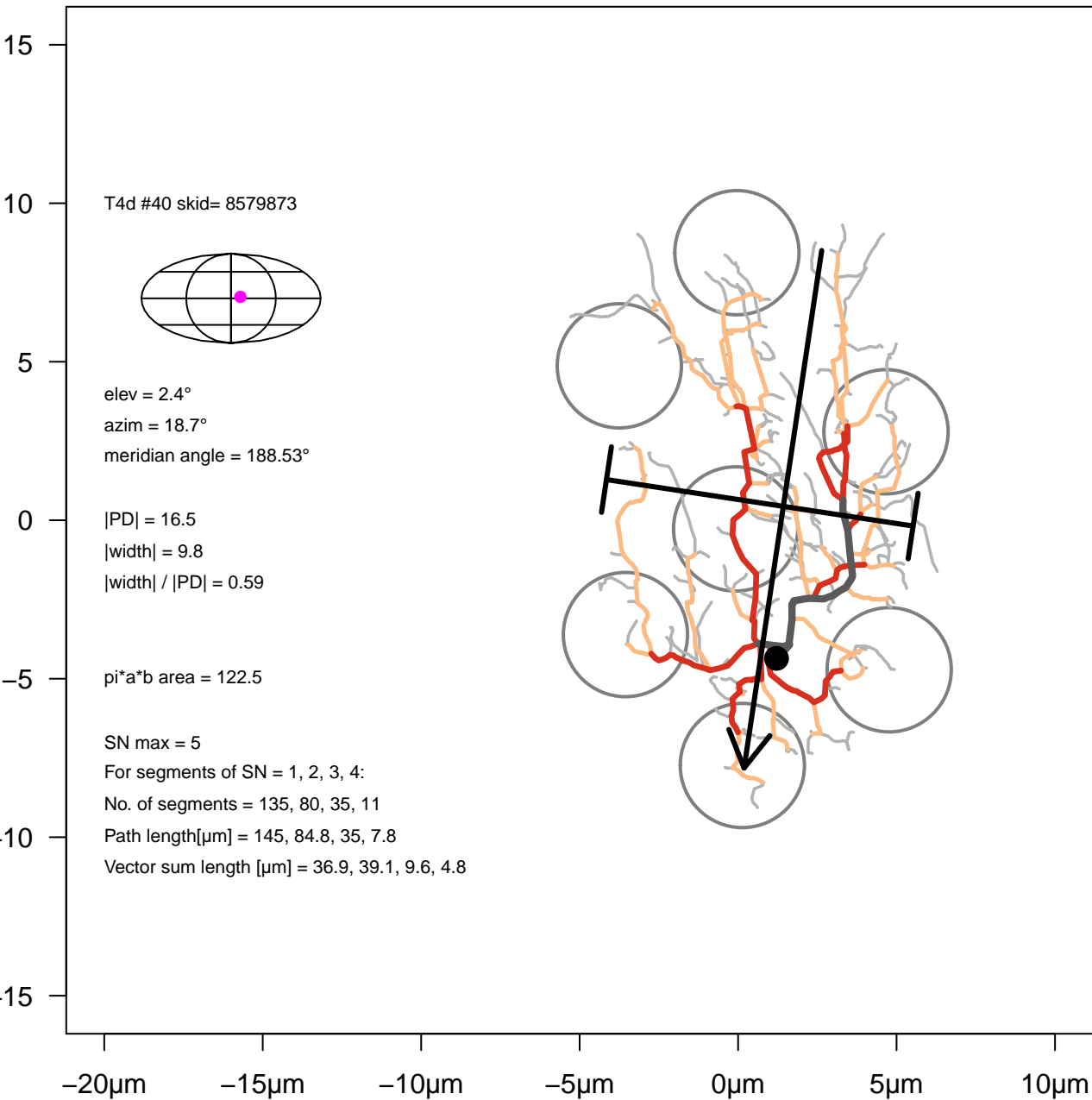

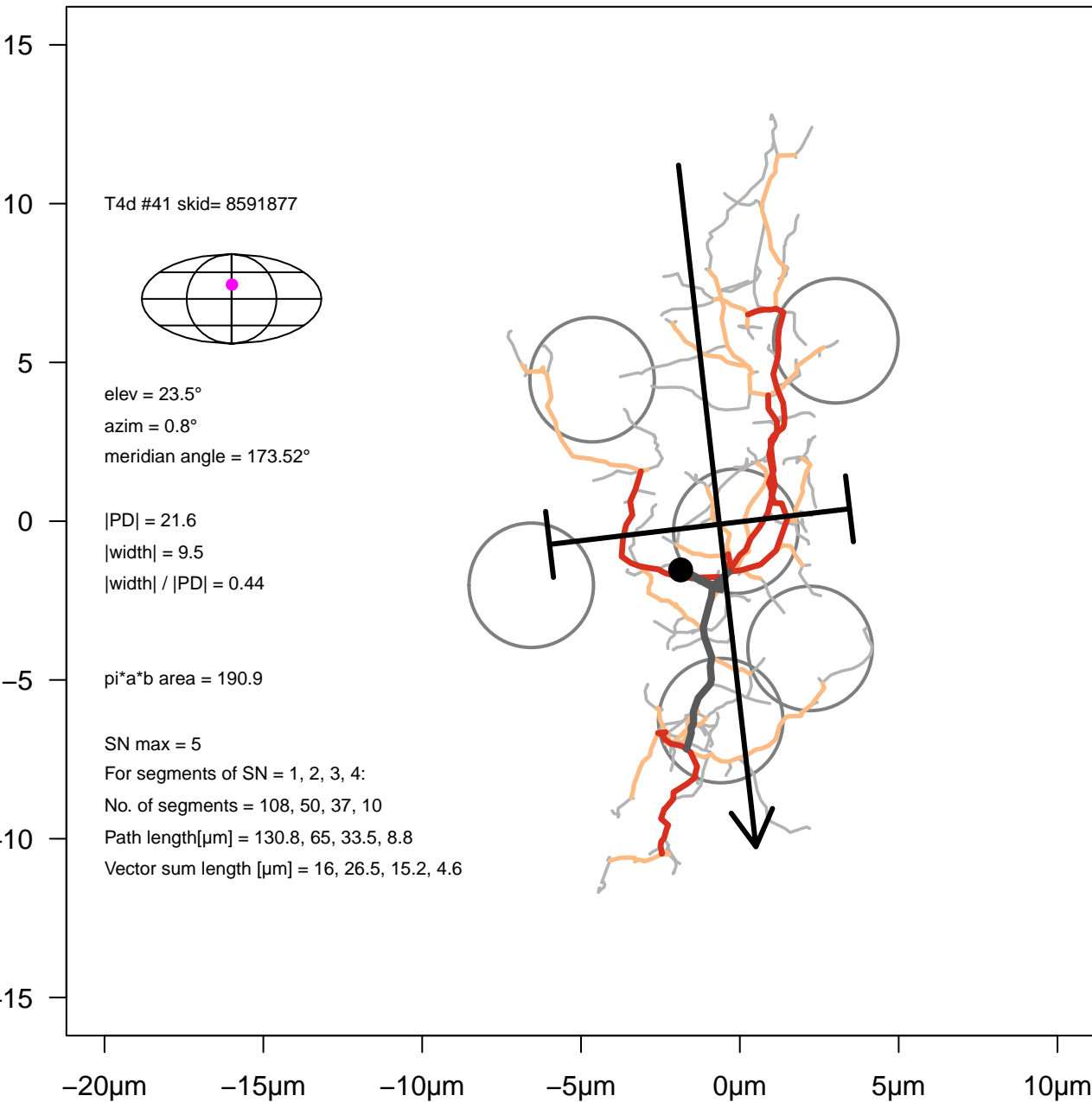

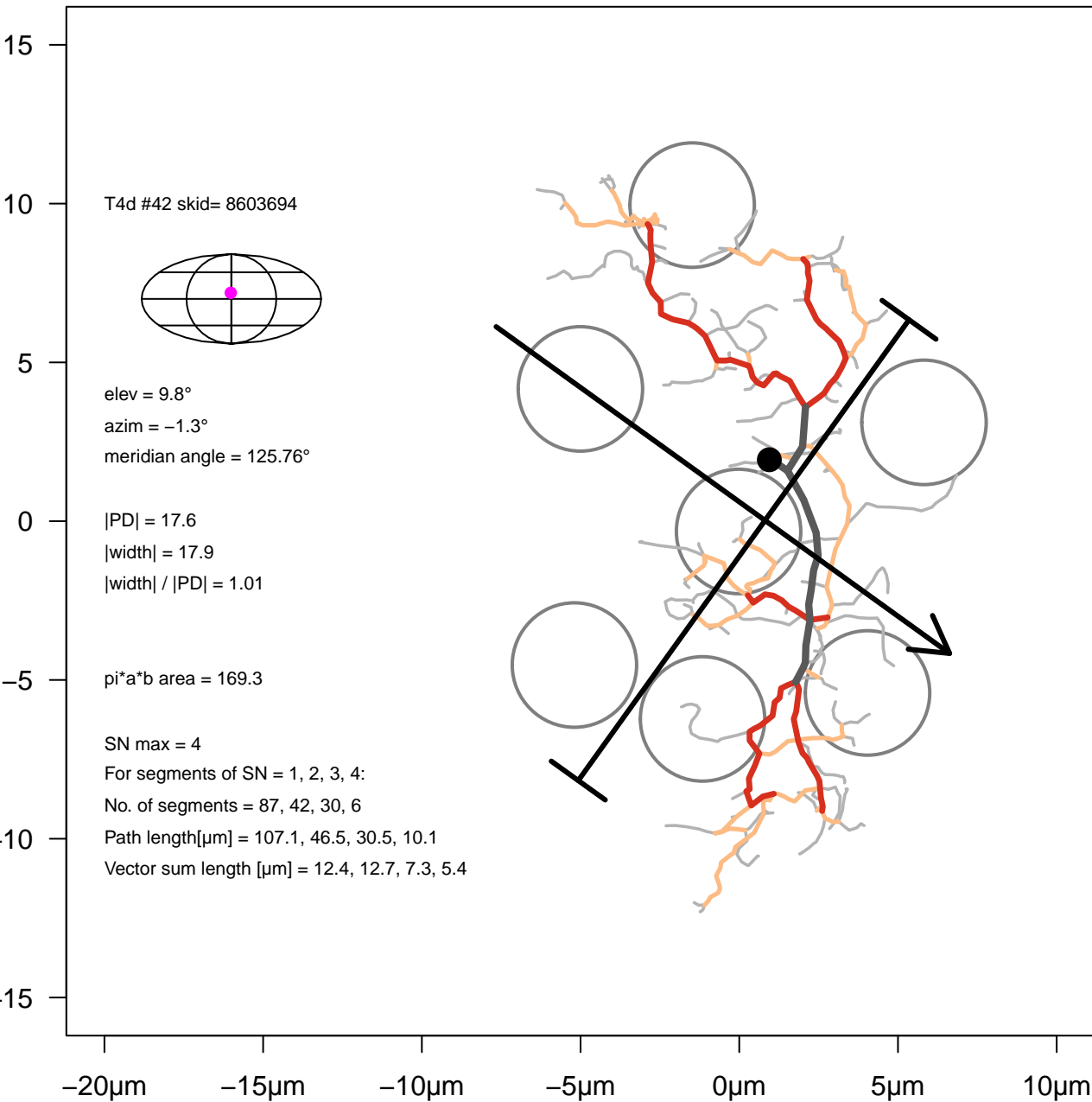

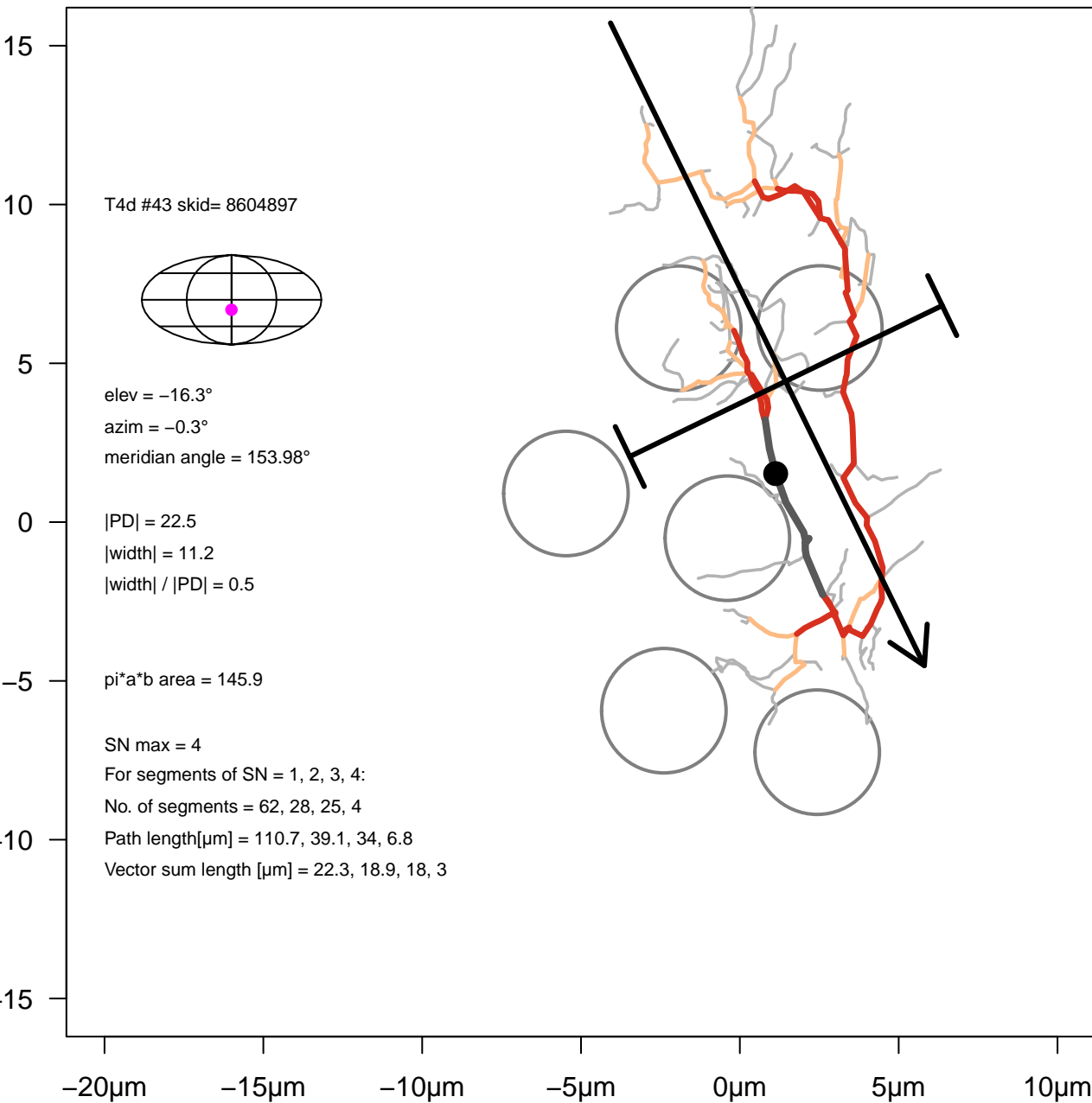

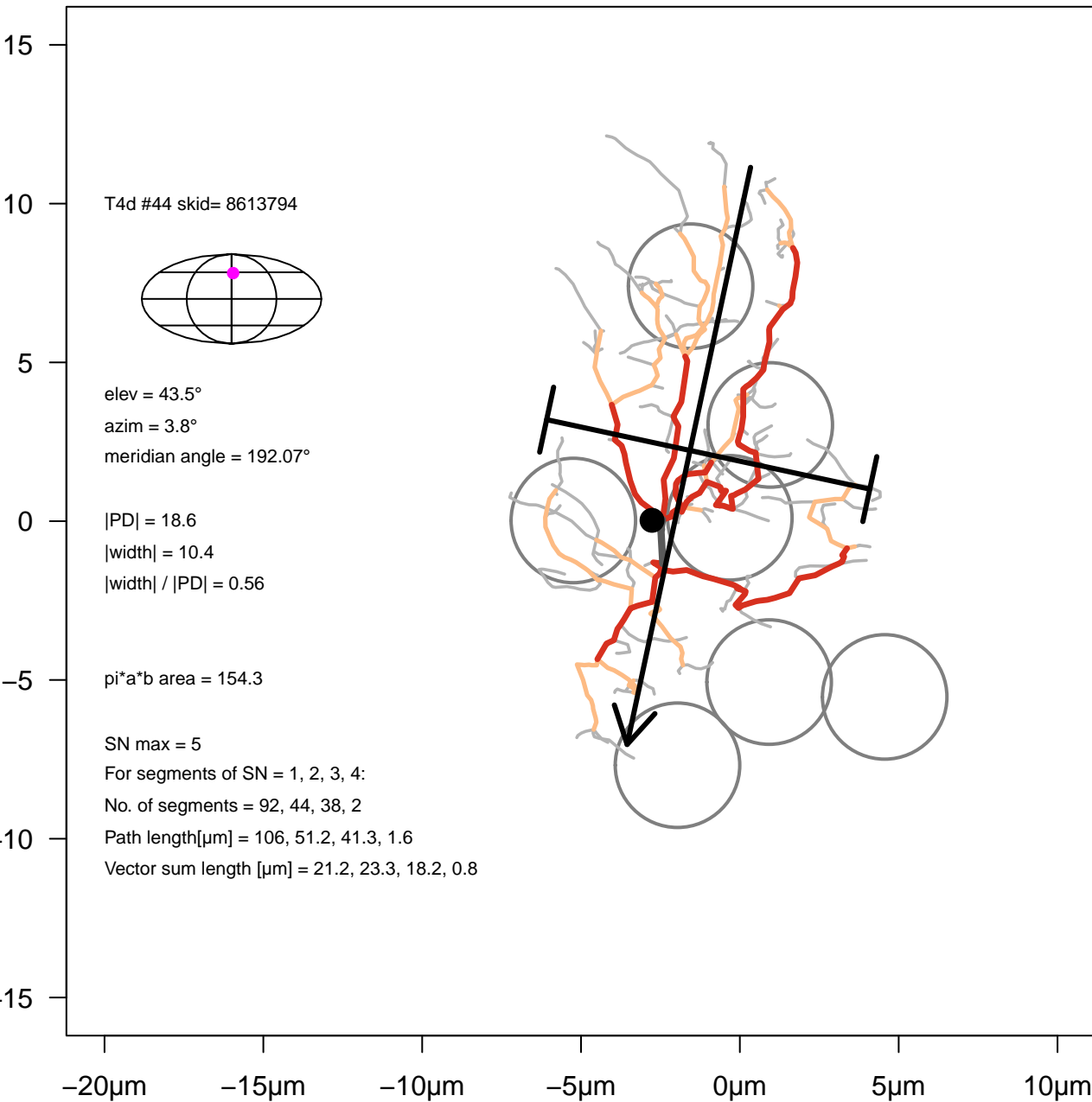

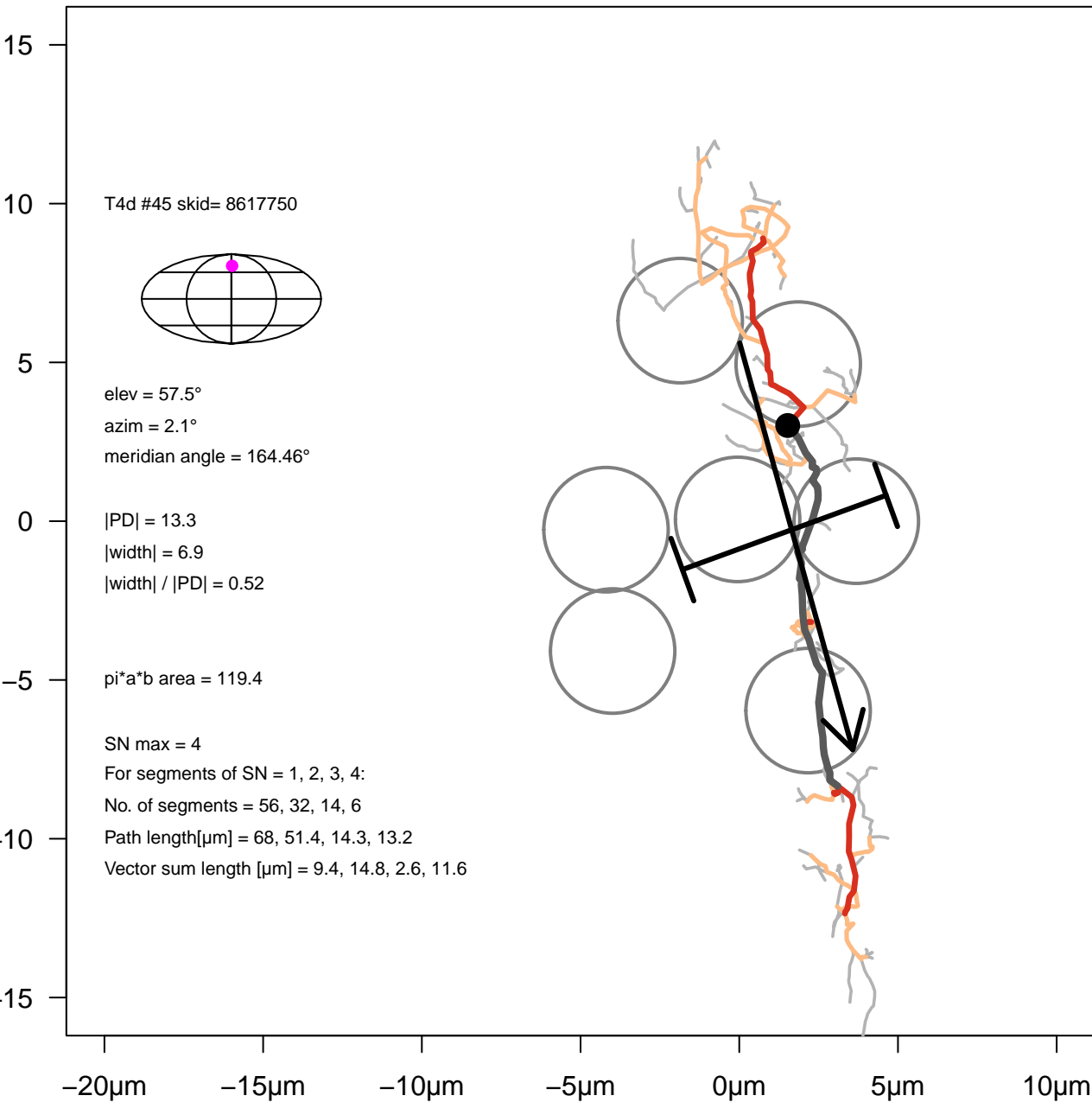

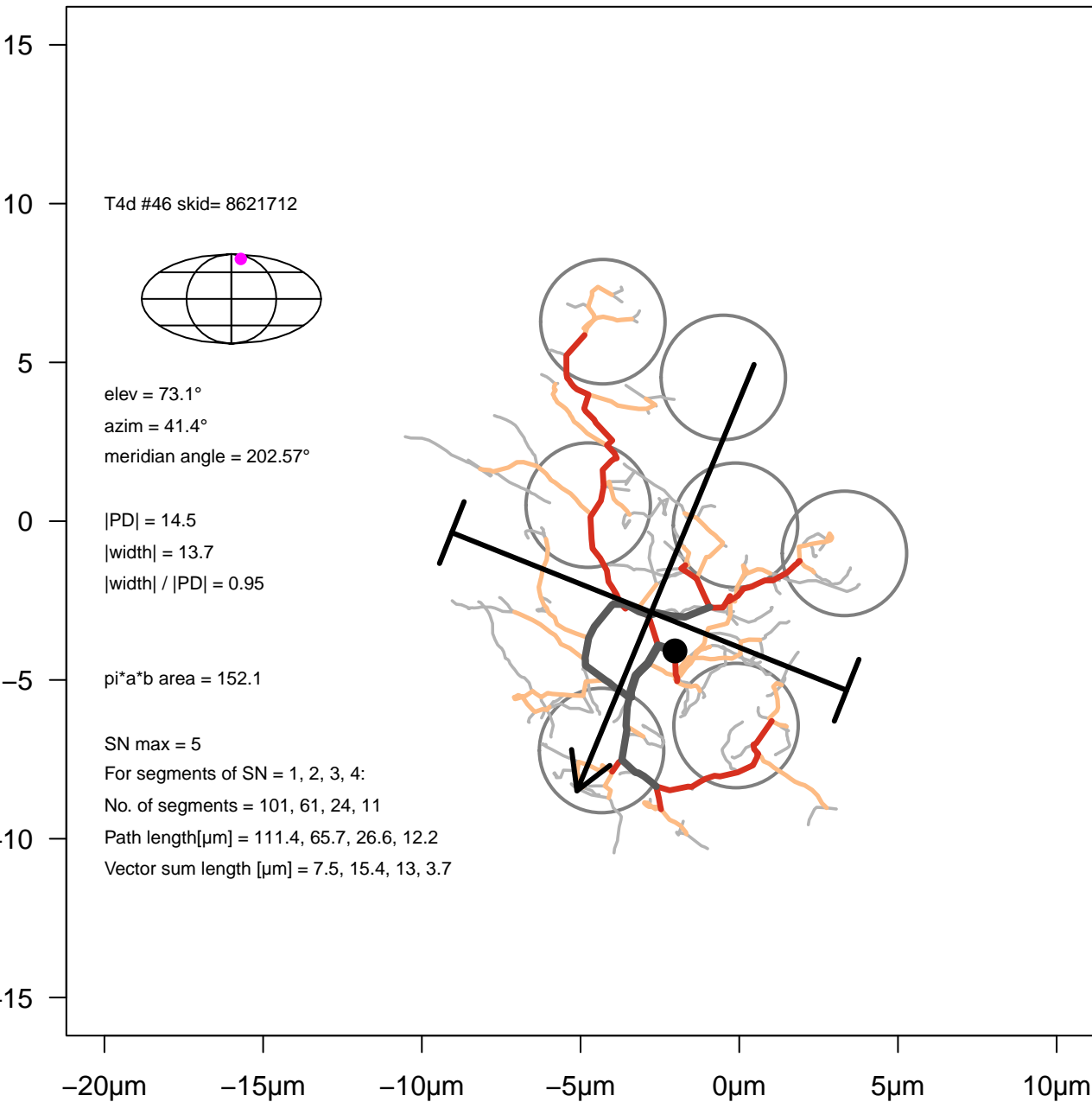

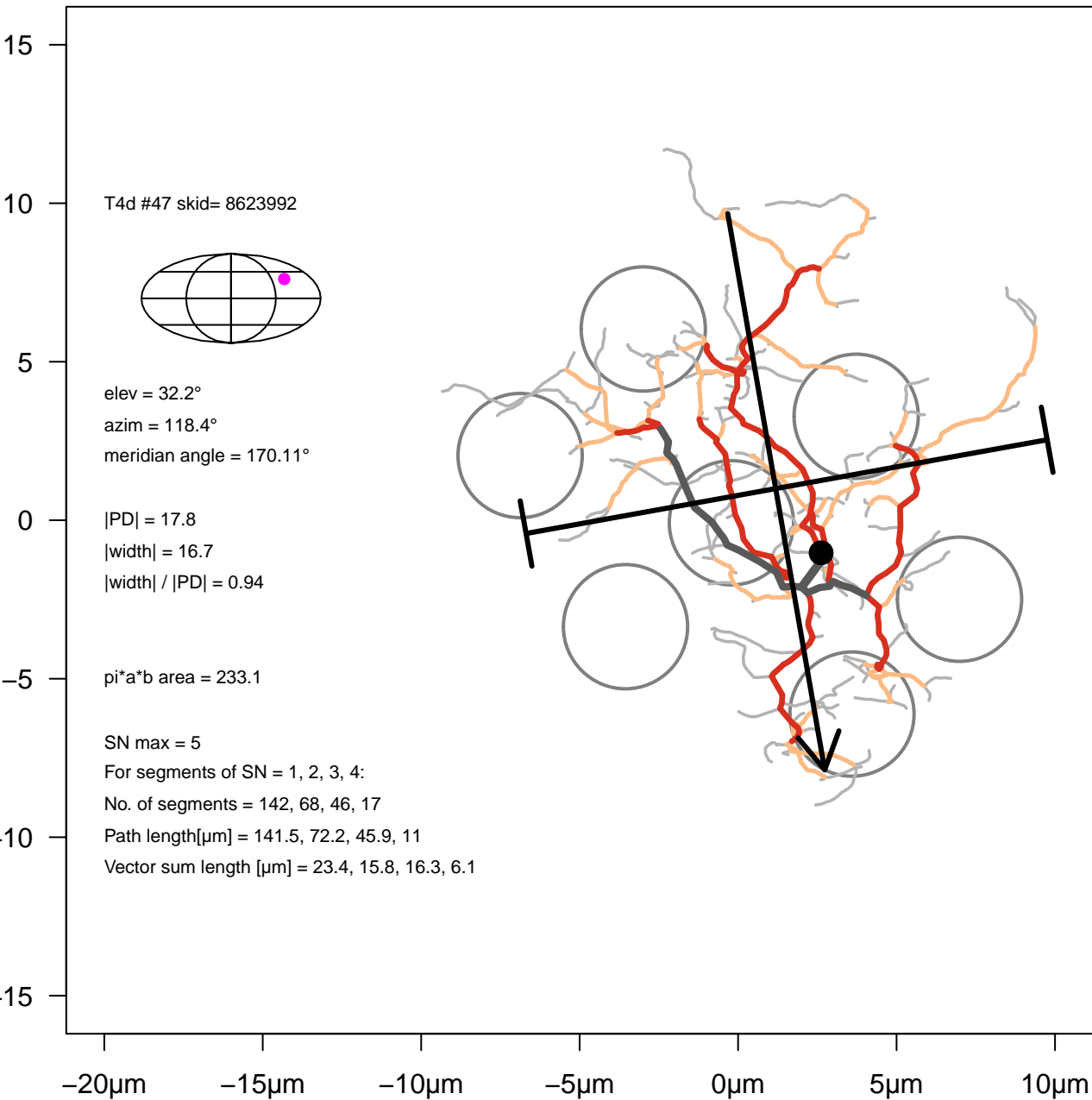

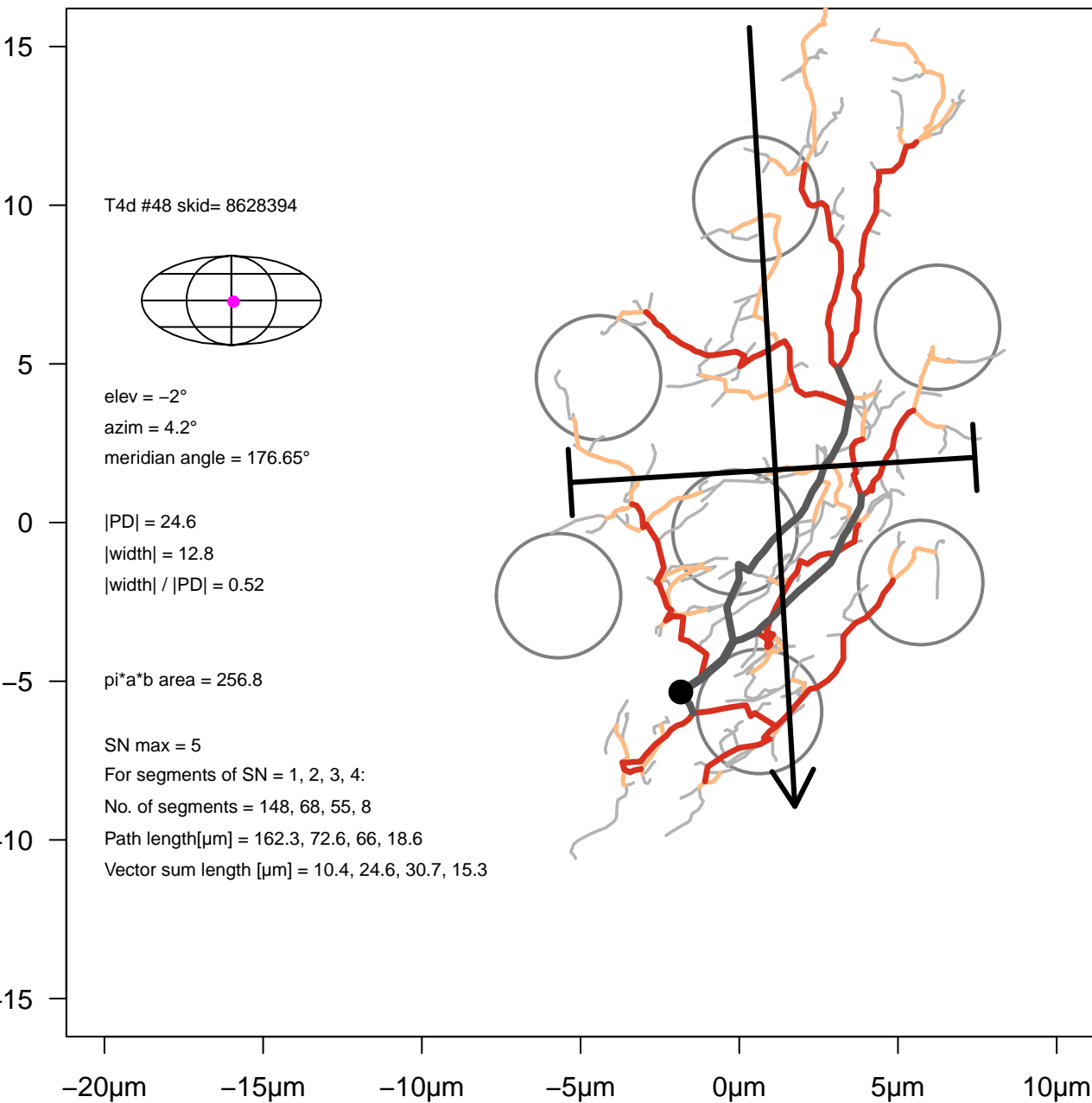

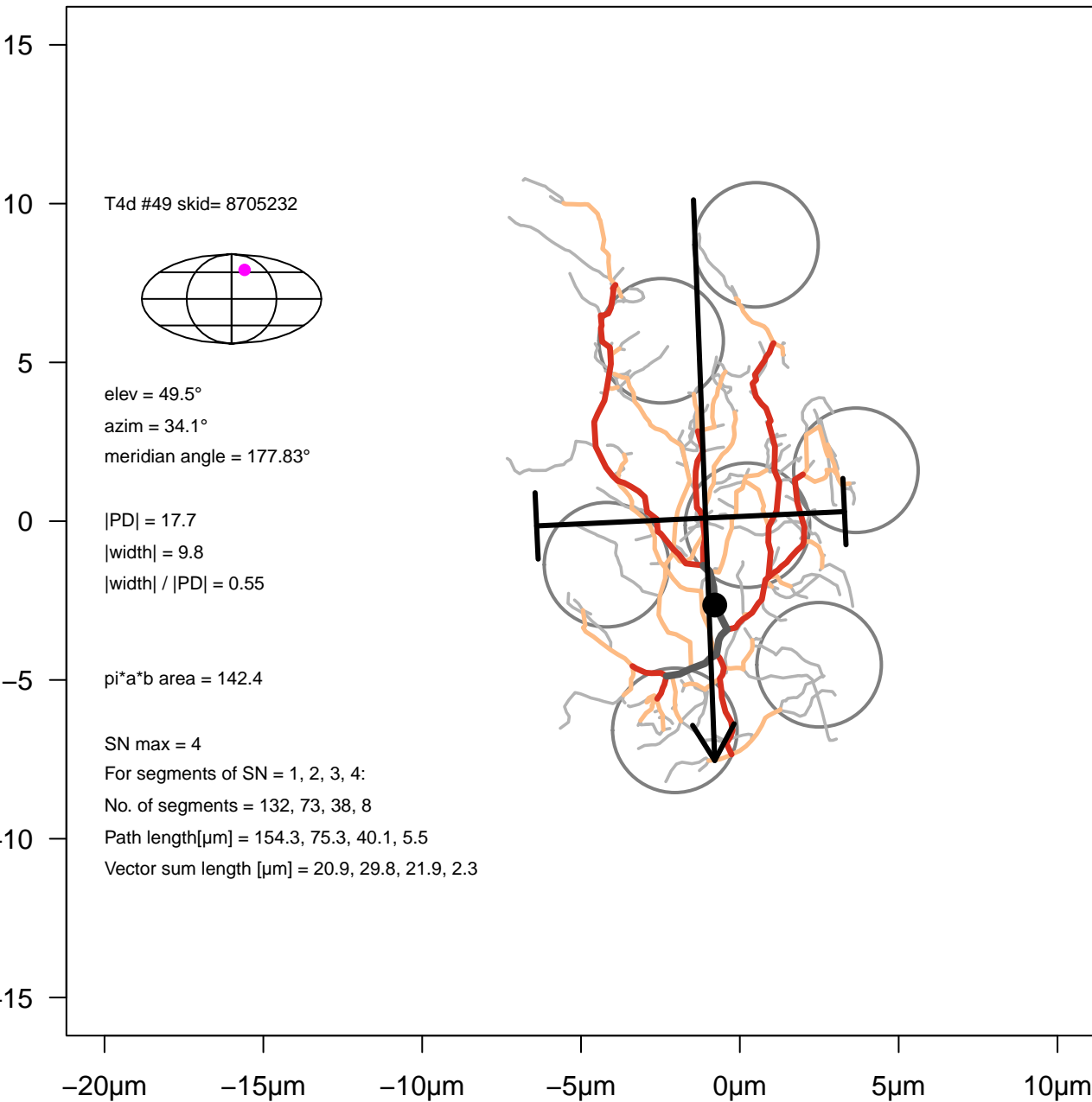

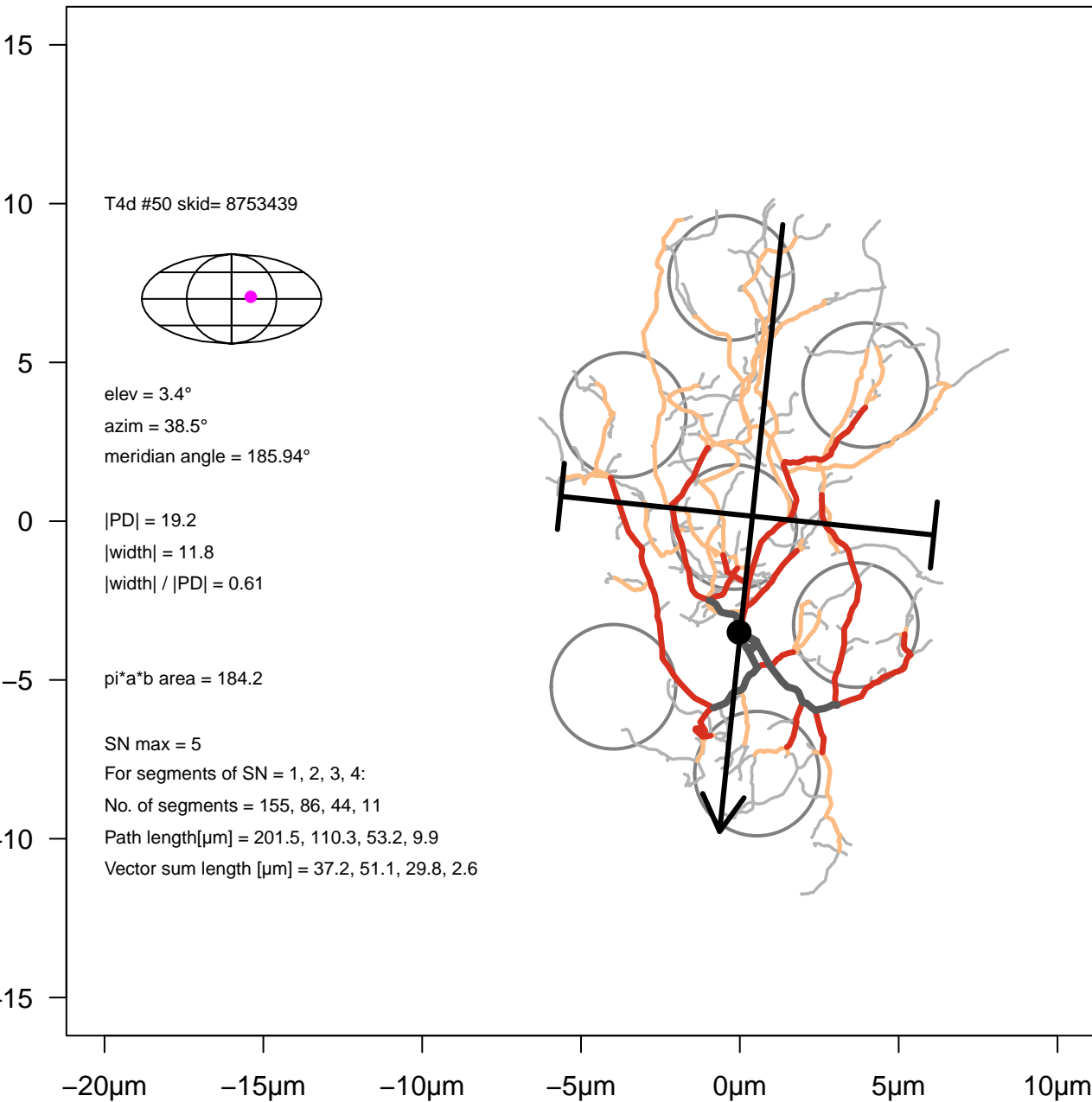

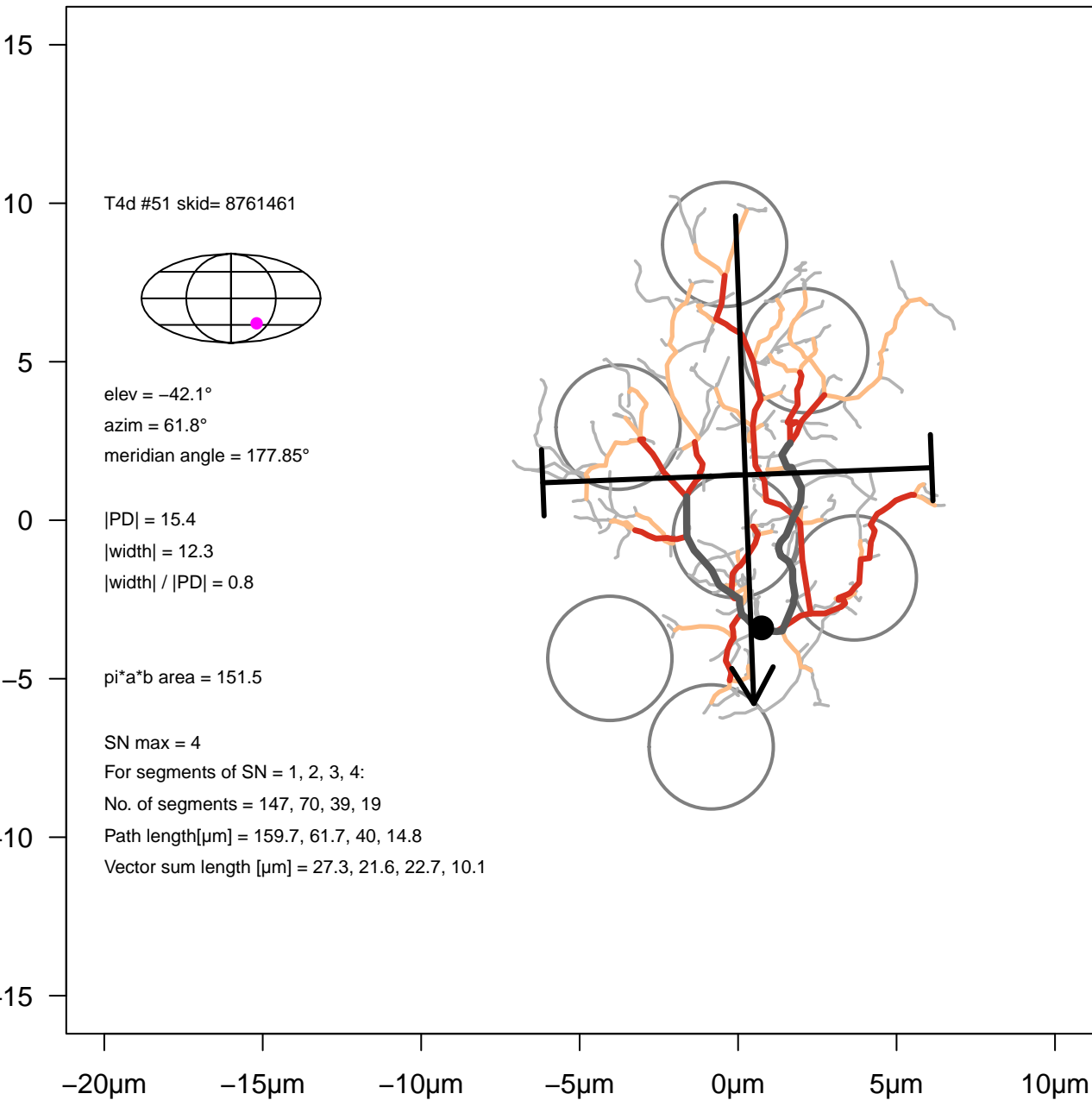

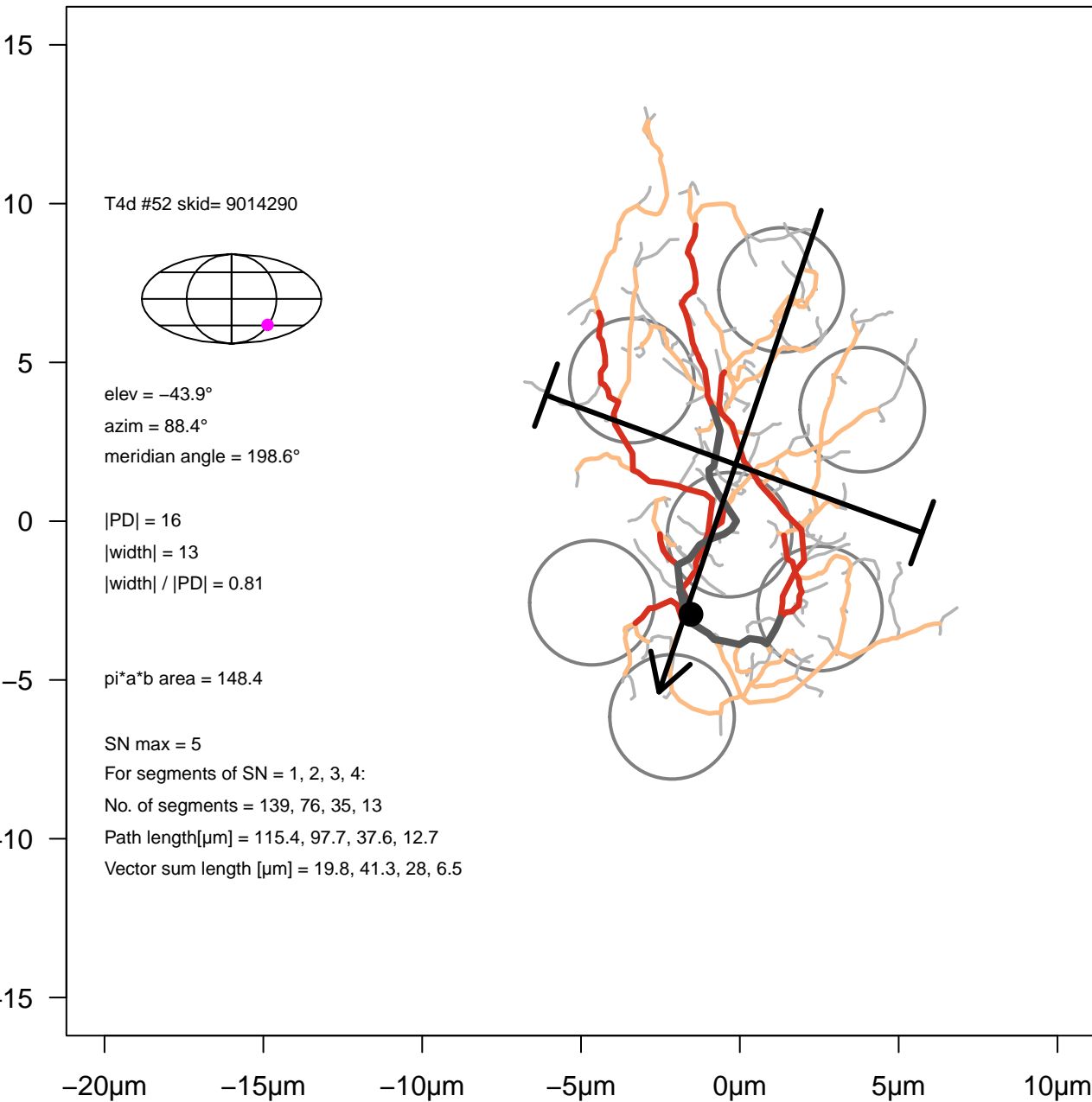

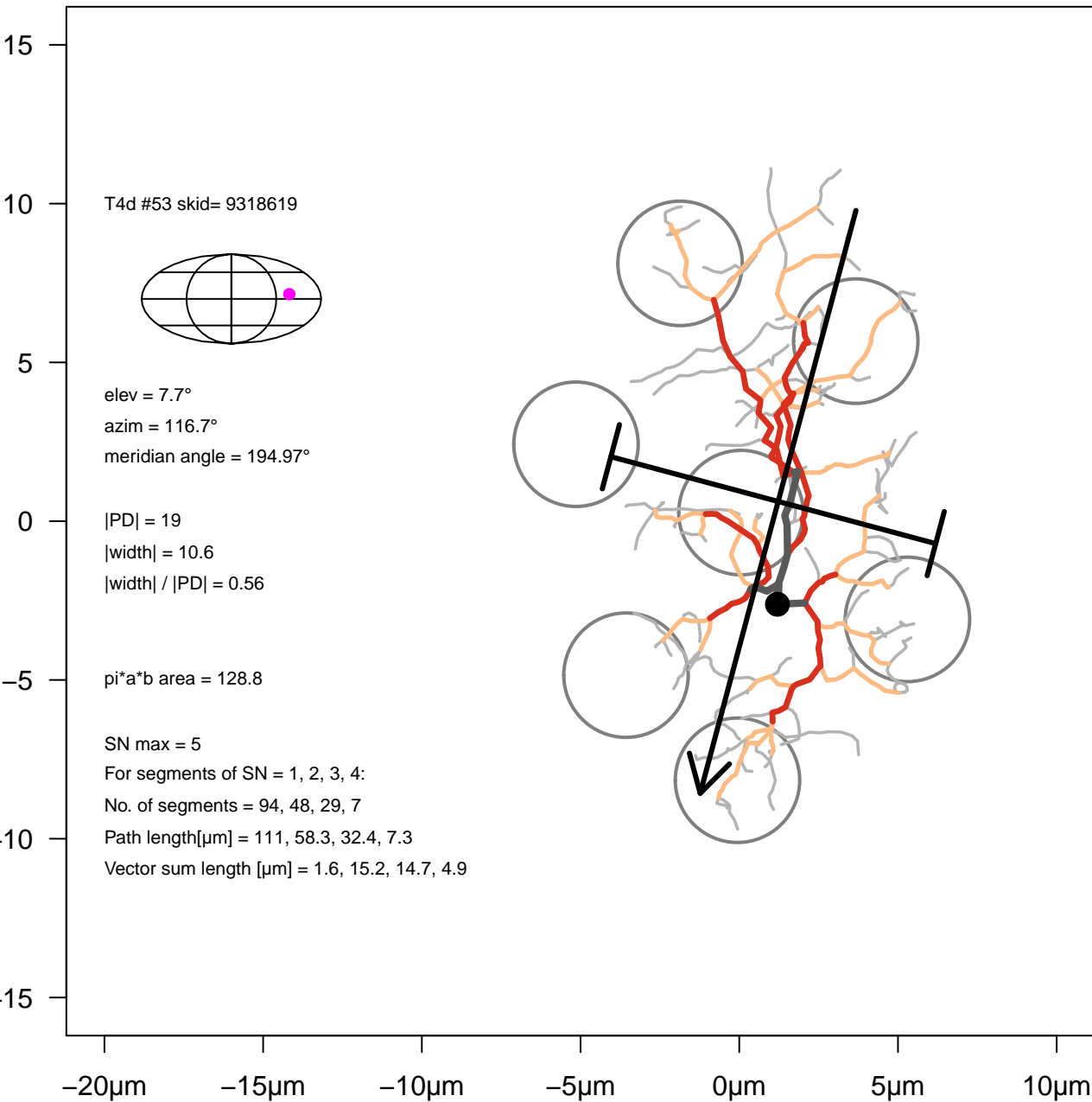

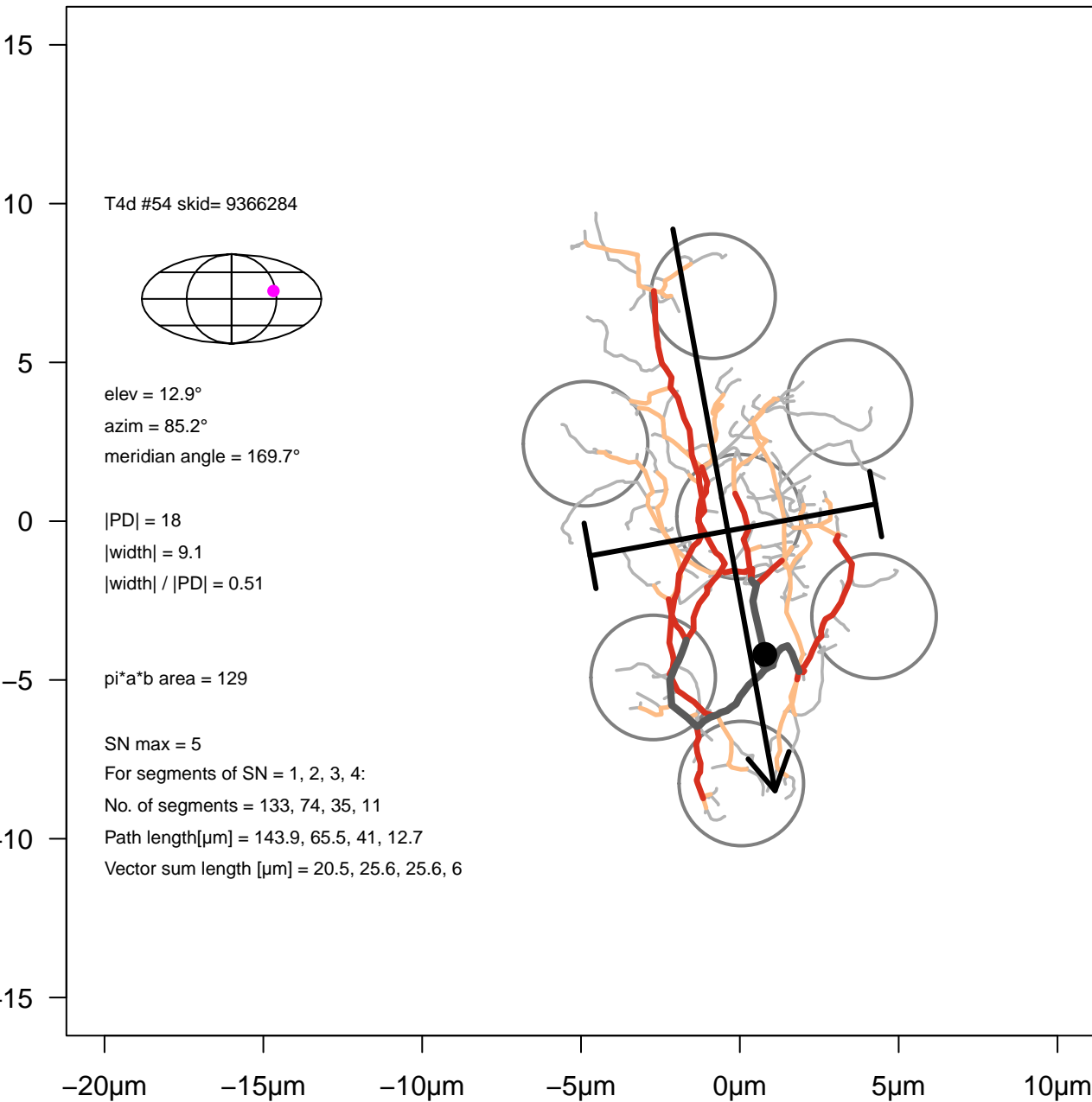

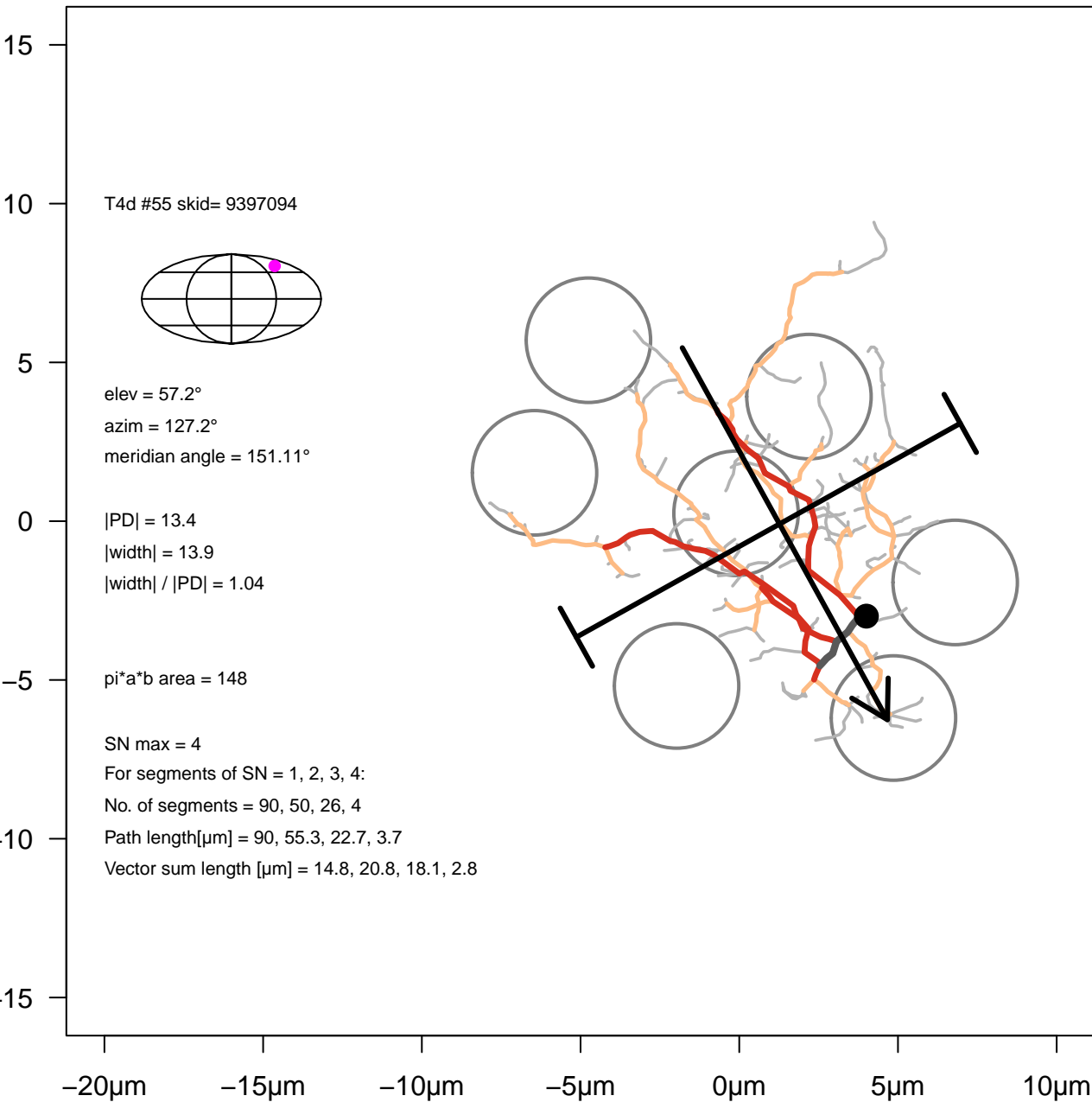

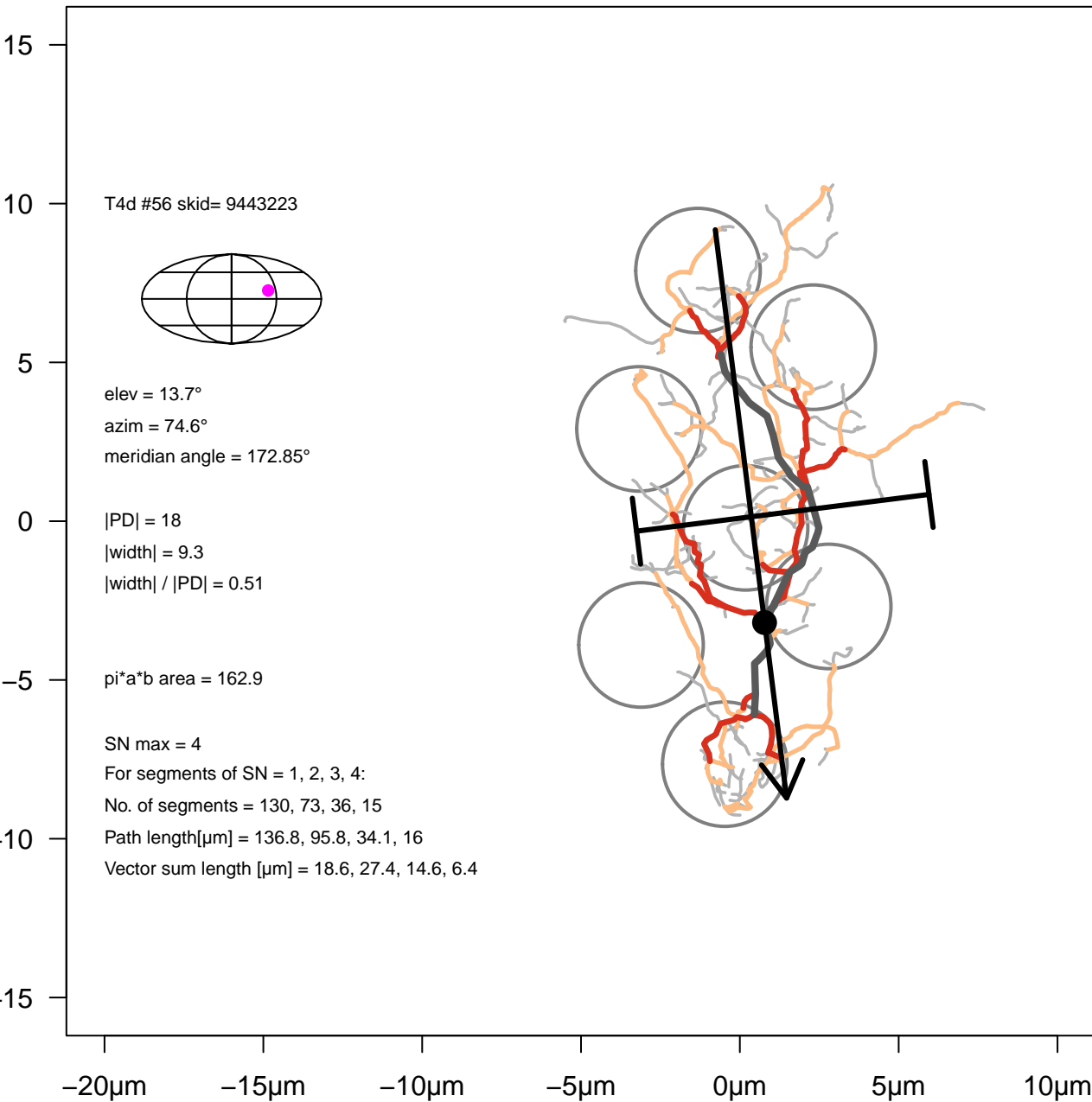

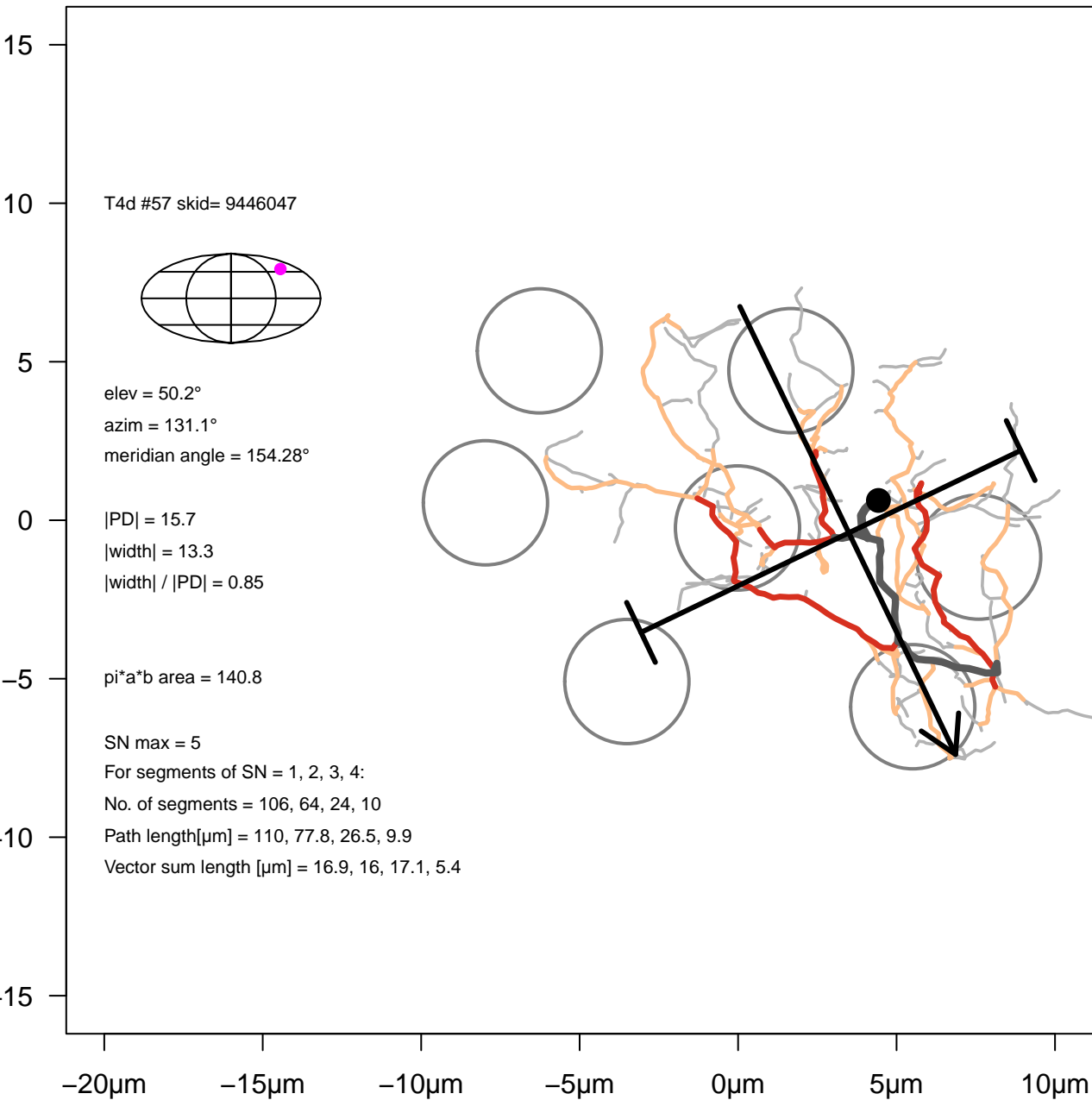

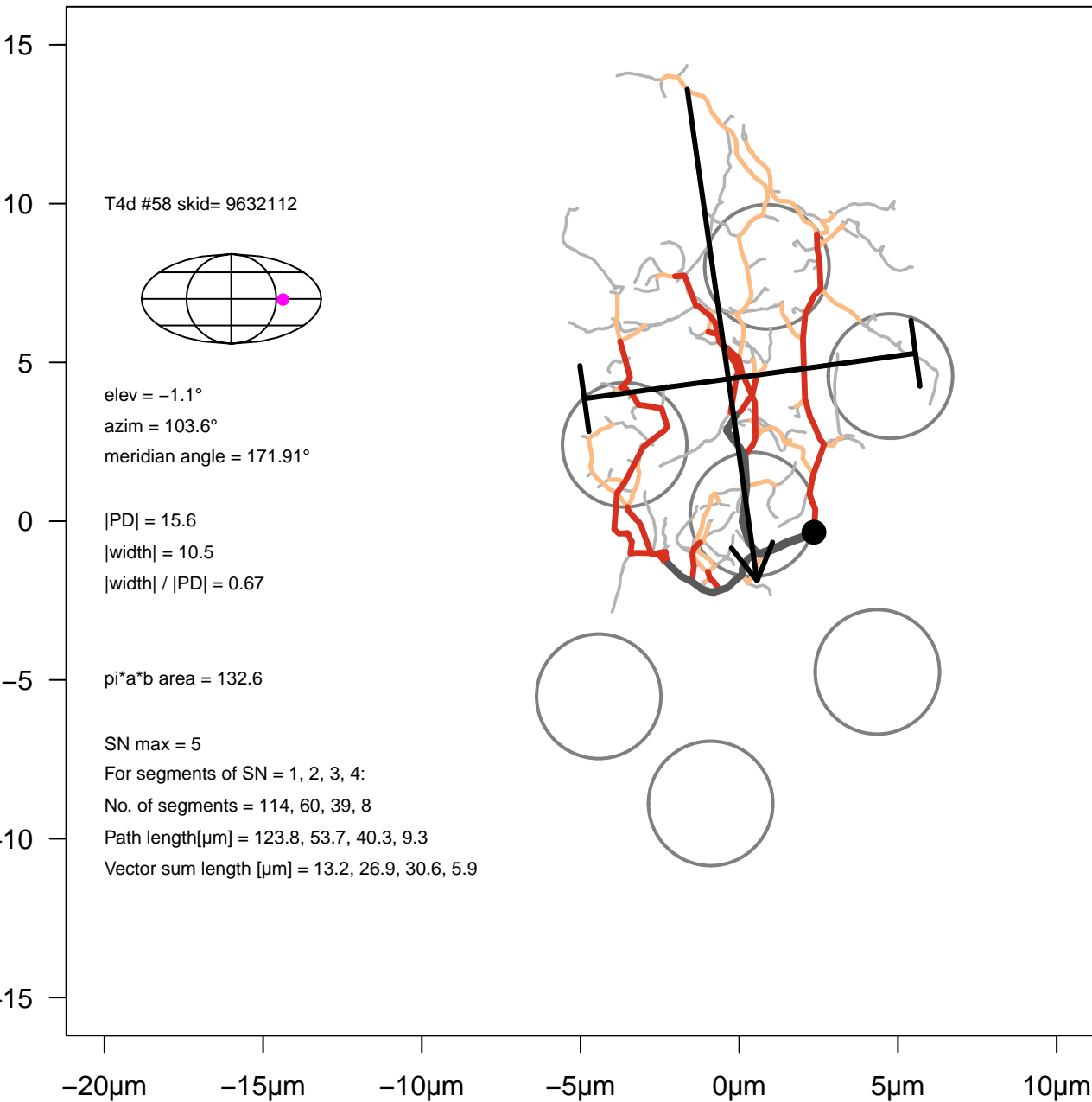

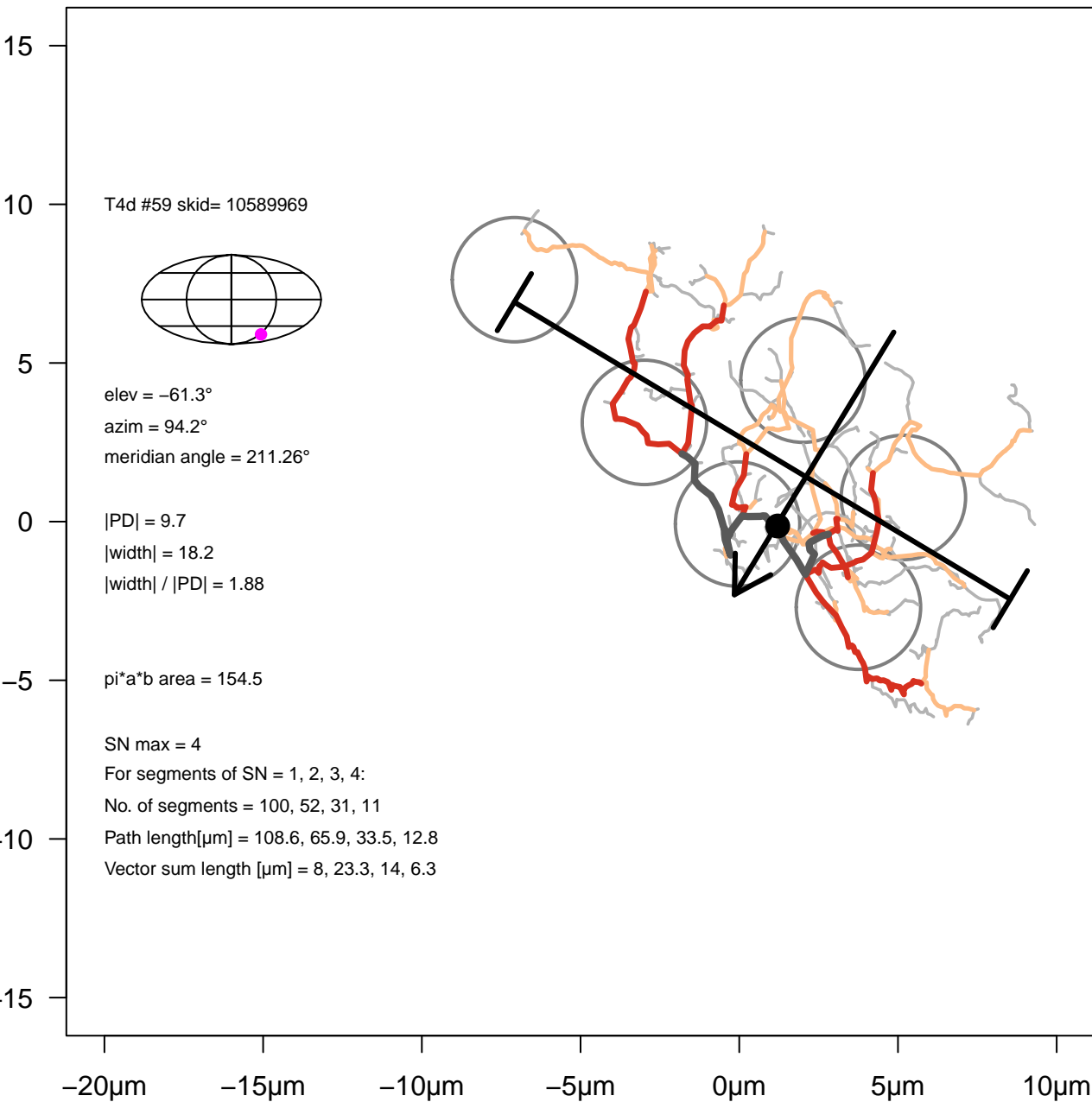

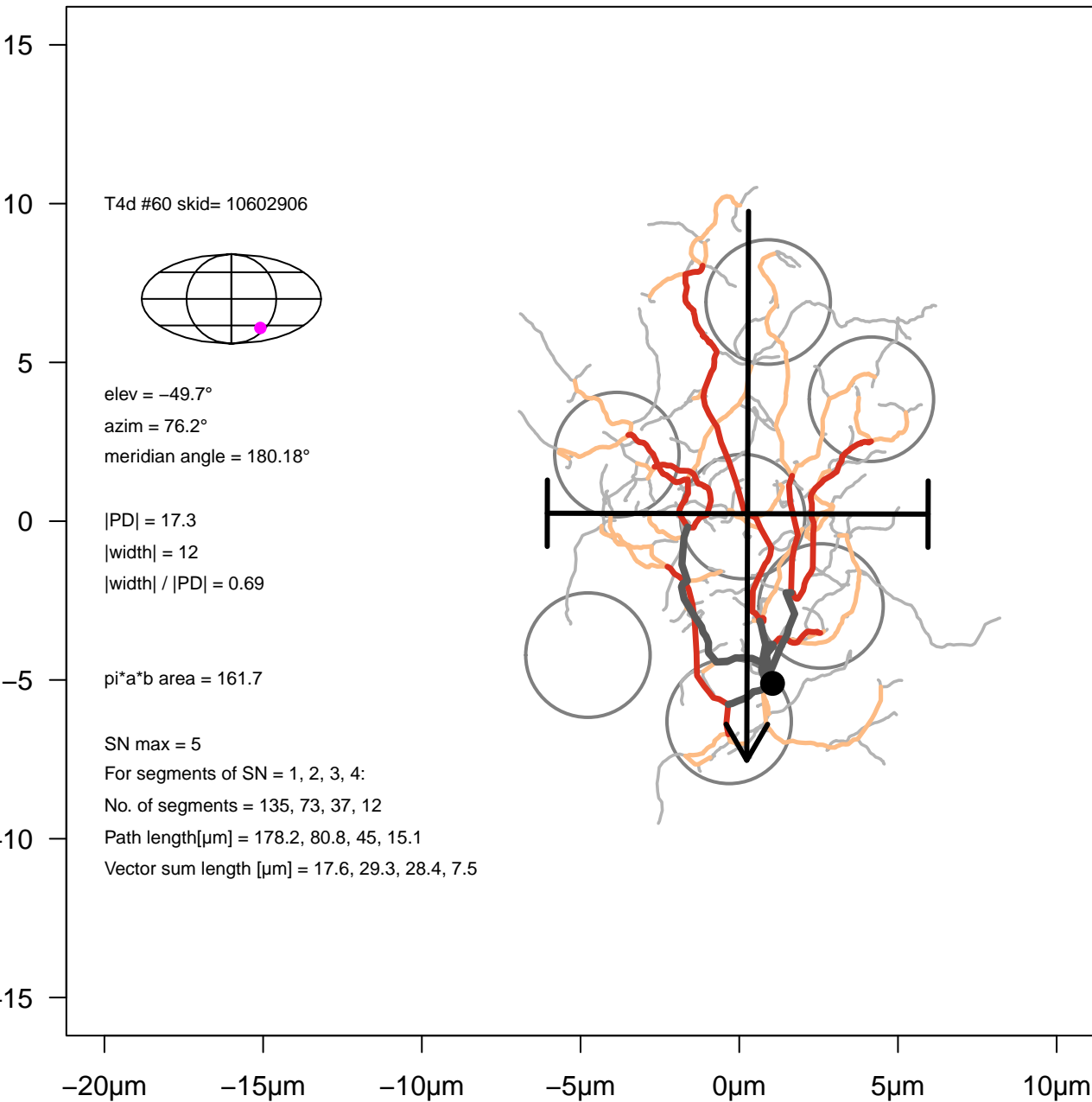

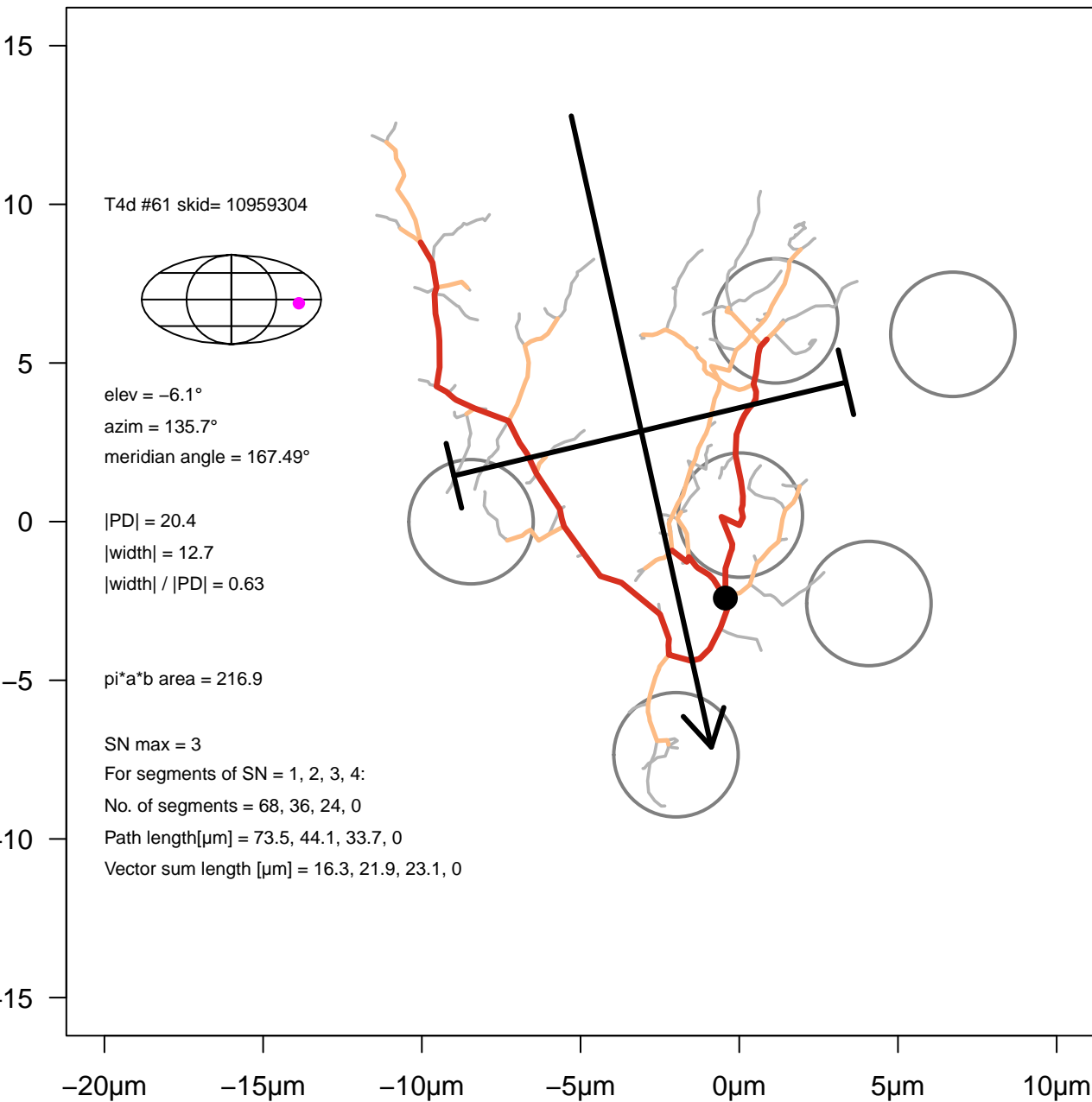

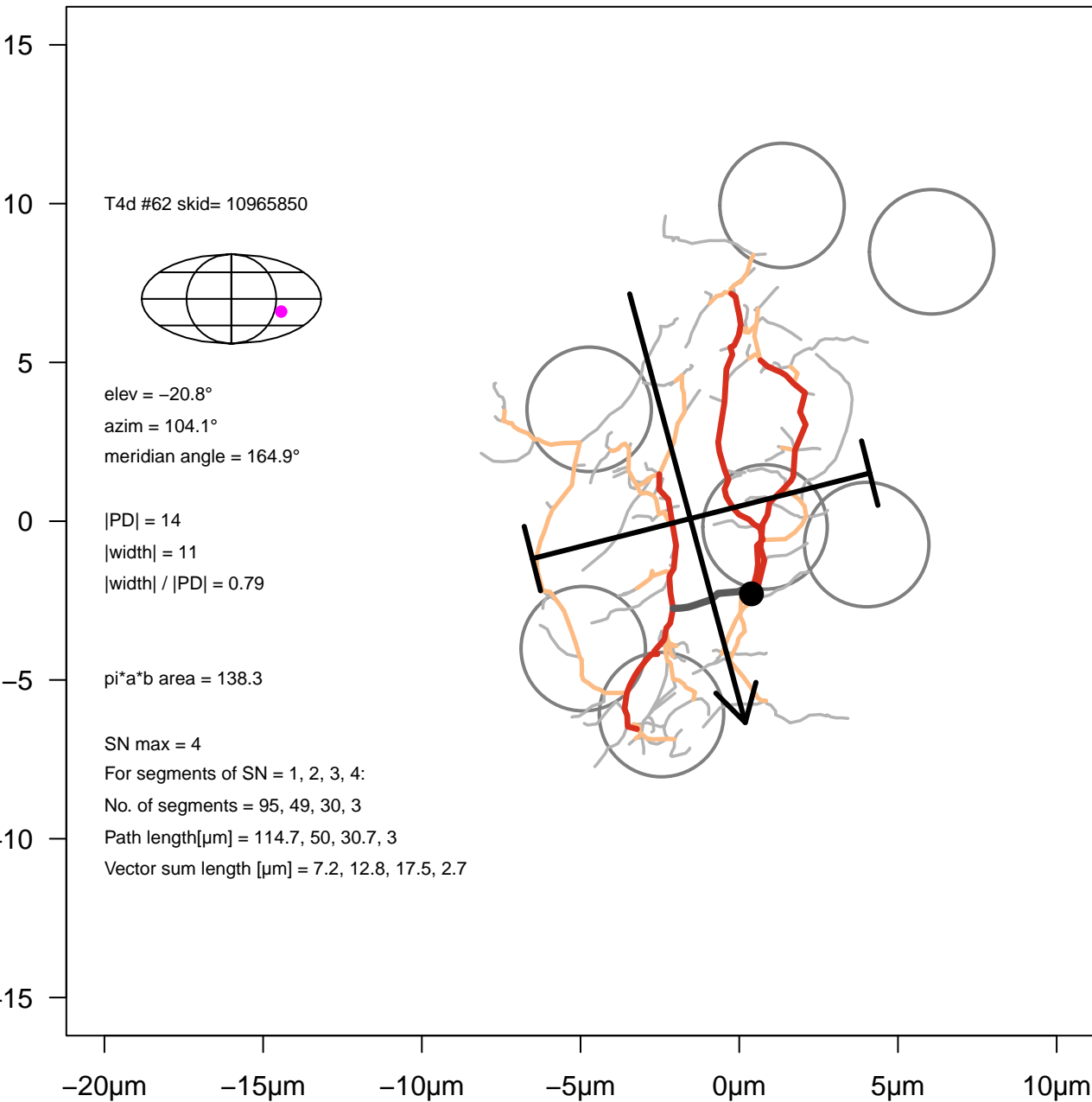

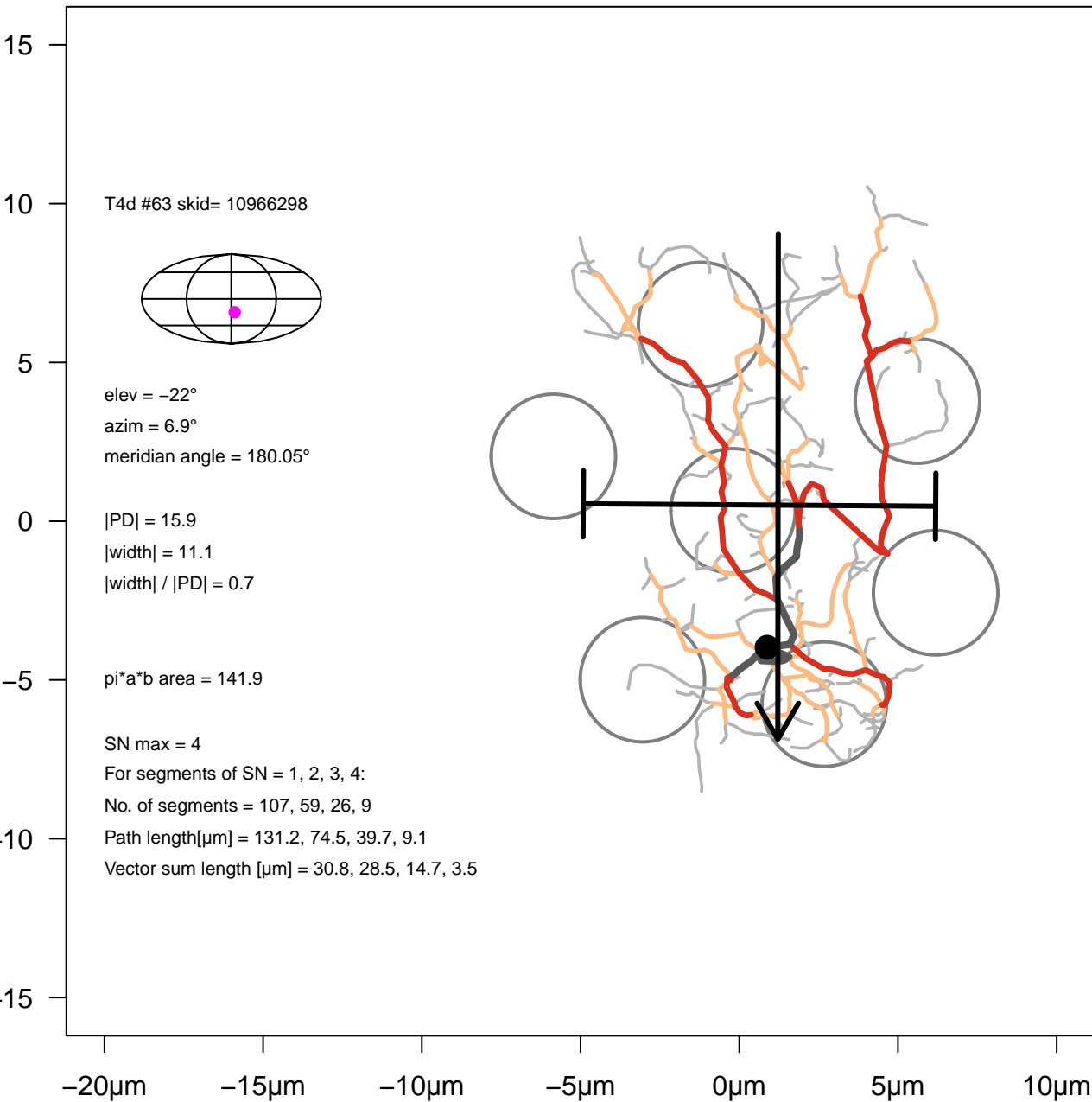

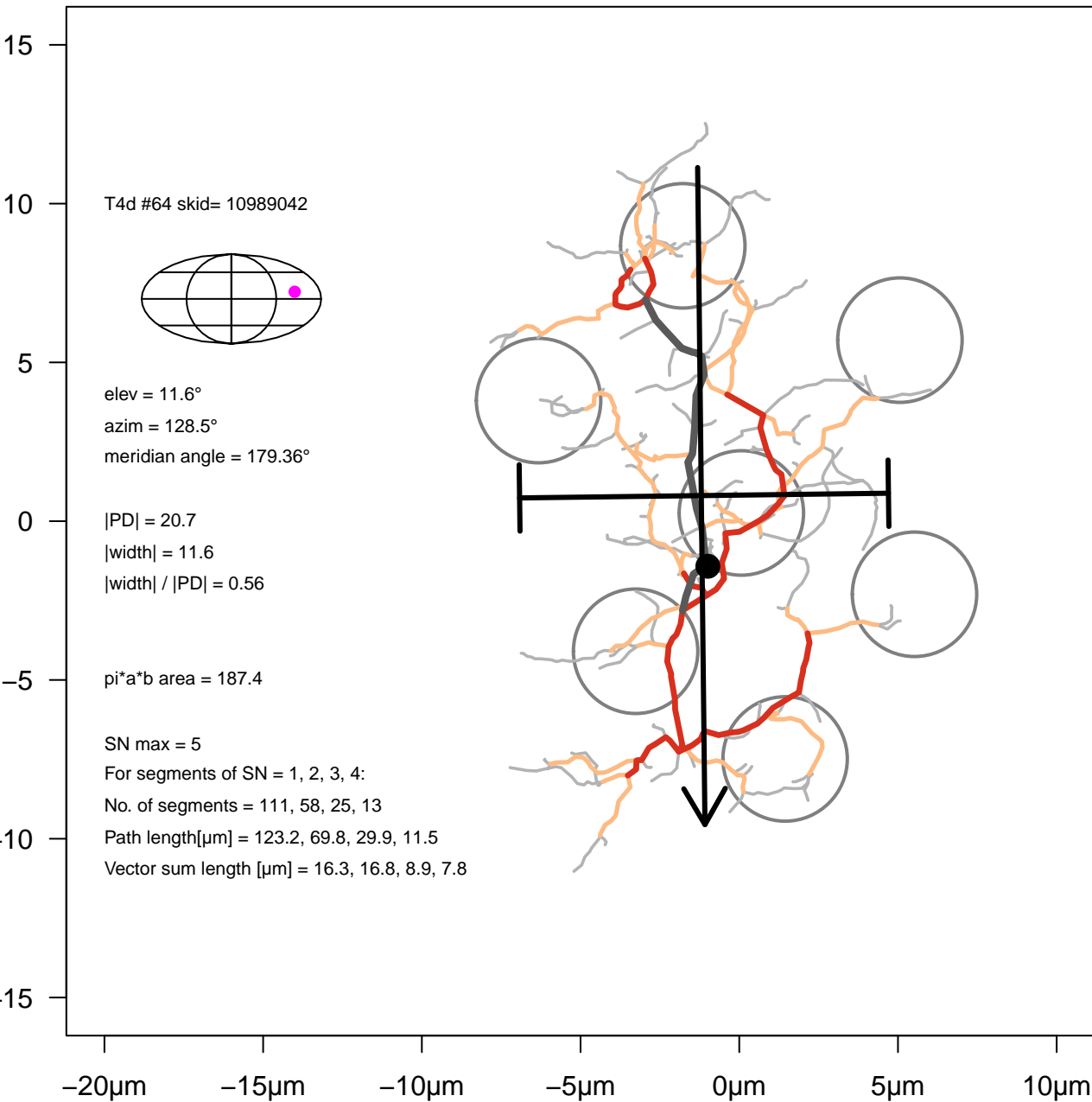

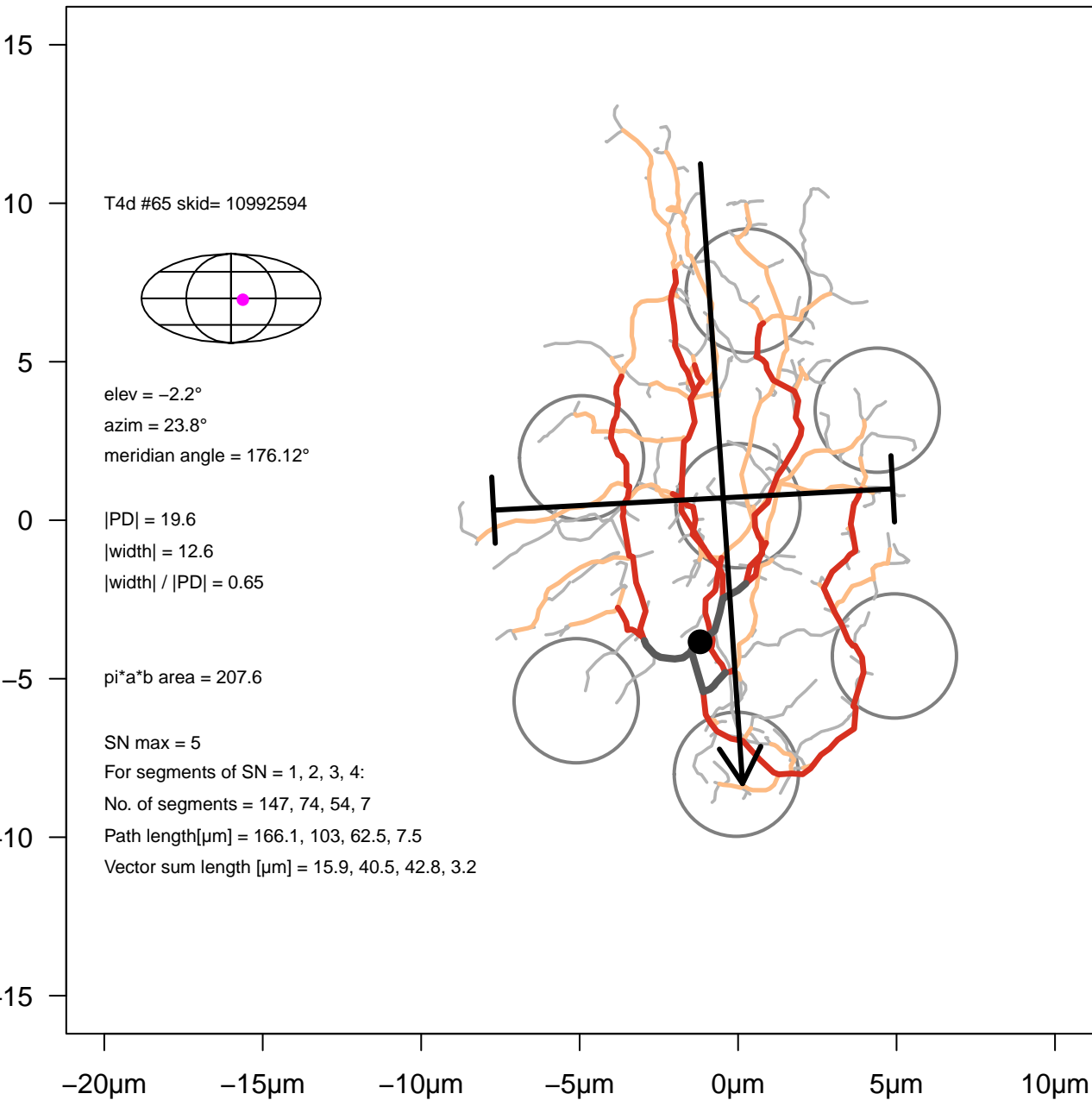

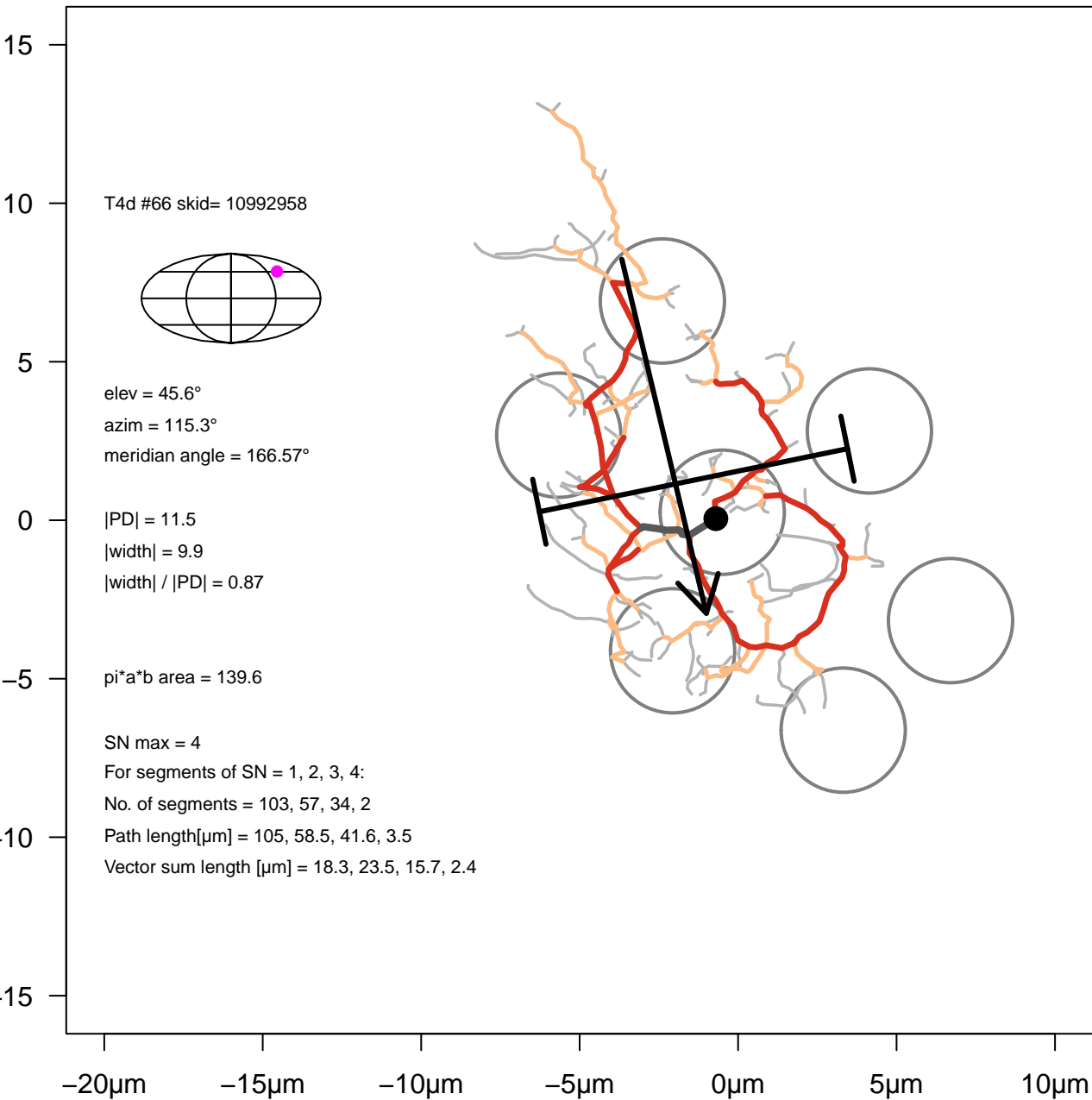

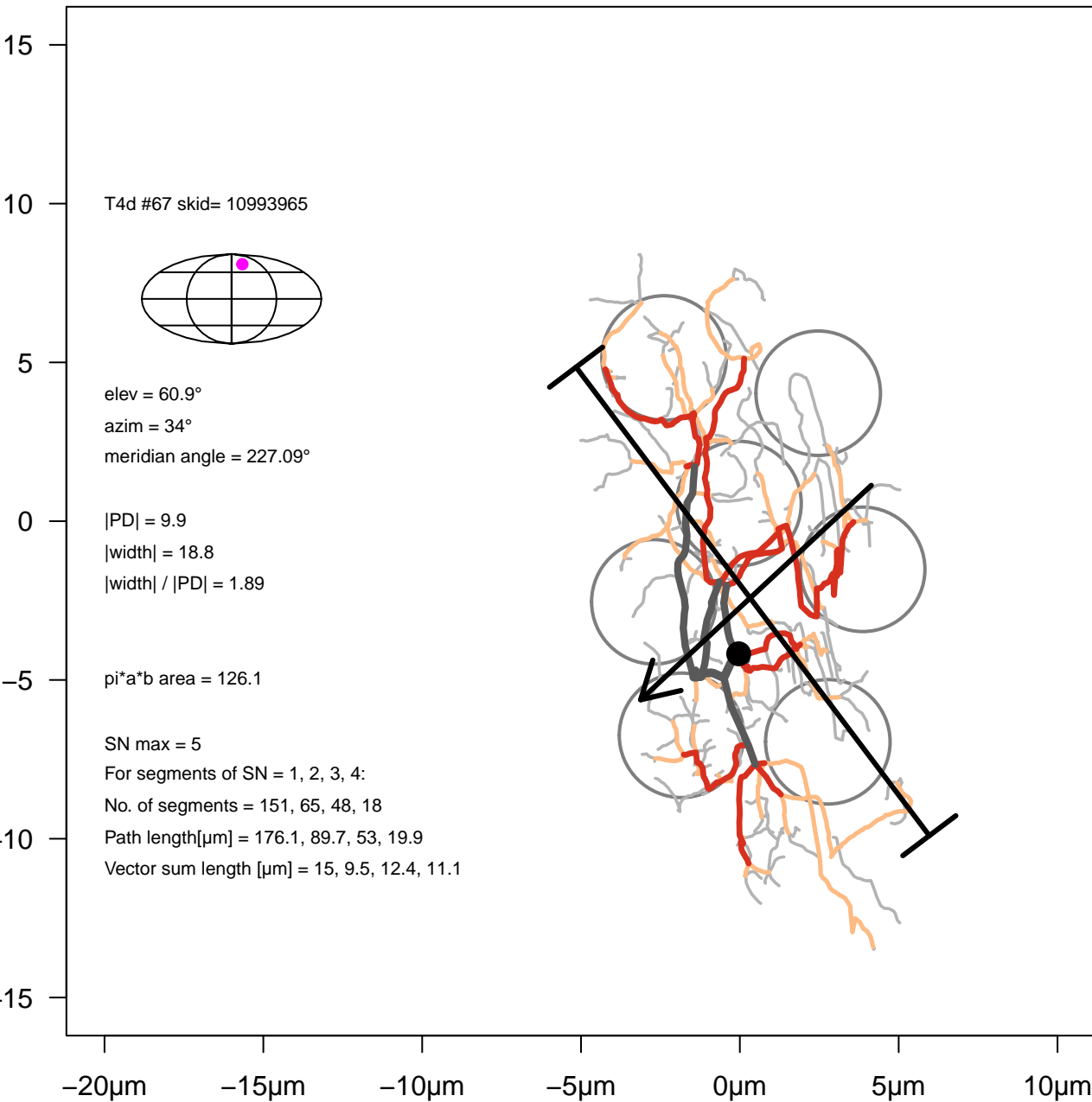

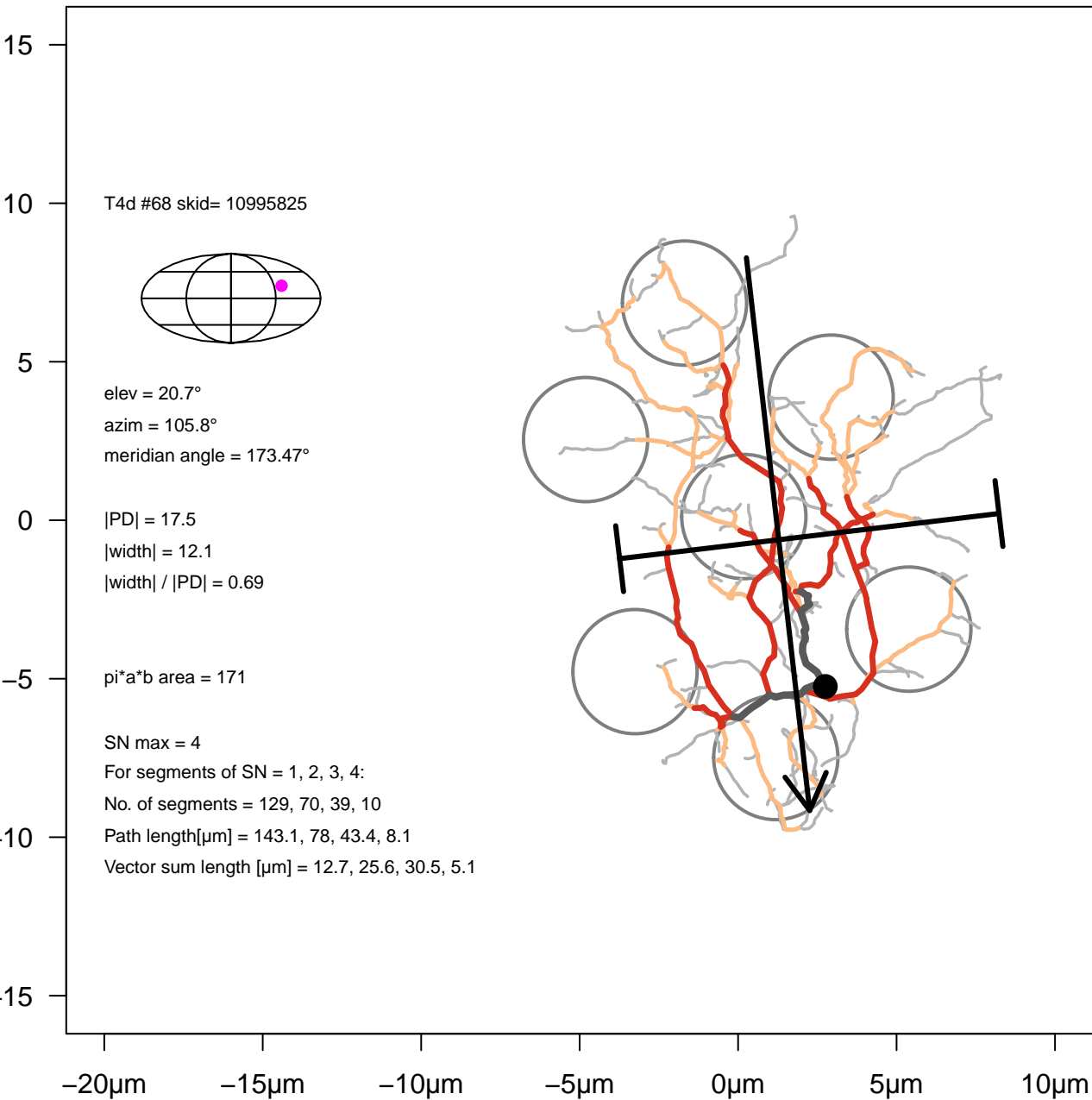

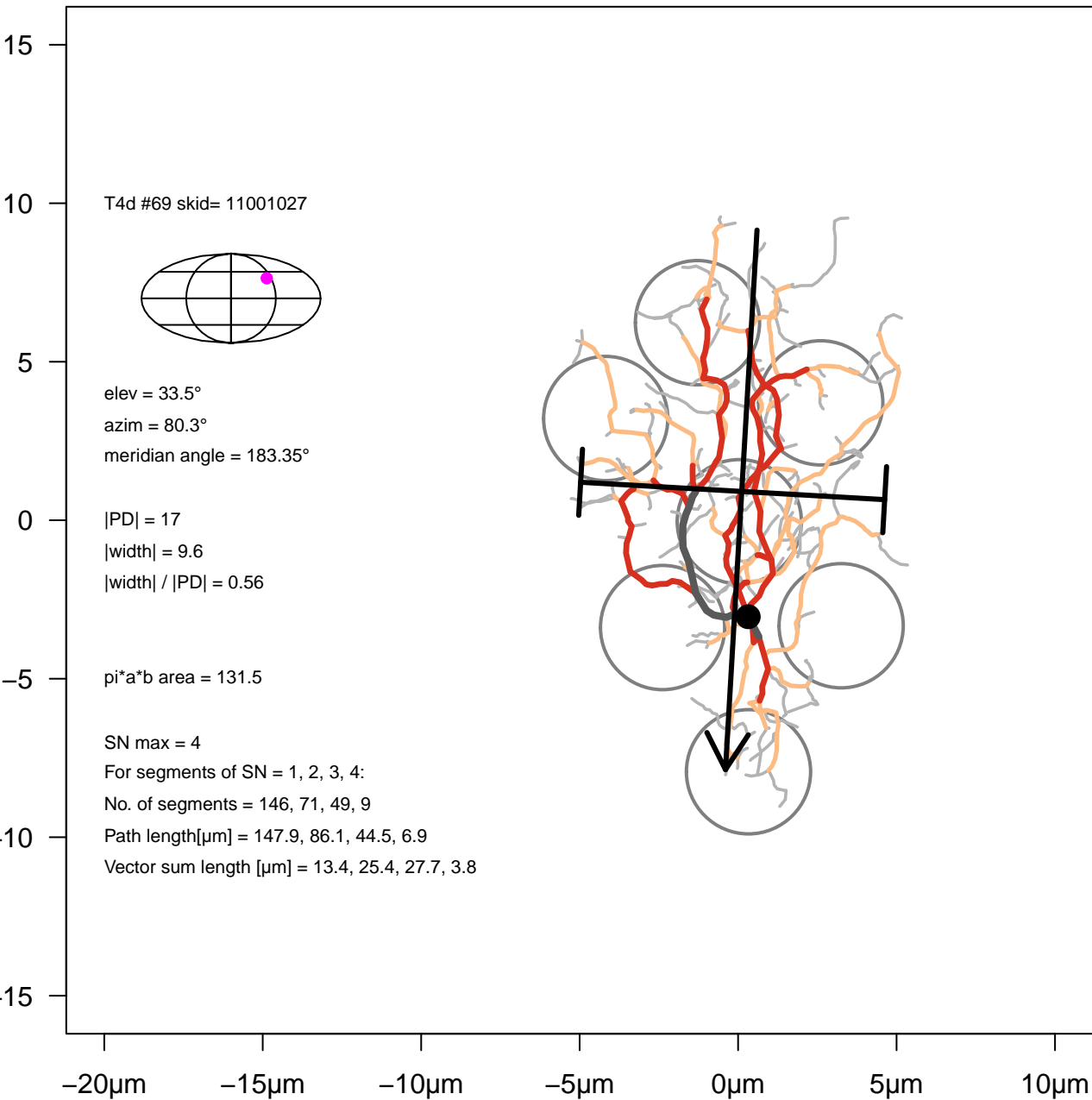

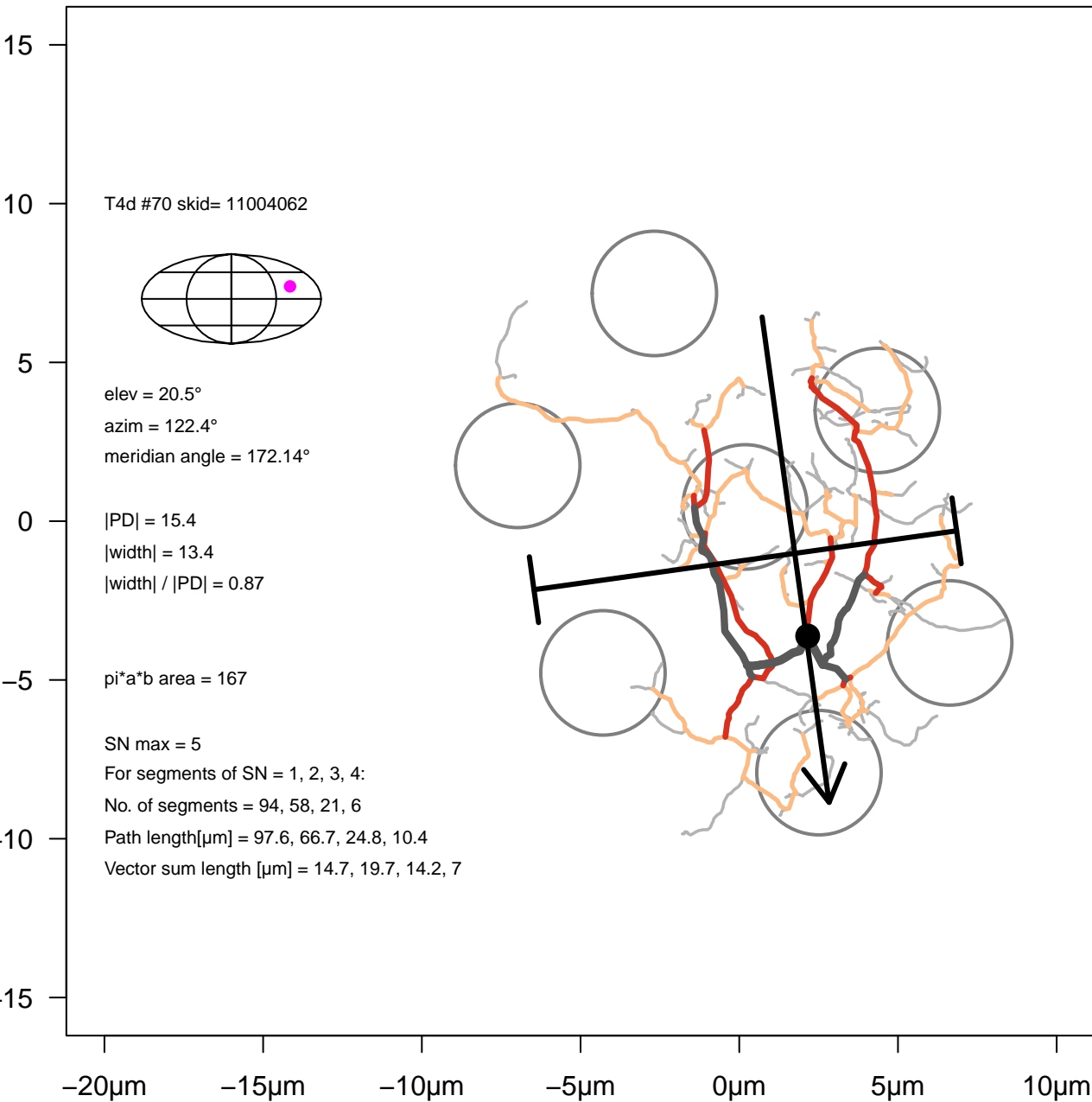

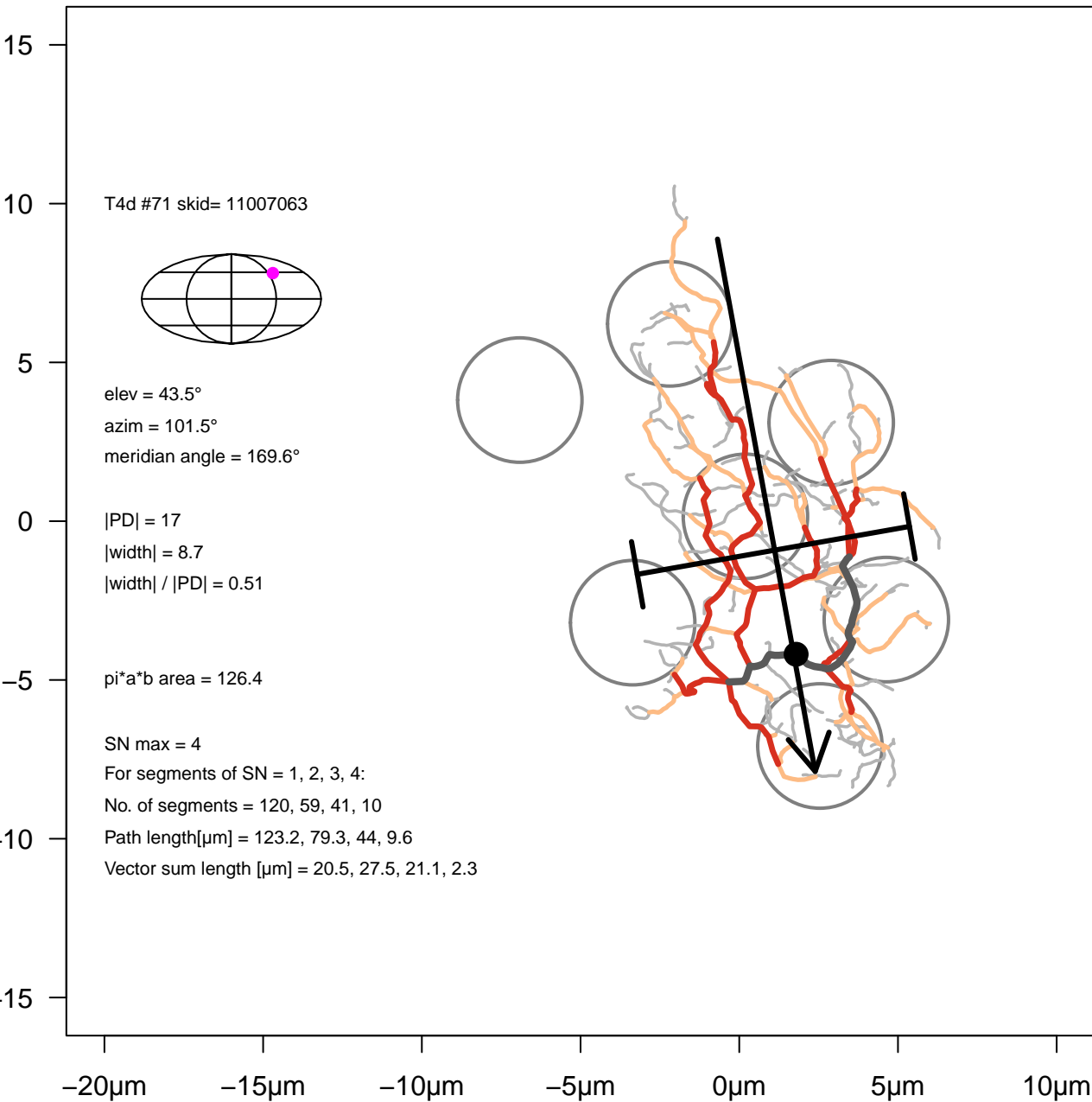

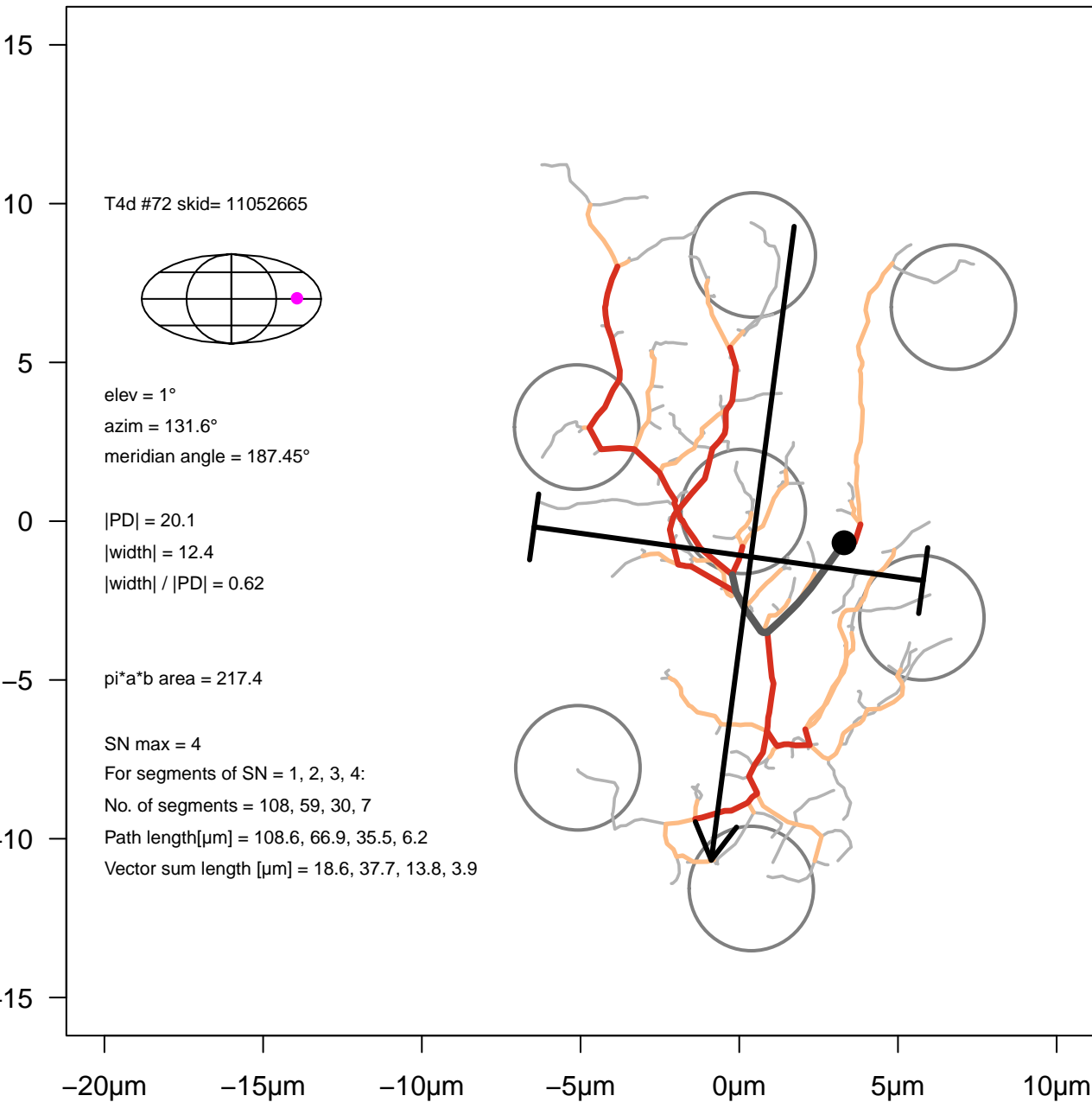

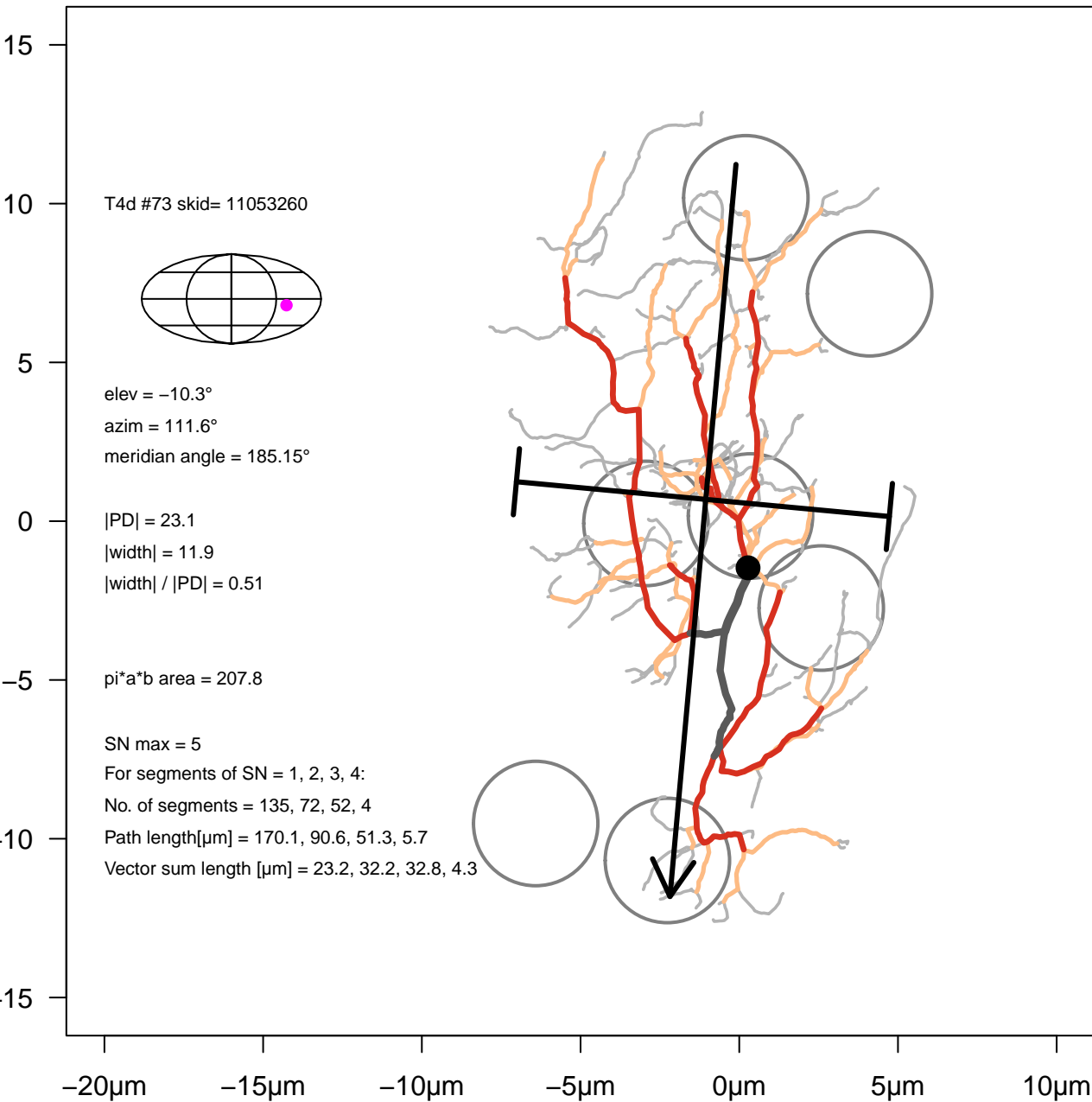

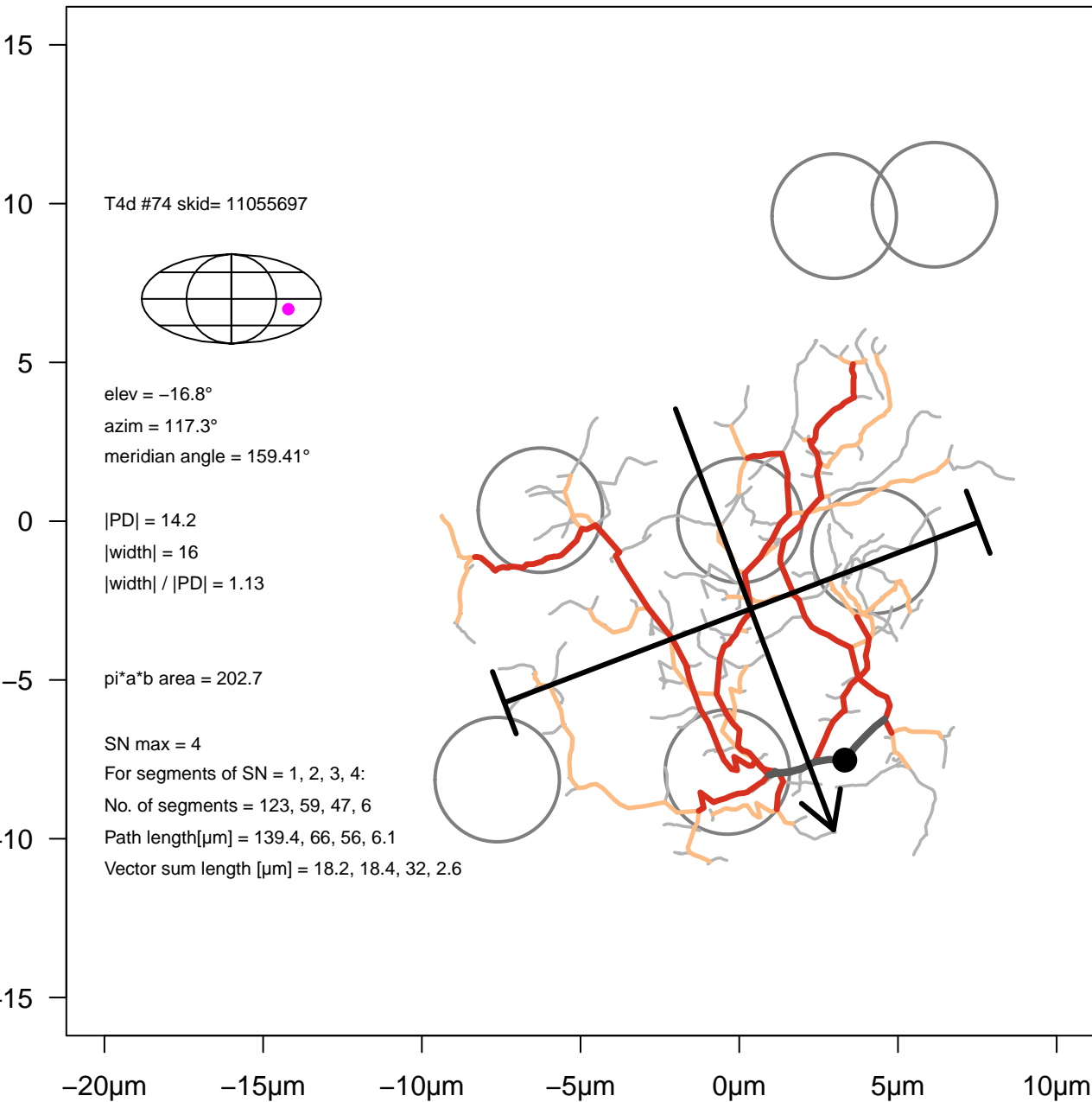

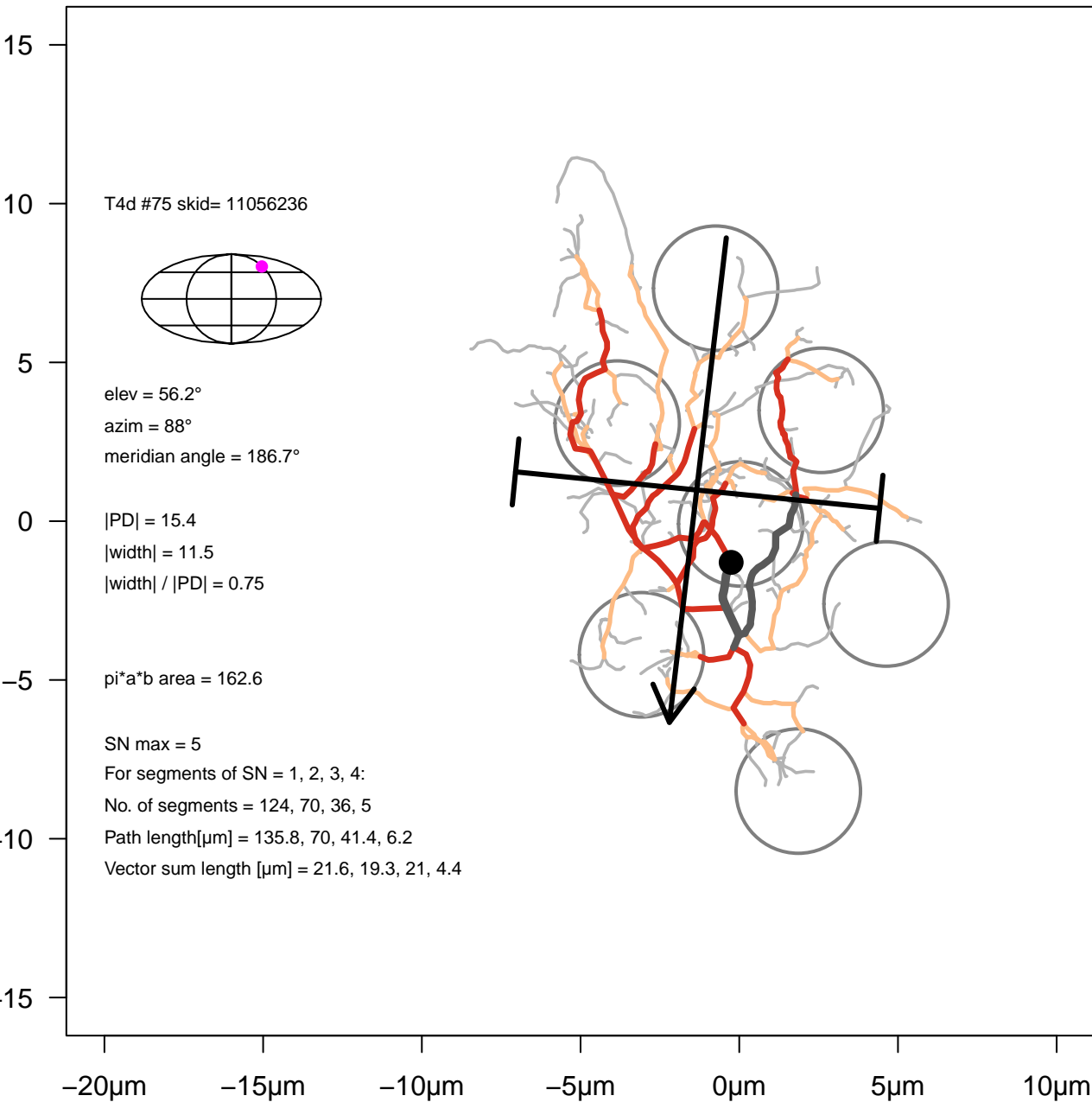

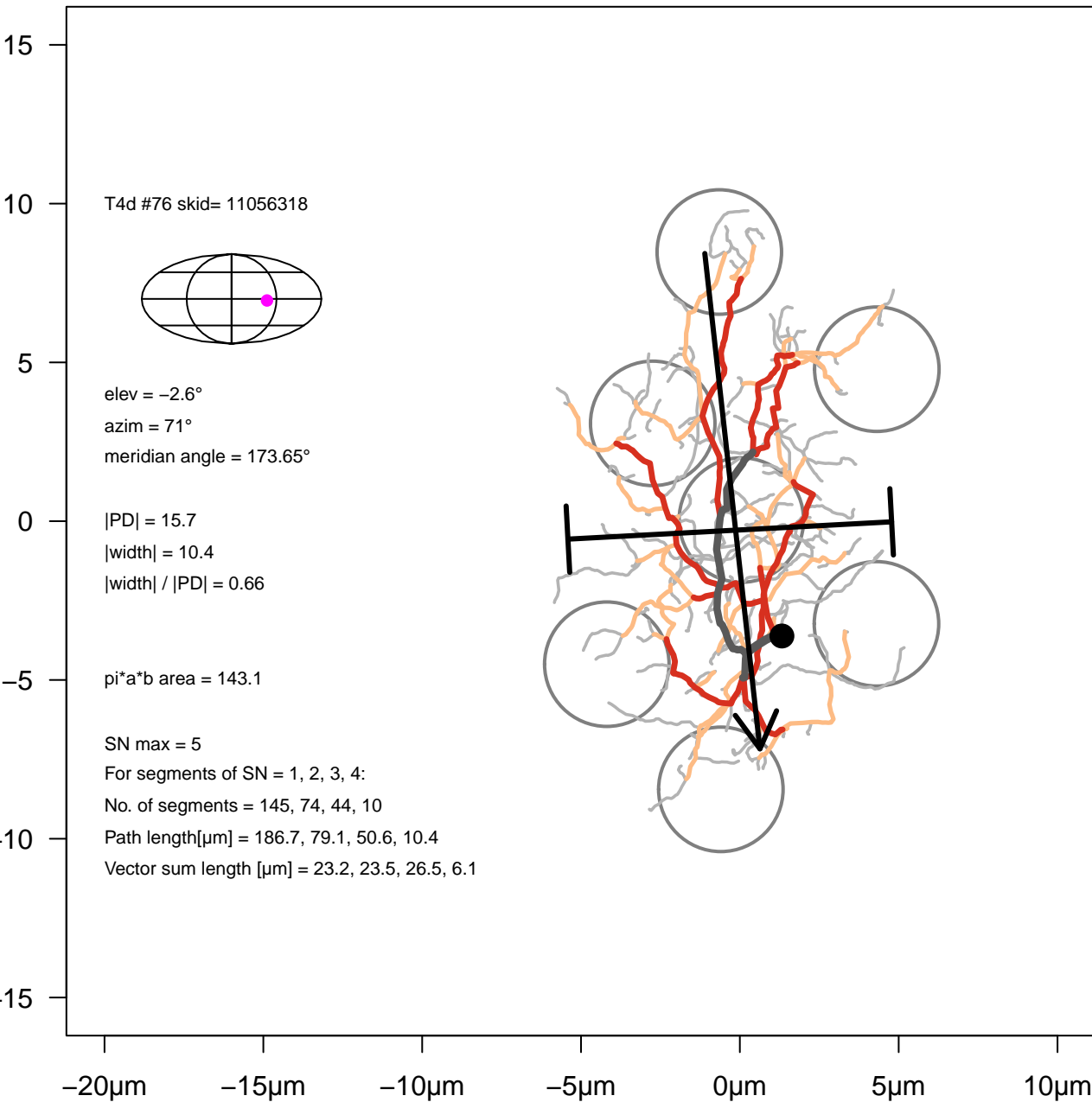

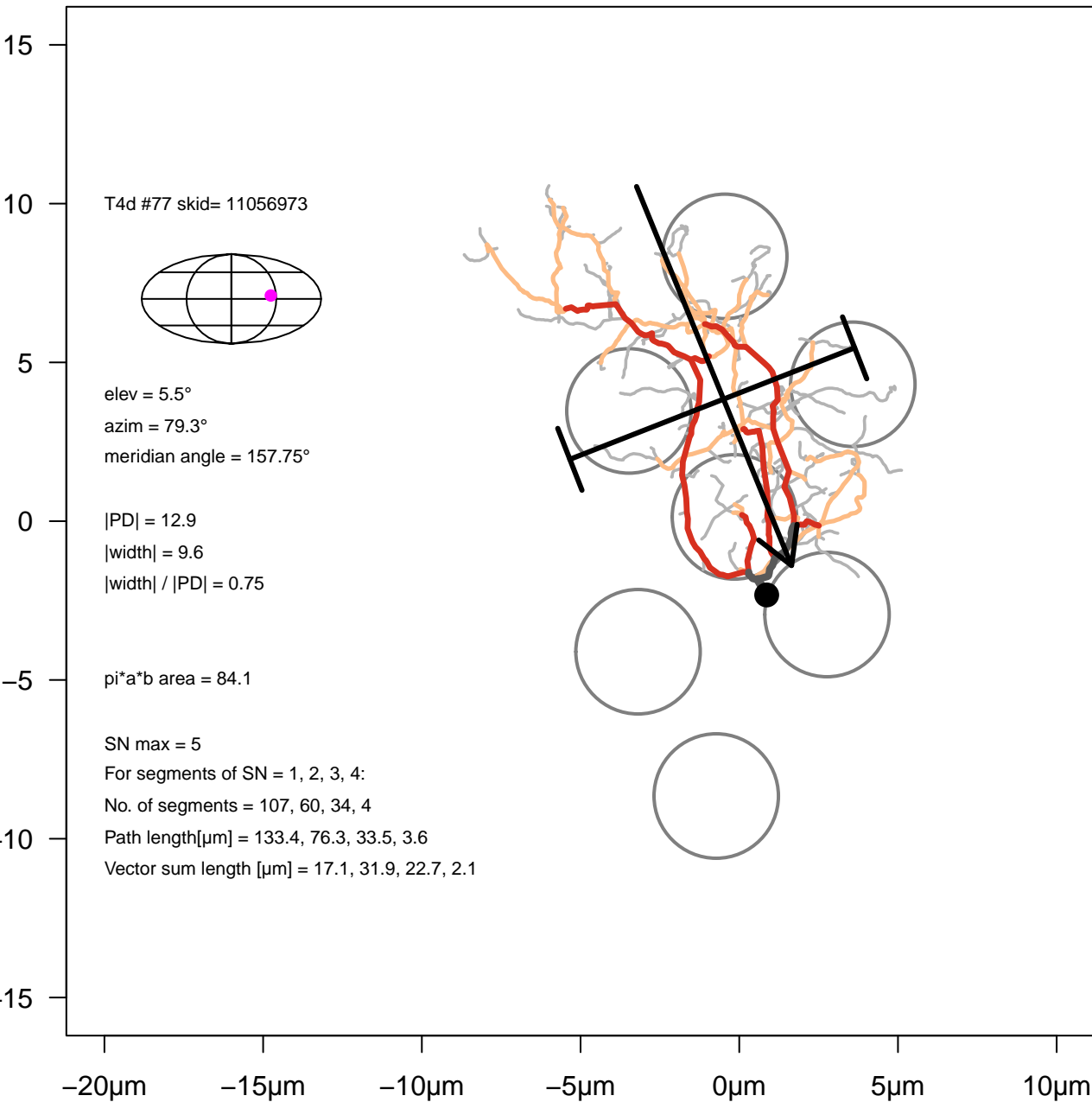

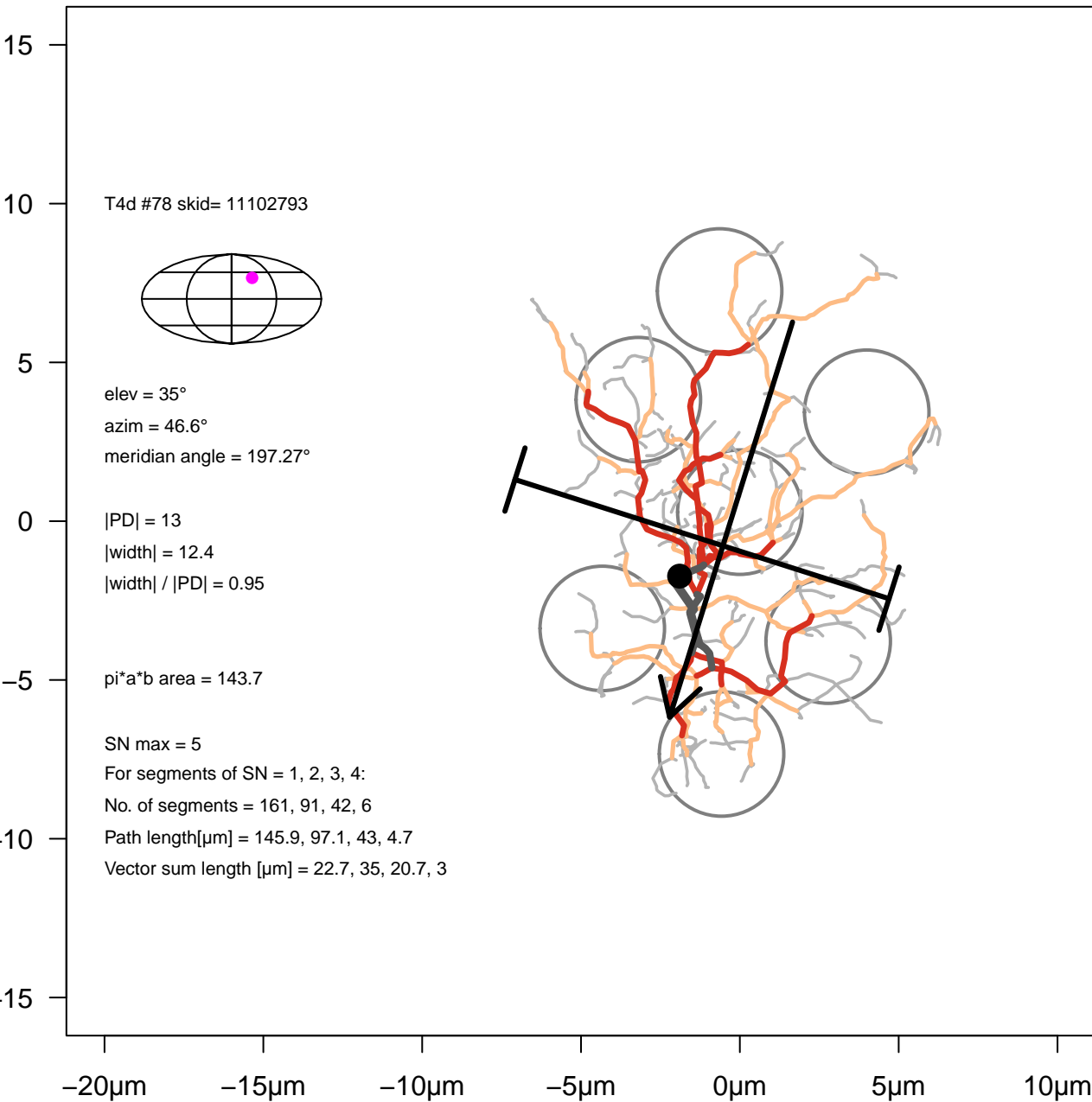

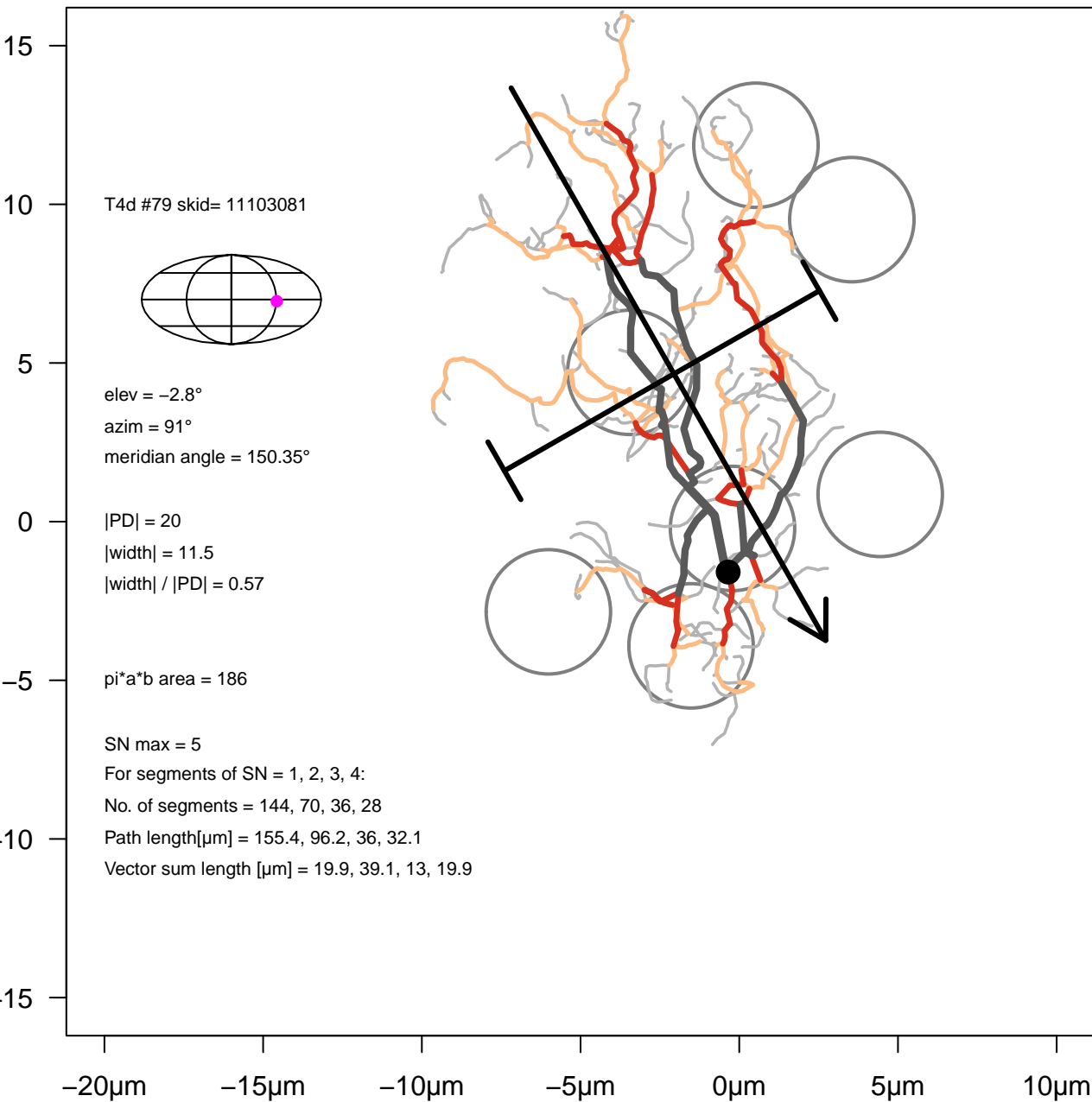

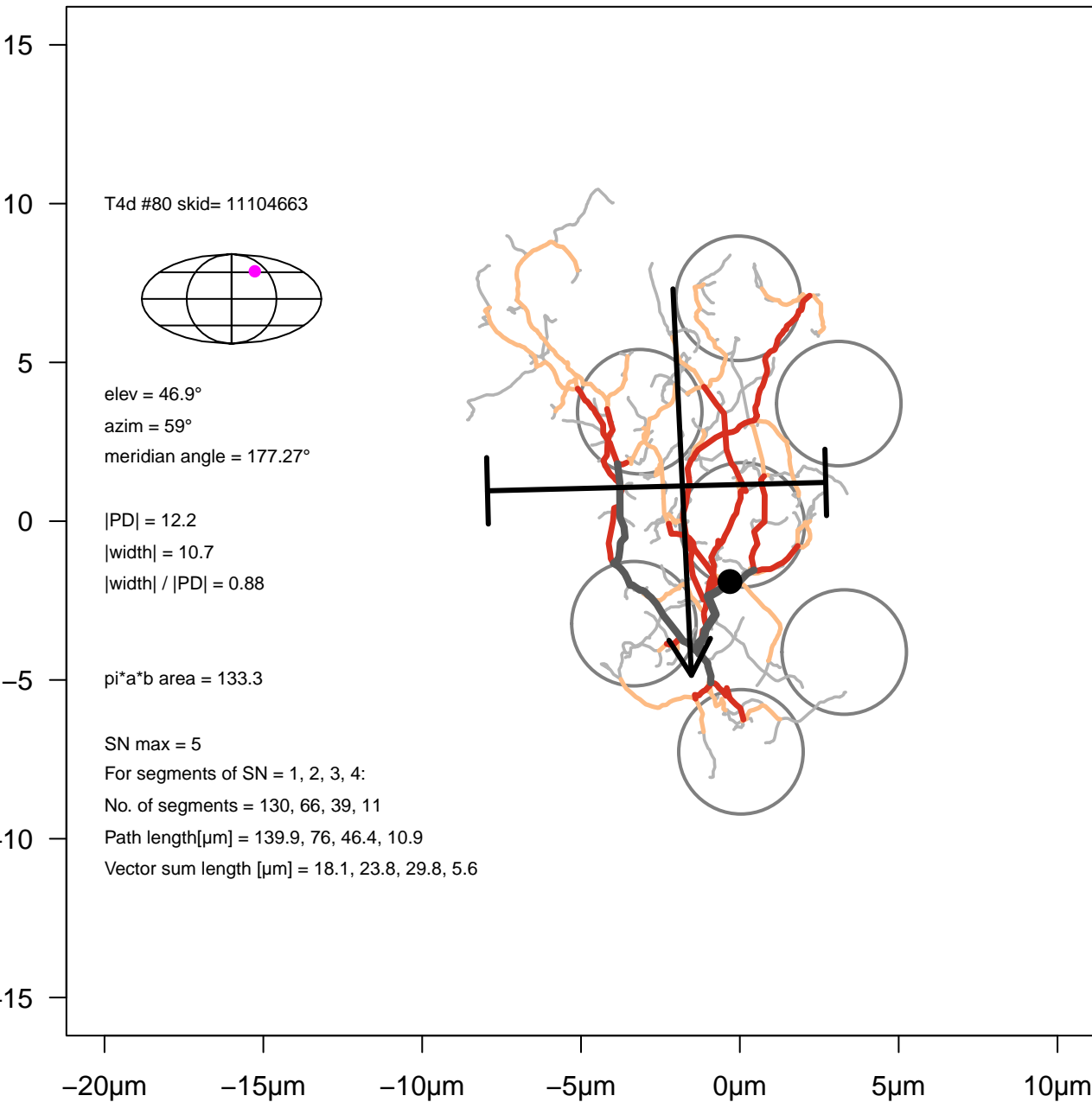

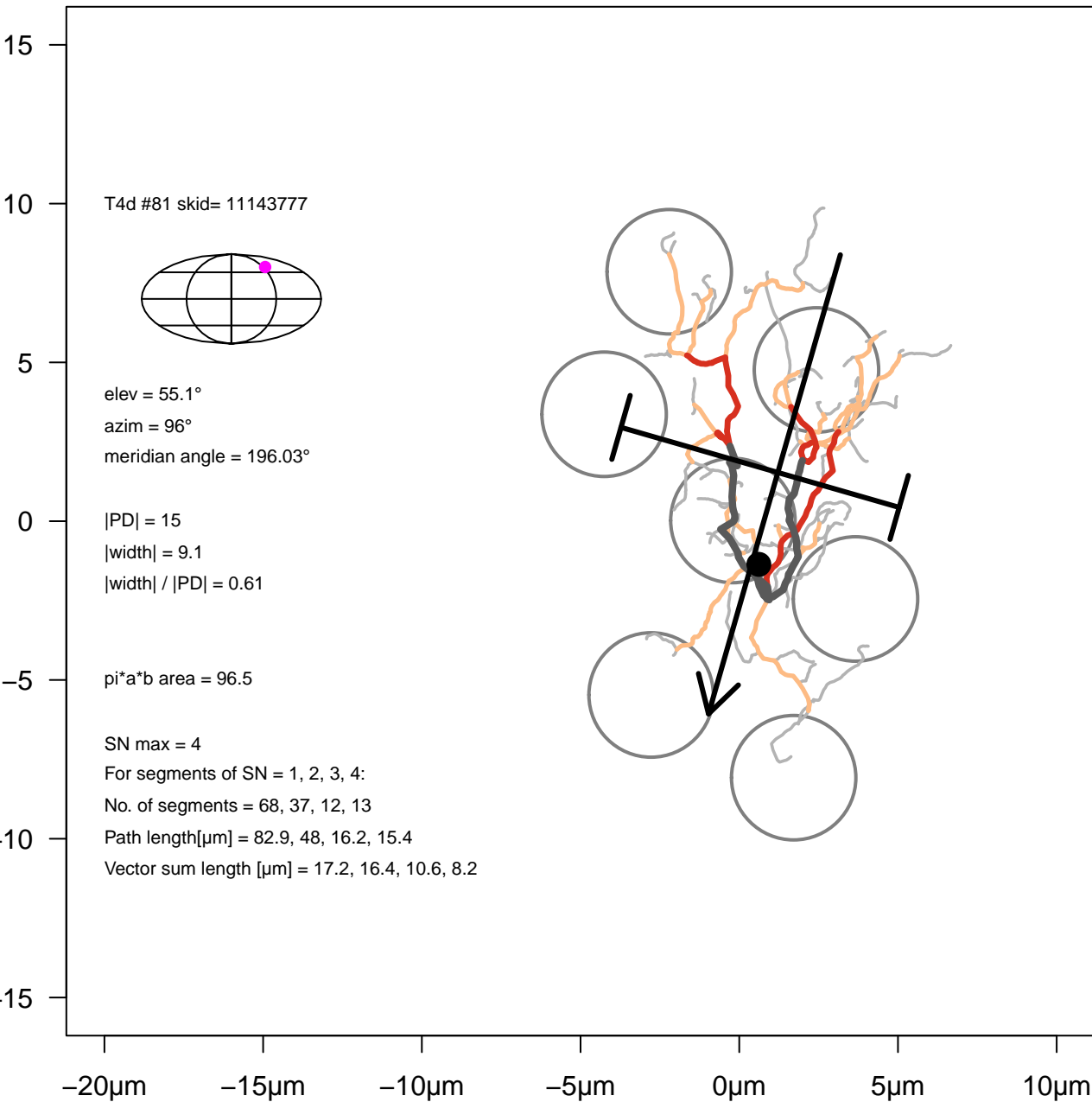

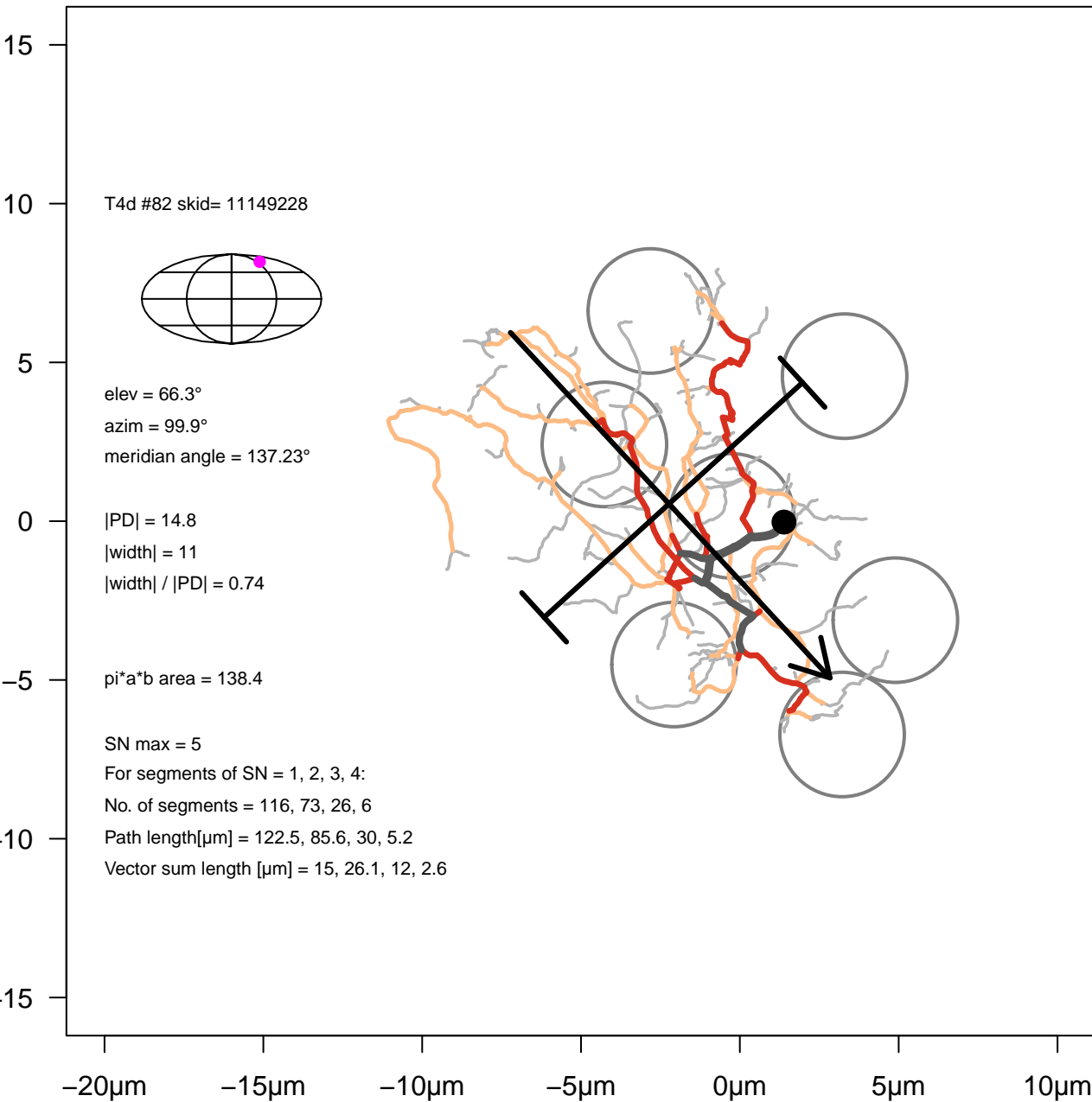

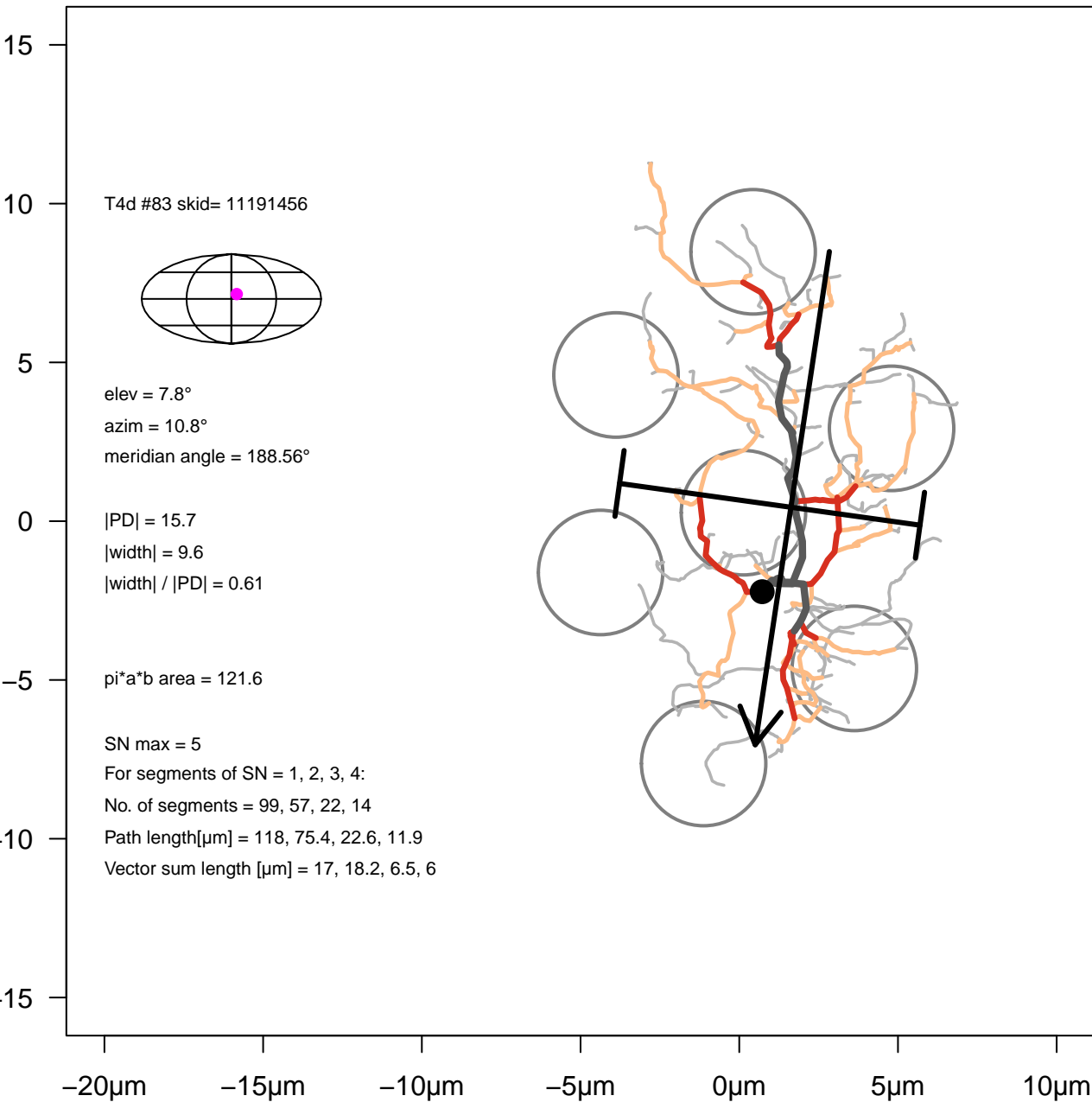

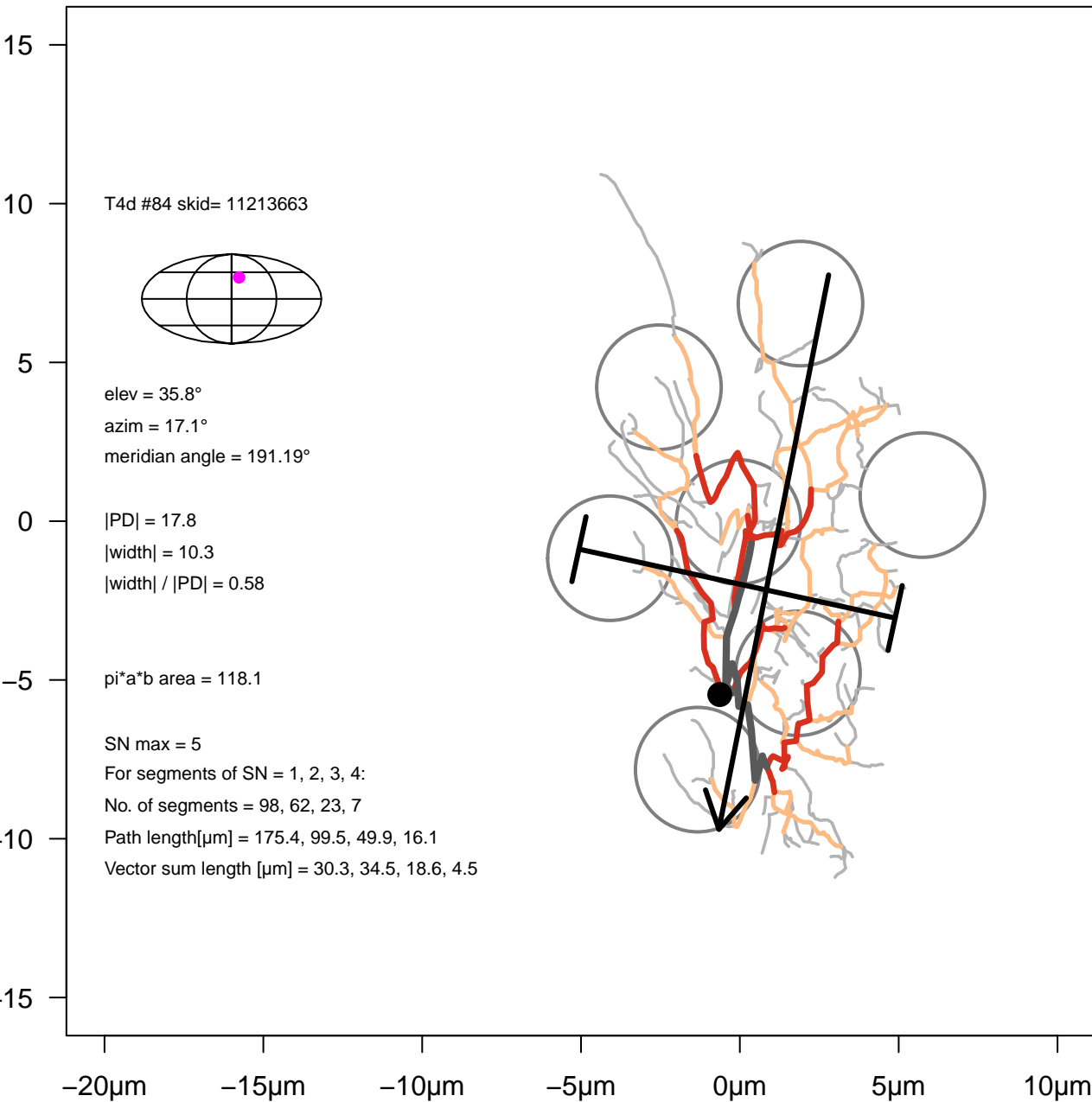

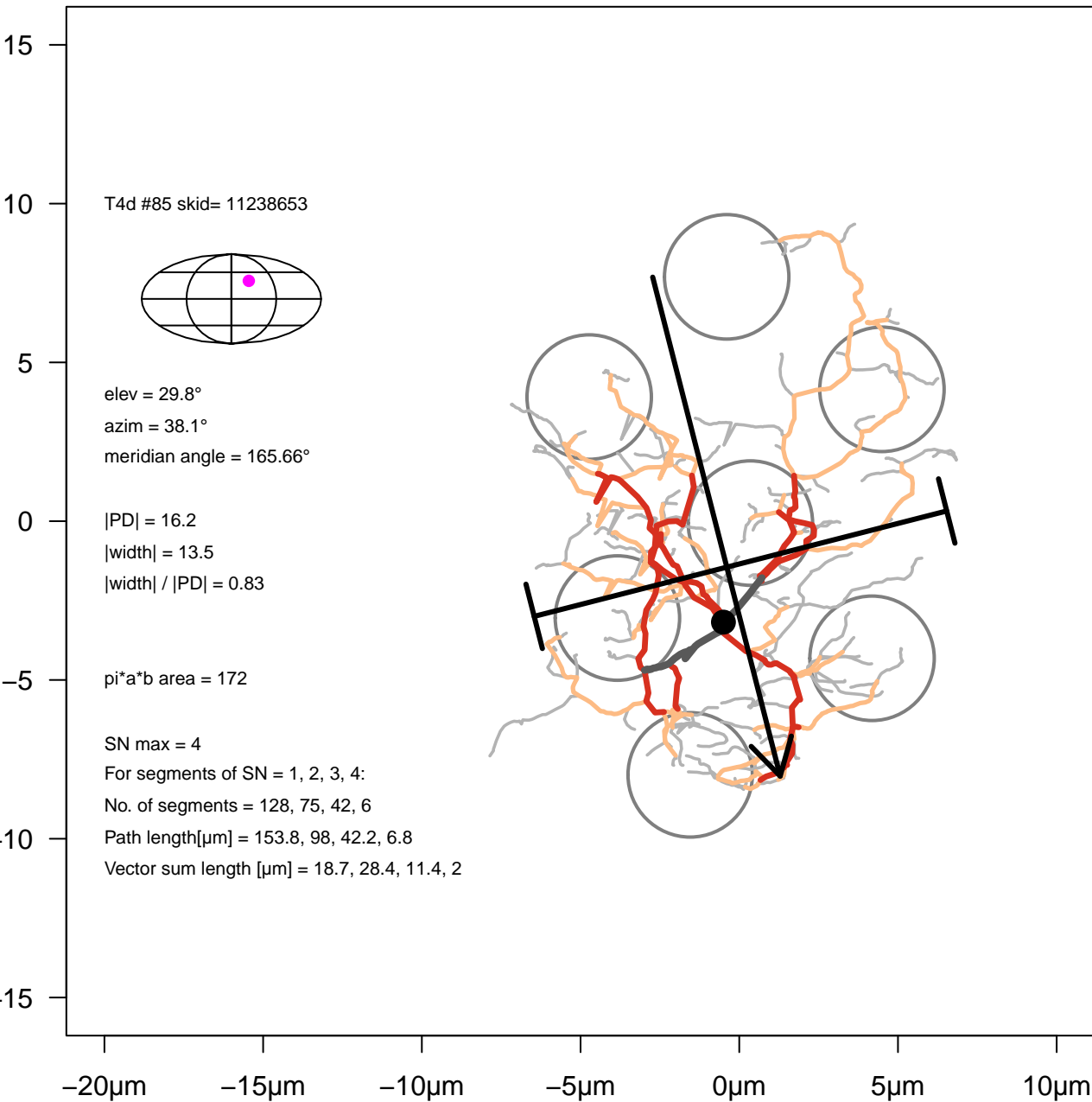

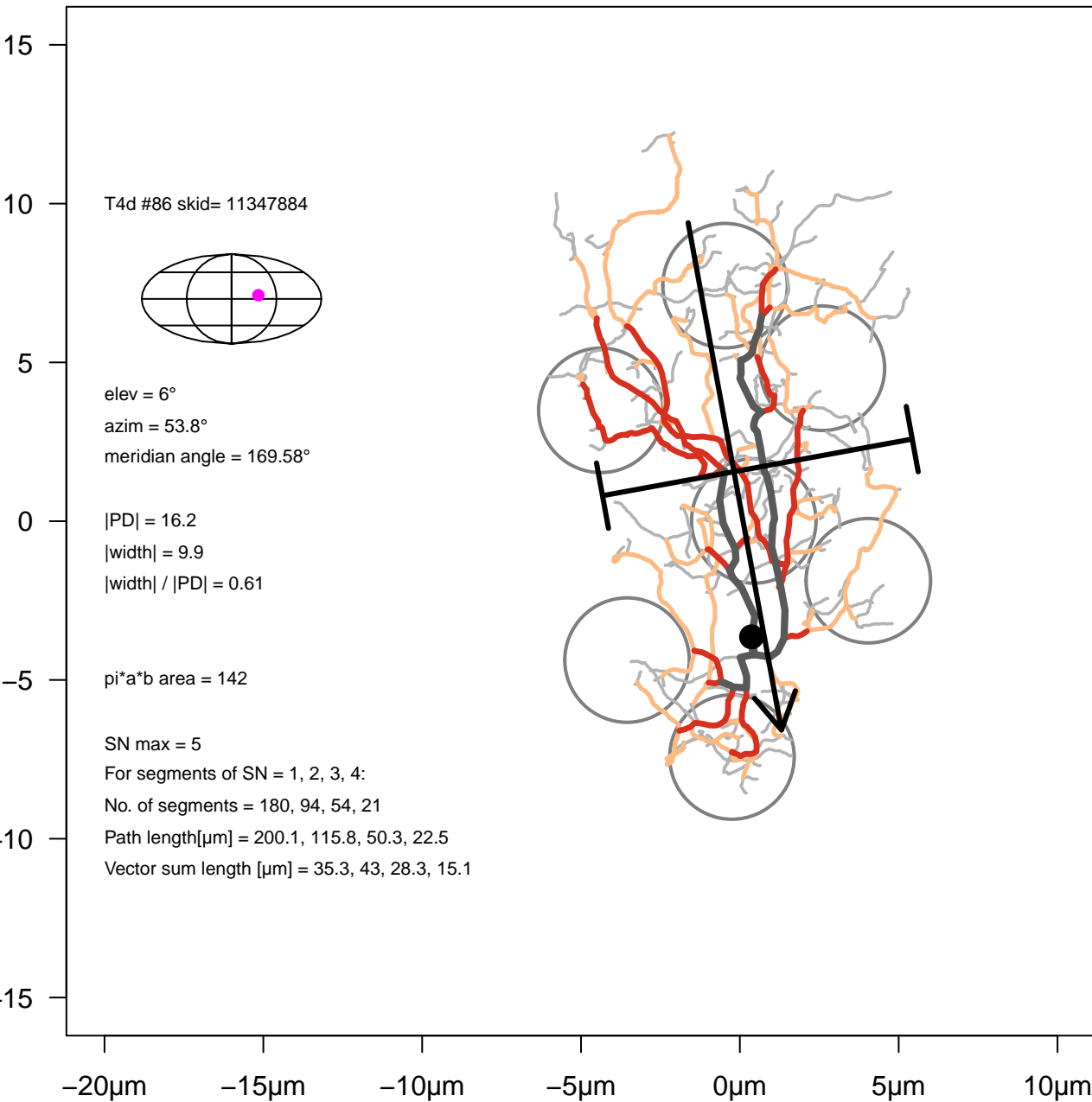

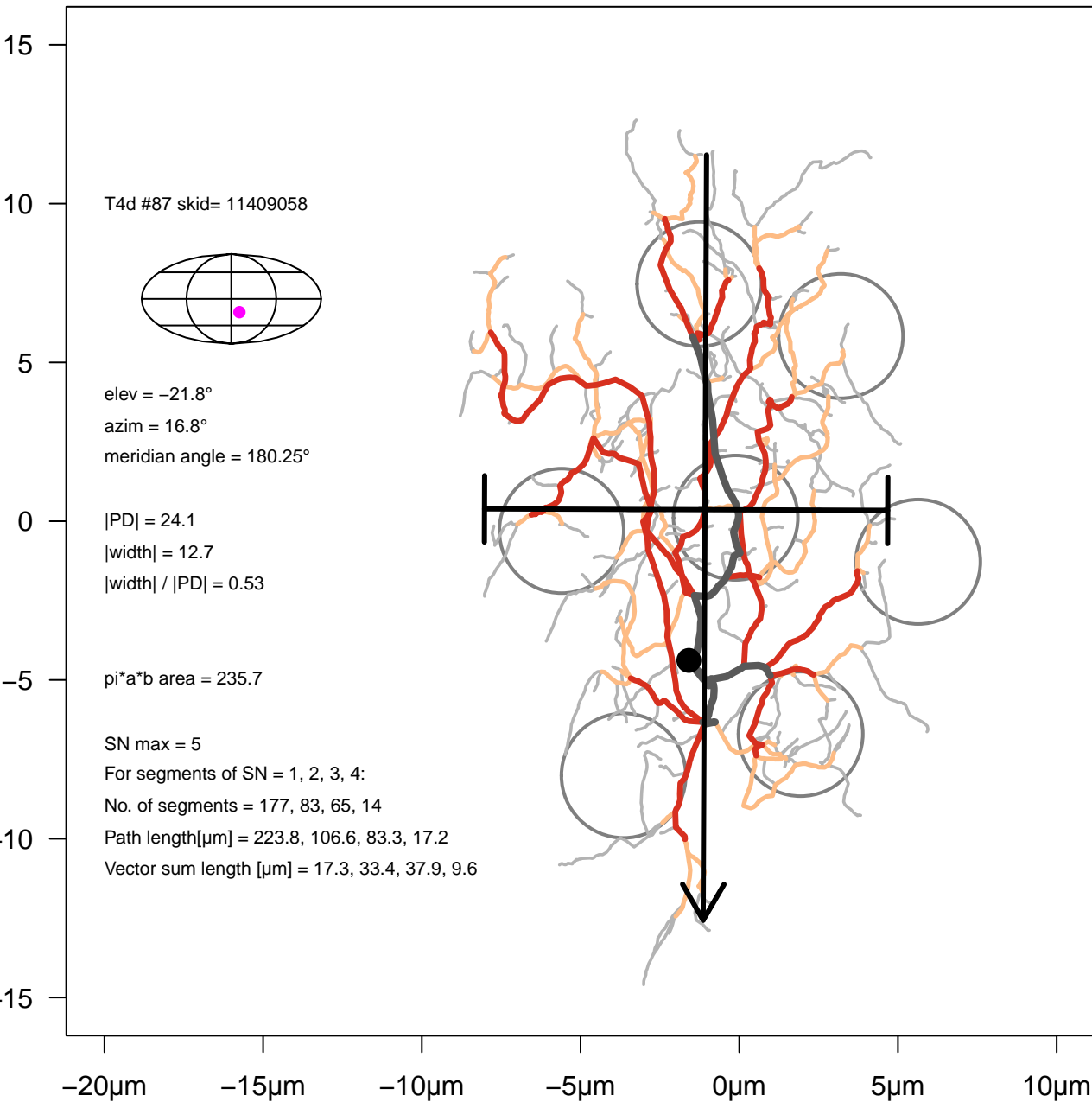

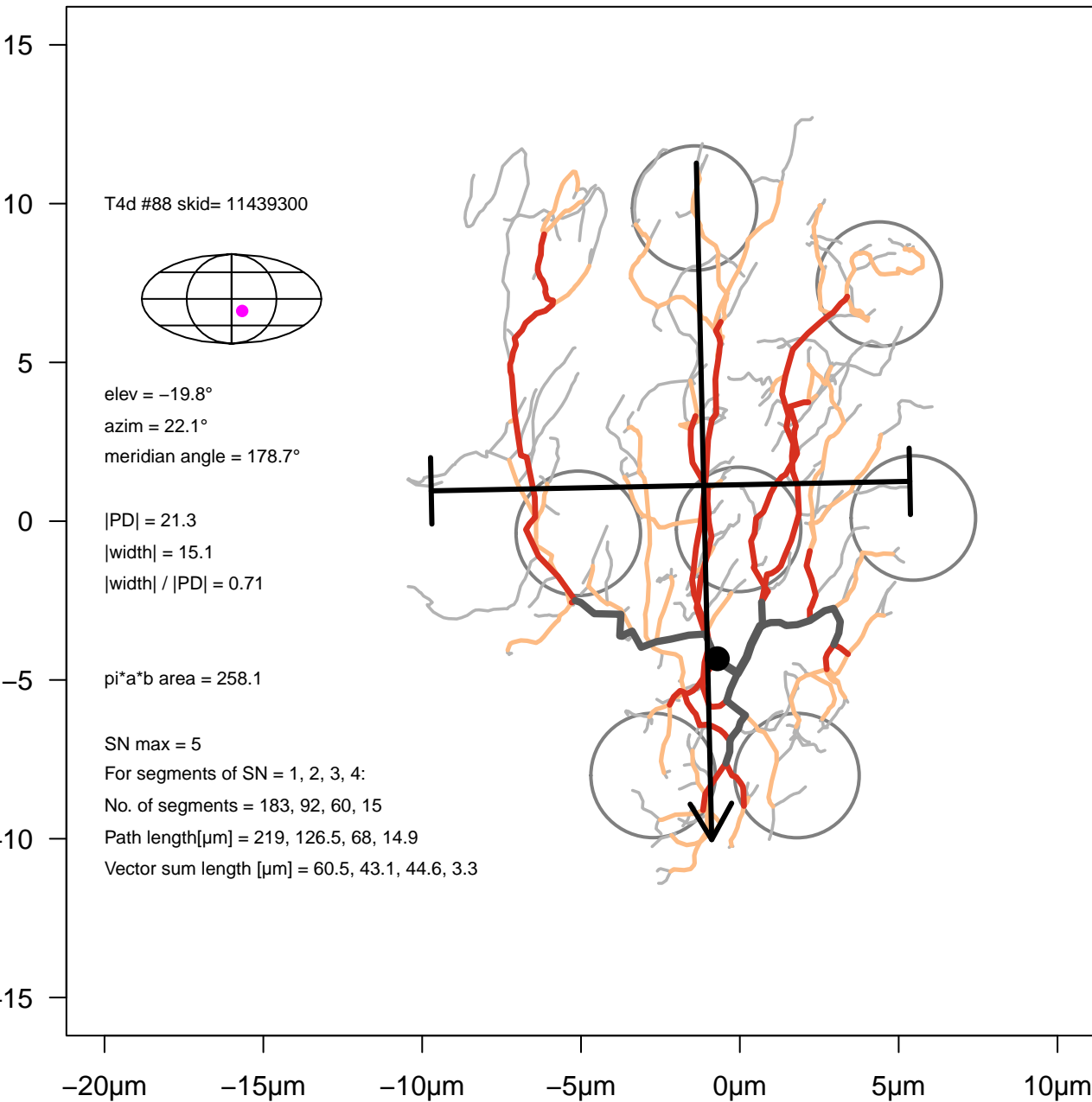

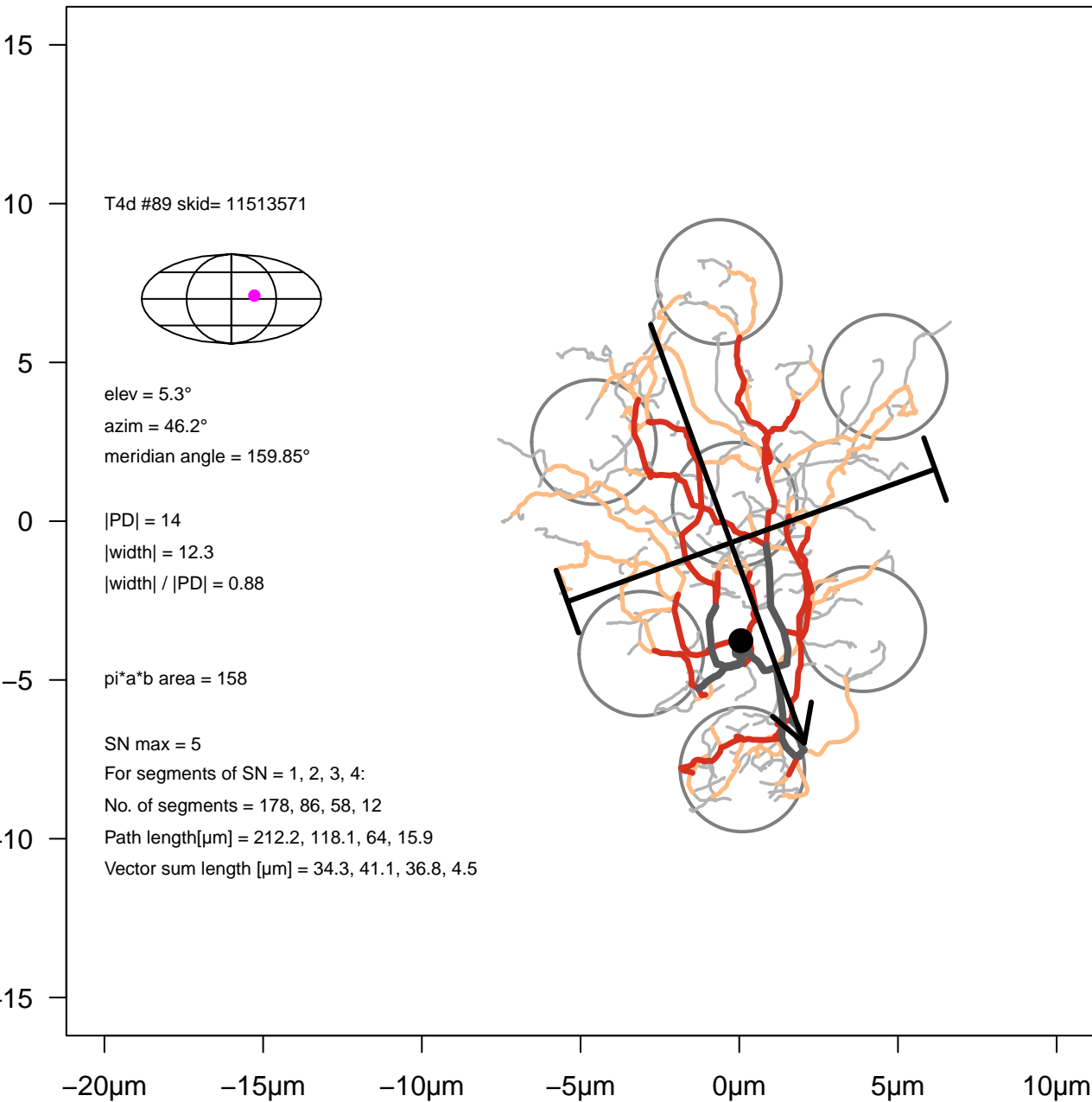

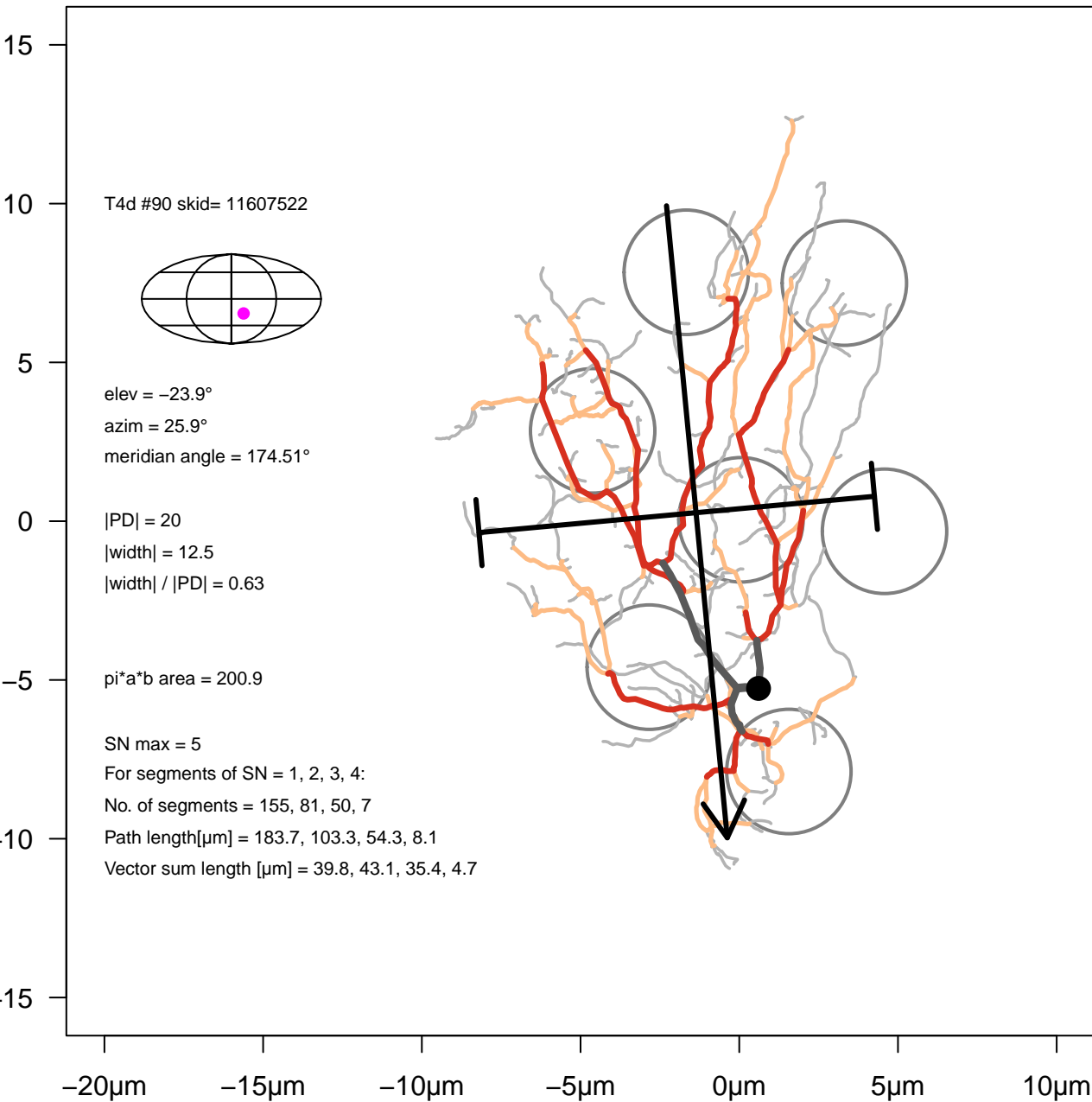

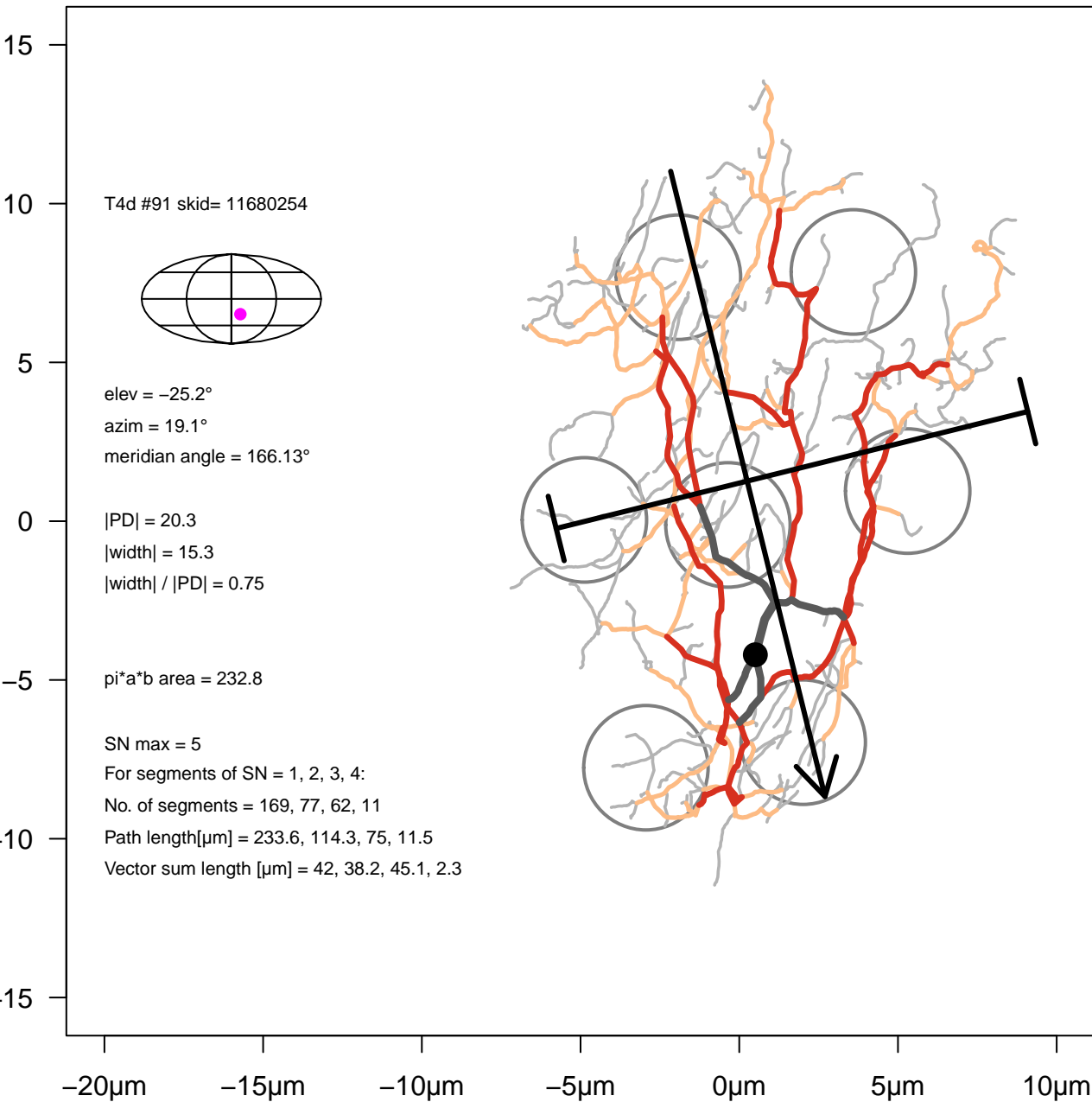

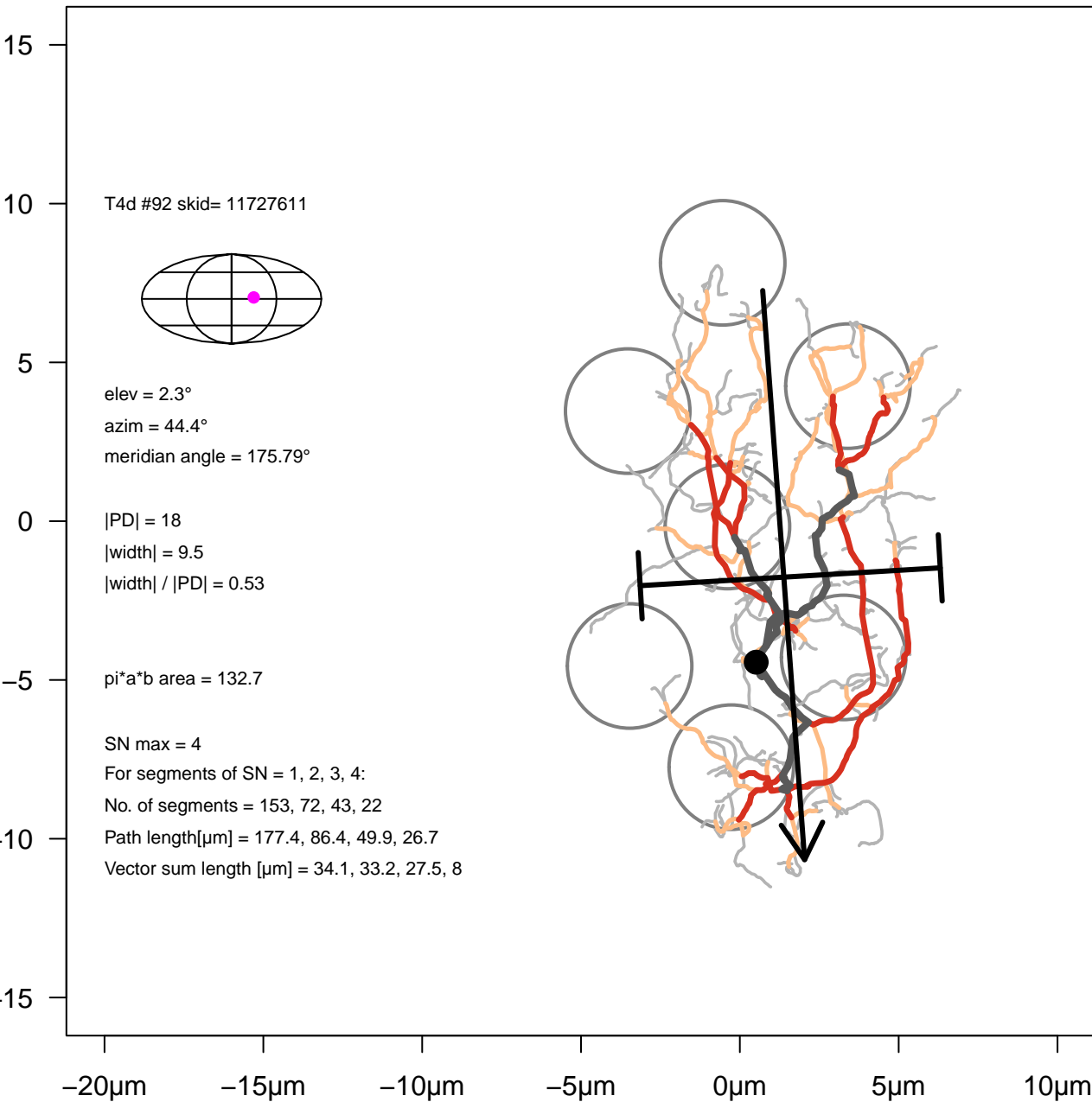

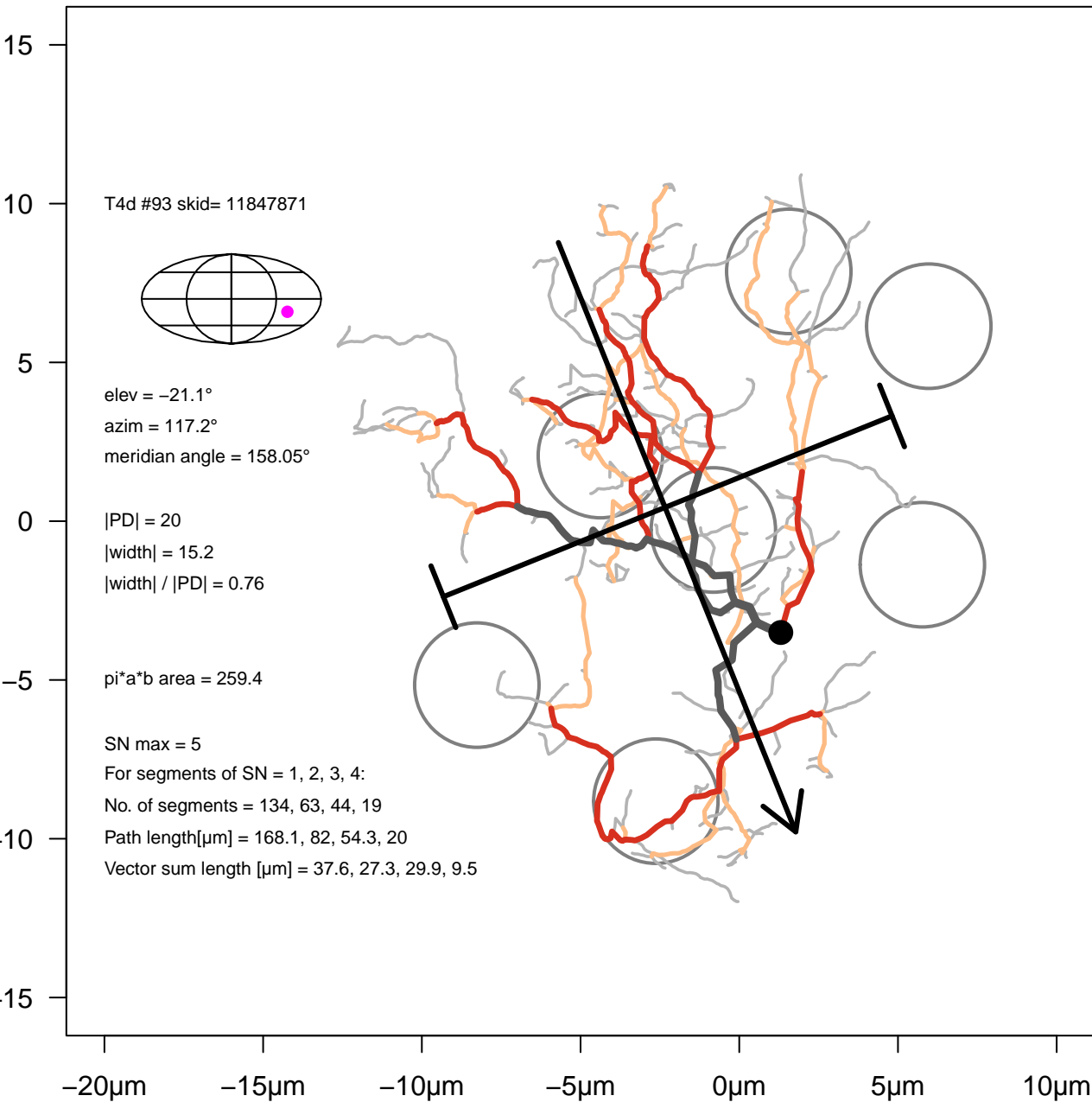

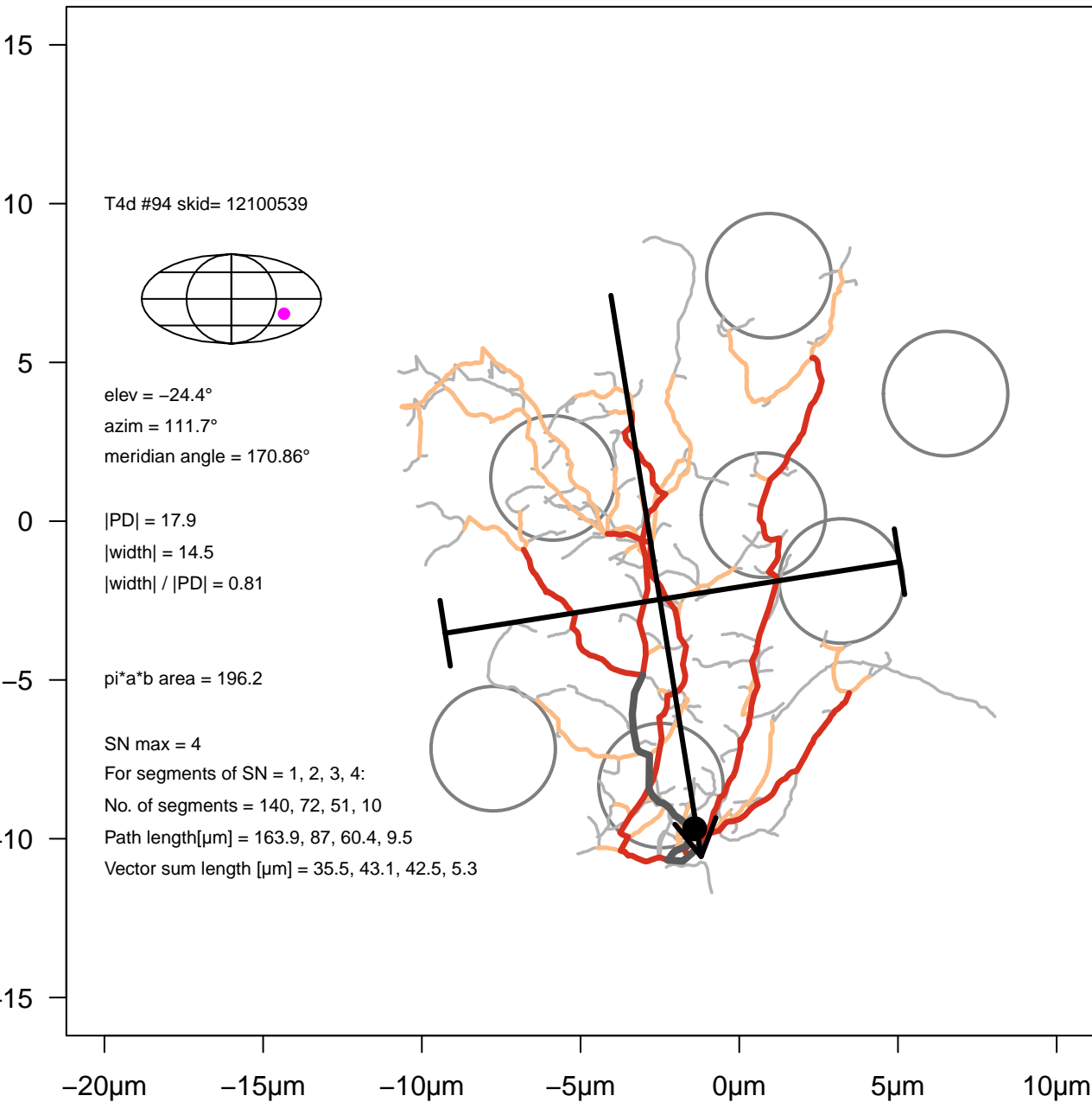

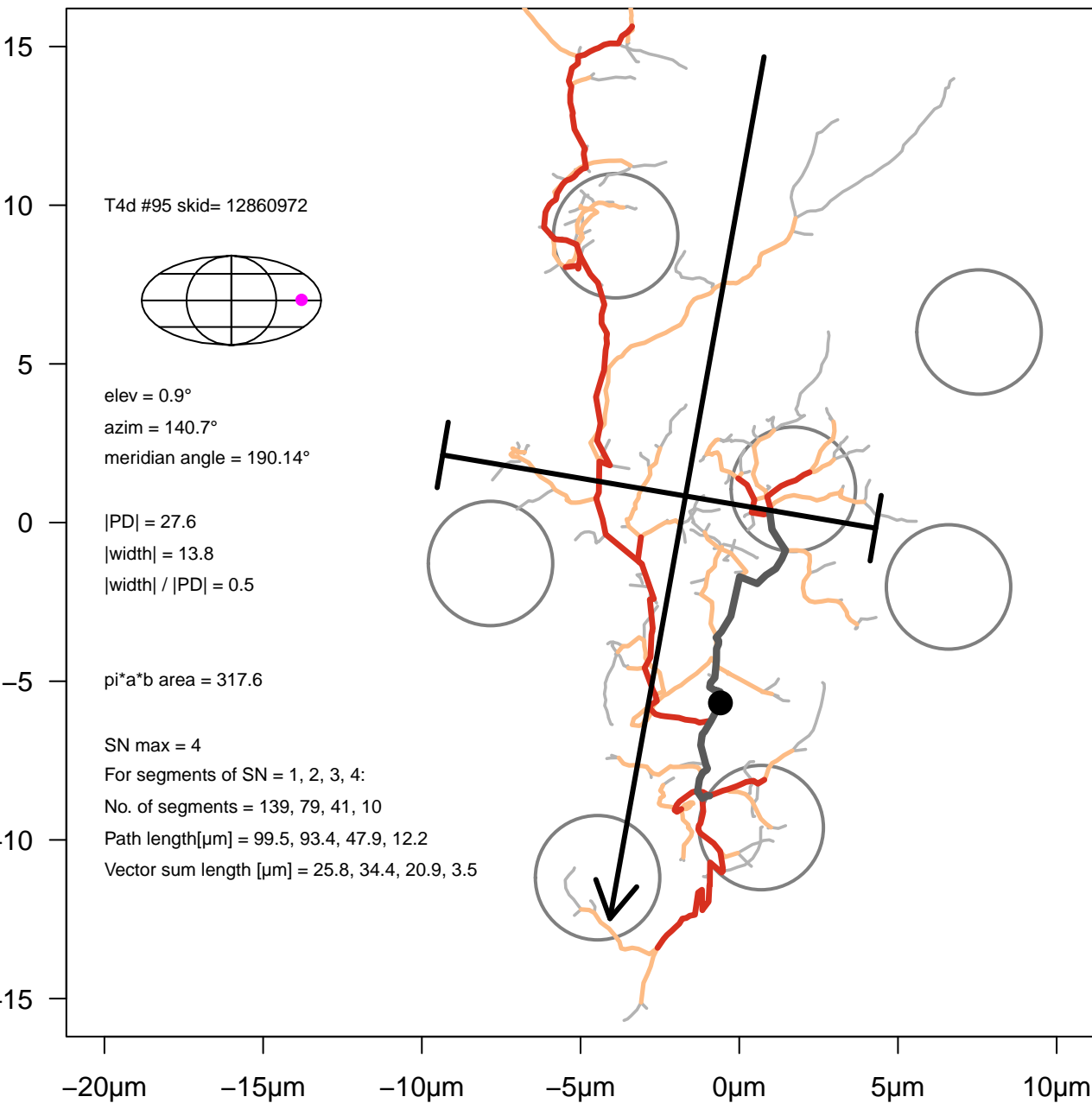

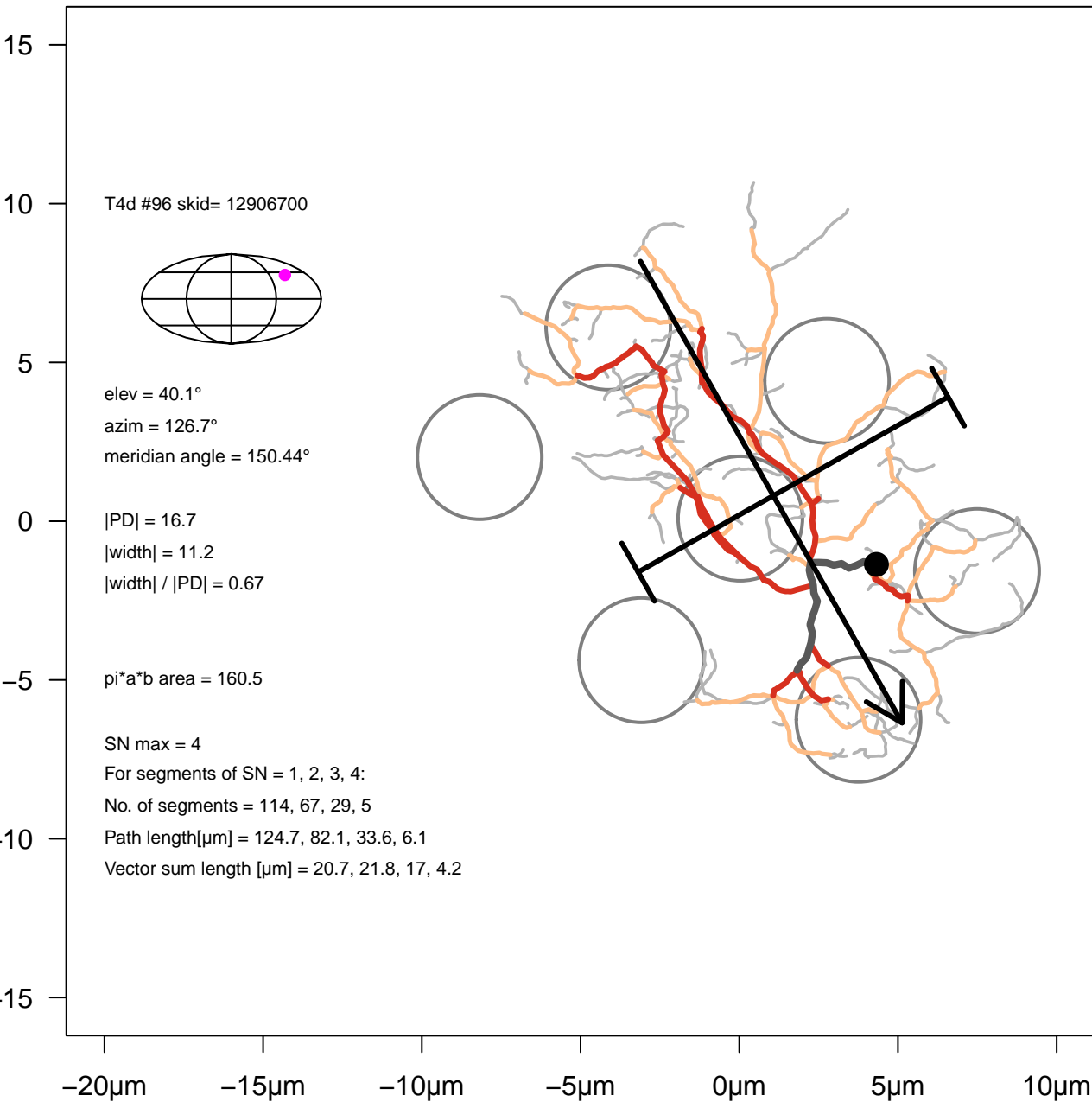

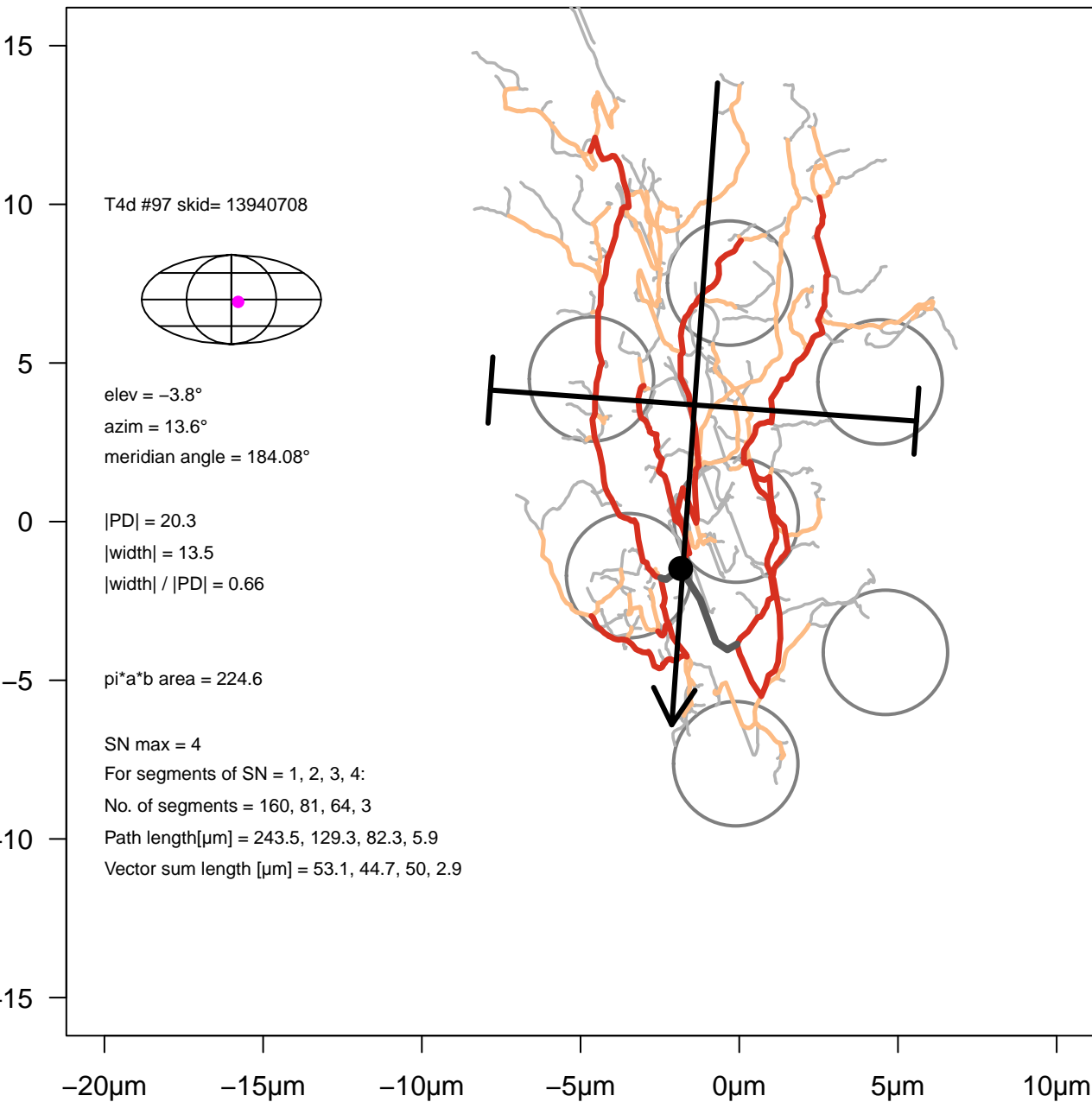

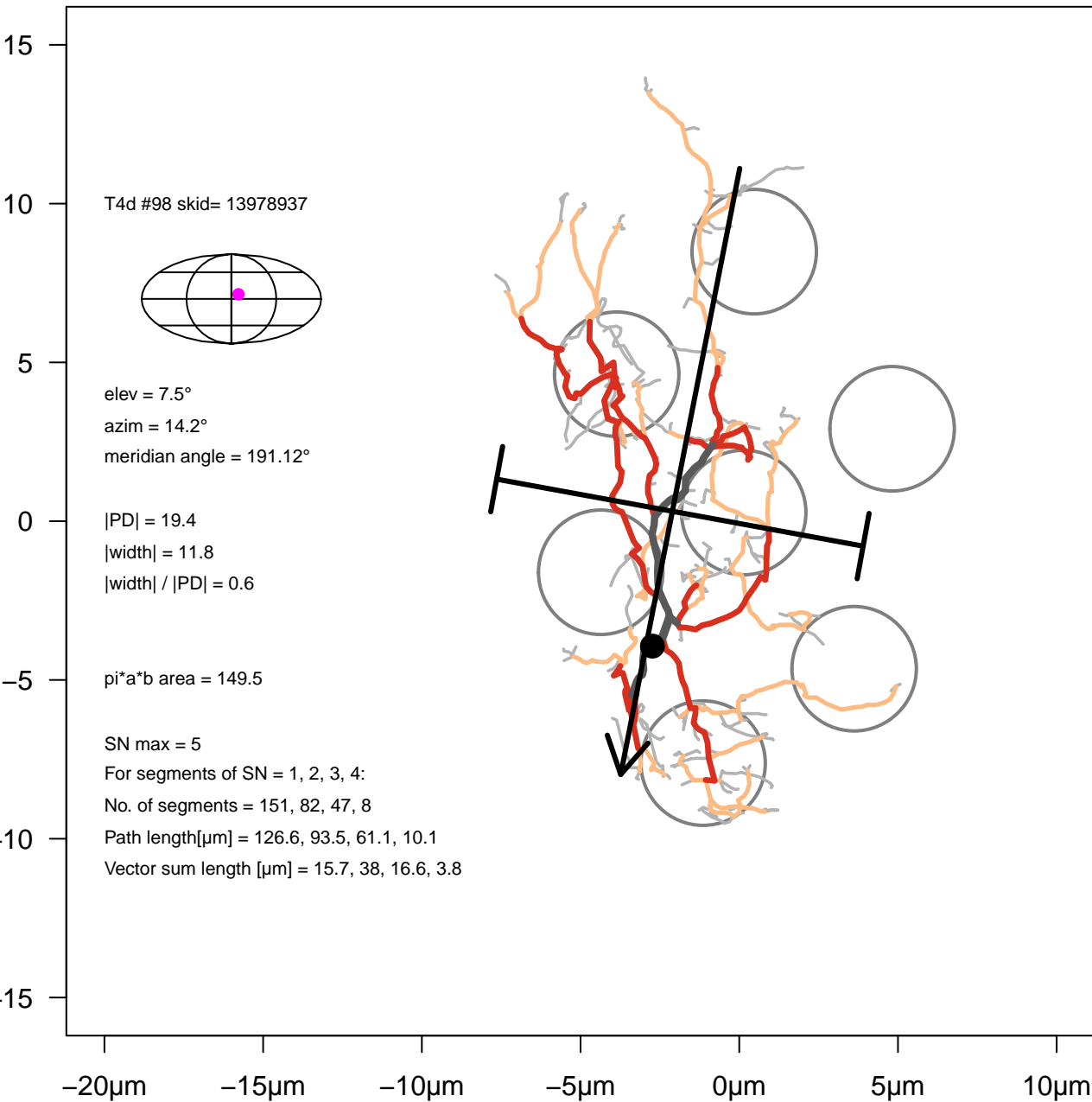

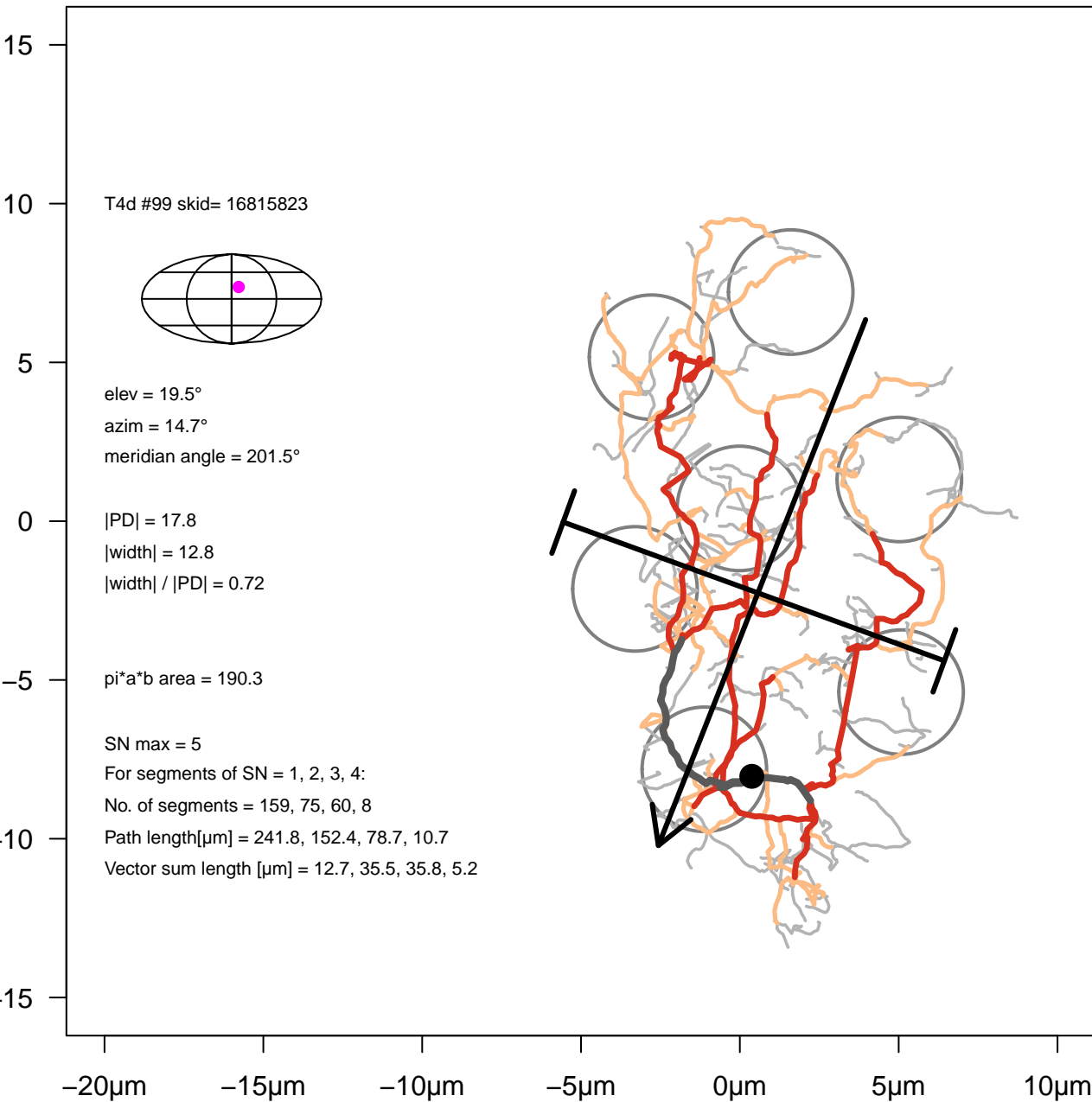

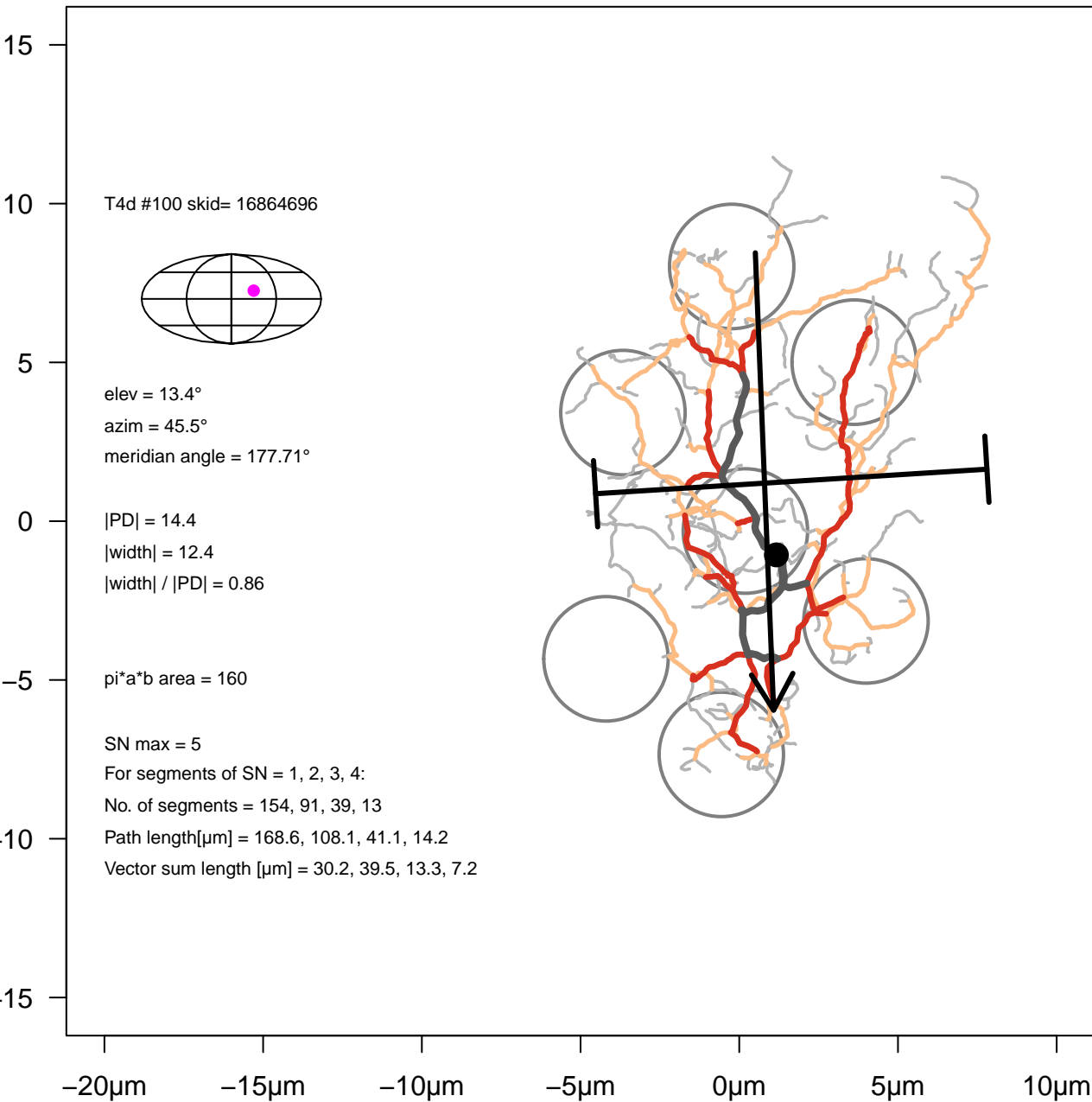

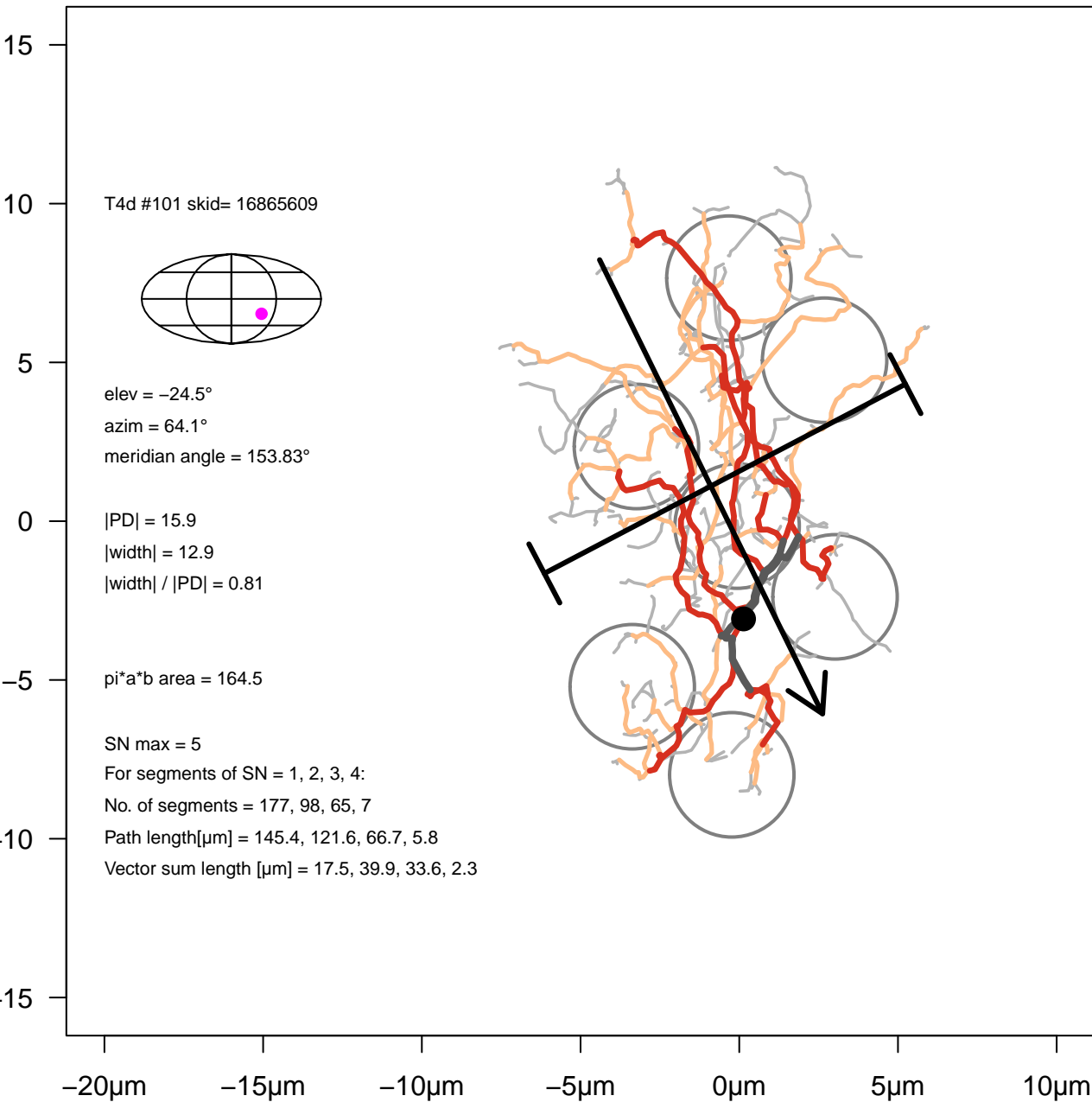

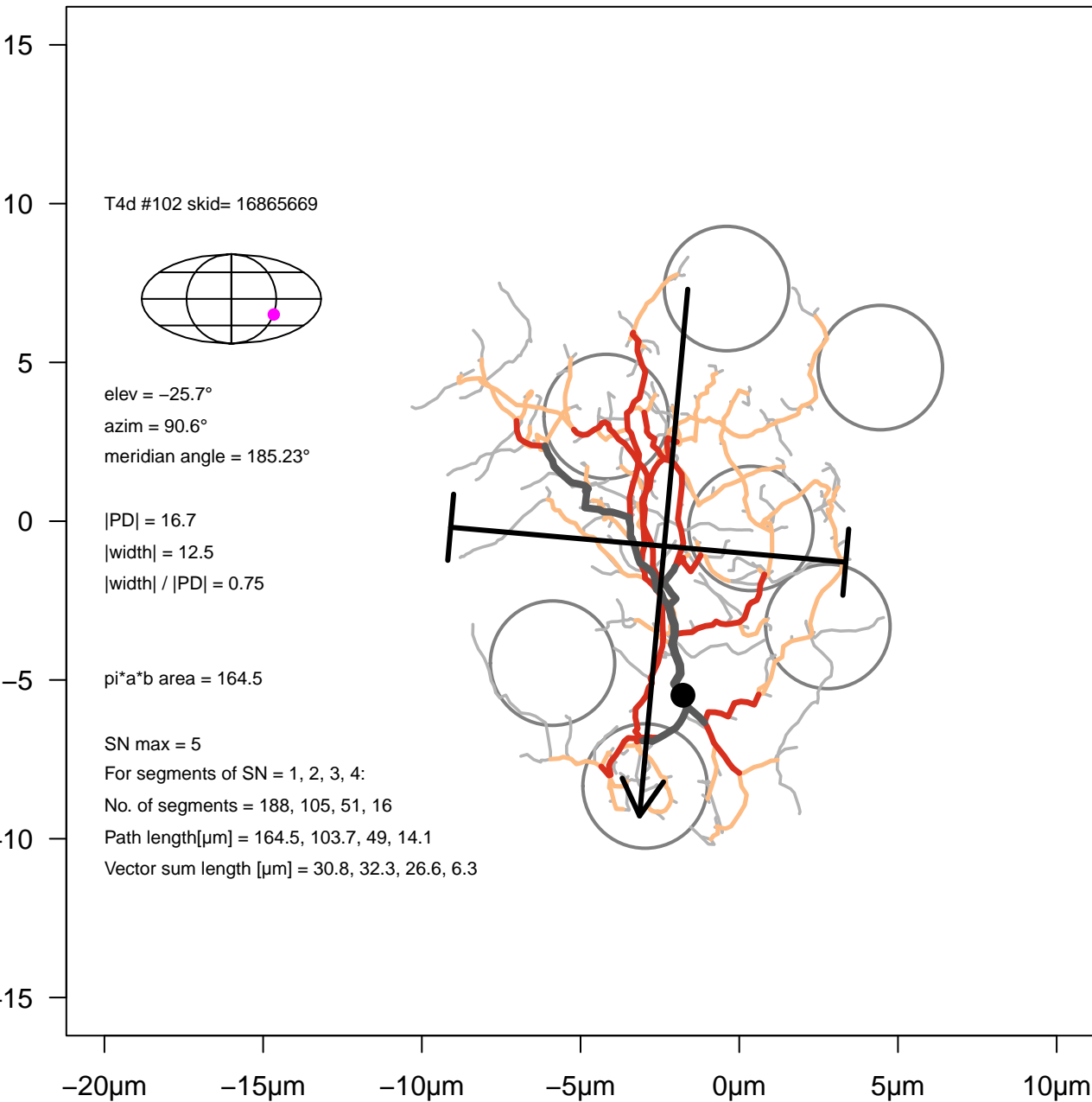

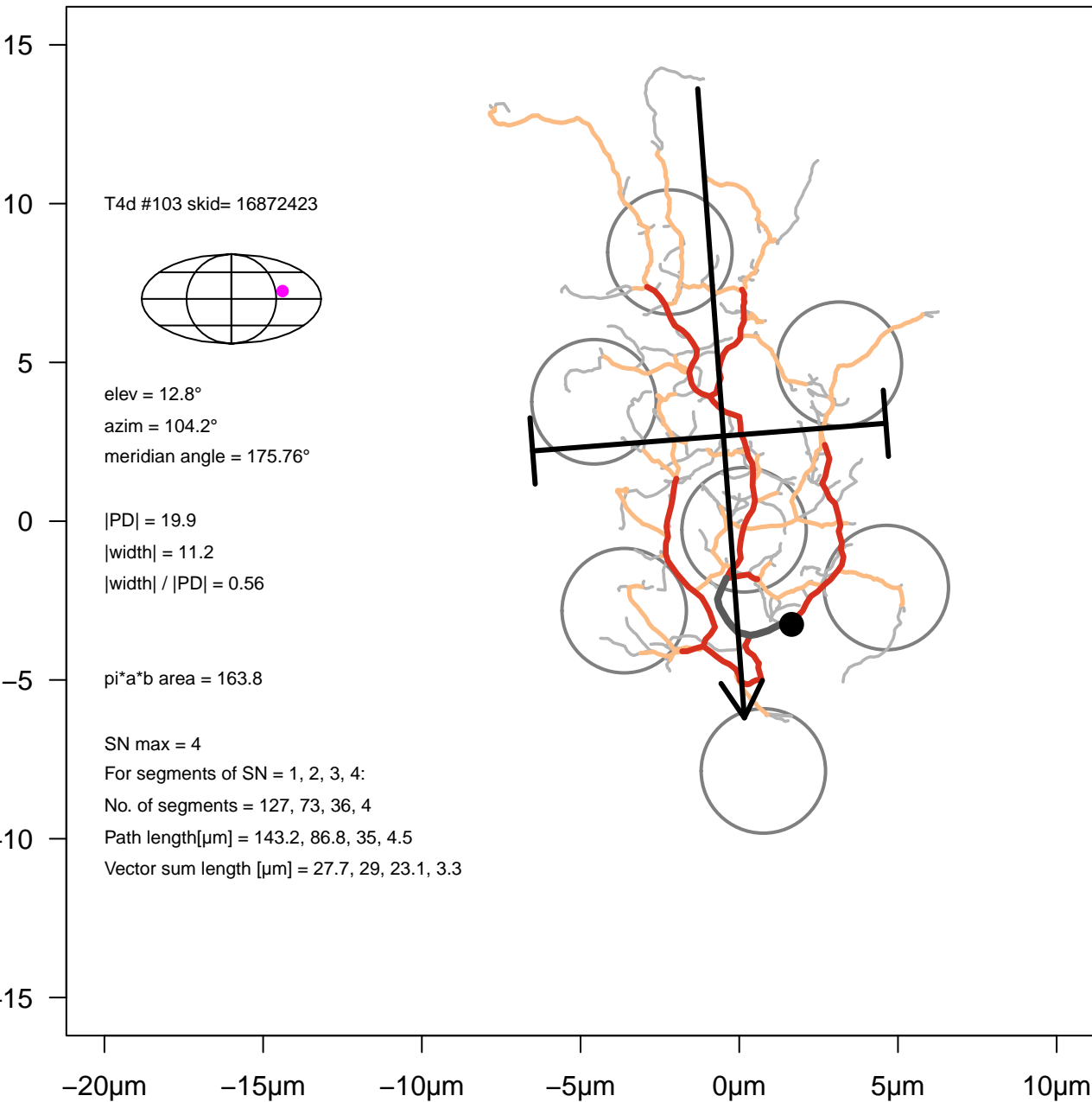

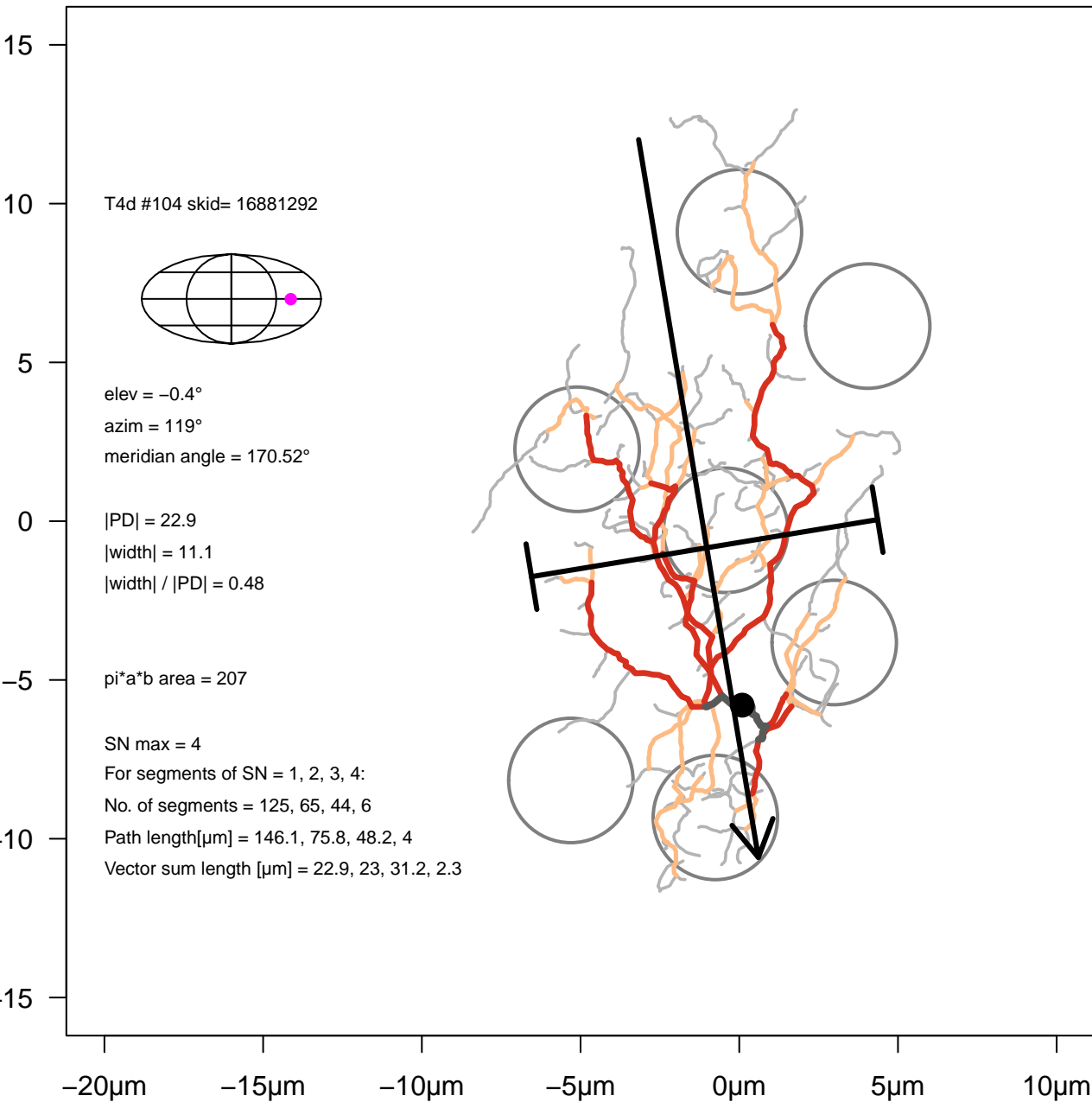

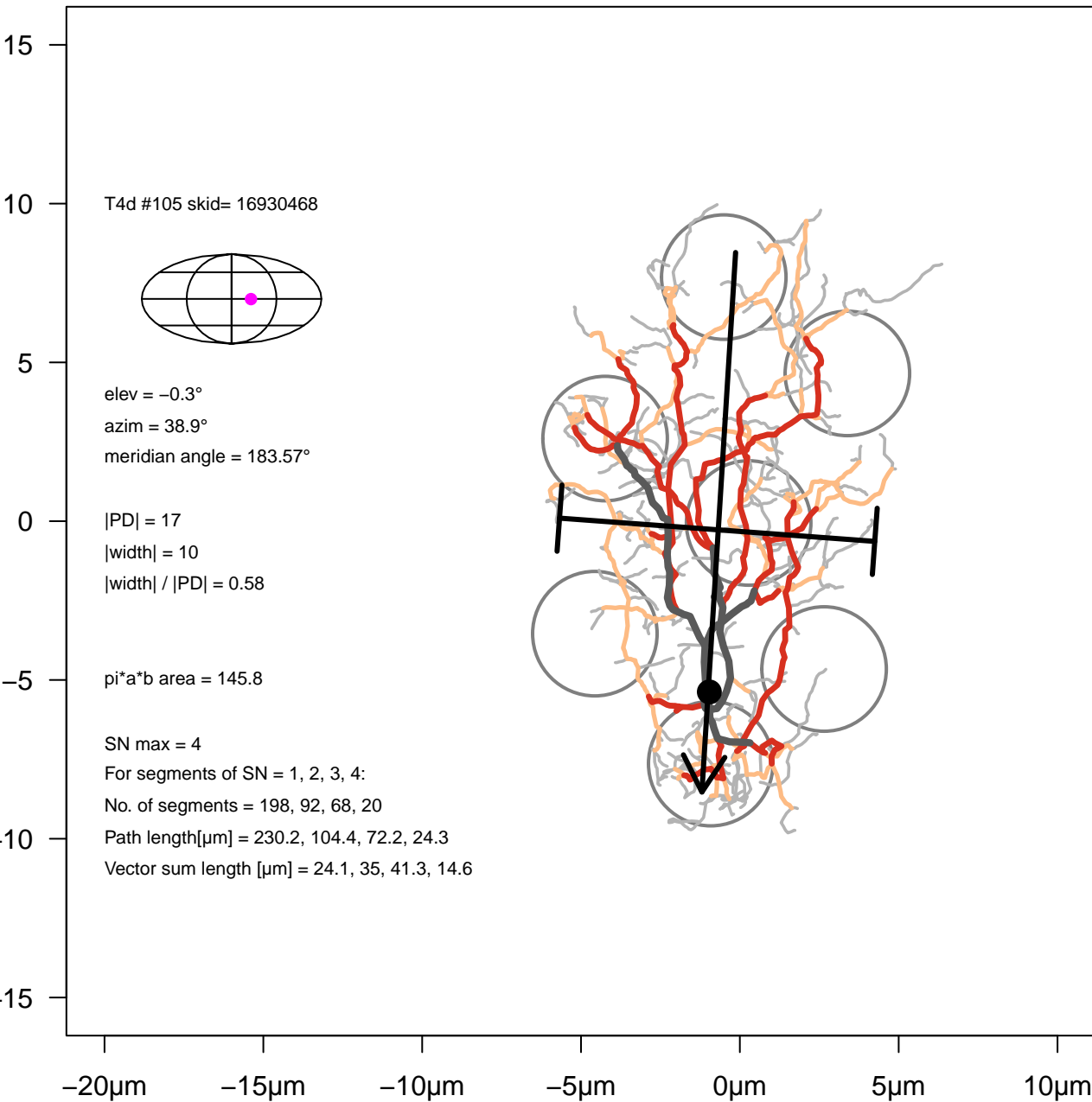

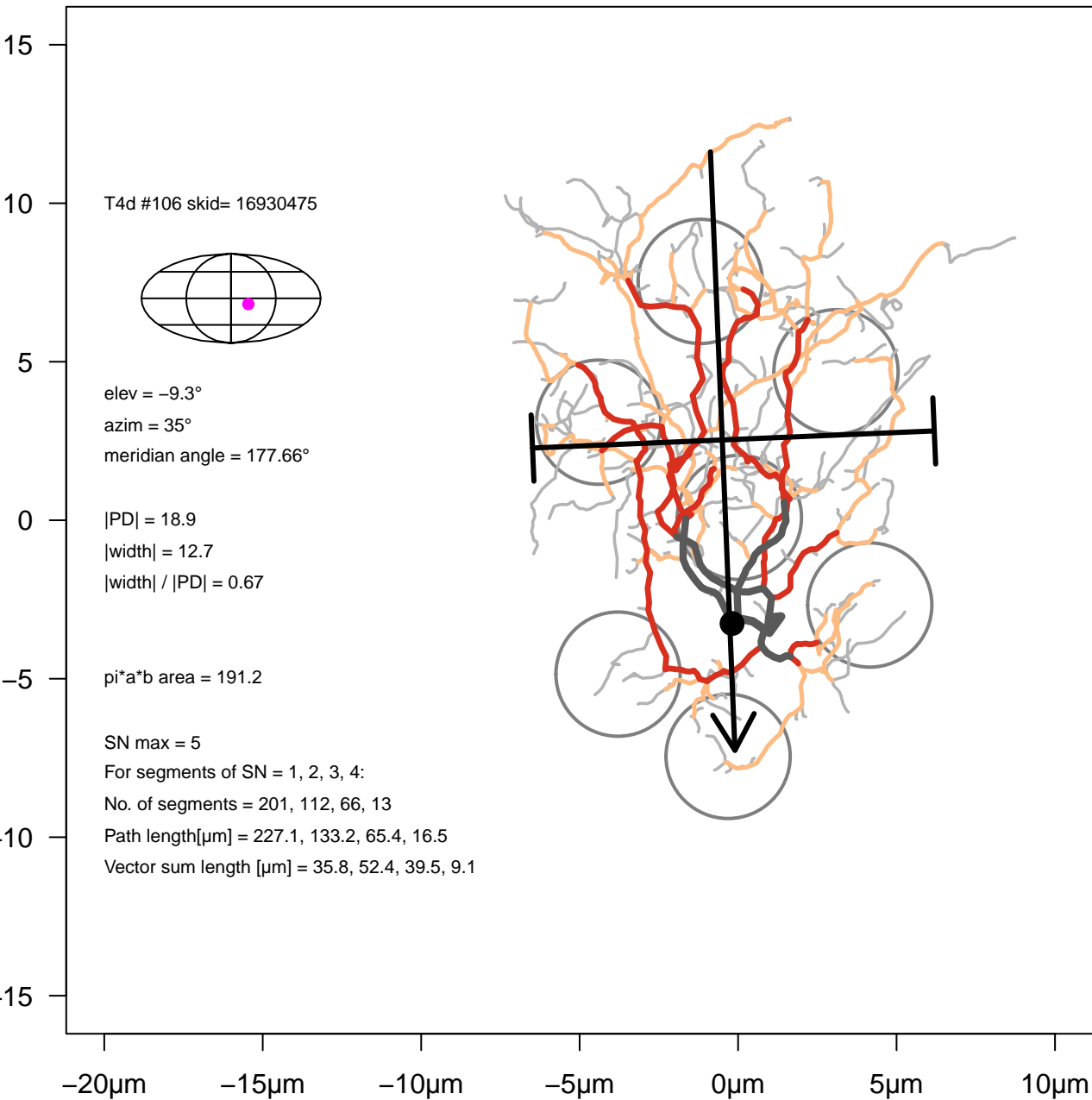

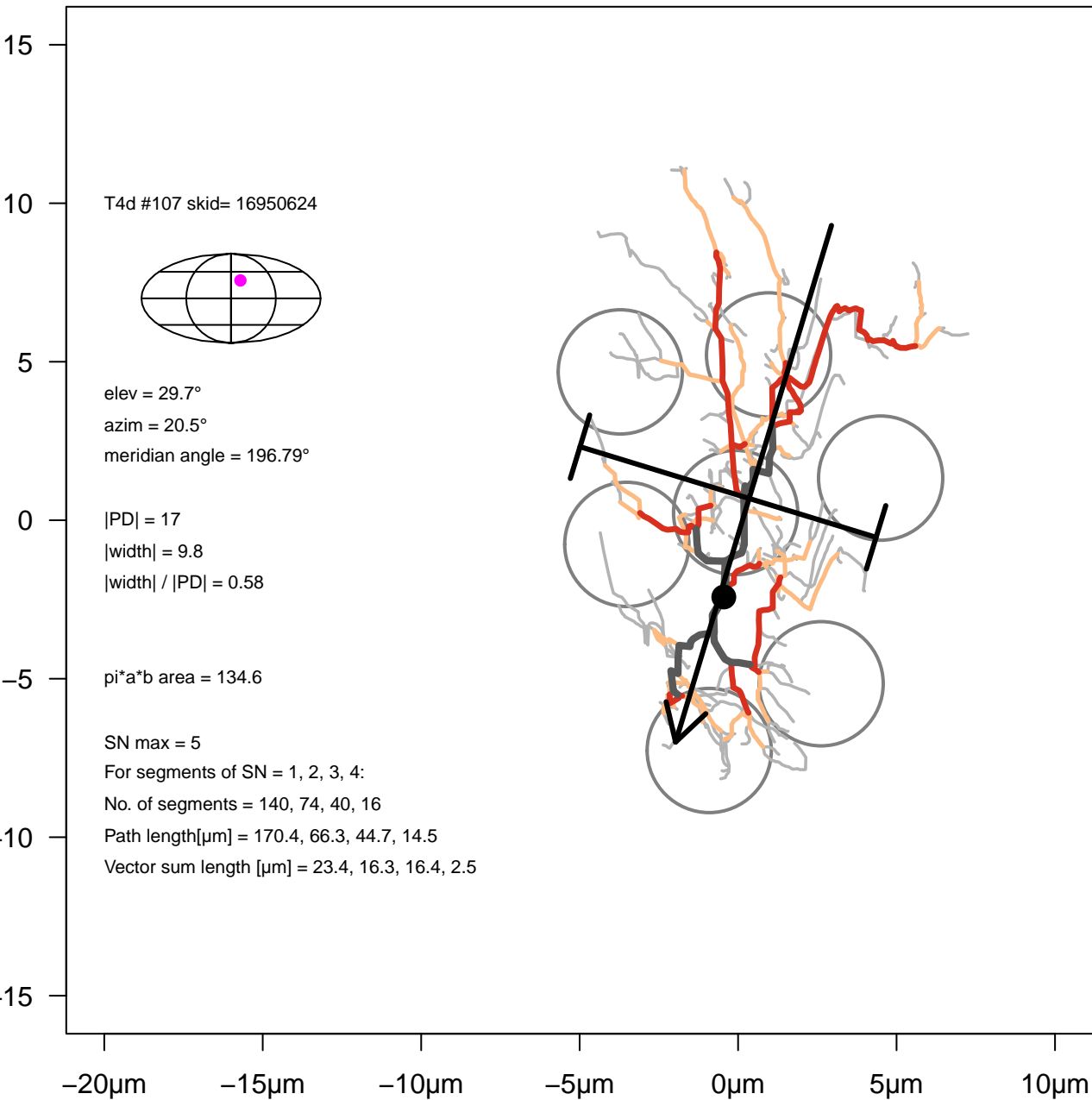

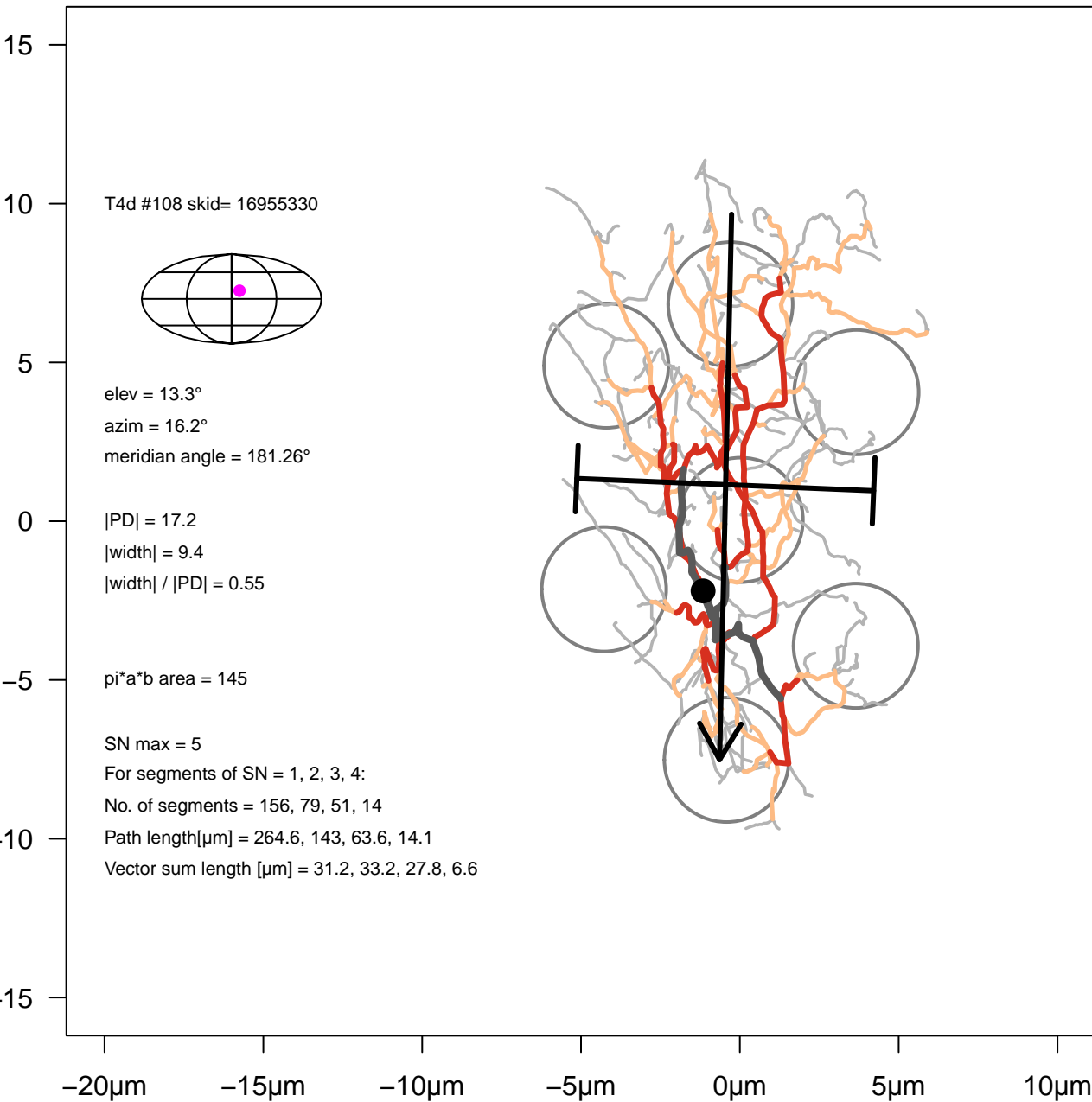

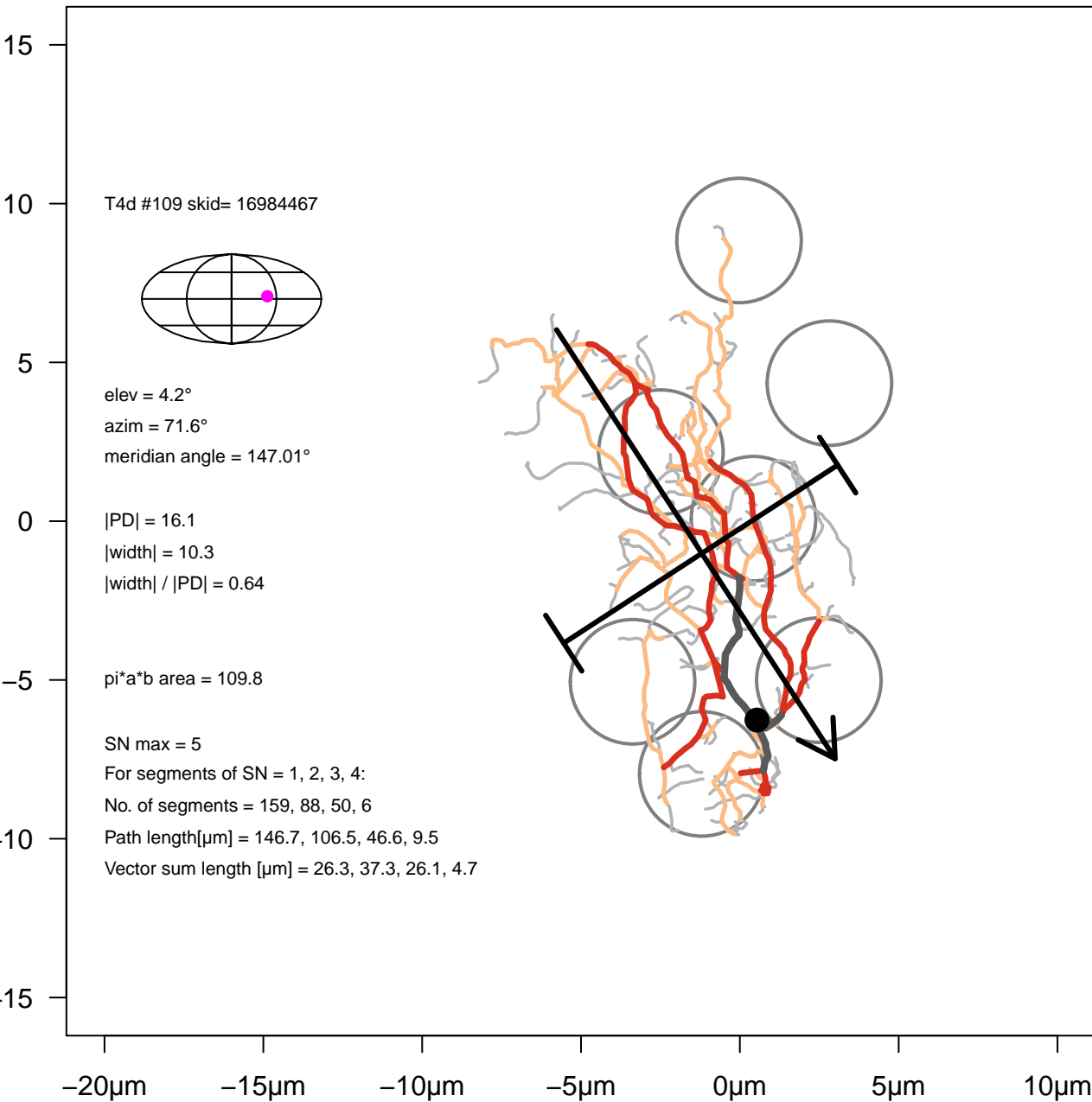

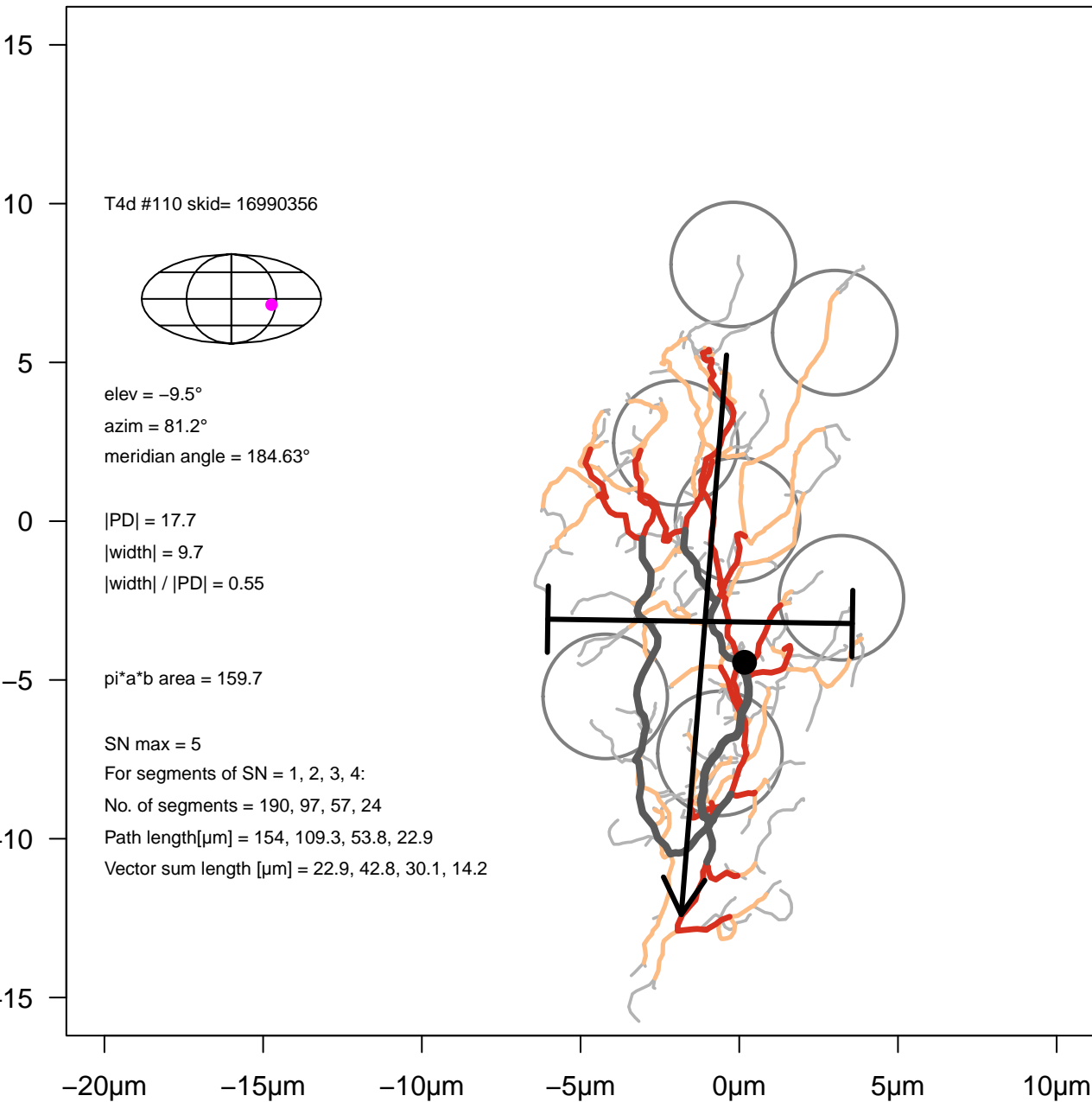

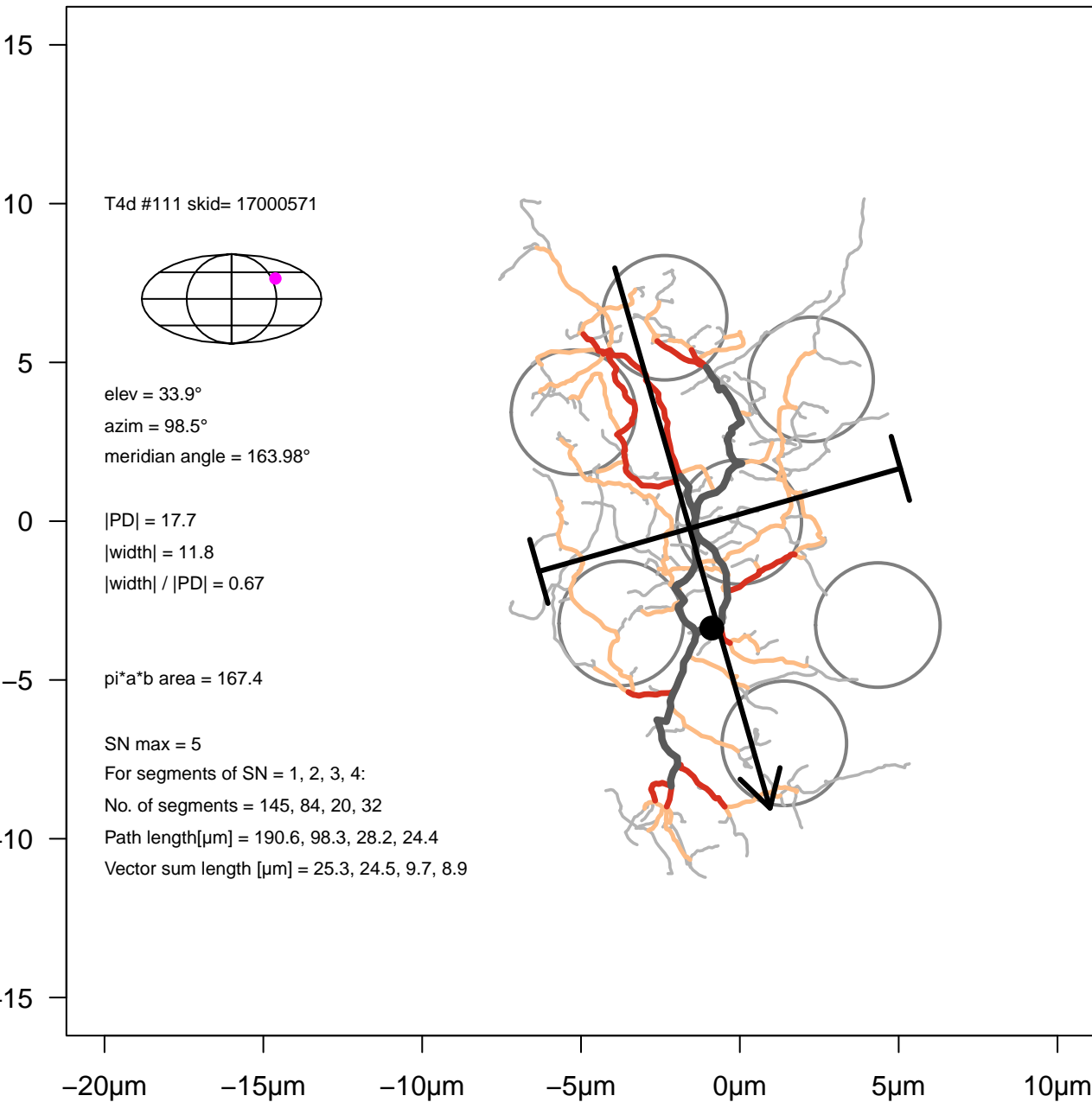

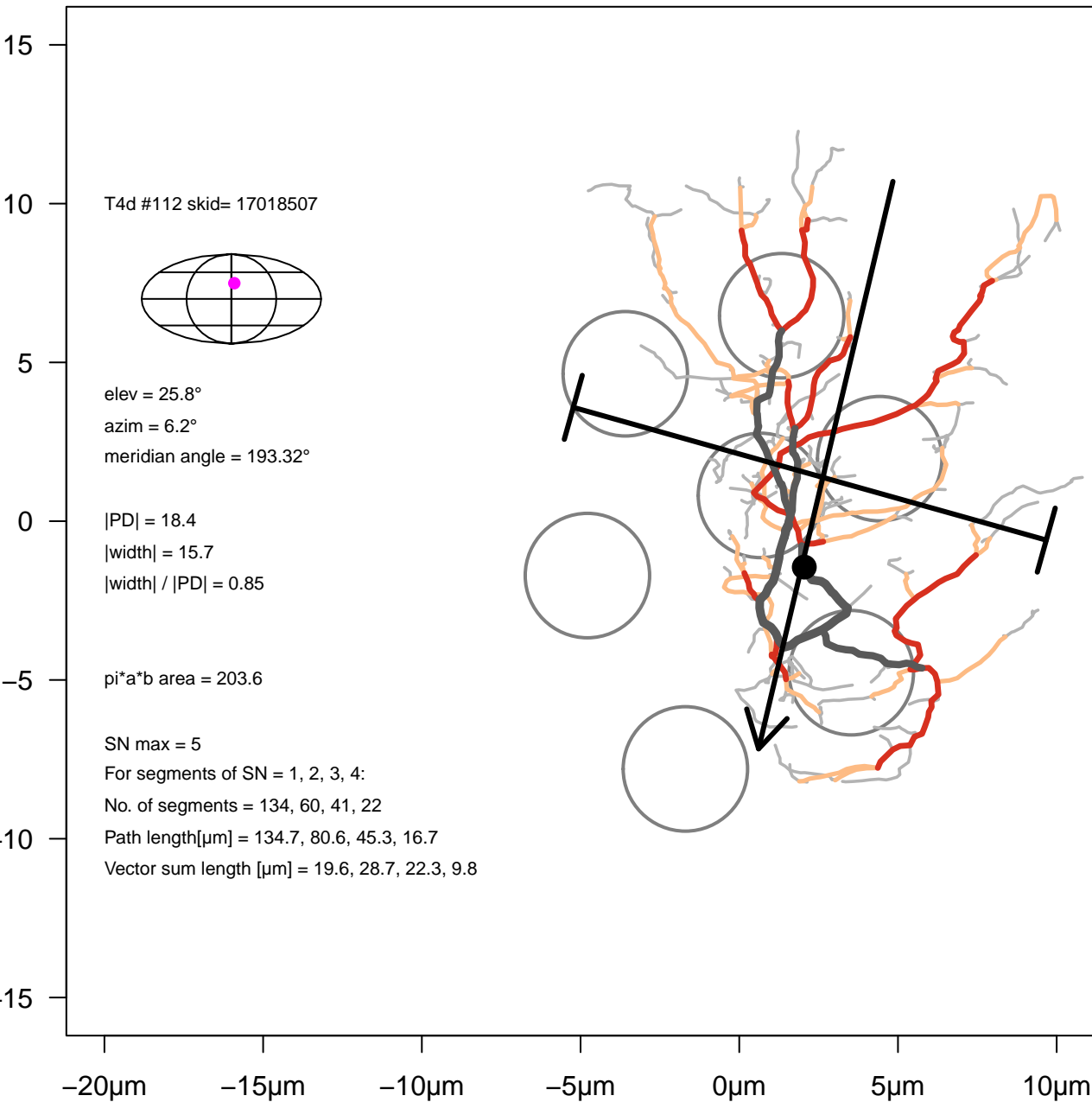

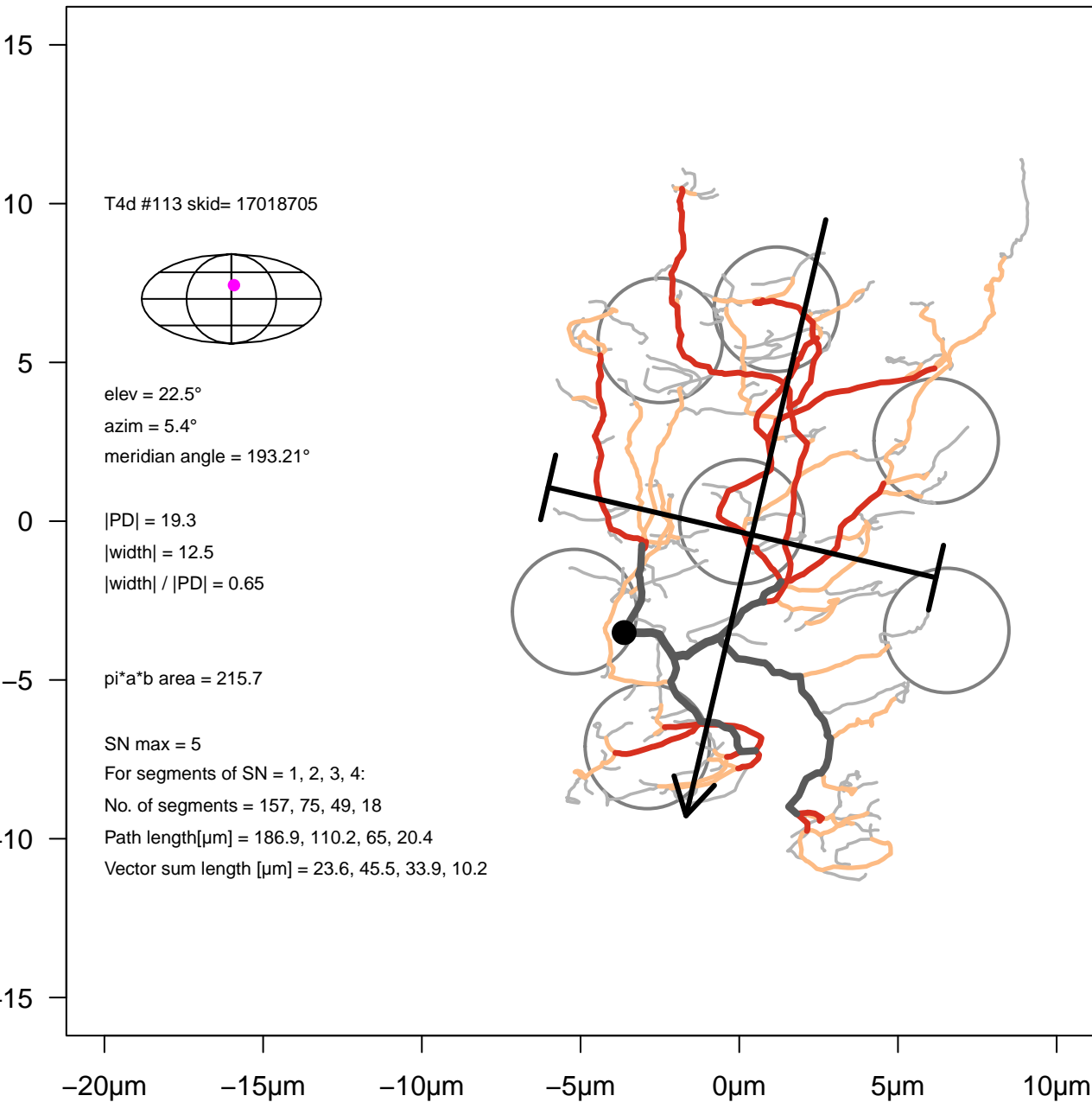

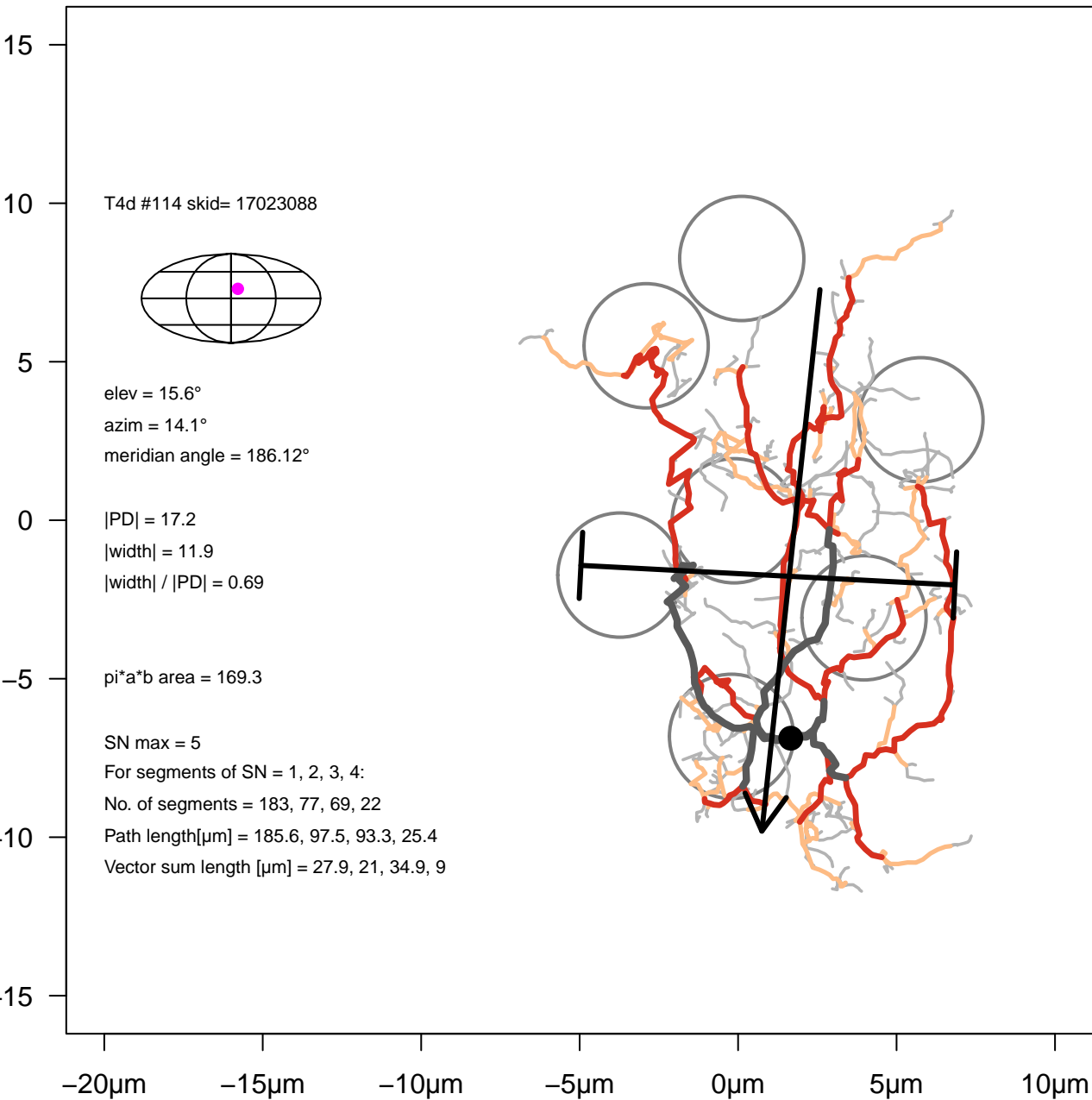

Supplement: Supplementary file 3 — Galleries of T4 neurons with PDs. All T4 neurons reconstructed in the FAFB dataset: 38 T4a, 176 T4b, 22 T4c, 114 T4d, are plotted similarly as in Fig. 2g. Using the eye map established in Fig. 4a, we include the position (elevation and azimuth angles) in the eye coordinate. The angle between T4’s PD and the local meridian line is computed instead of using the +v-axis as the reference, as in Fig. 2g. The meridian line is defined as the direction line going from the south pole to the north pole in the eye reference frame (often close to the +v-axis). The cell and surrounding columns are also aligned such that the vertical direction in the plot coincides with the meridian direction. A summary of the Strahler number analysis for each cell is included. [file 41586_2025_9276_MOESM3_ESM.zip › gallery_T4d.pdf]

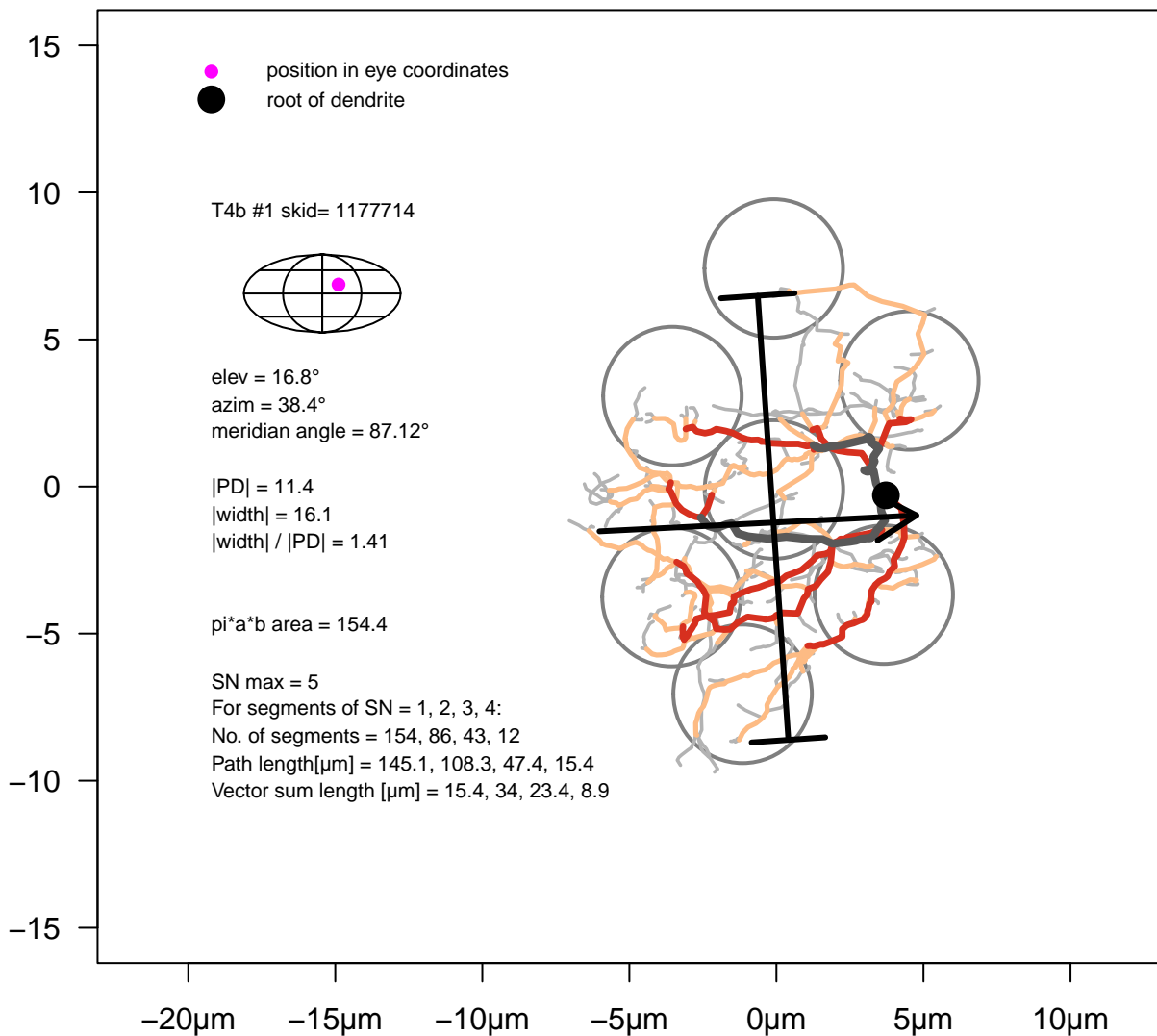

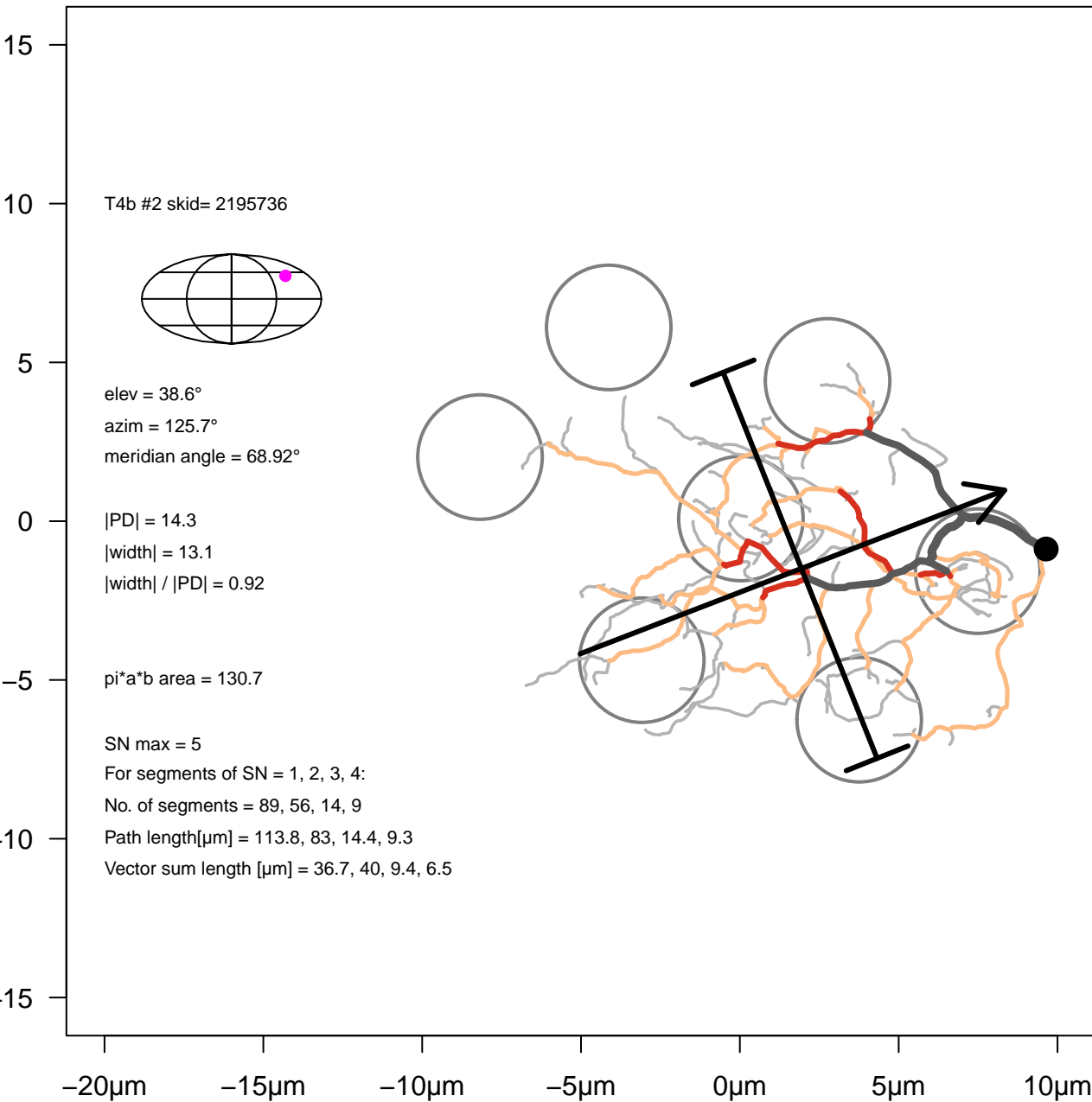

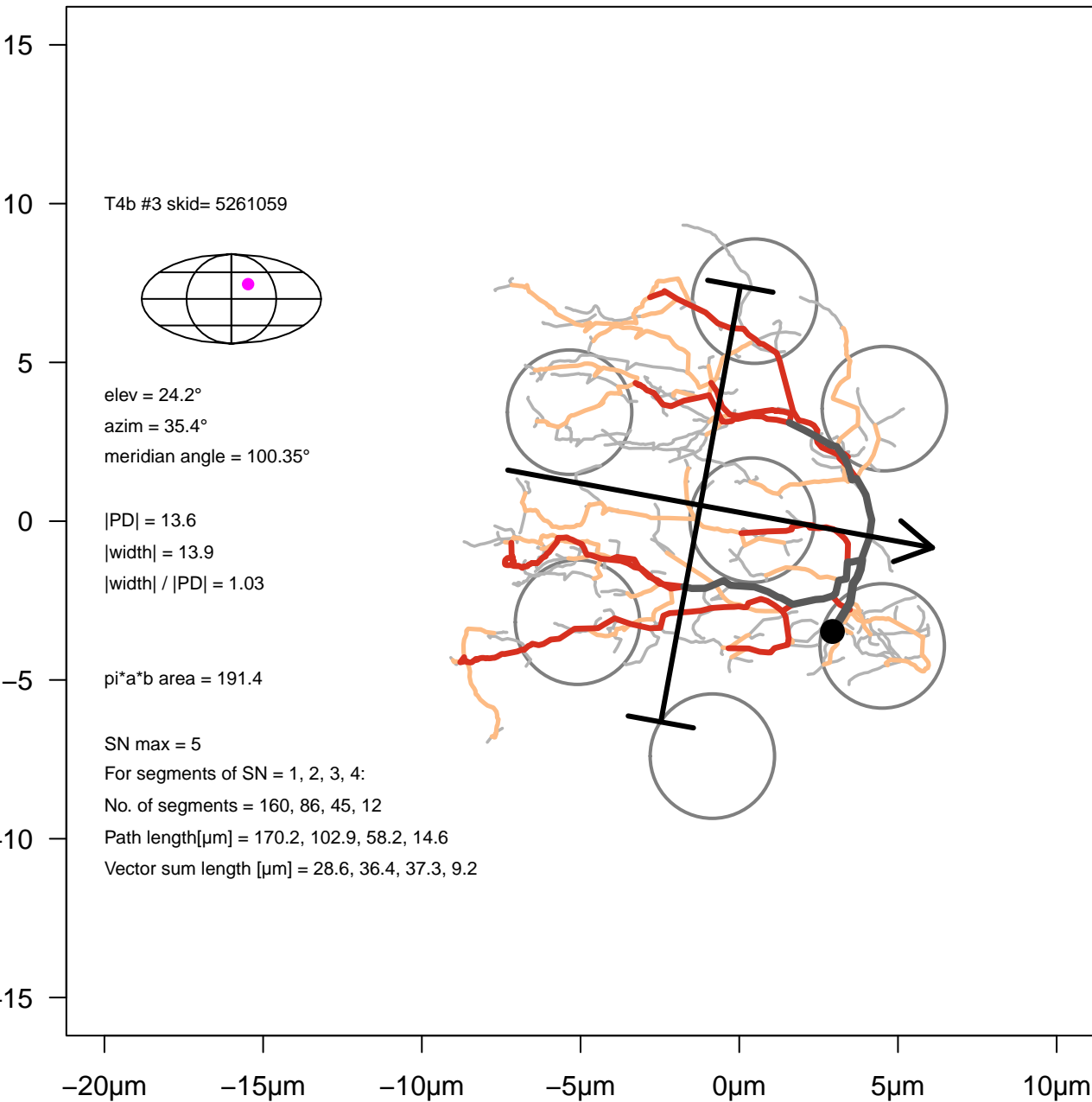

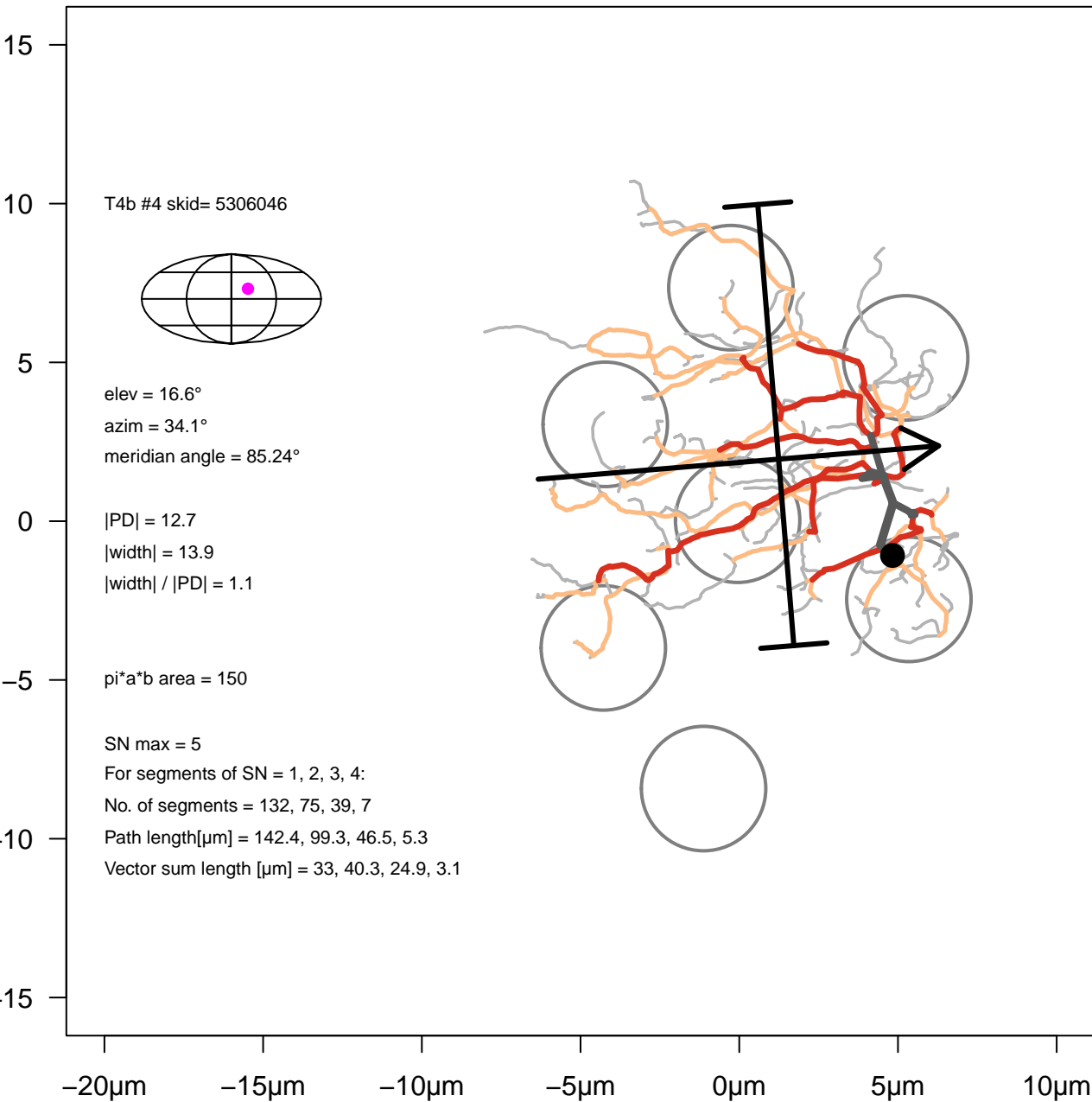

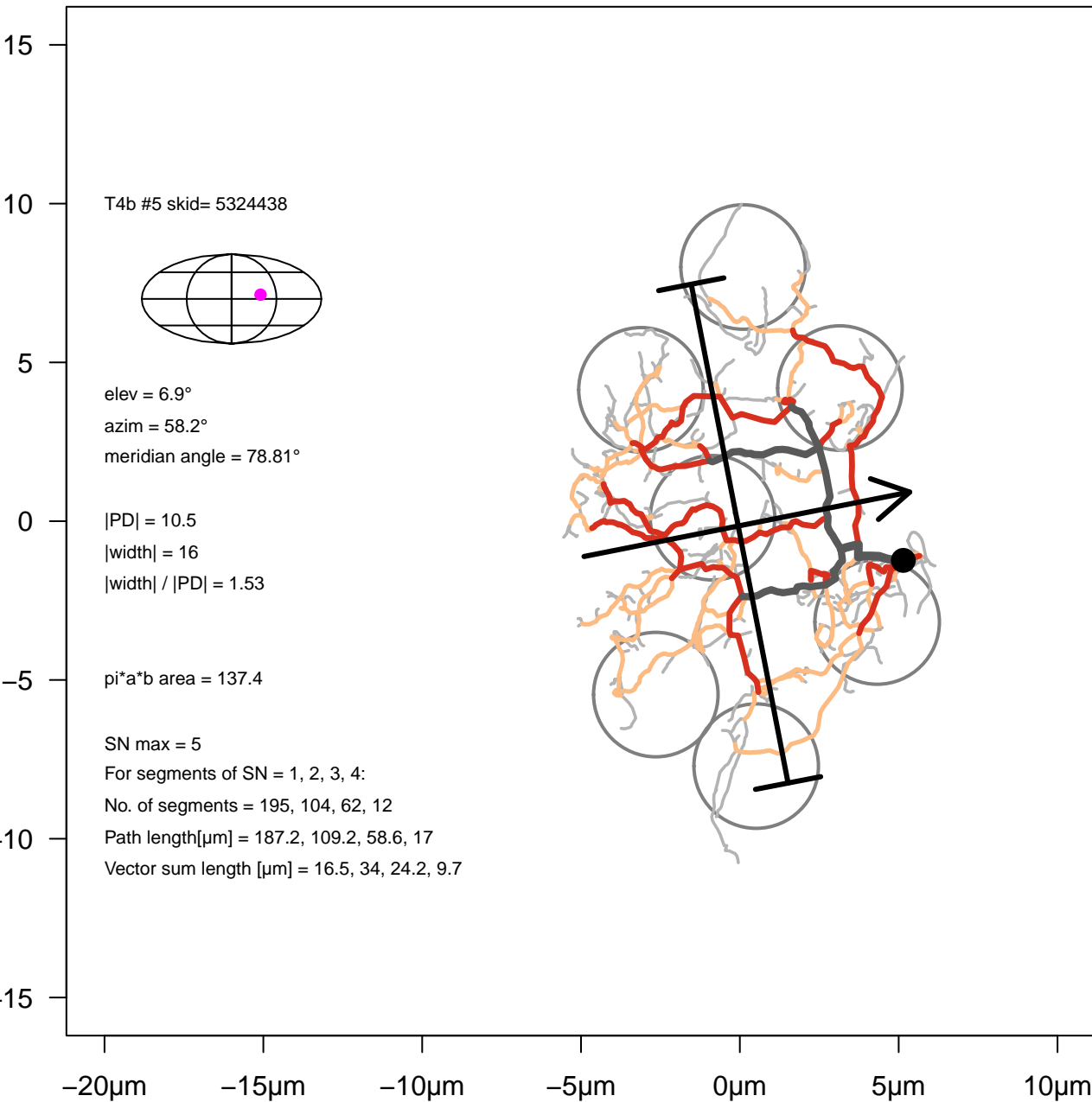

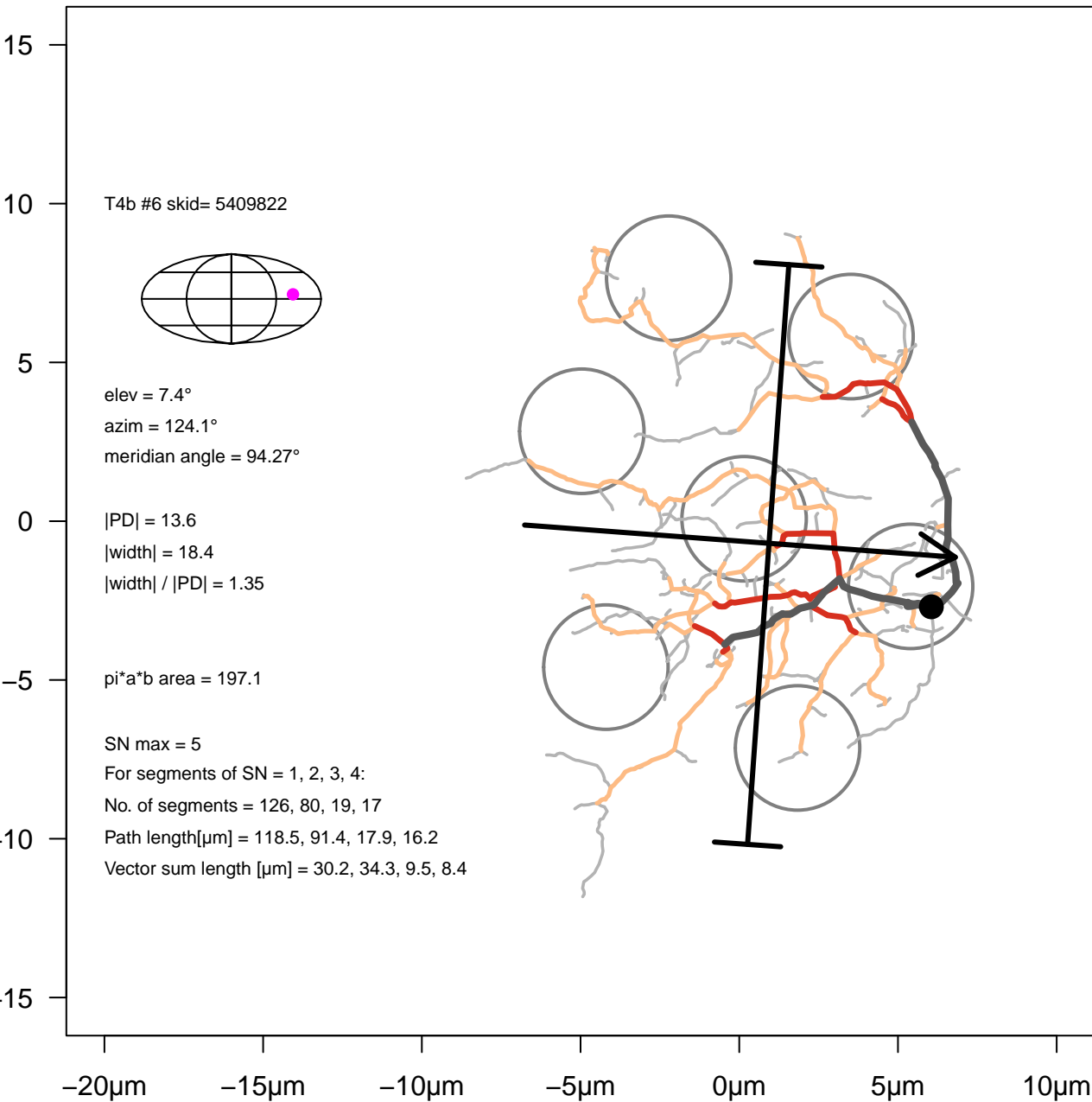

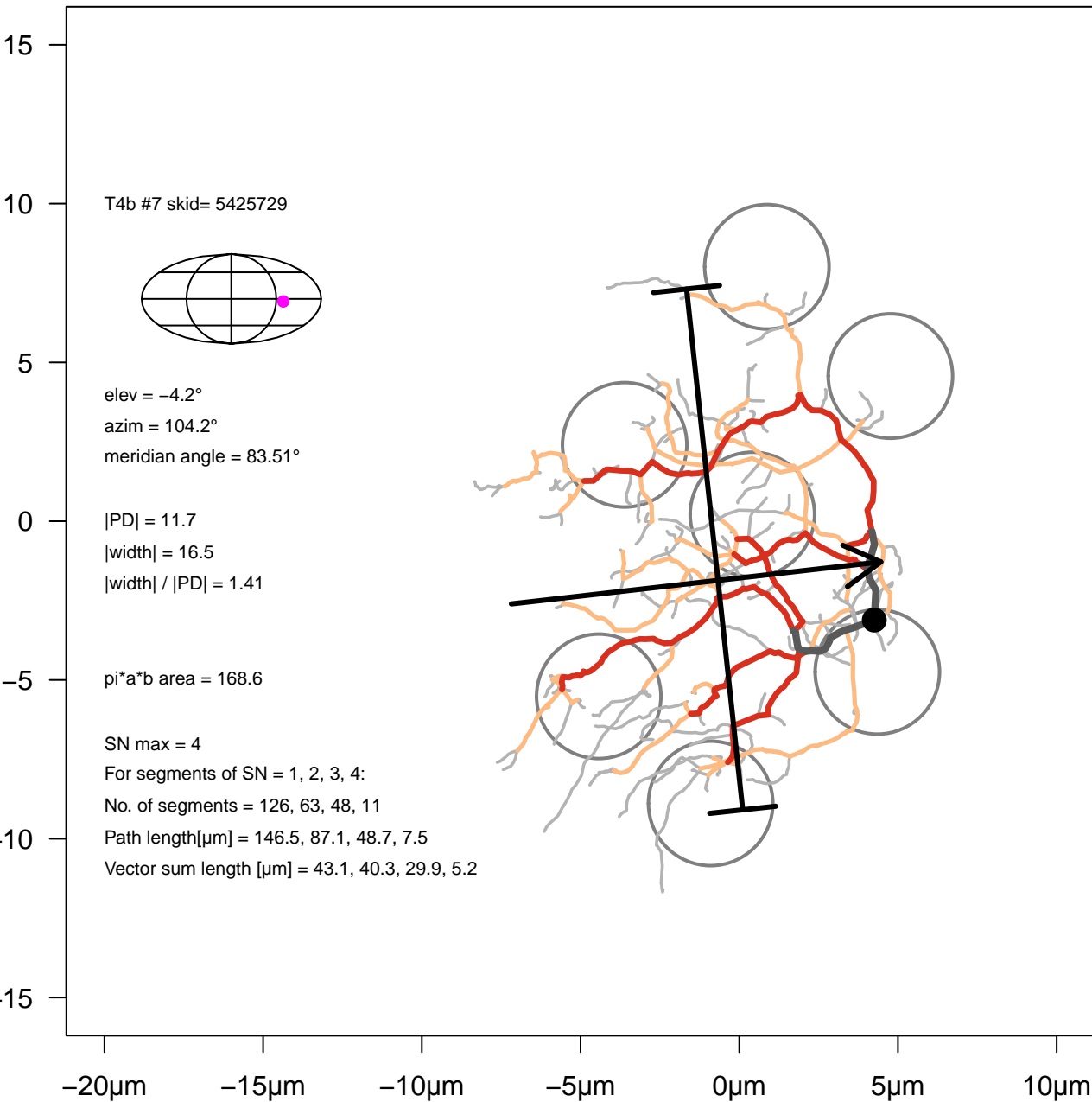

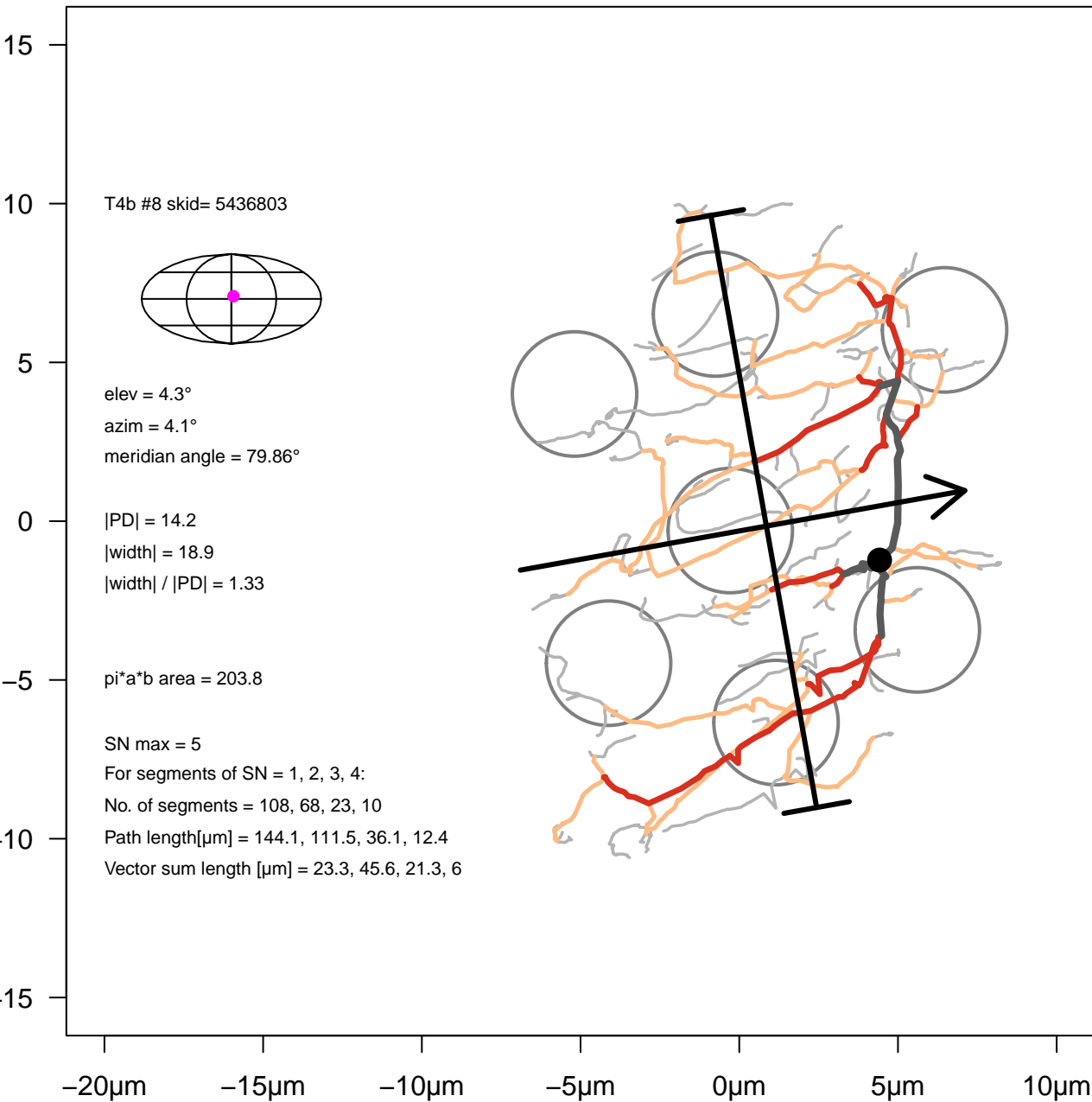

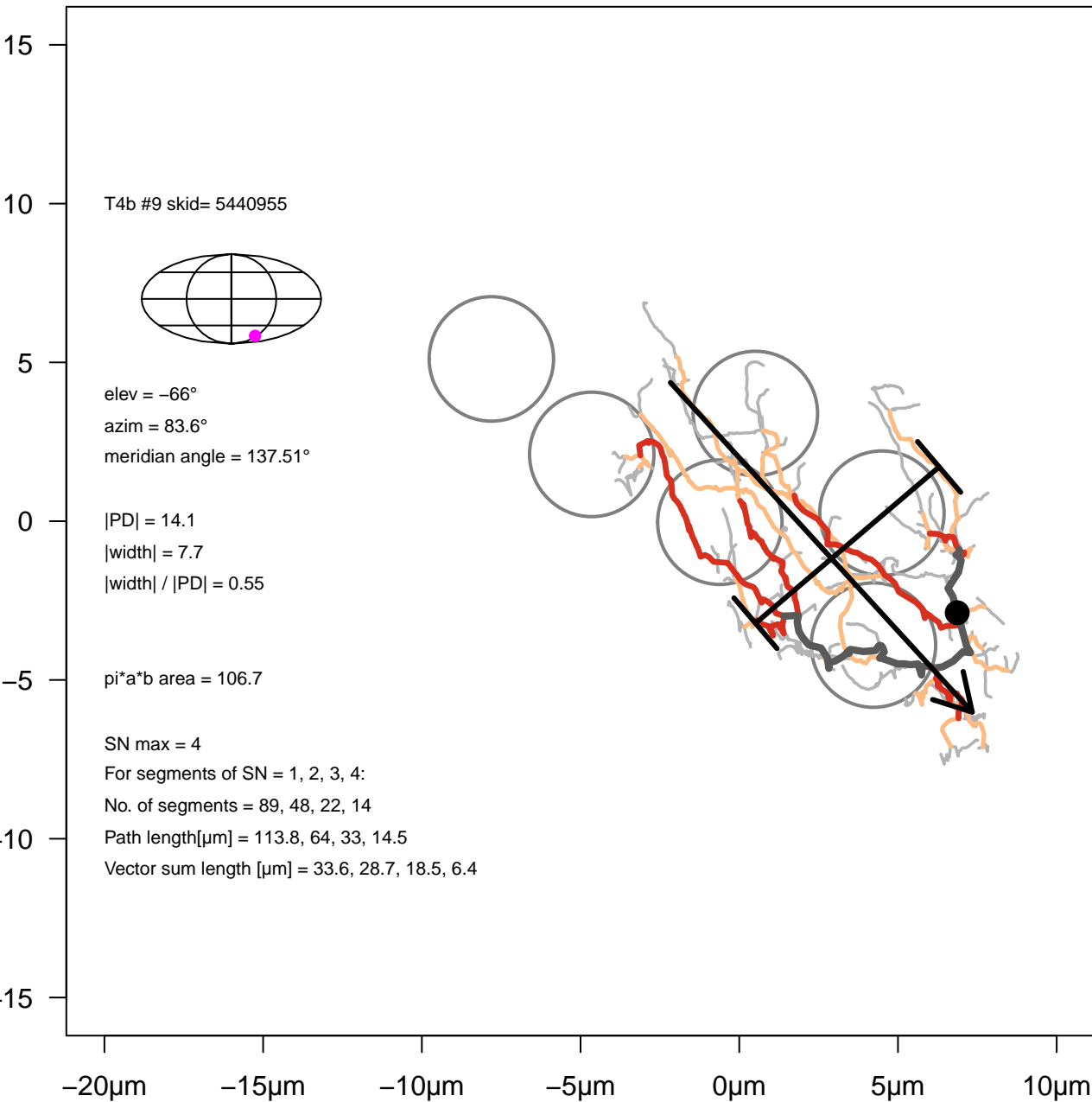

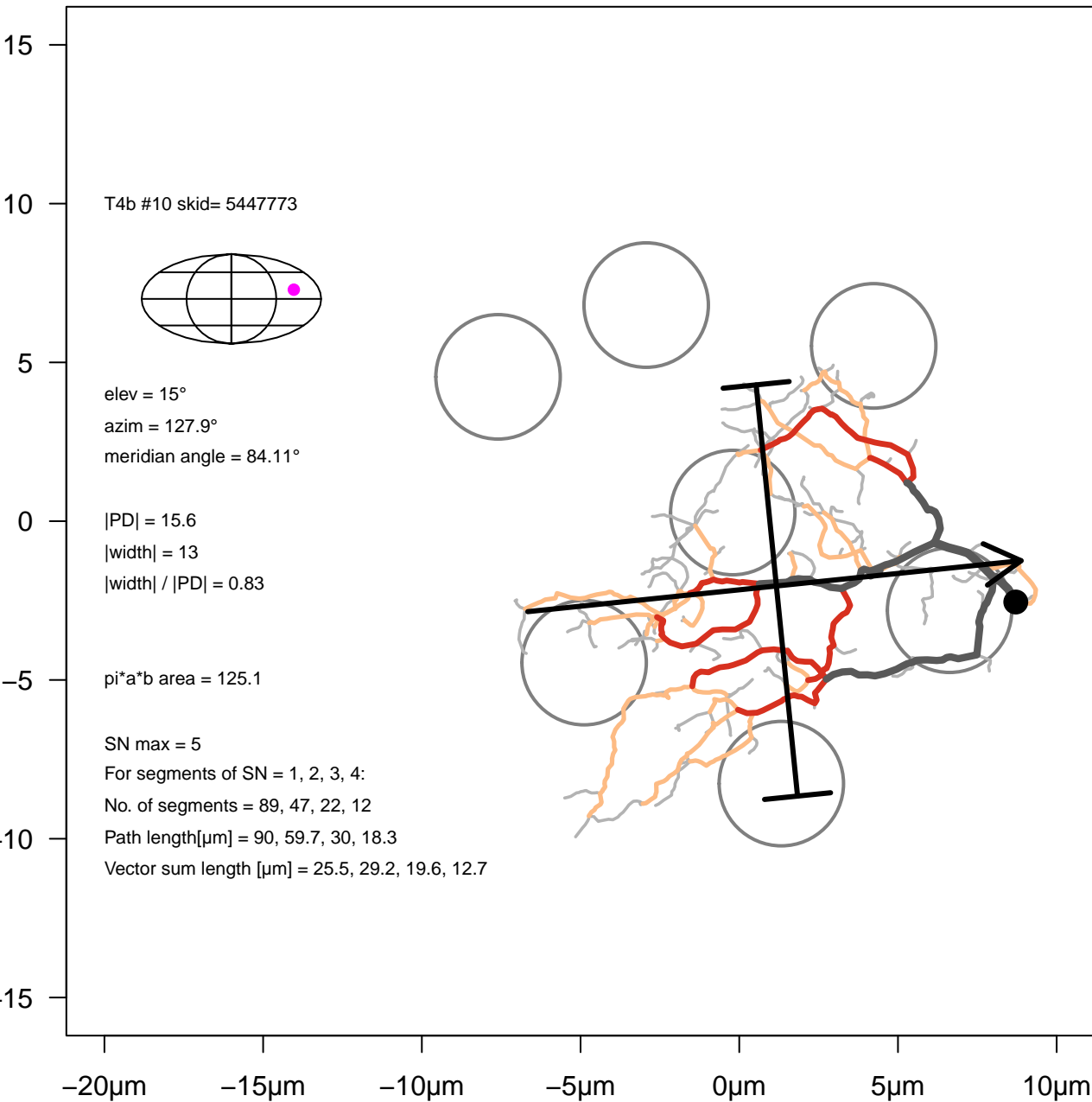

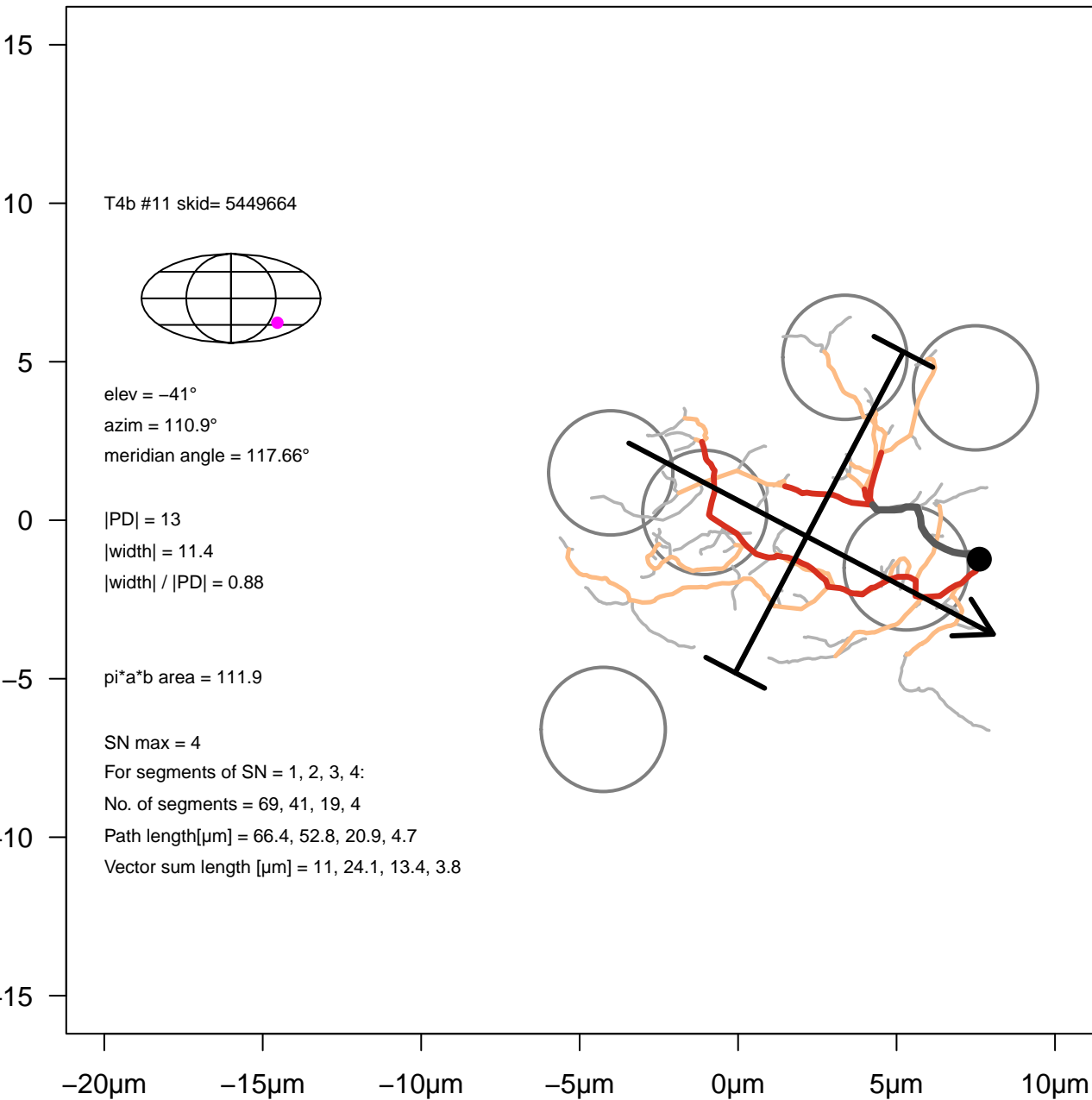

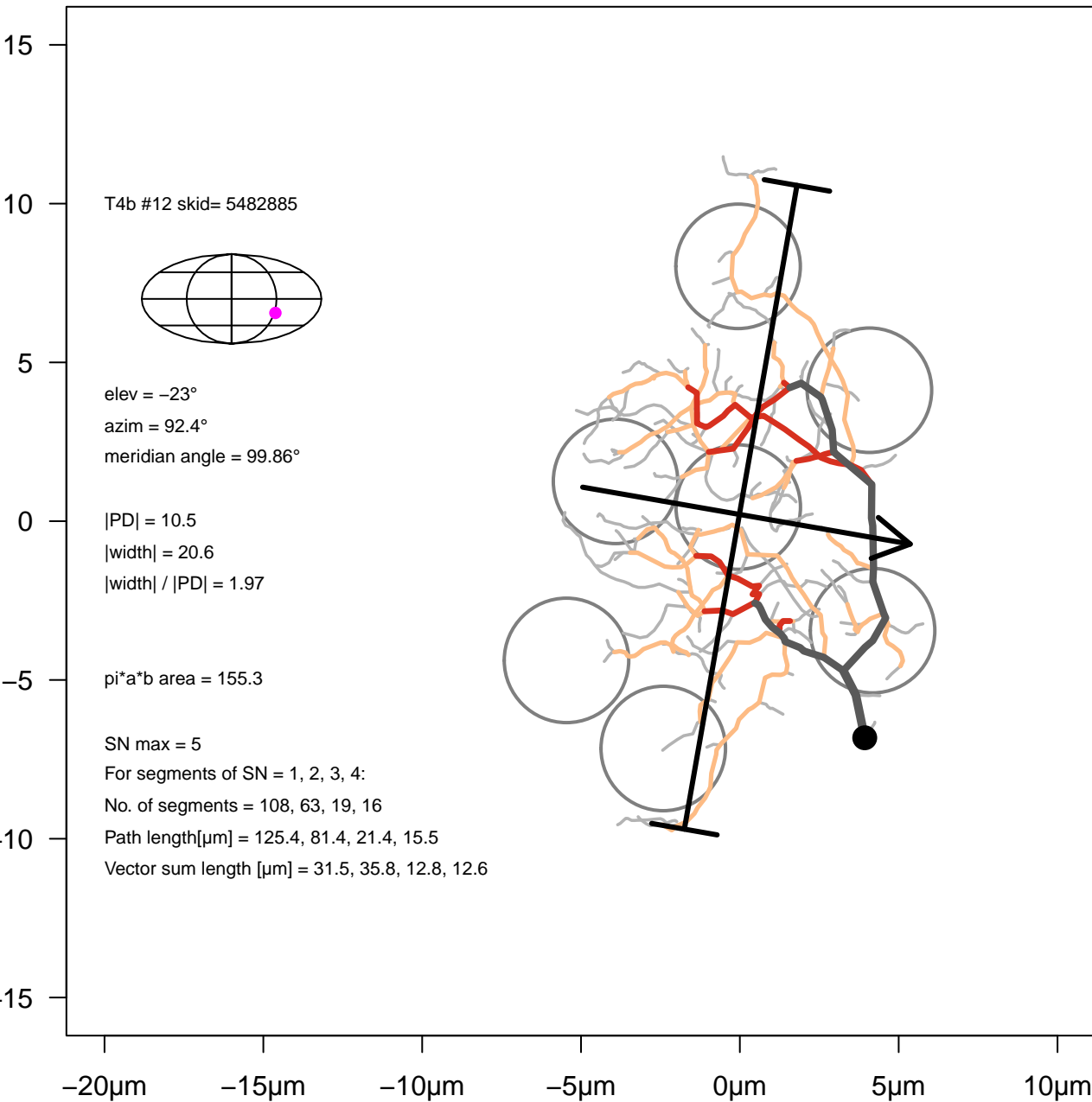

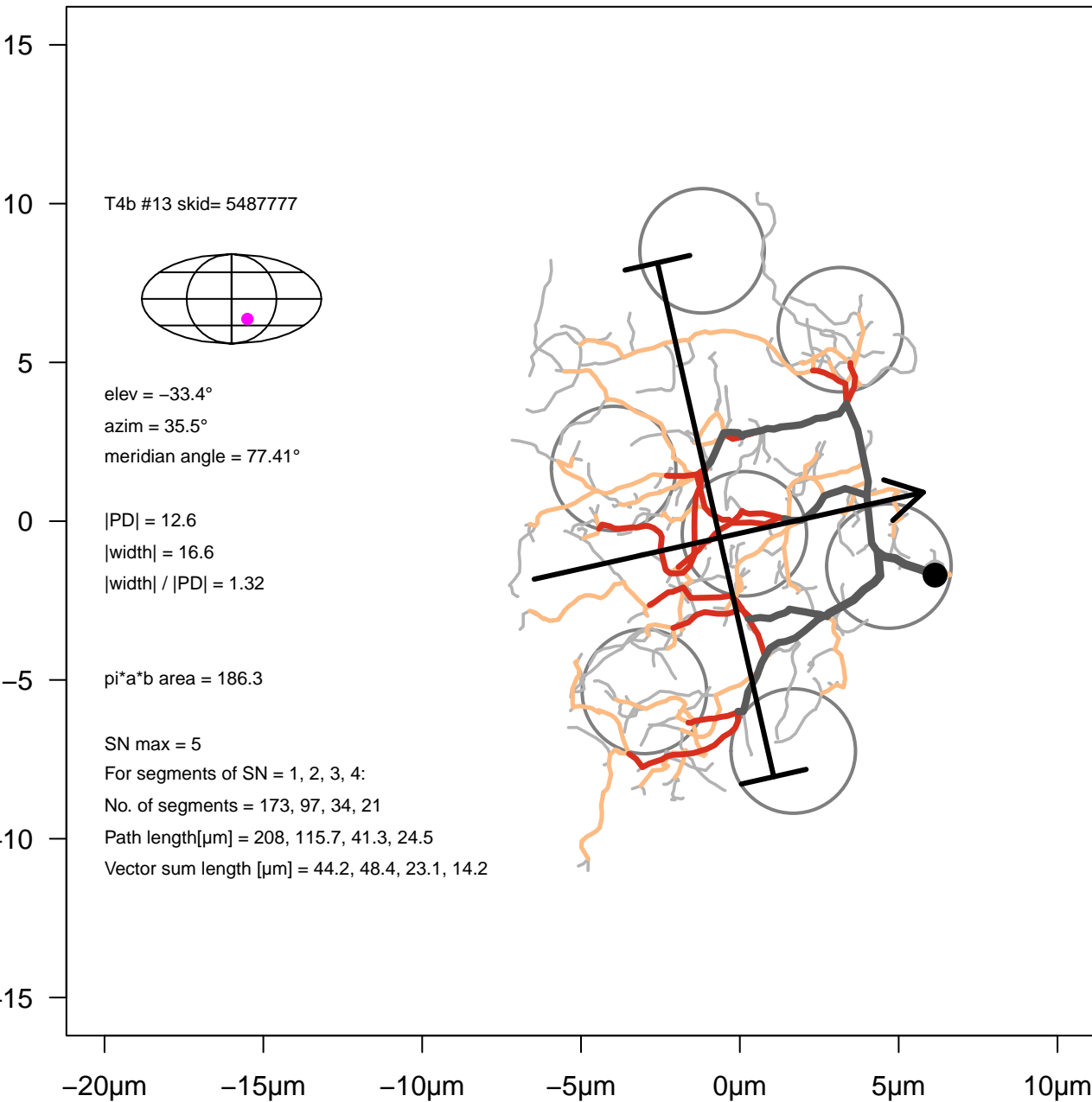

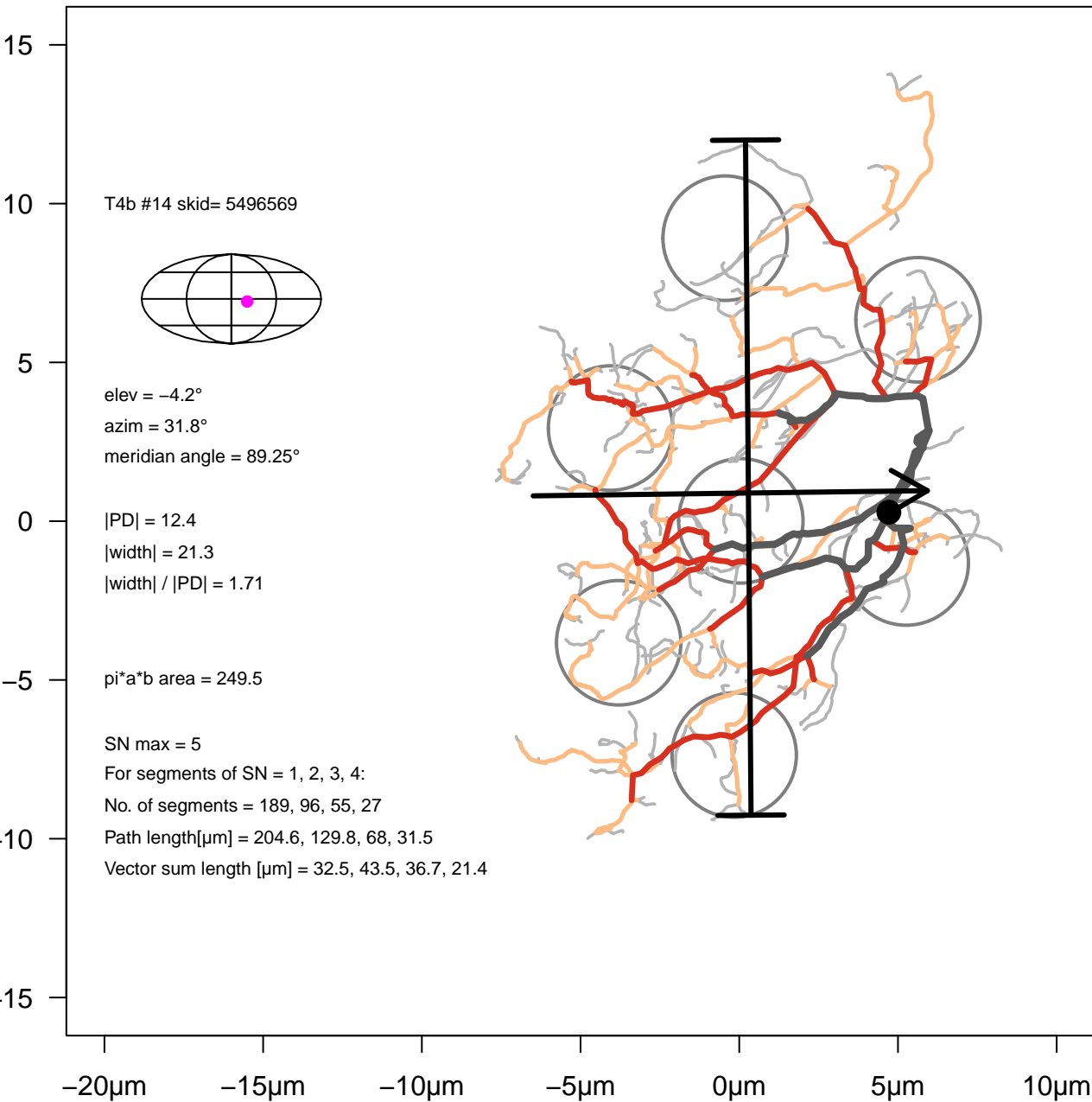

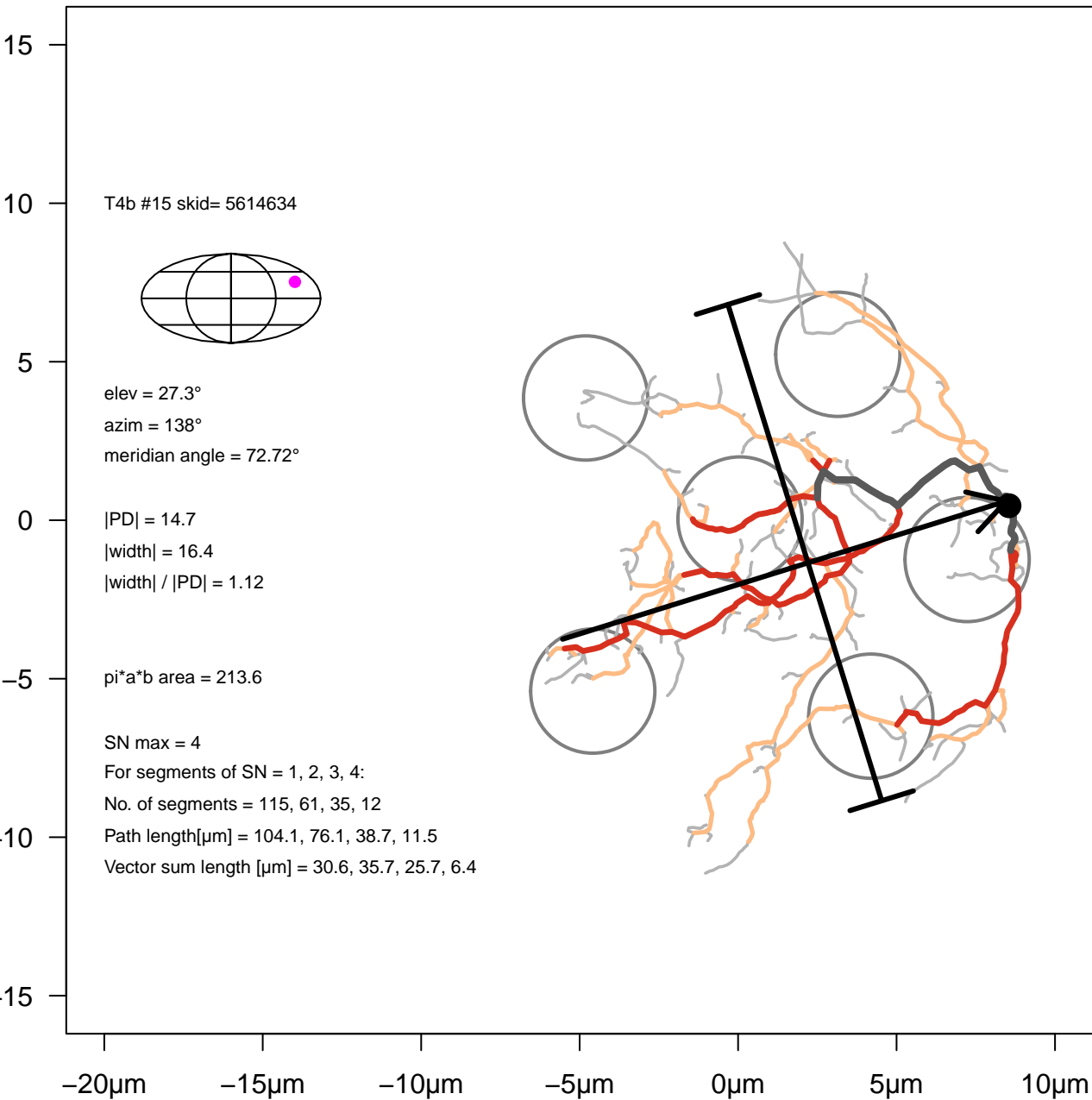

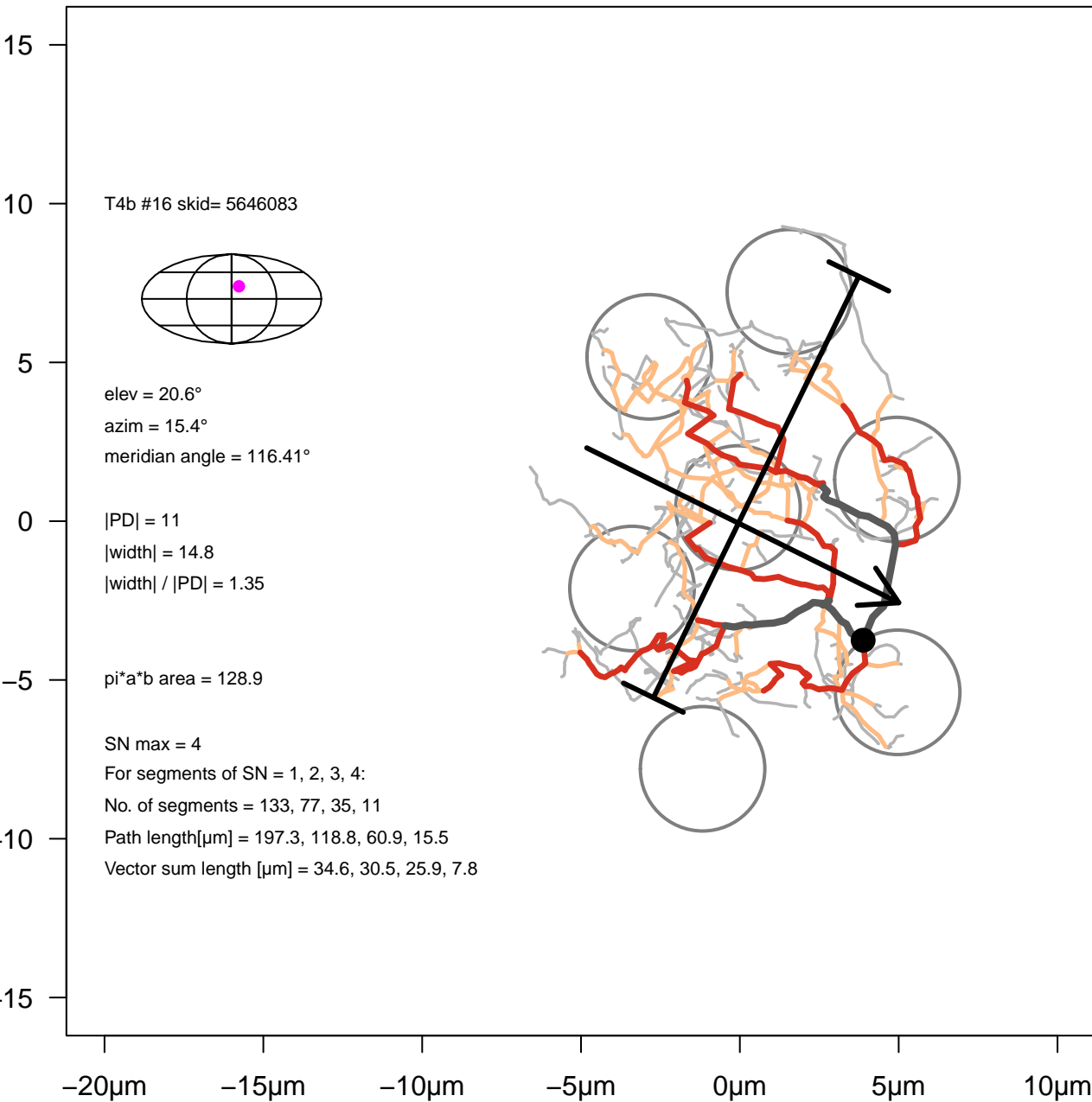

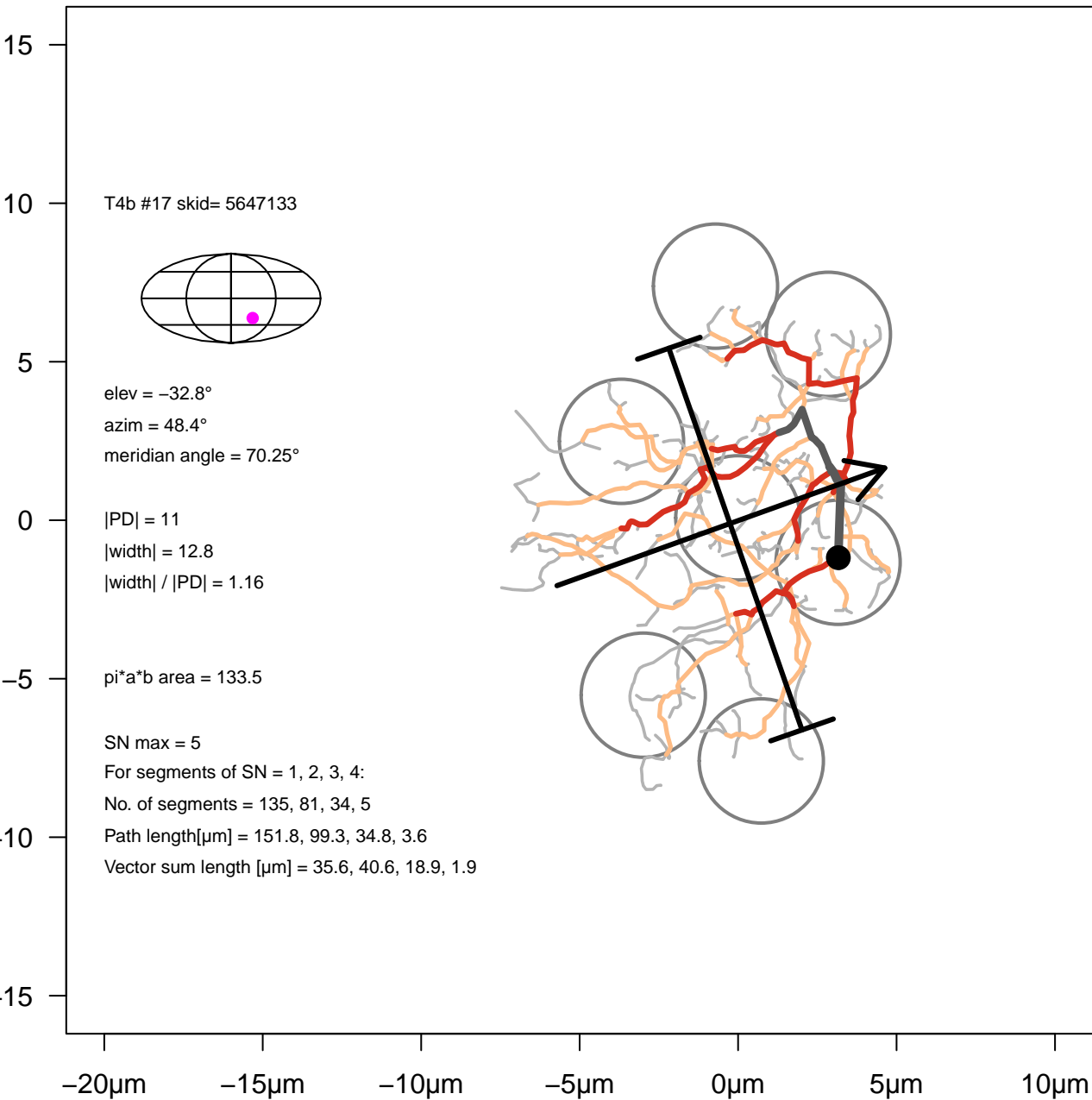

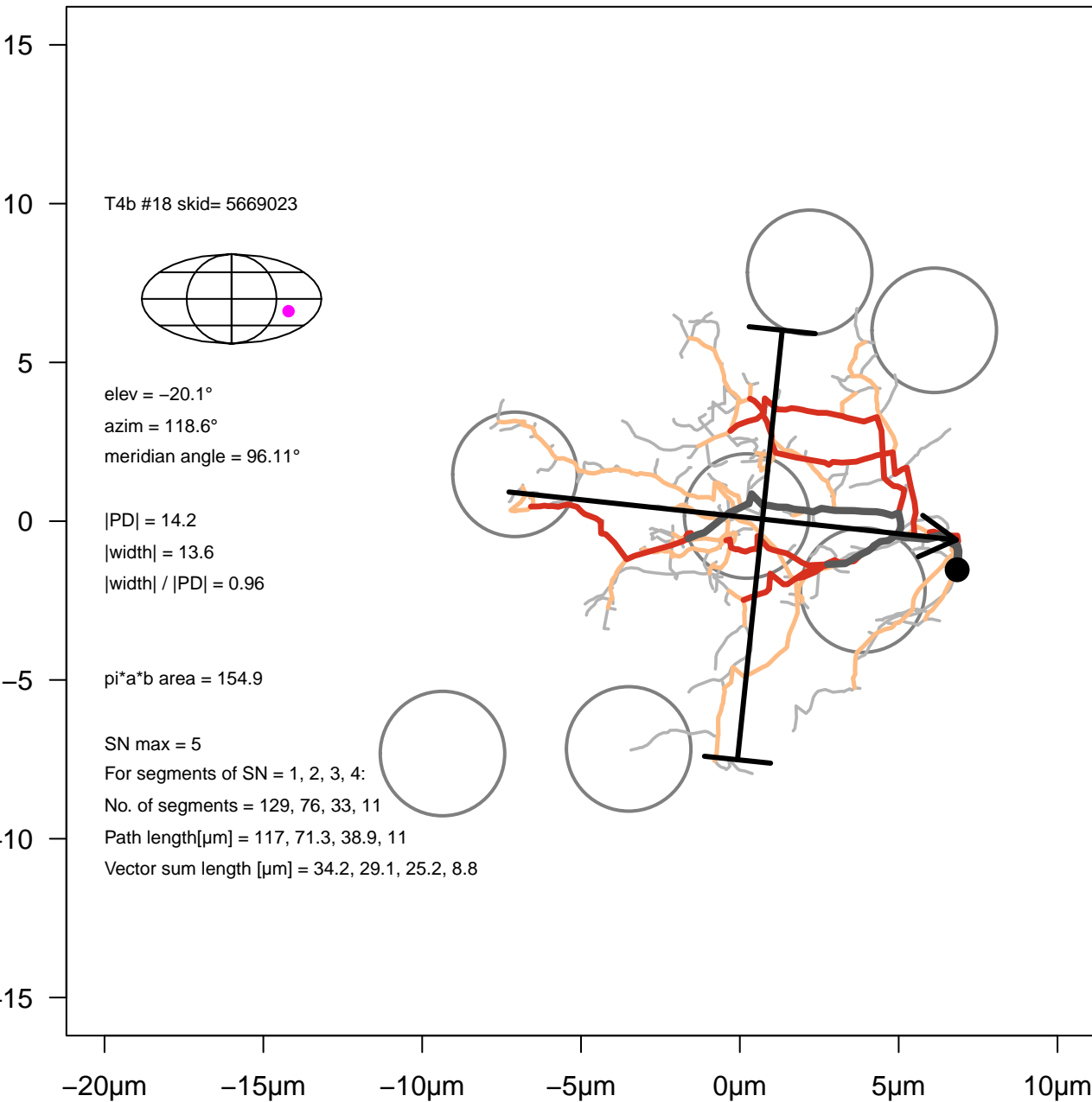

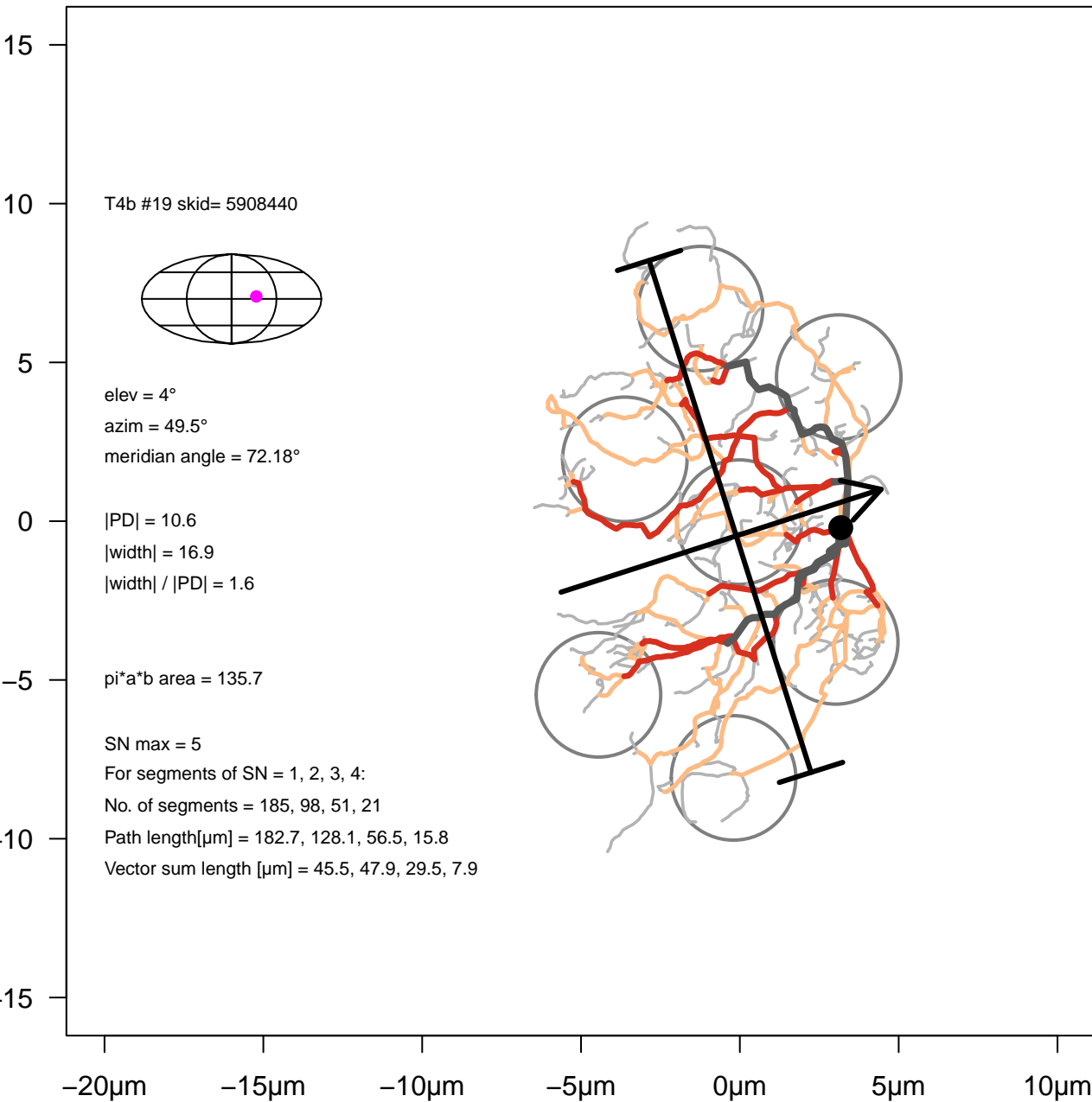

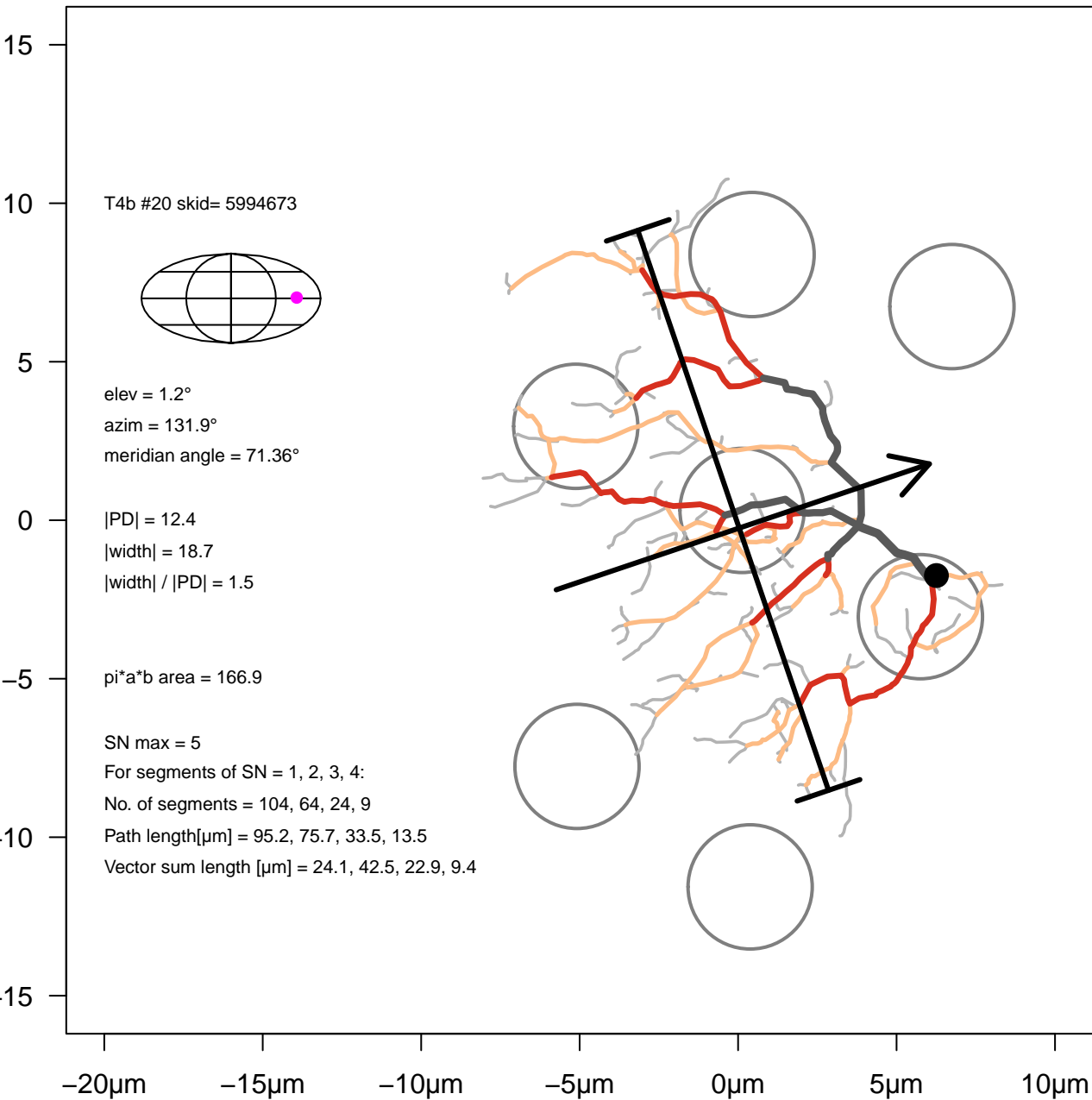

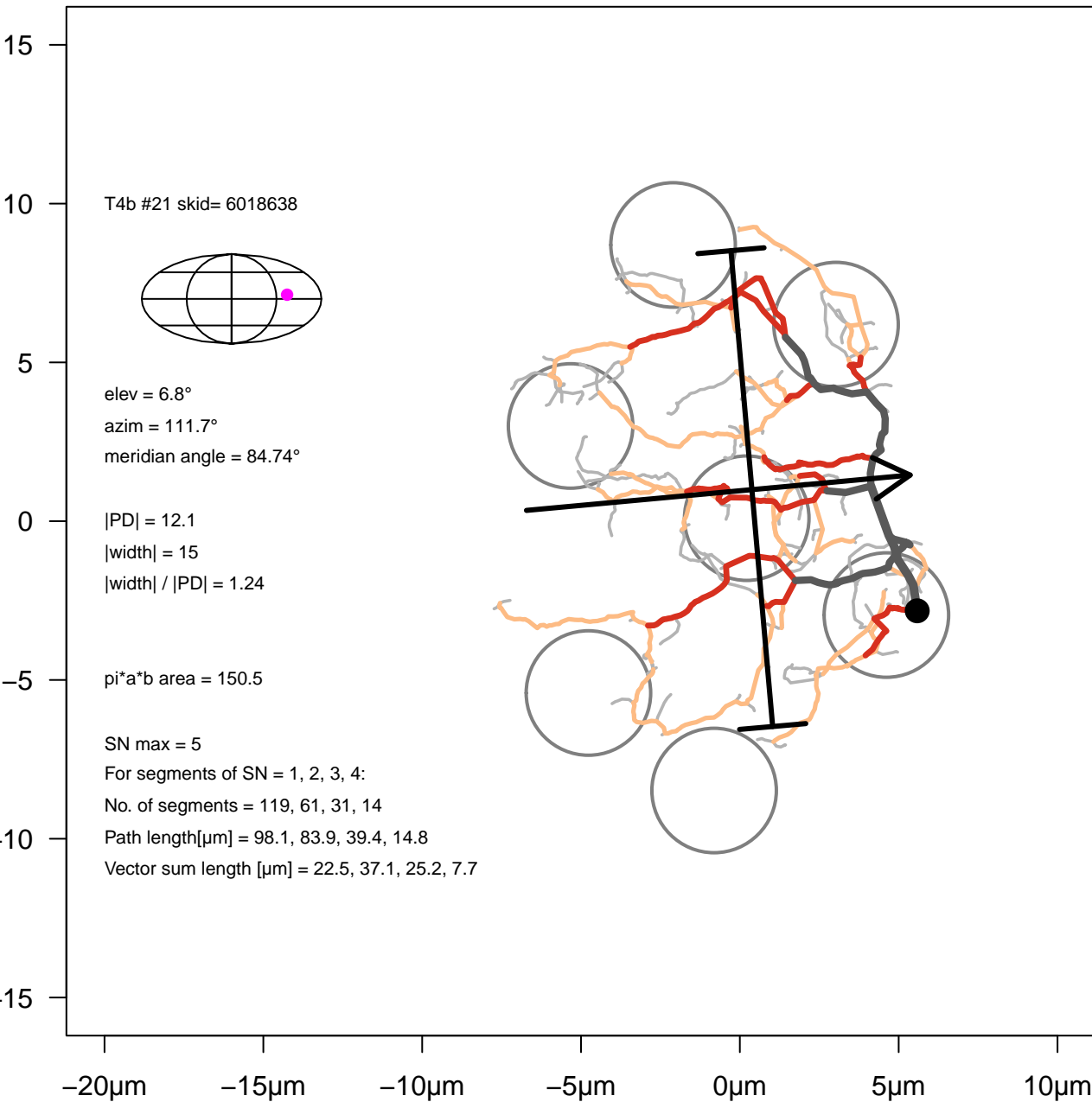

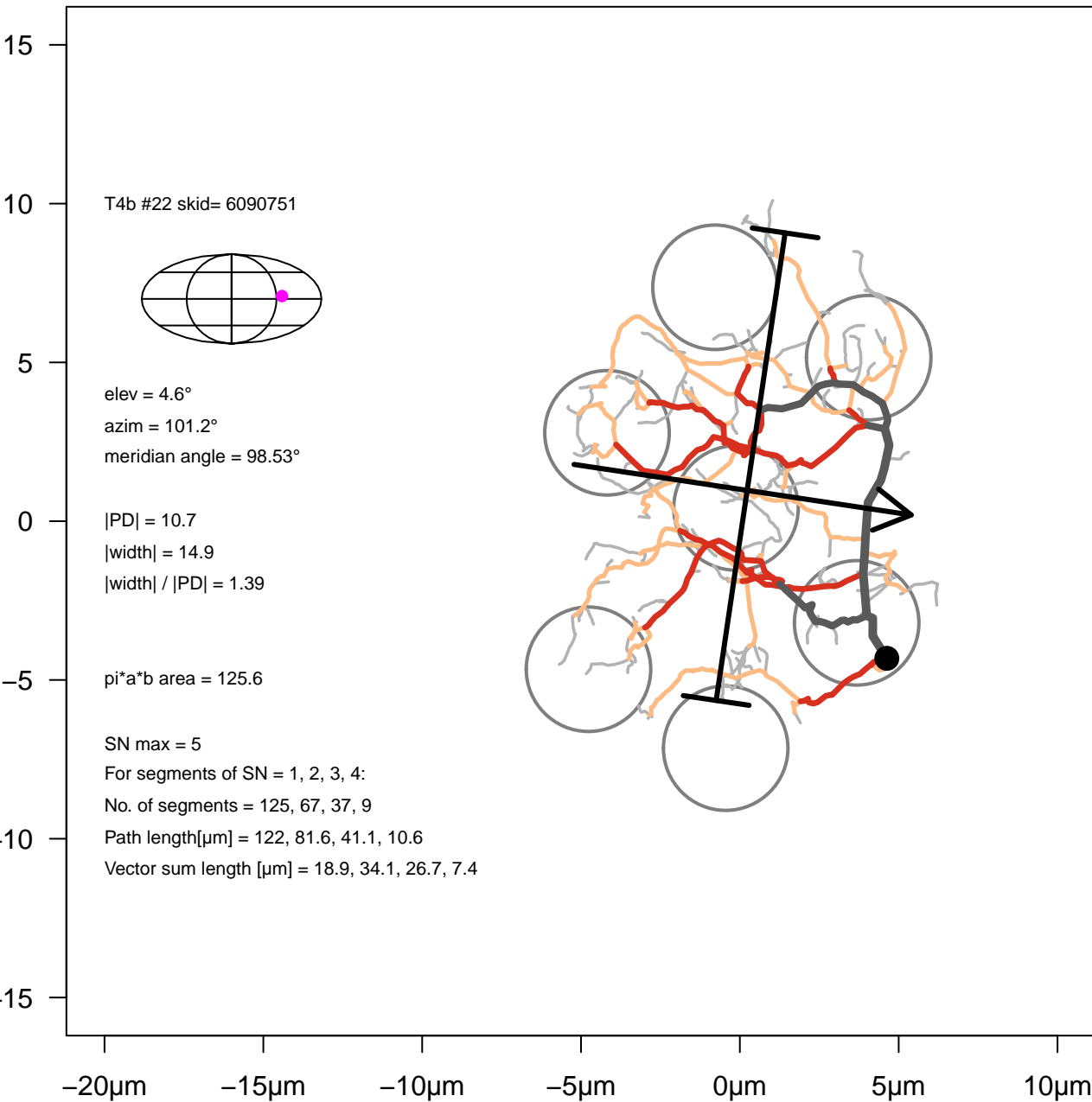

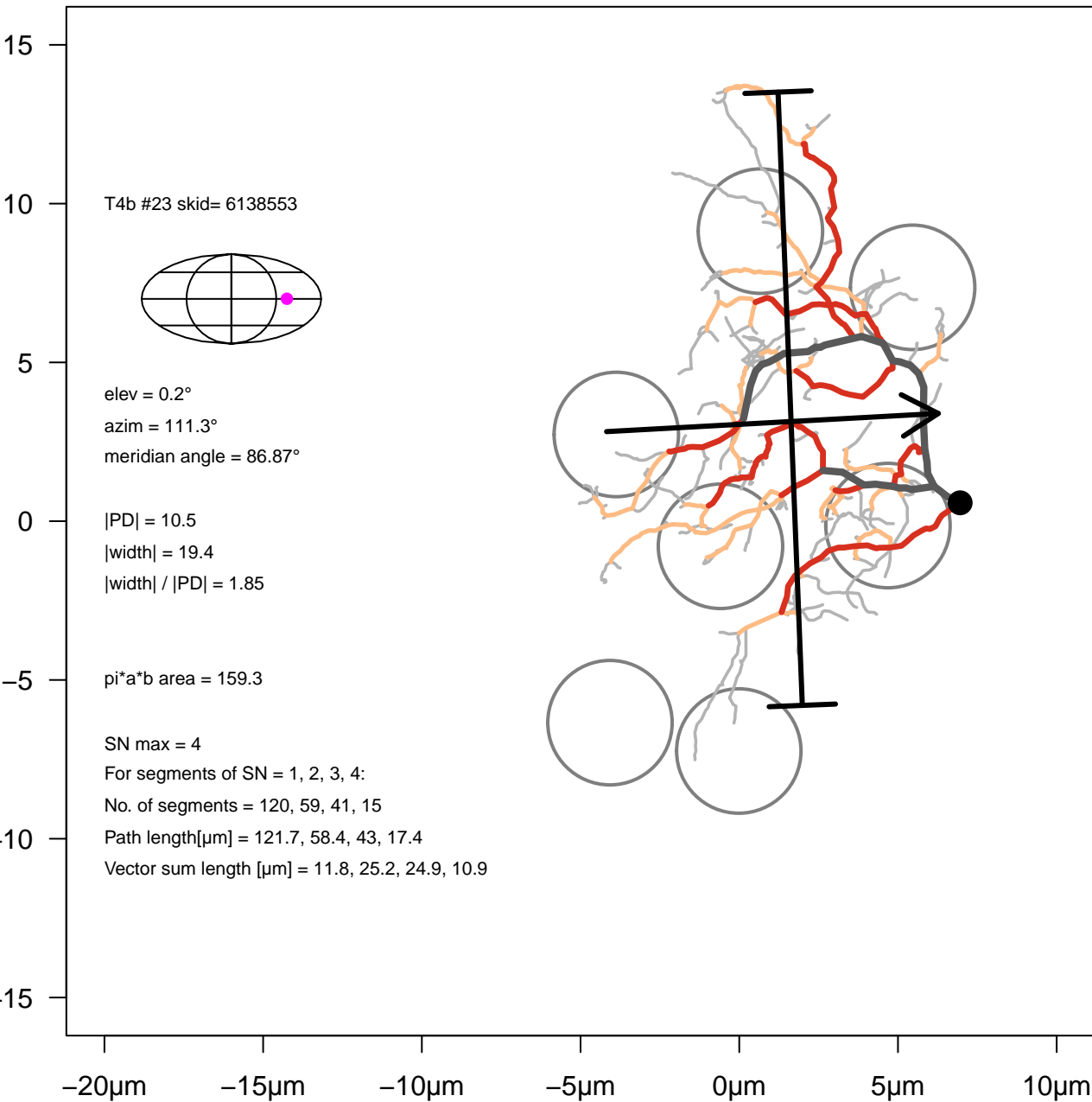

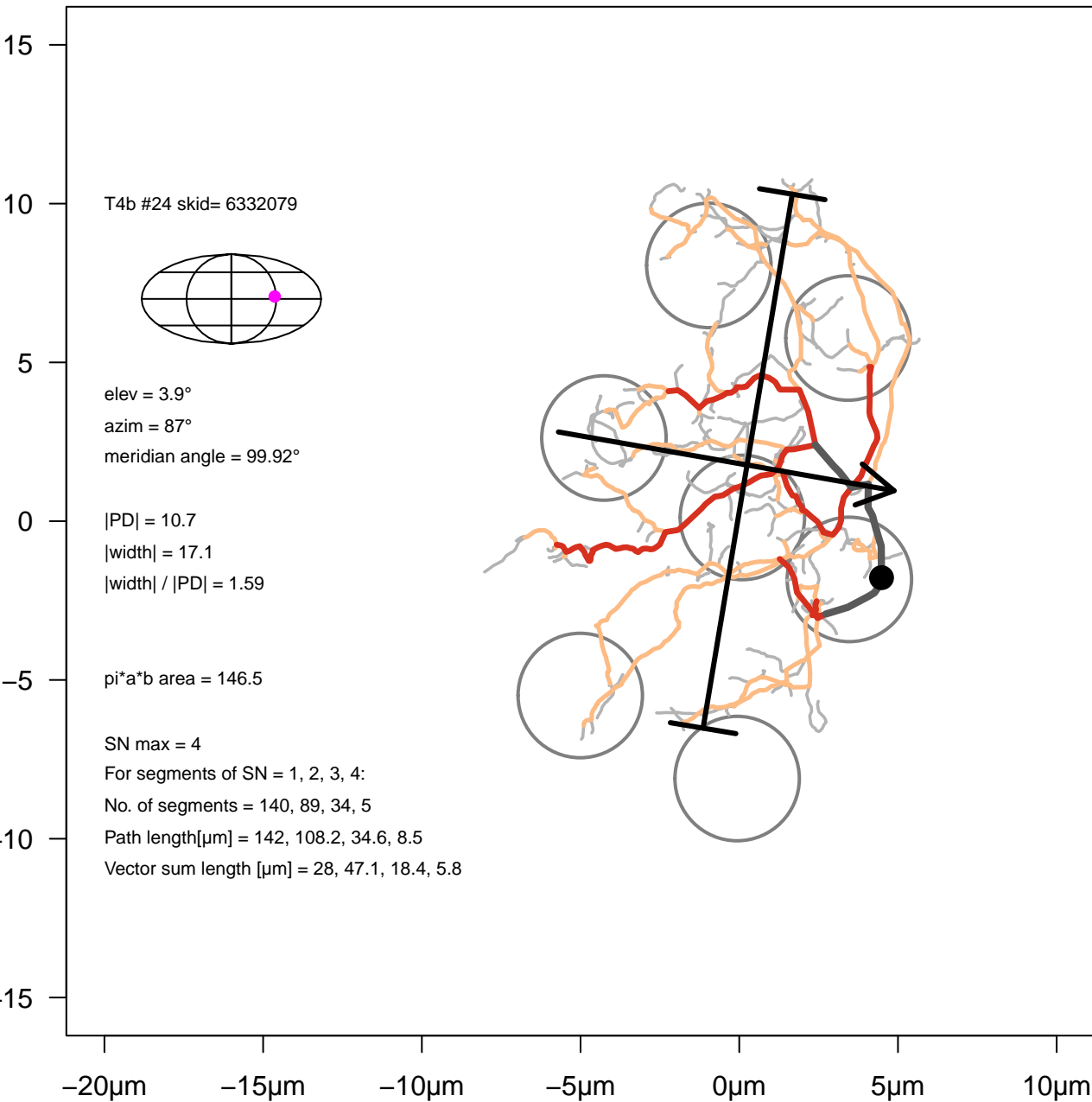

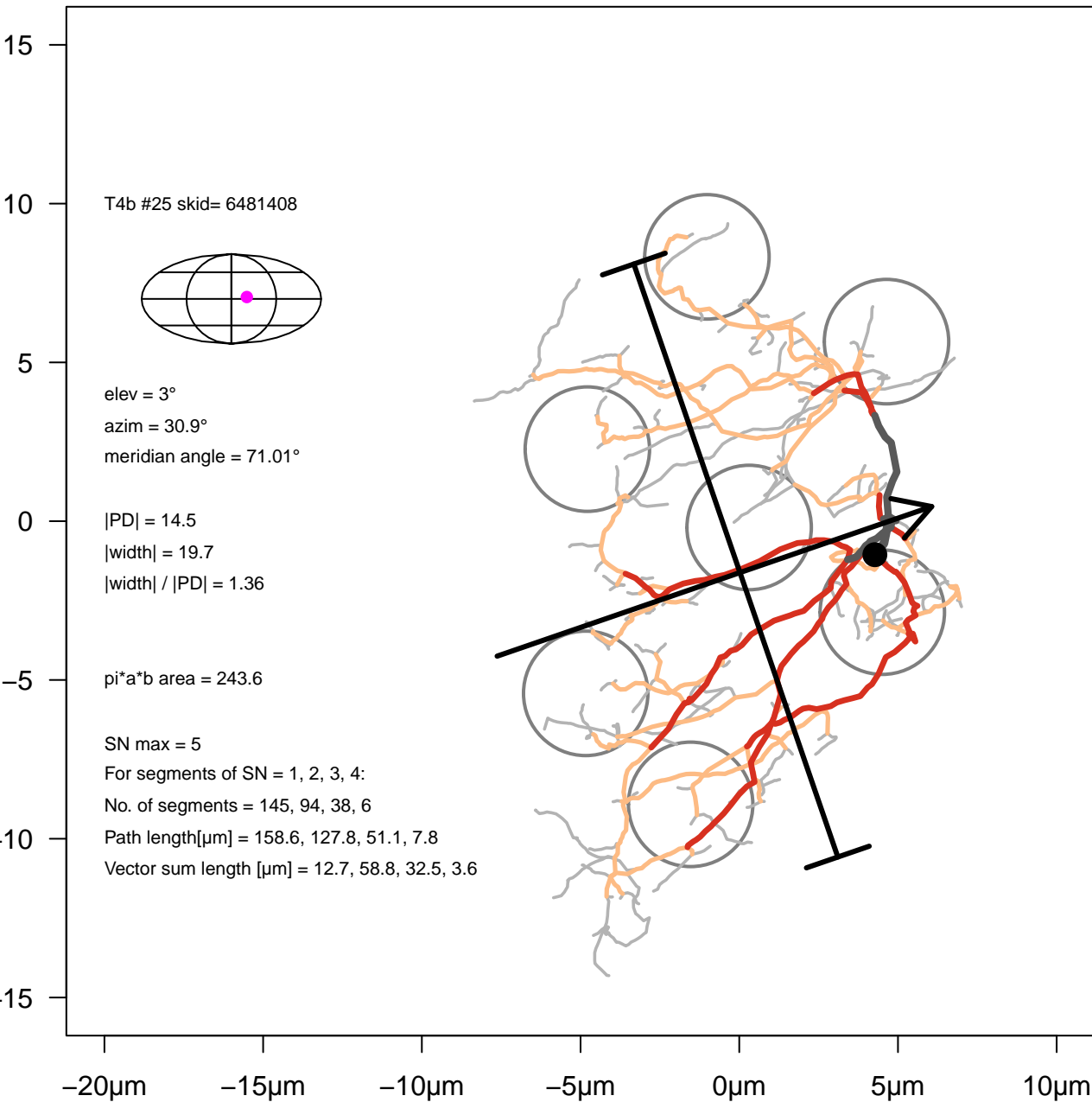

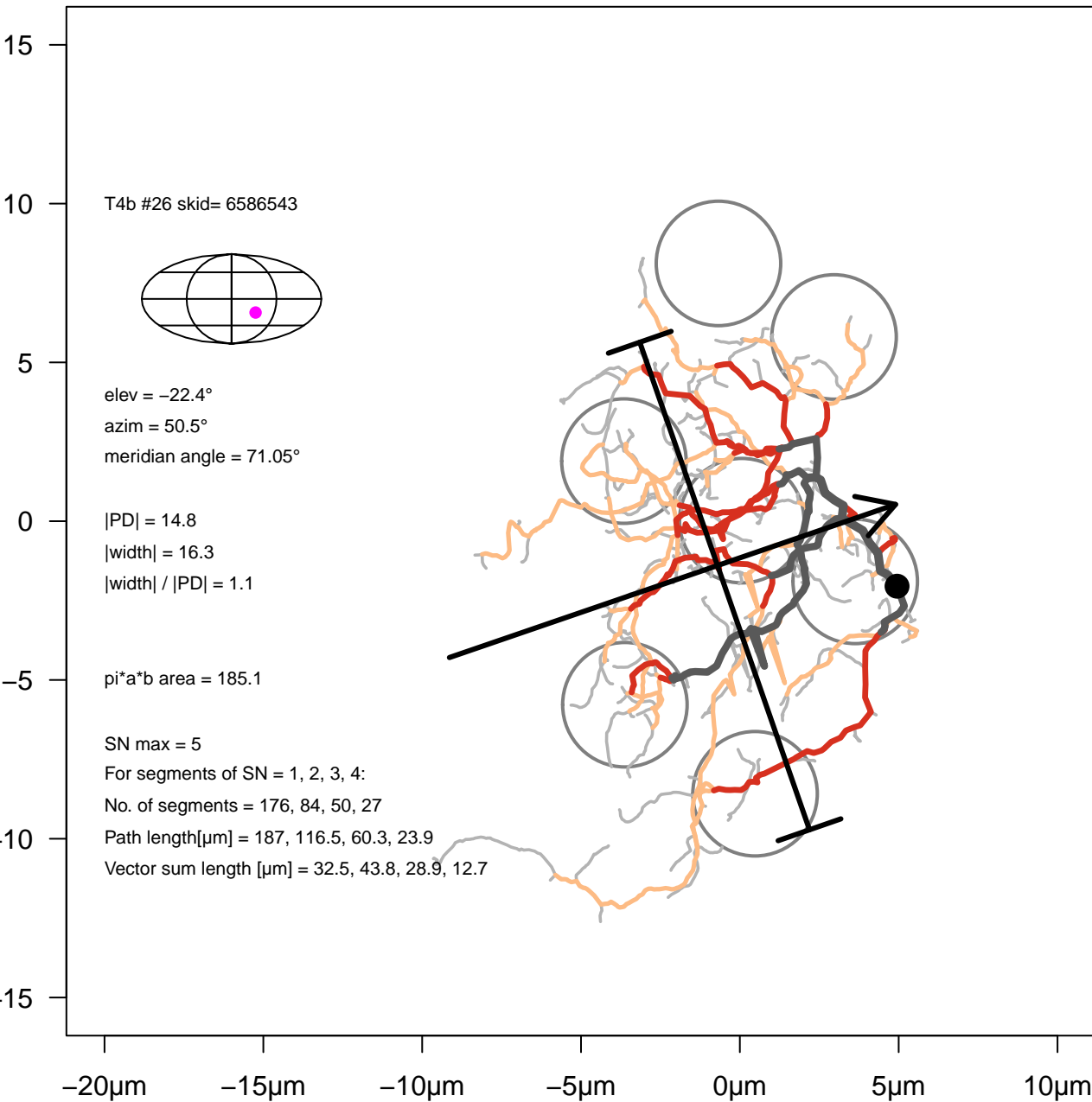

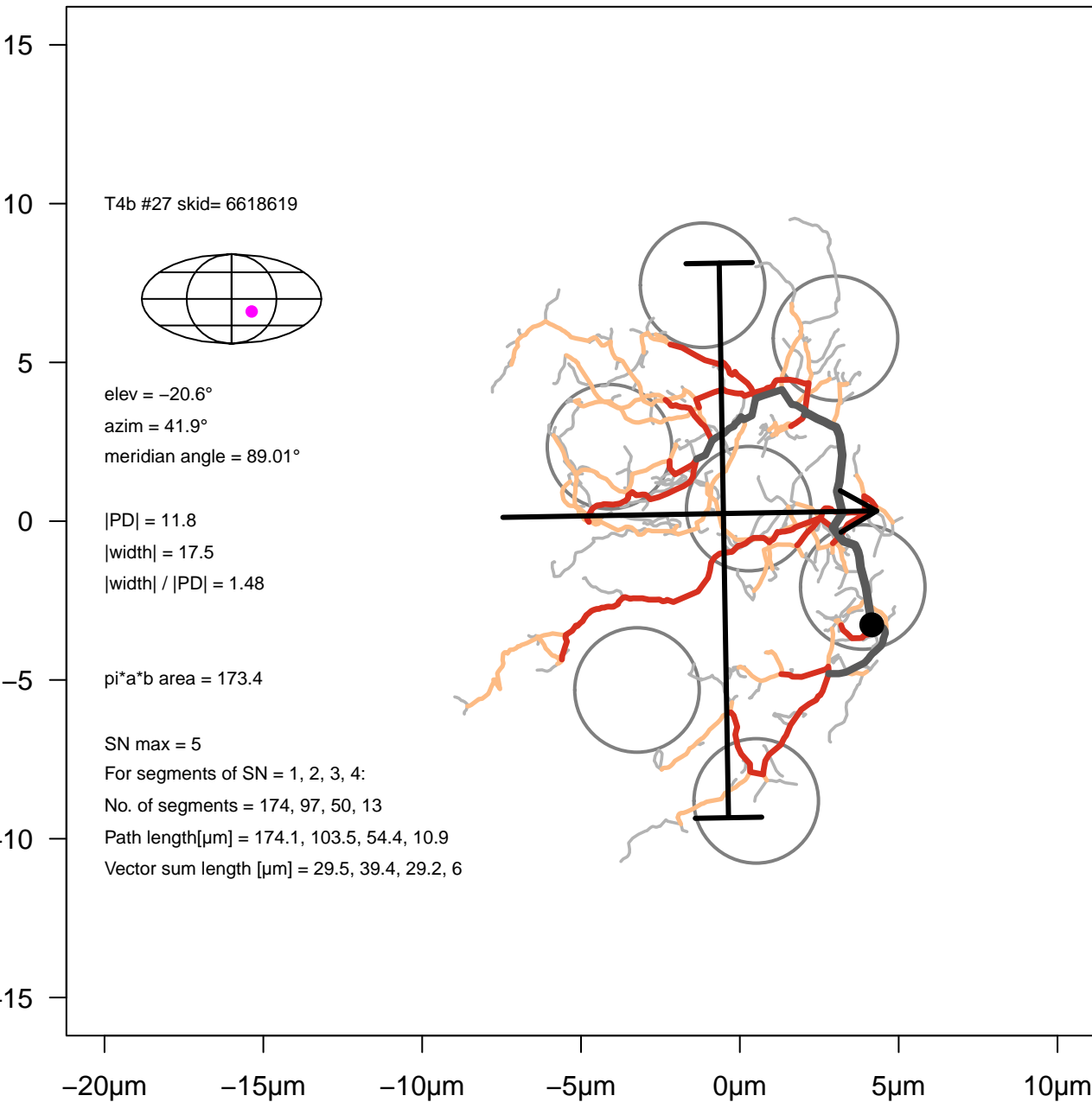

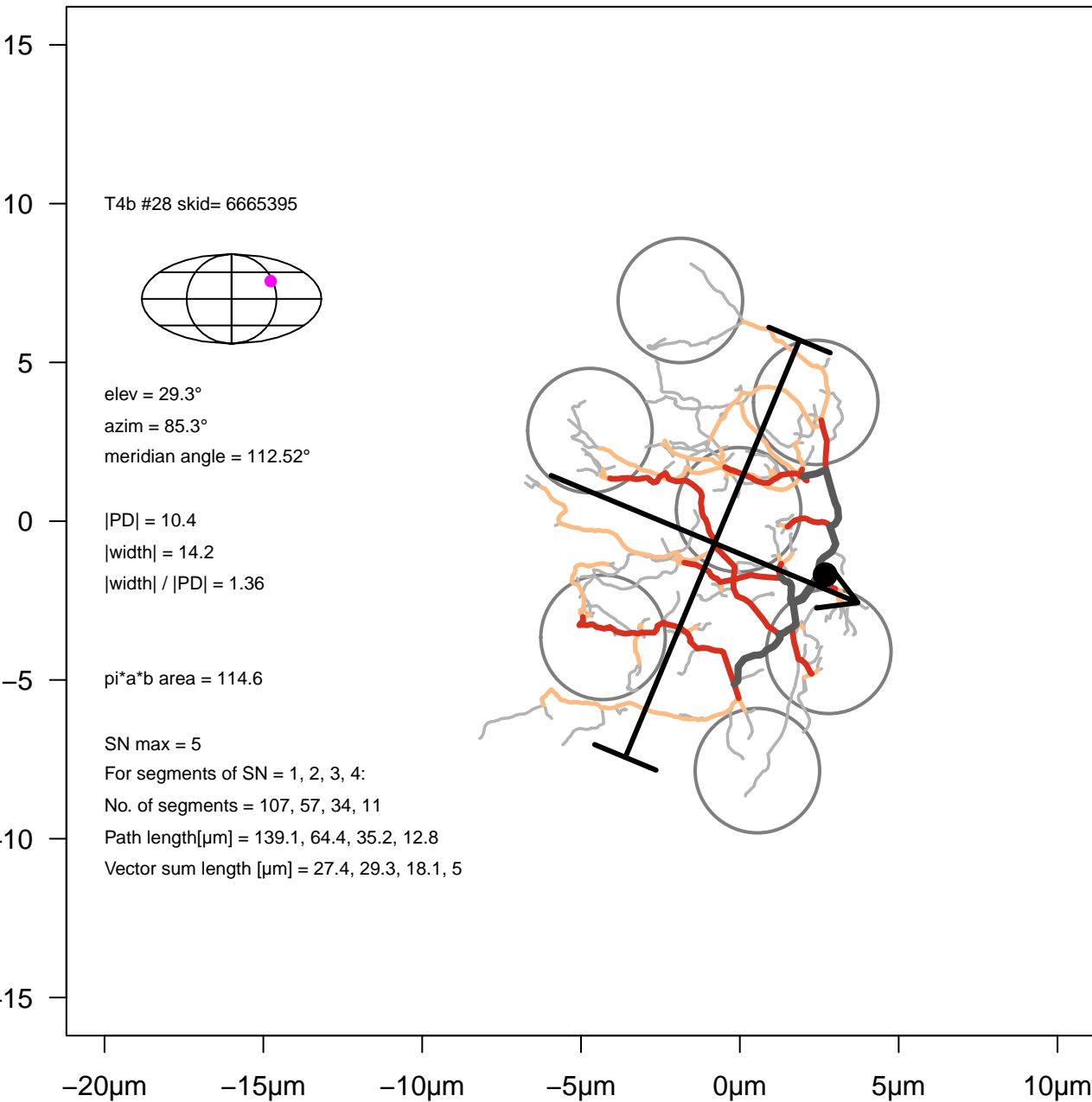

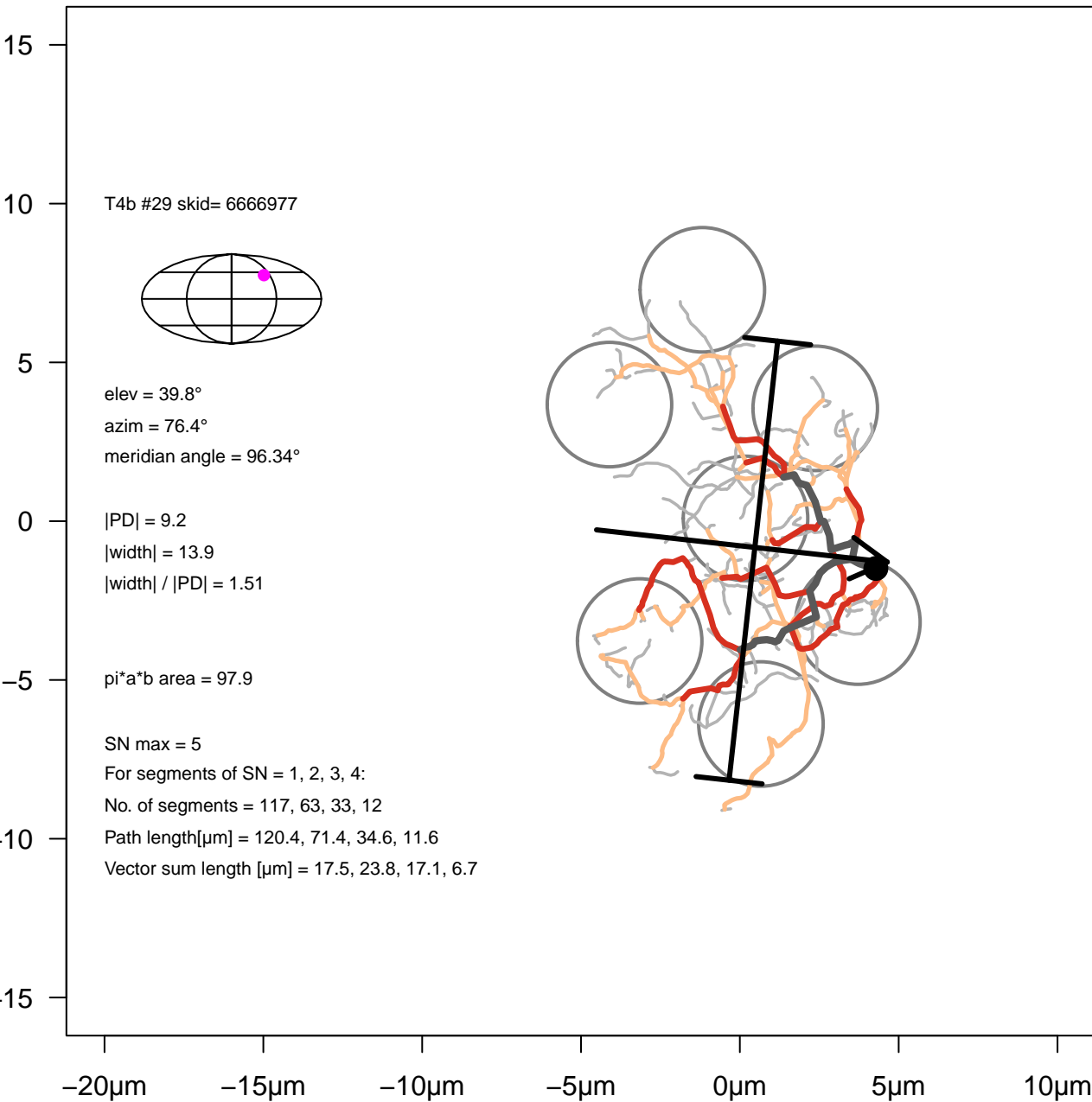

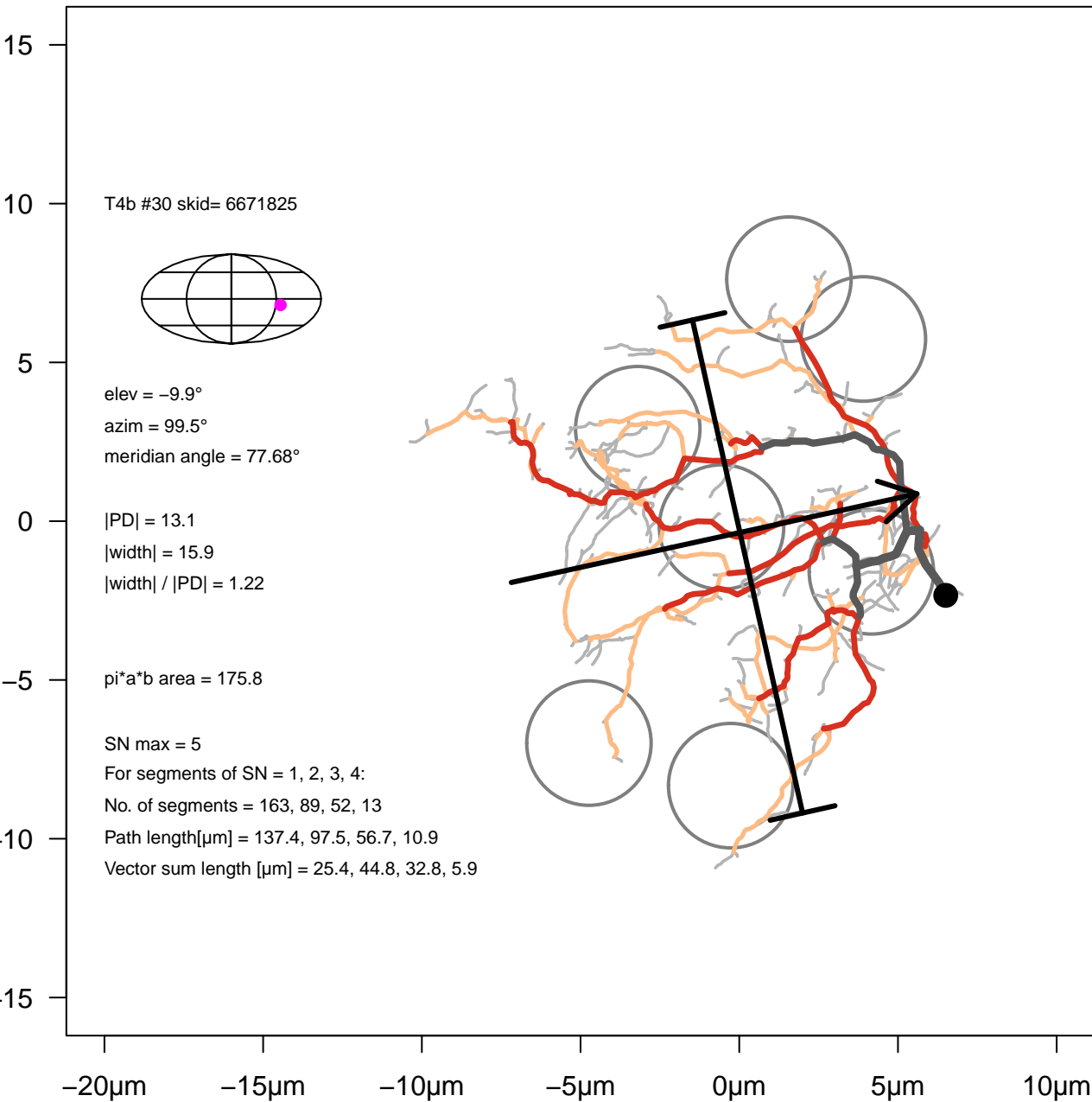

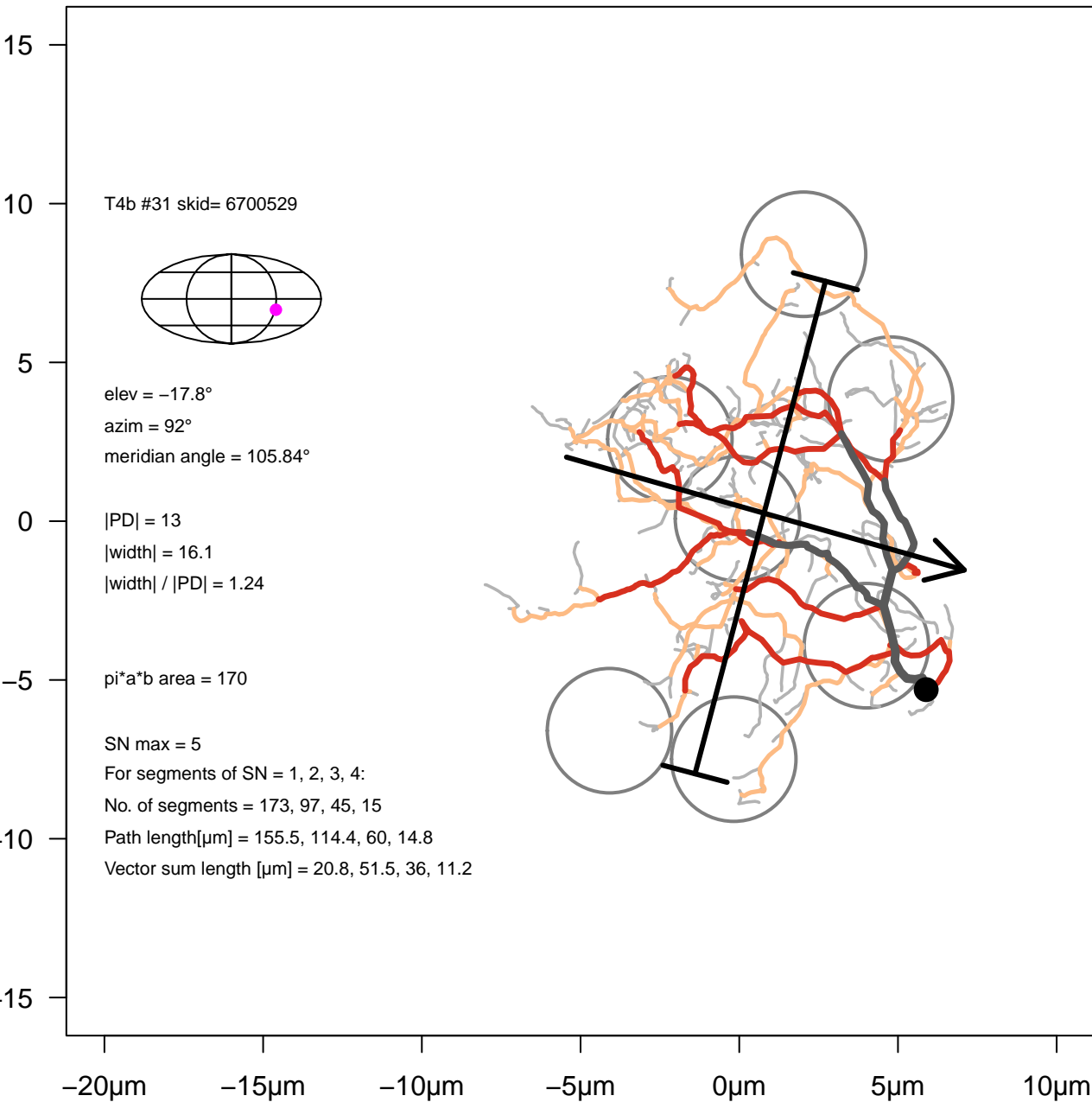

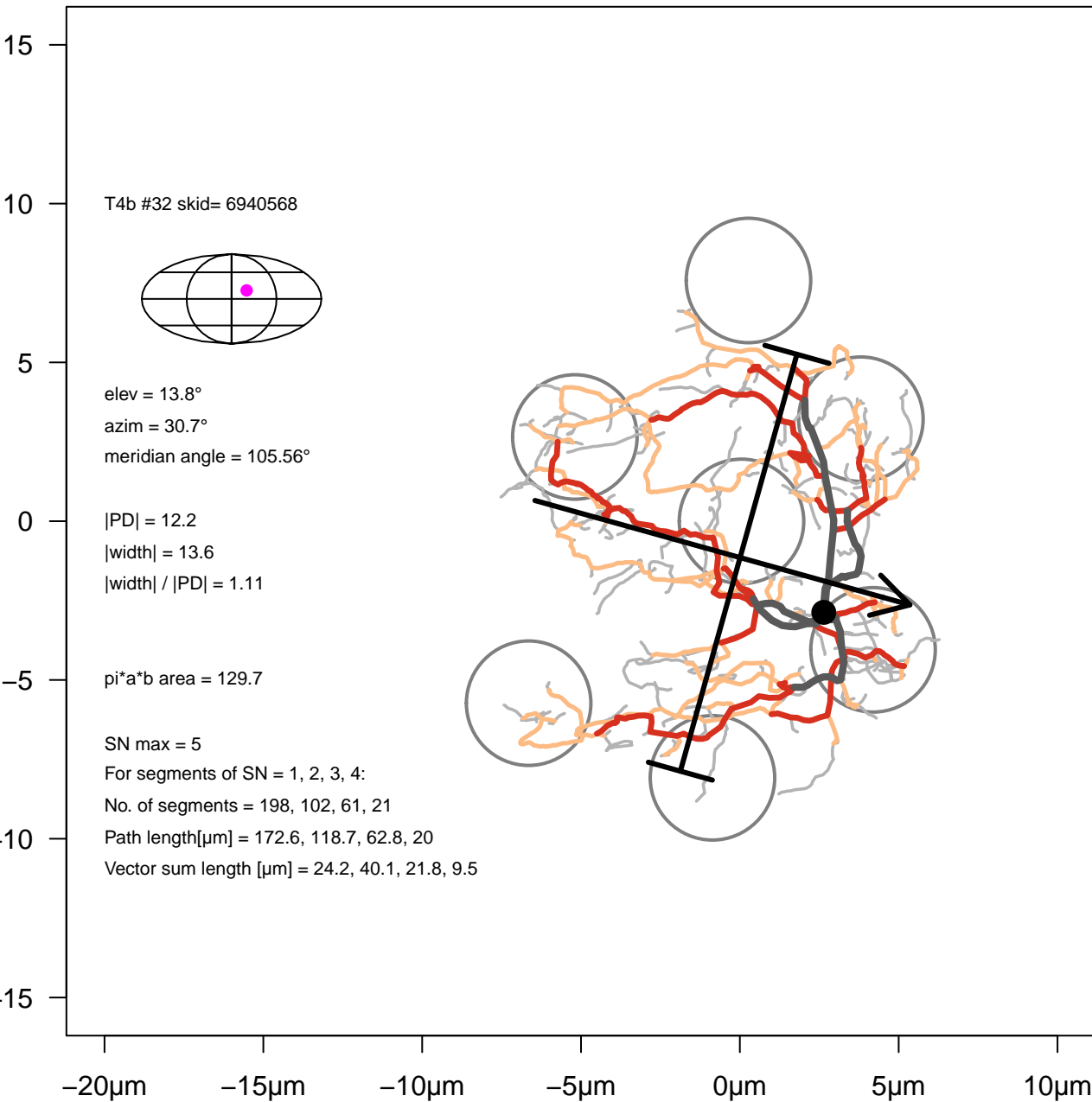

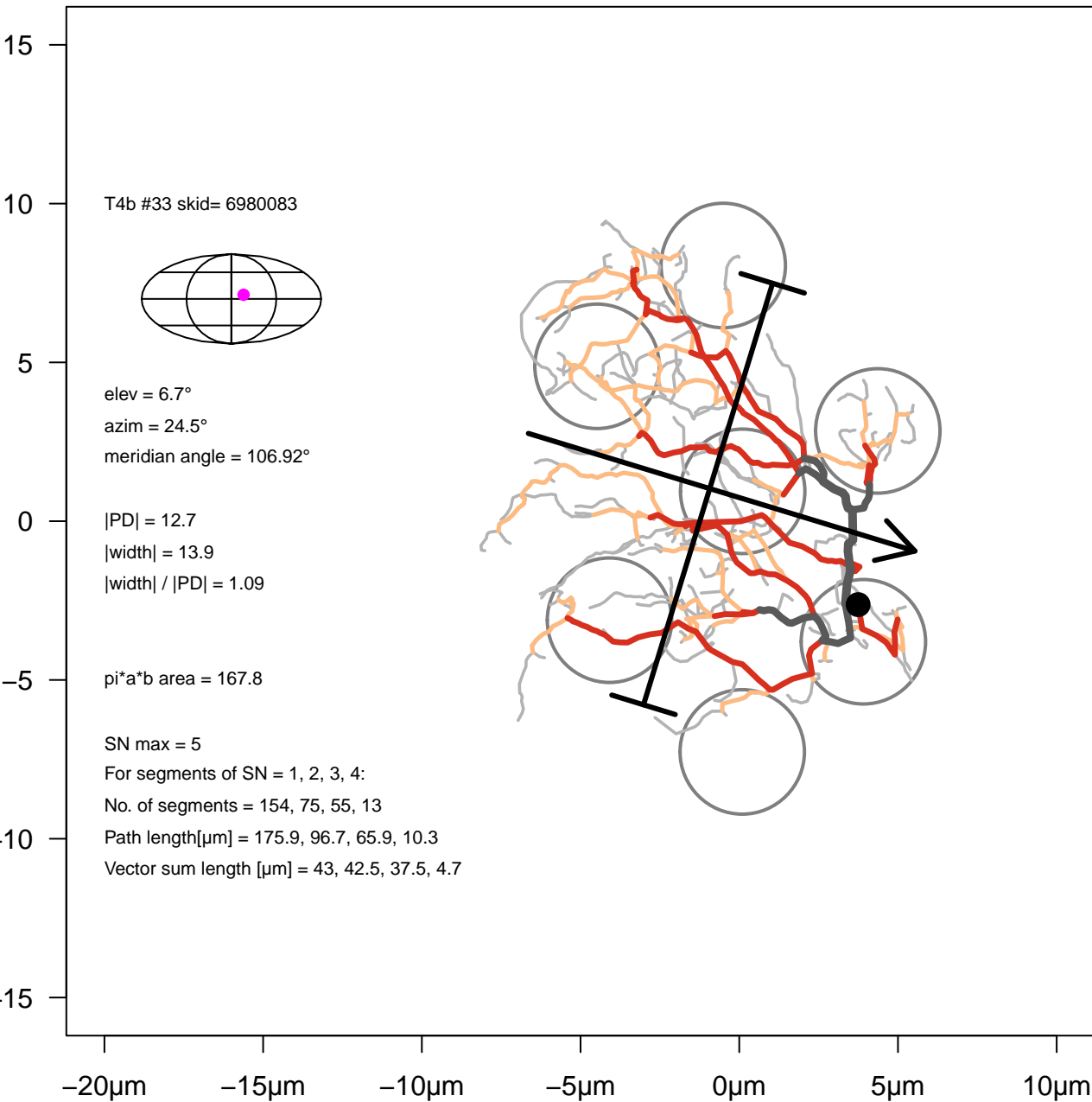

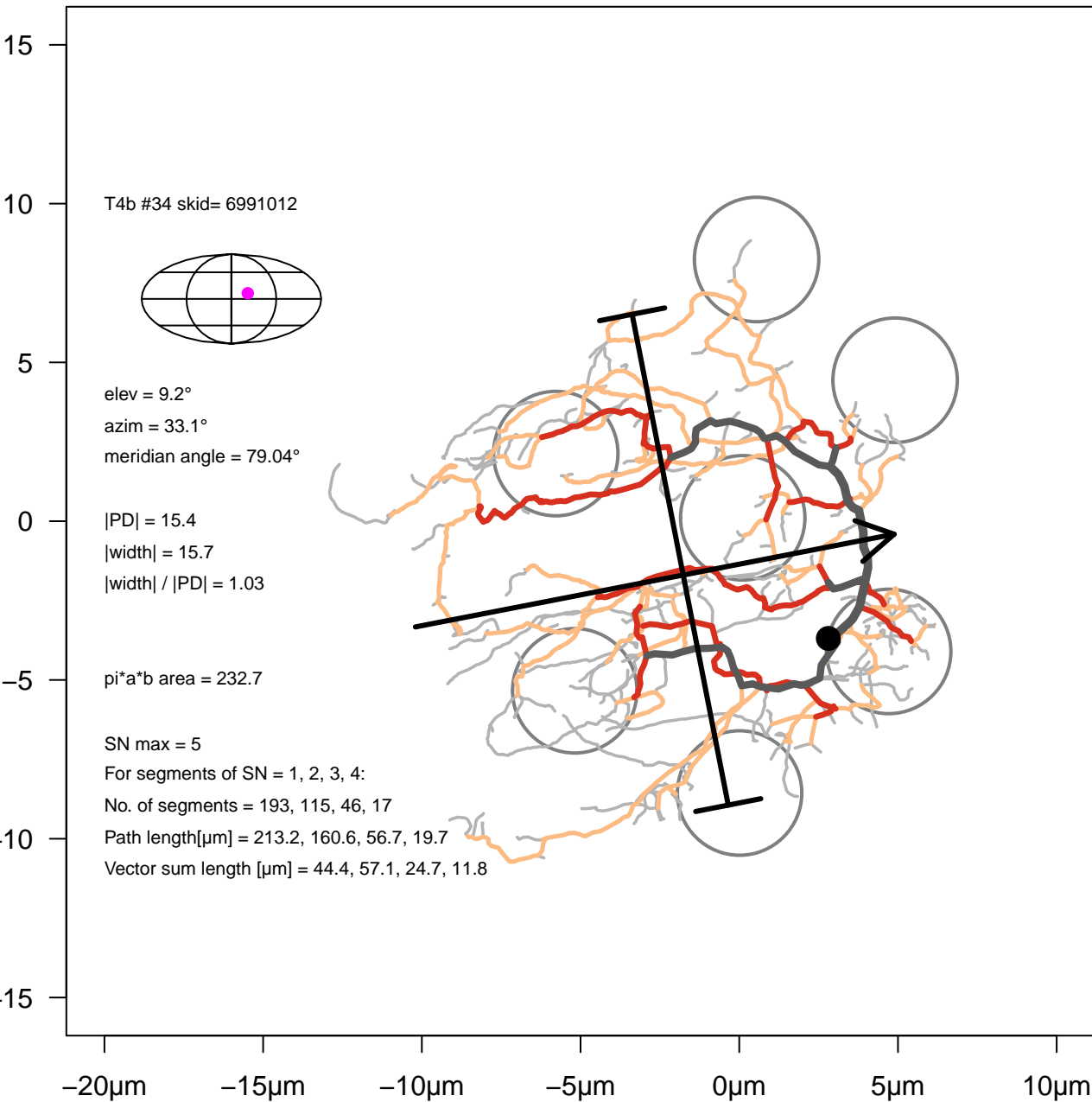

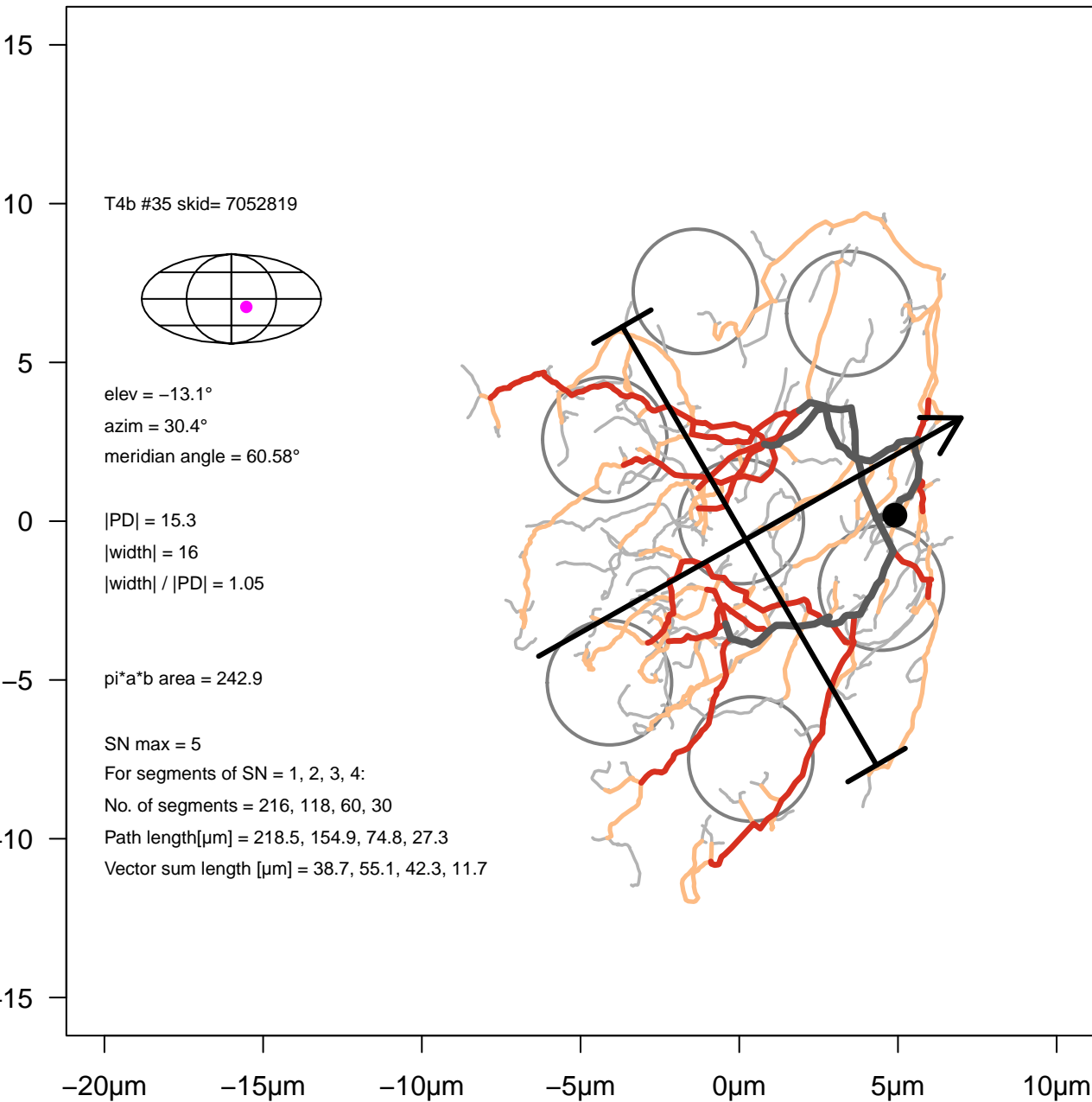

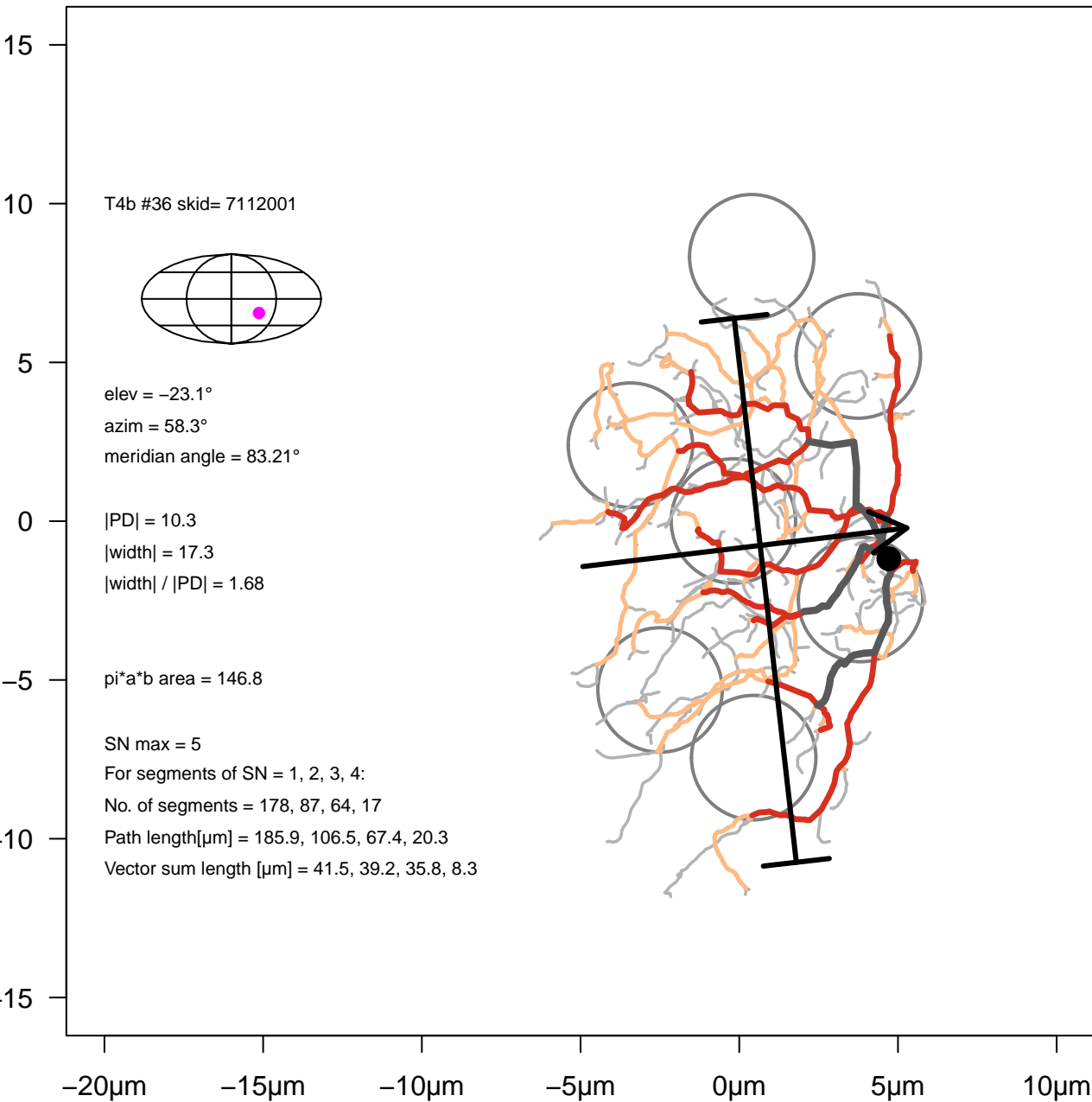

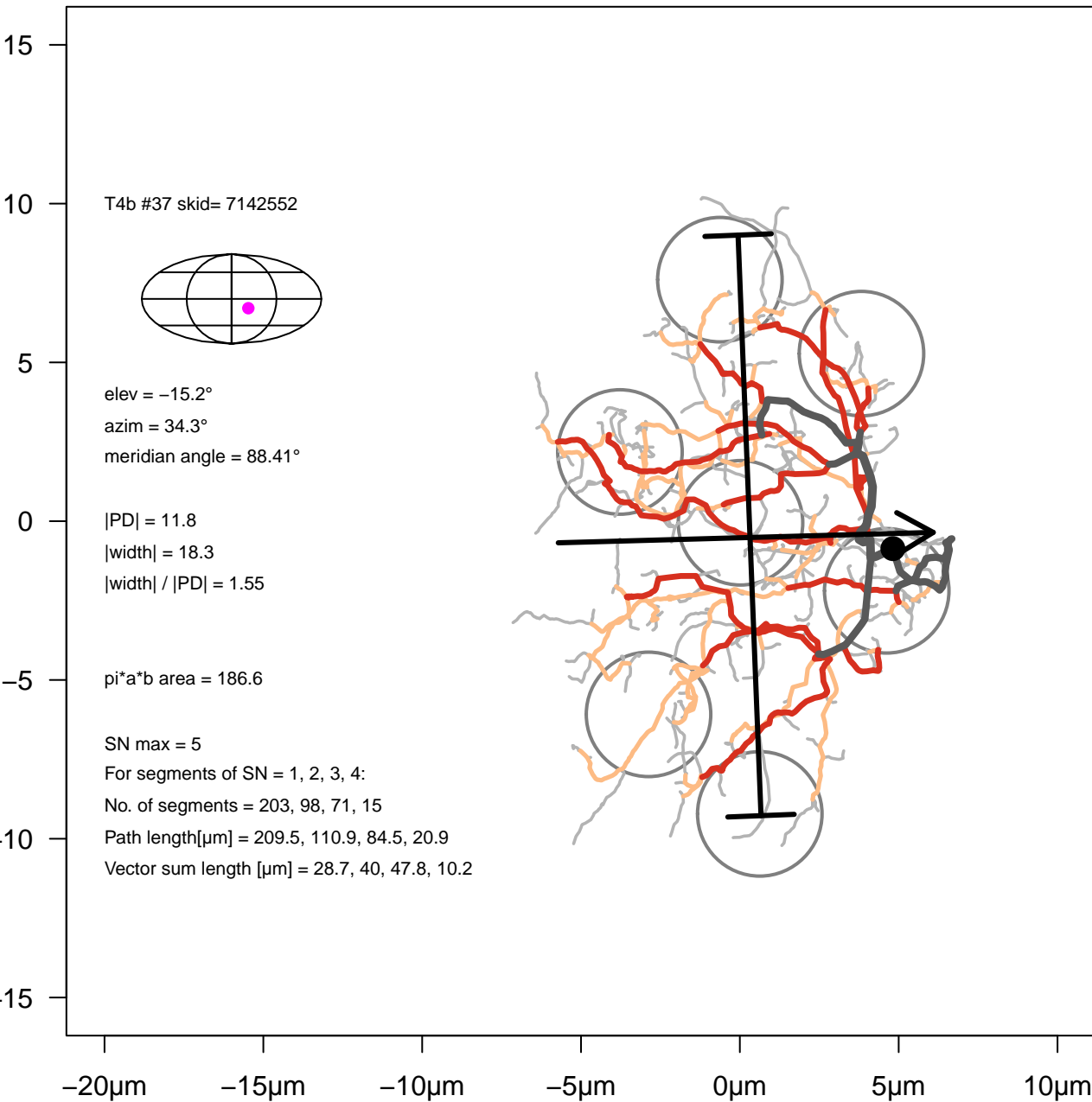

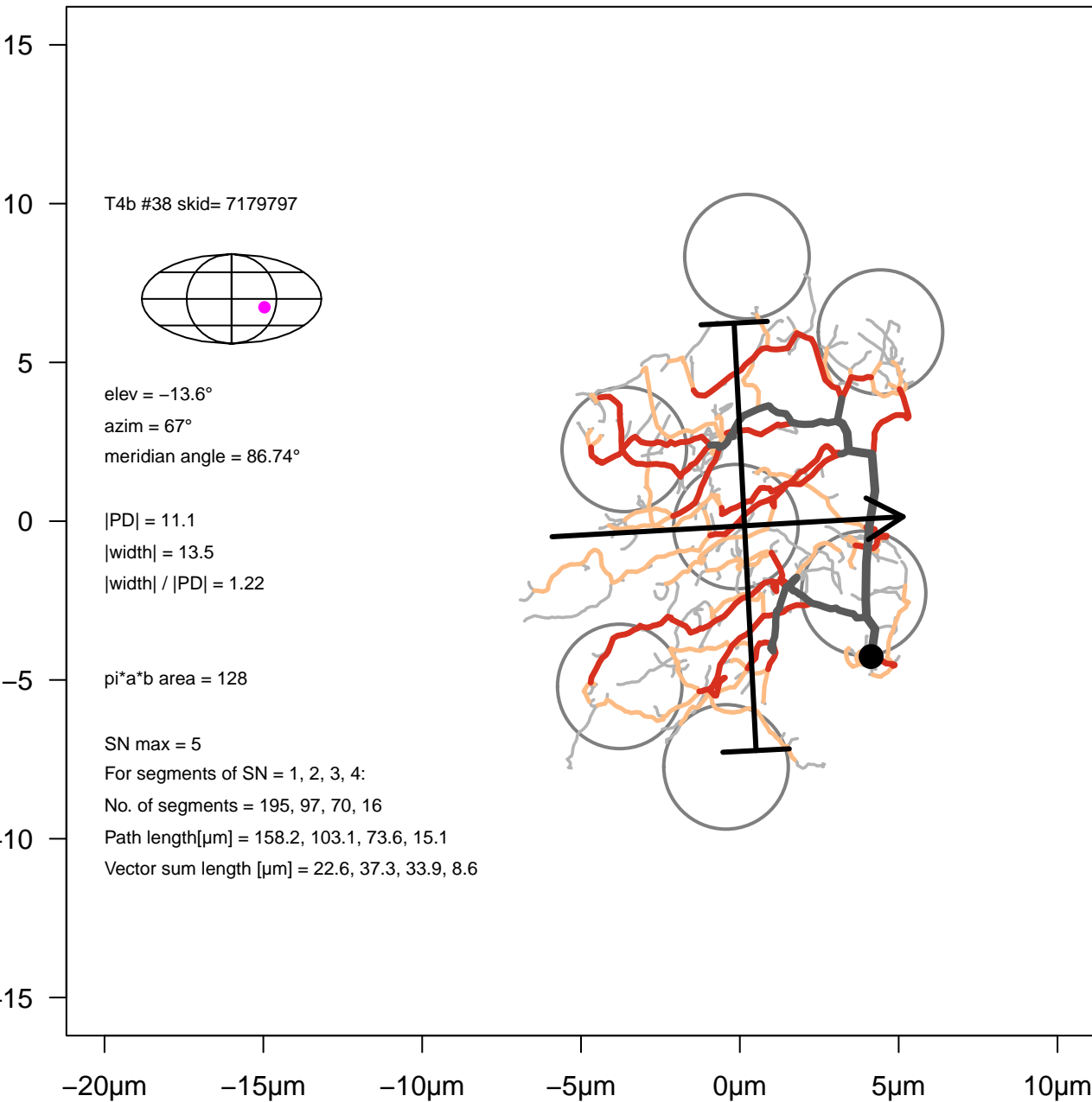

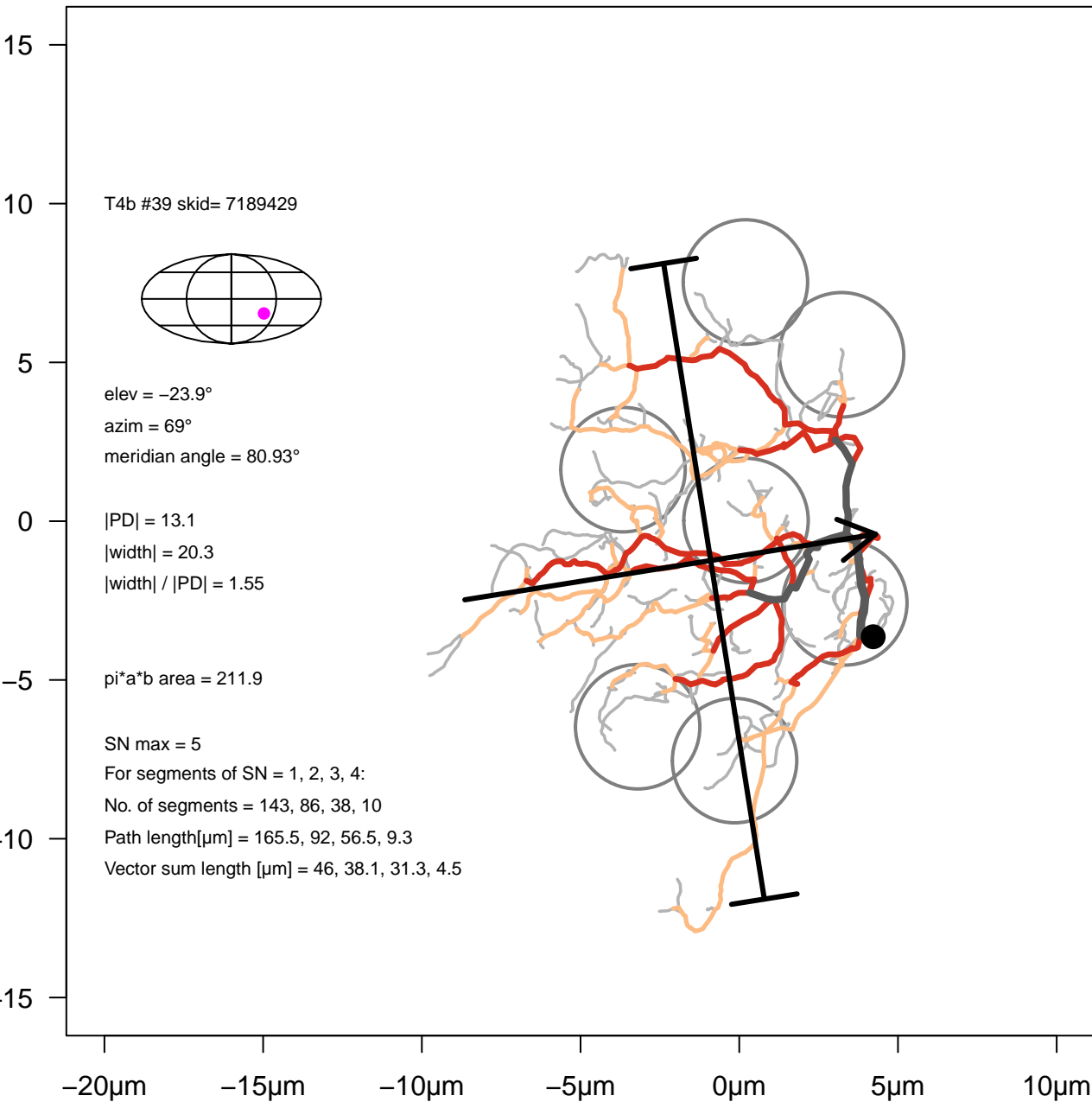

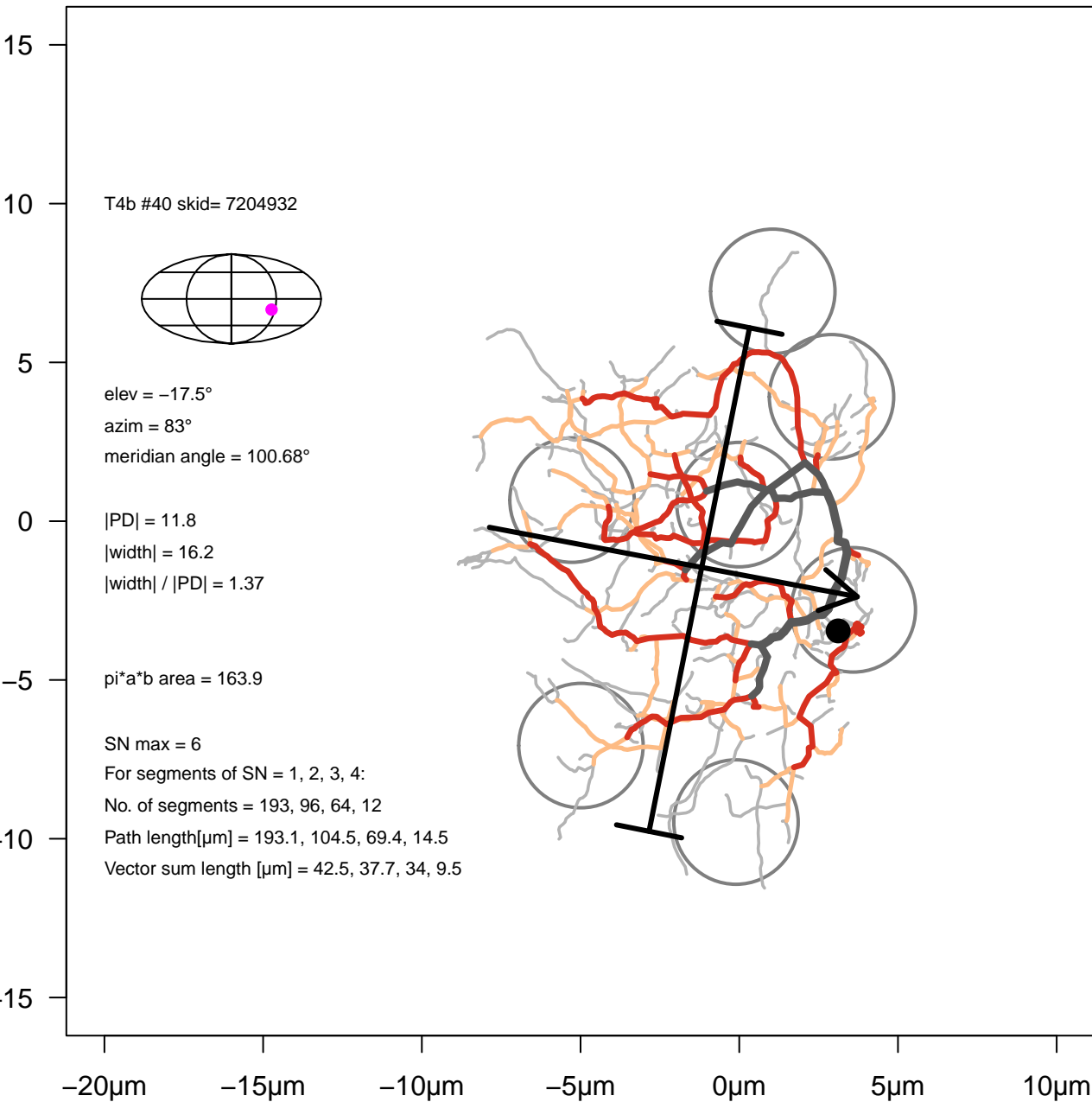

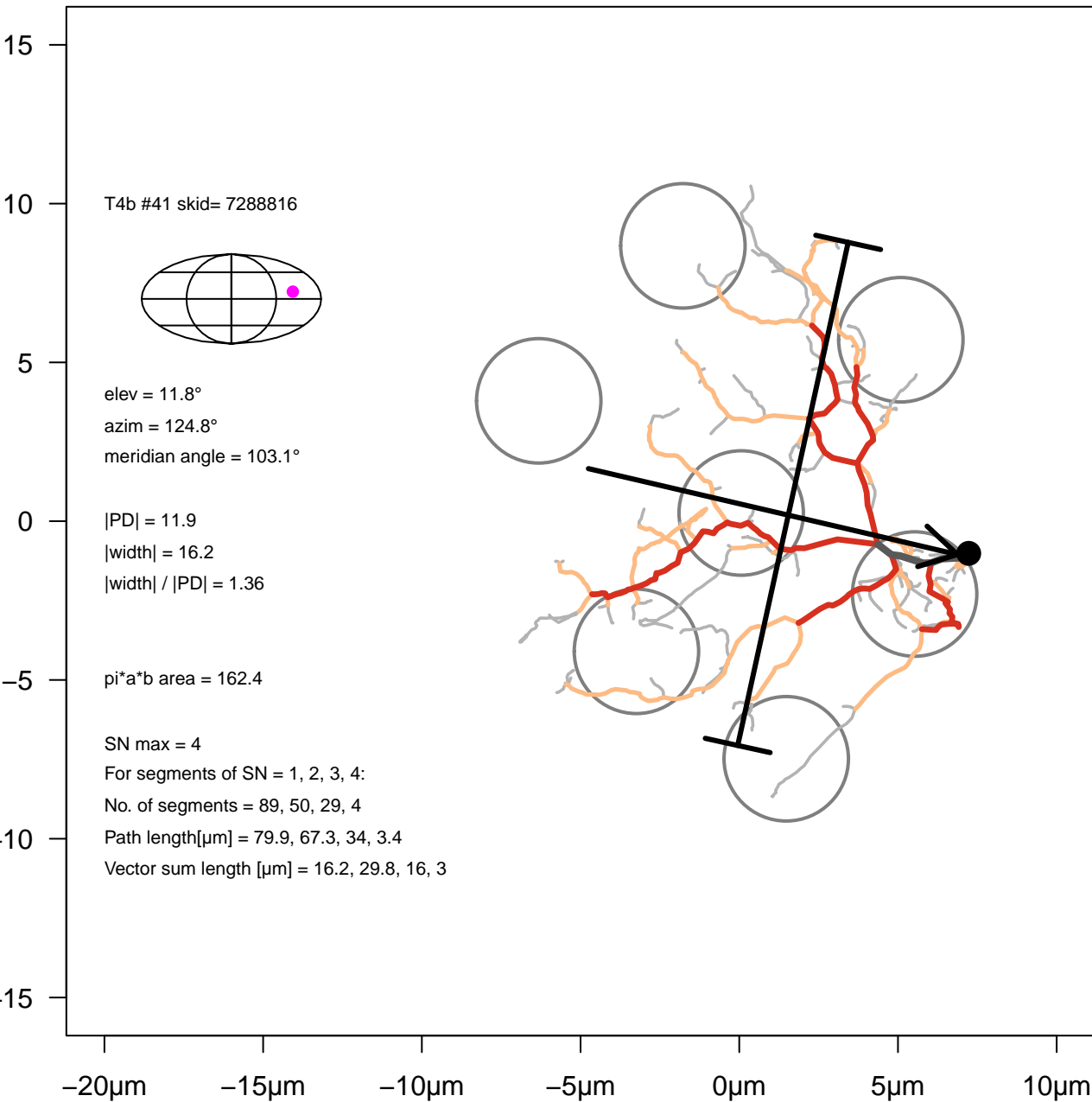

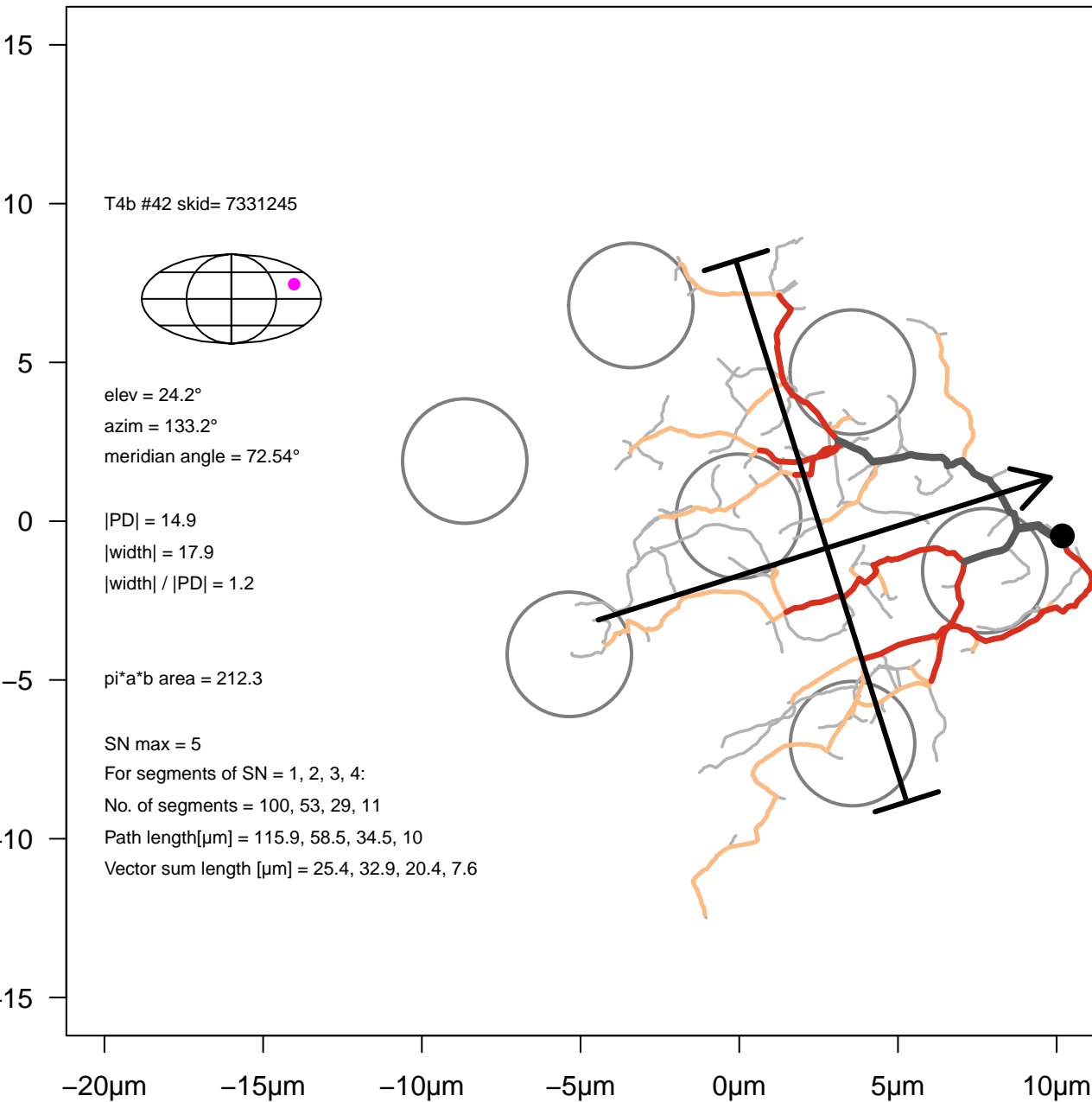

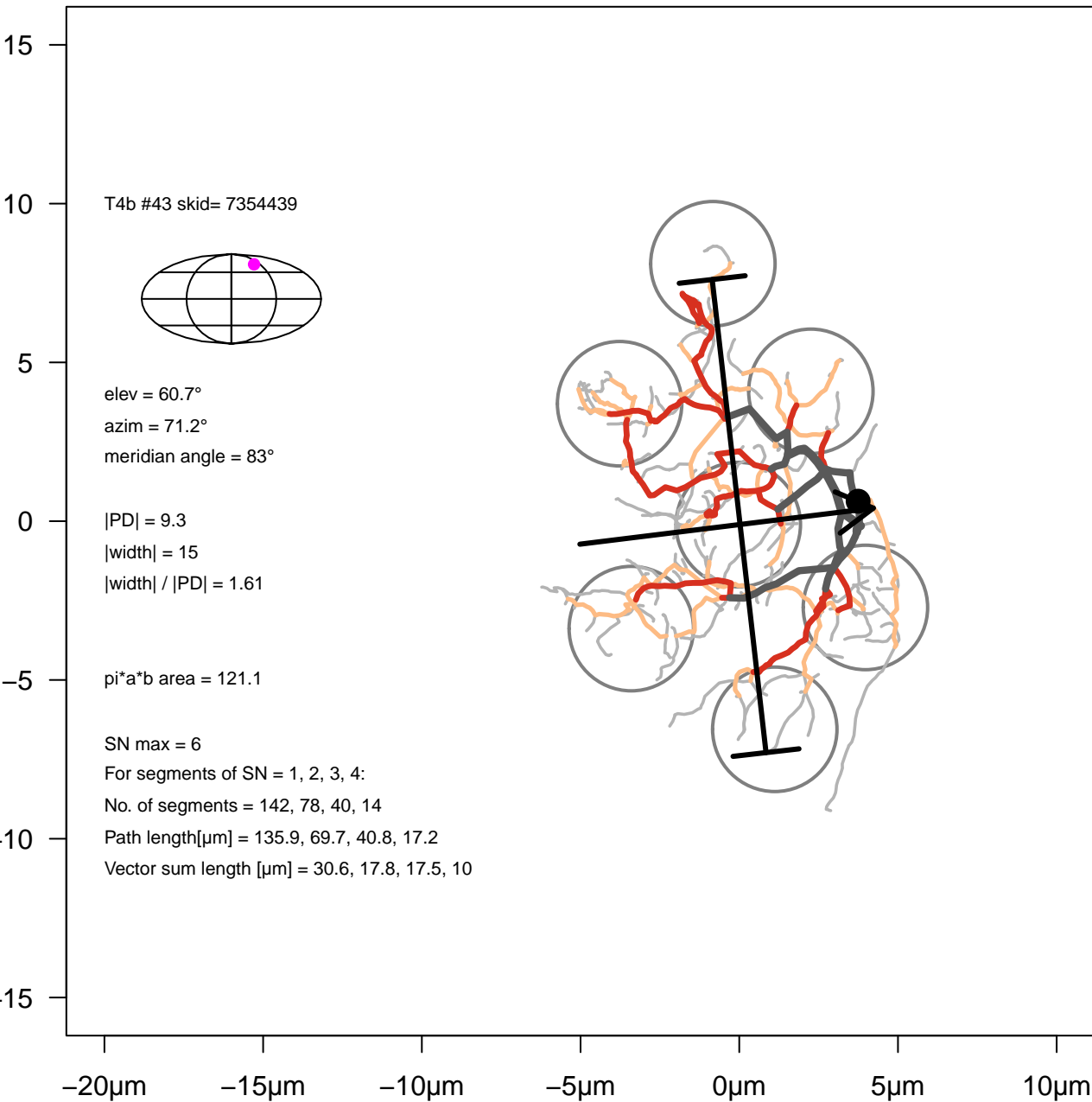

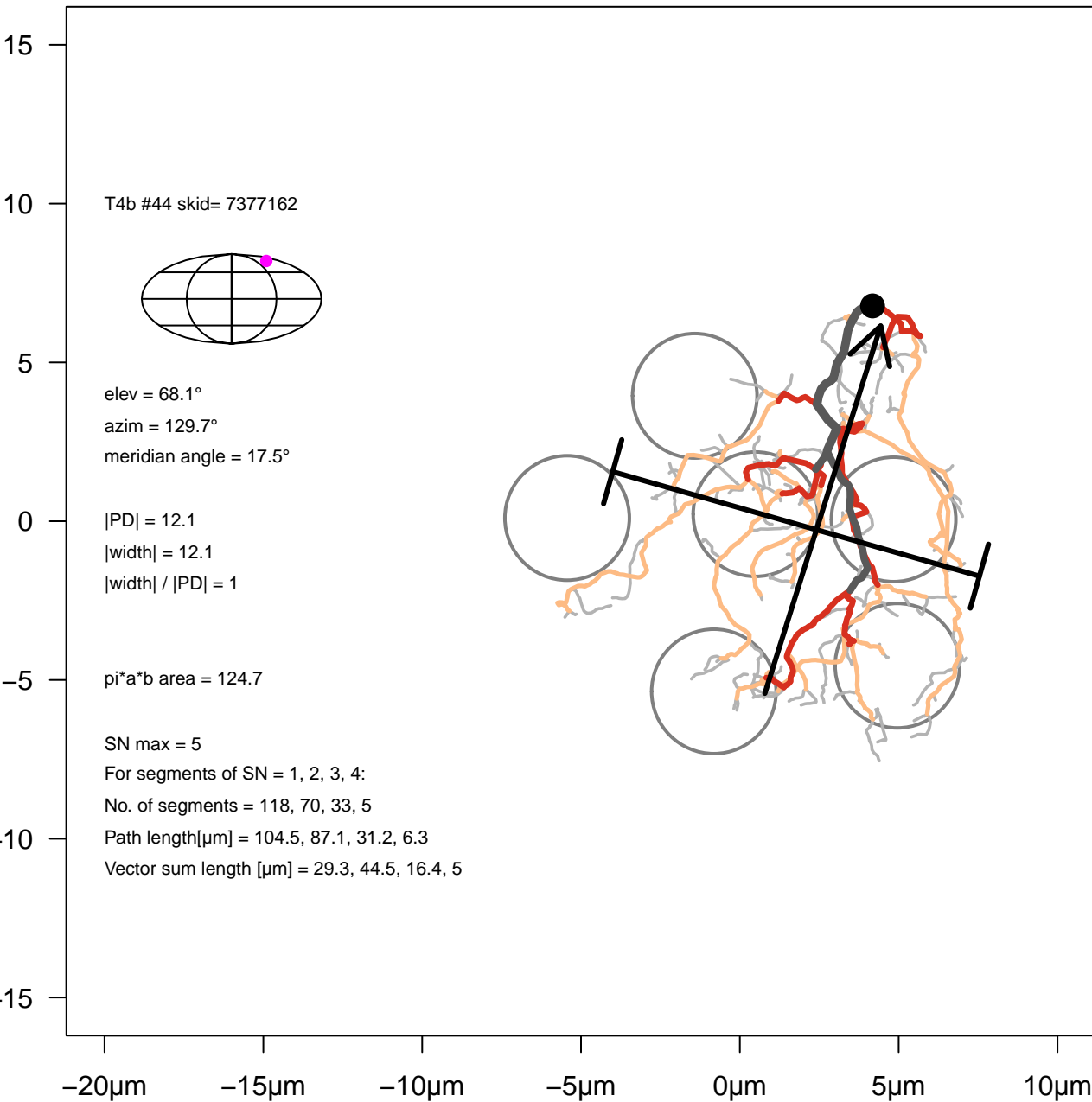

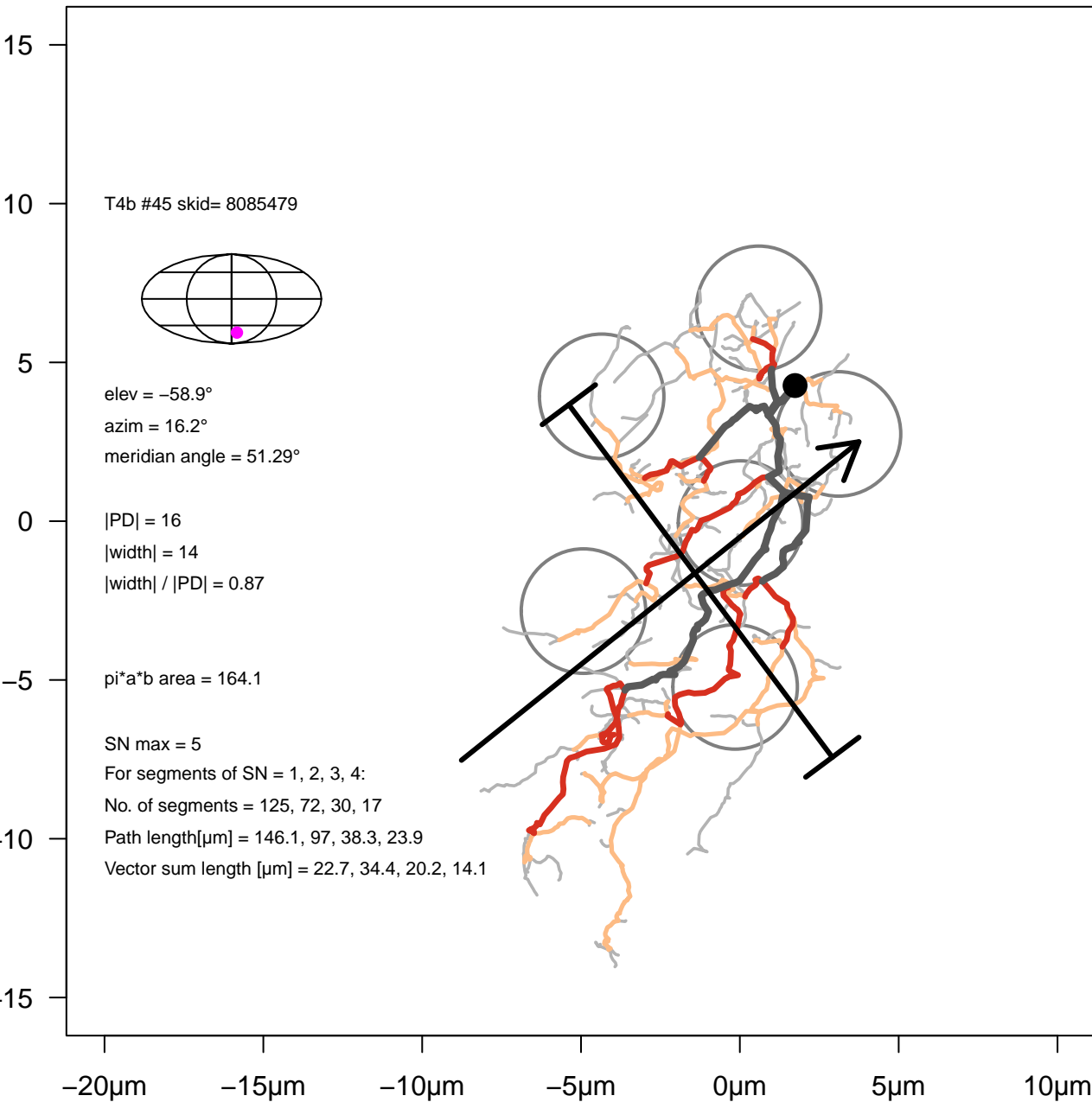

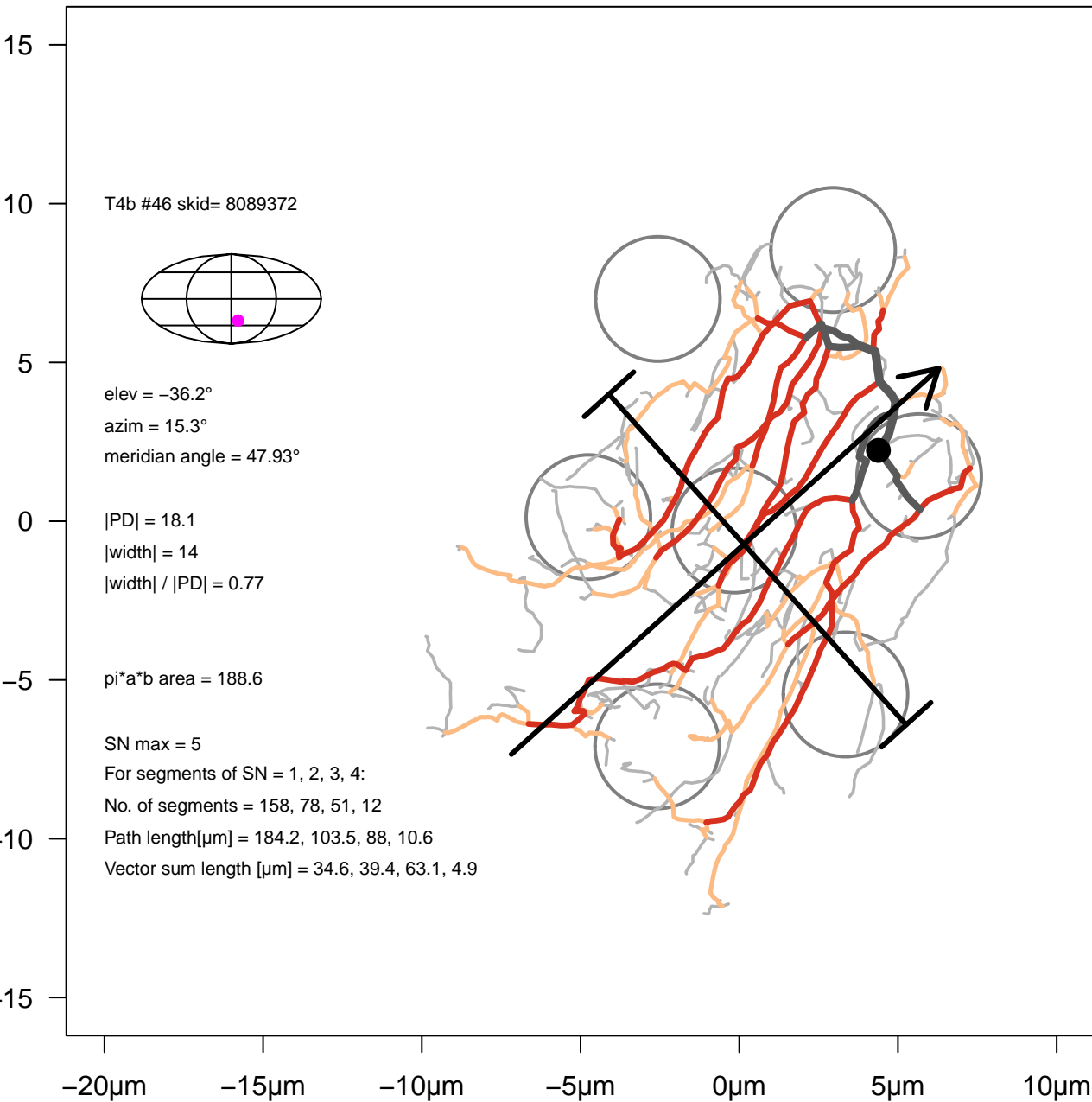

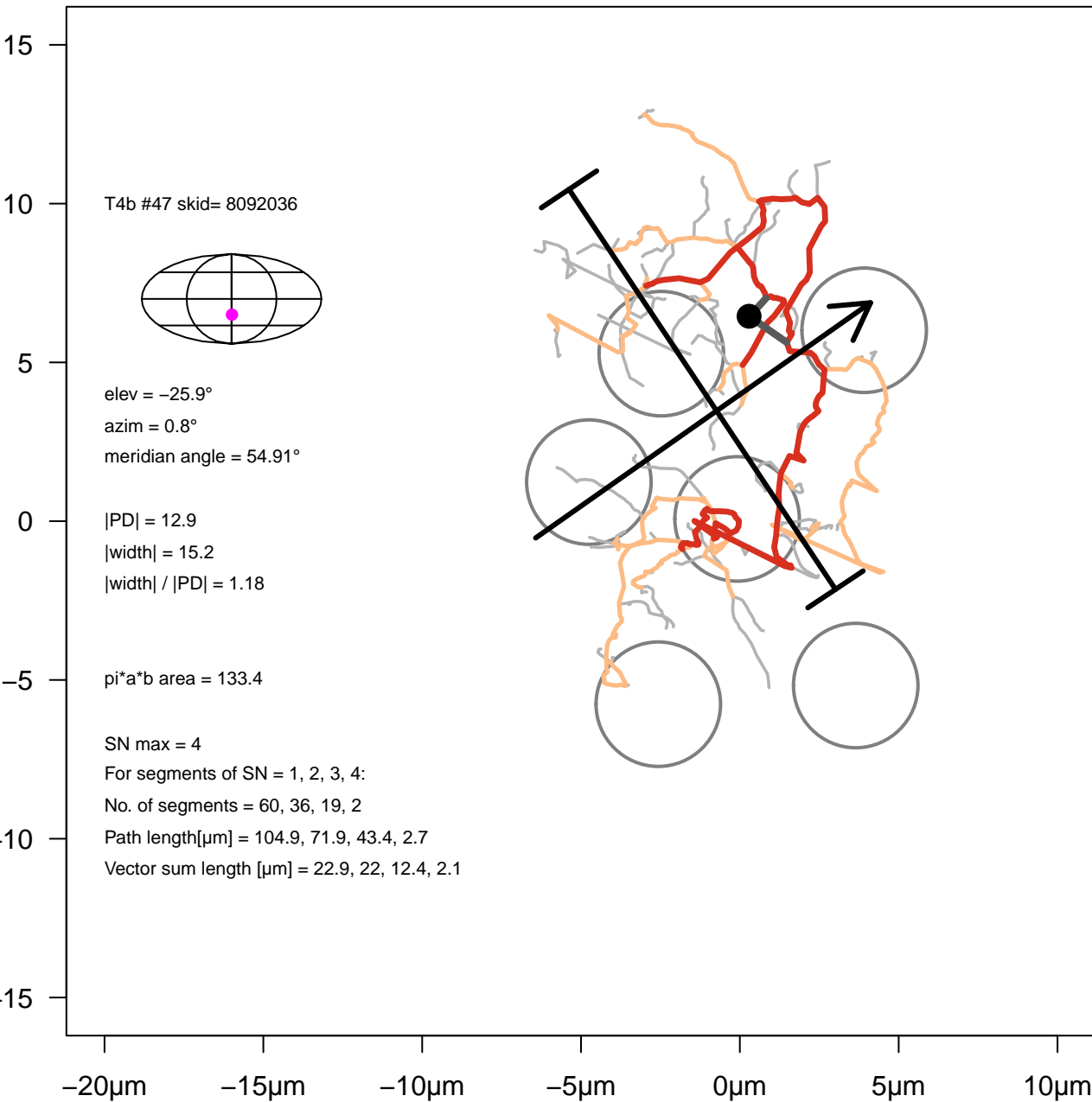

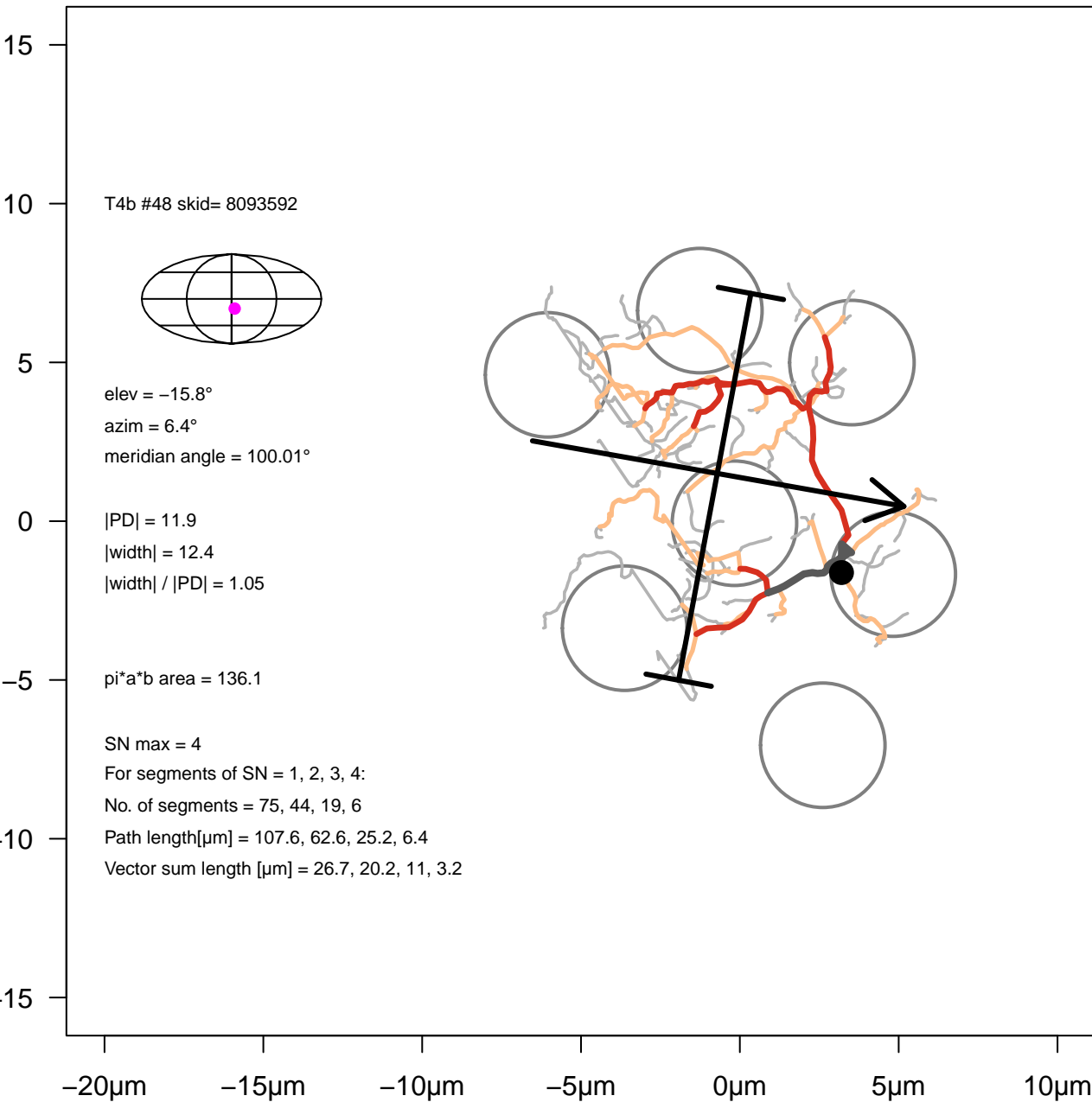

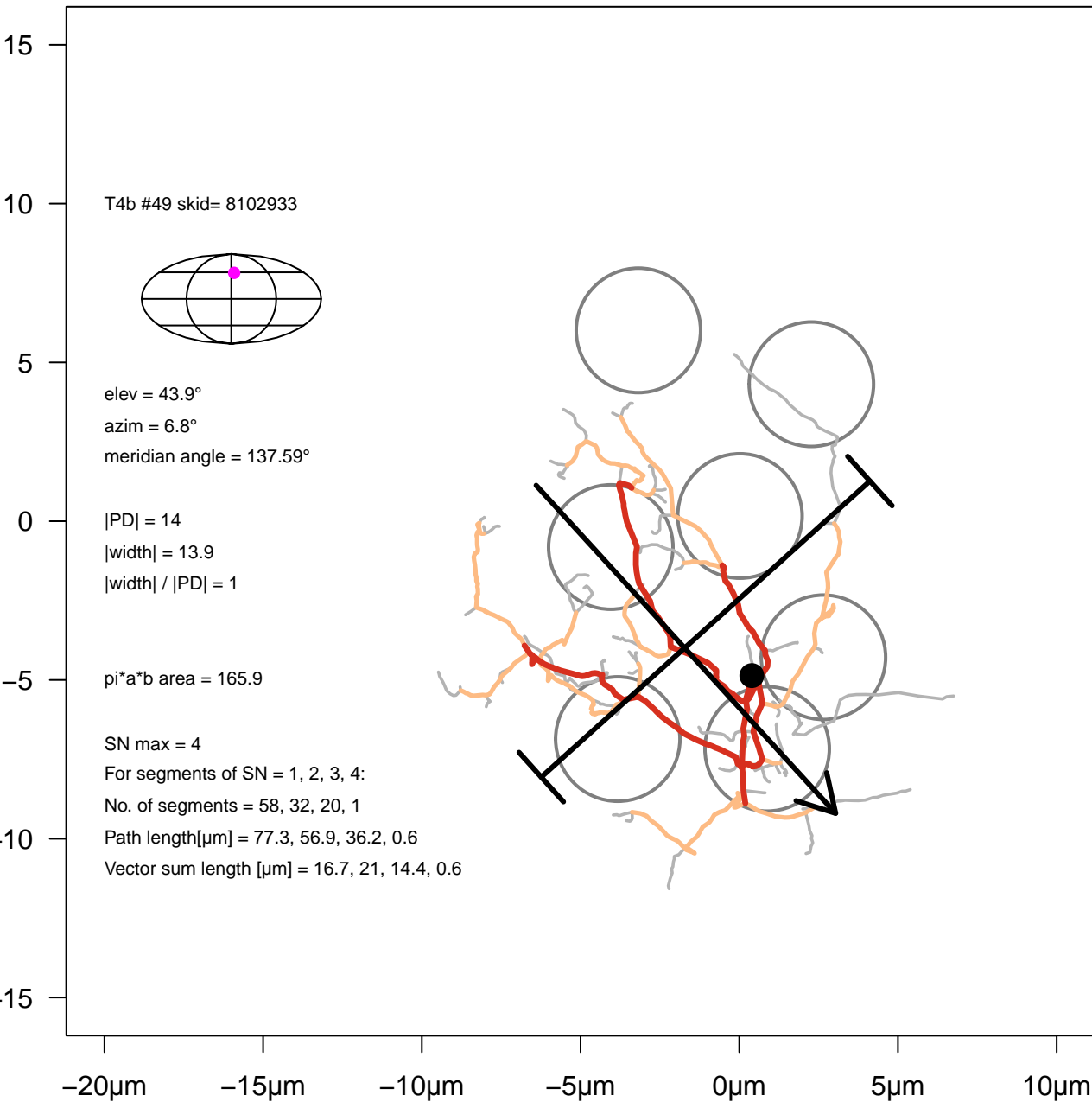

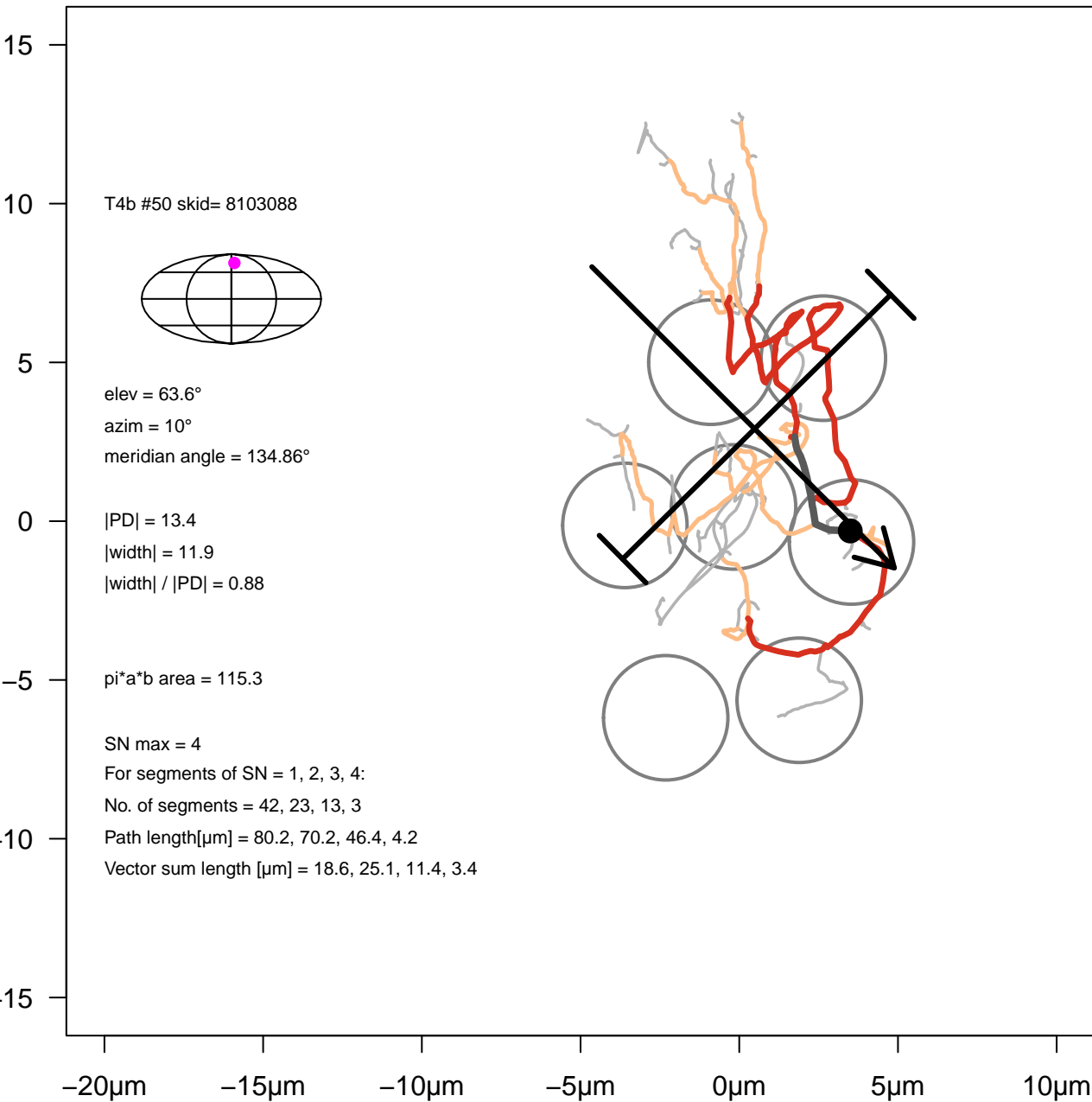

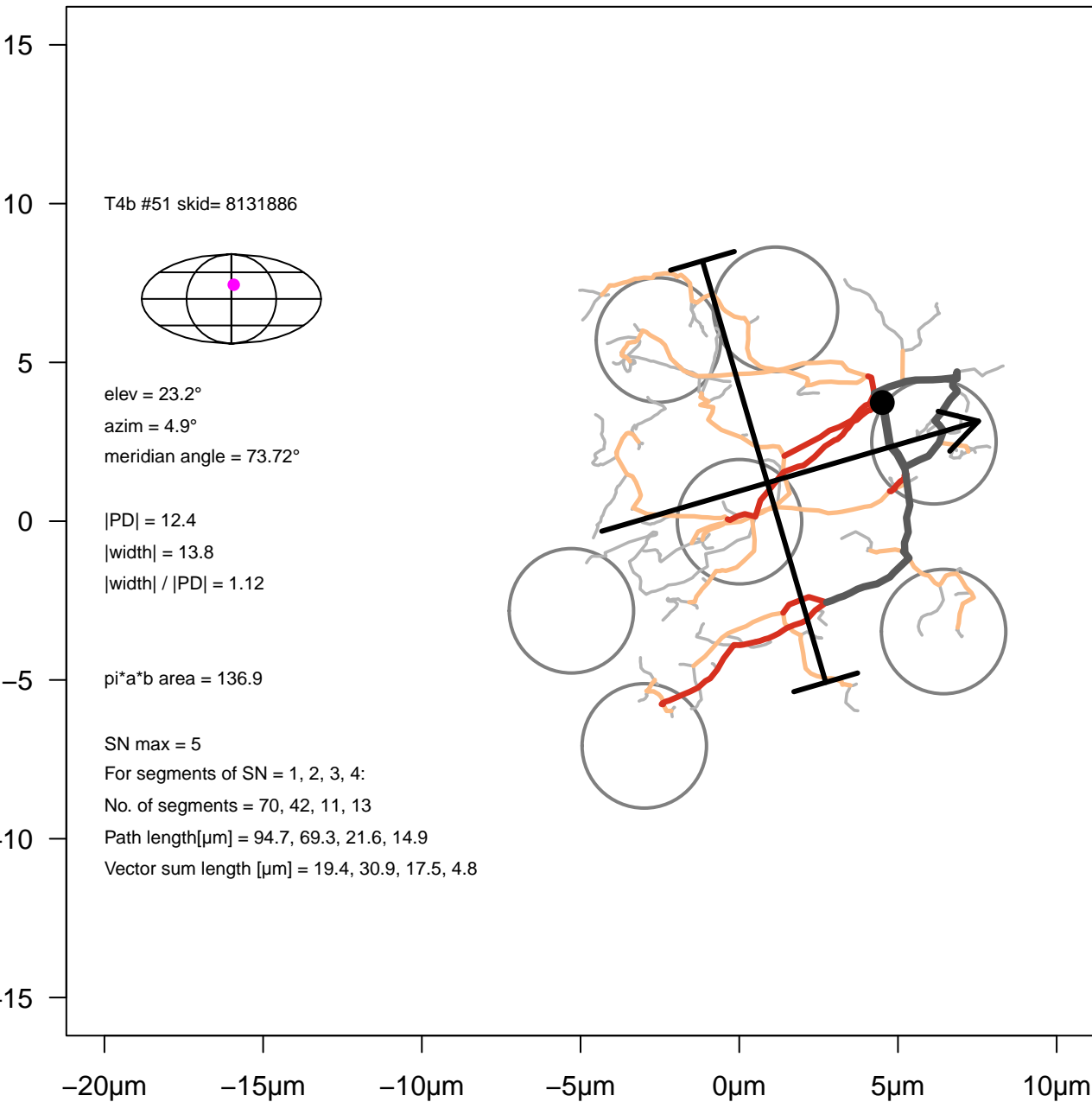

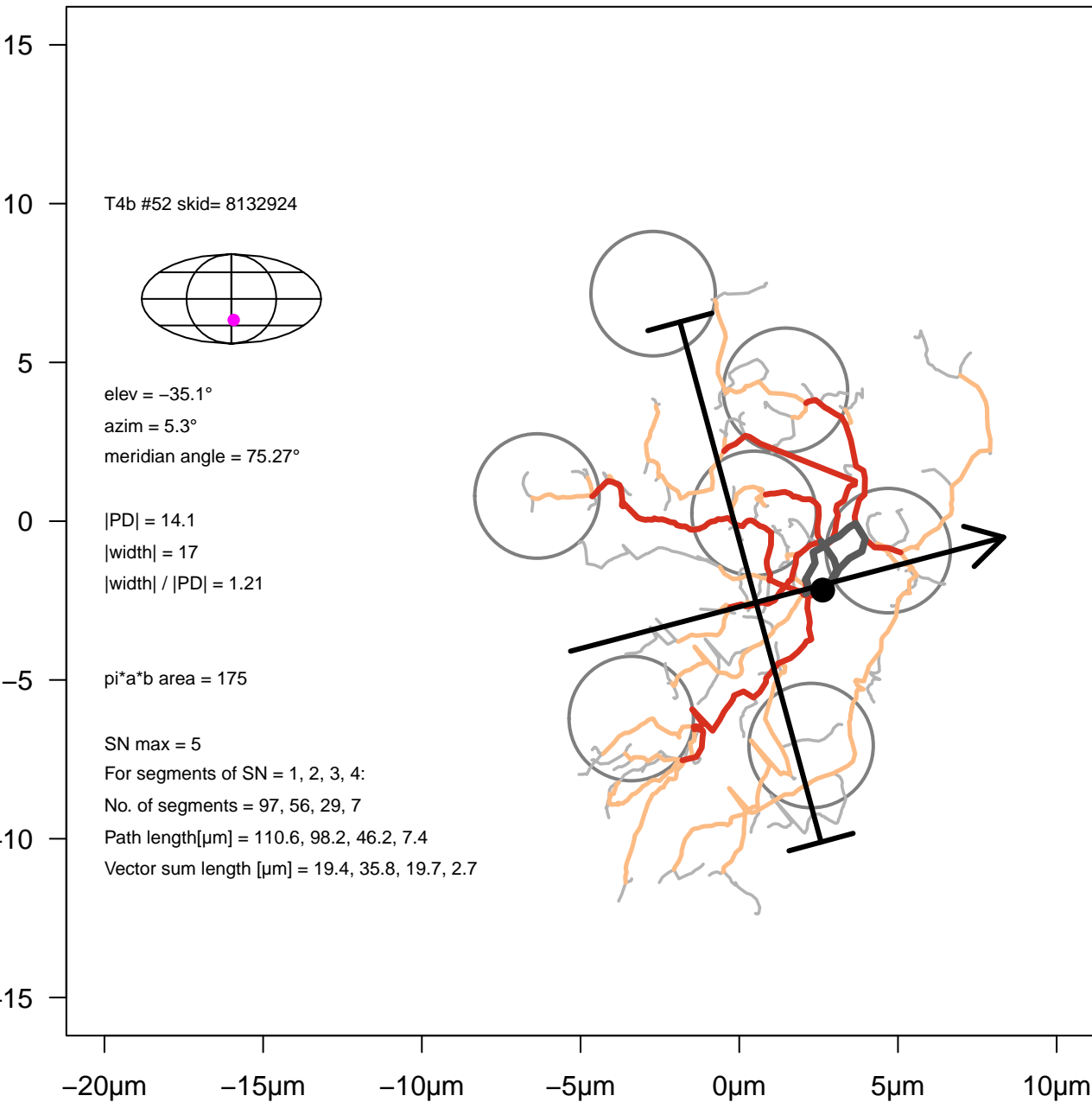

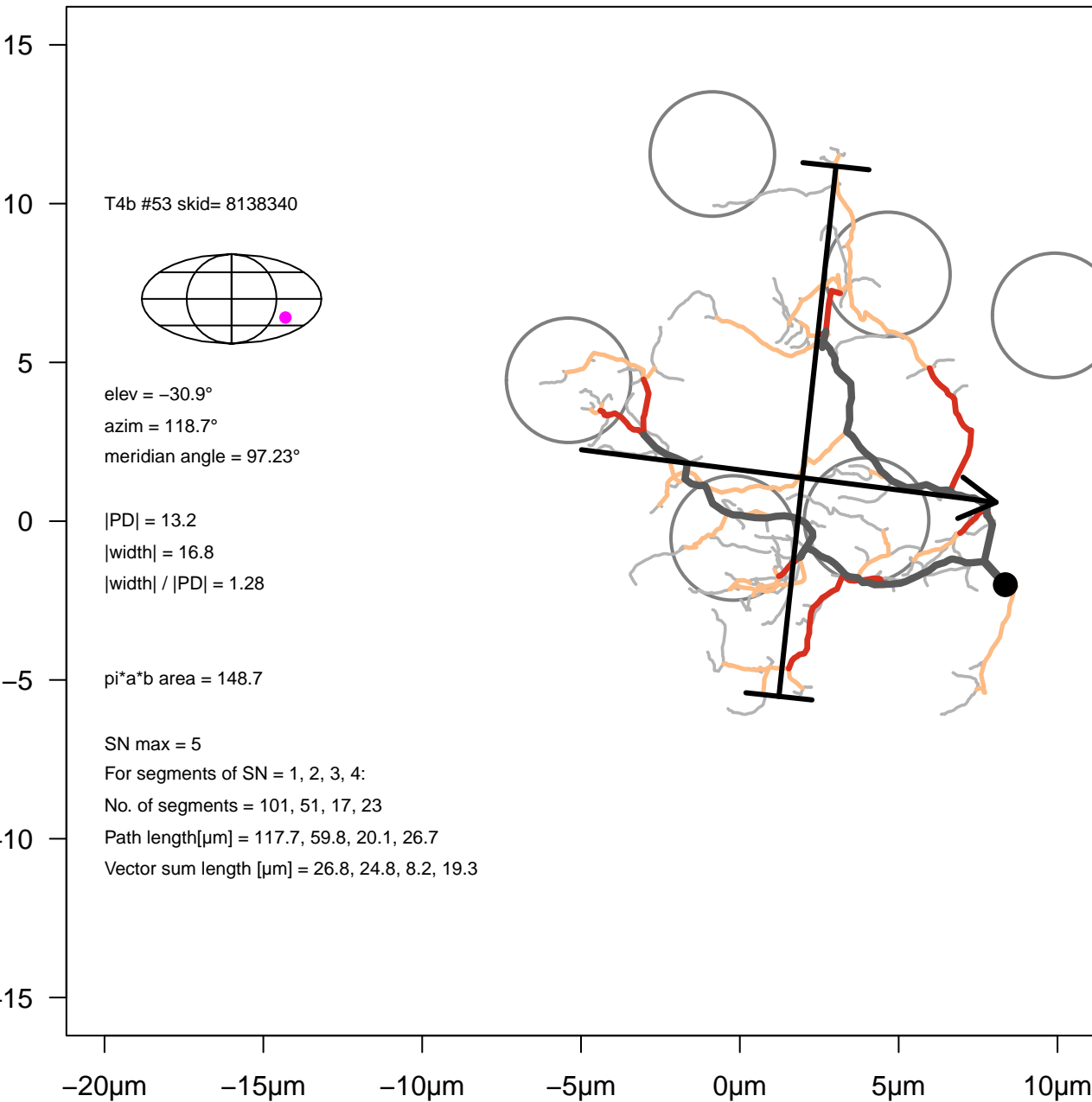

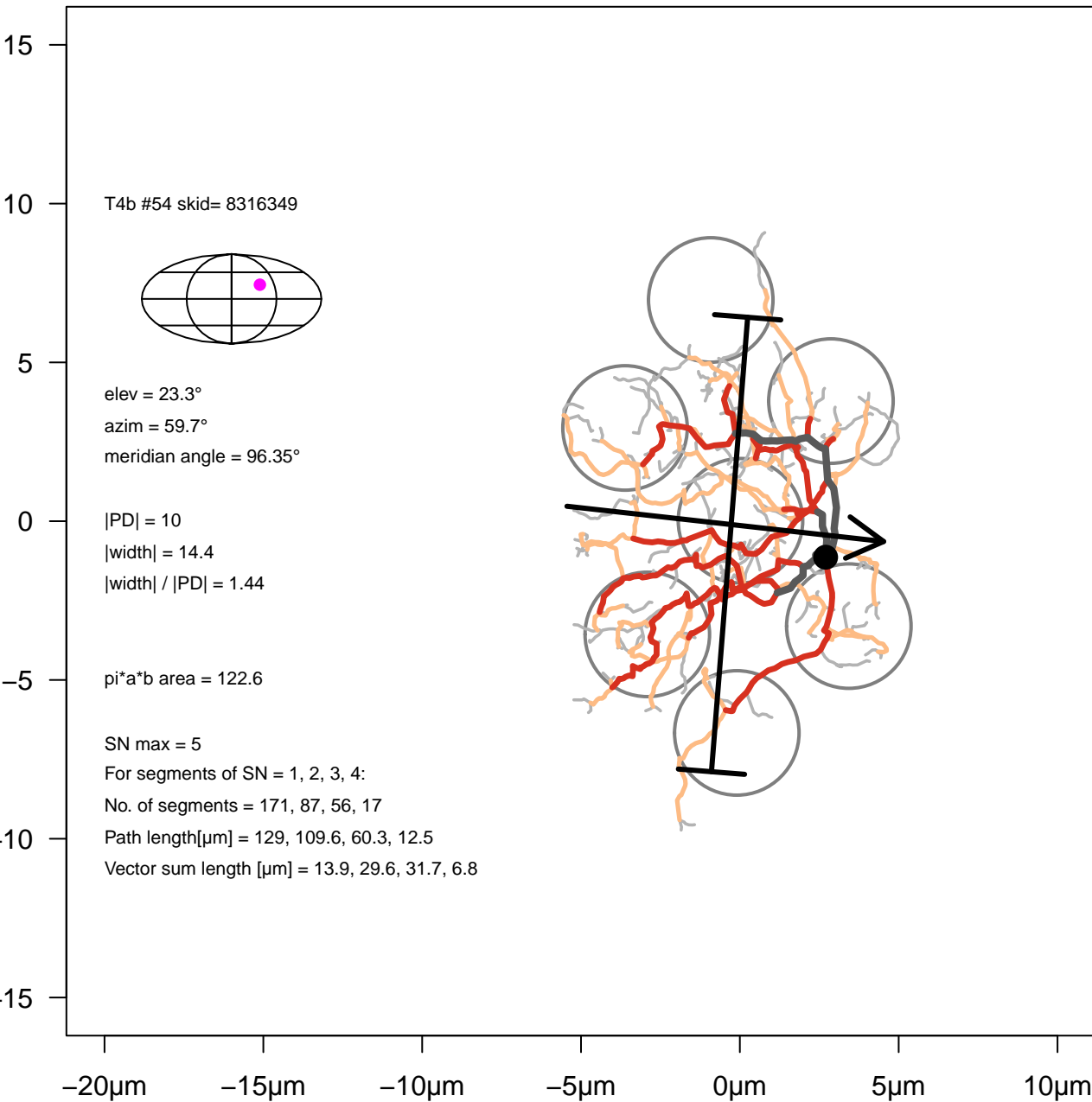

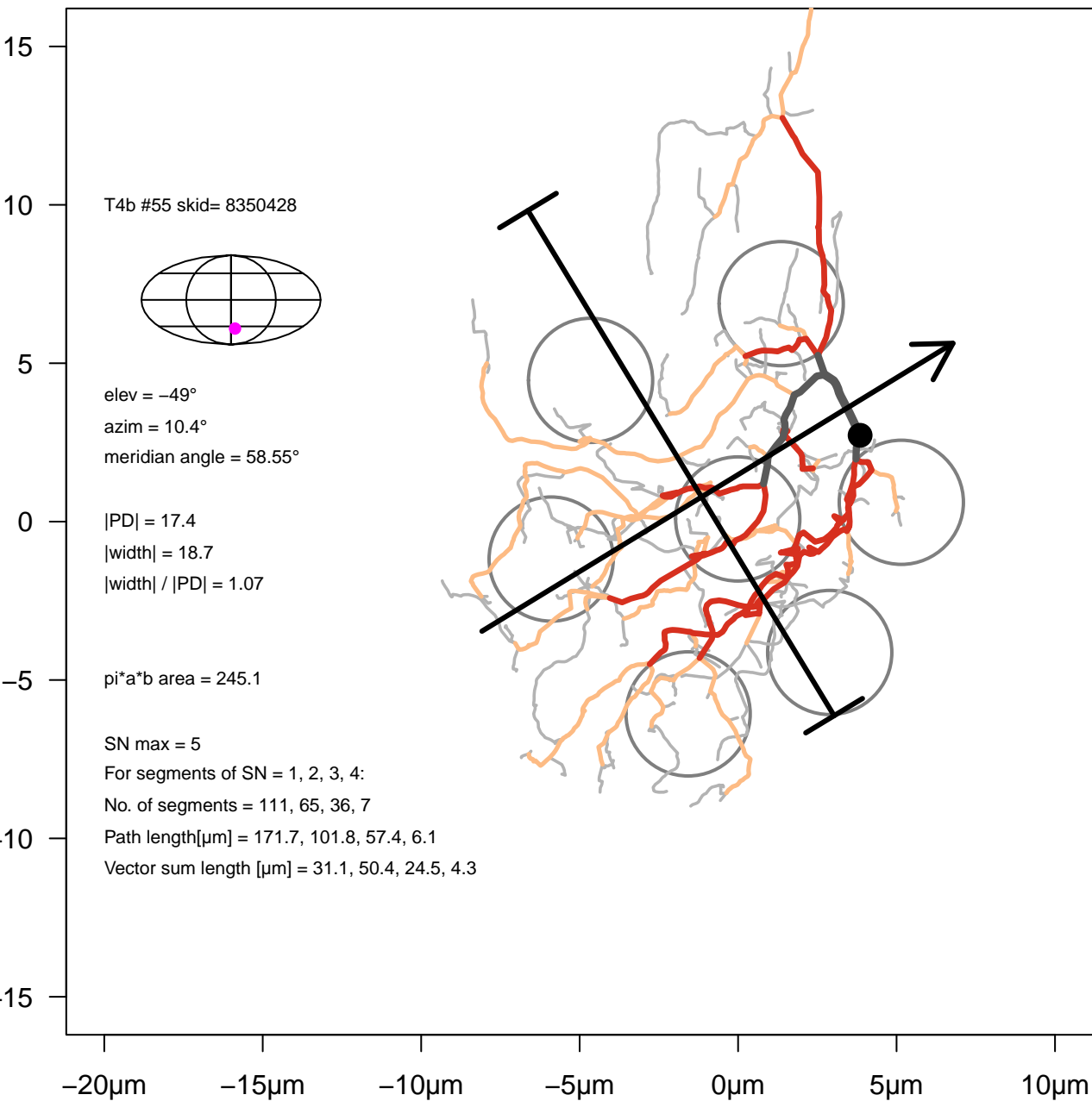

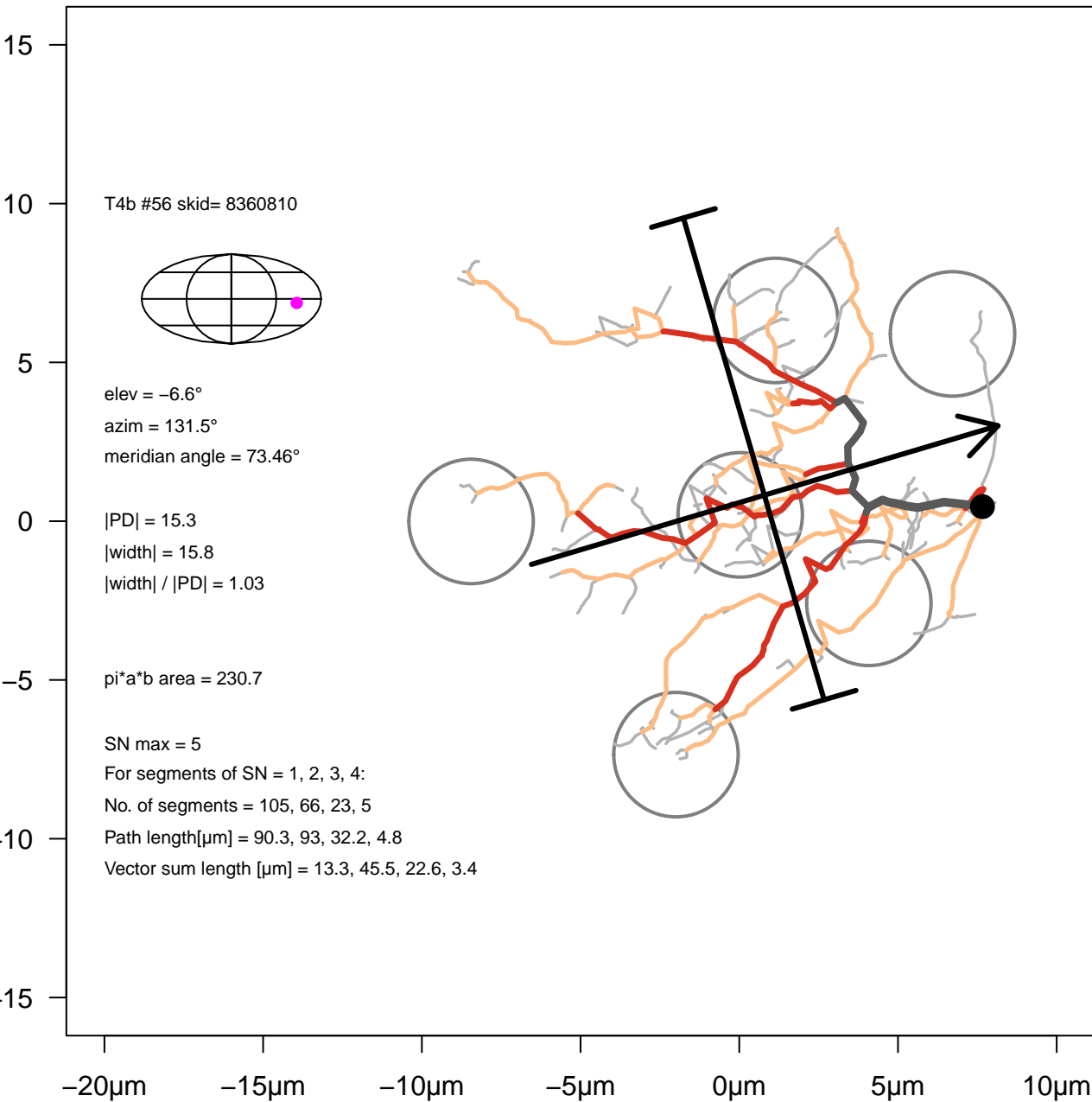

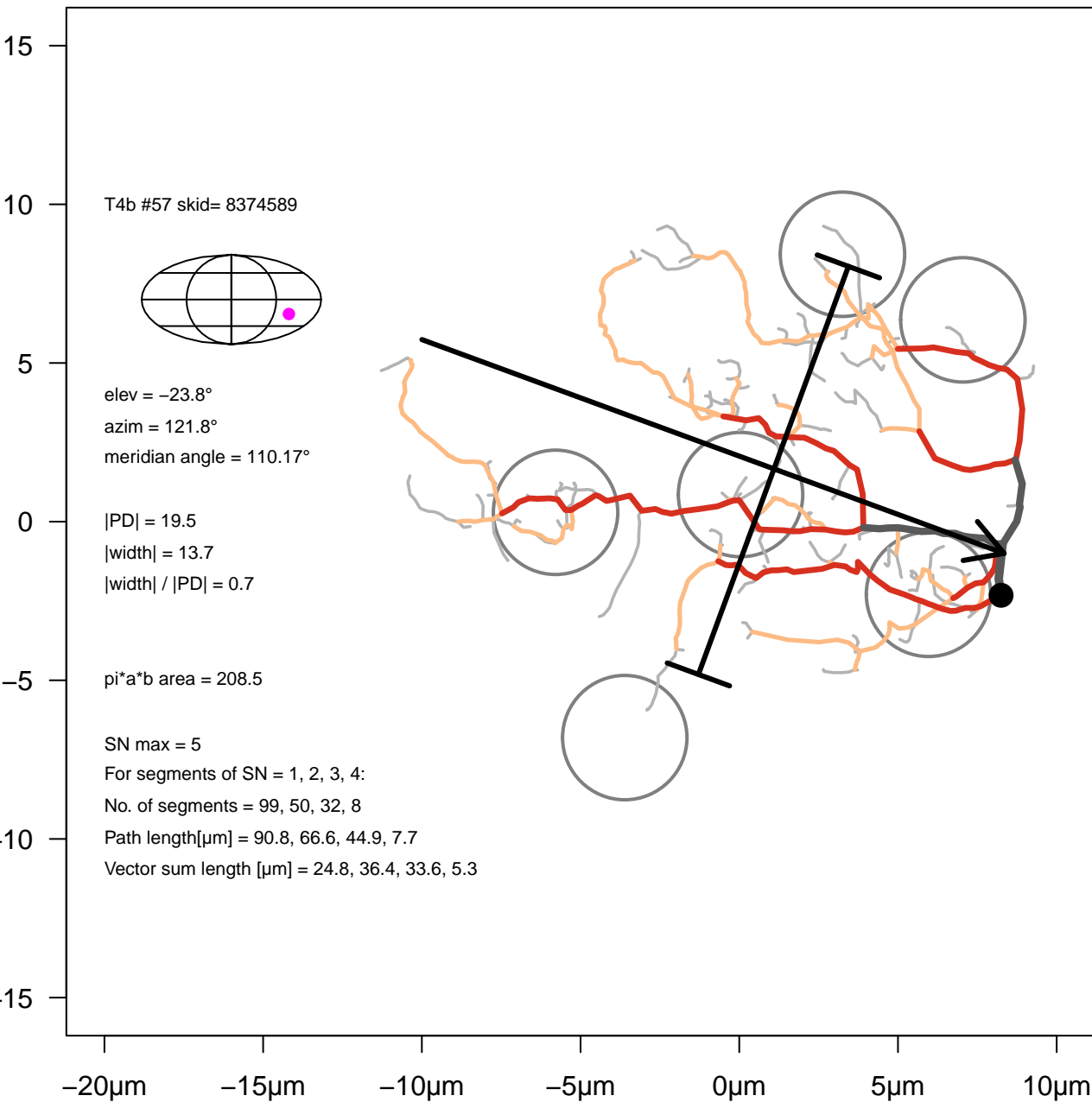

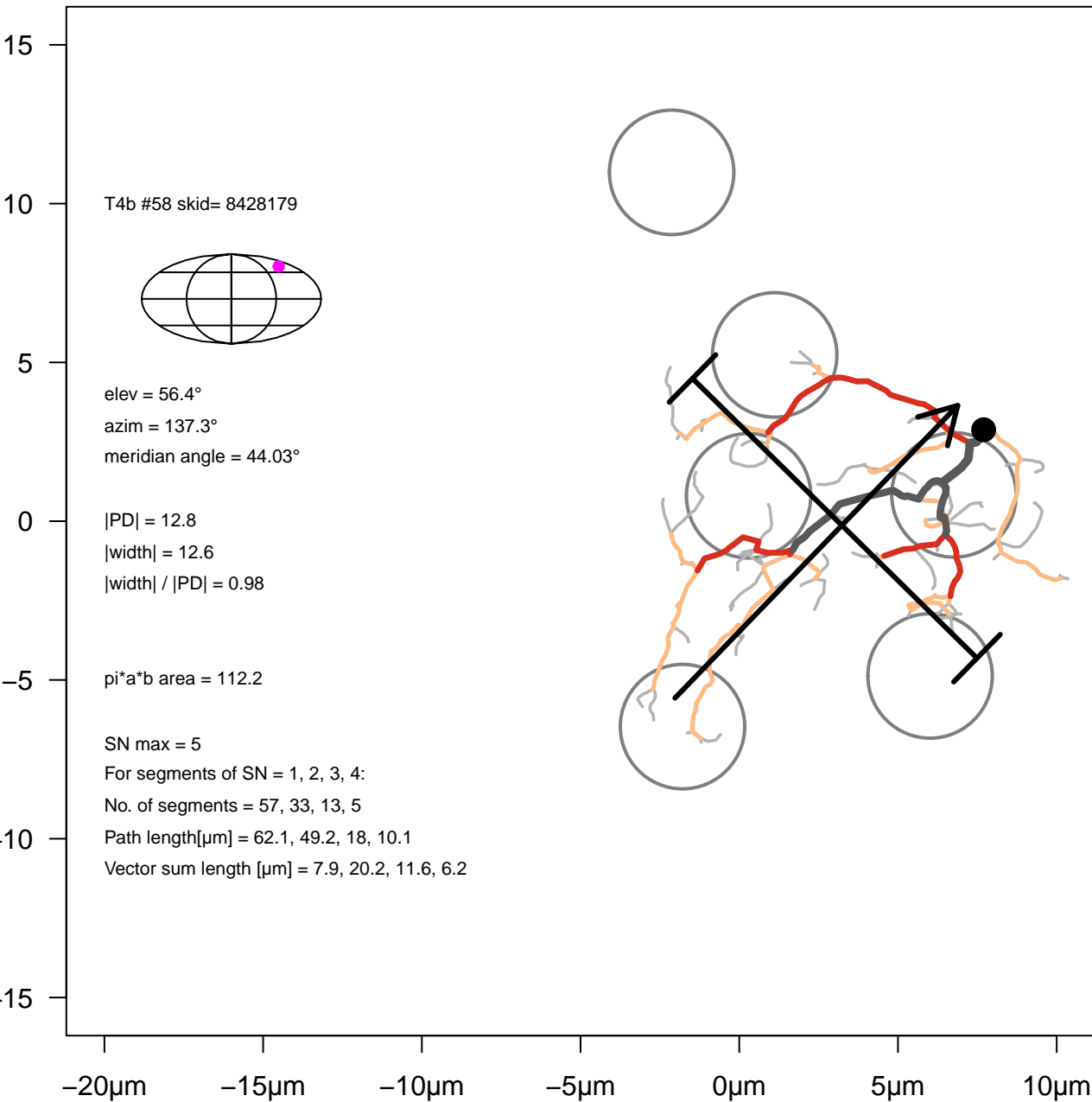

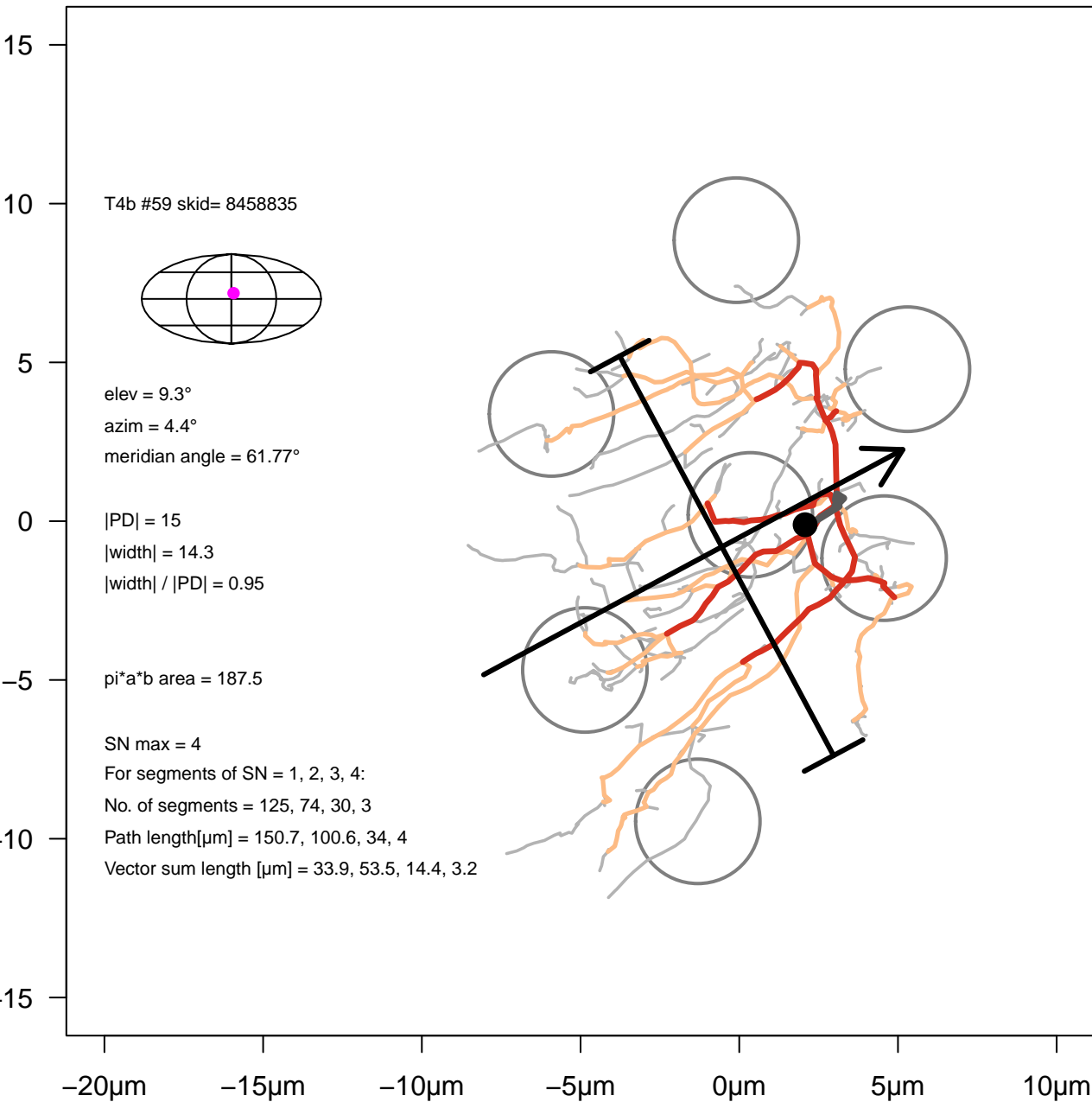

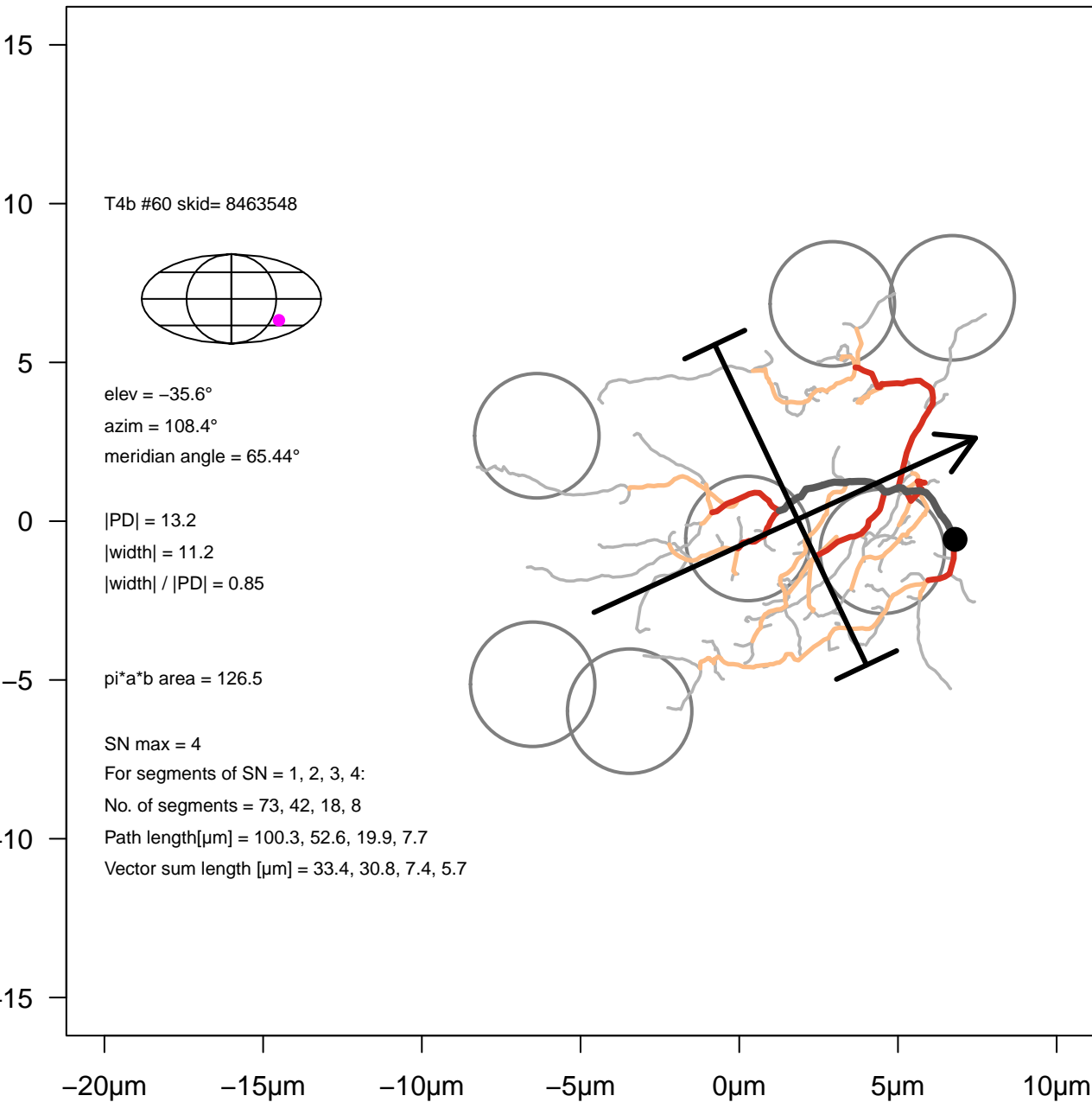

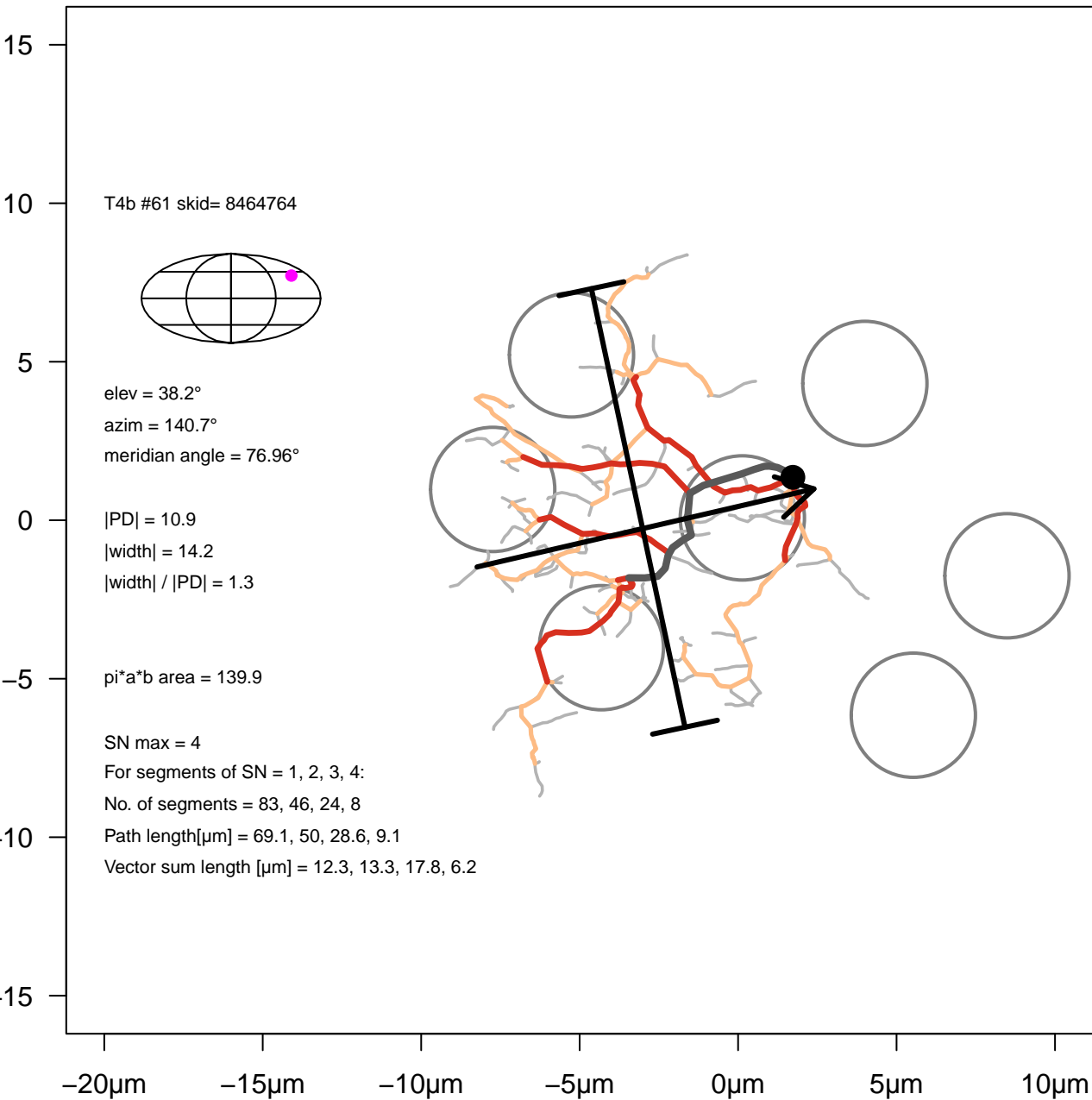

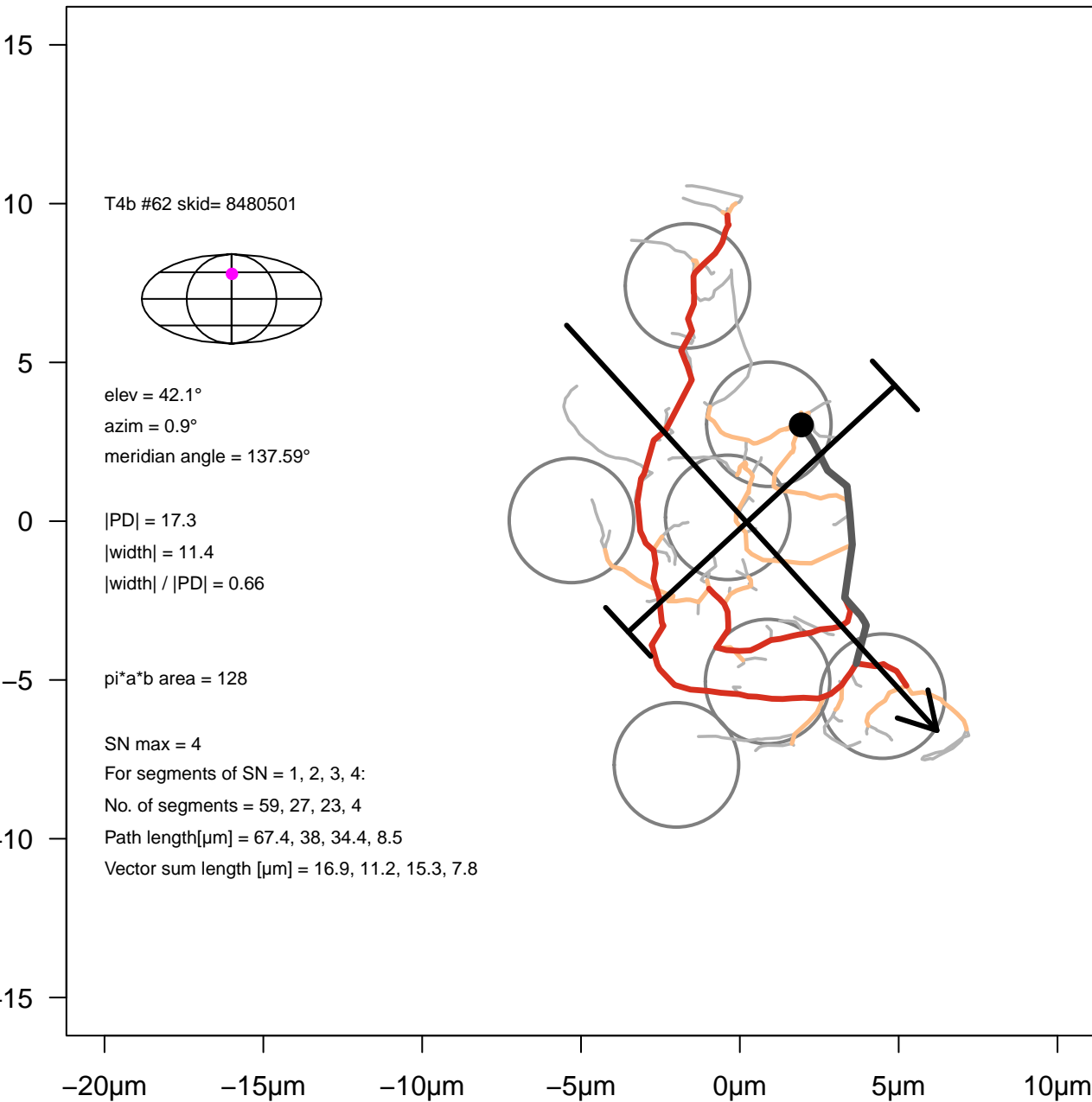

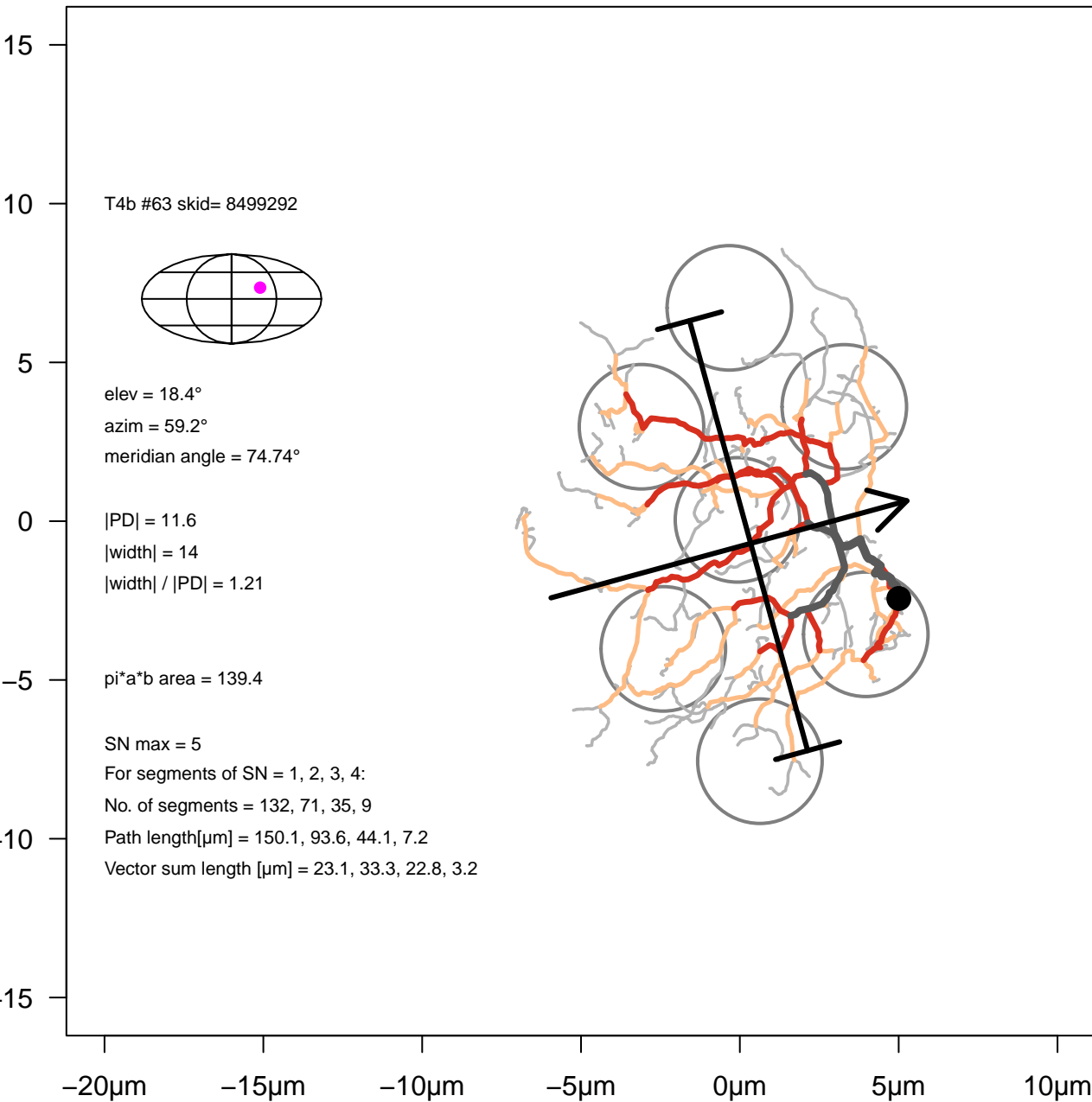

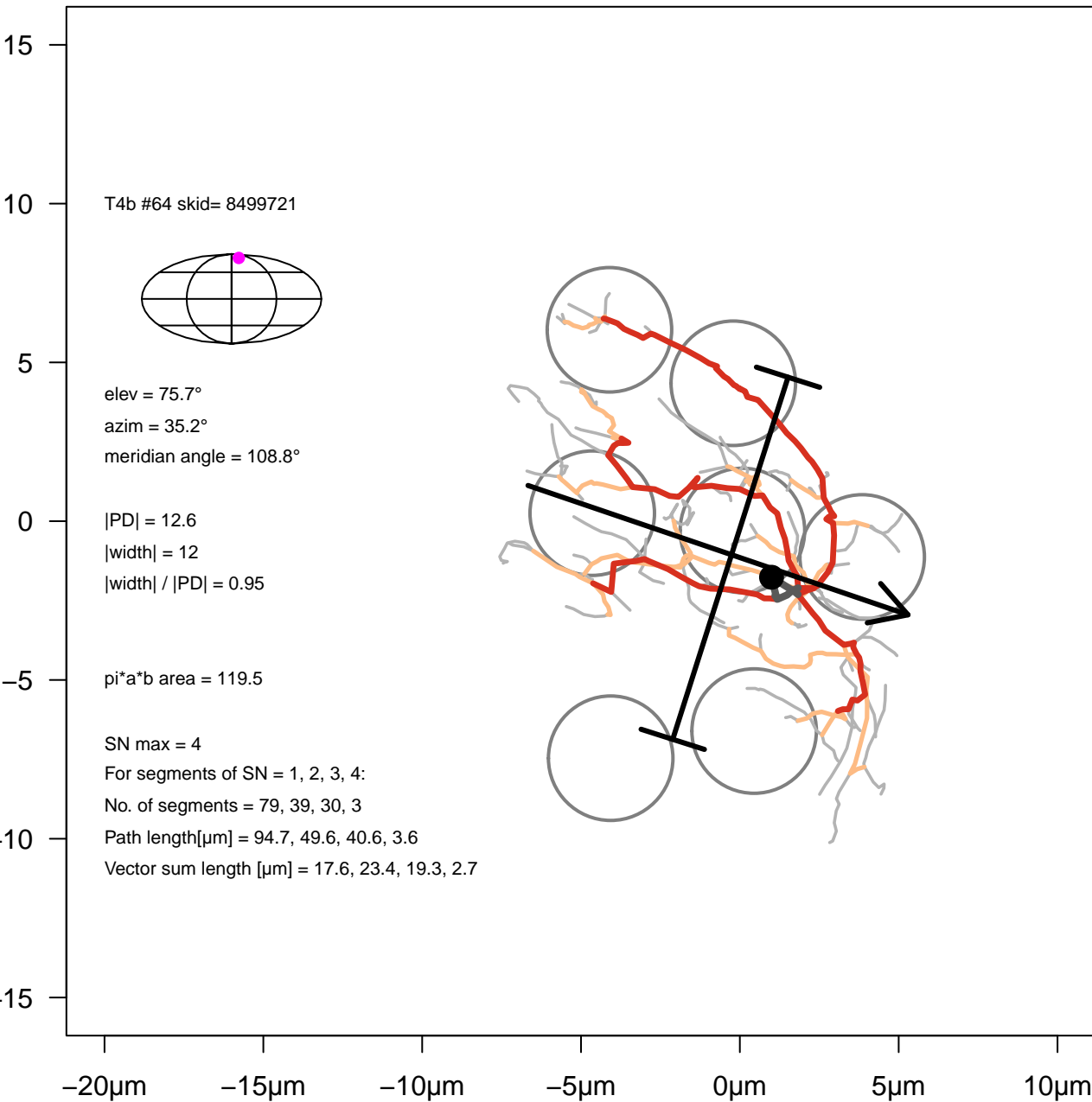

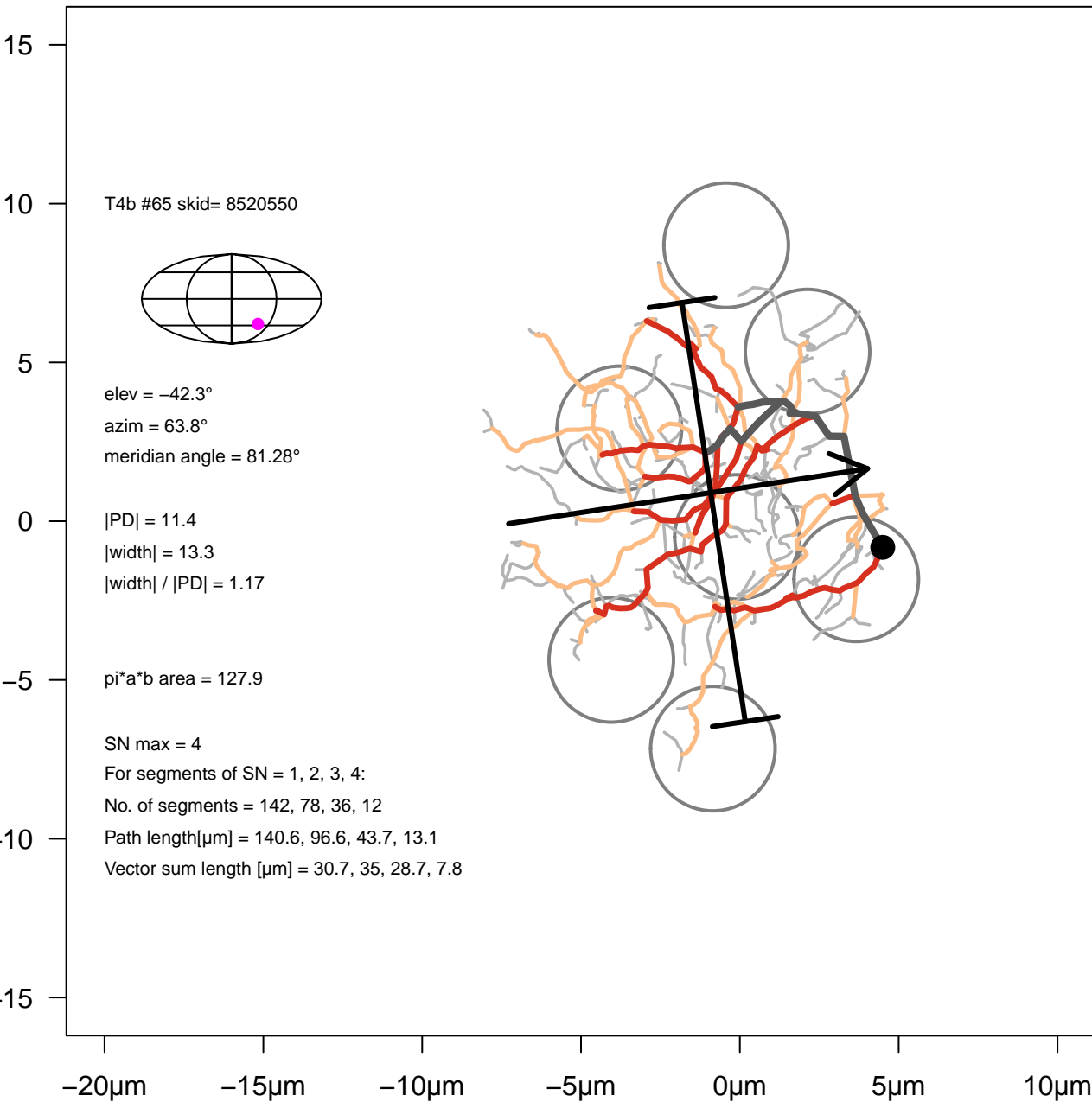

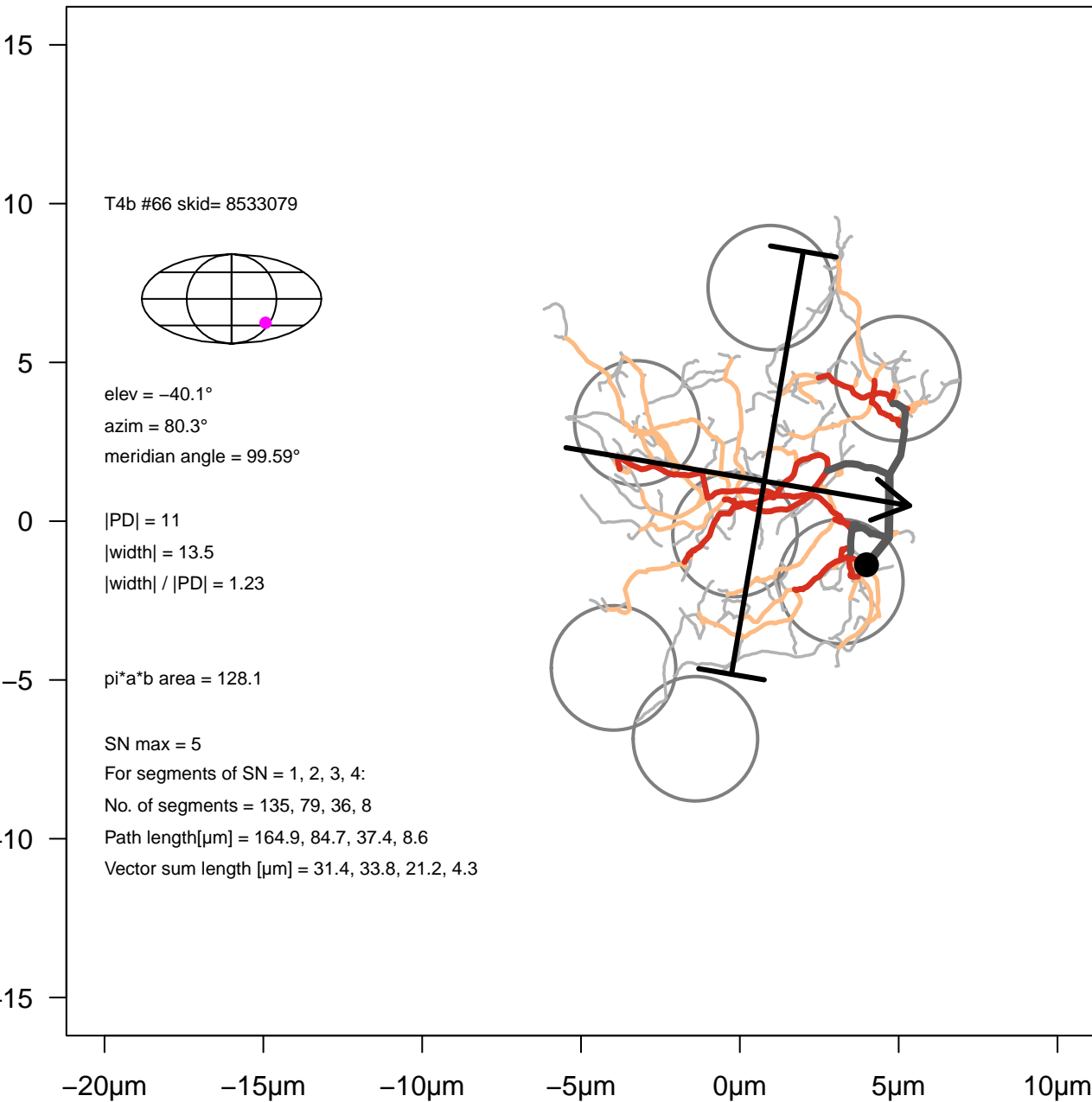

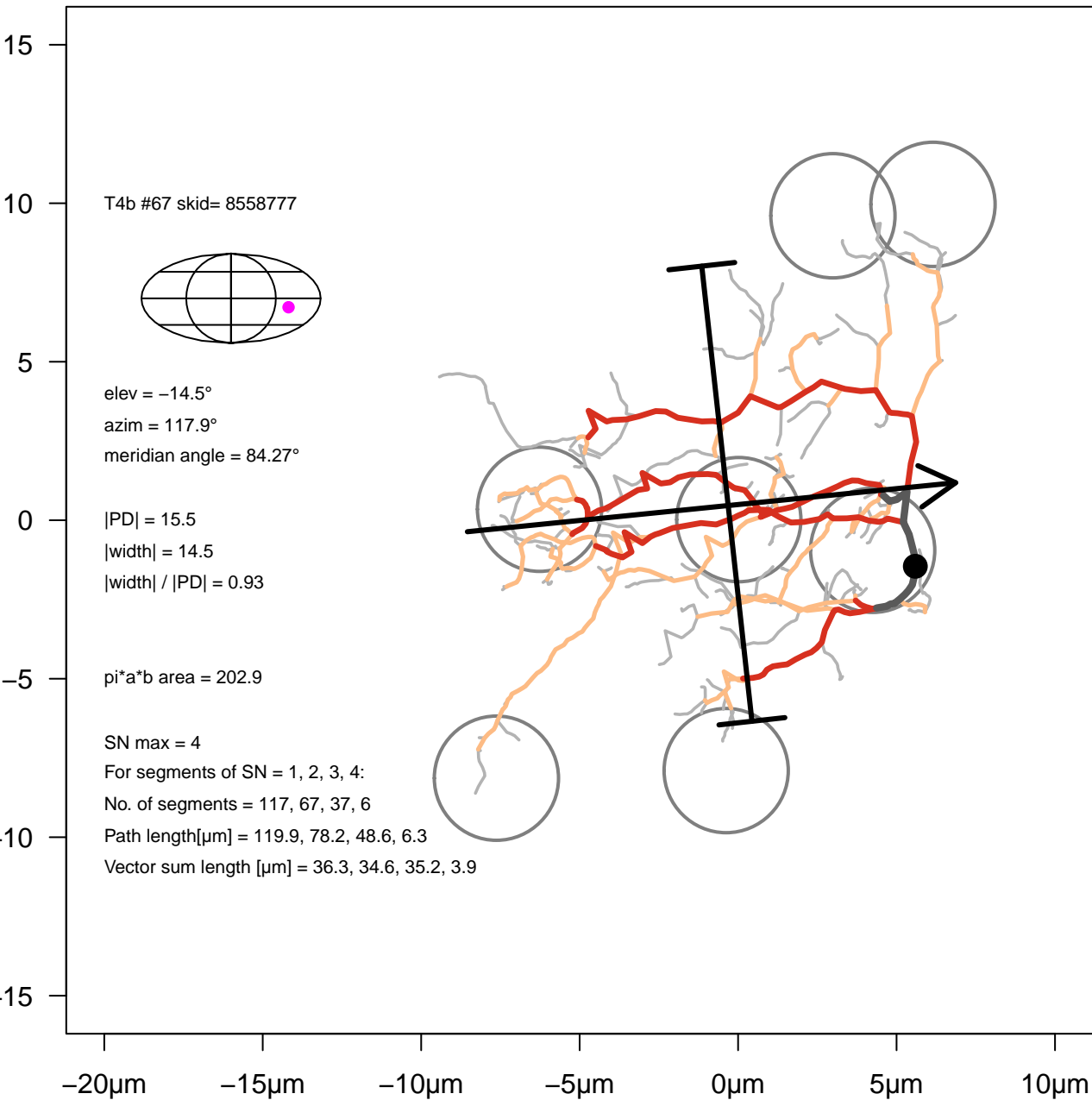

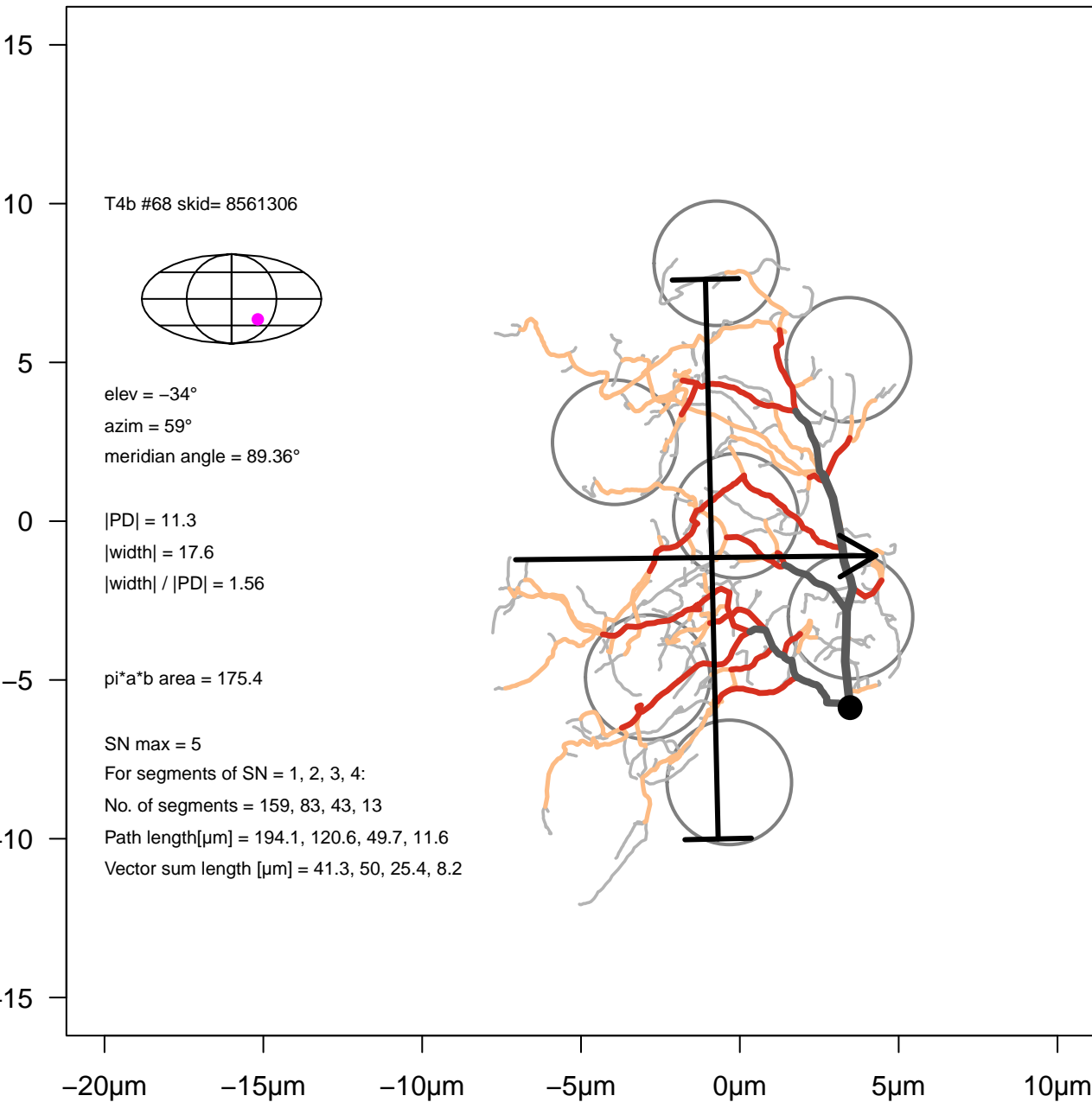

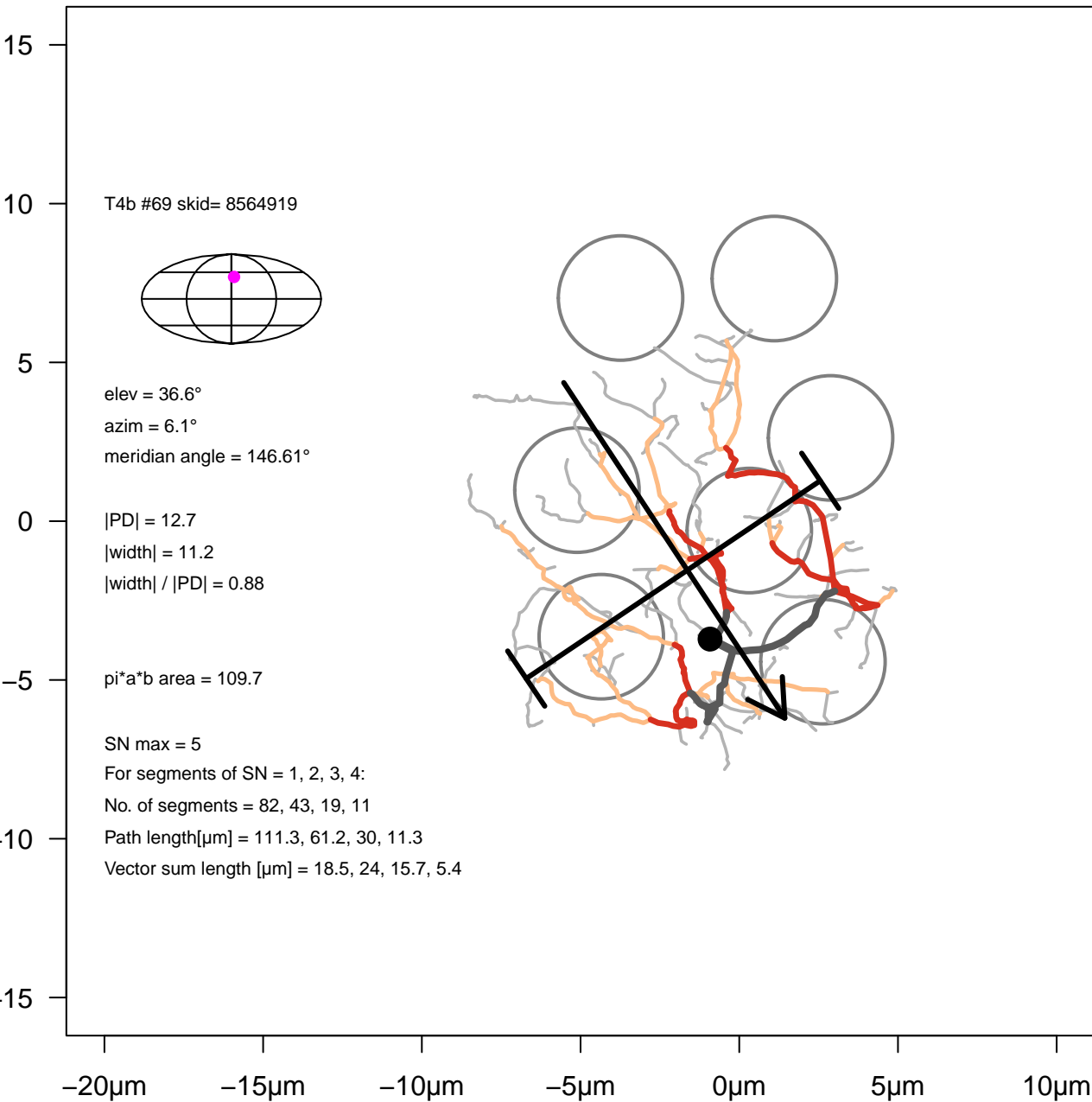

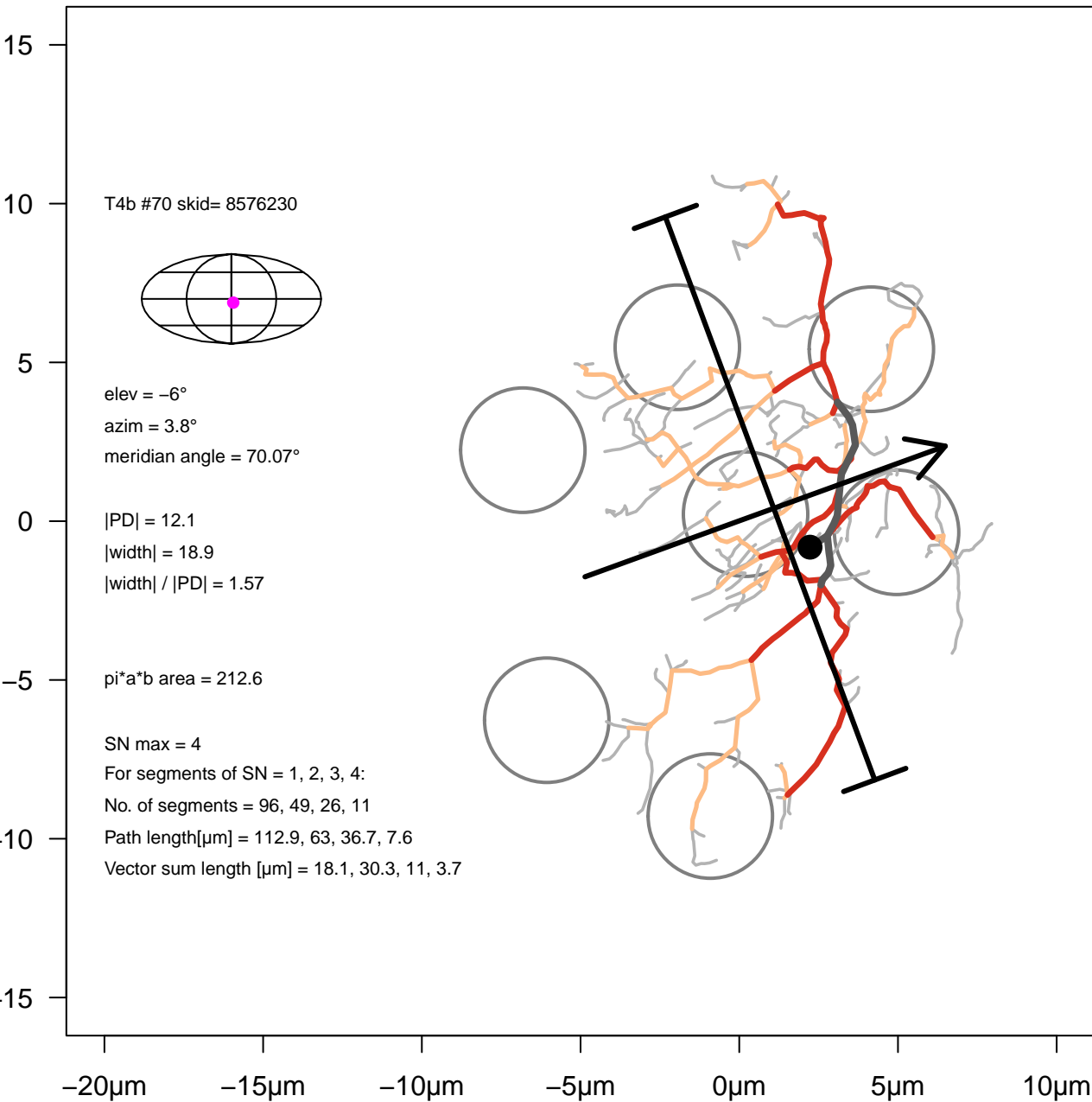

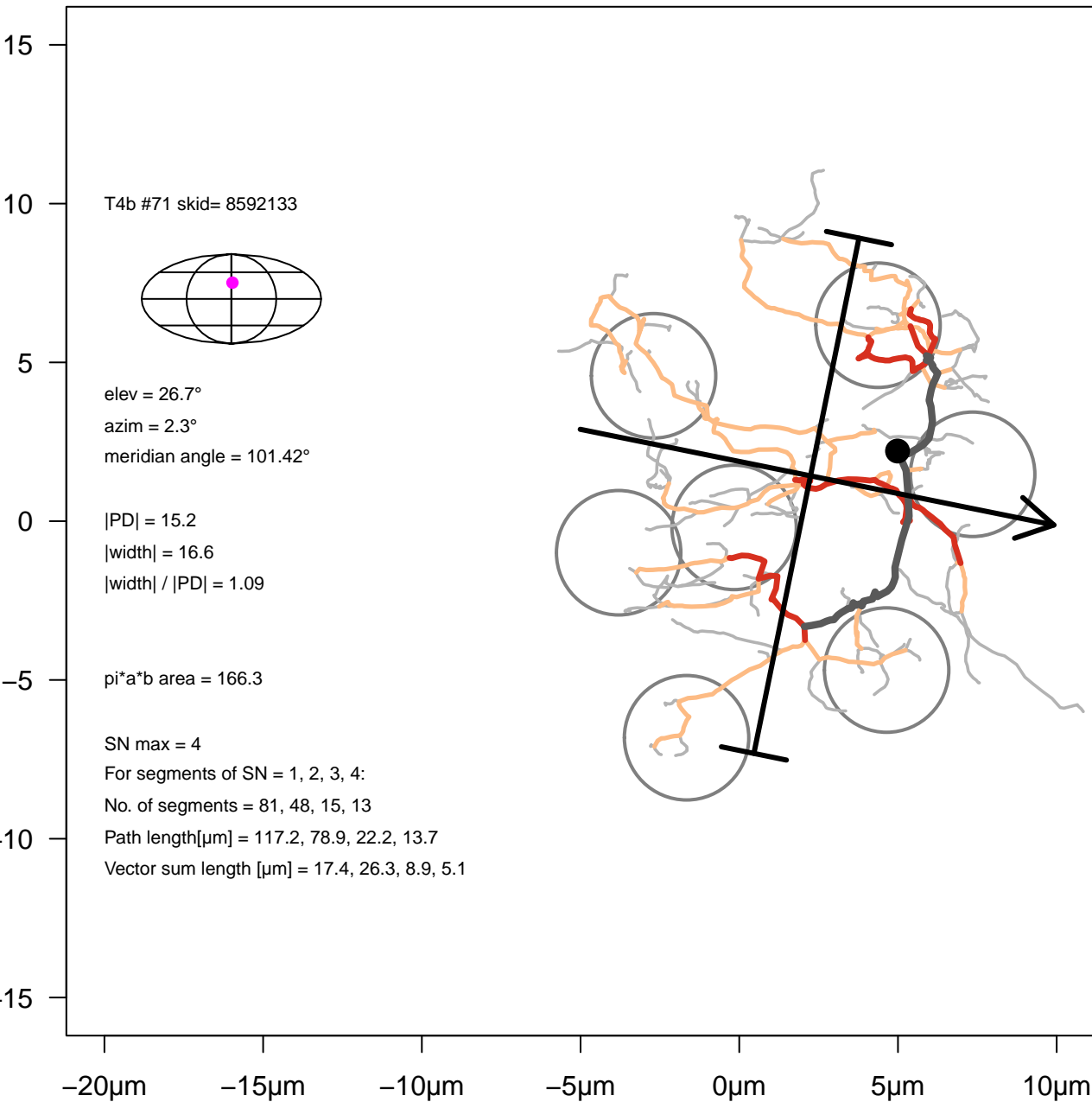

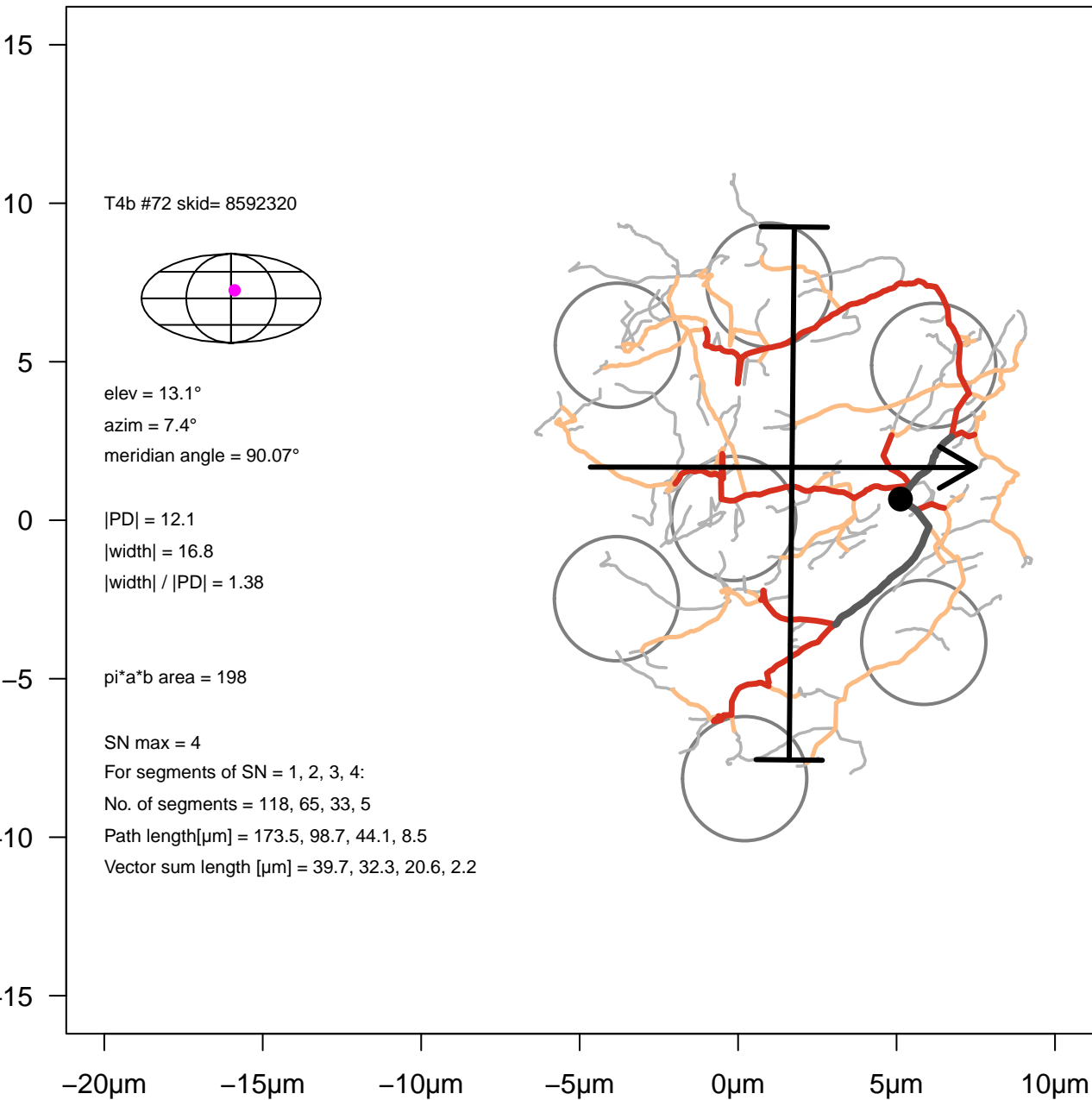

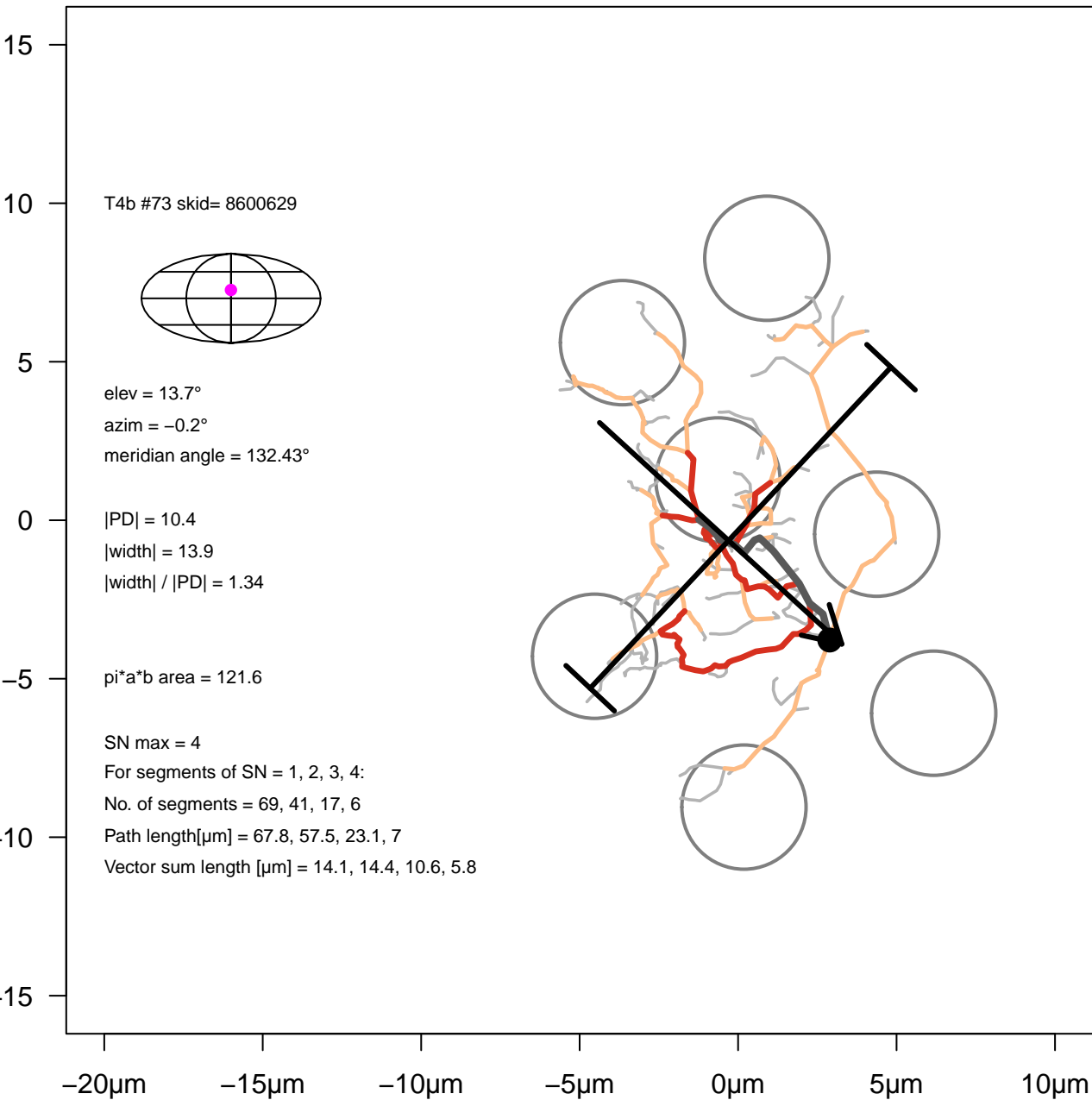

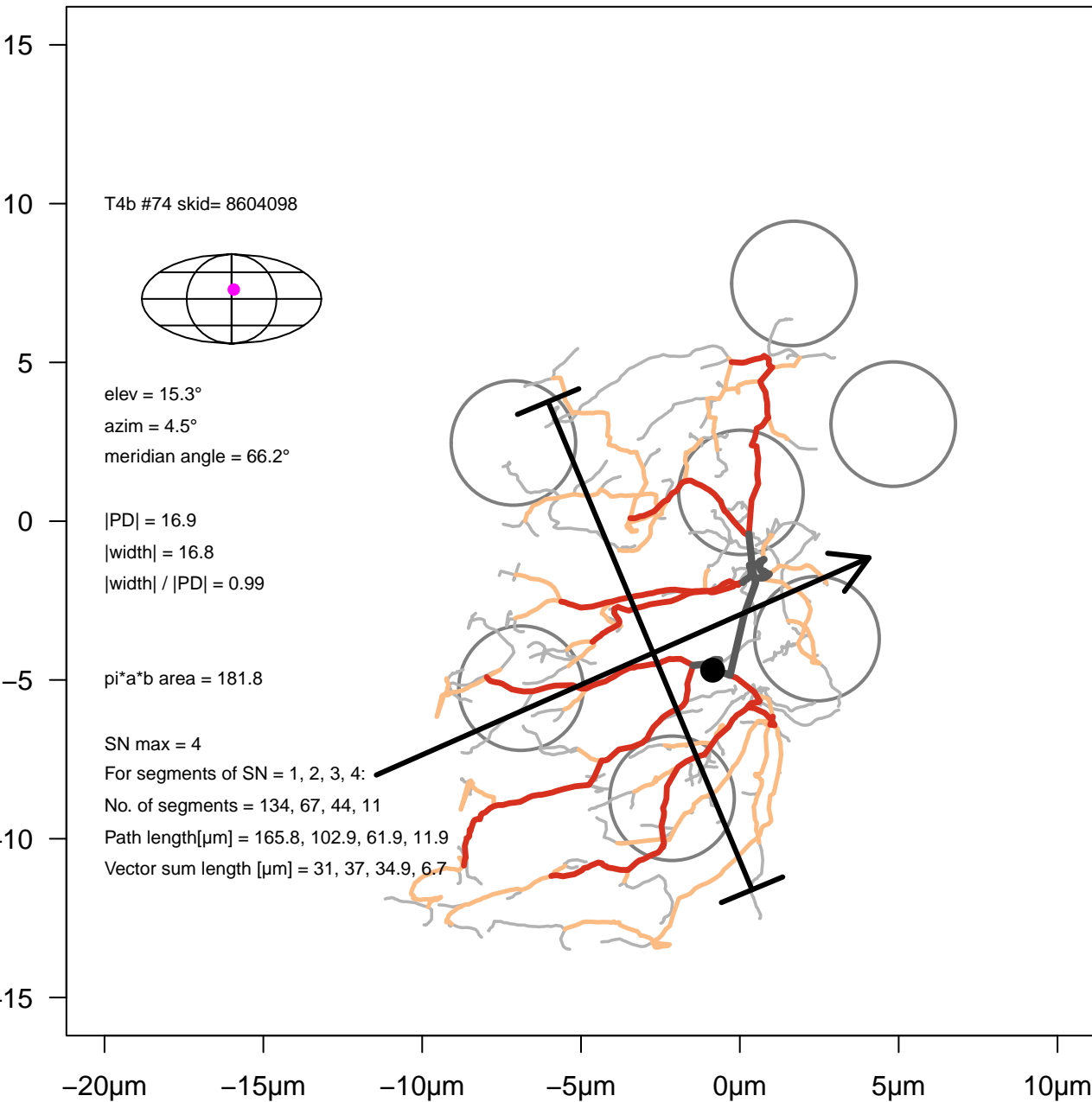

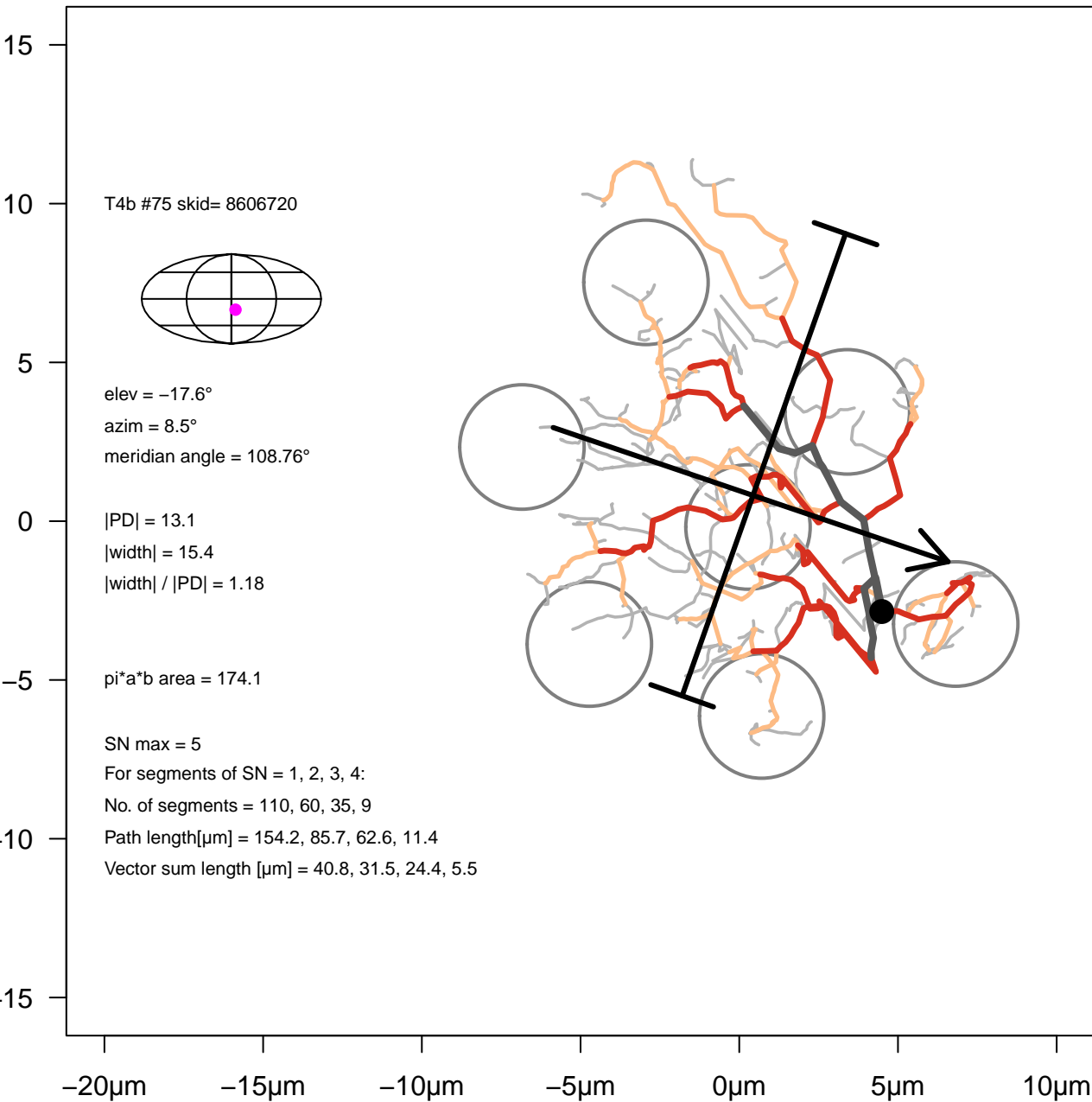

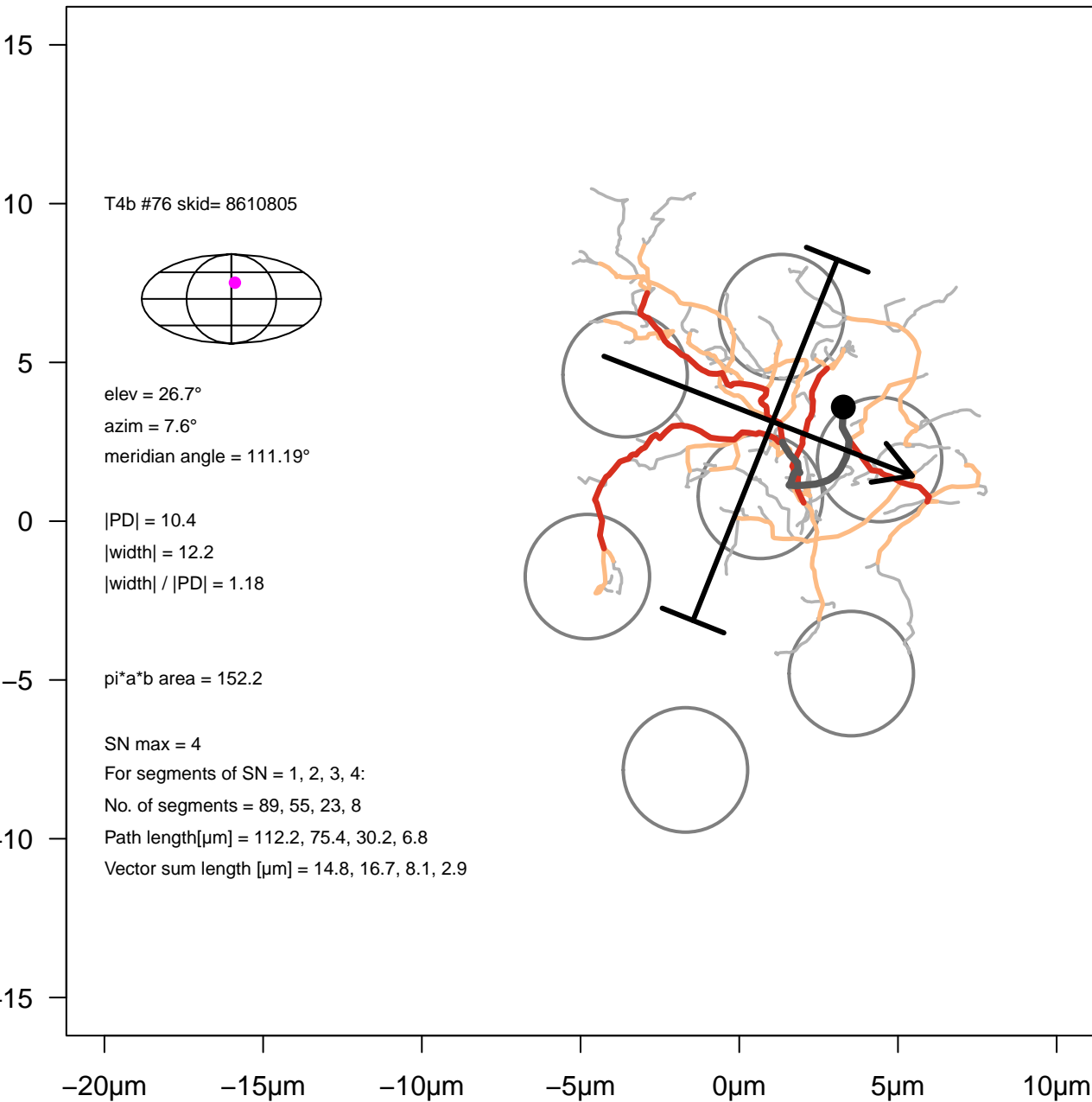

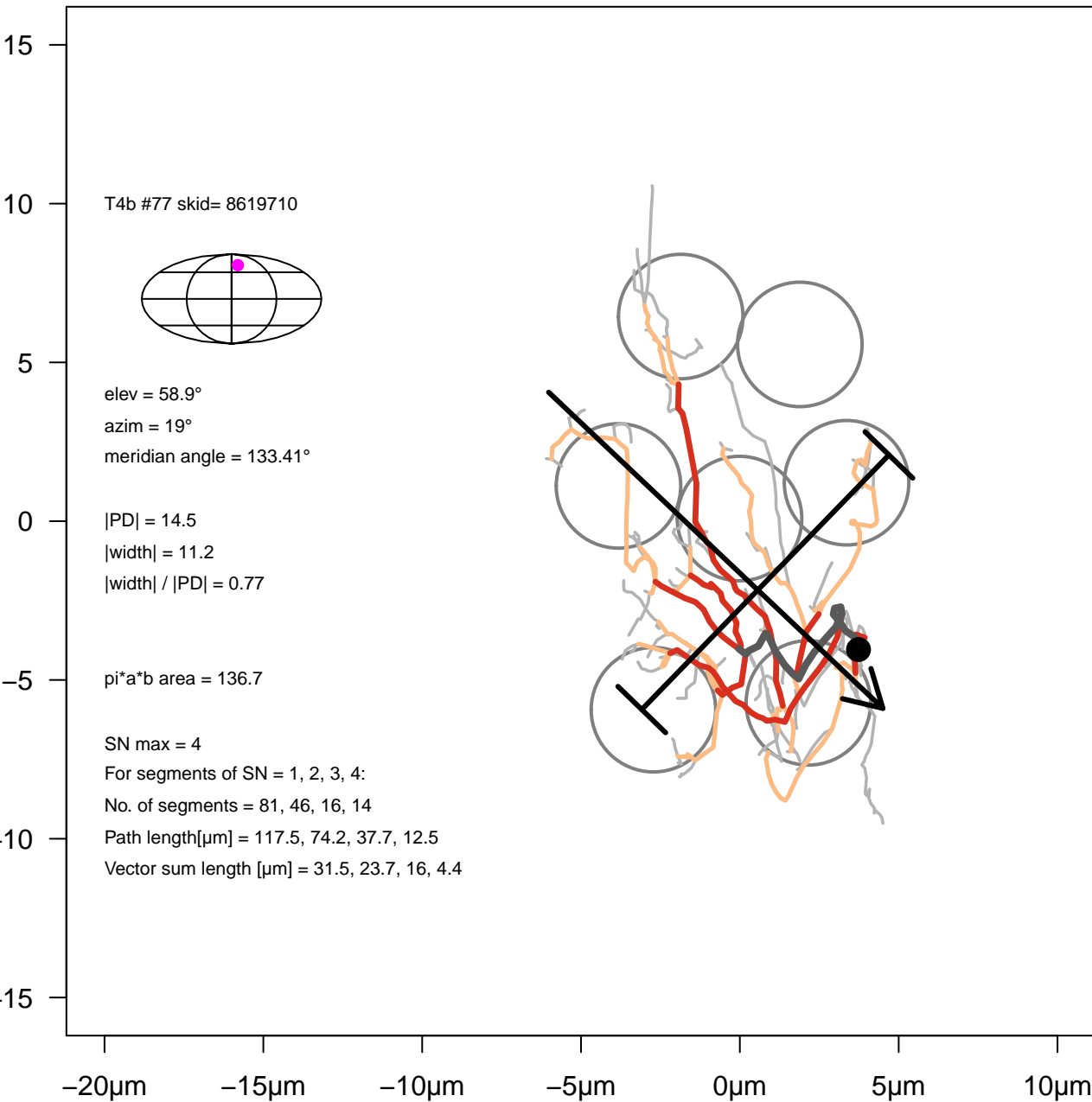

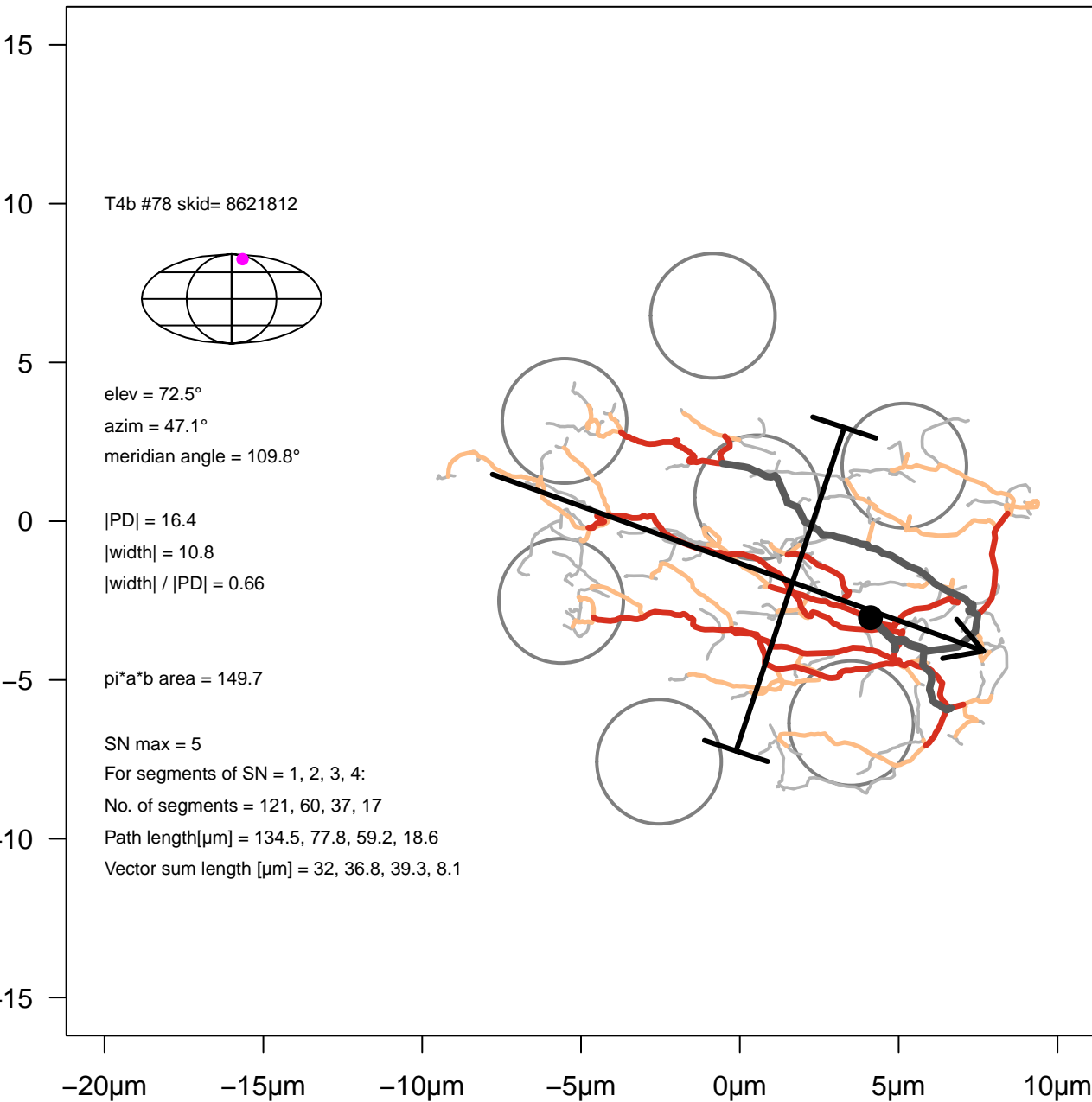

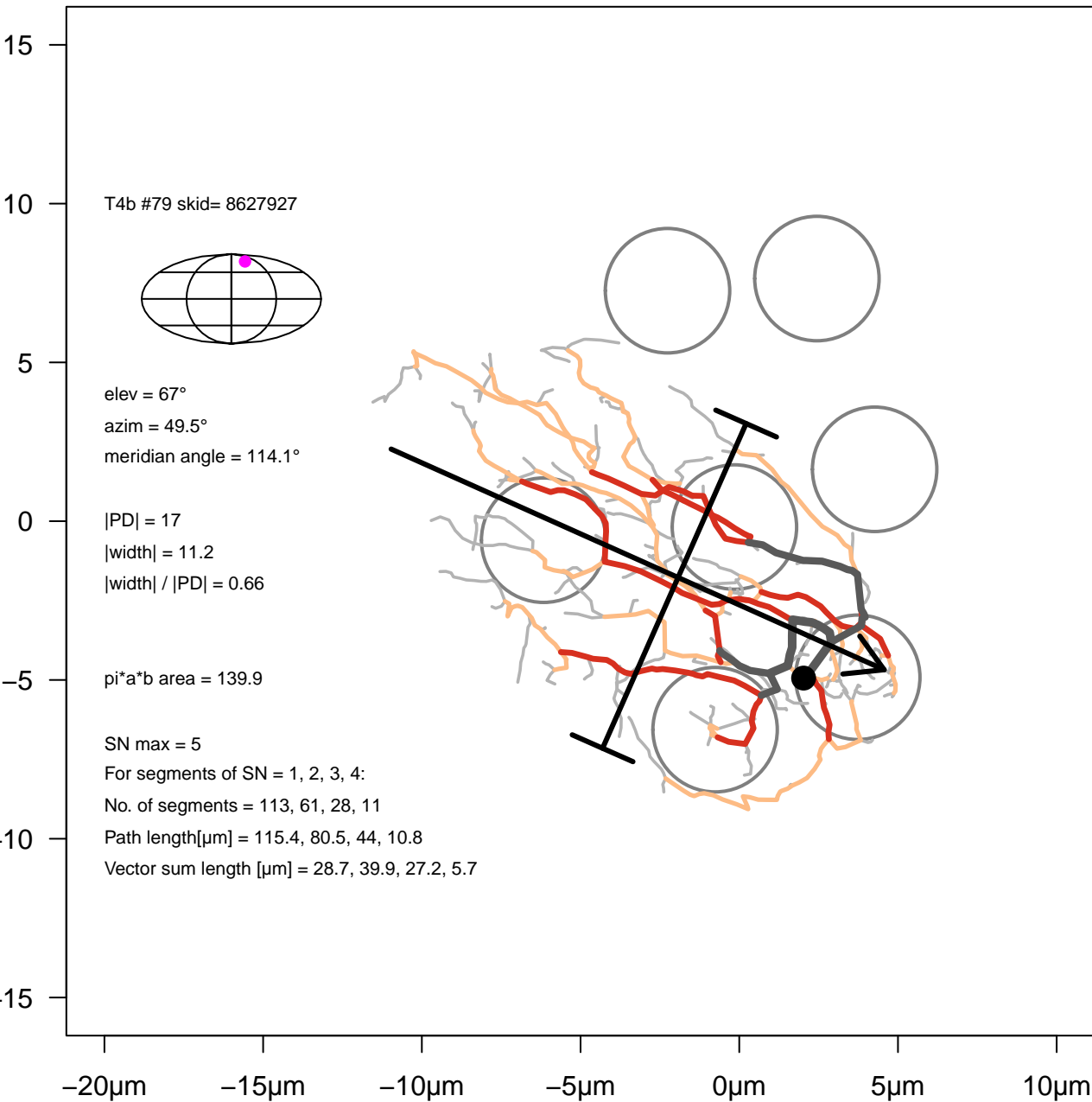

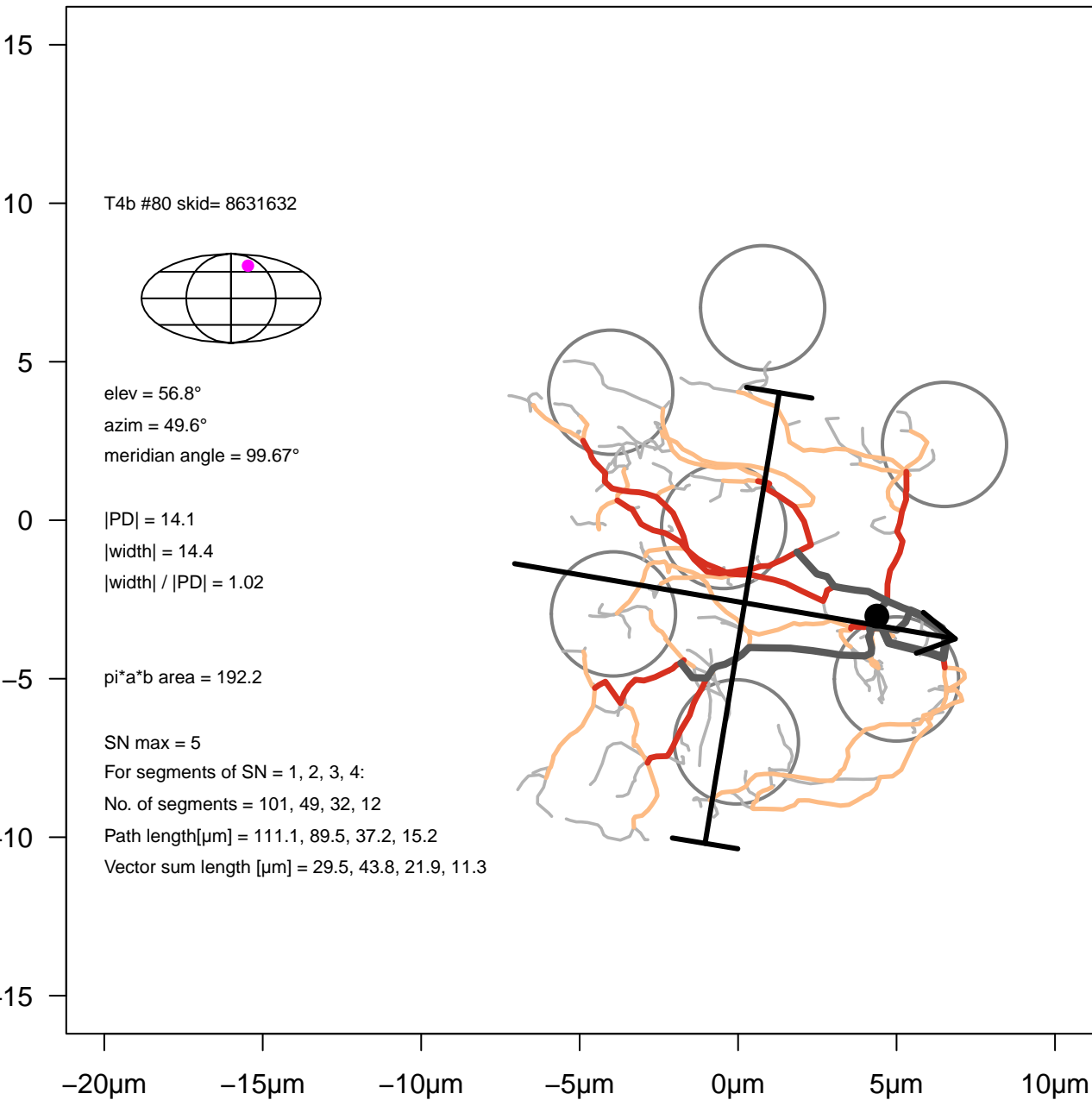

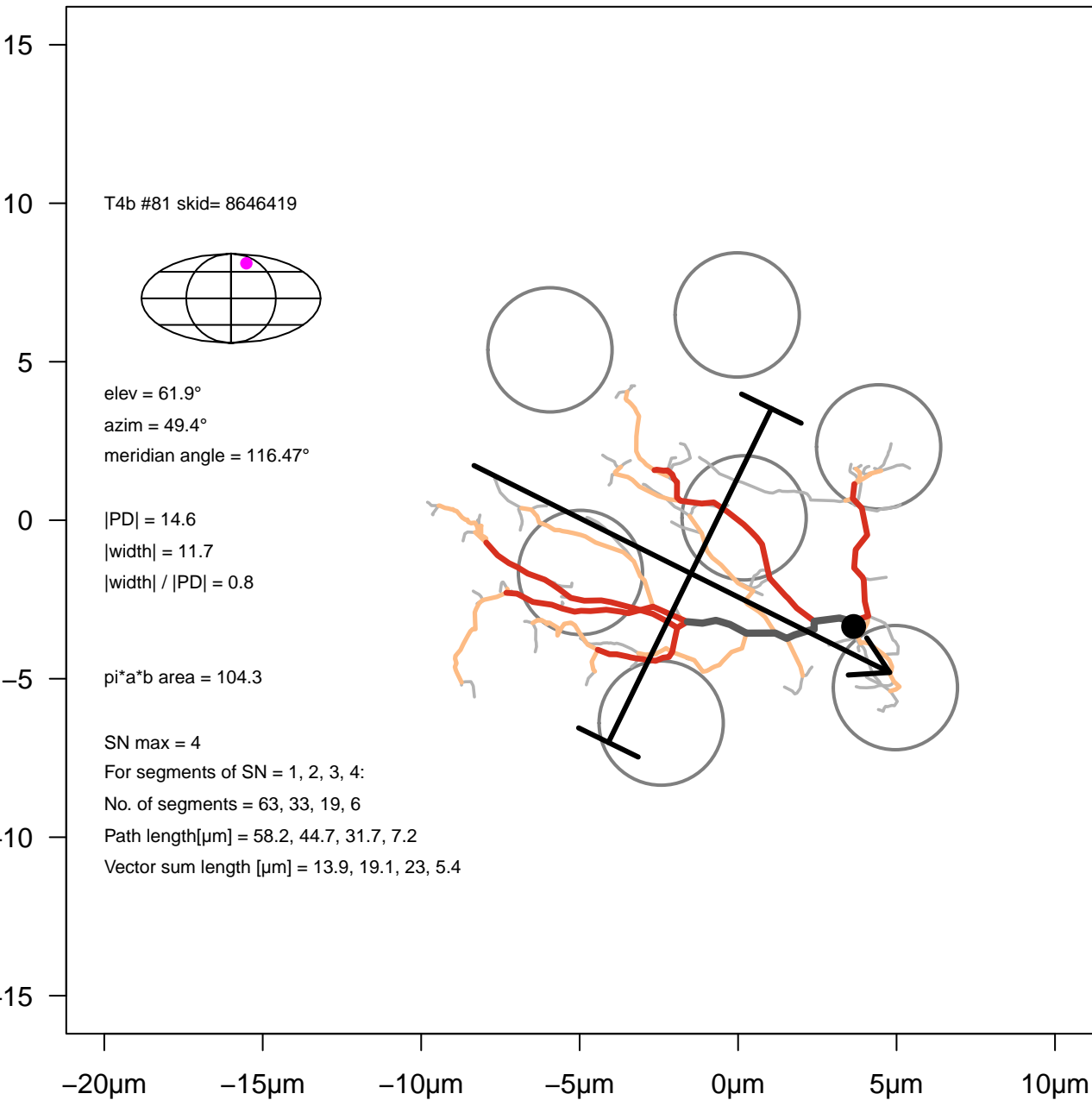

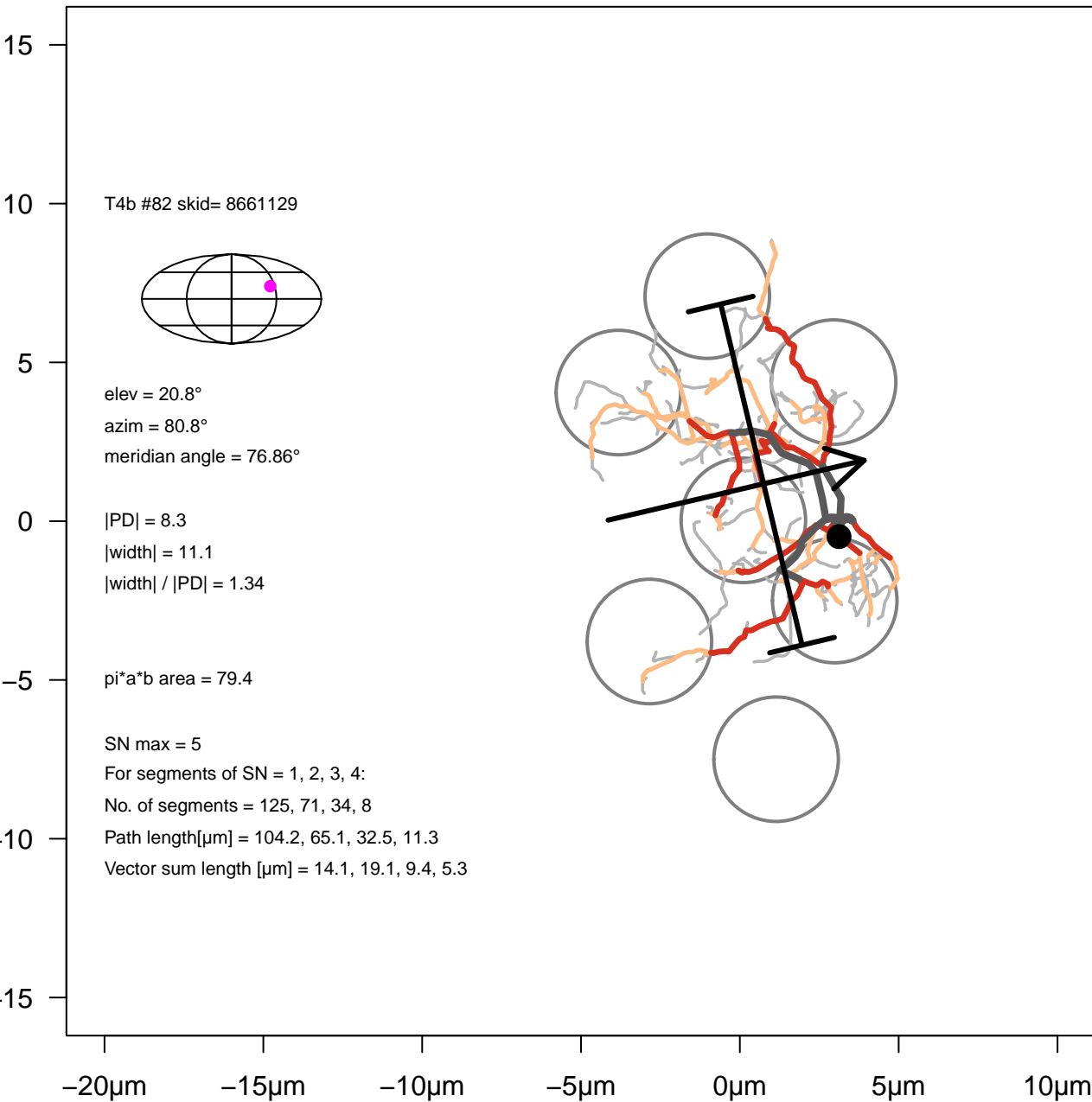

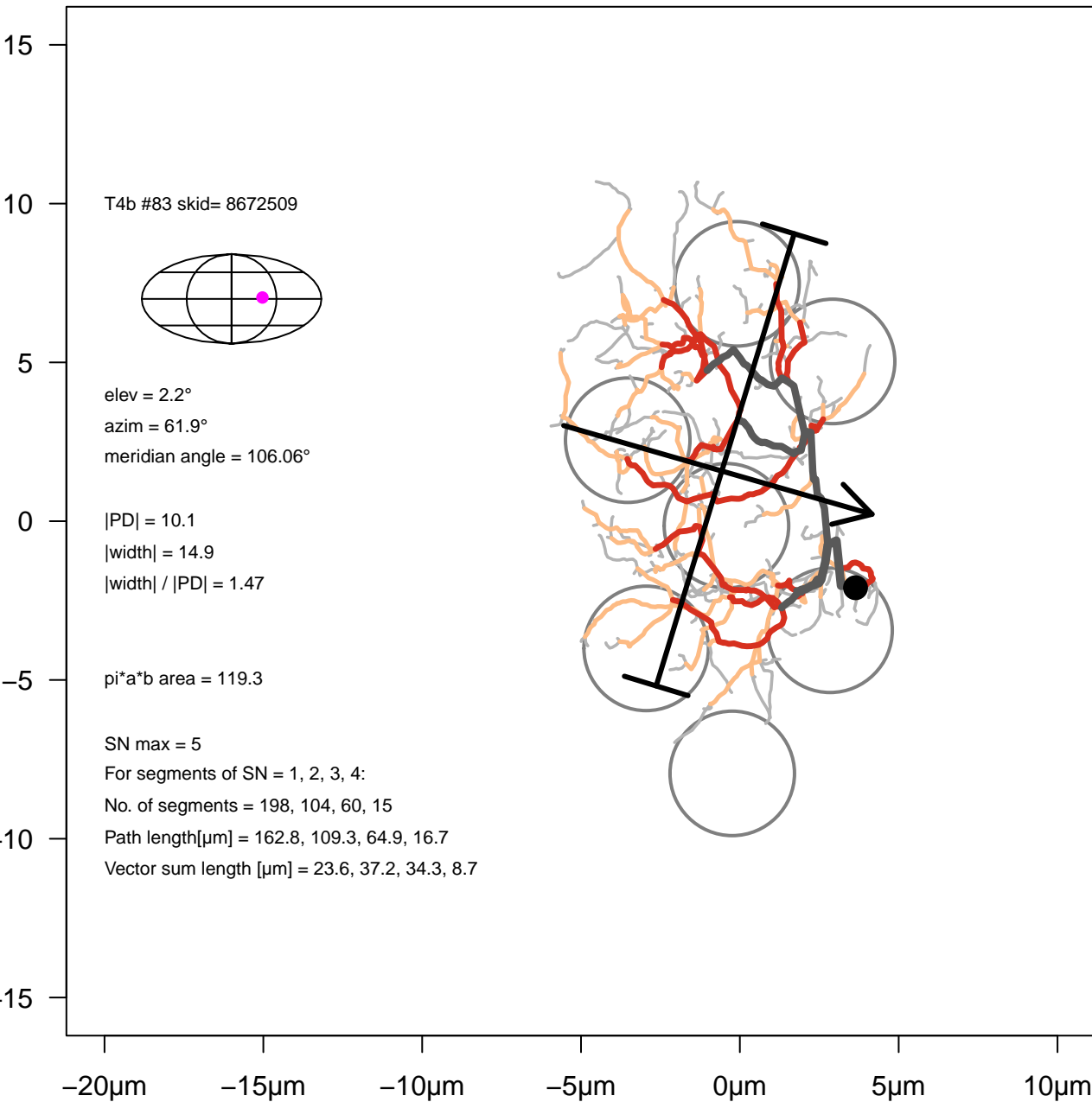

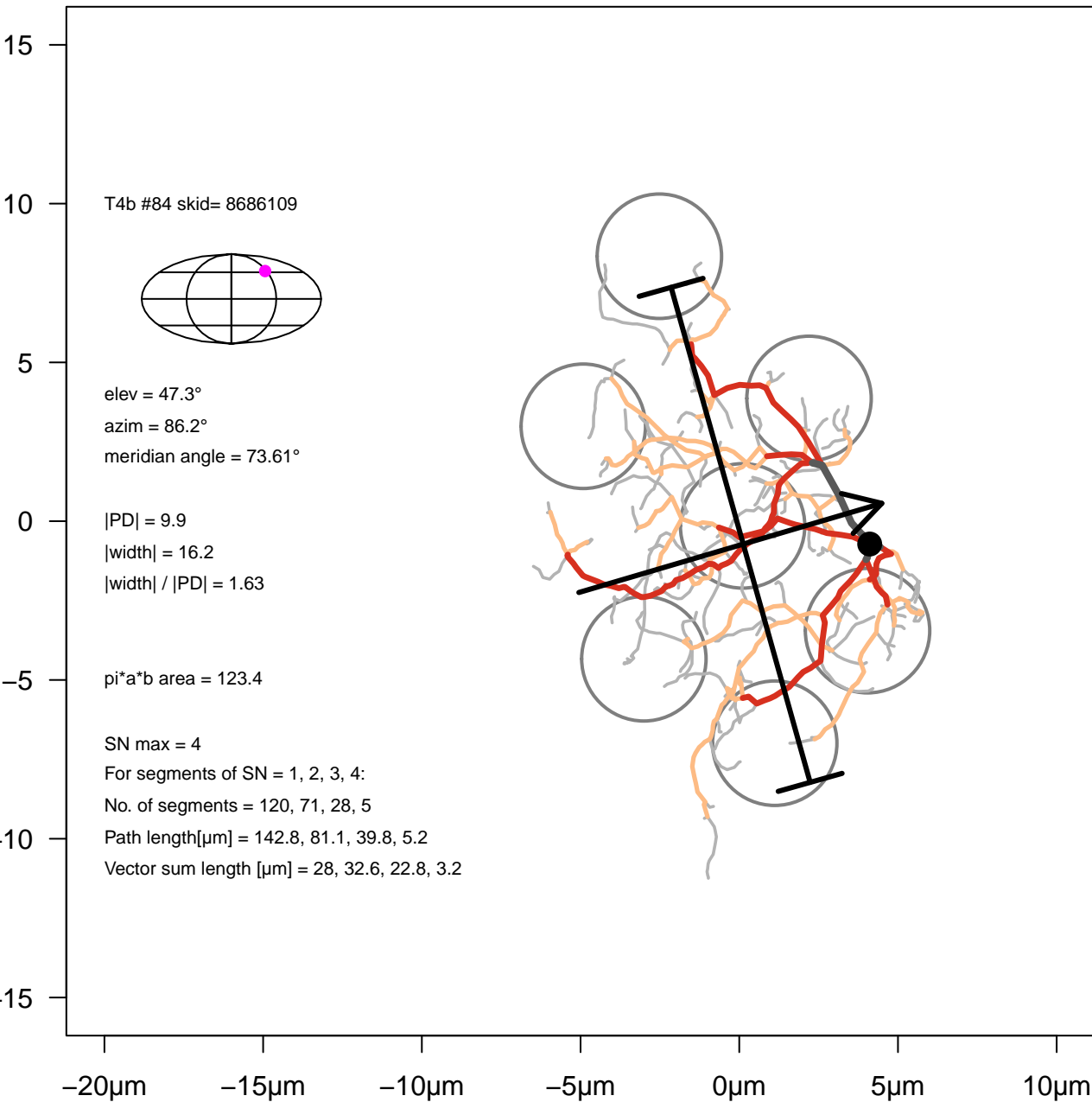

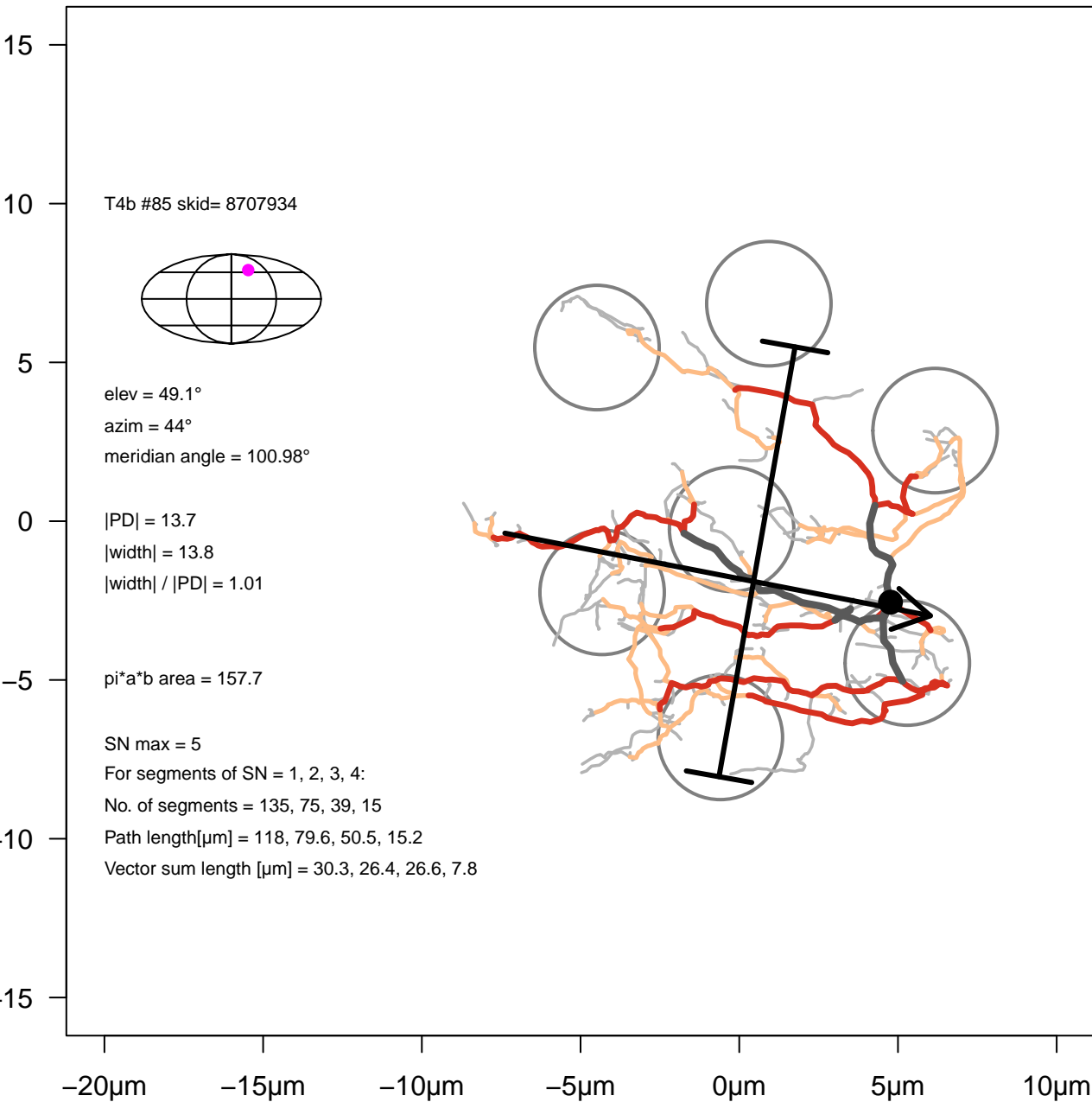

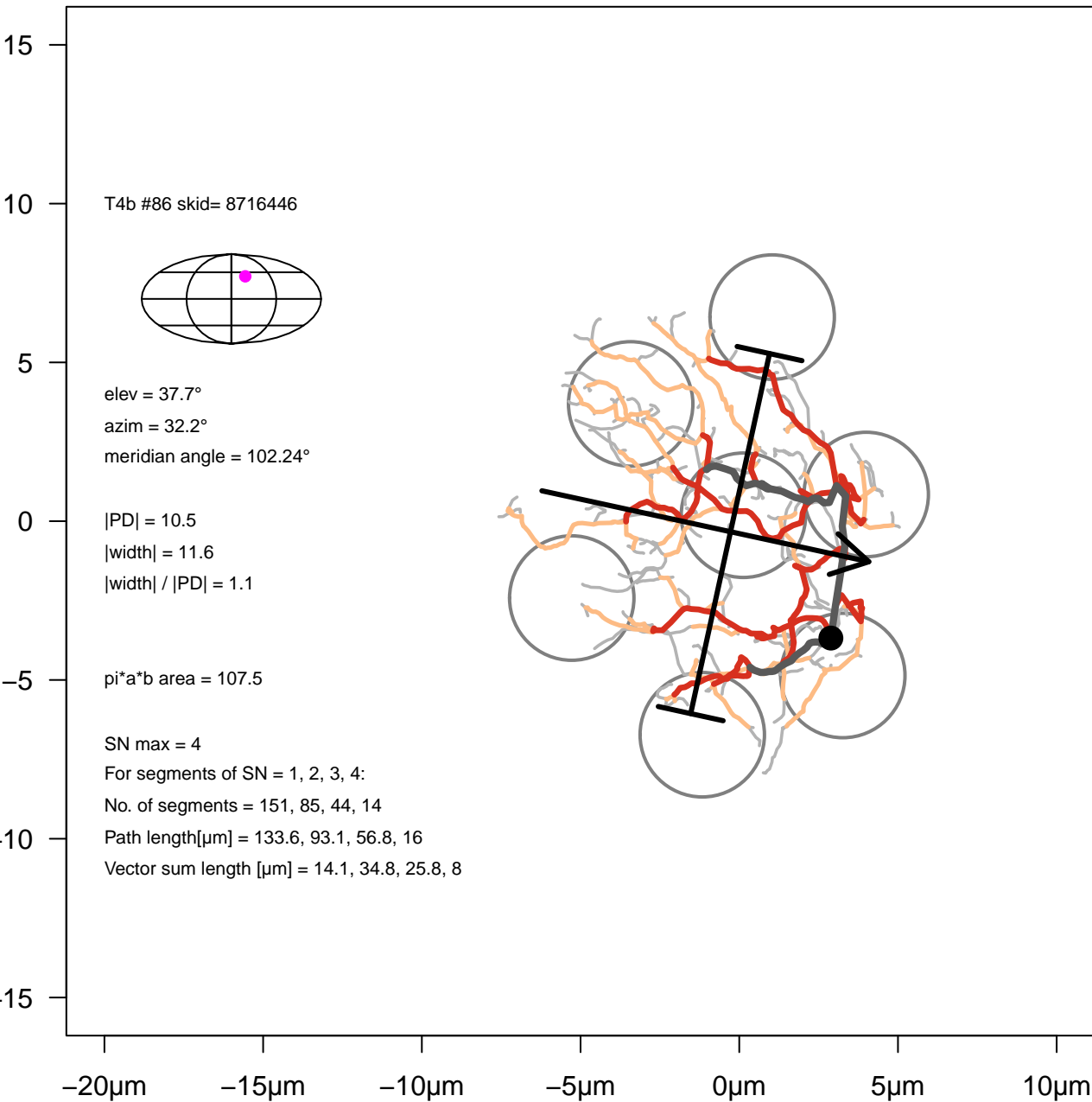

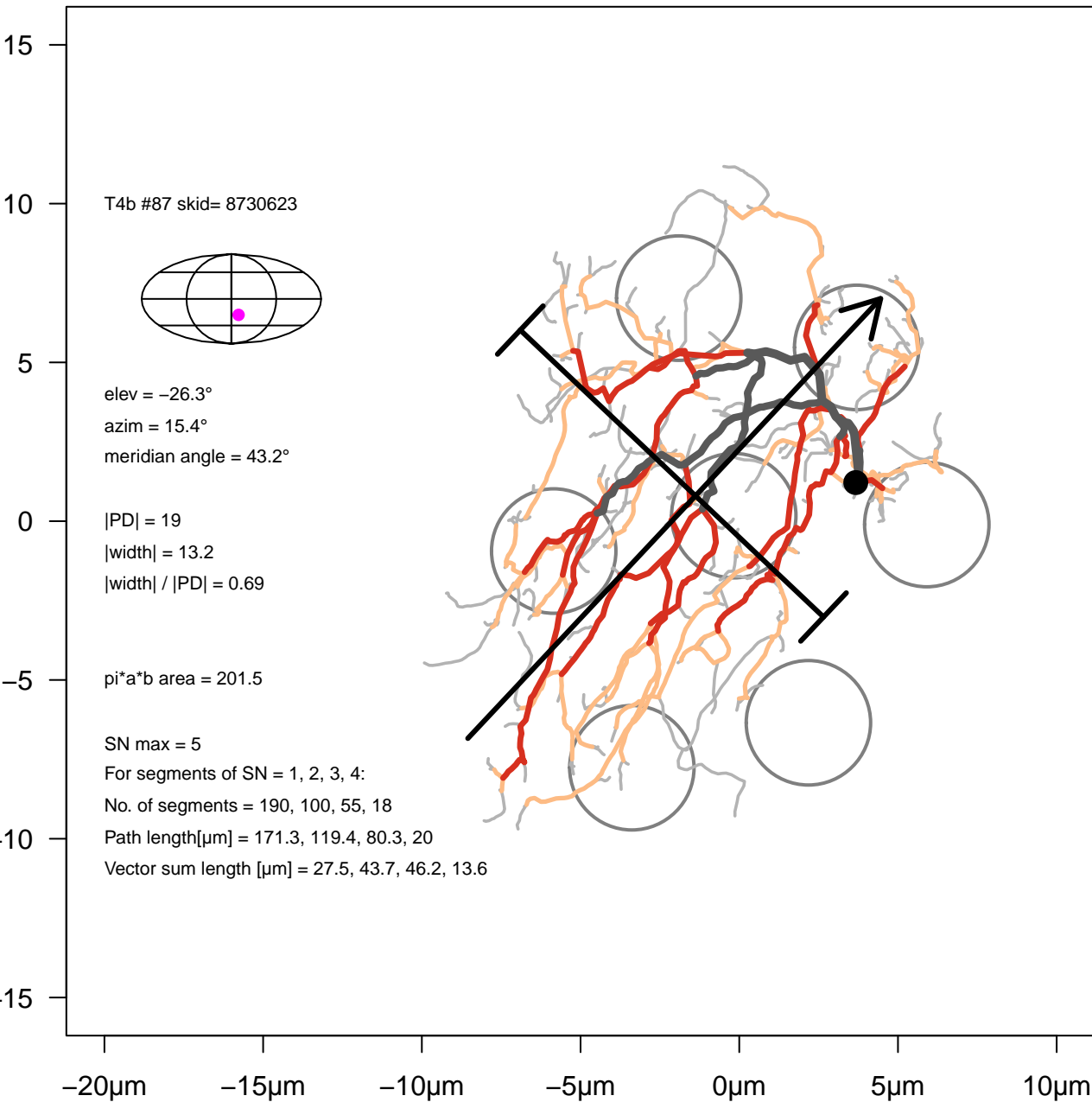

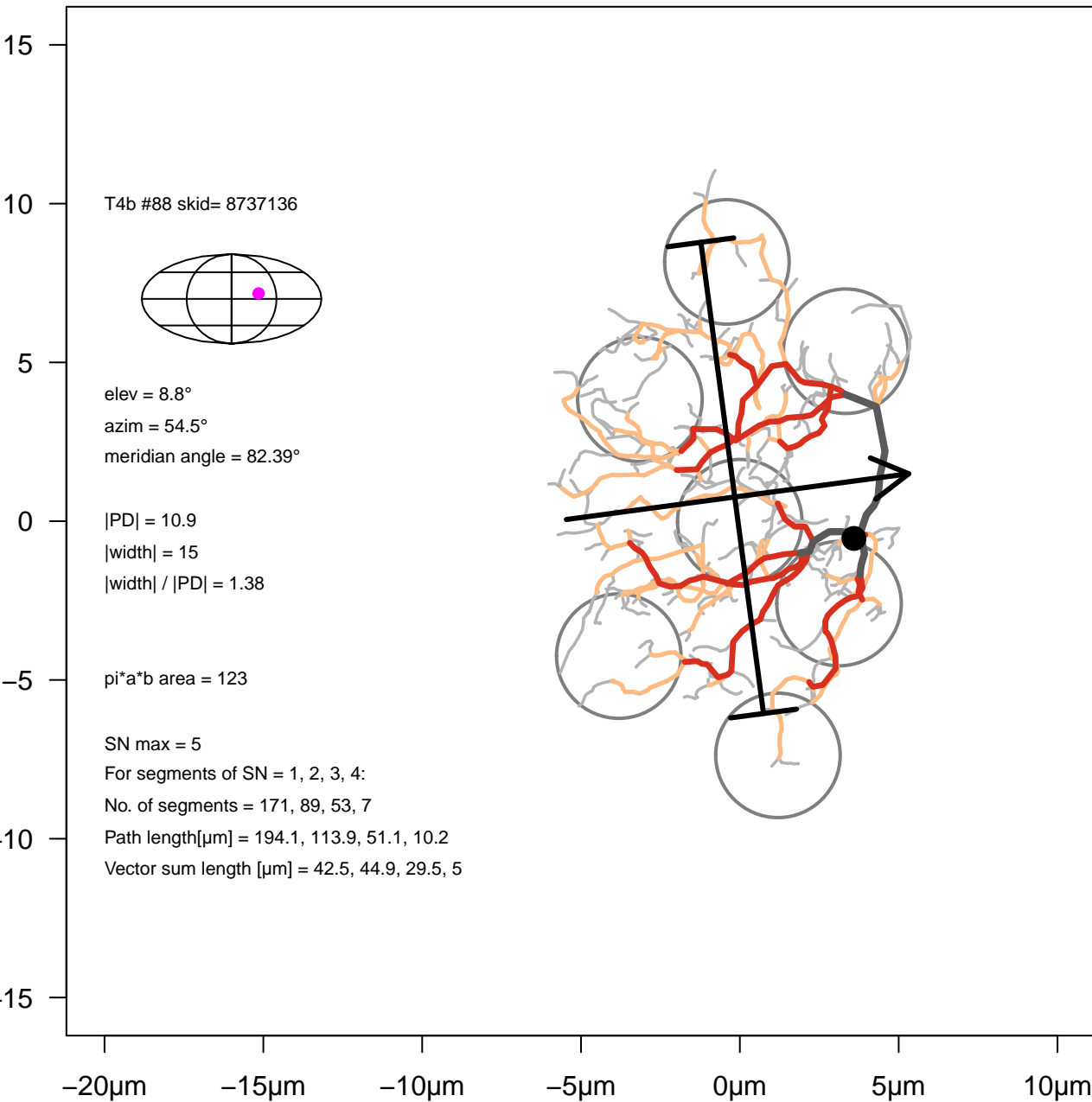

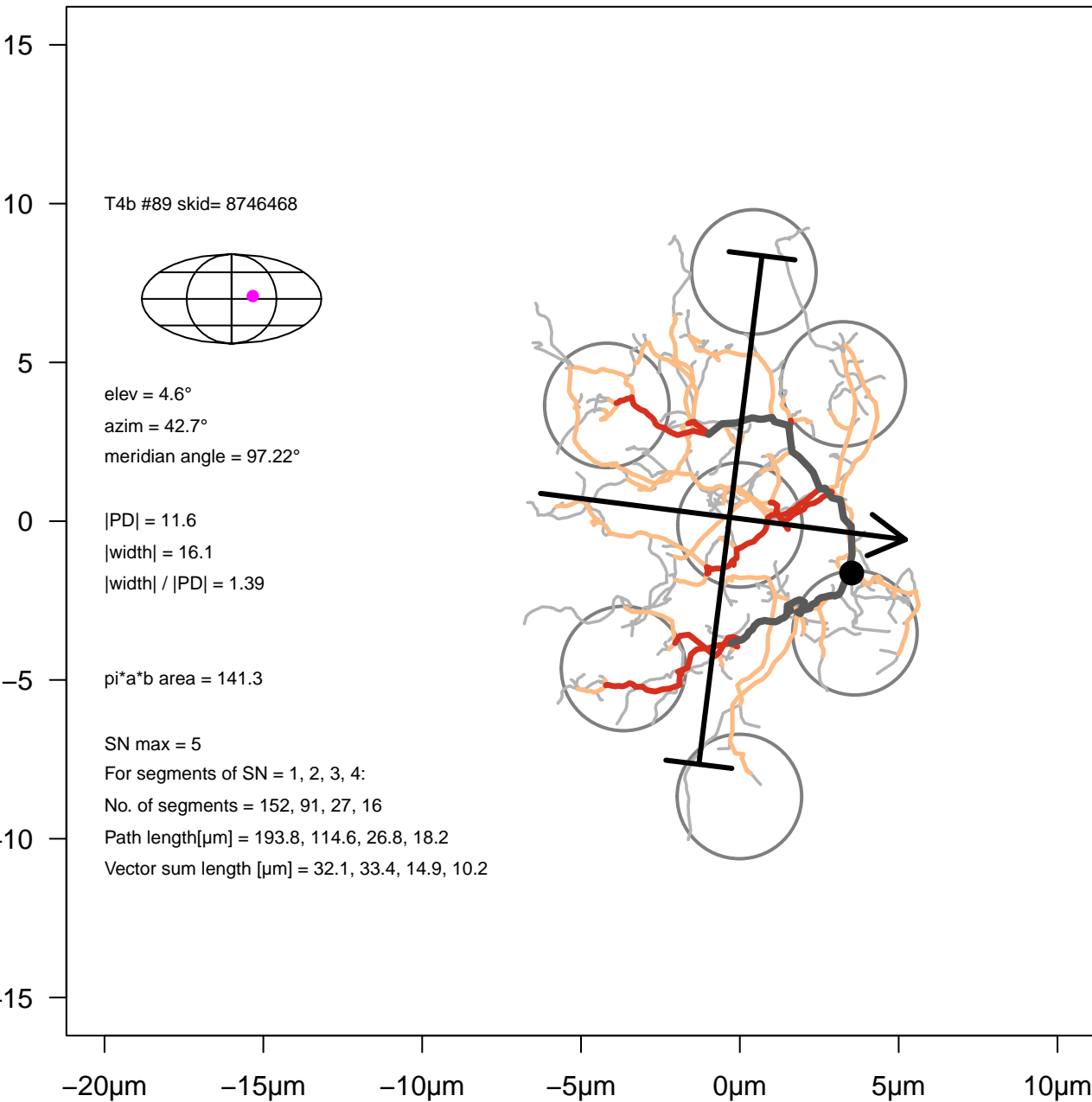

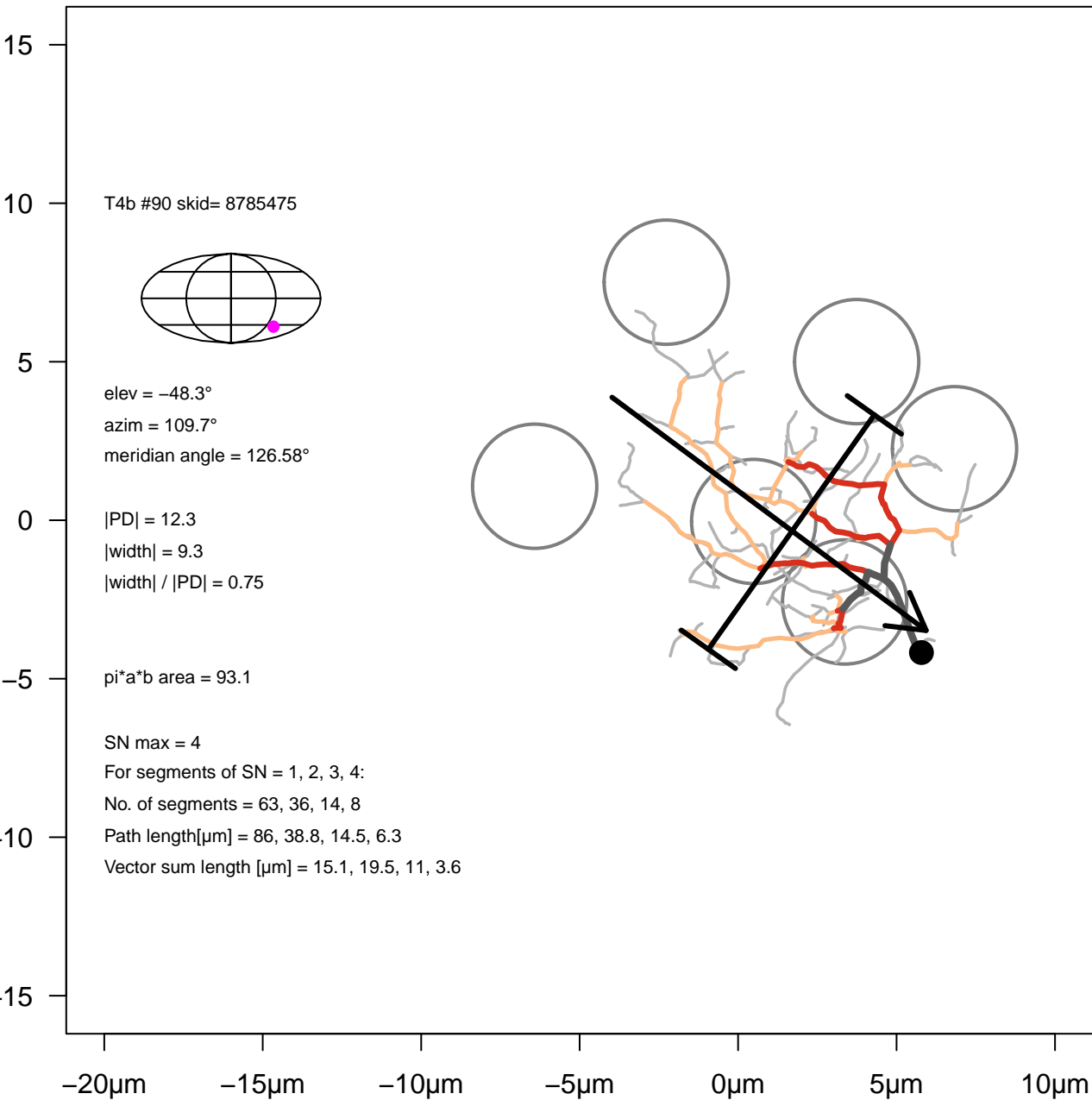

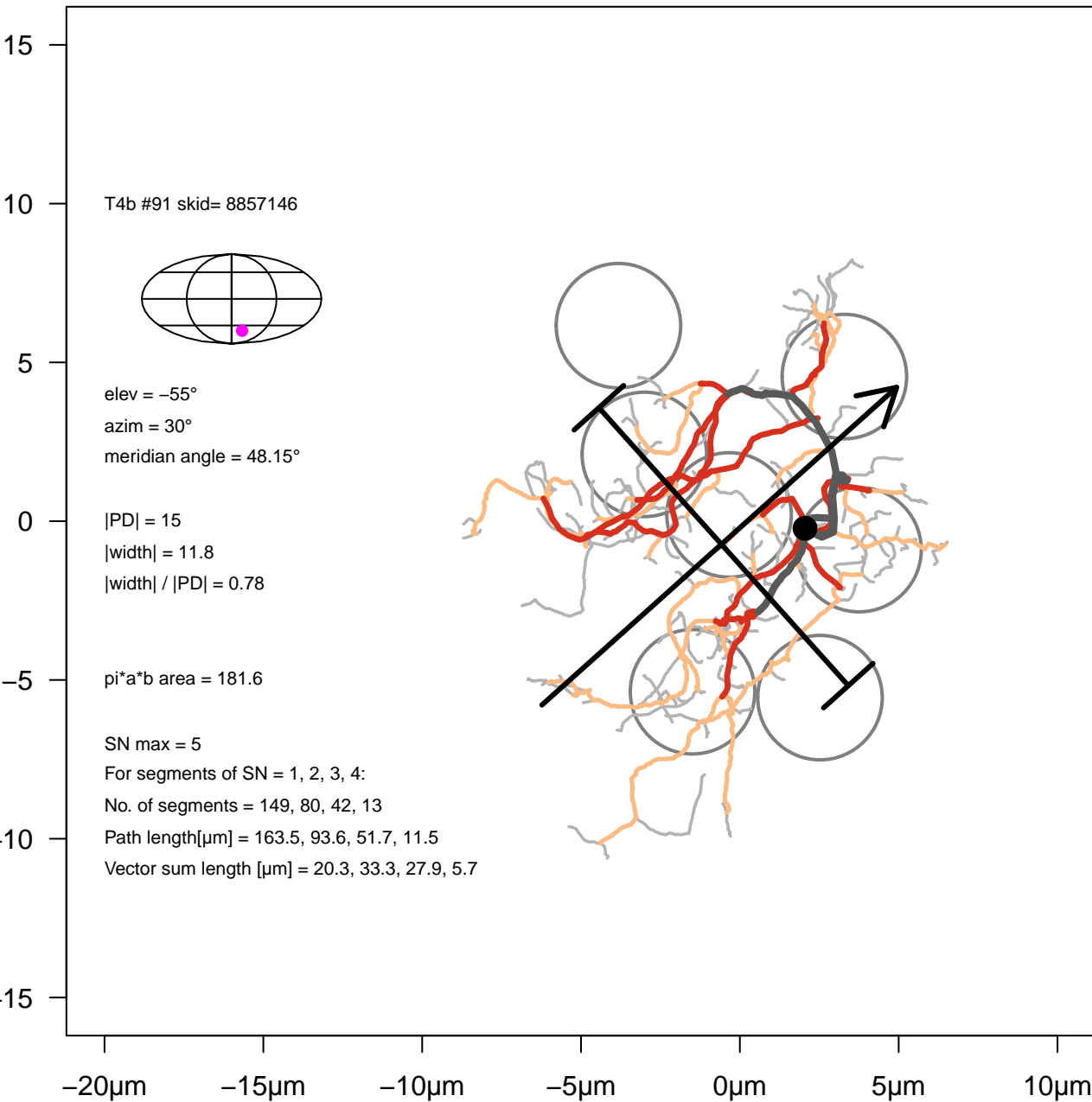

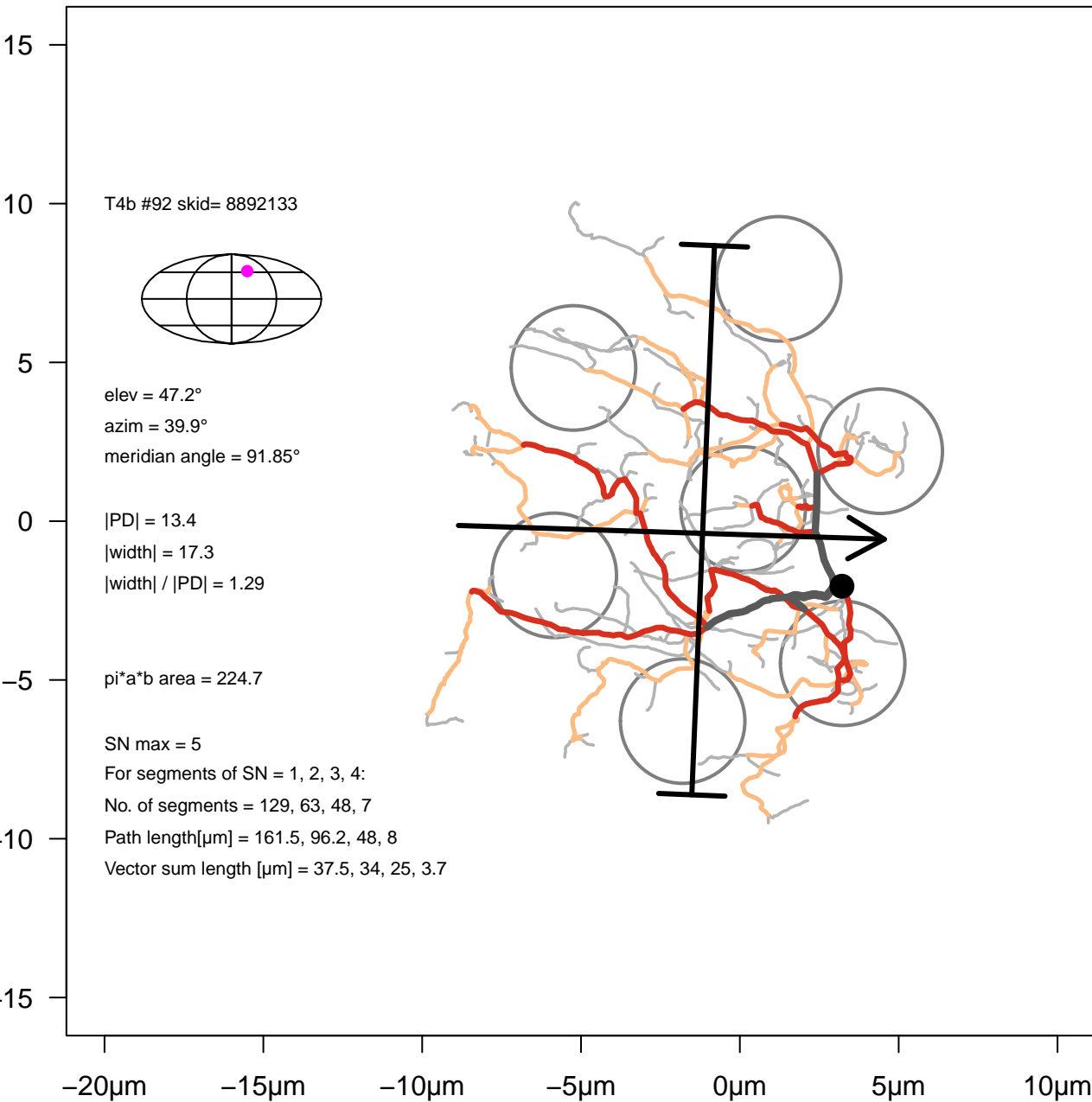

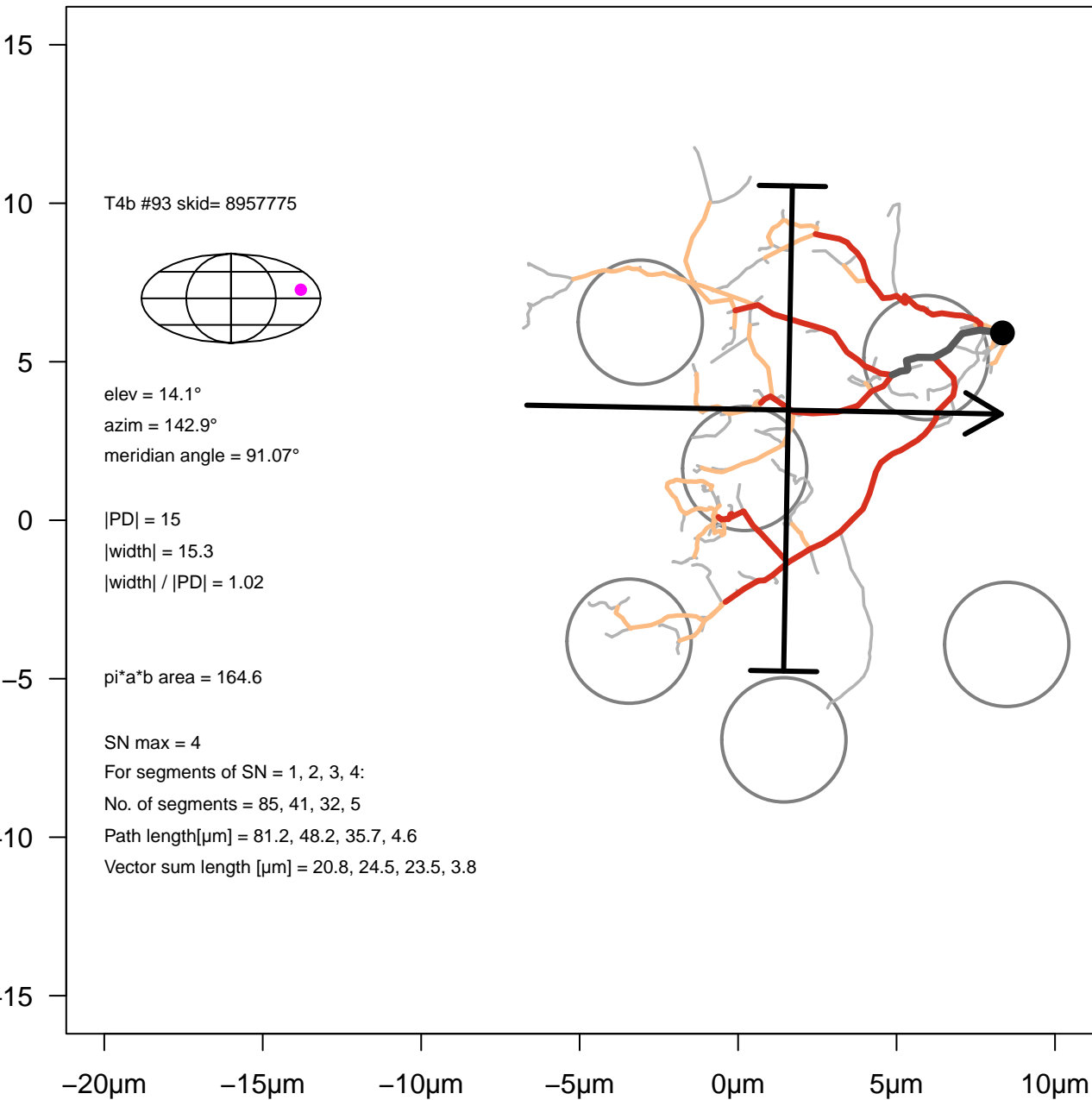

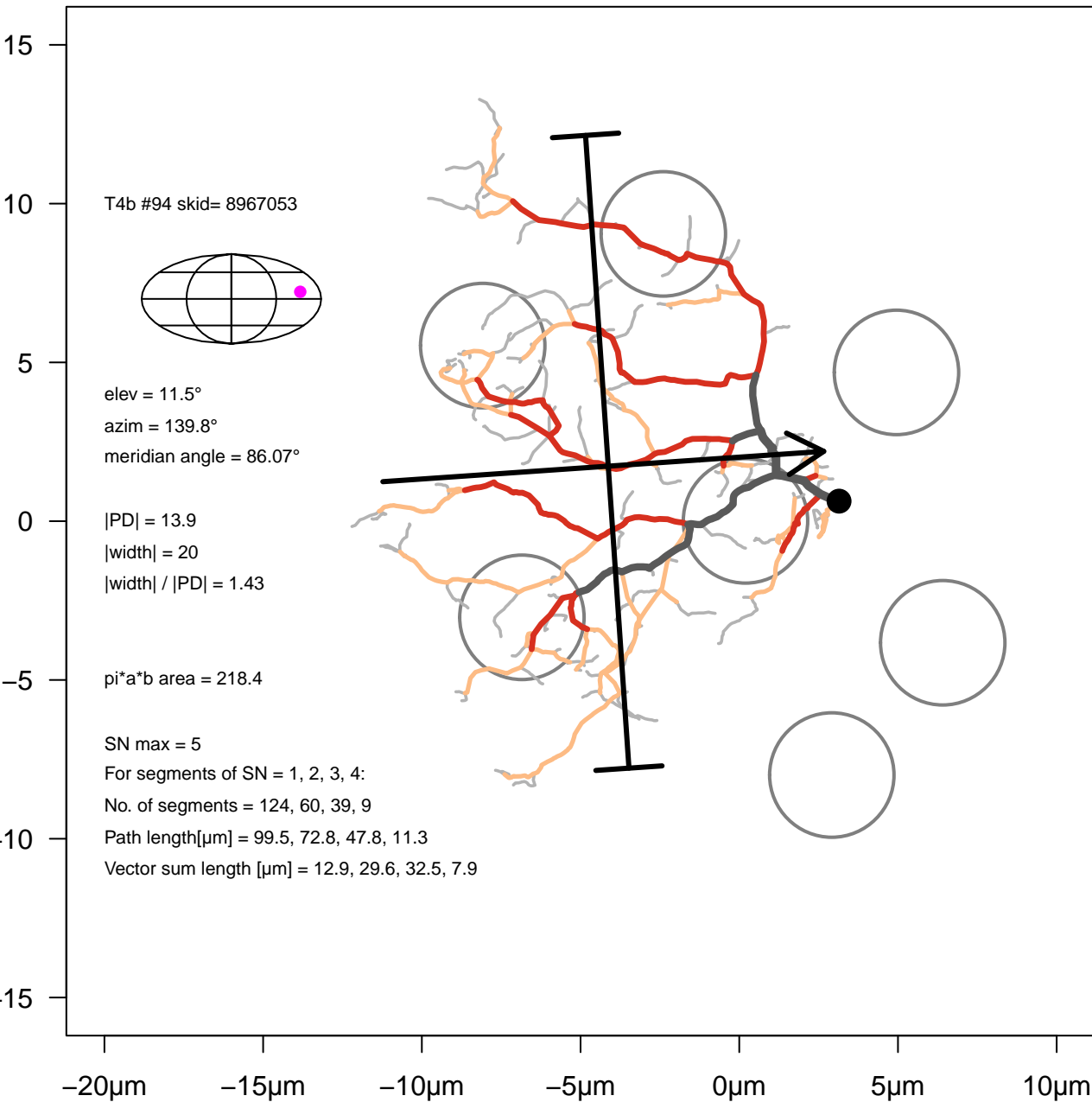

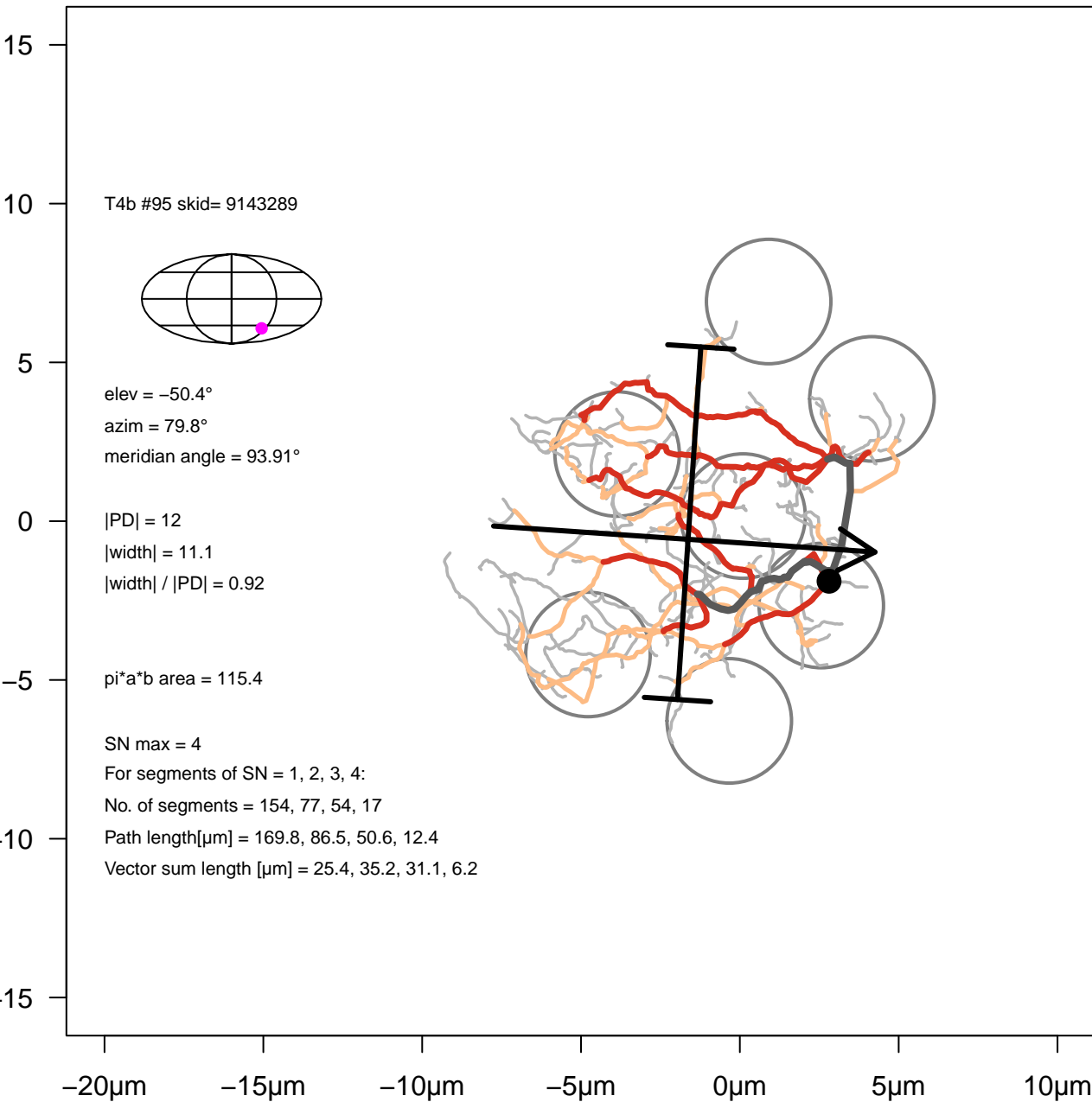

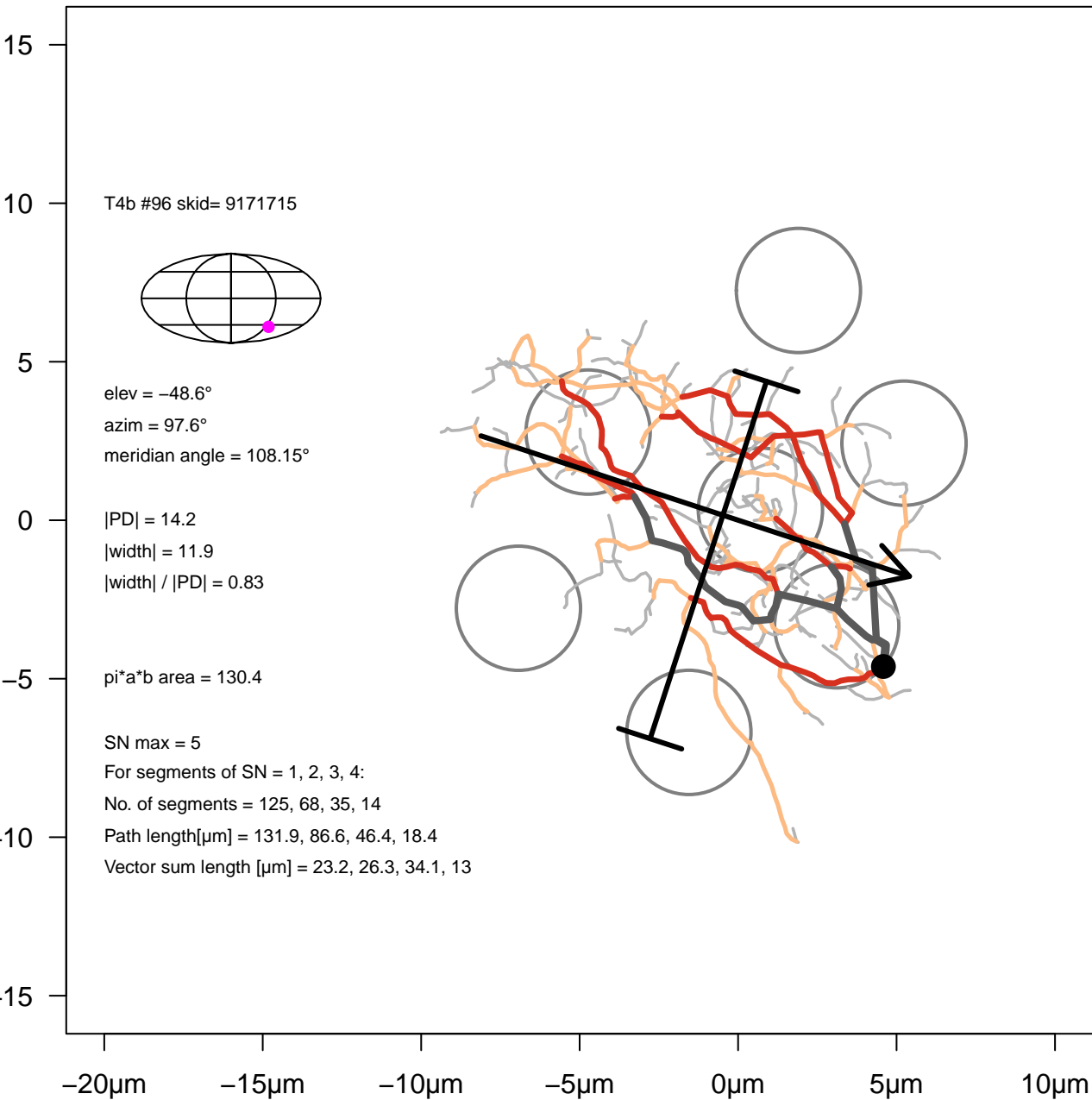

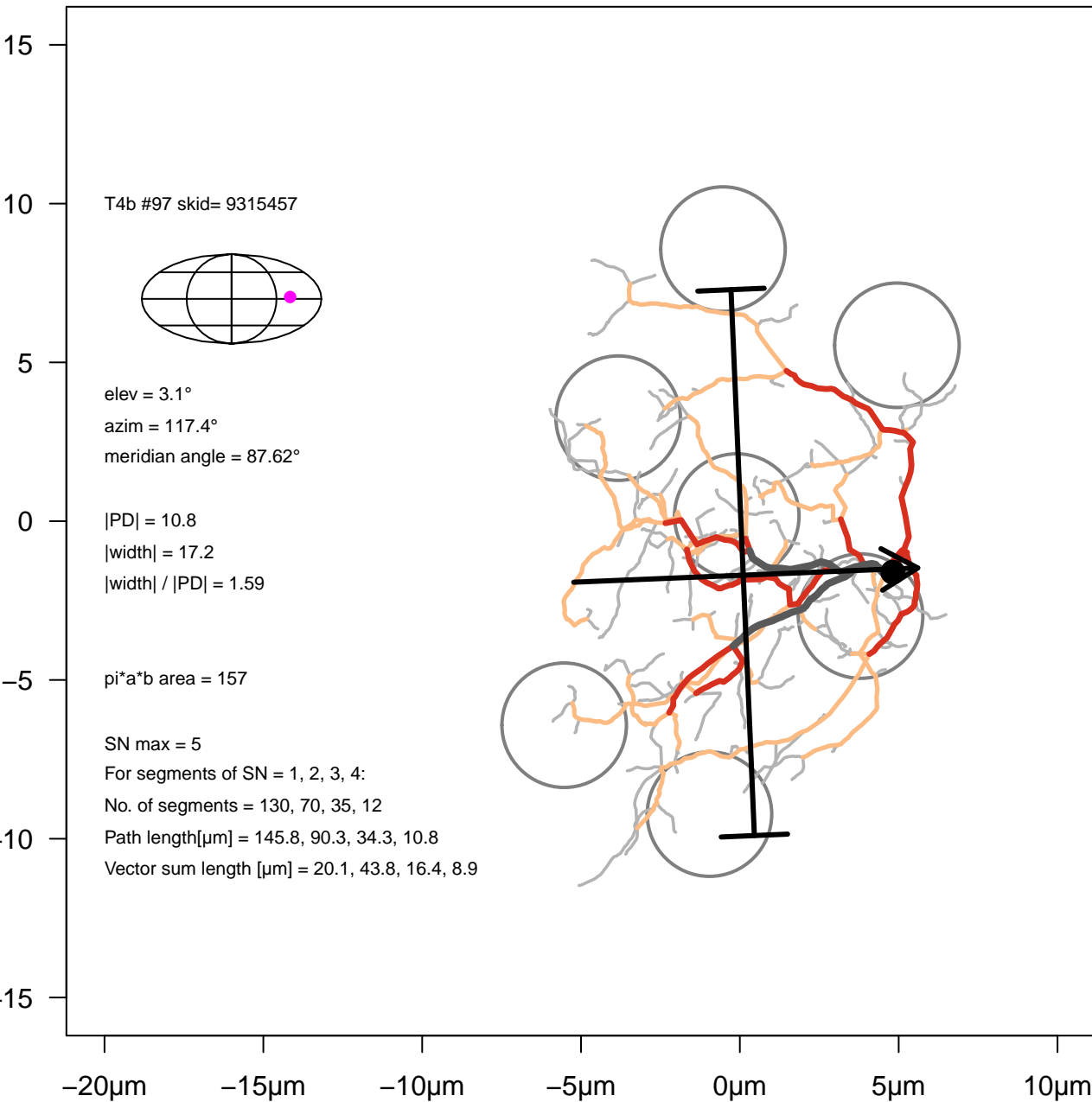

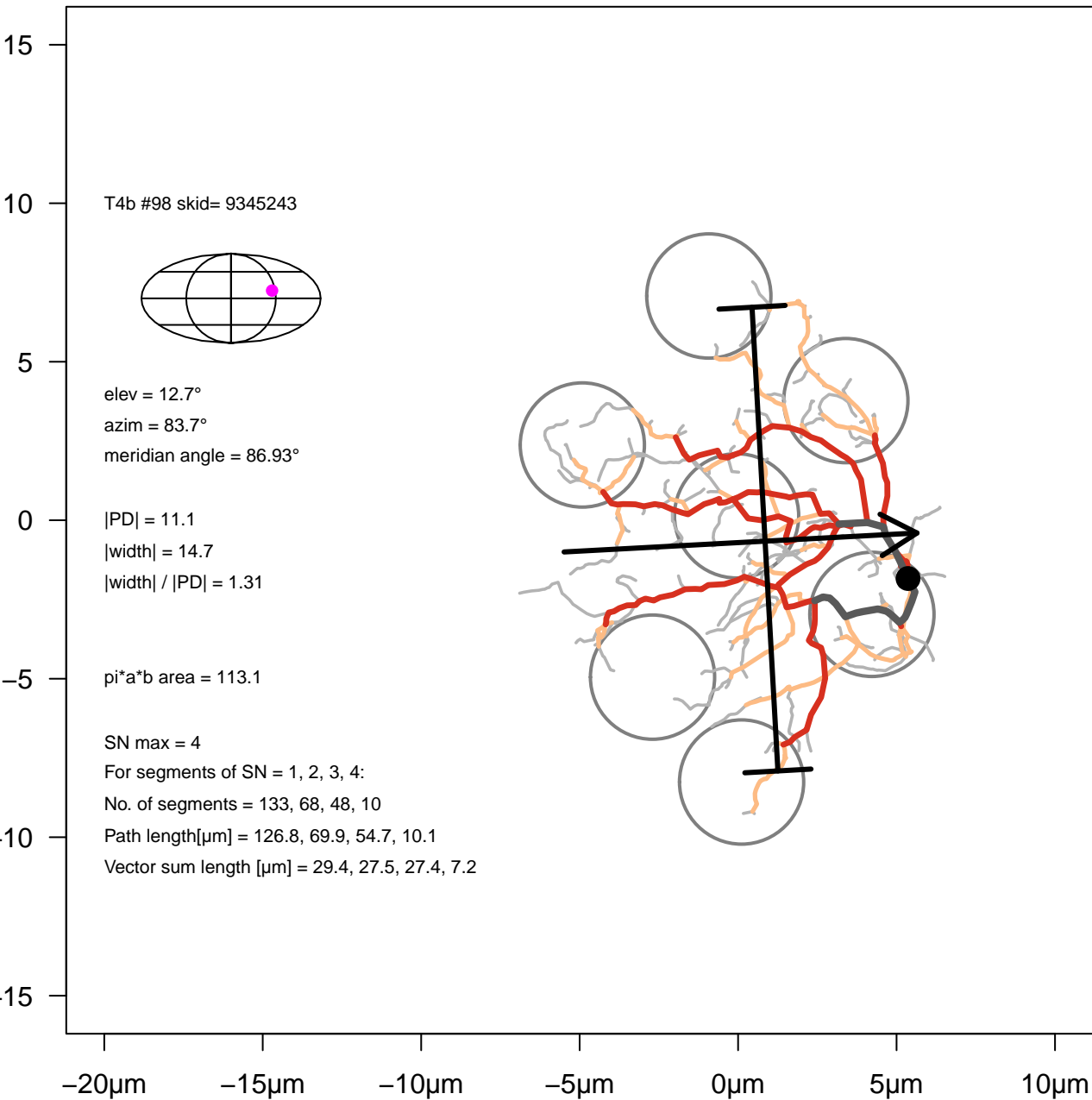

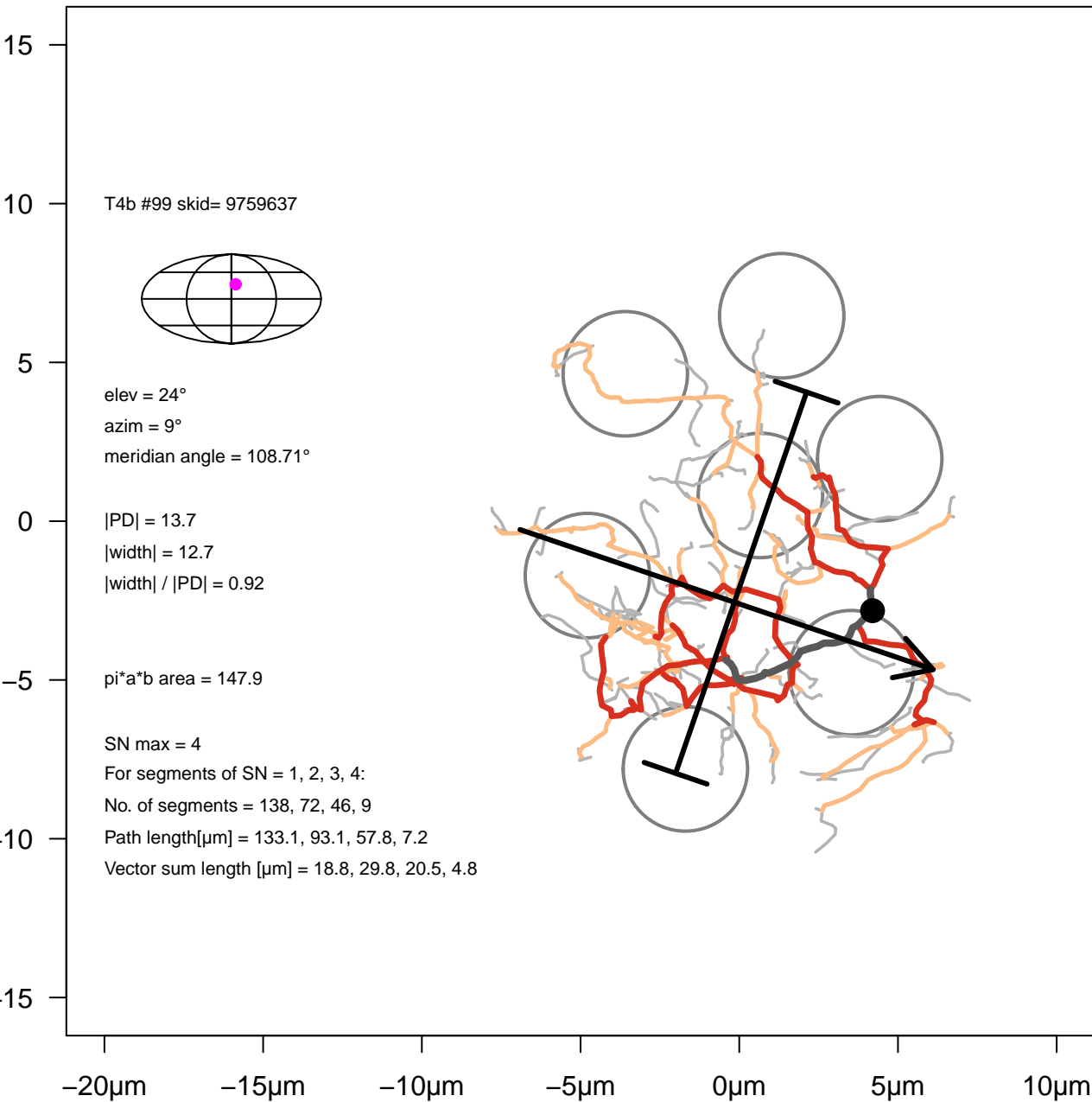

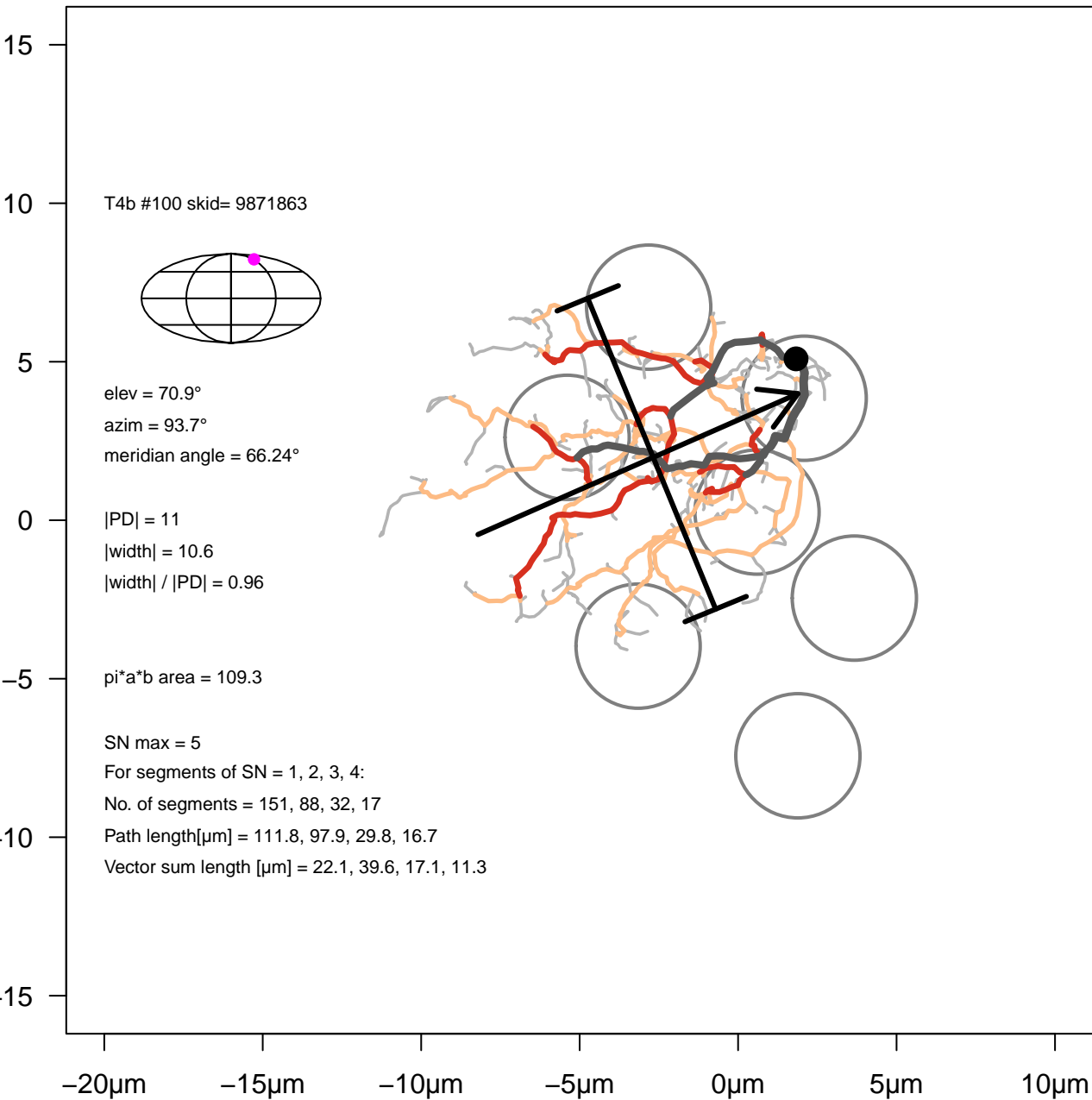

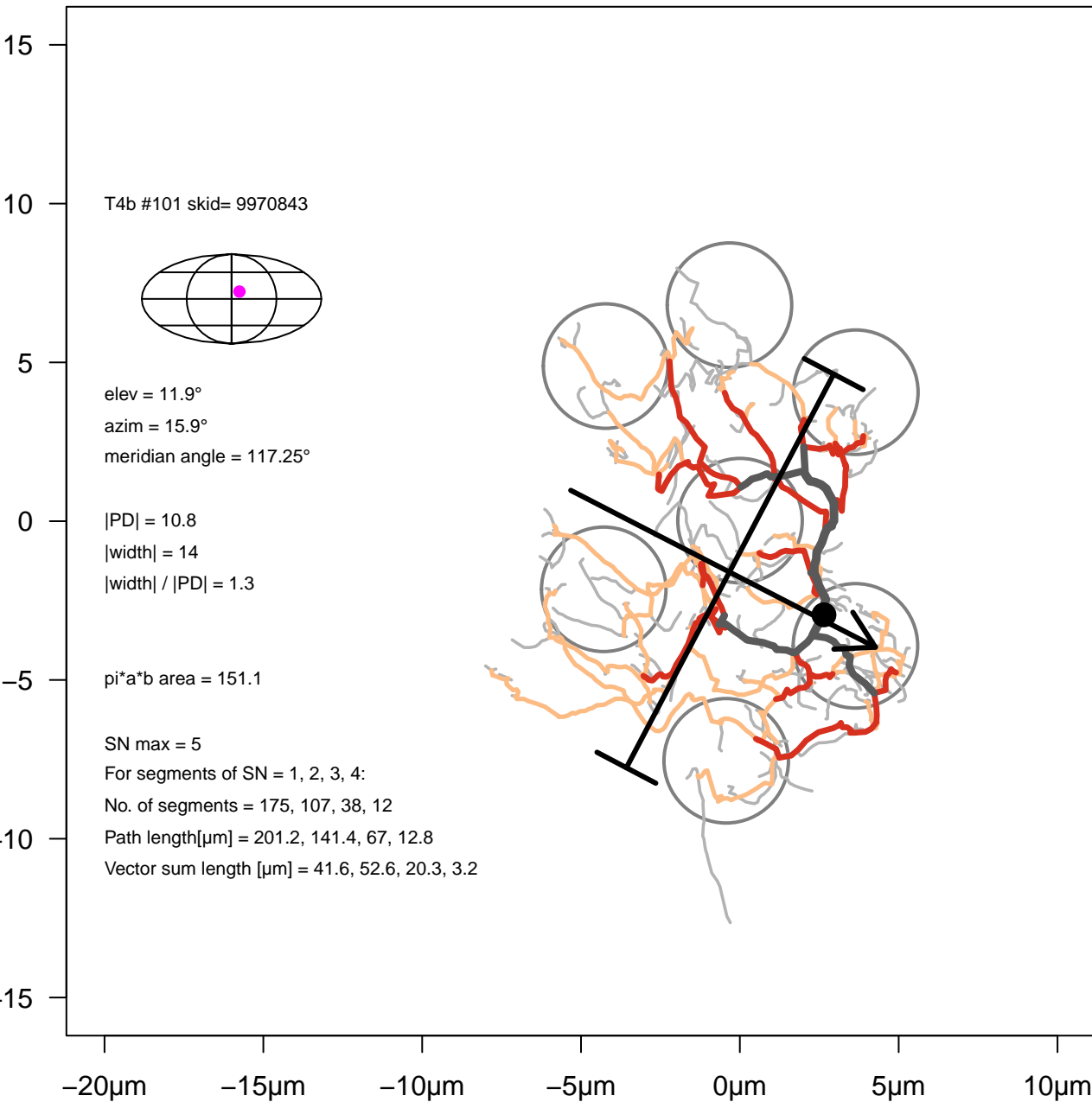

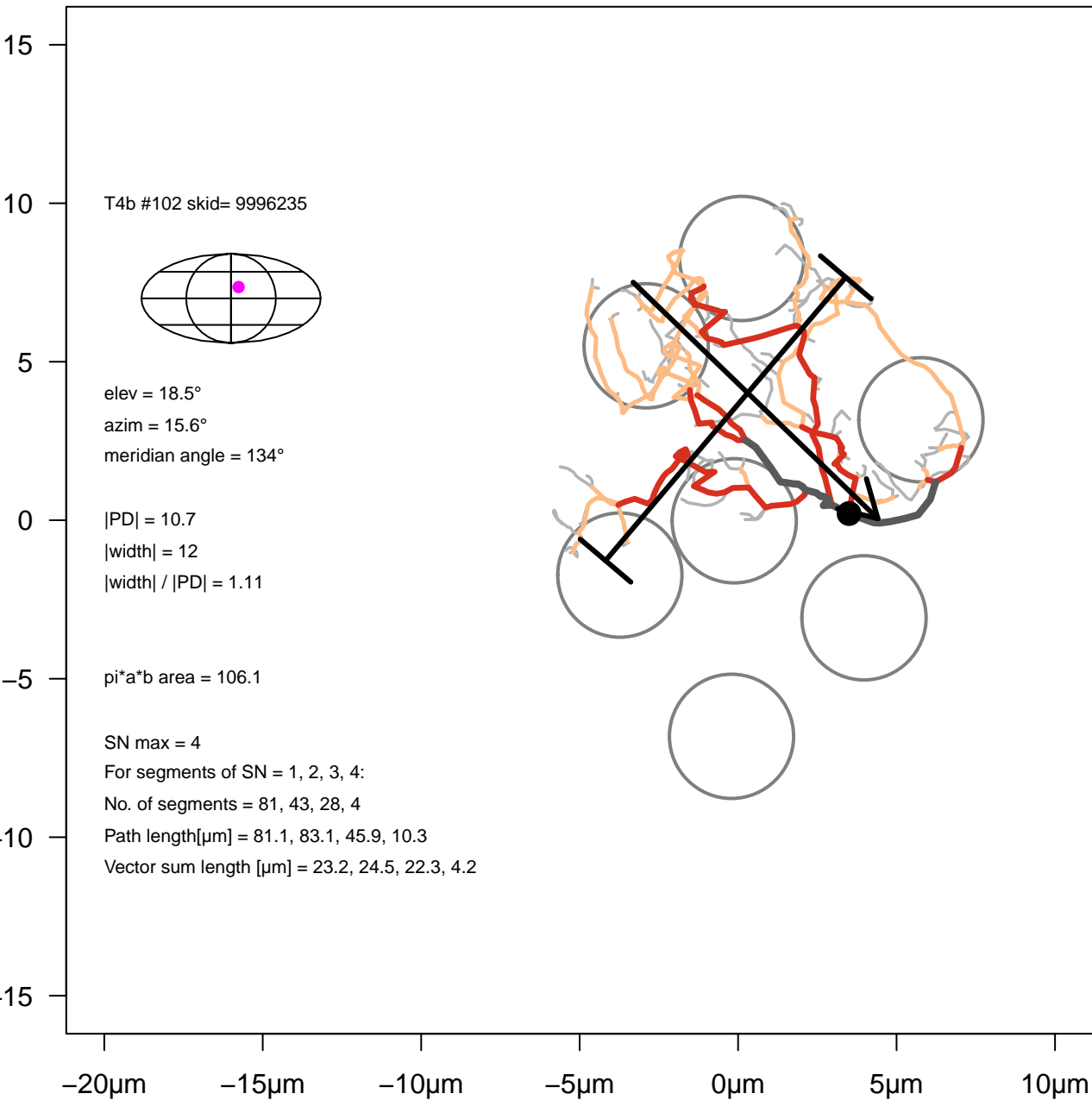

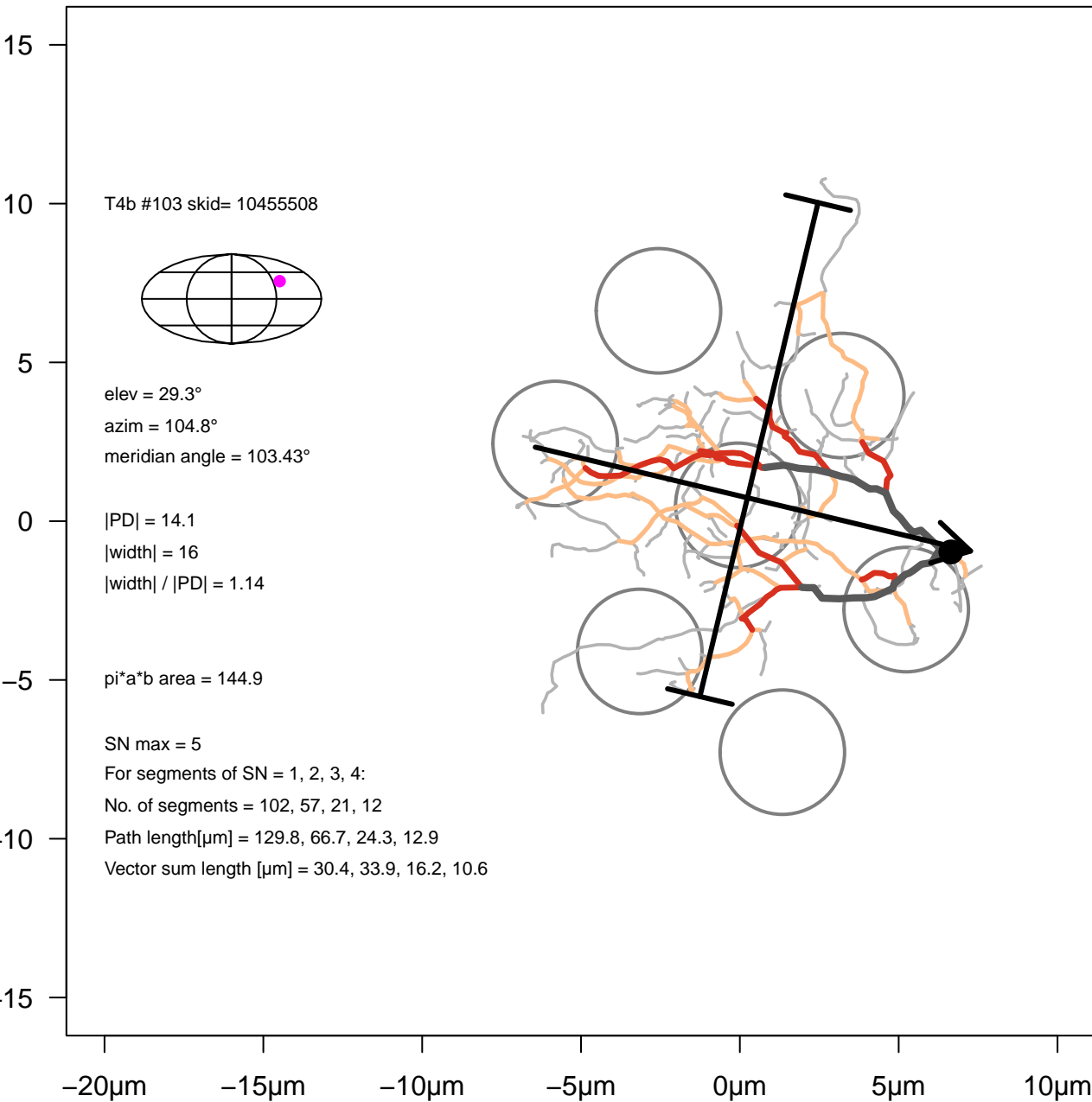

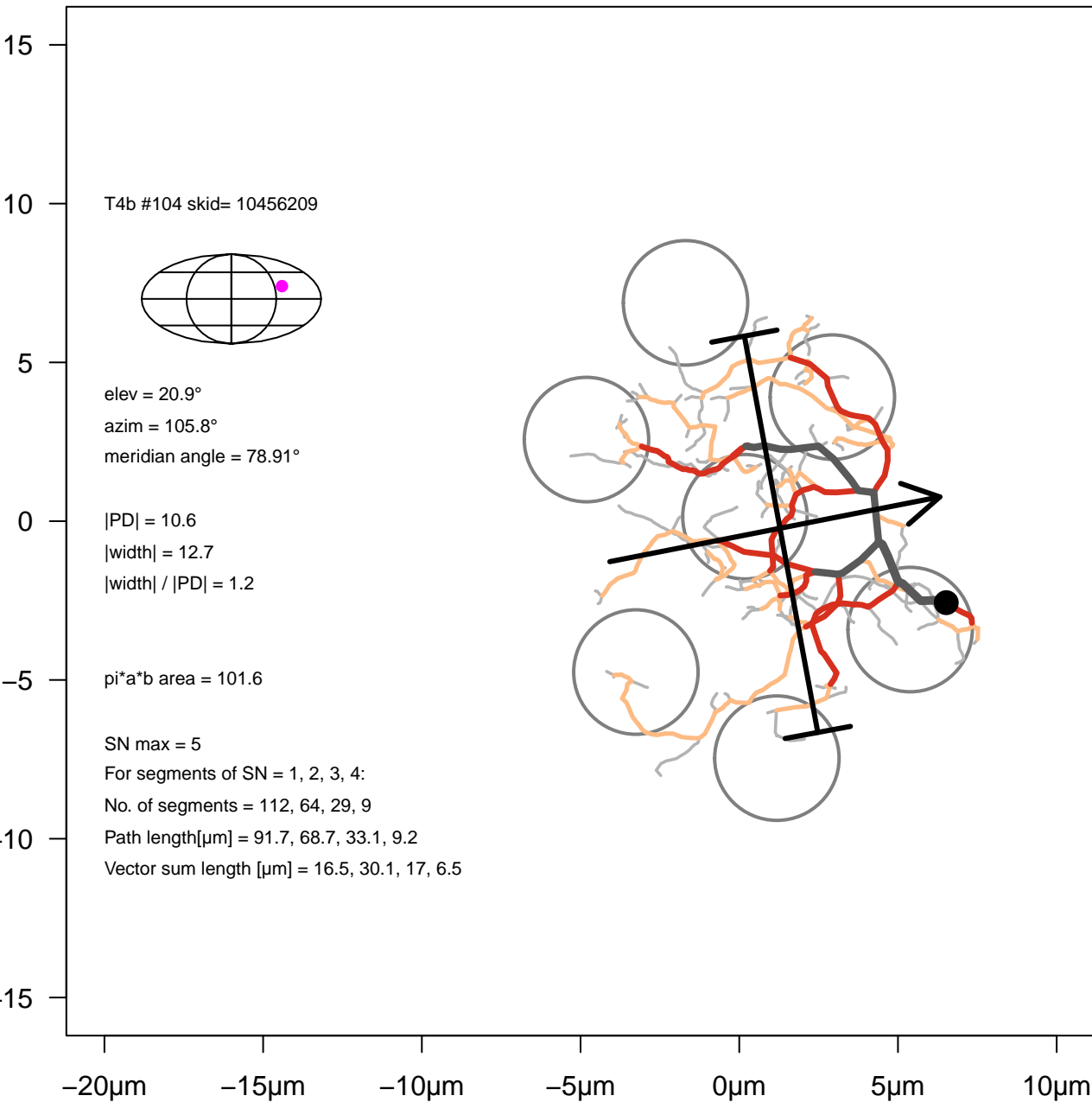

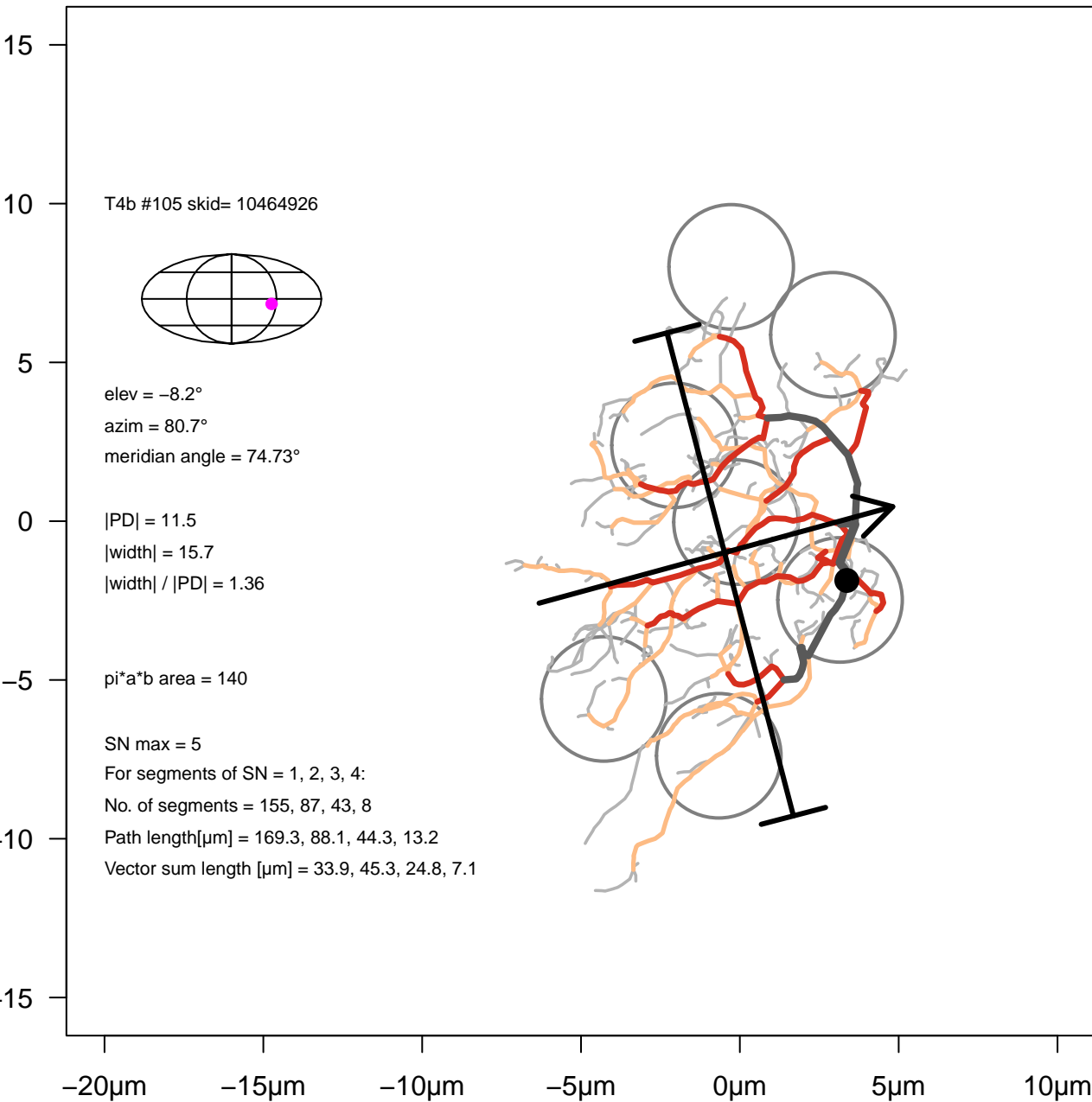

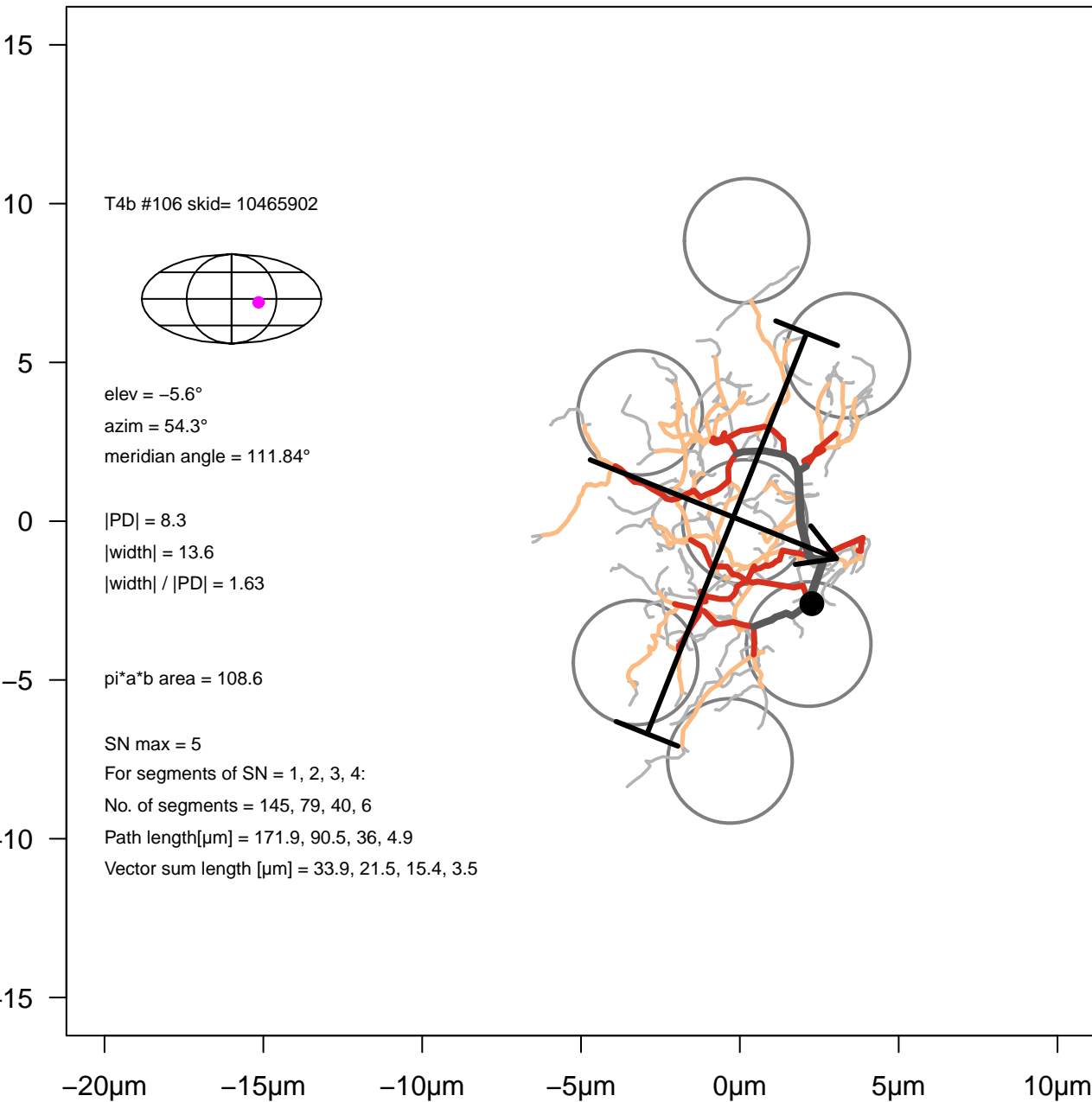

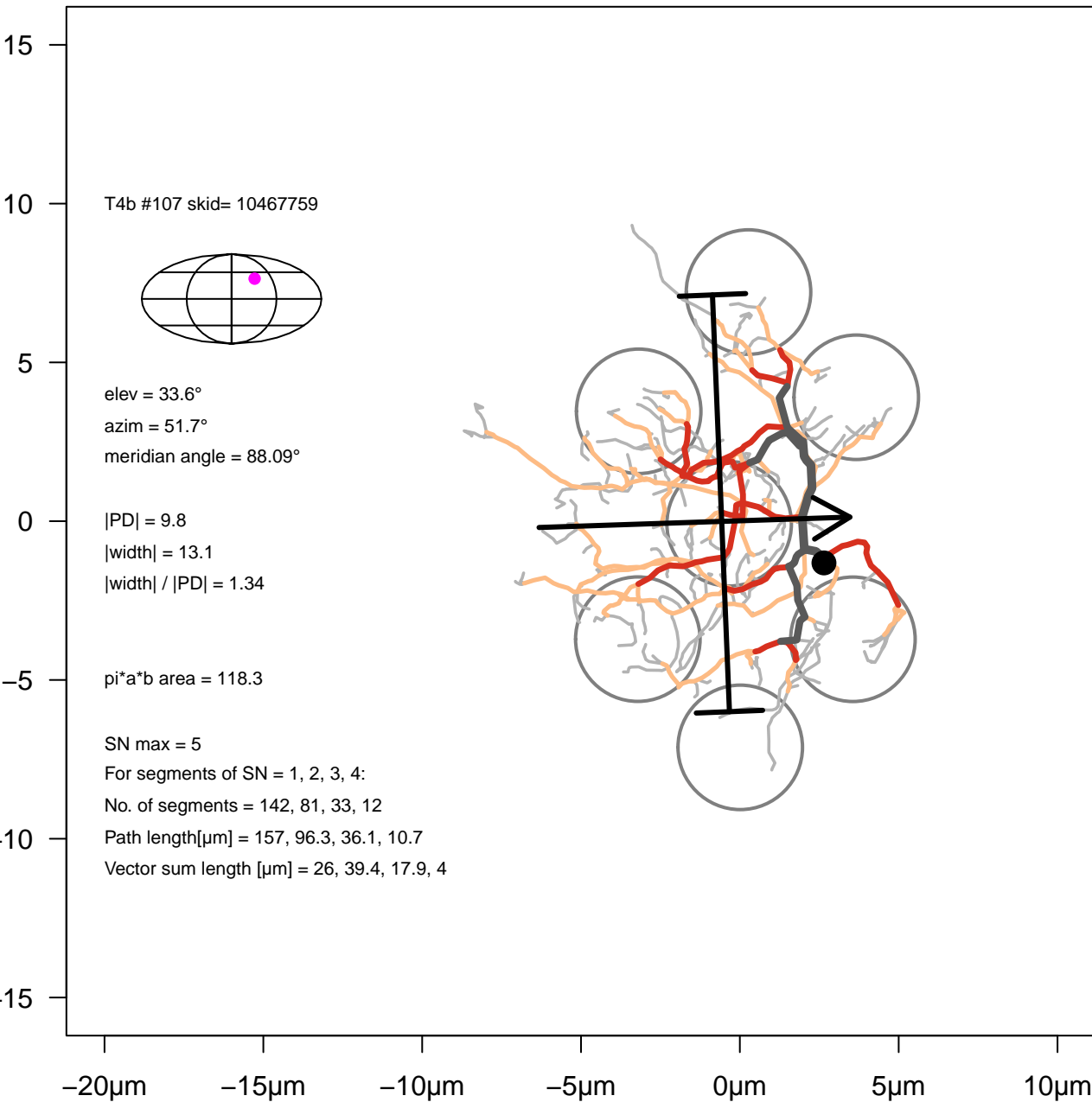

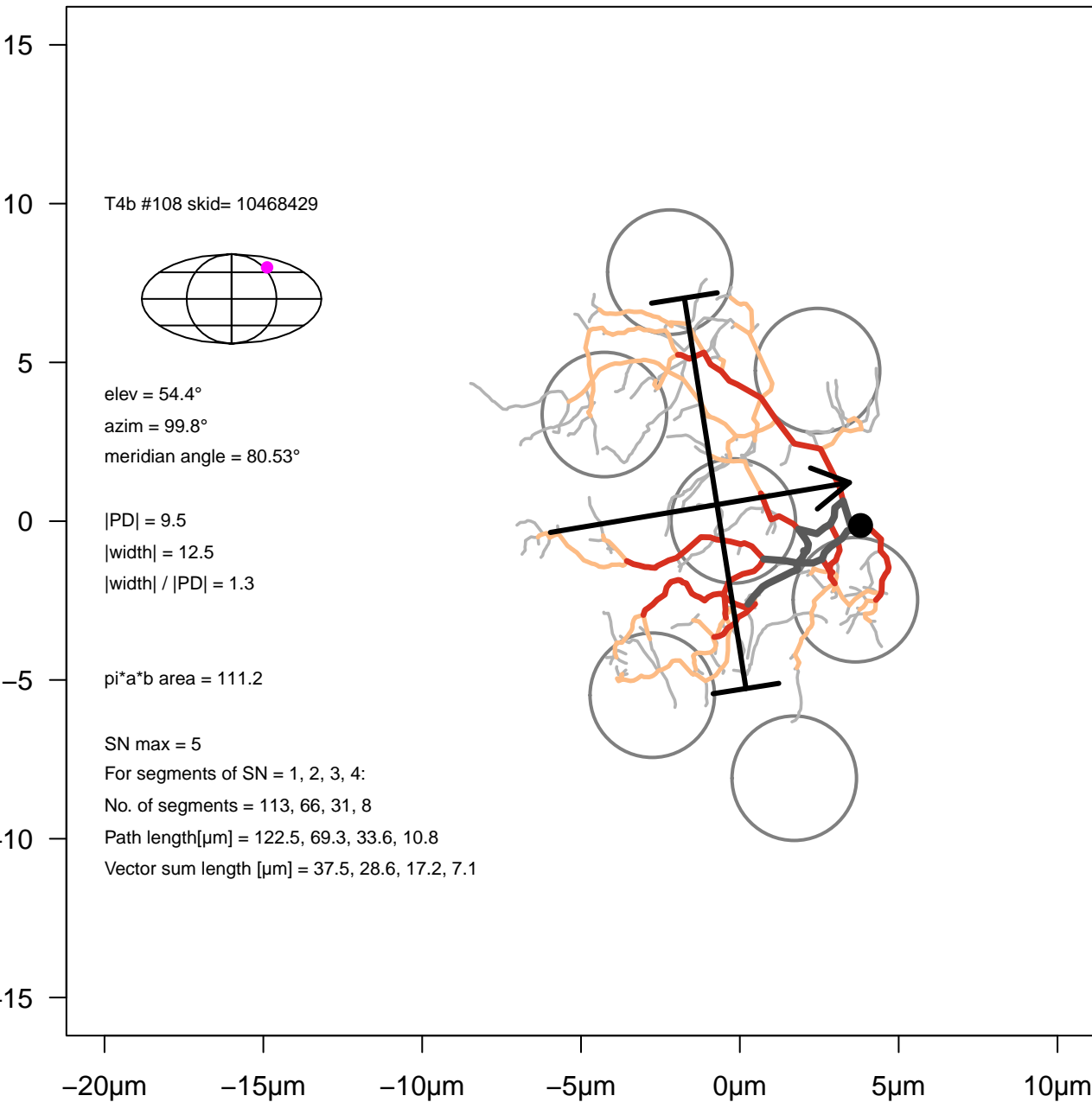

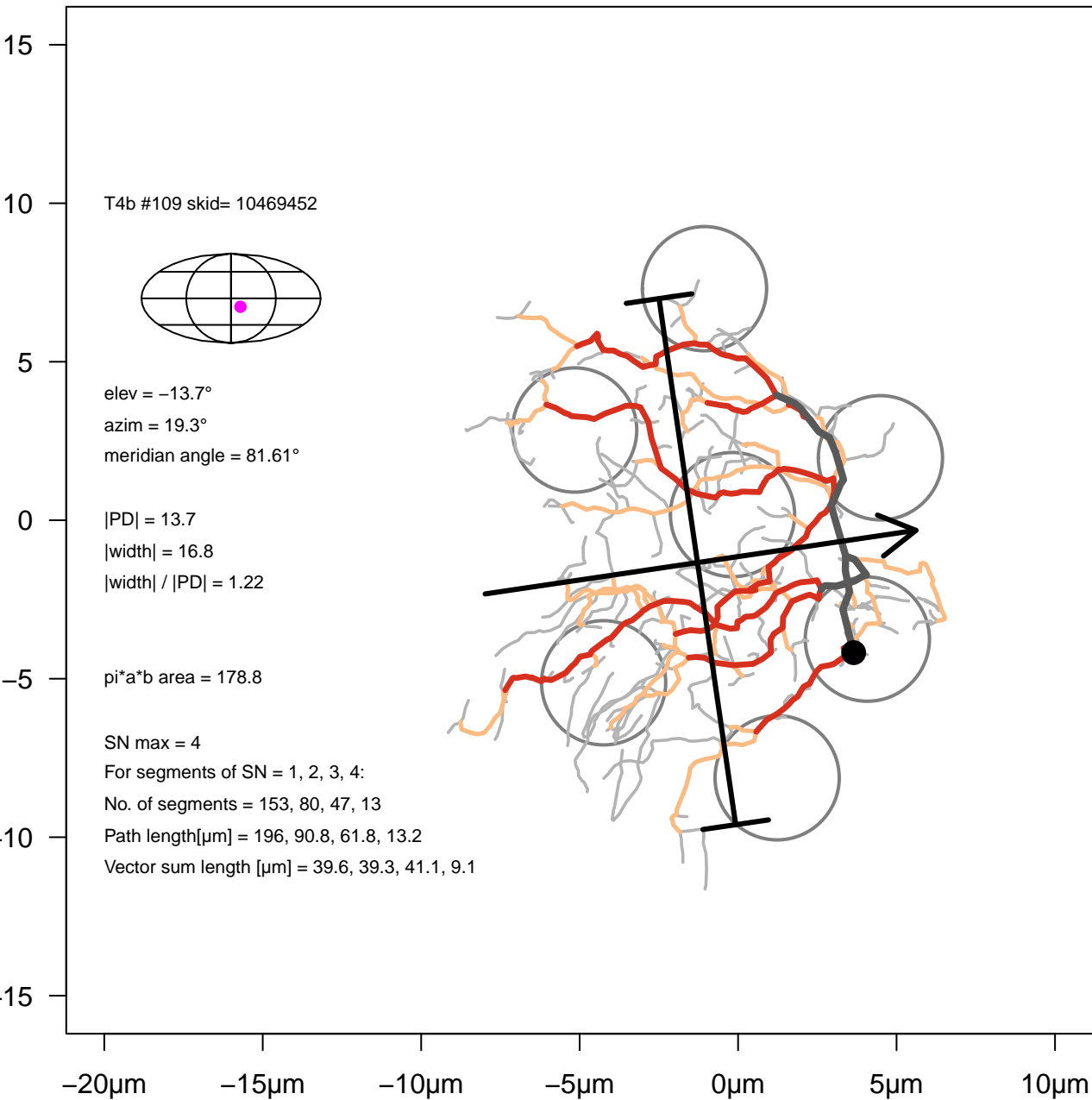

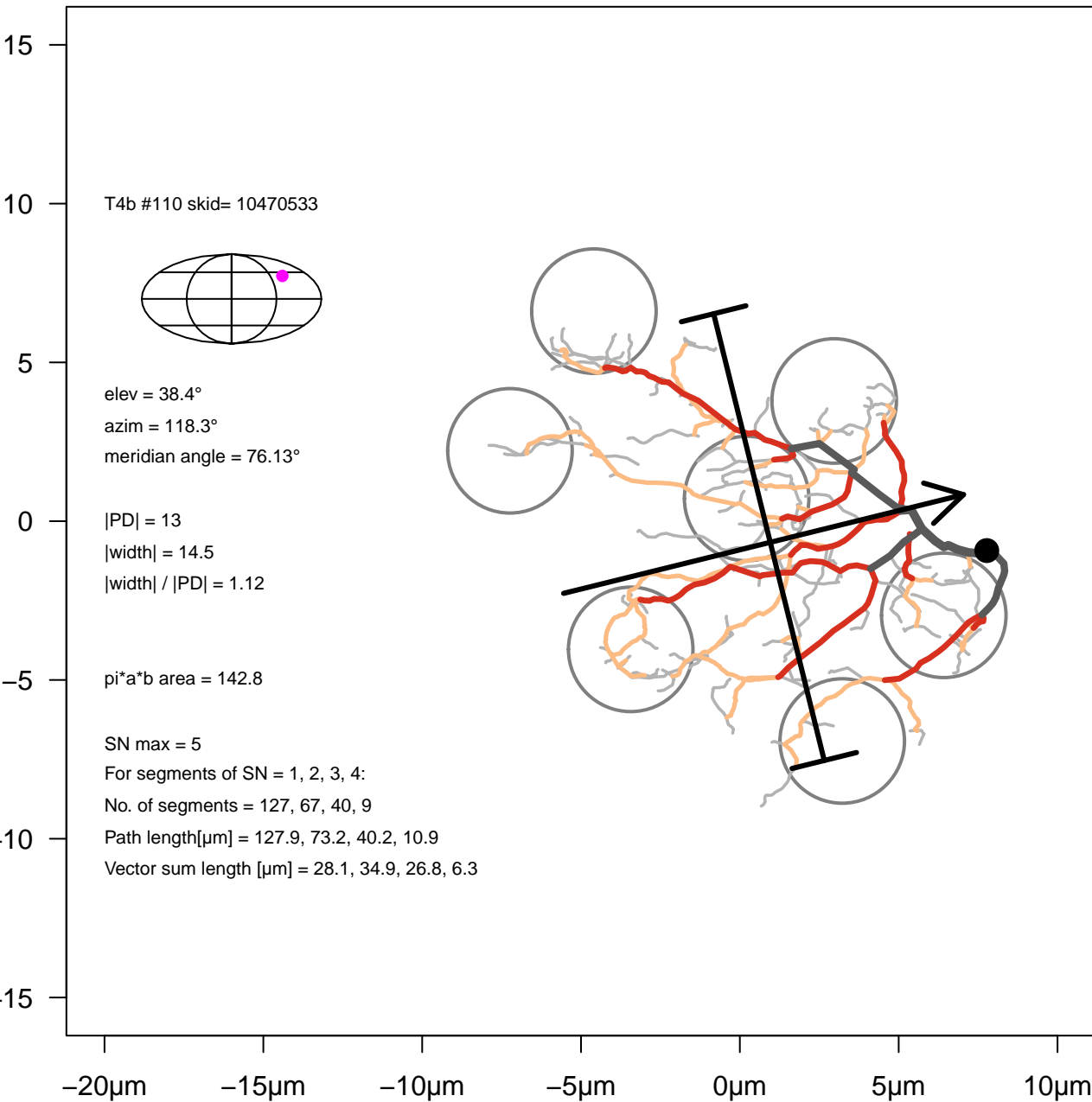

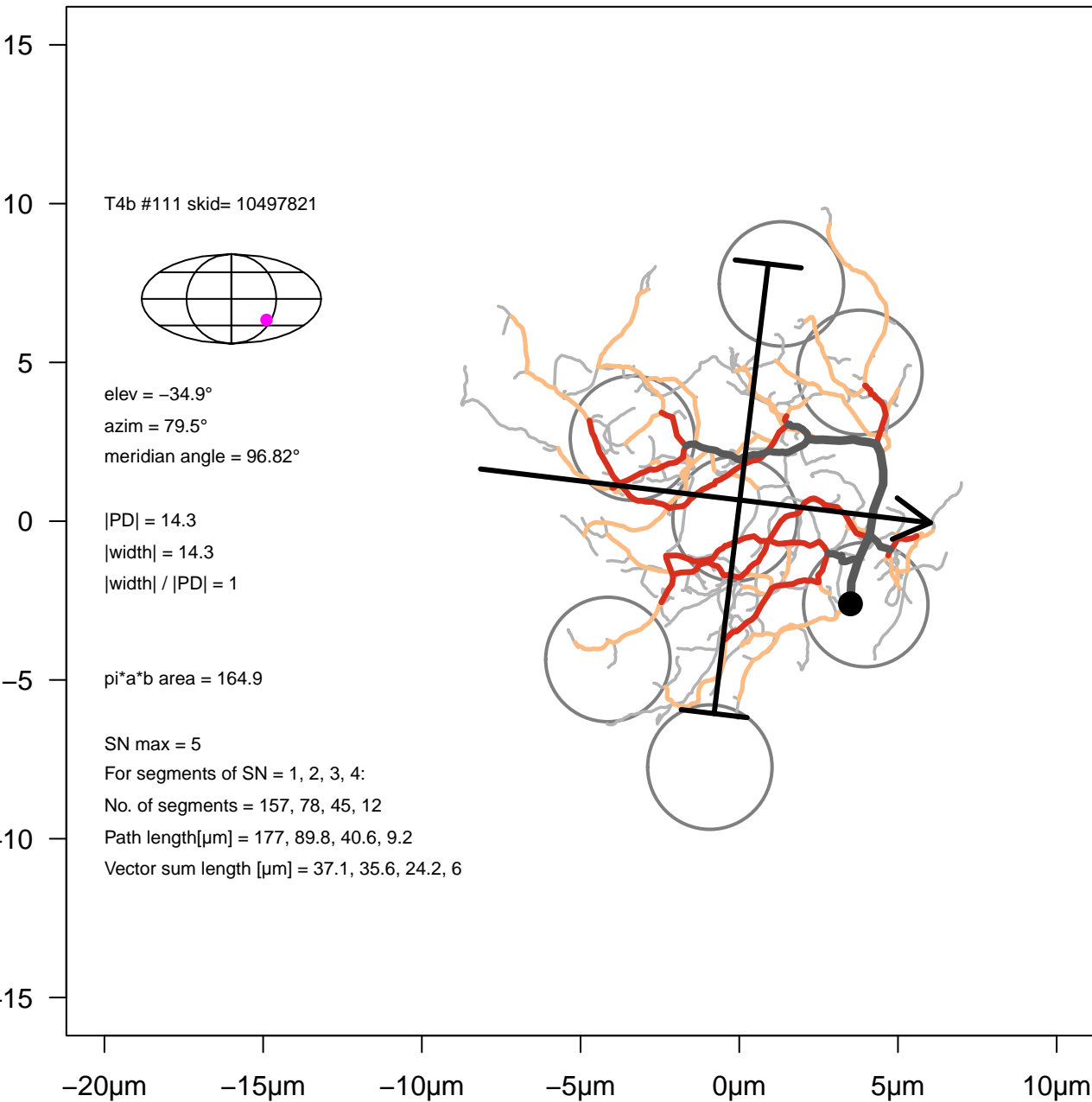

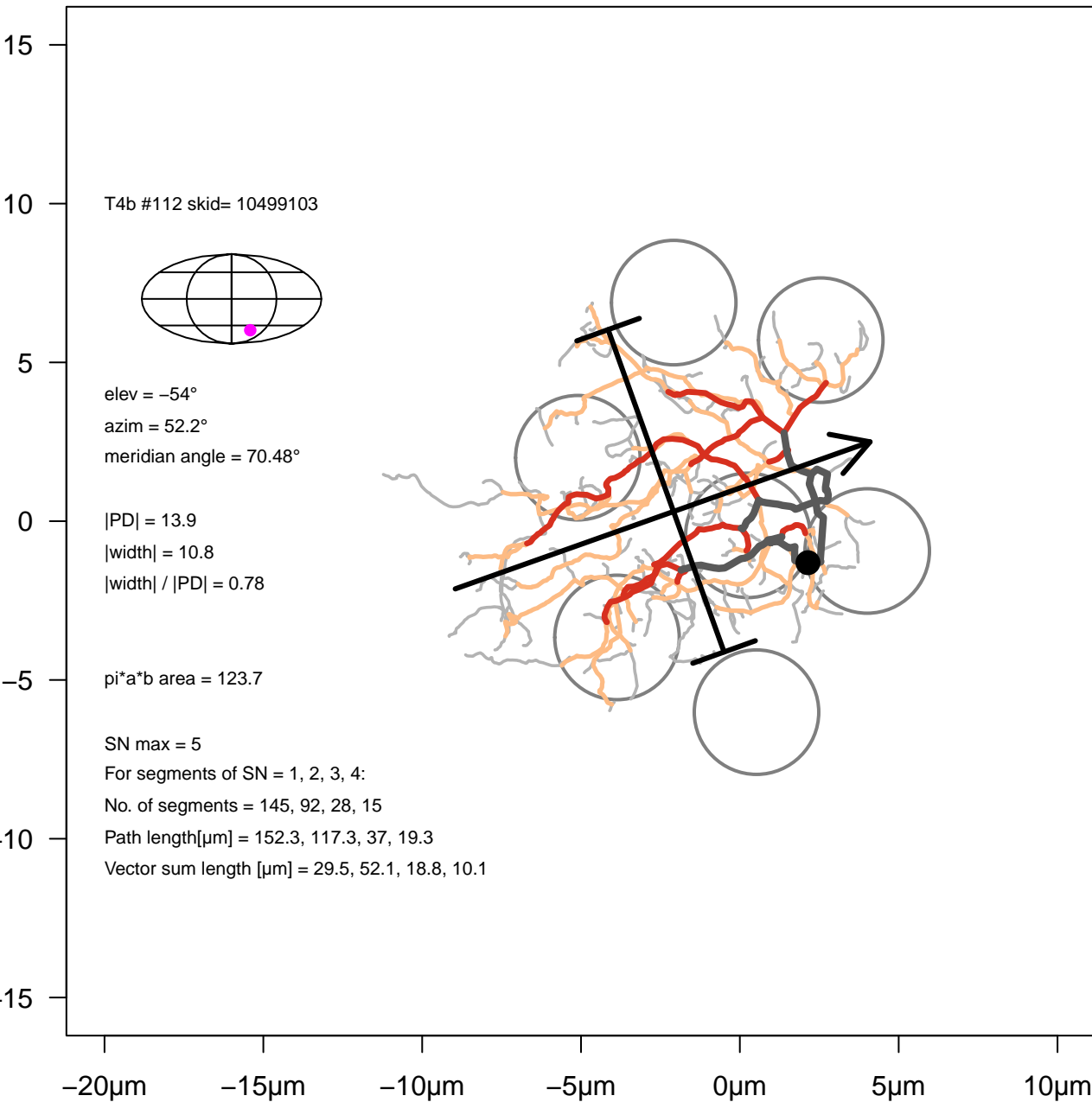

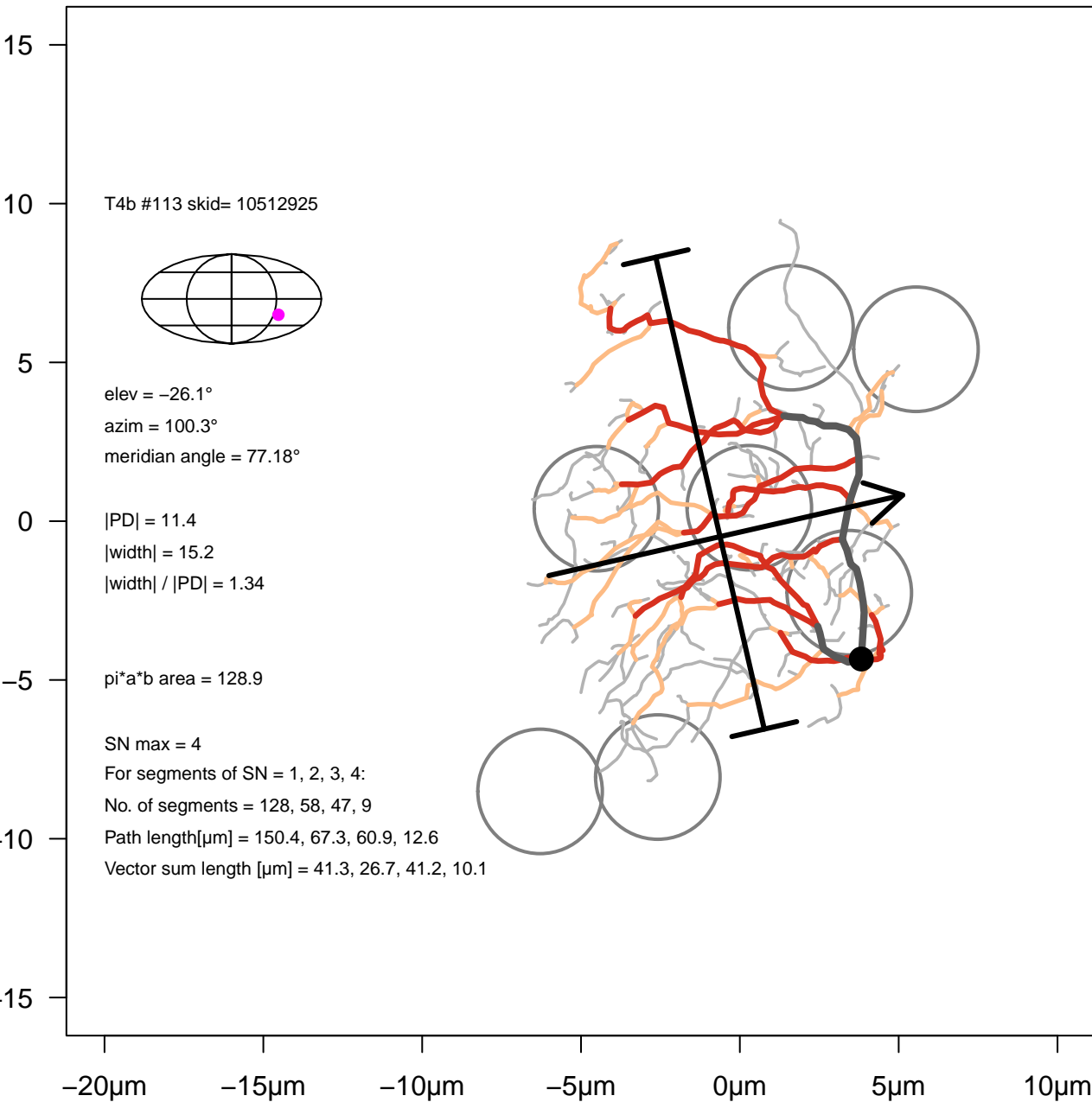

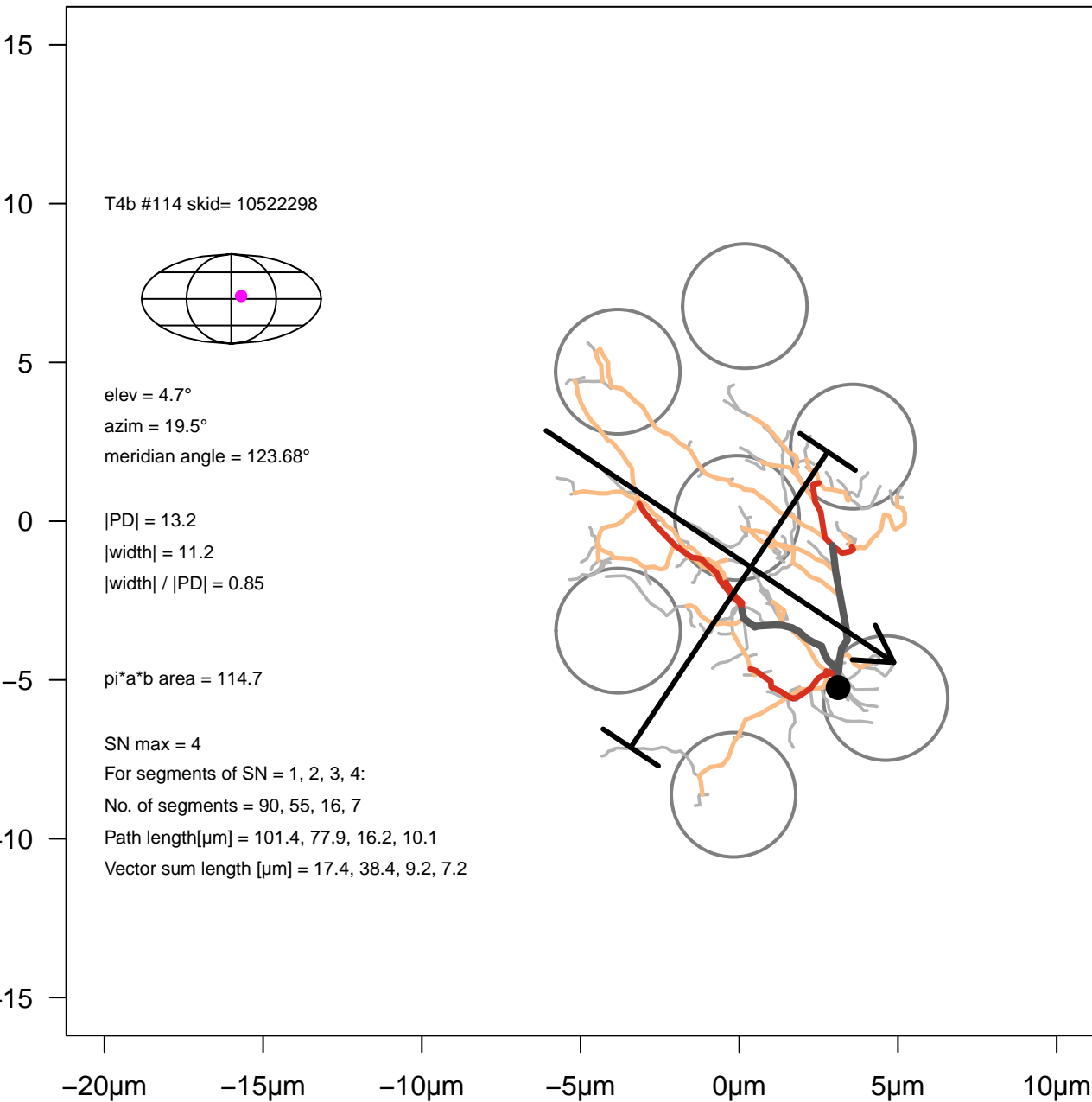

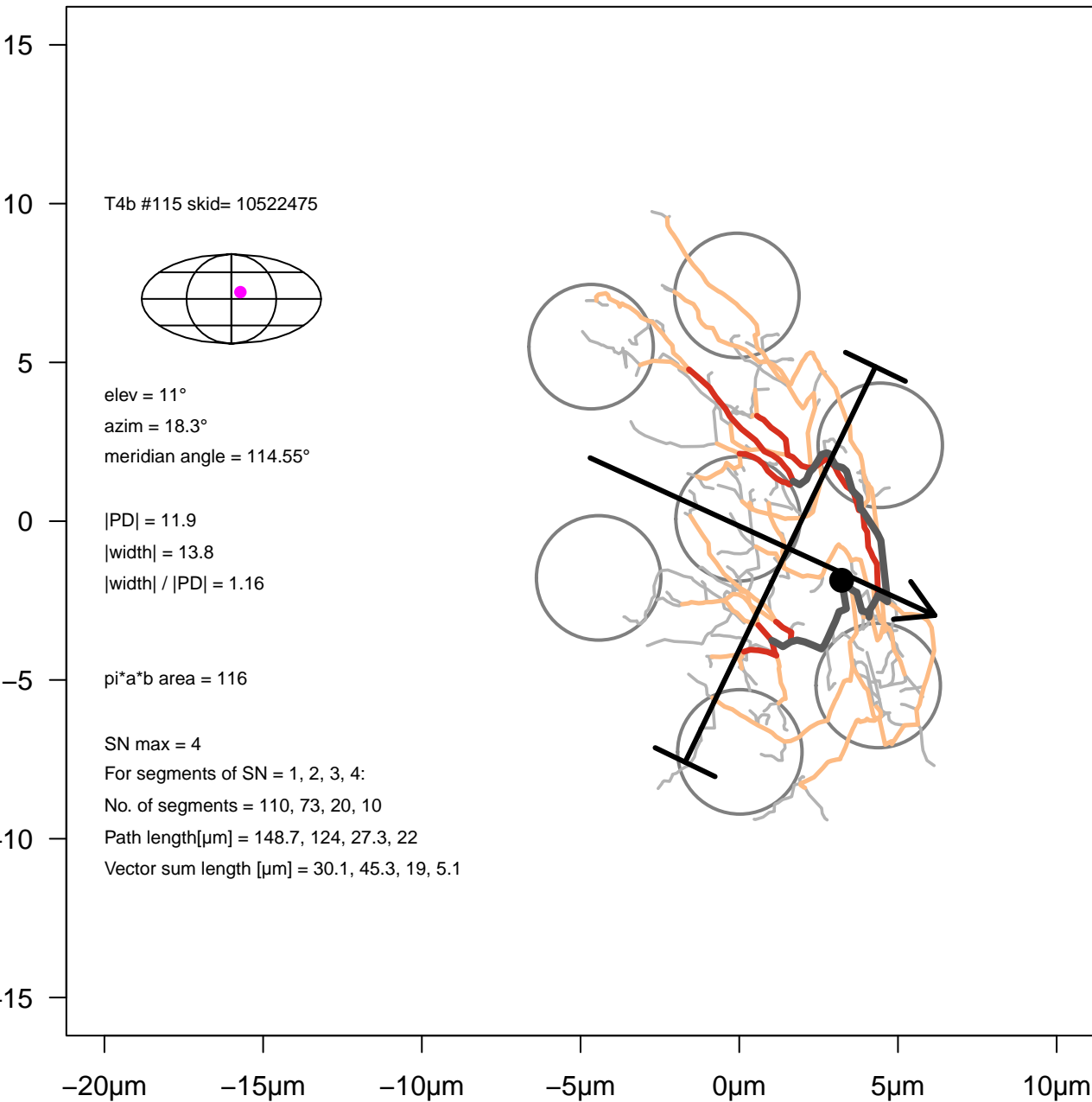

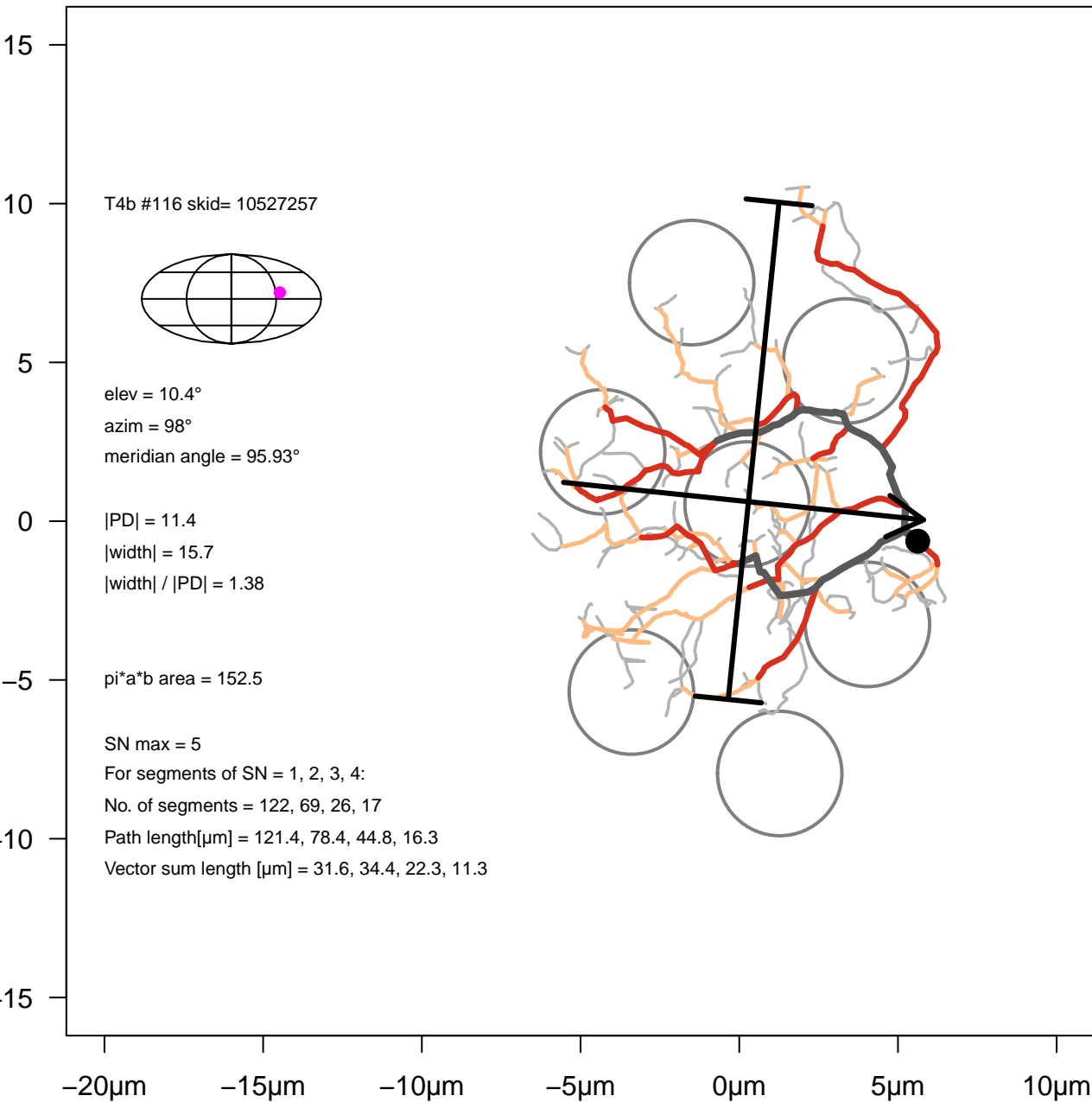

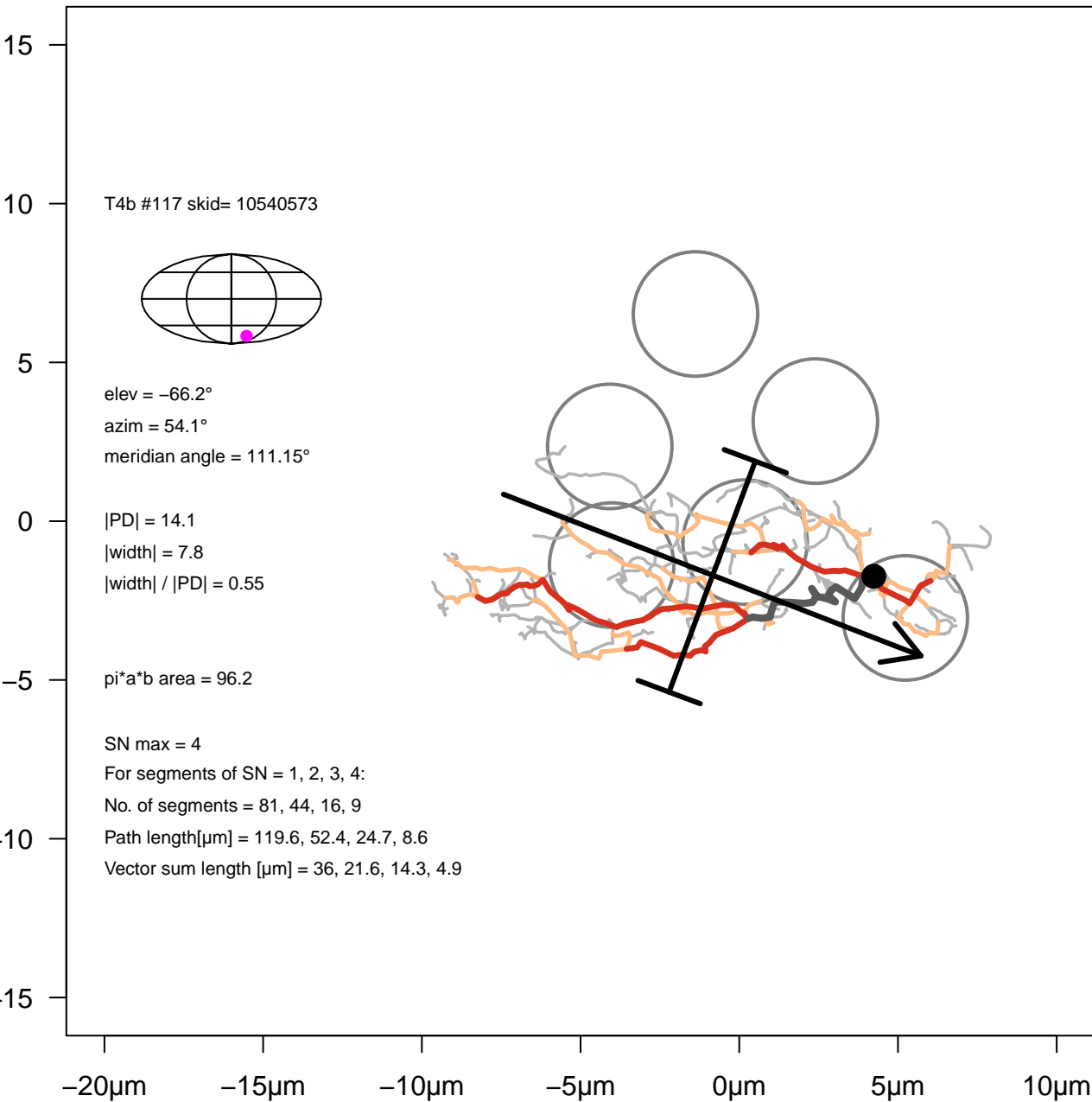

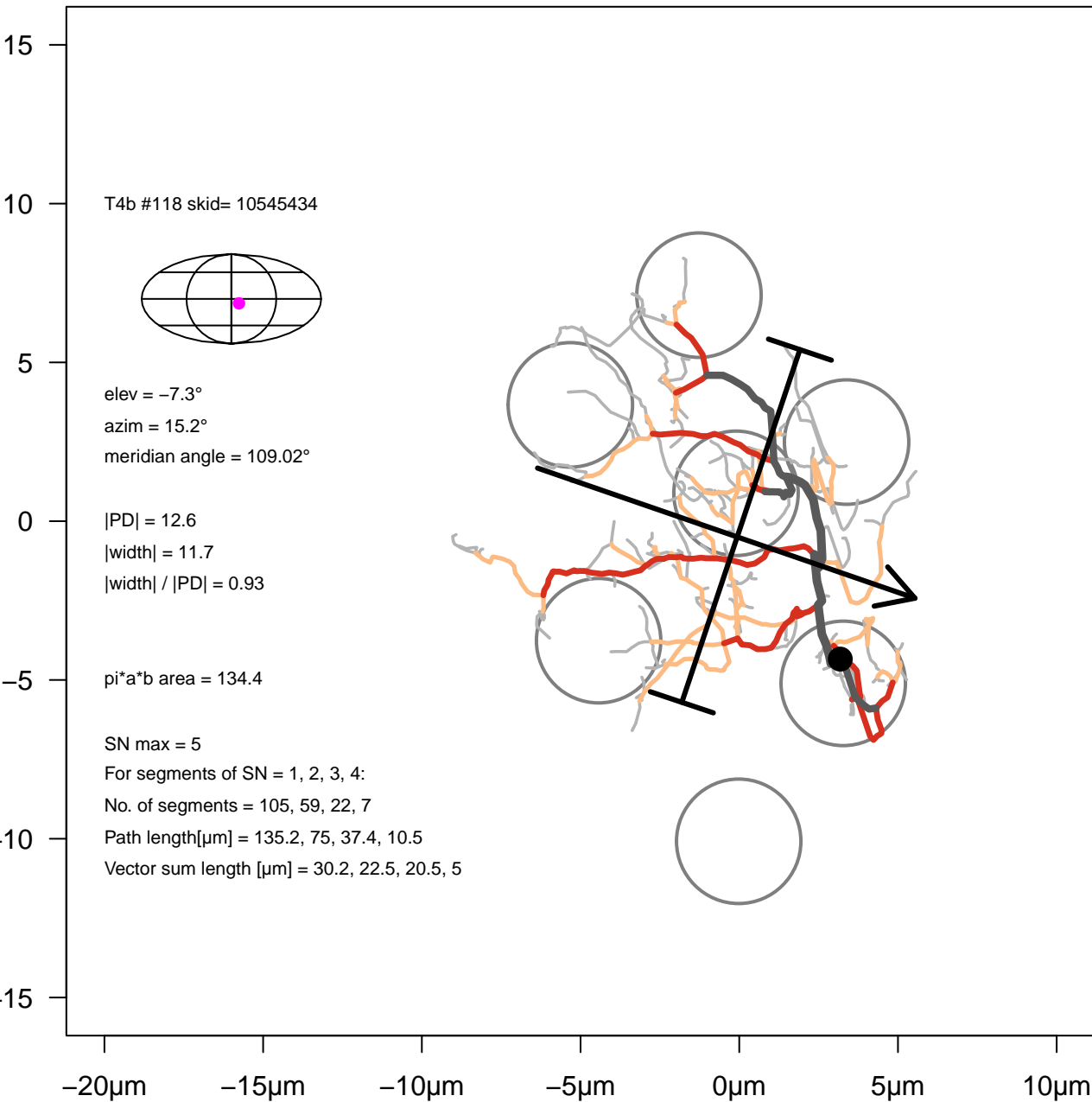

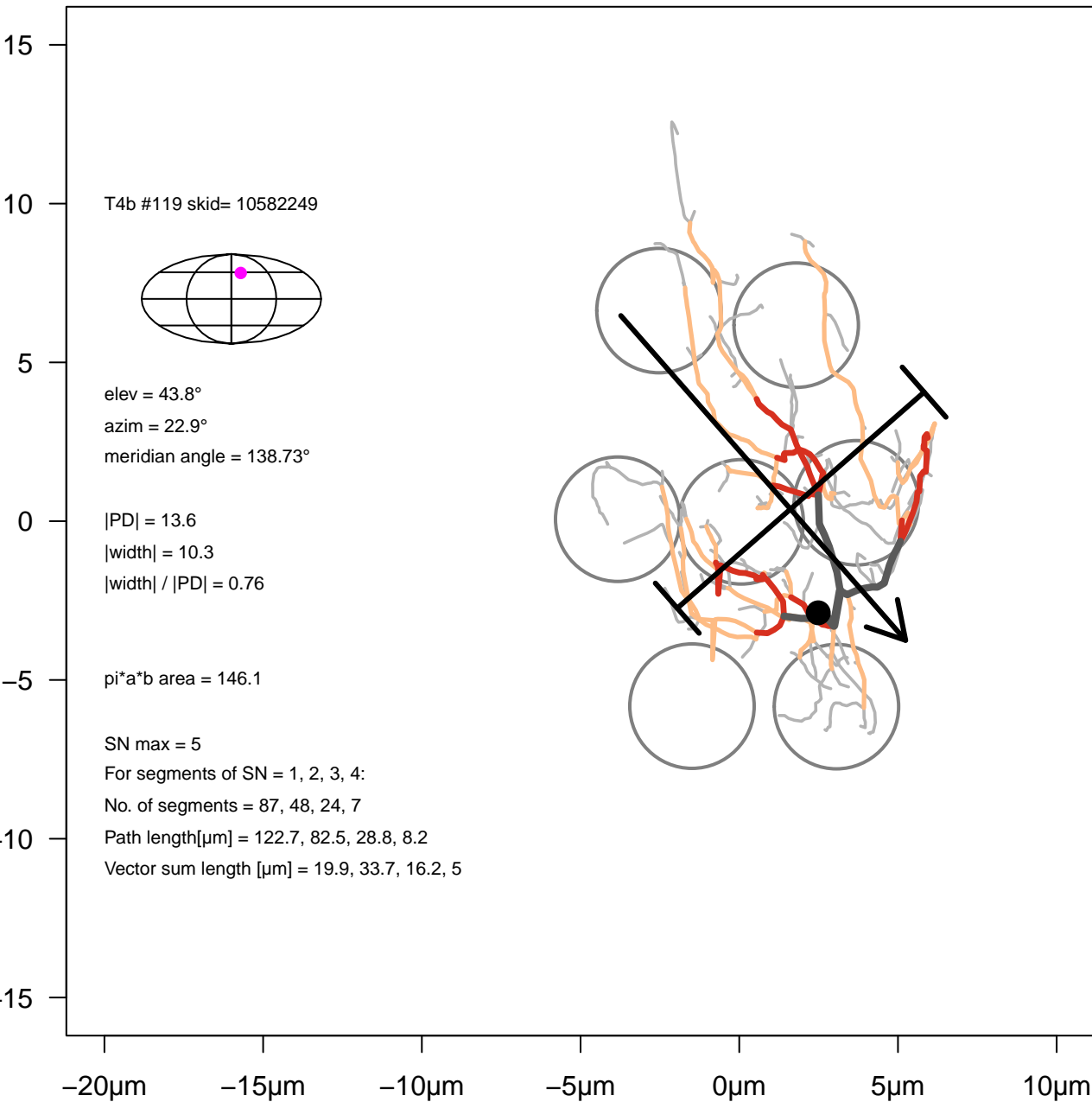

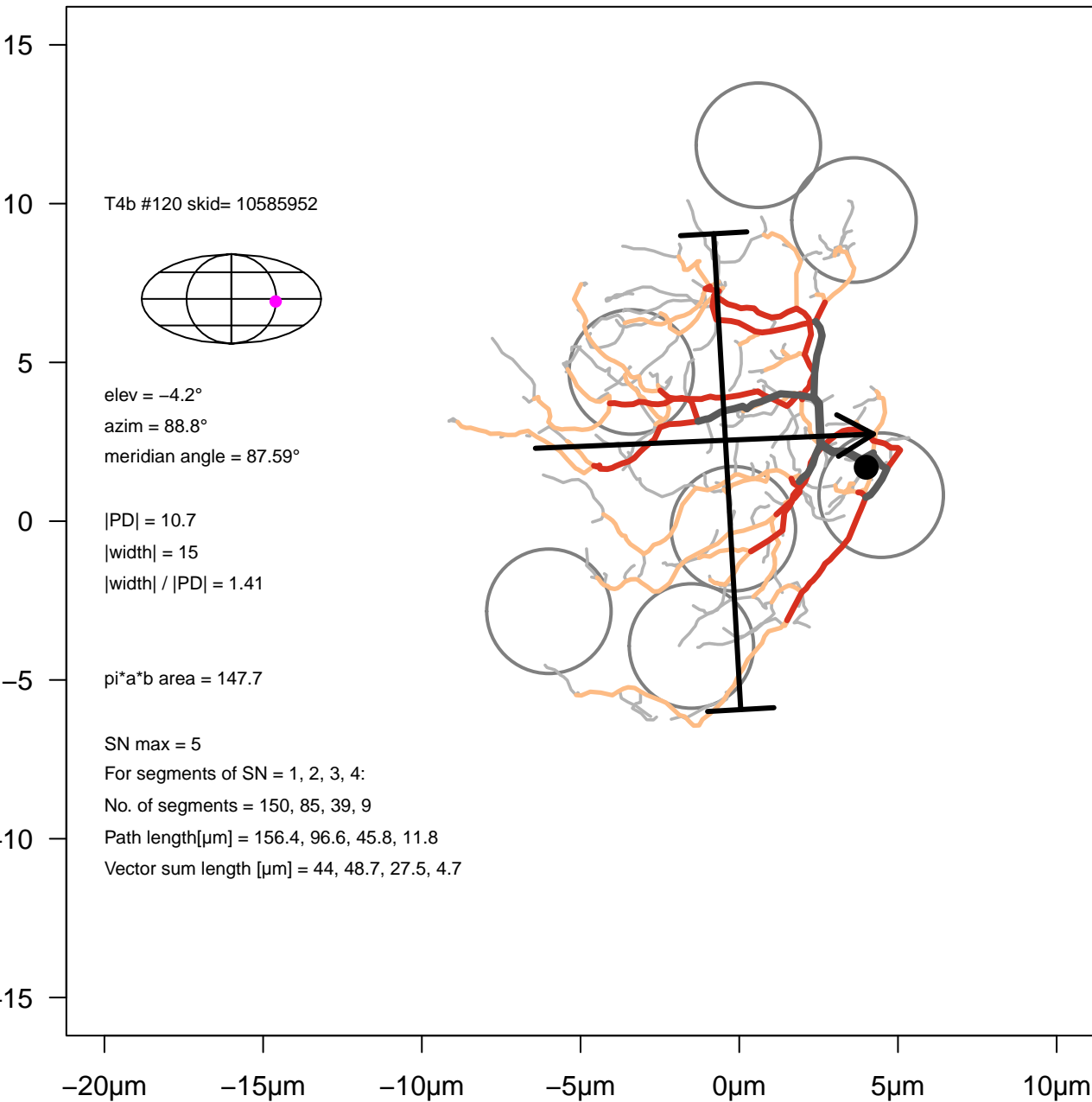

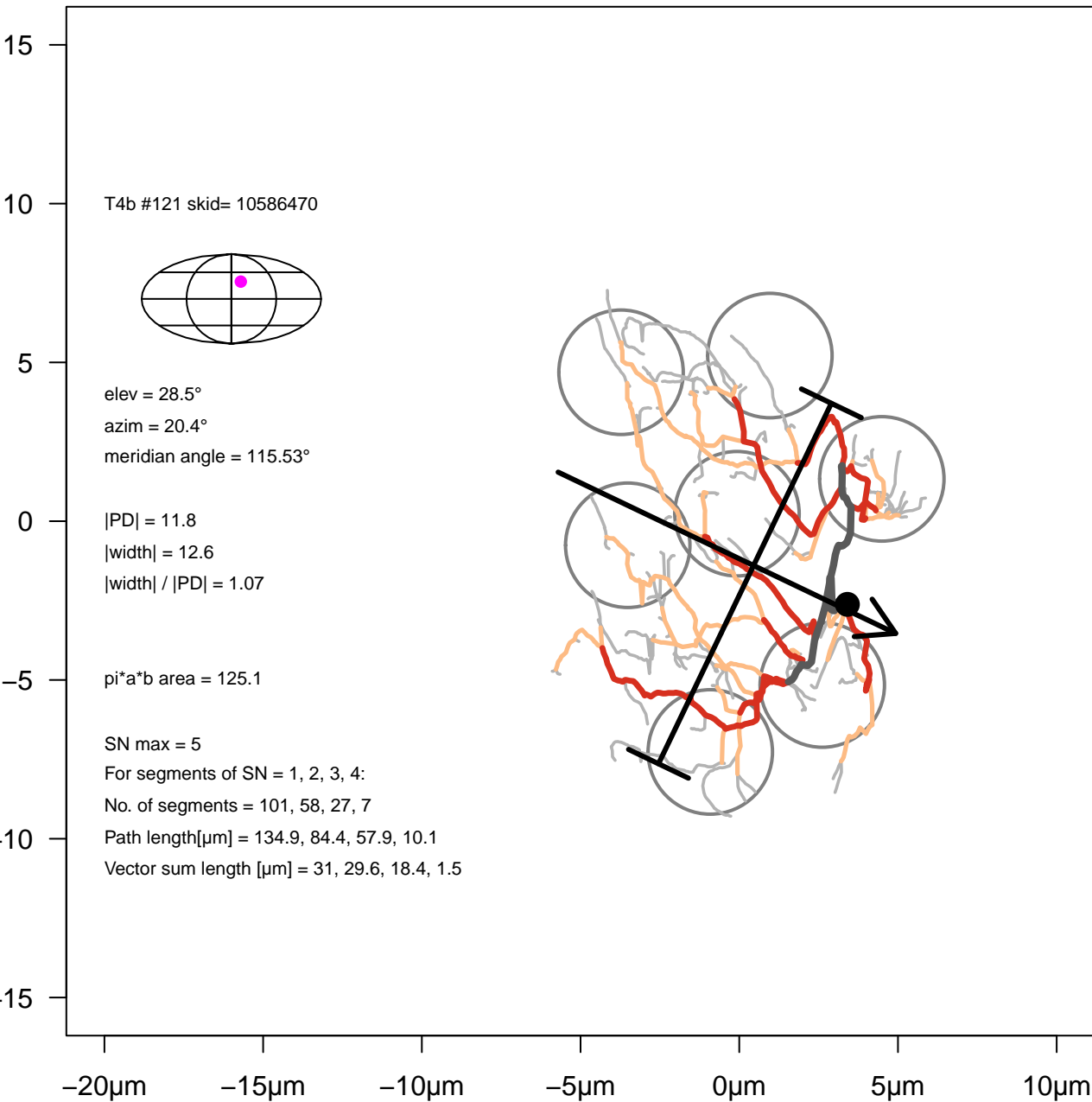

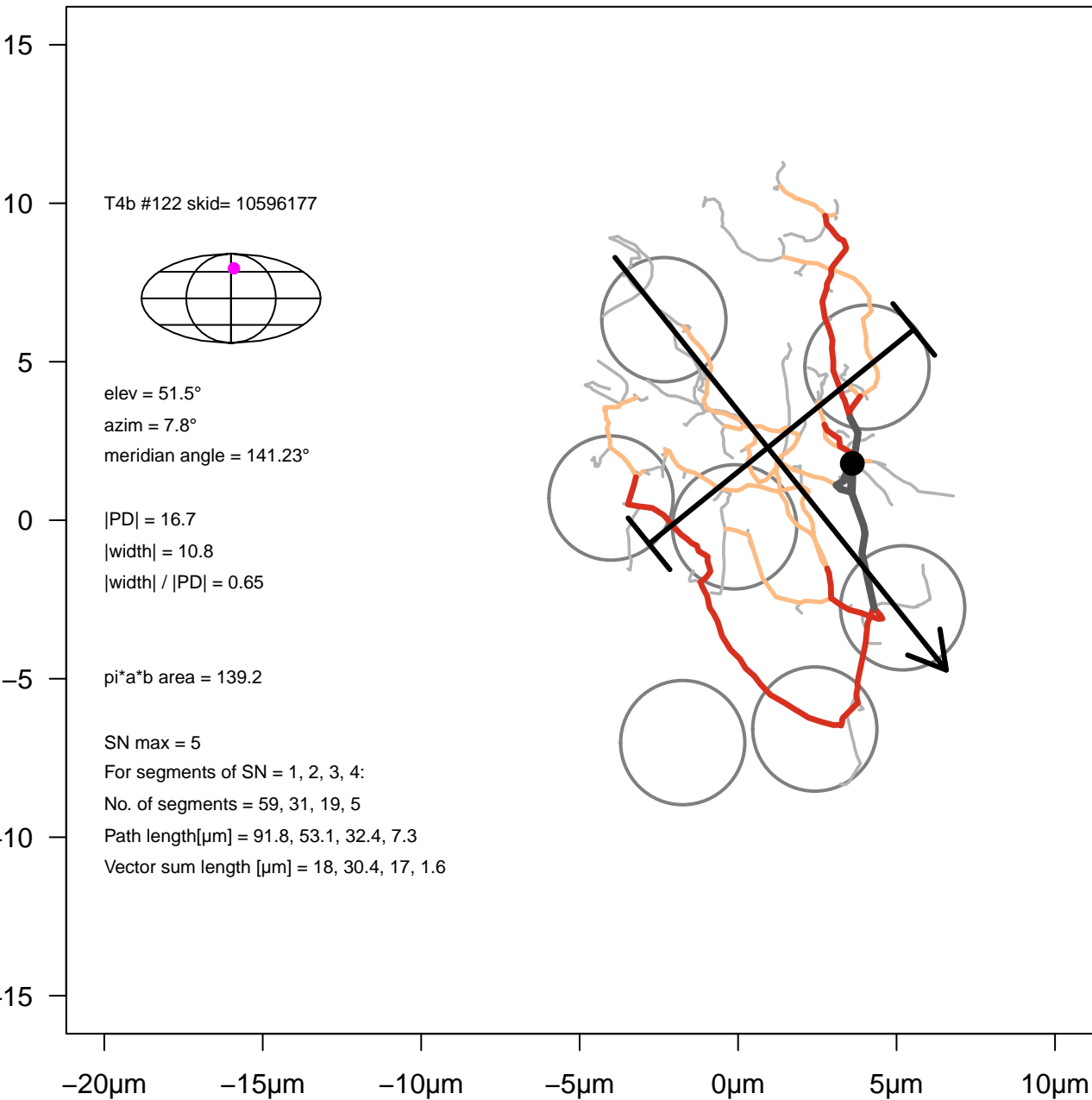

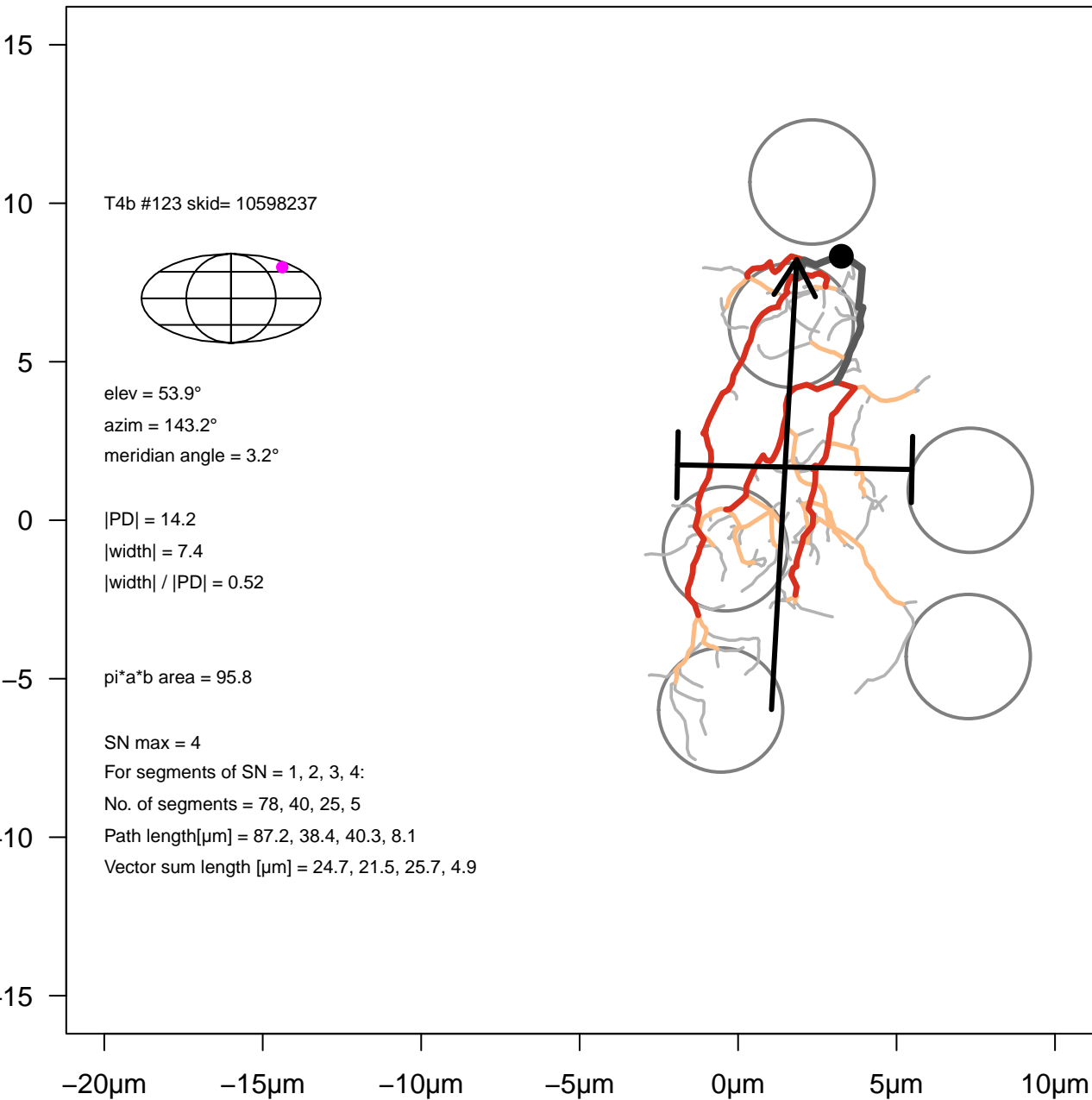

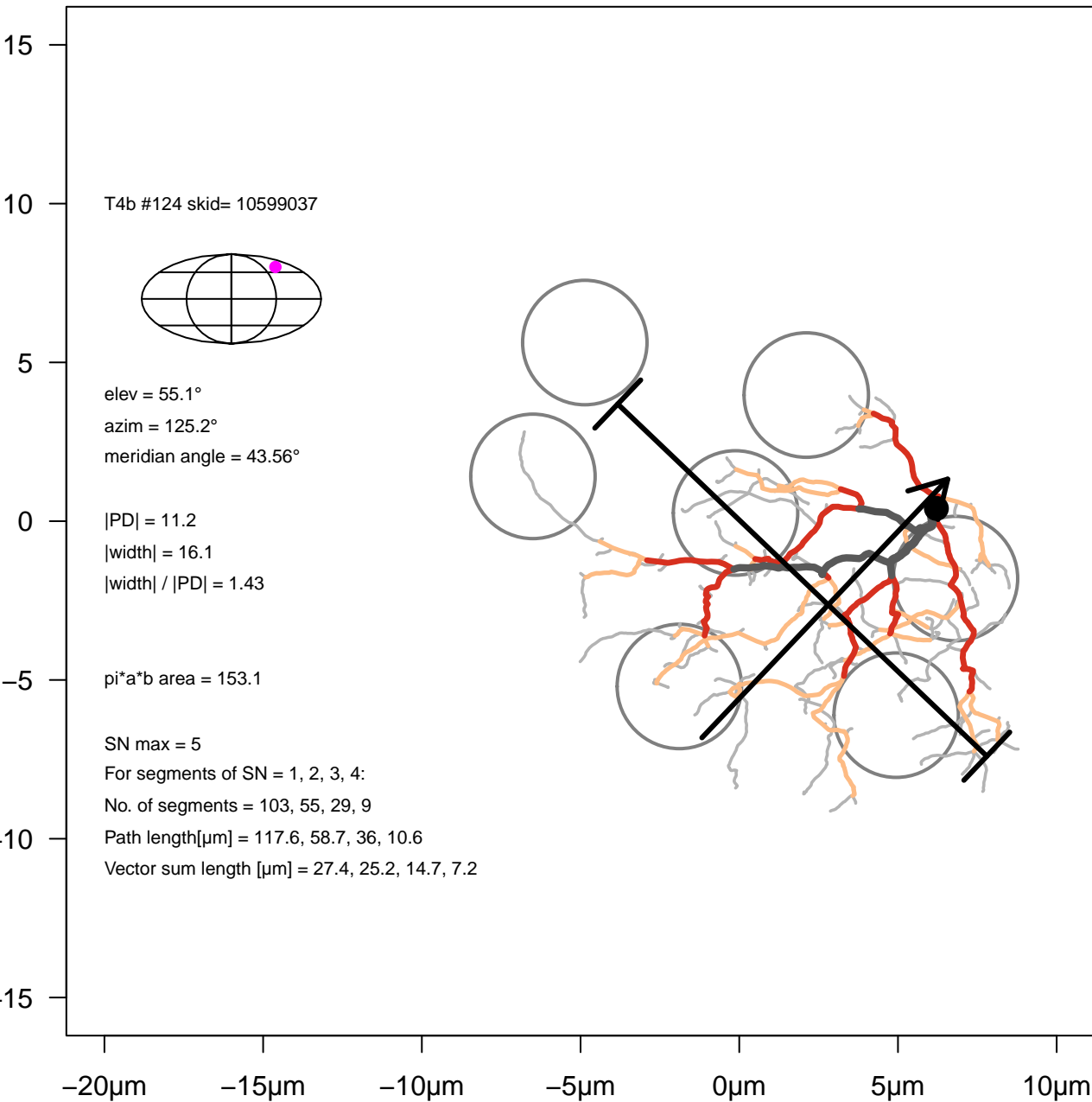

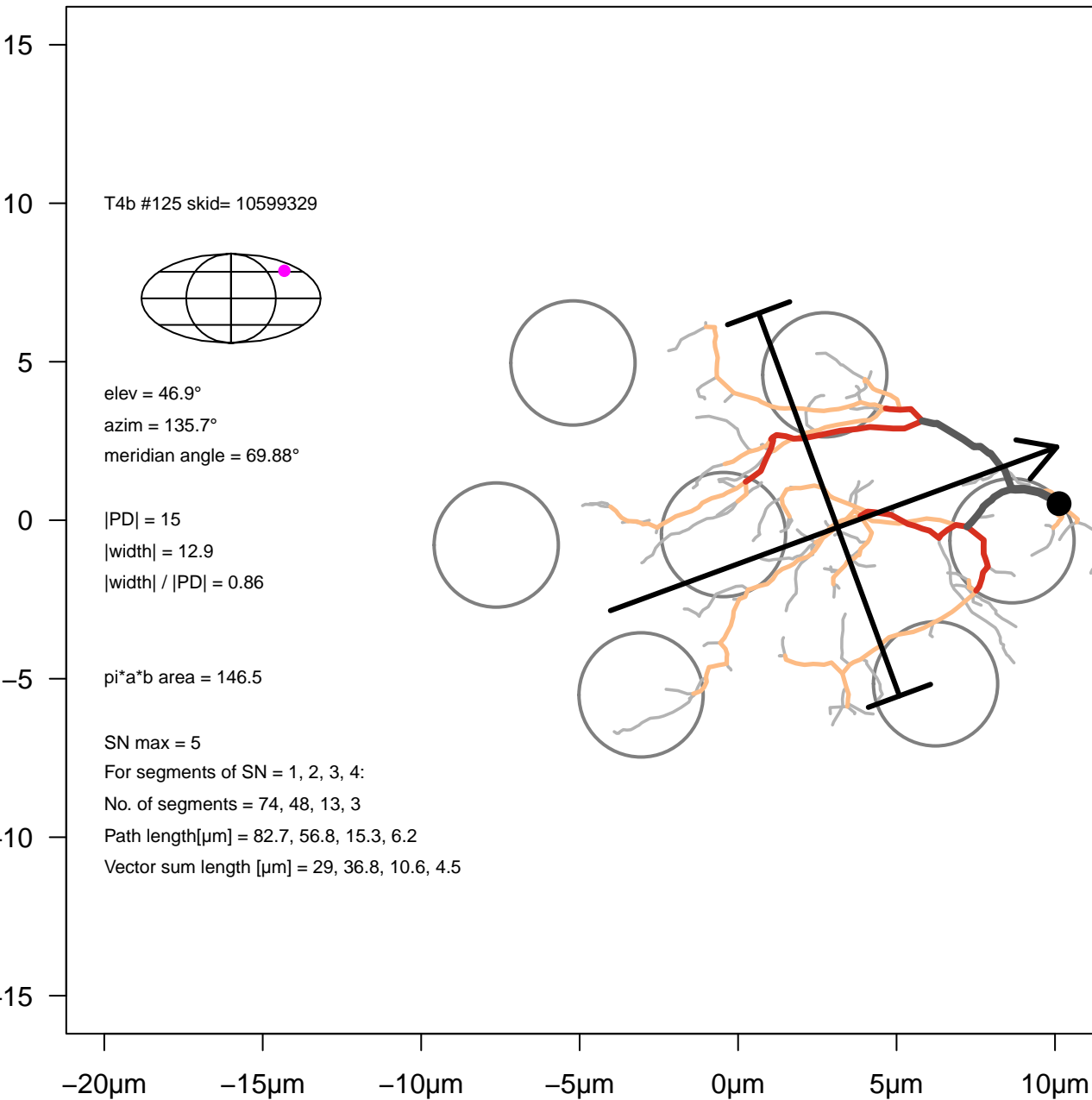

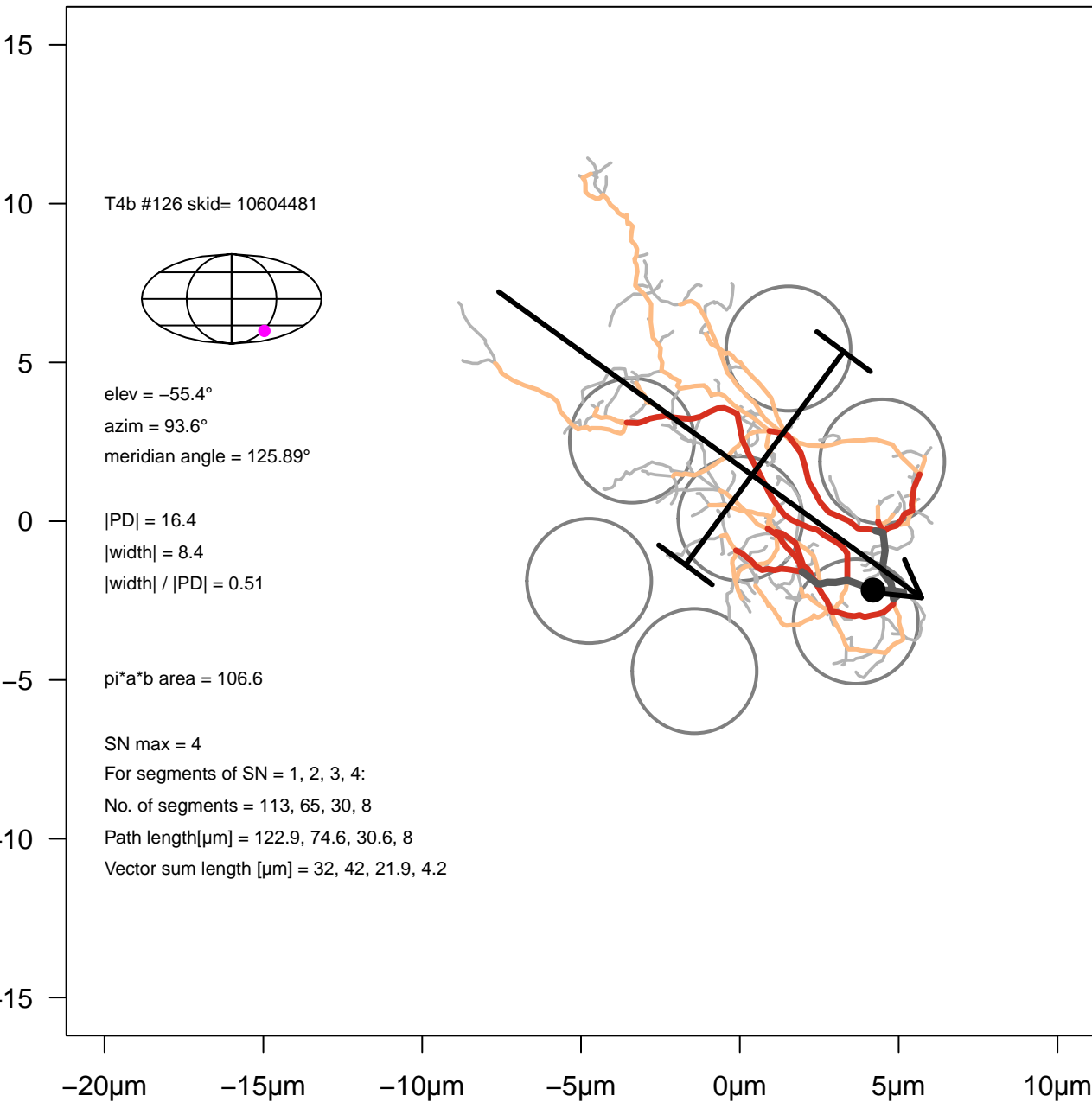

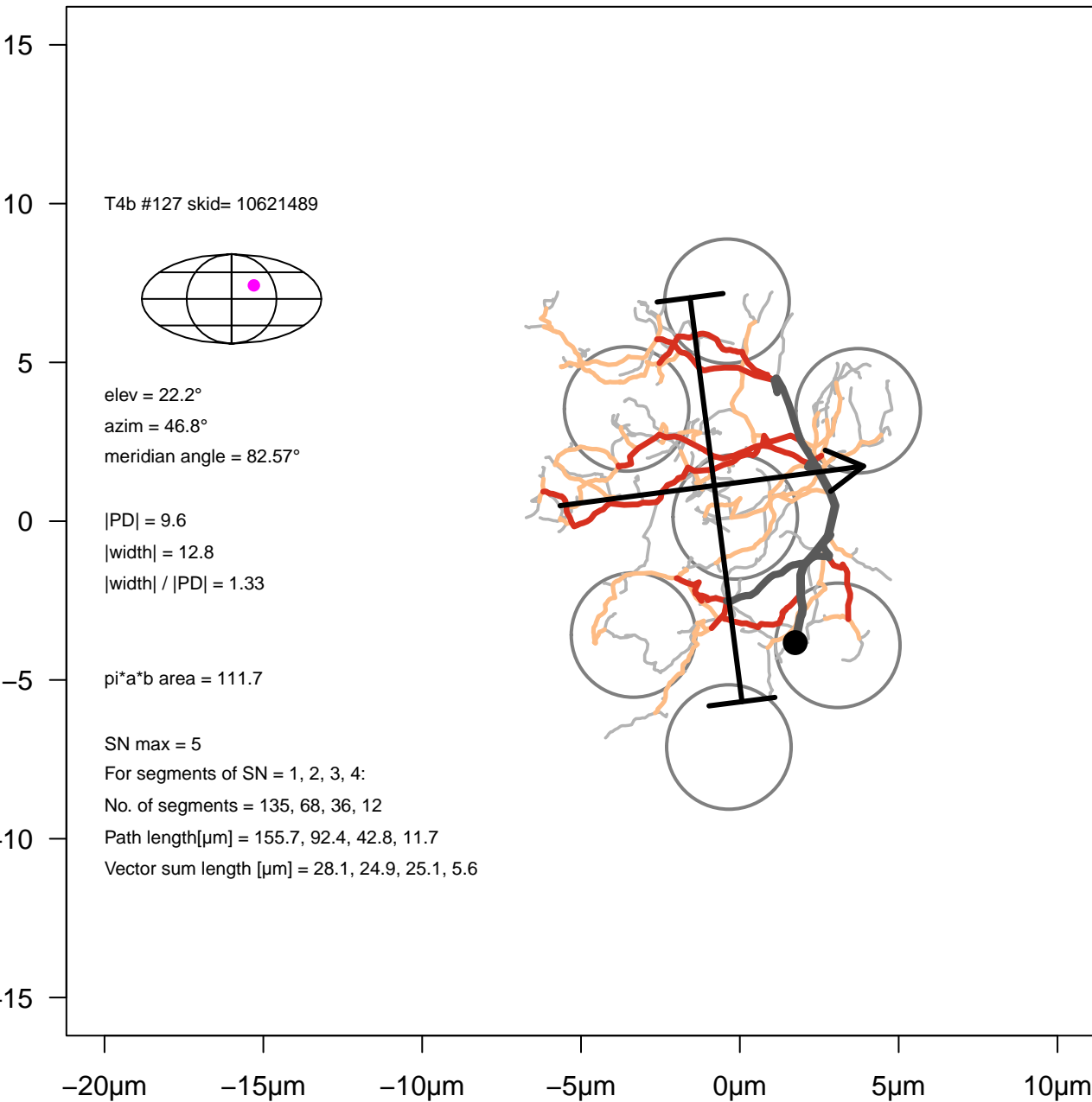

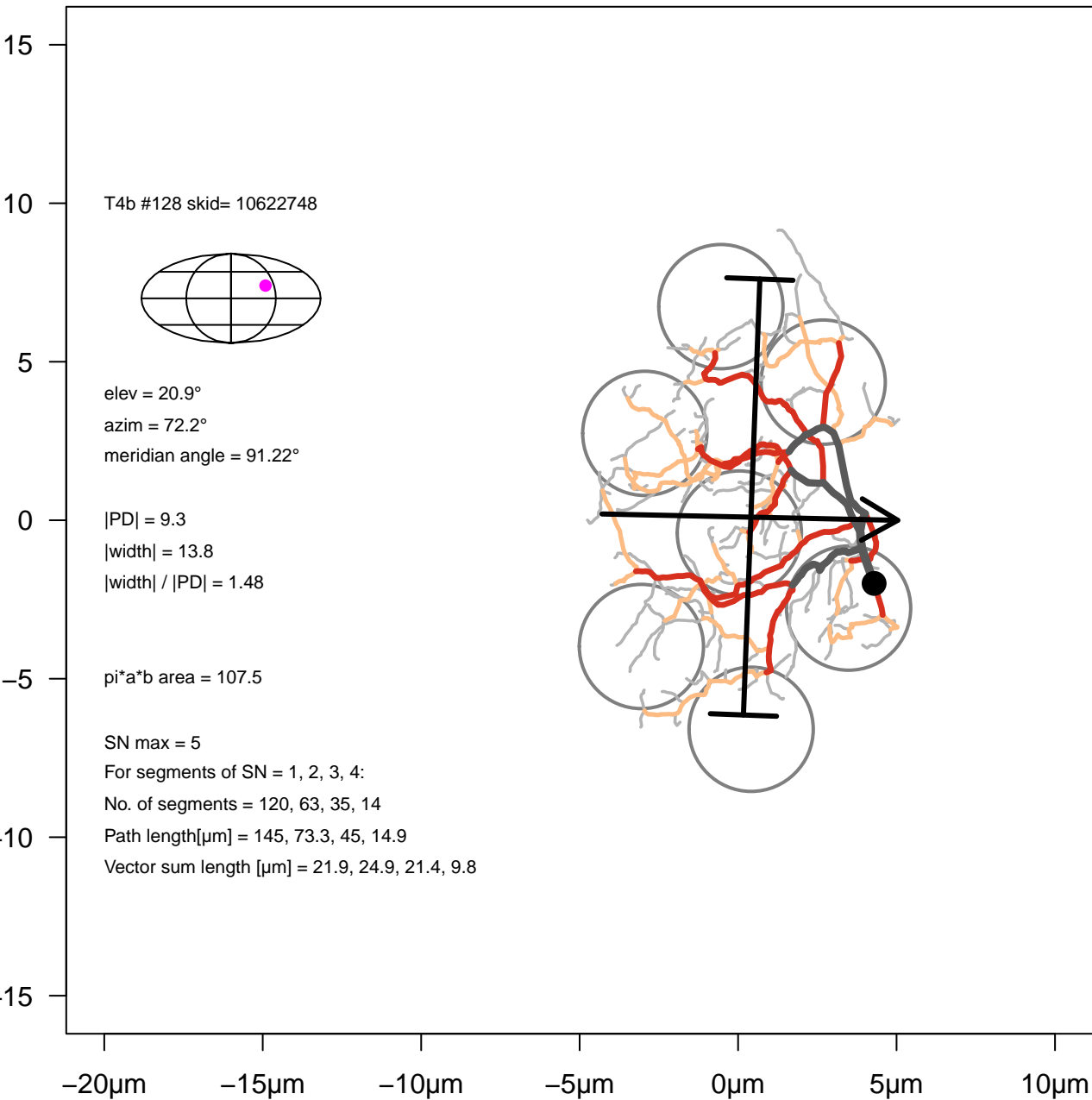

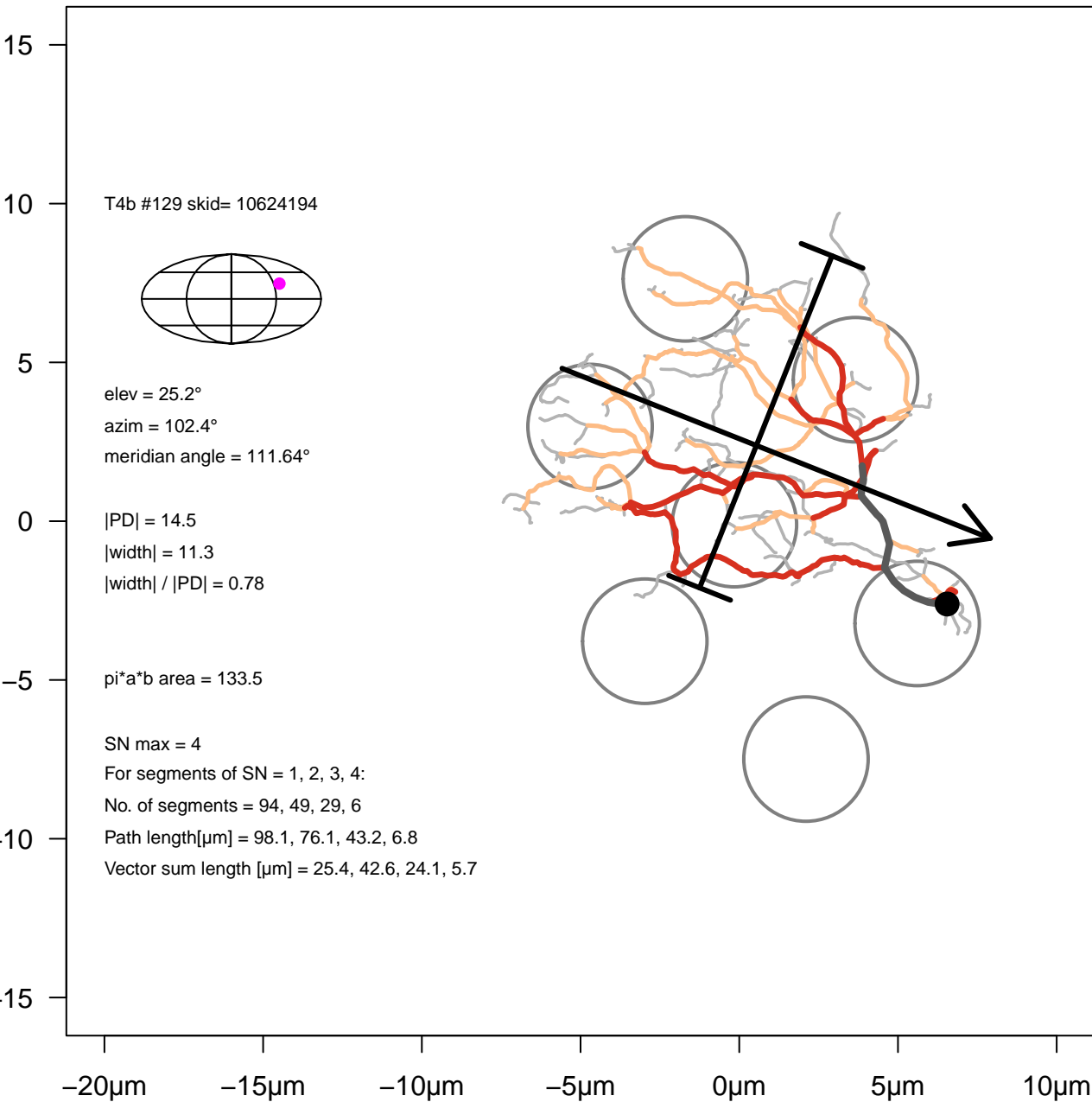

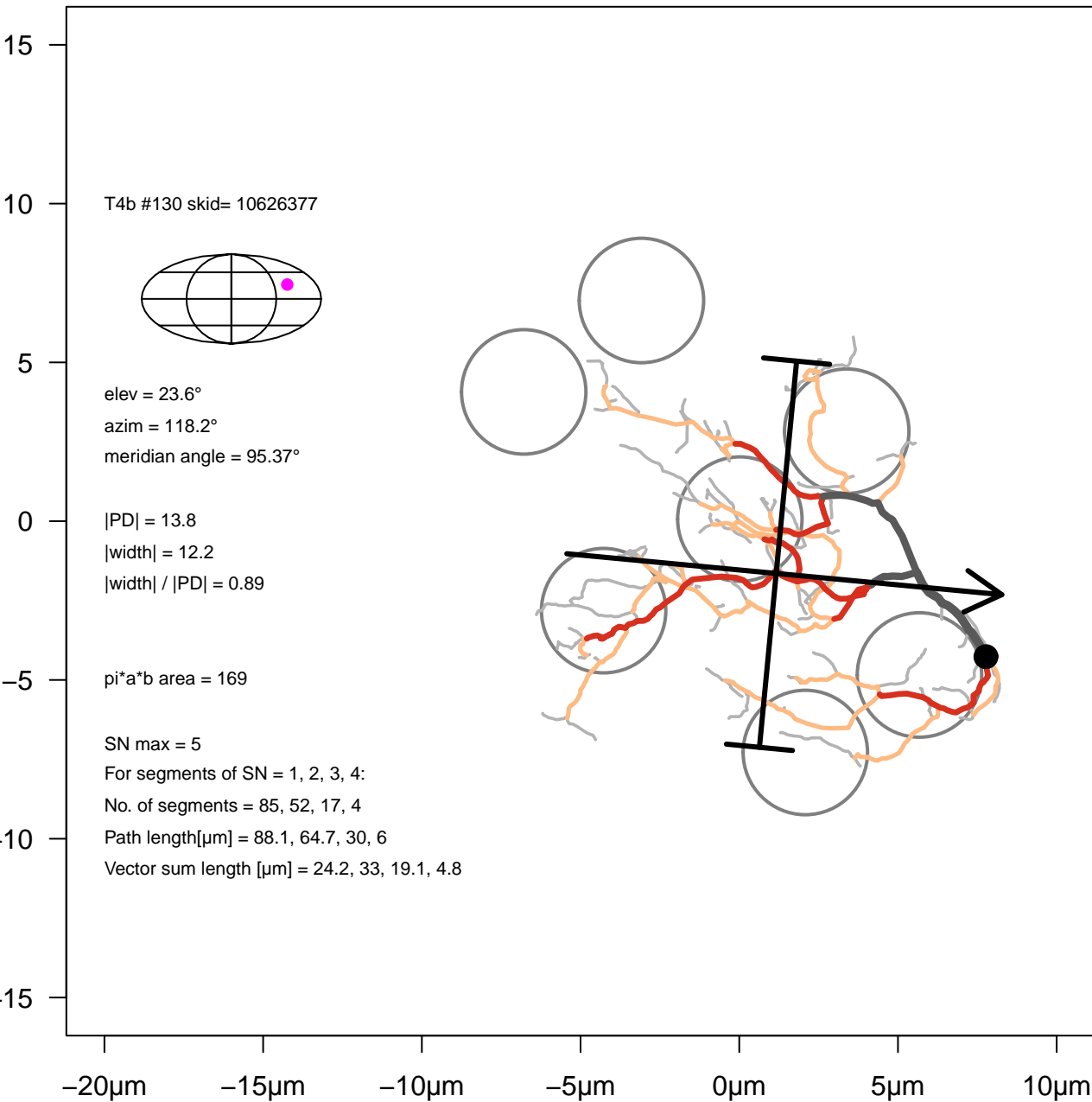

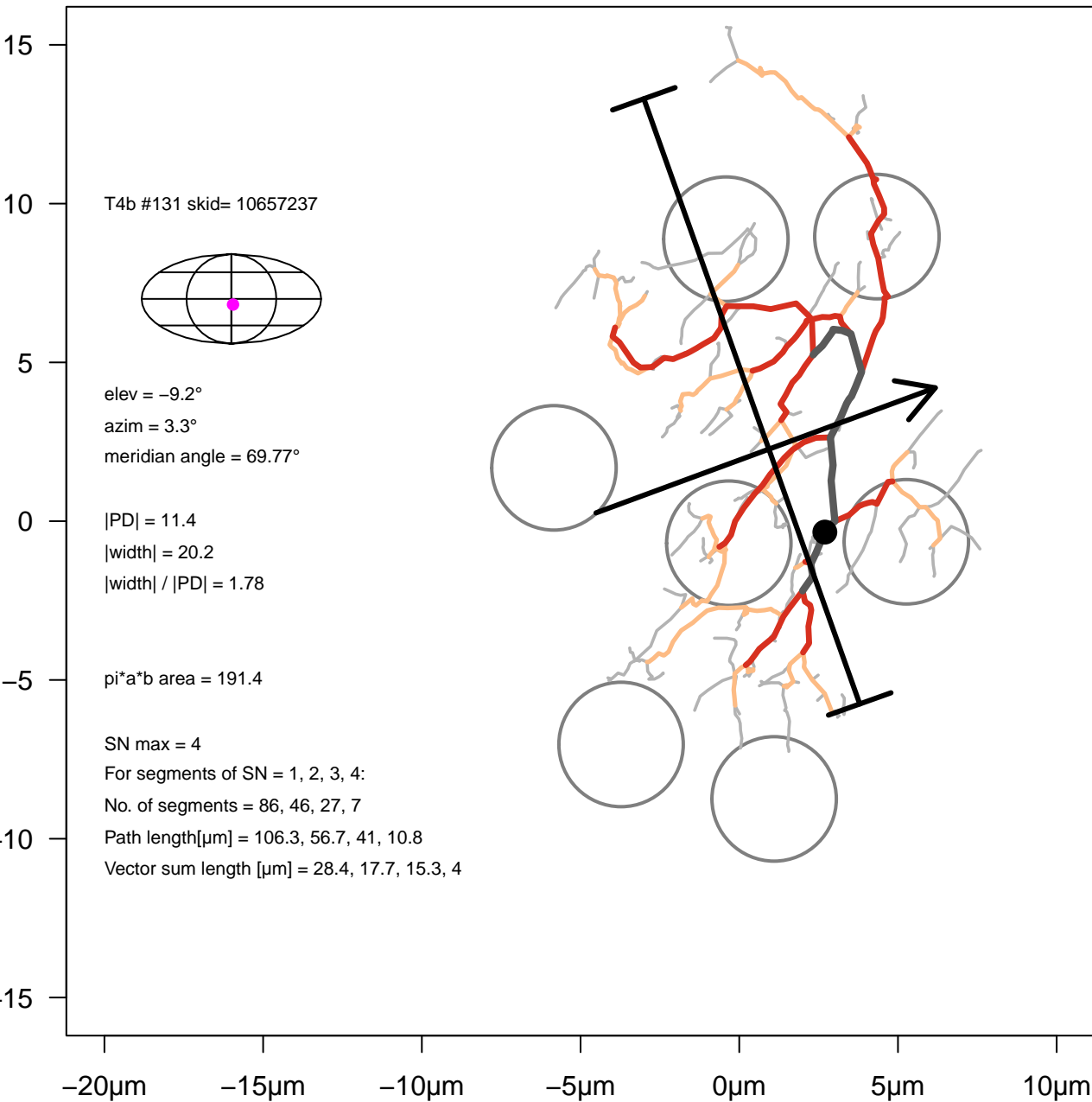

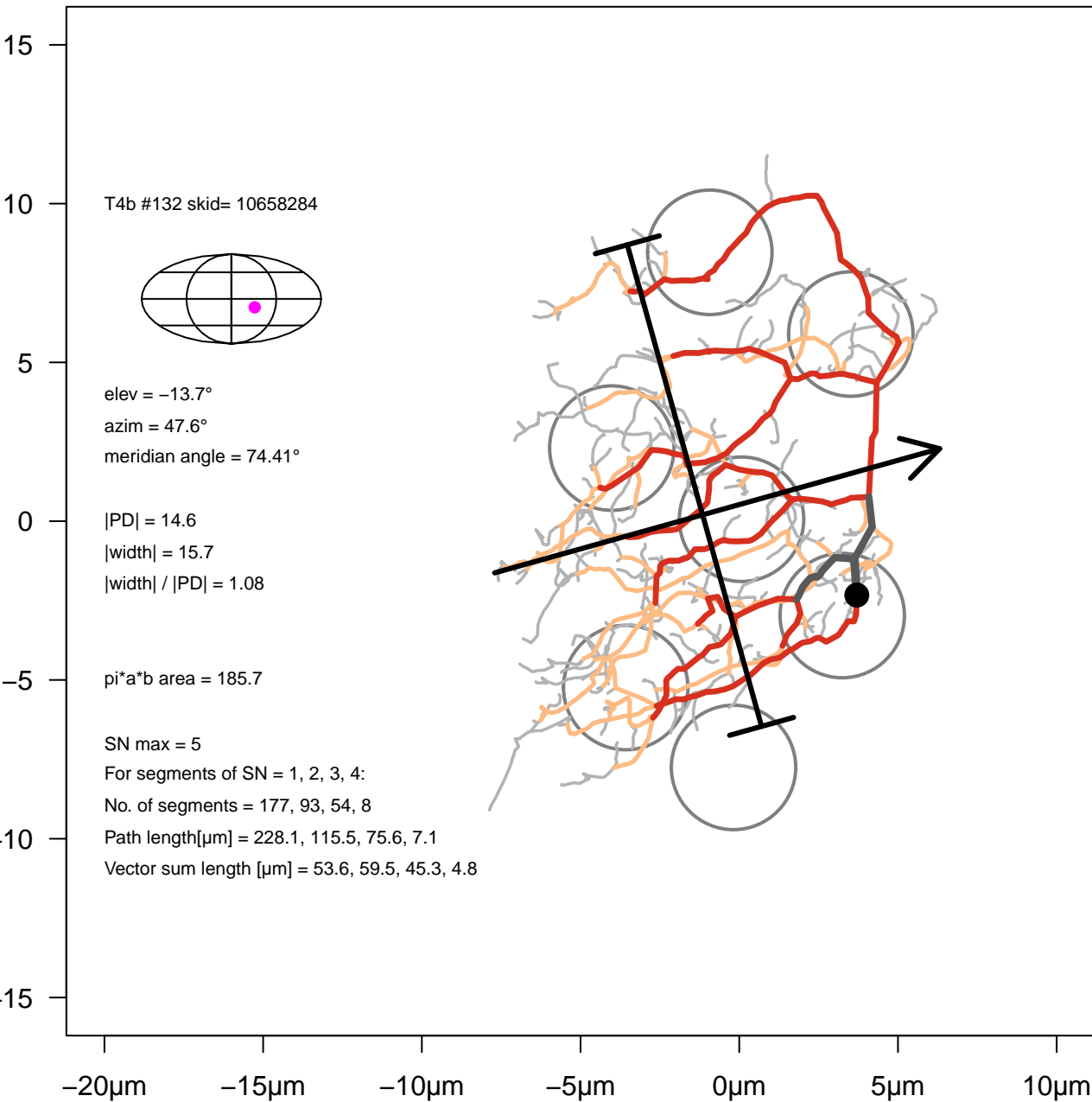

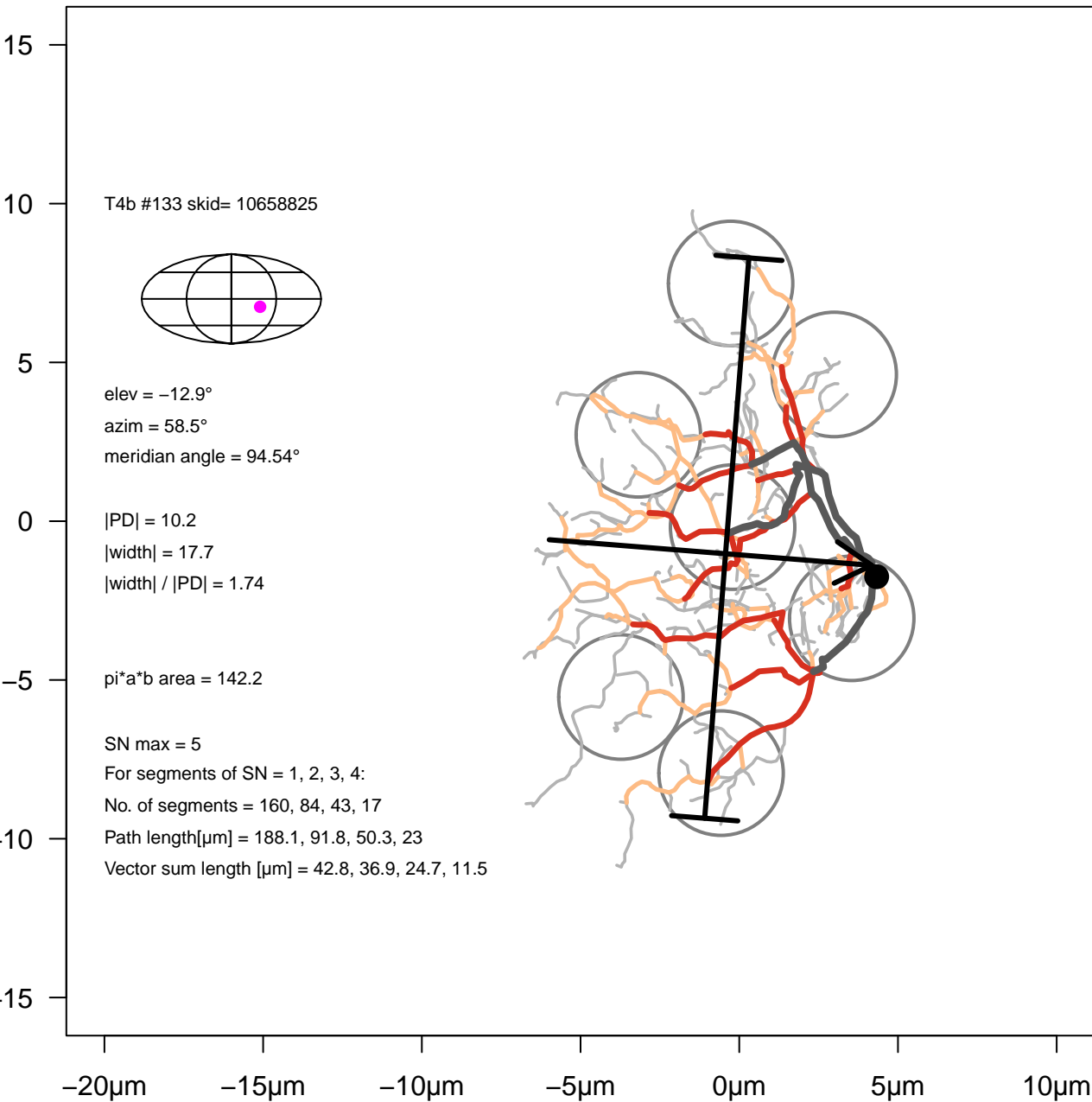

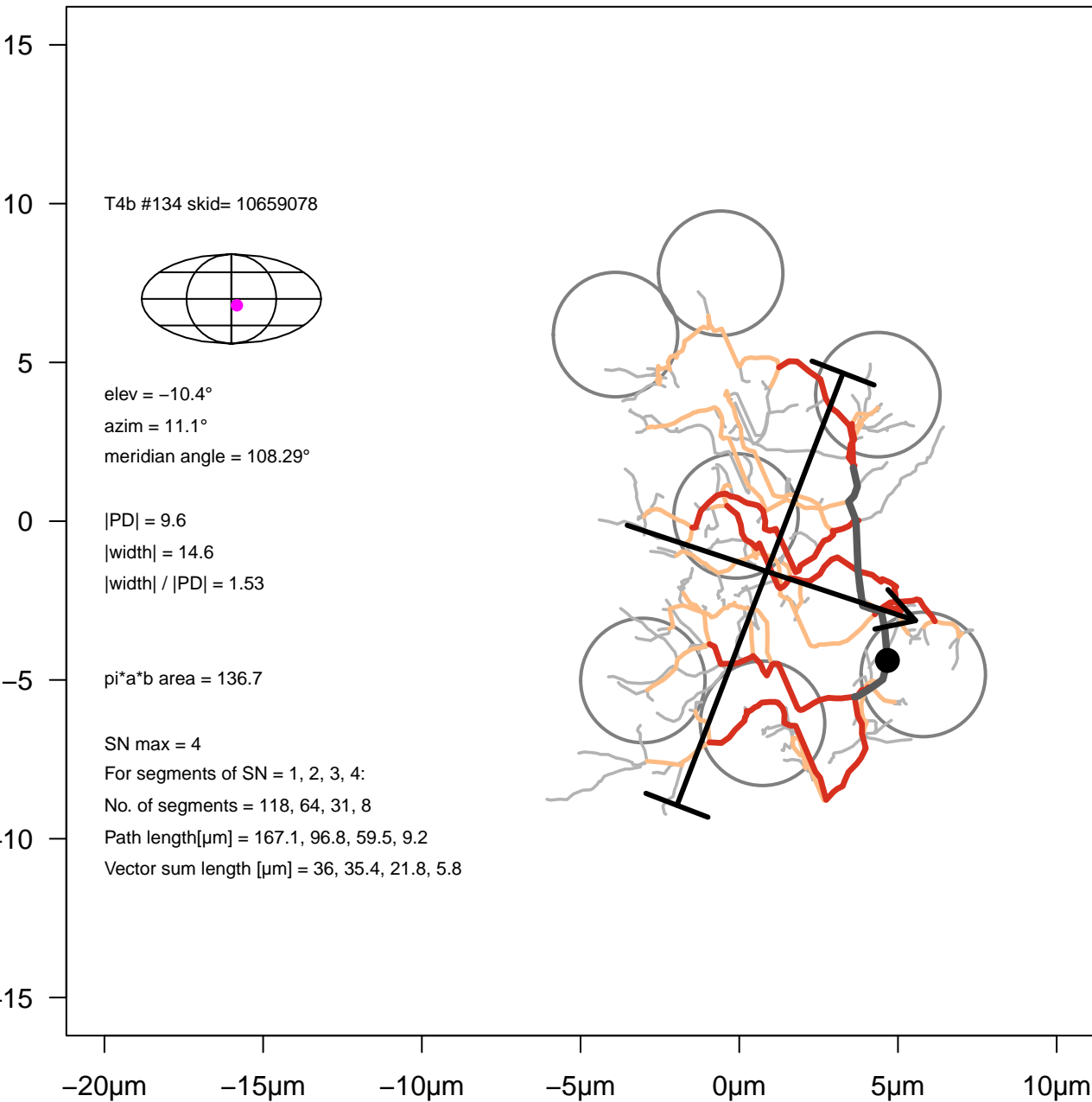

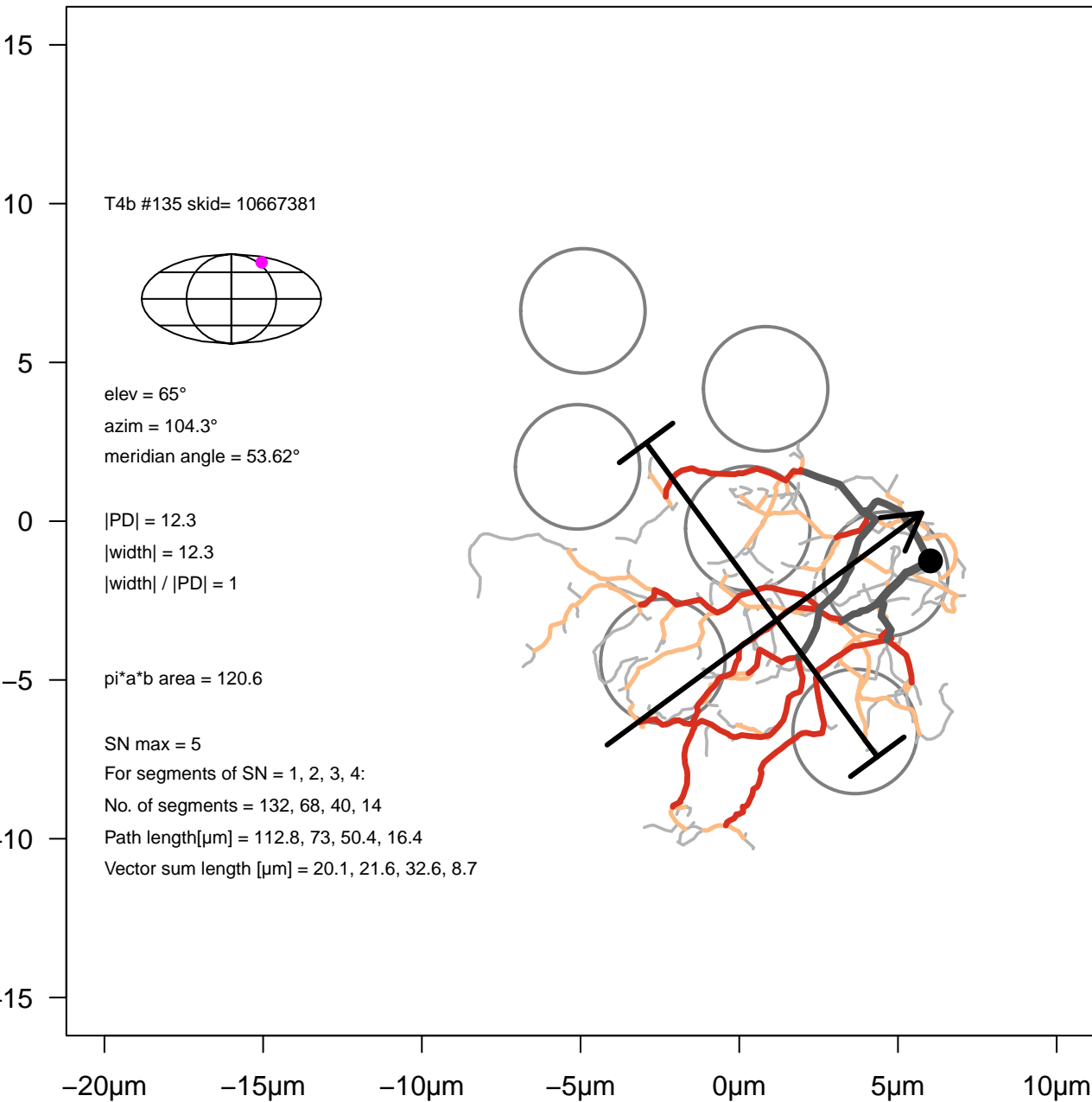

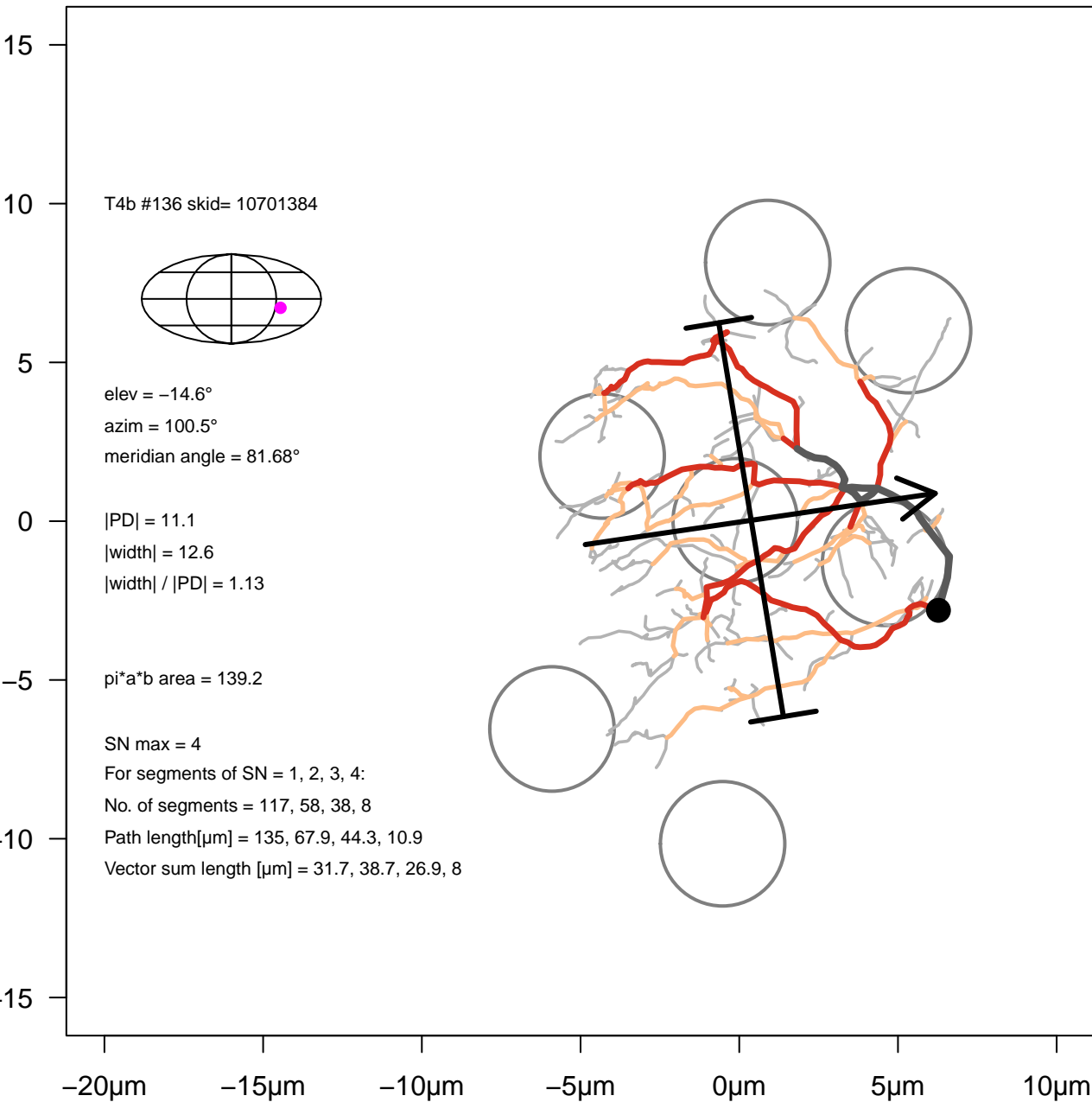

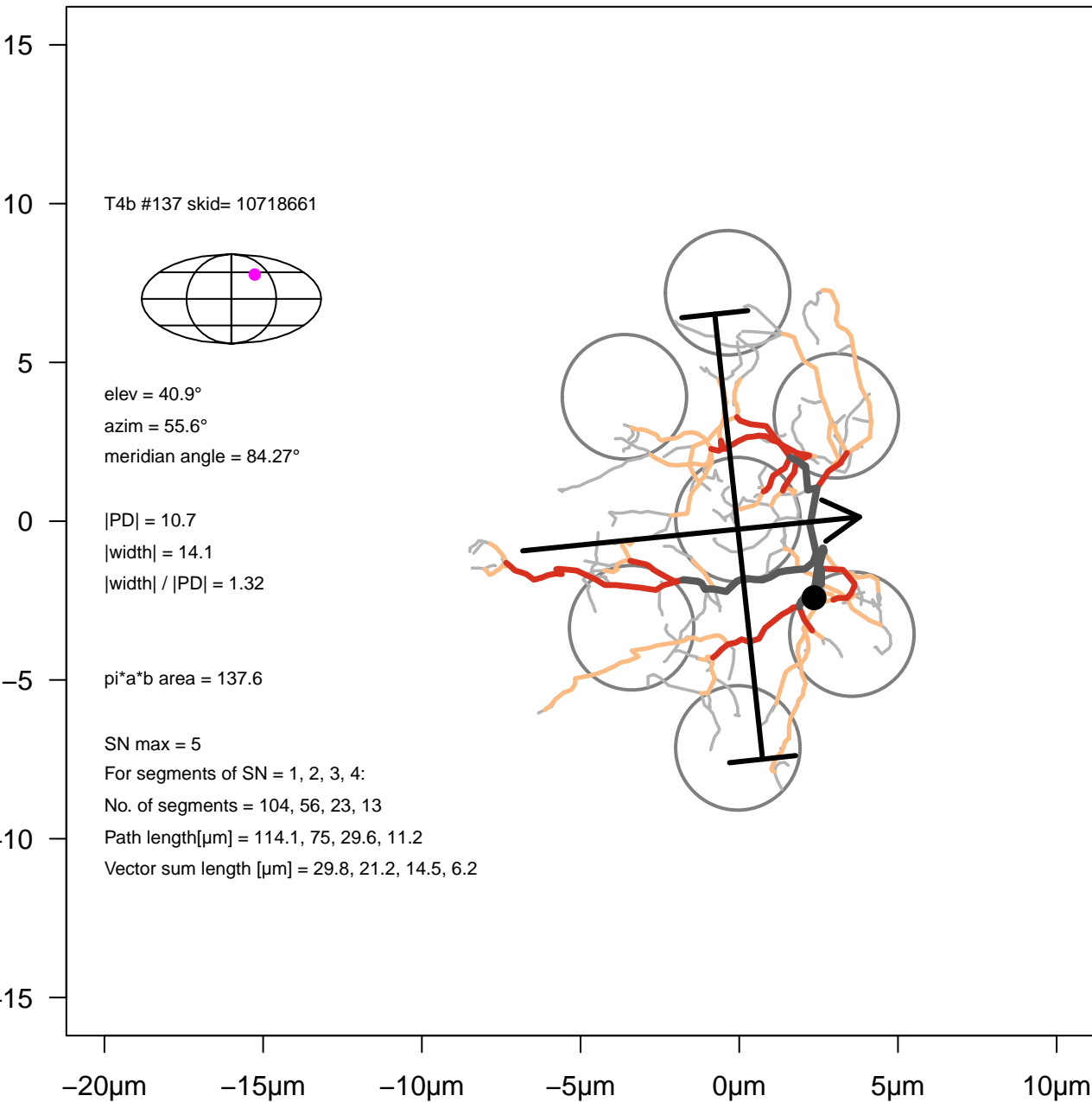

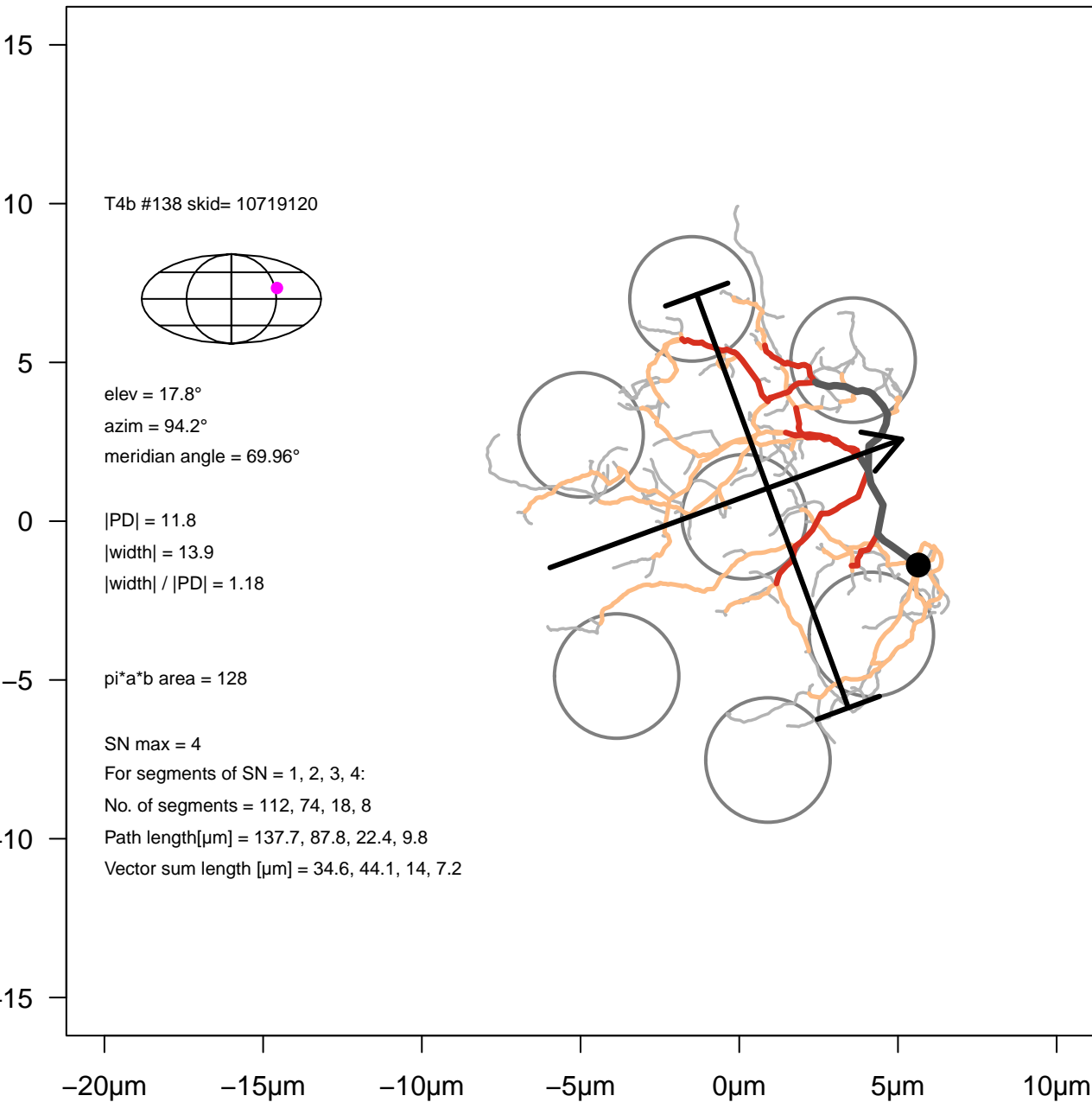

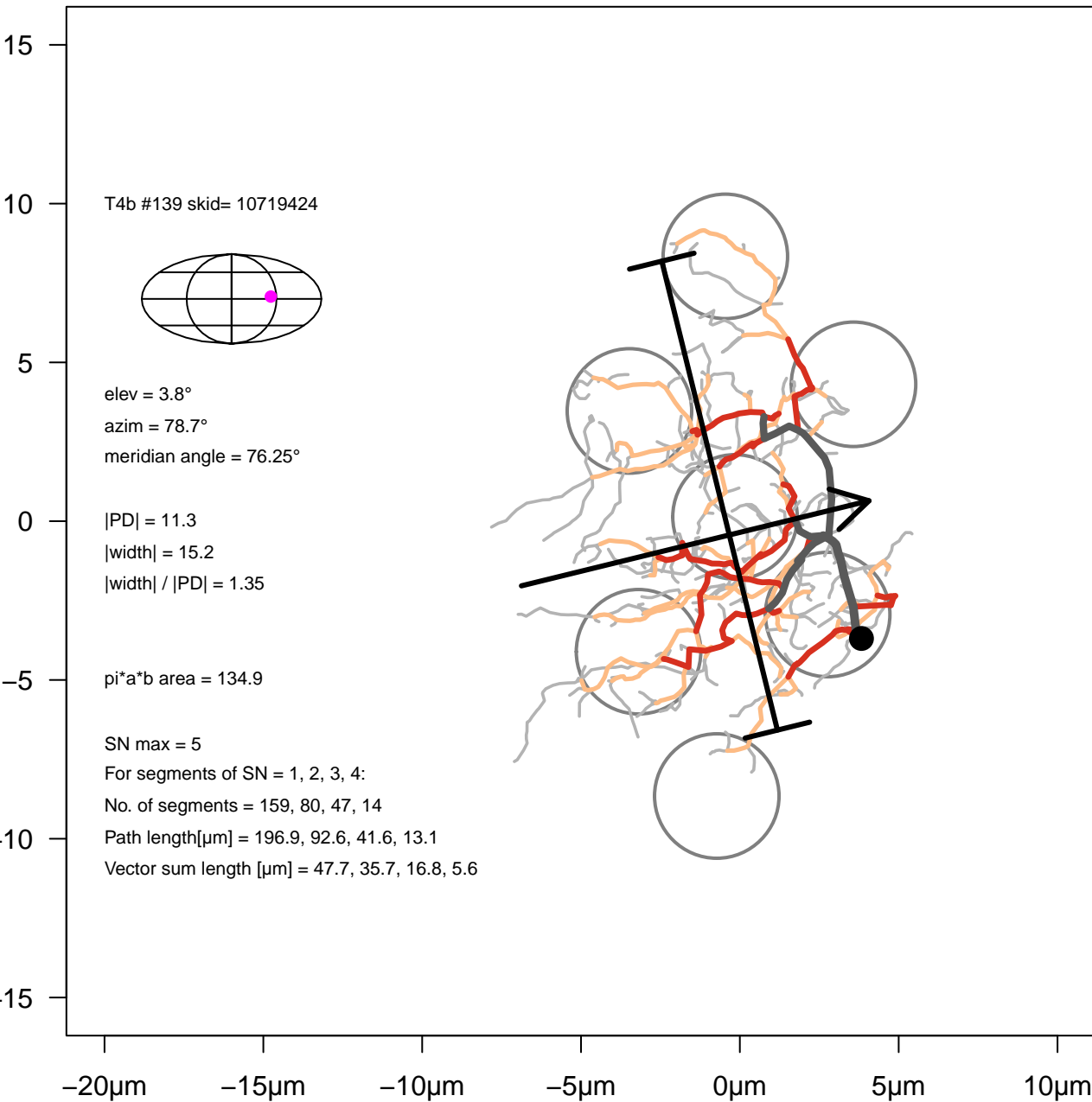

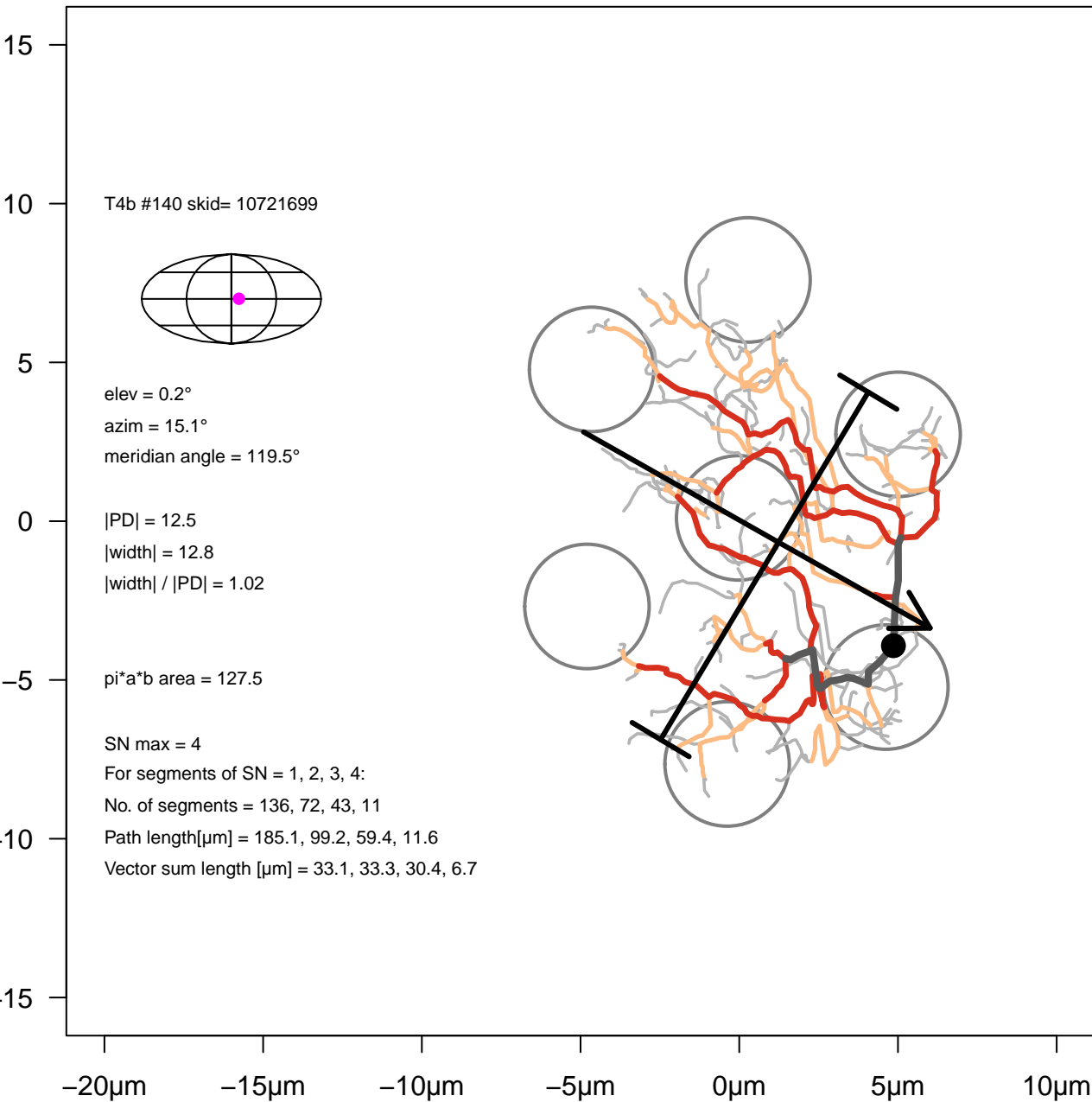

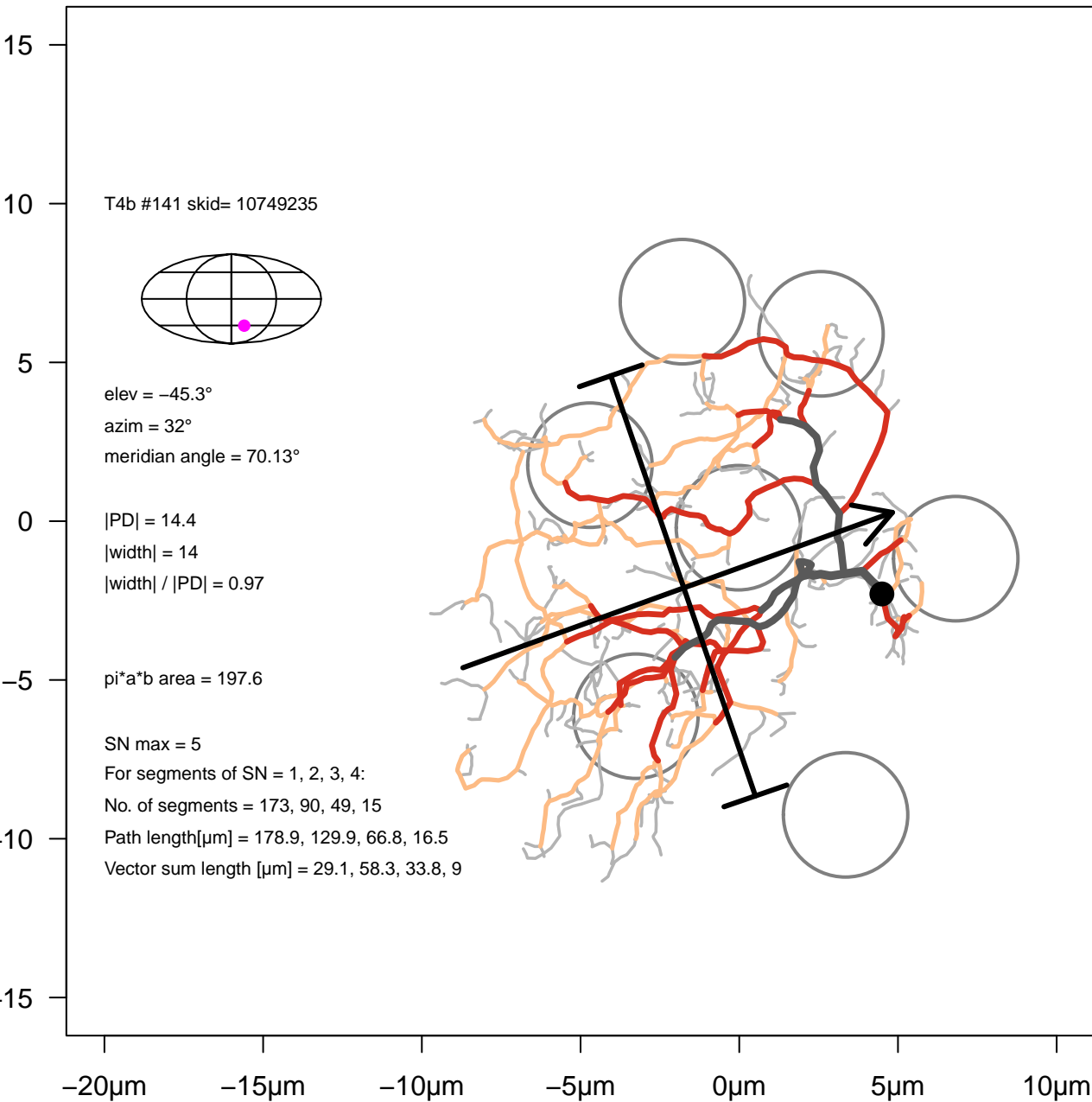

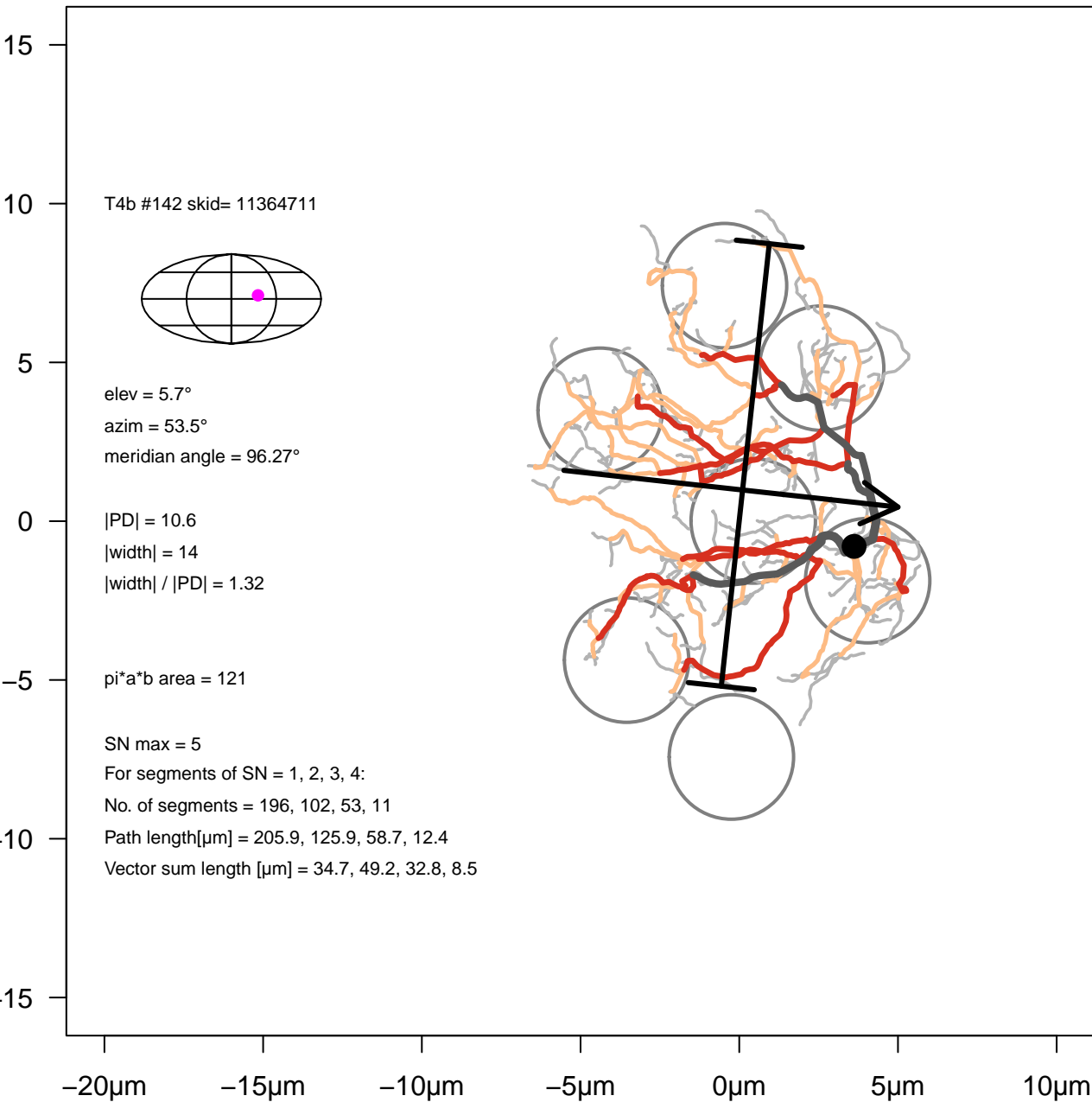

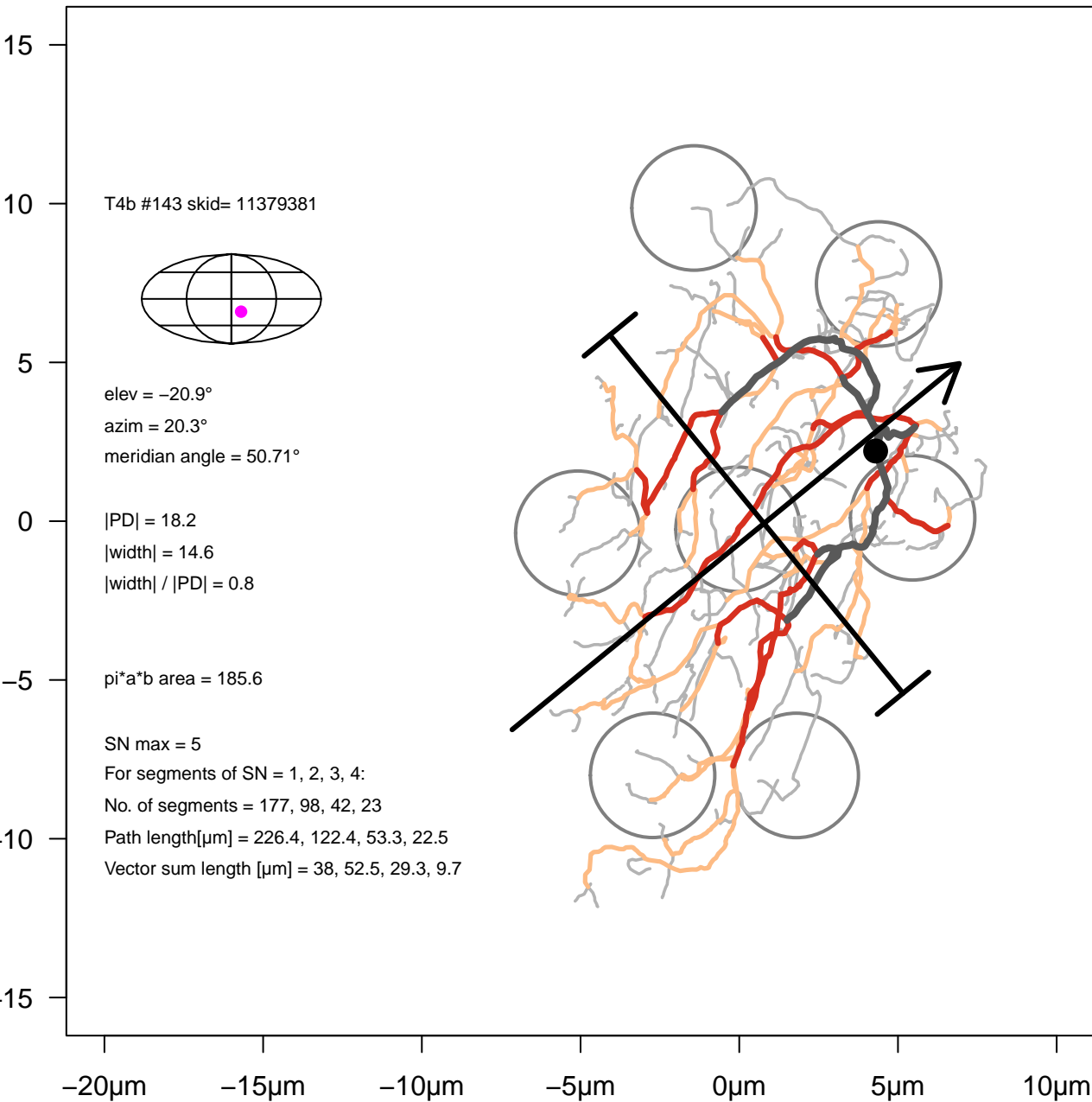

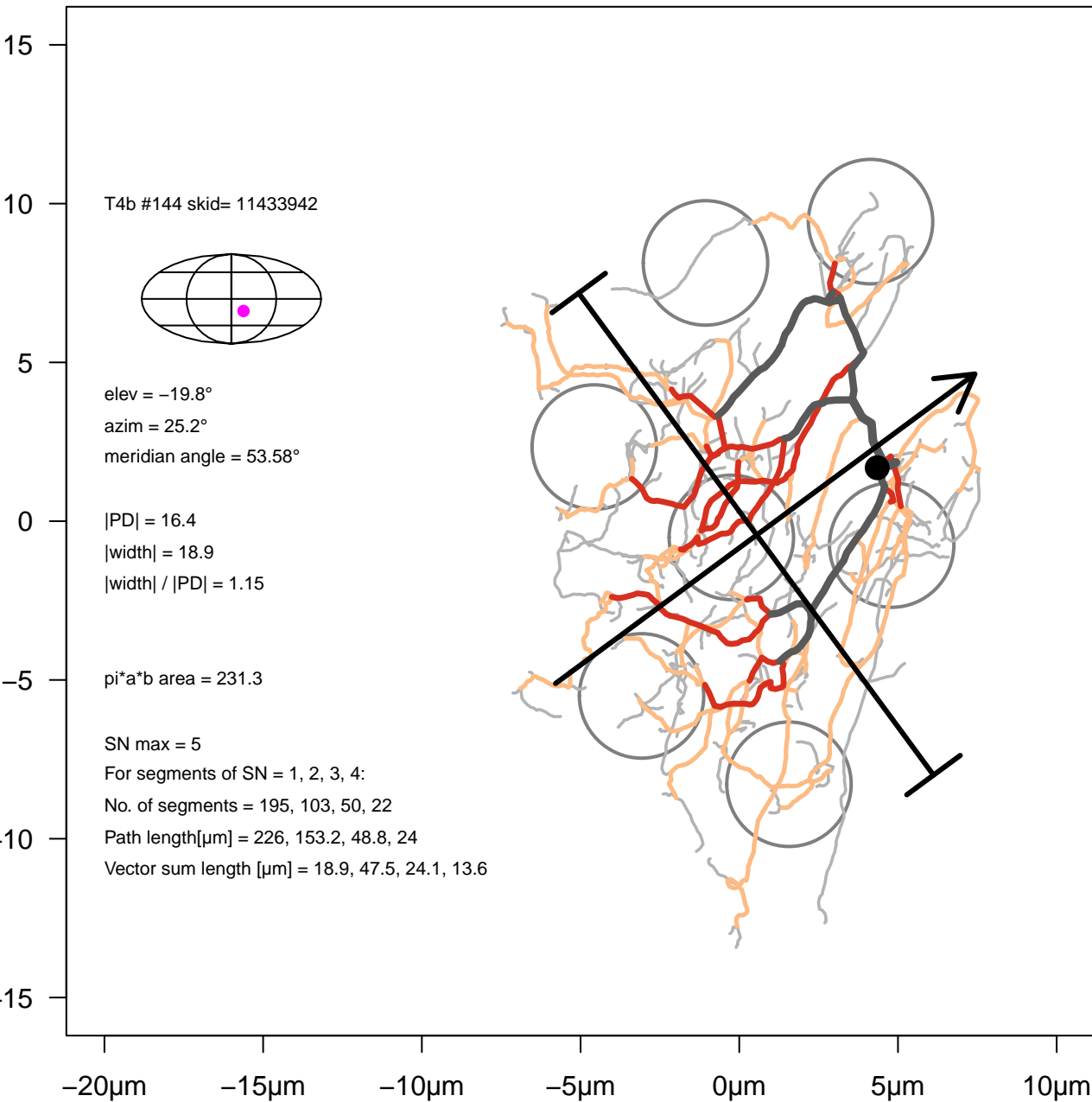

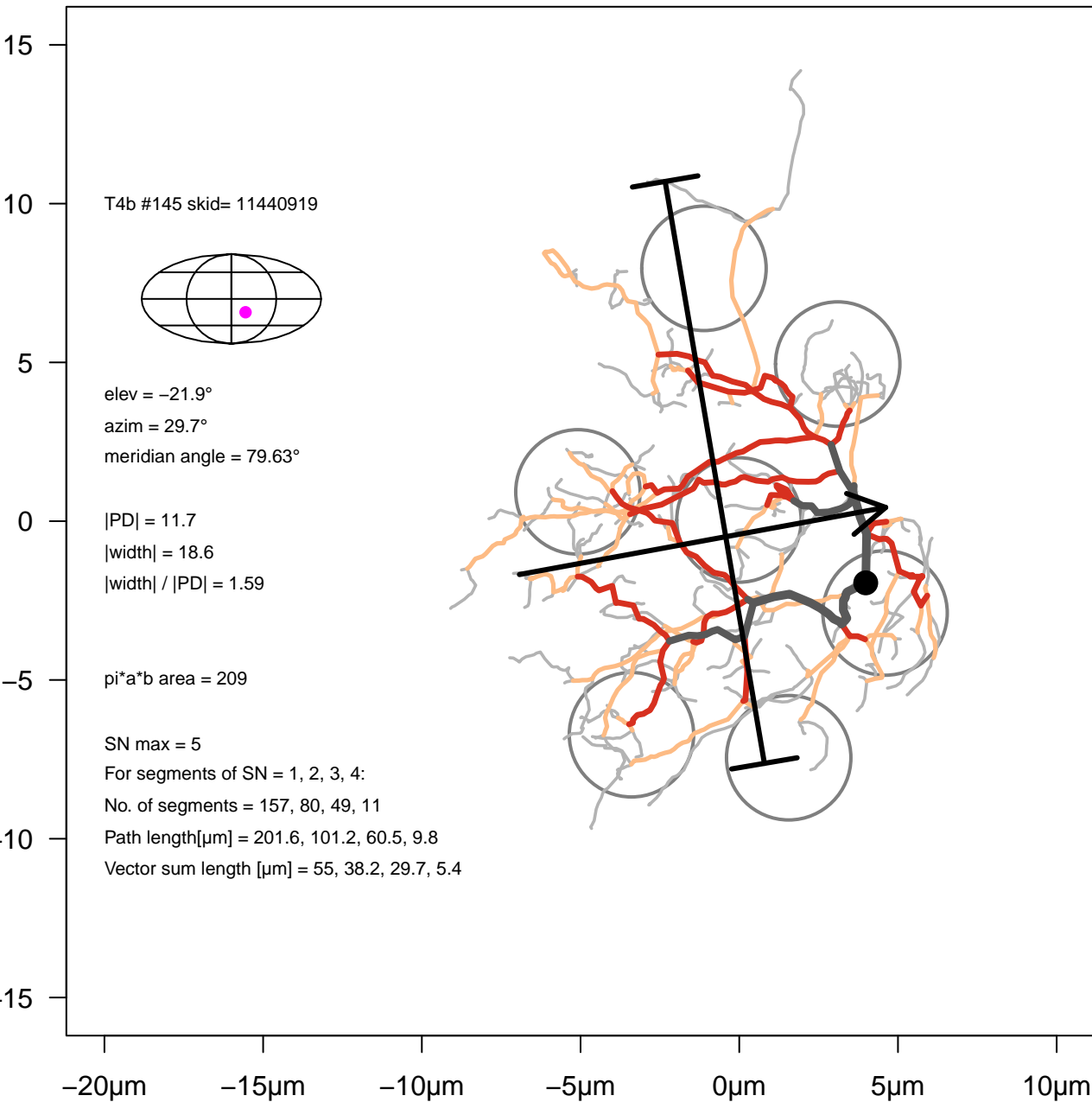

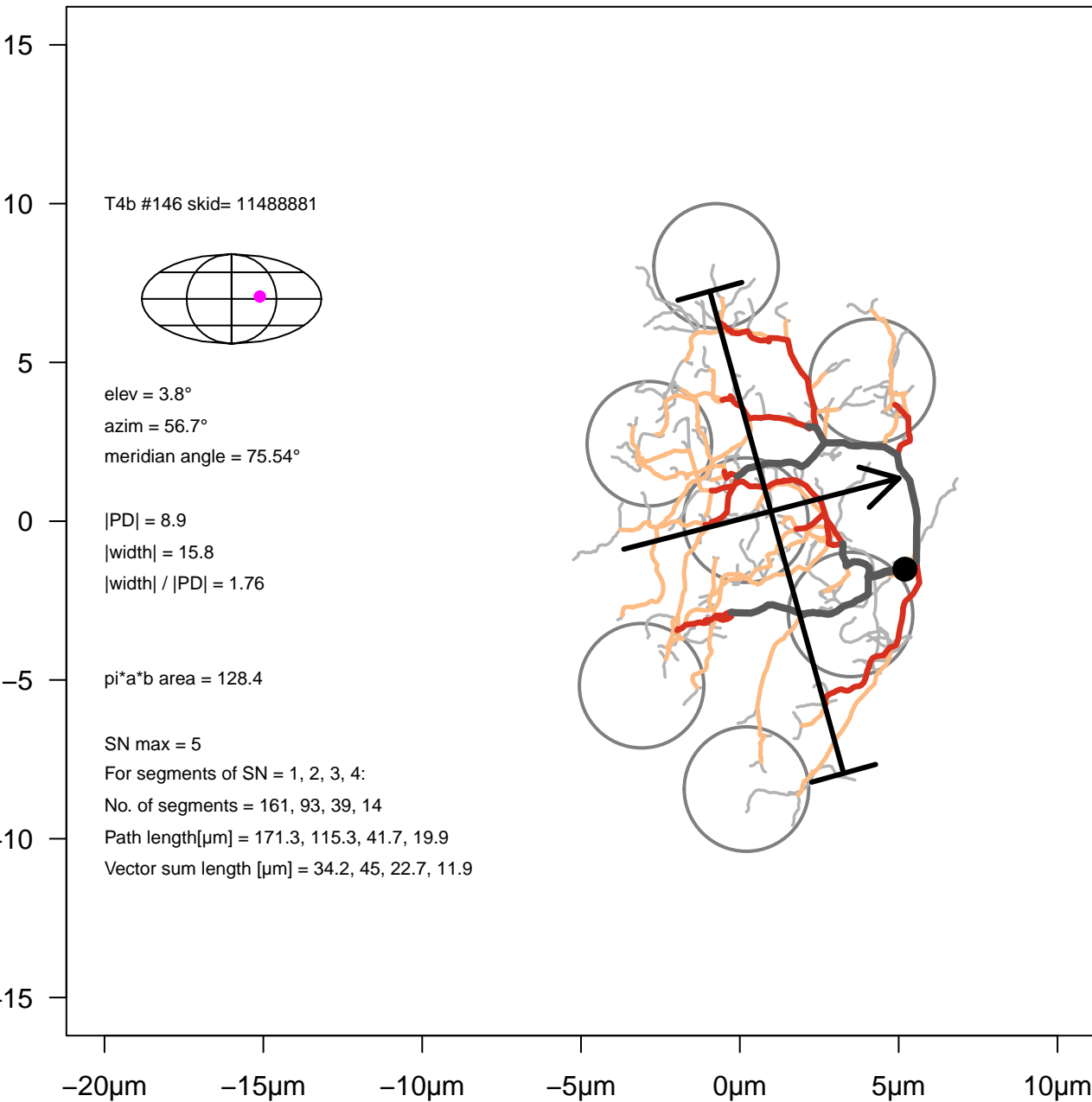

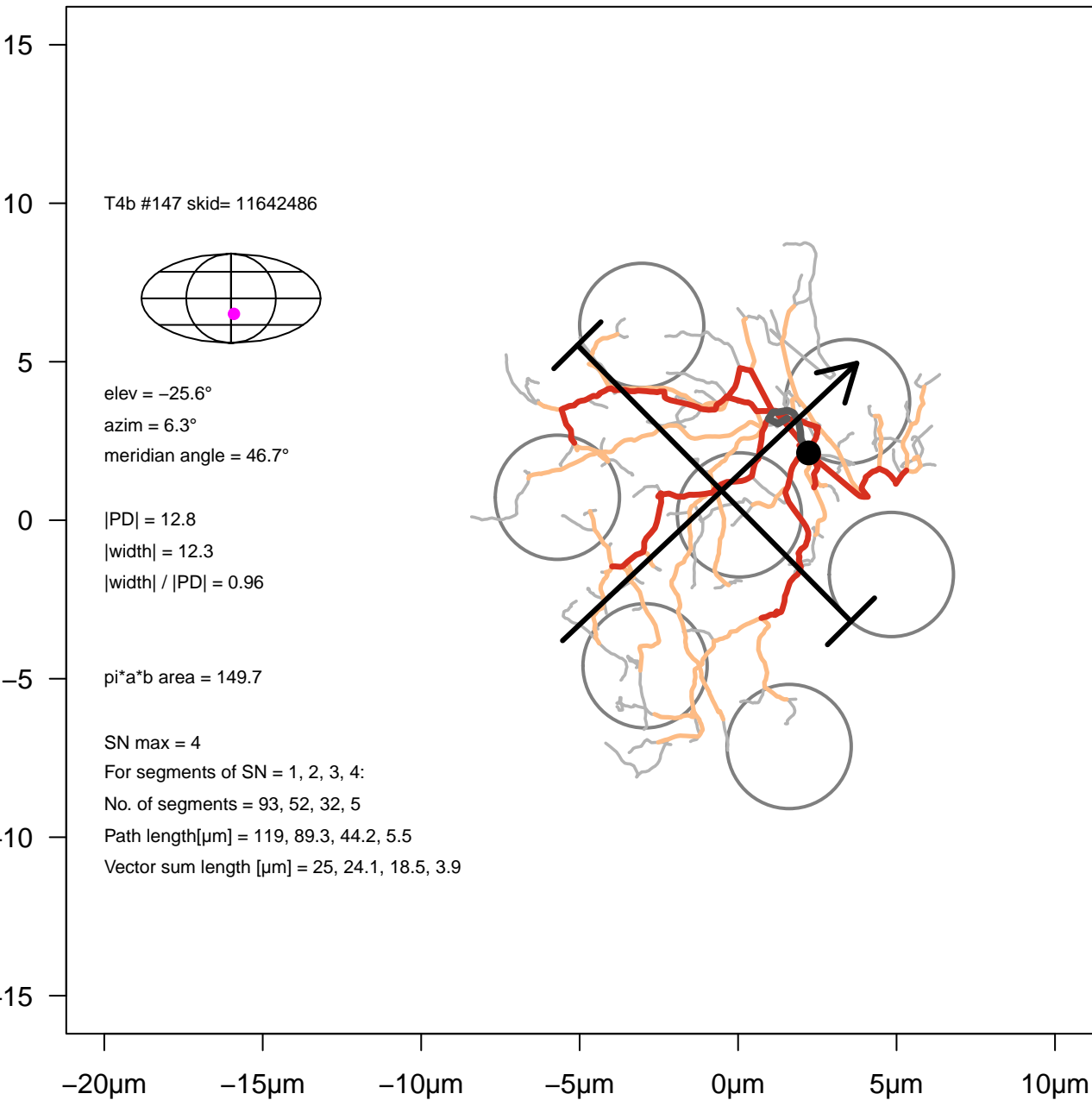

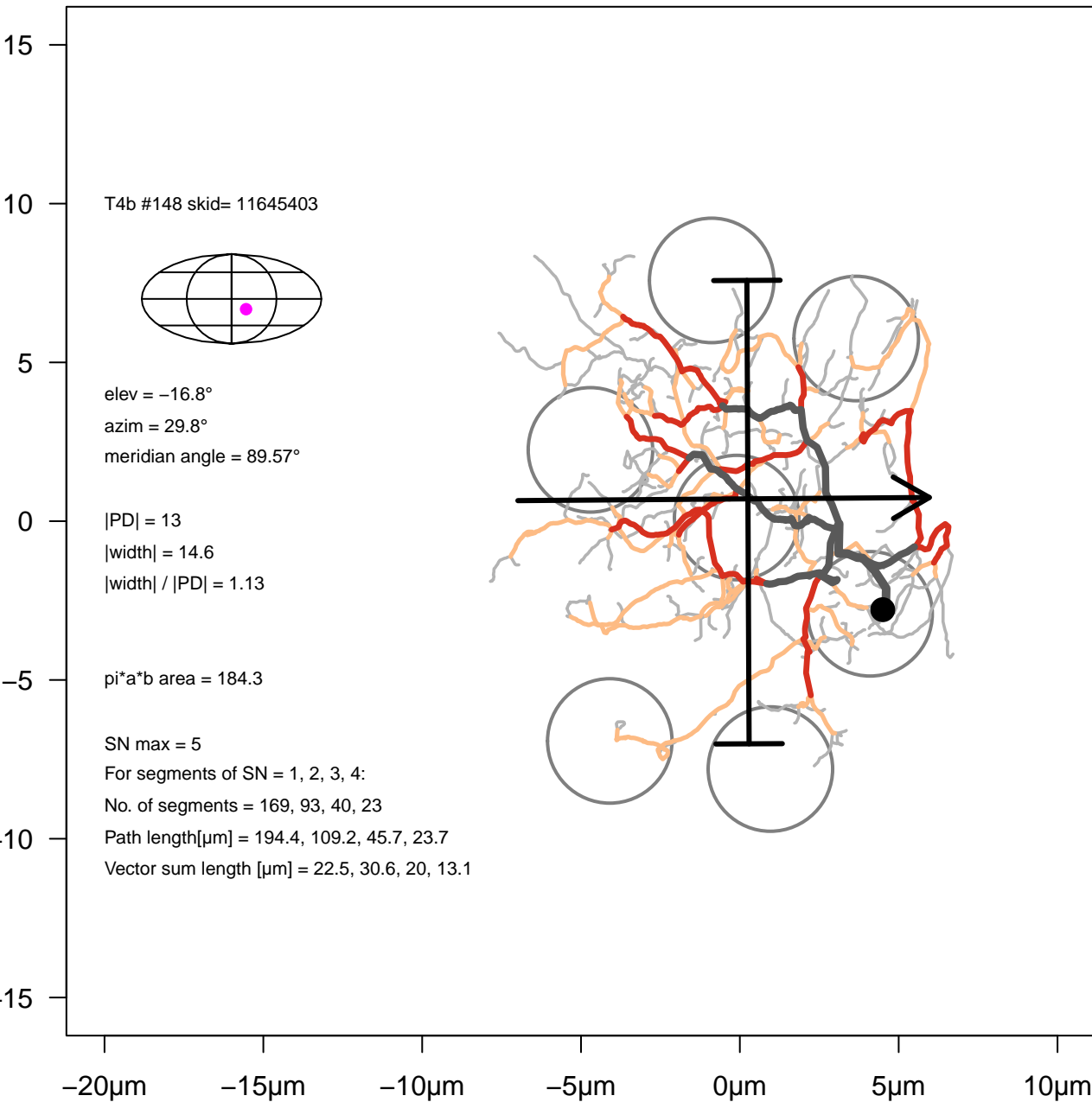

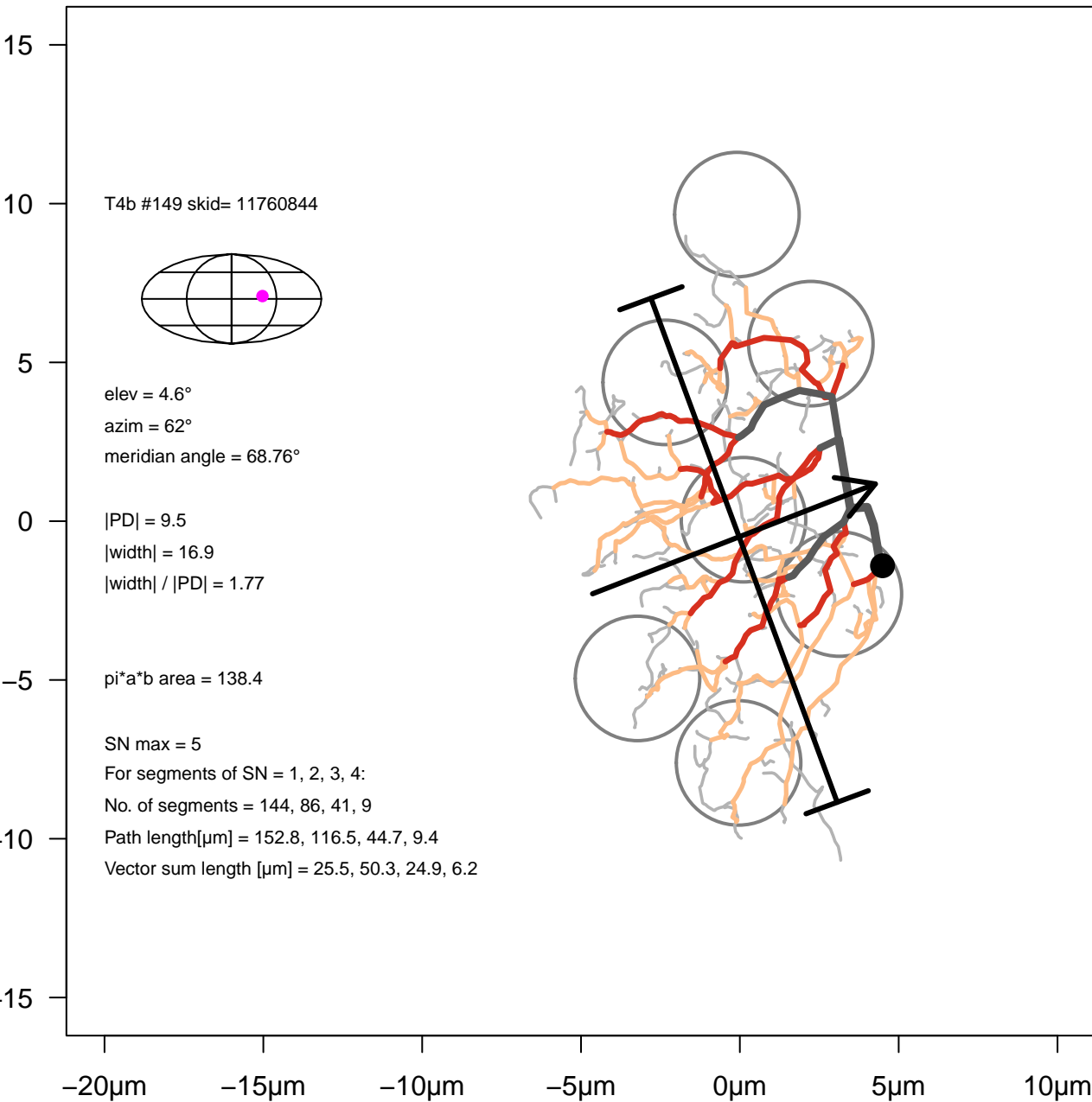

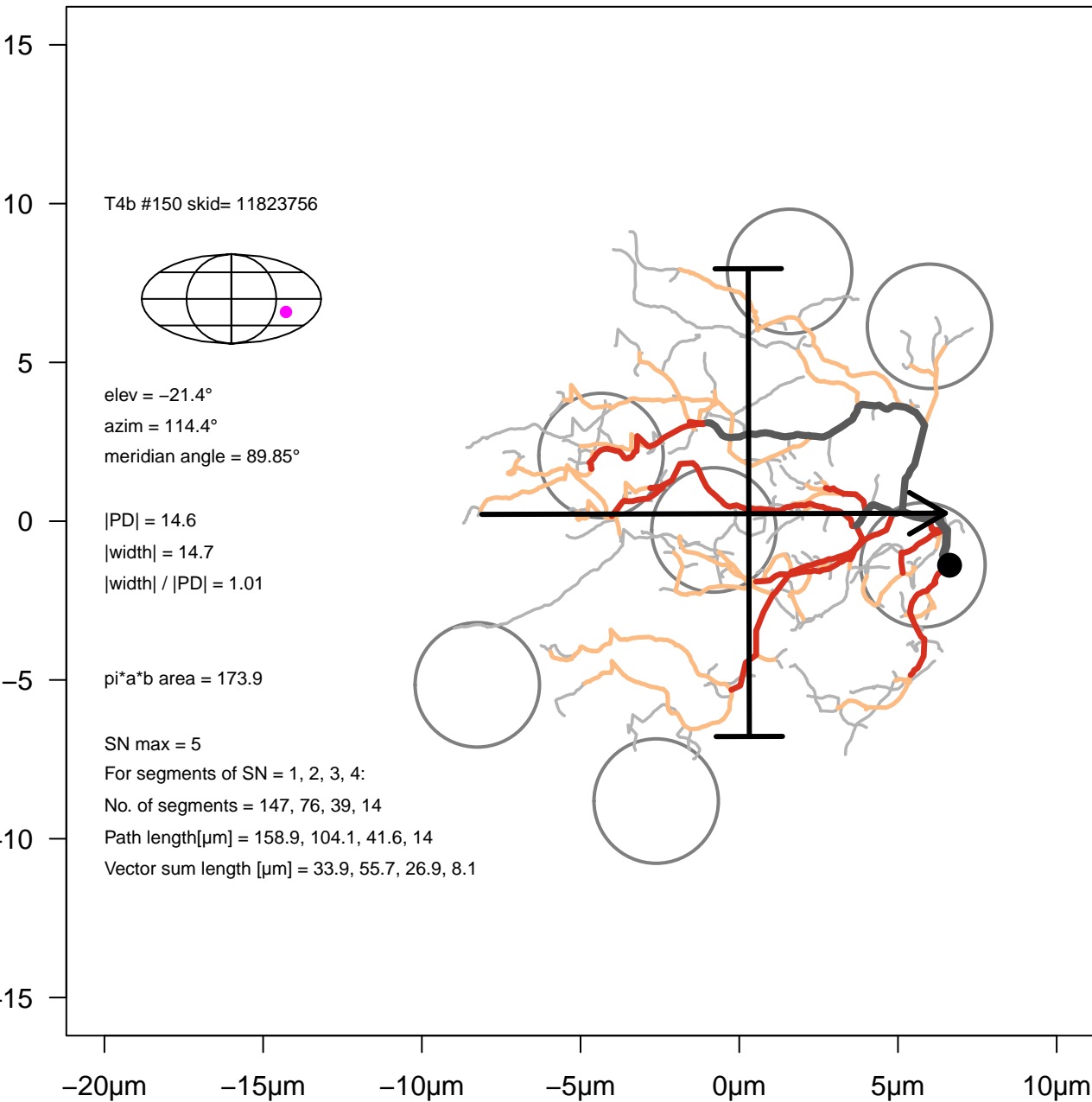

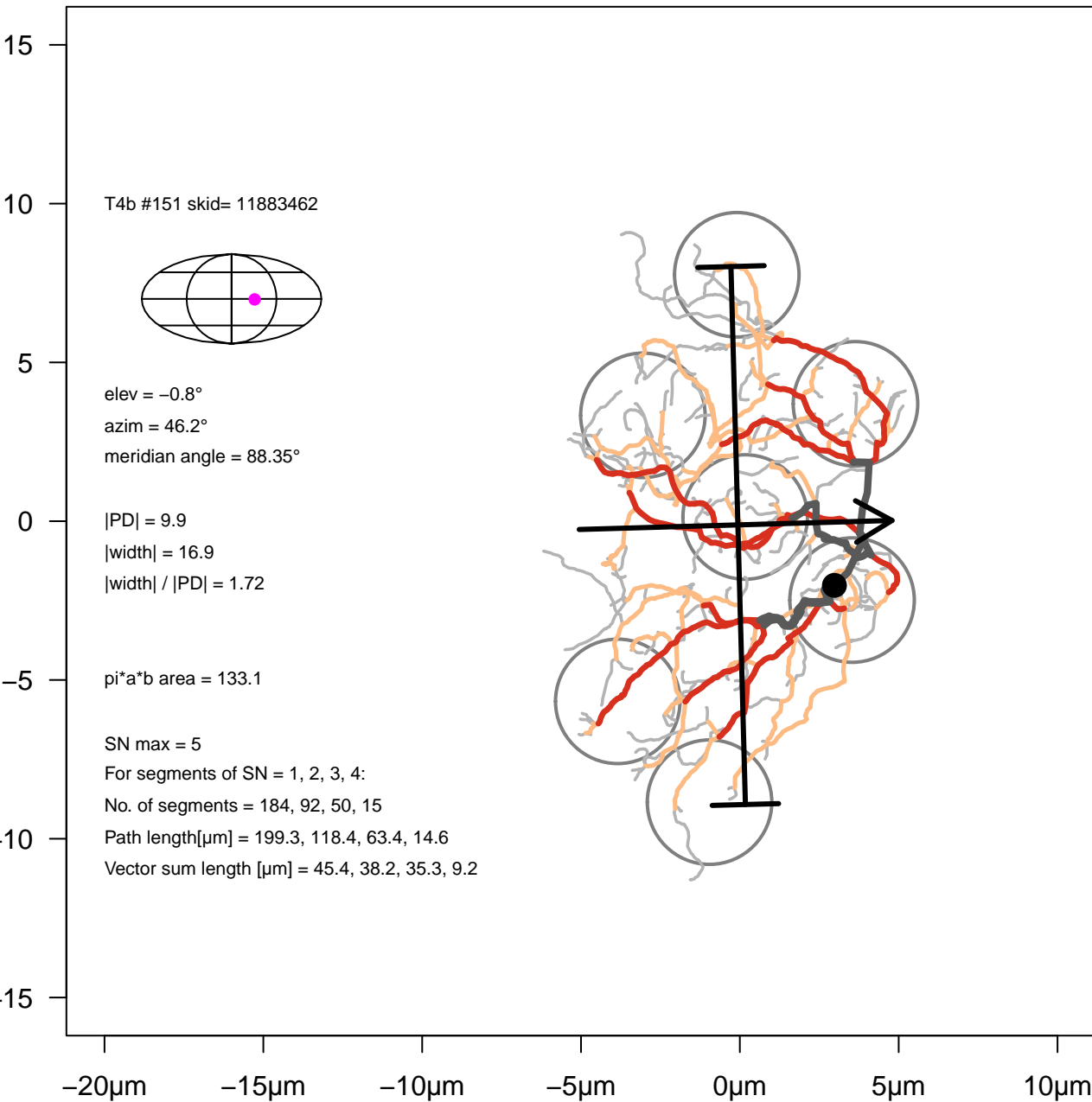

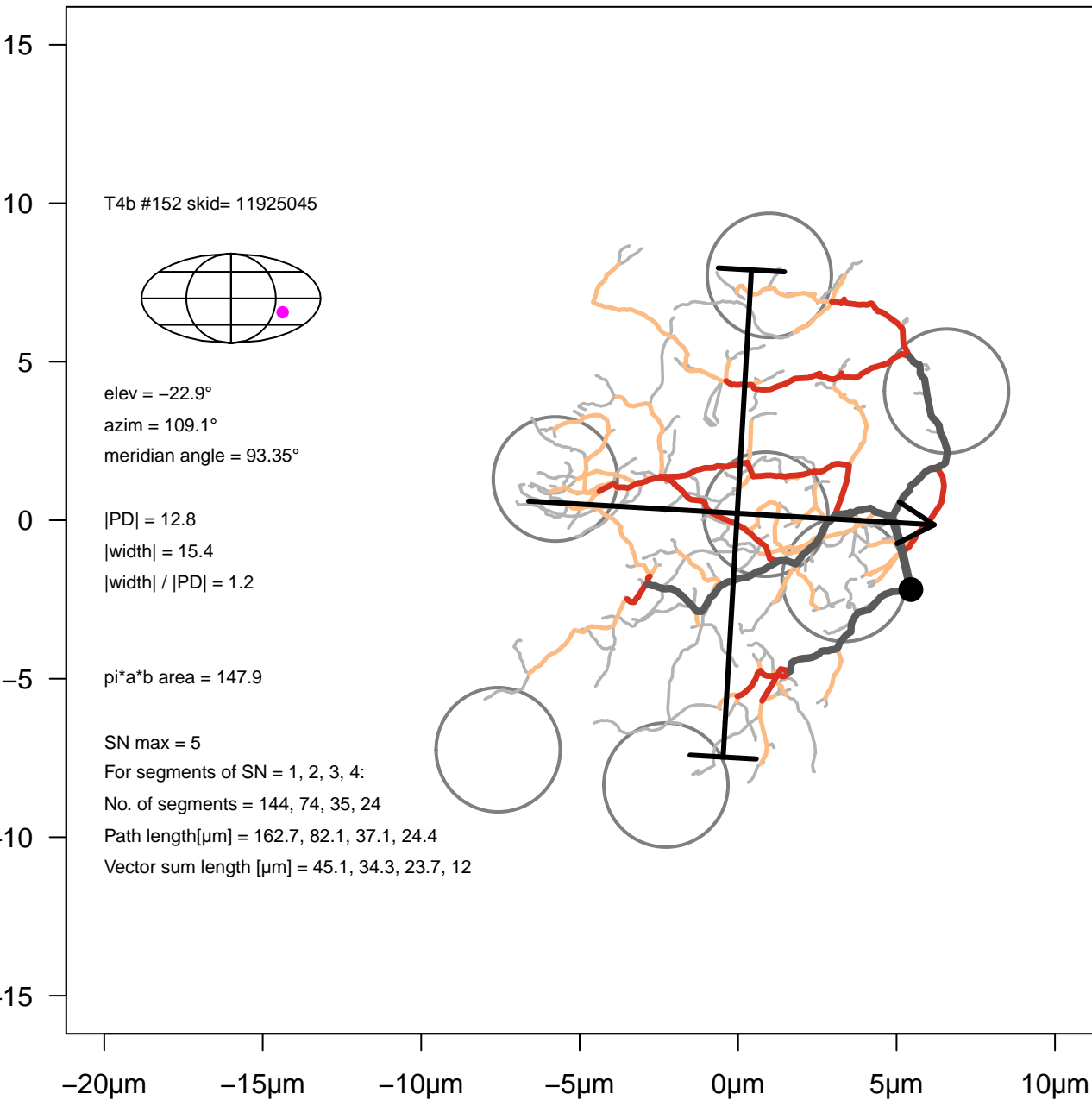

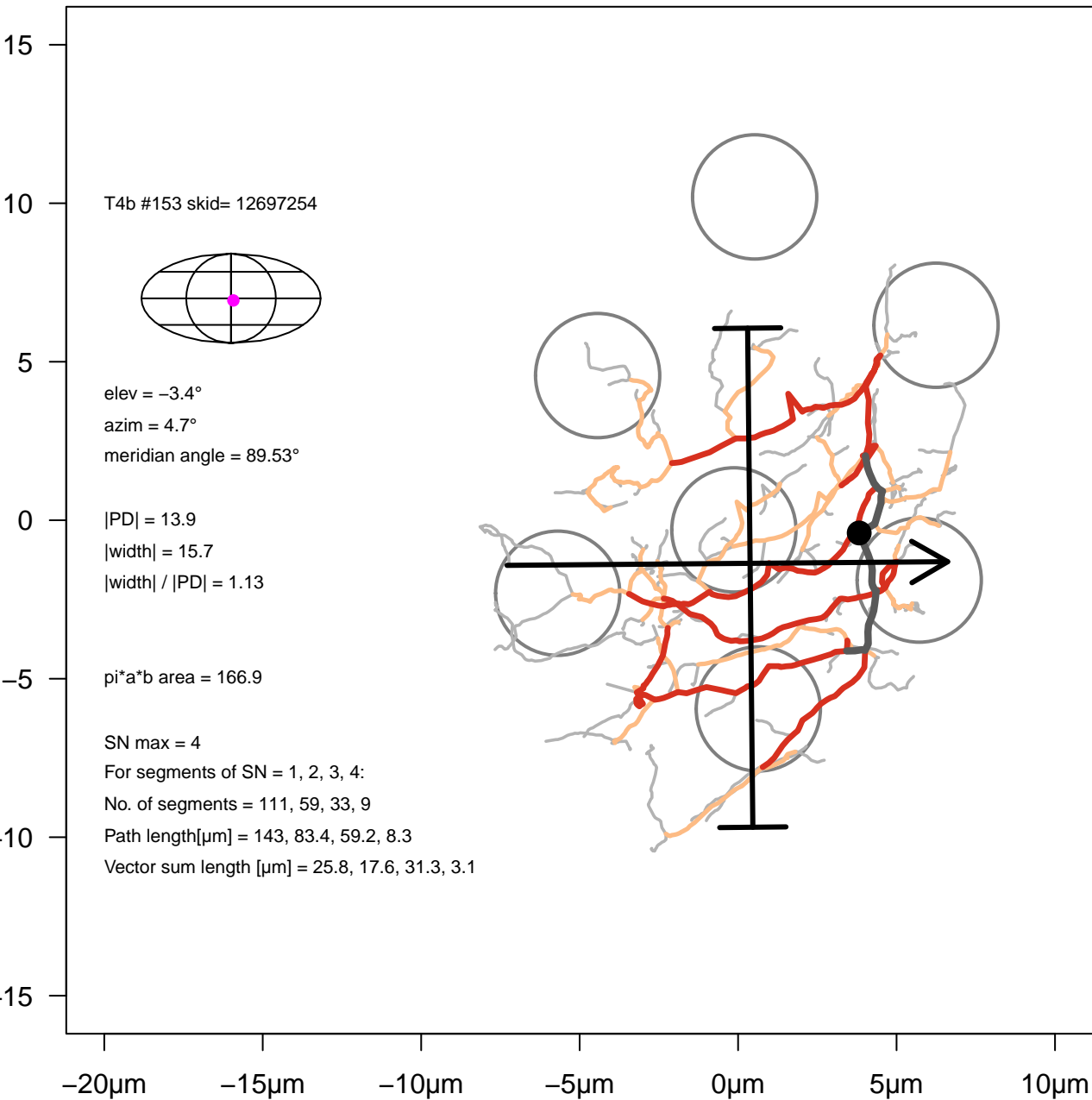

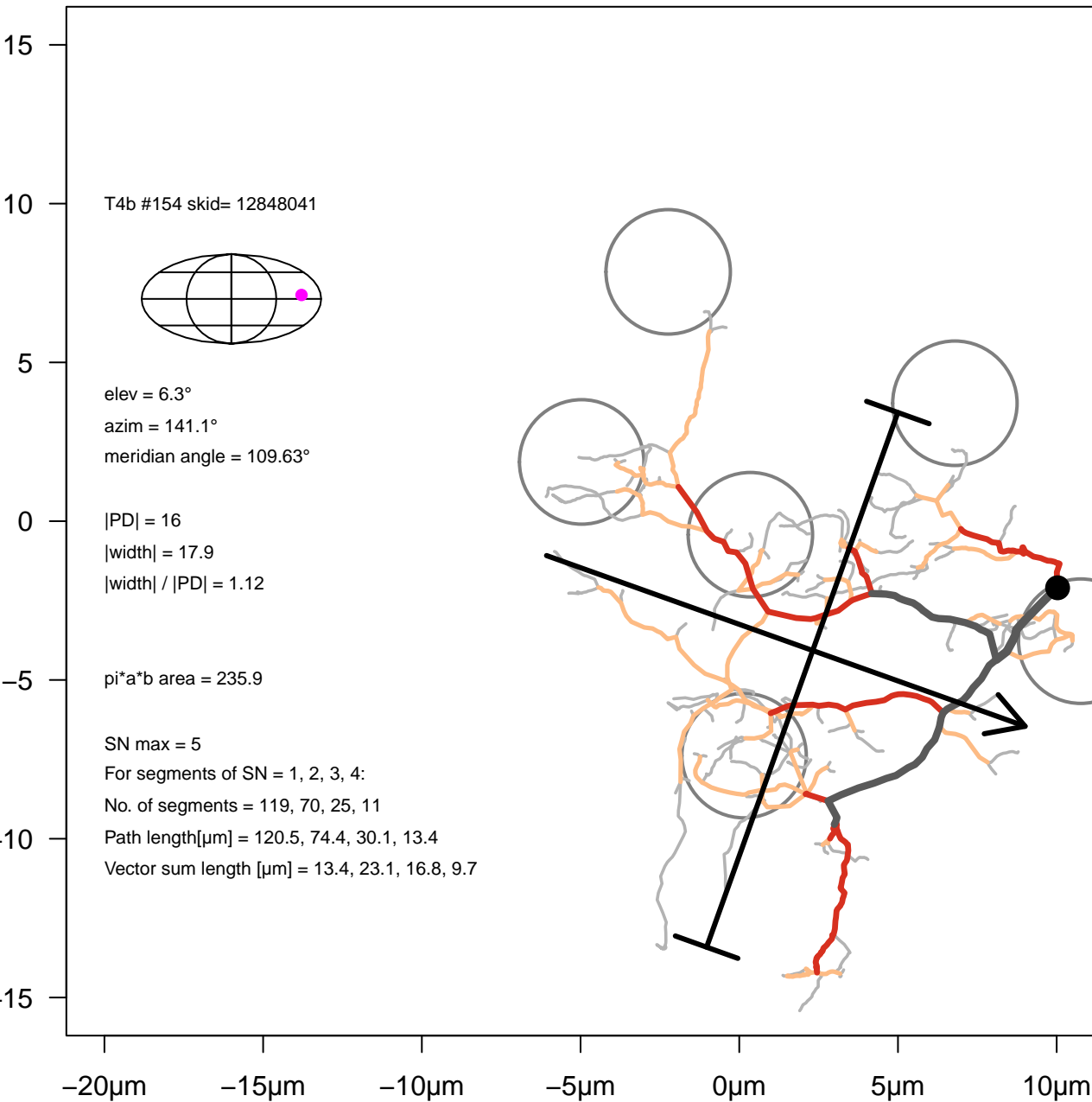

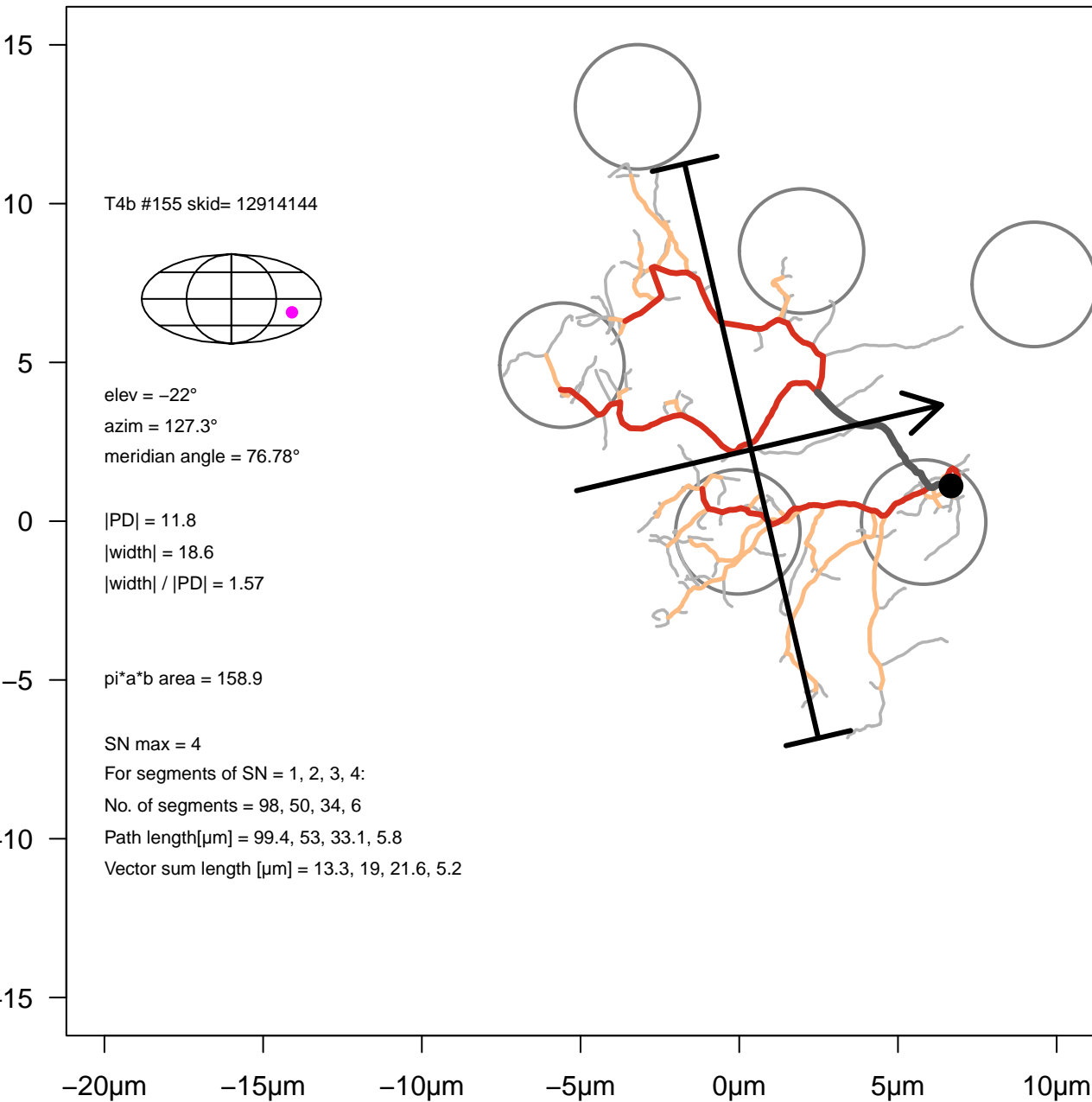

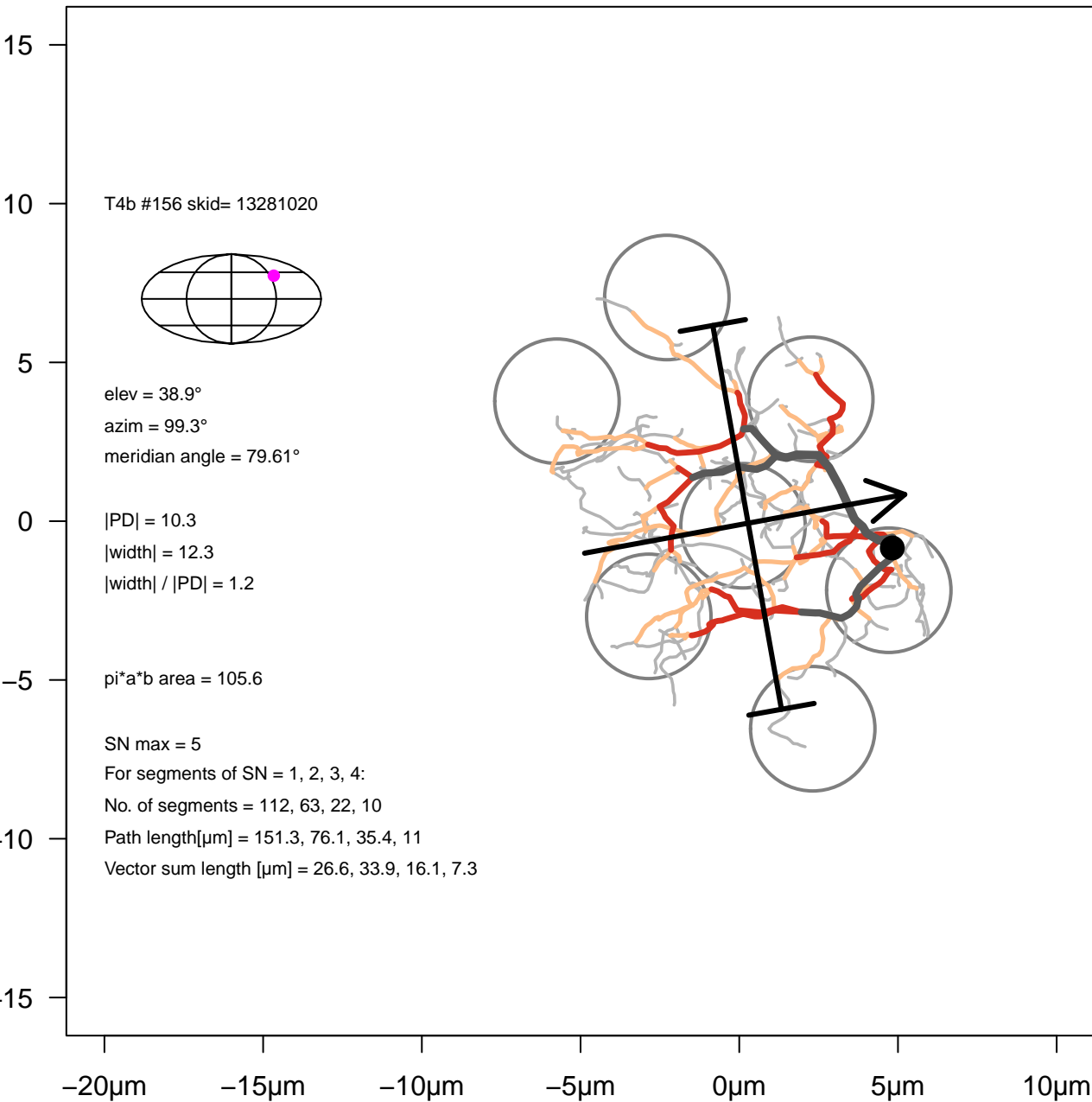

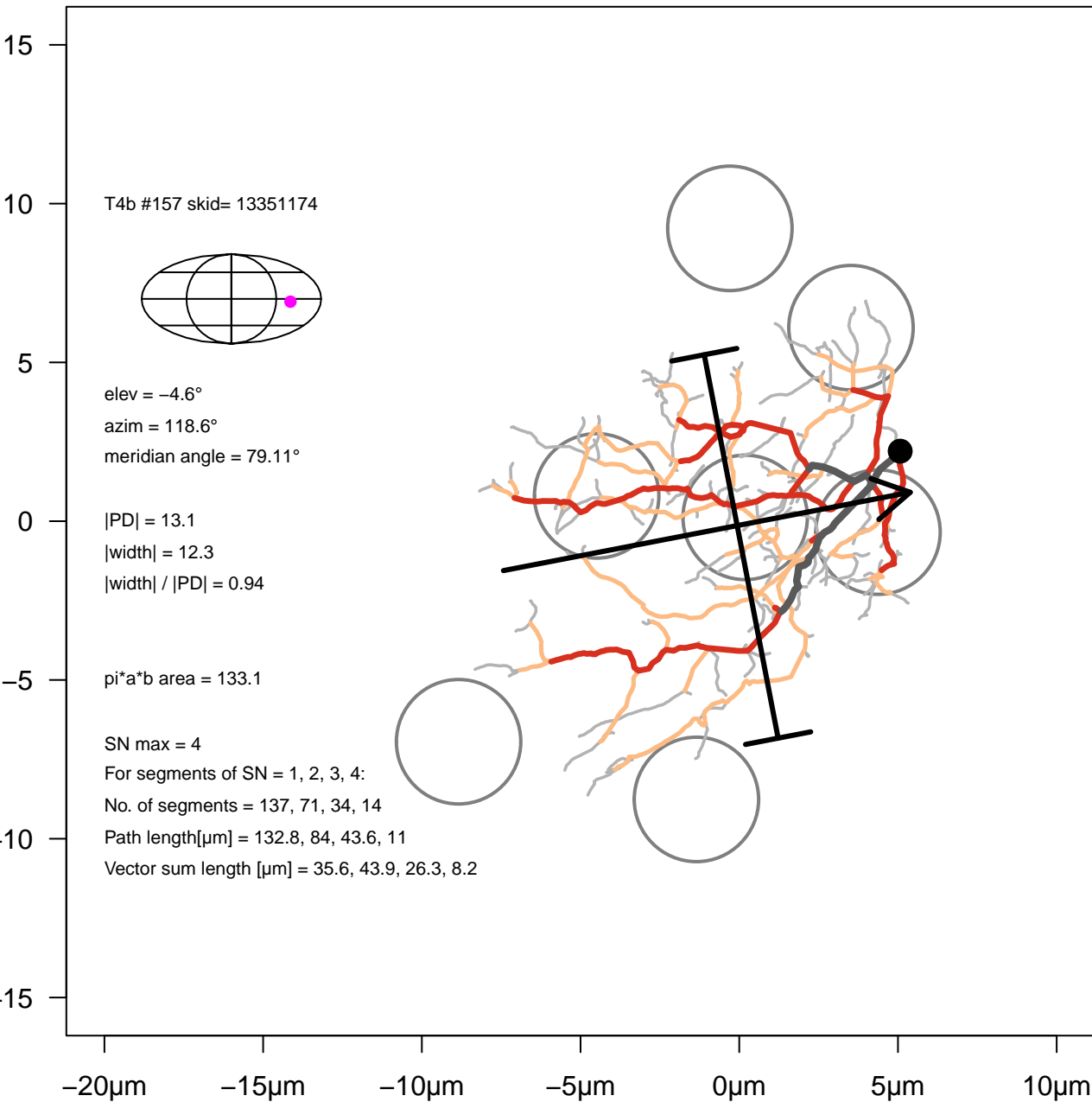

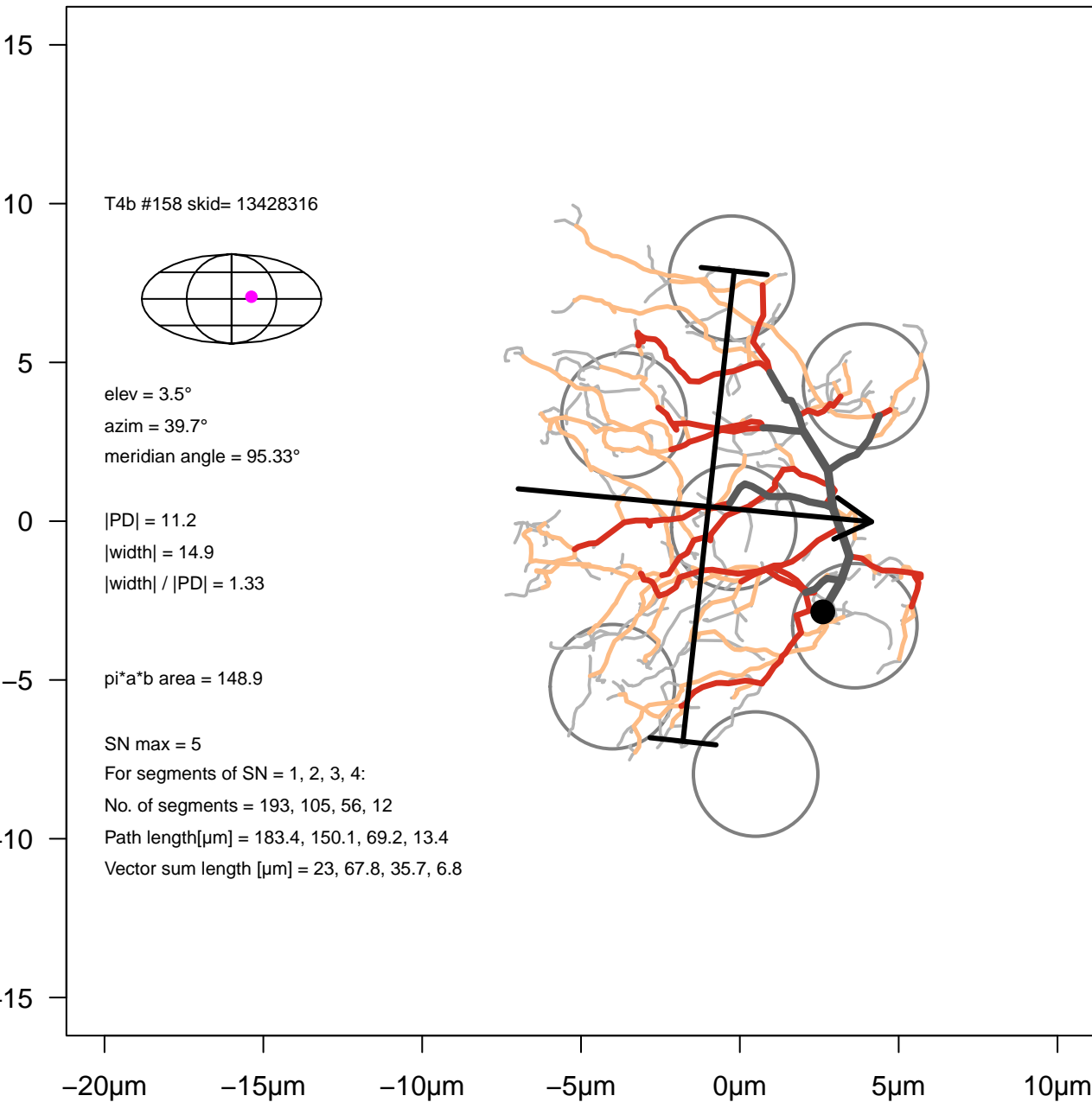

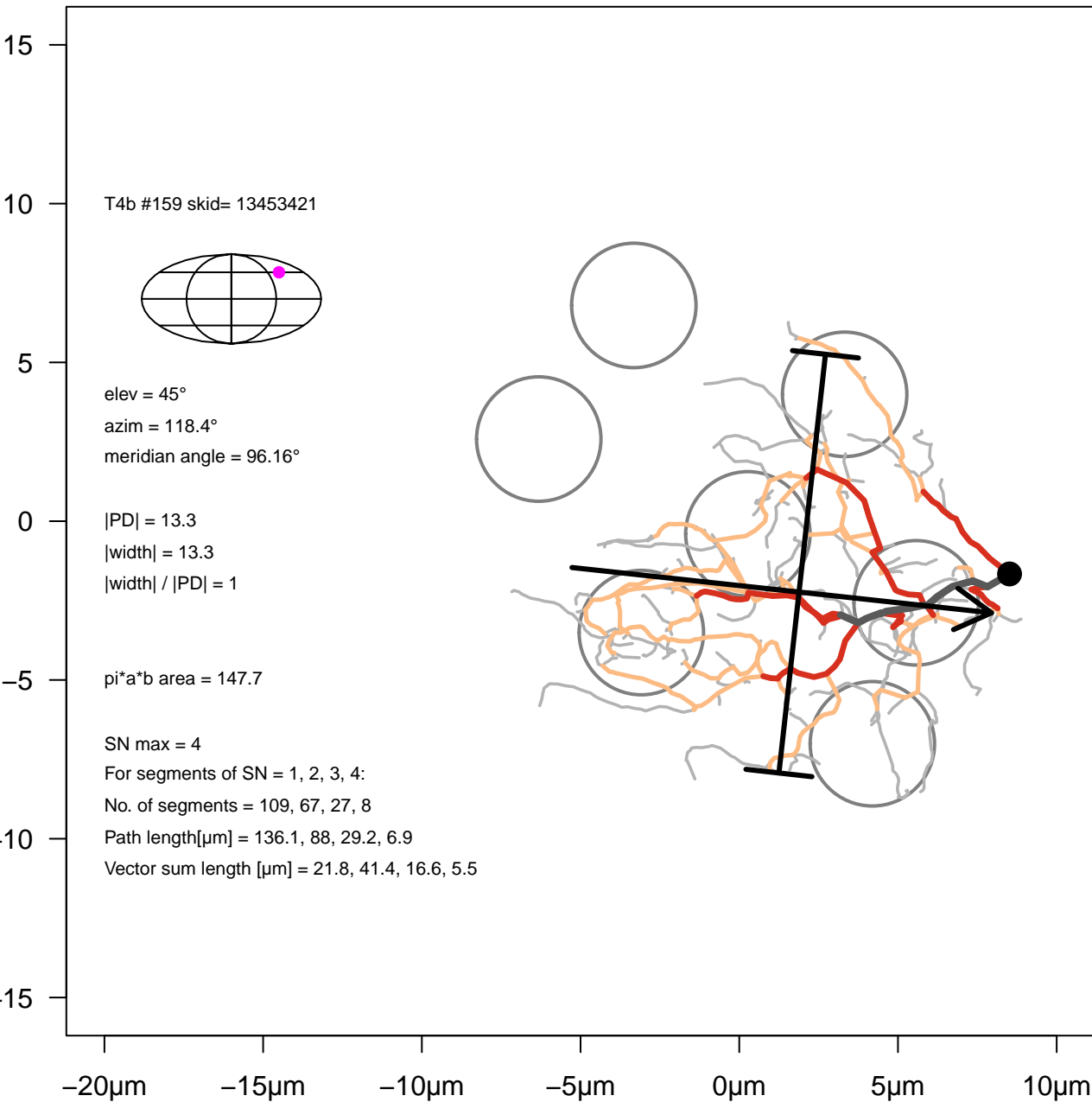

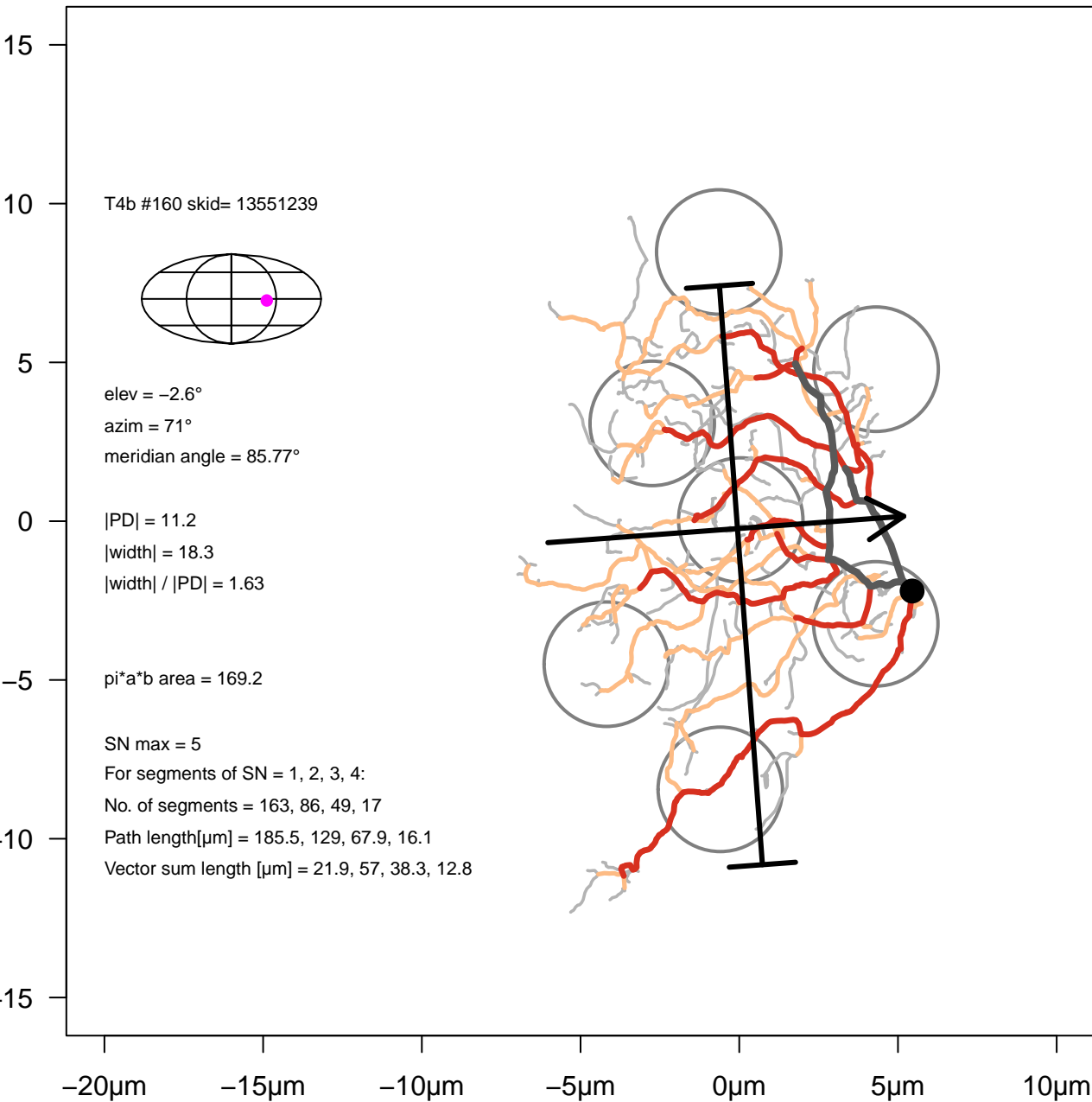

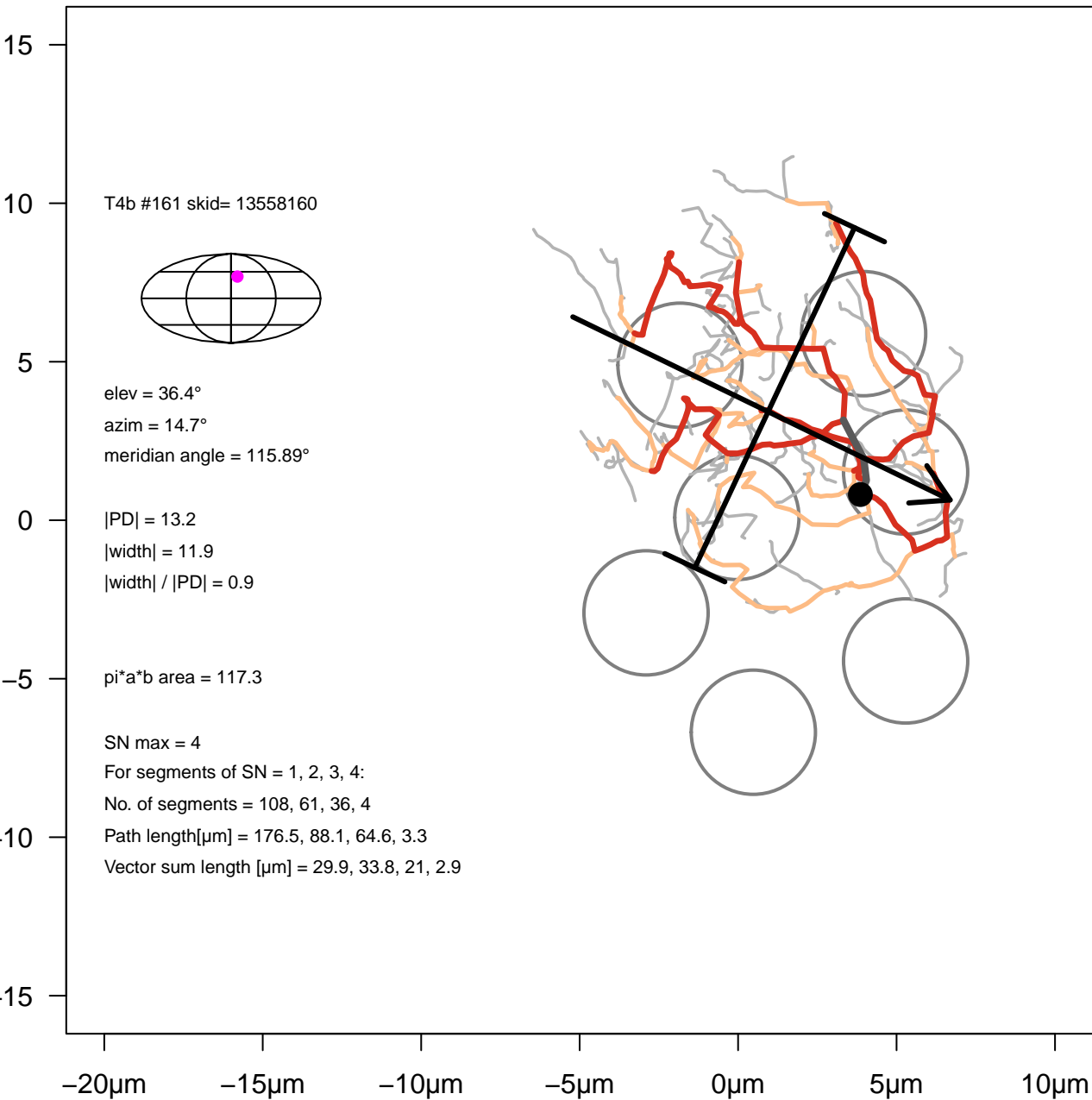

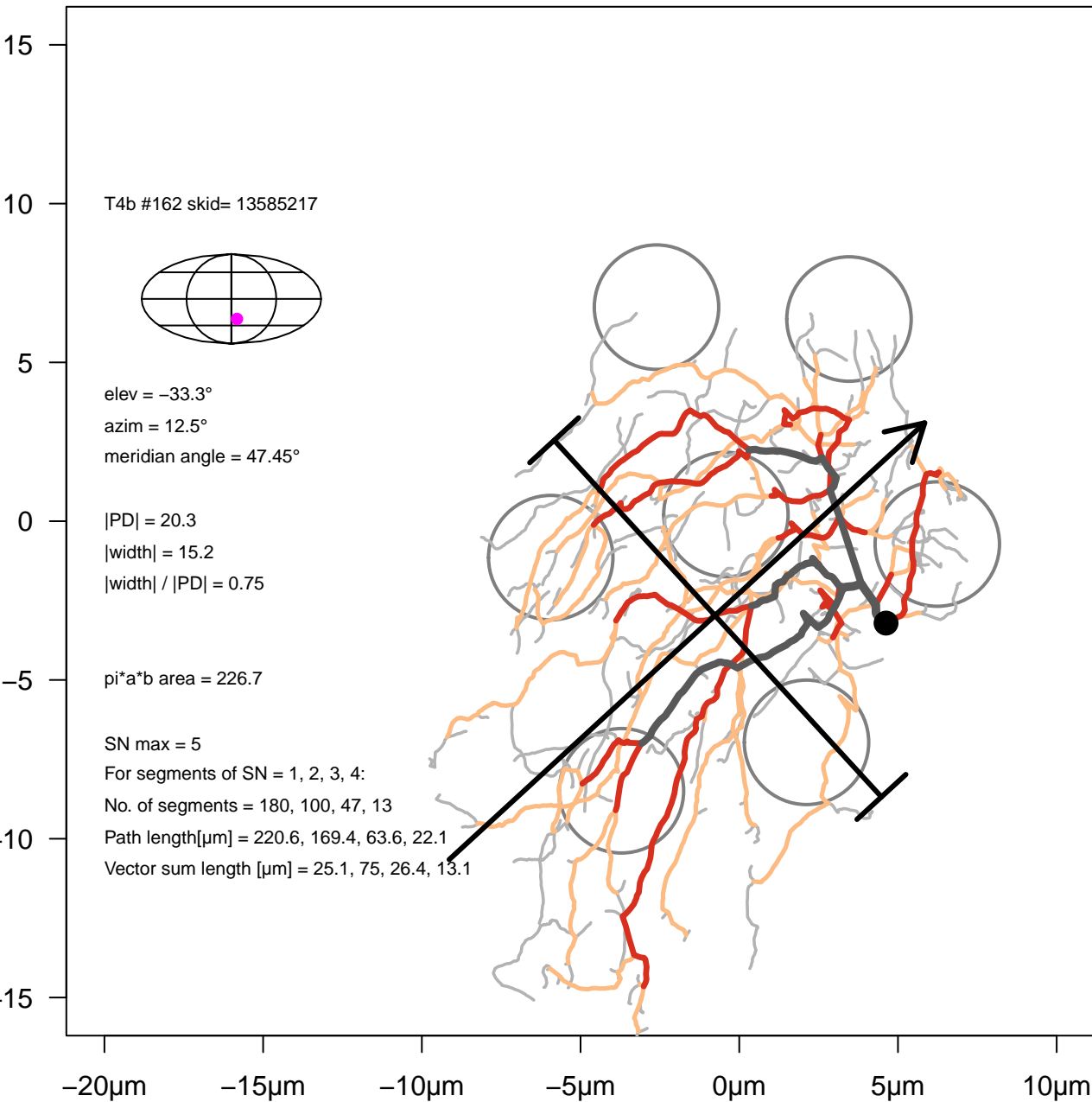

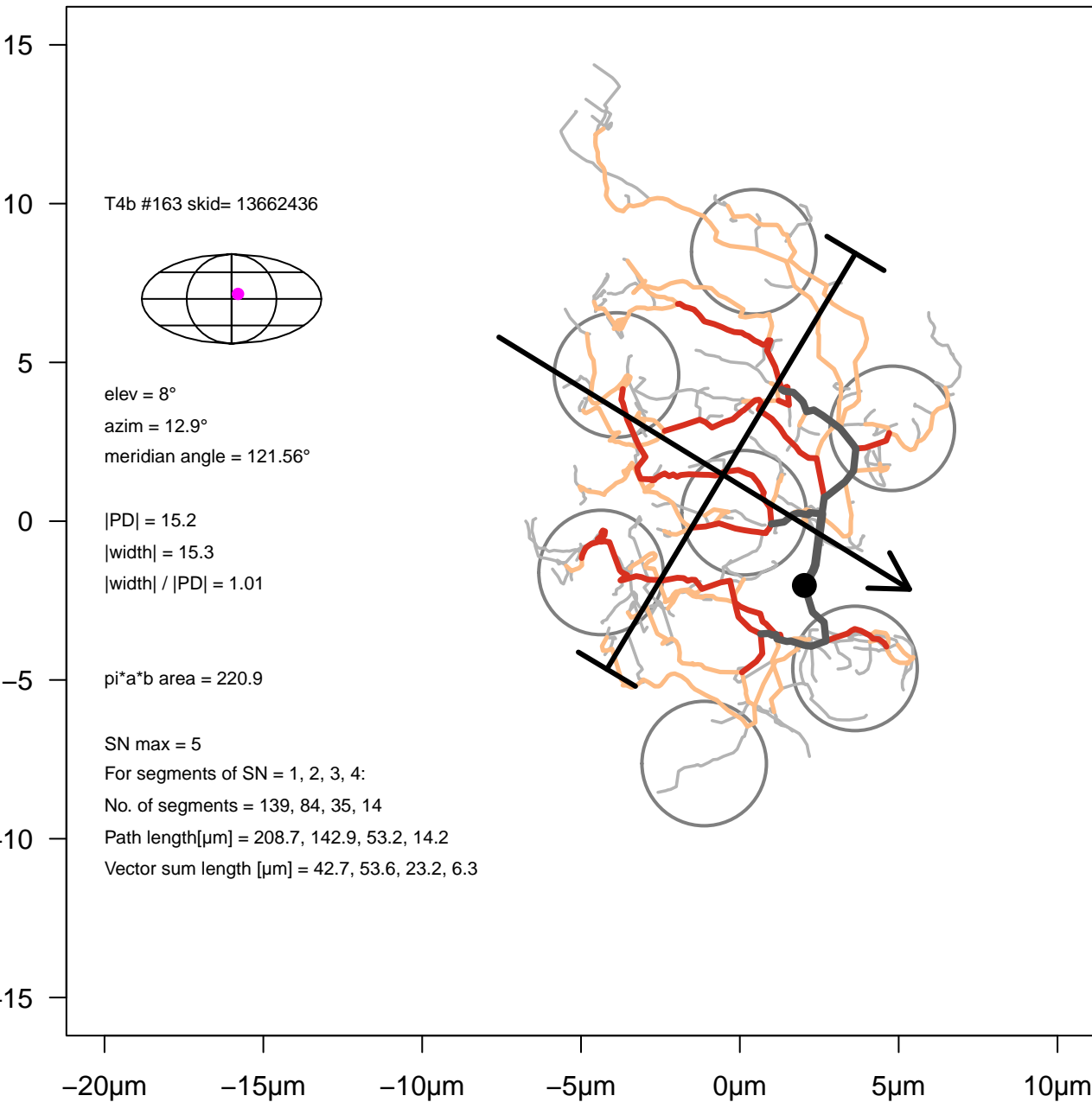

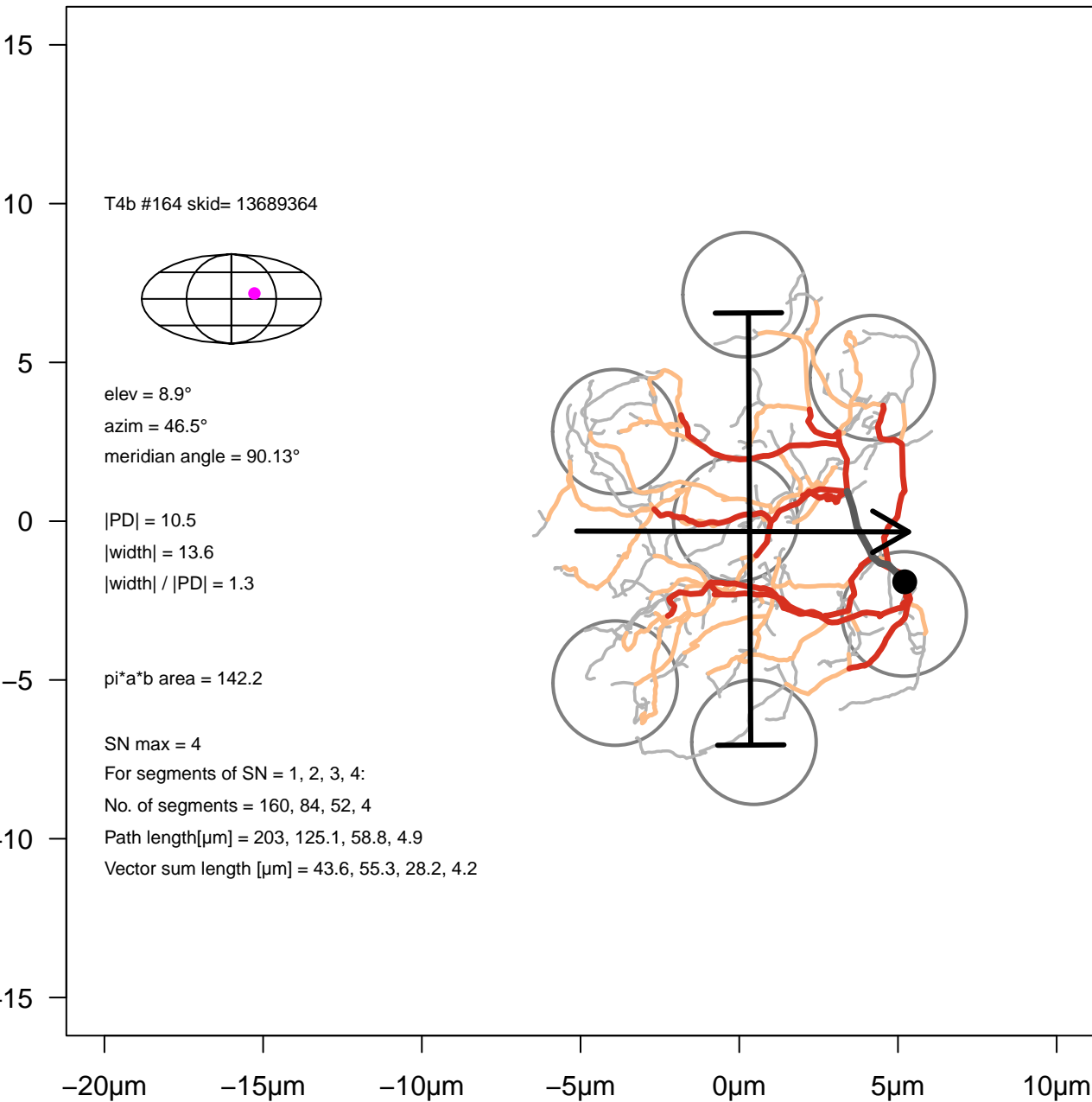

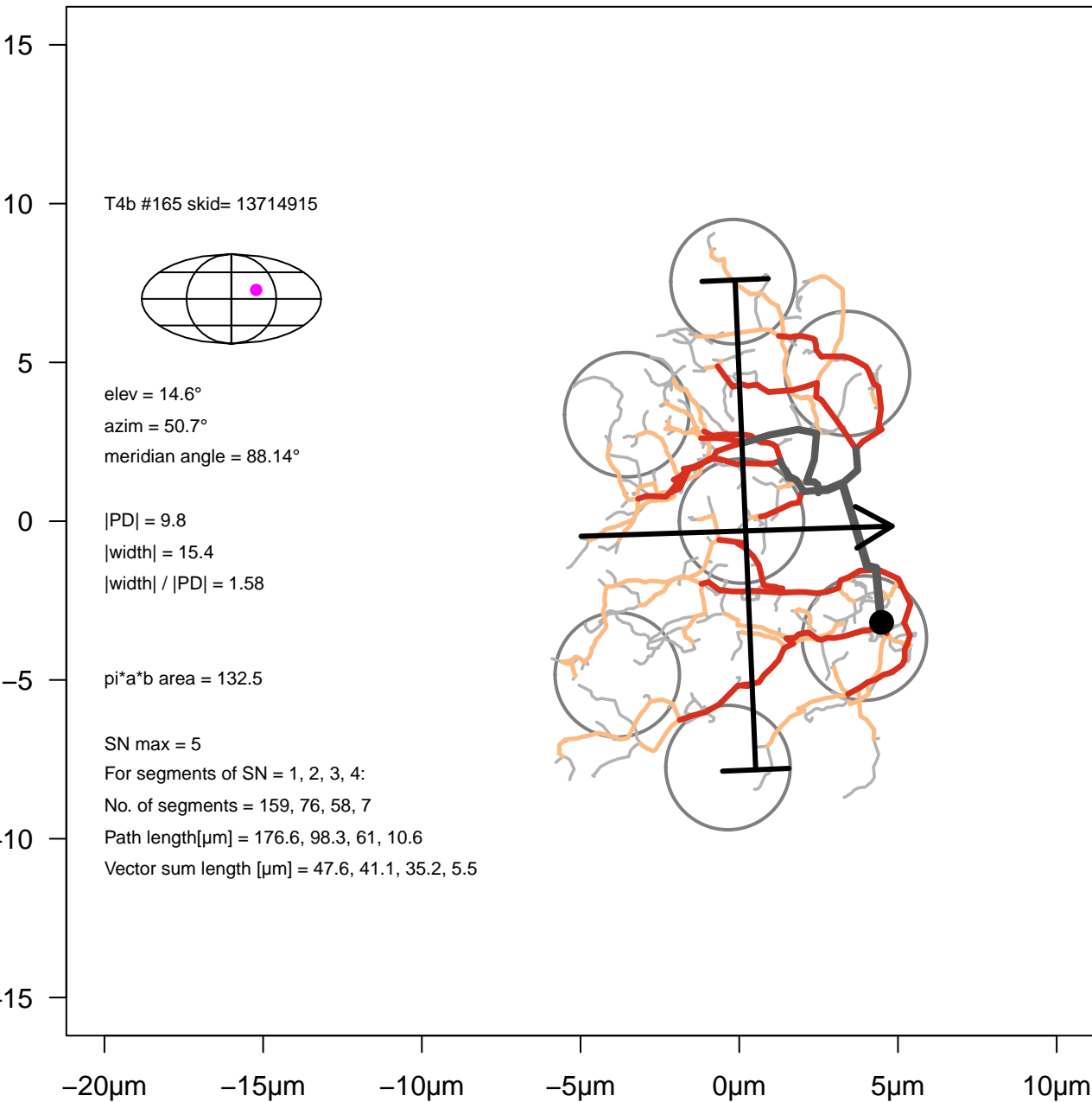

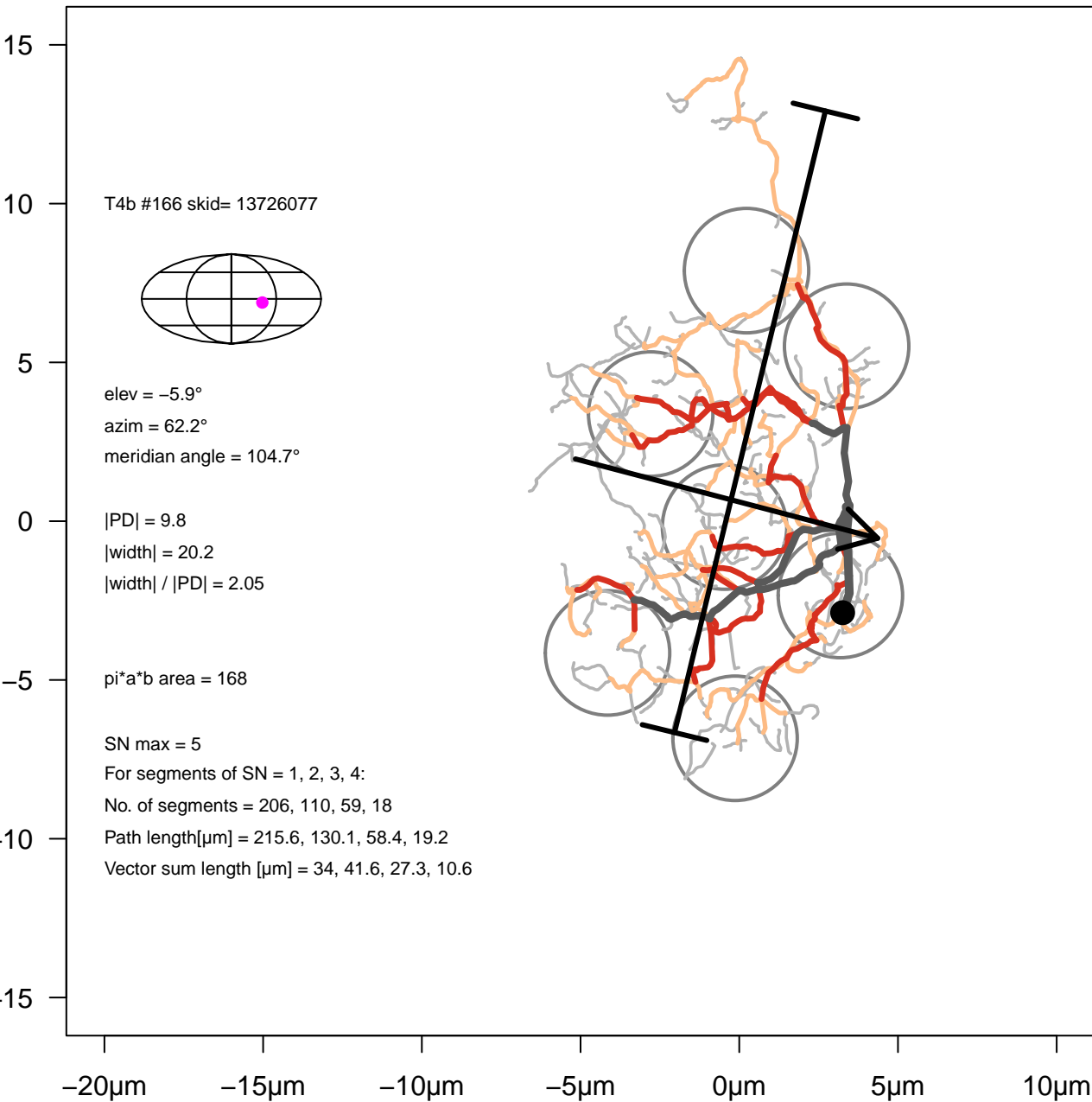

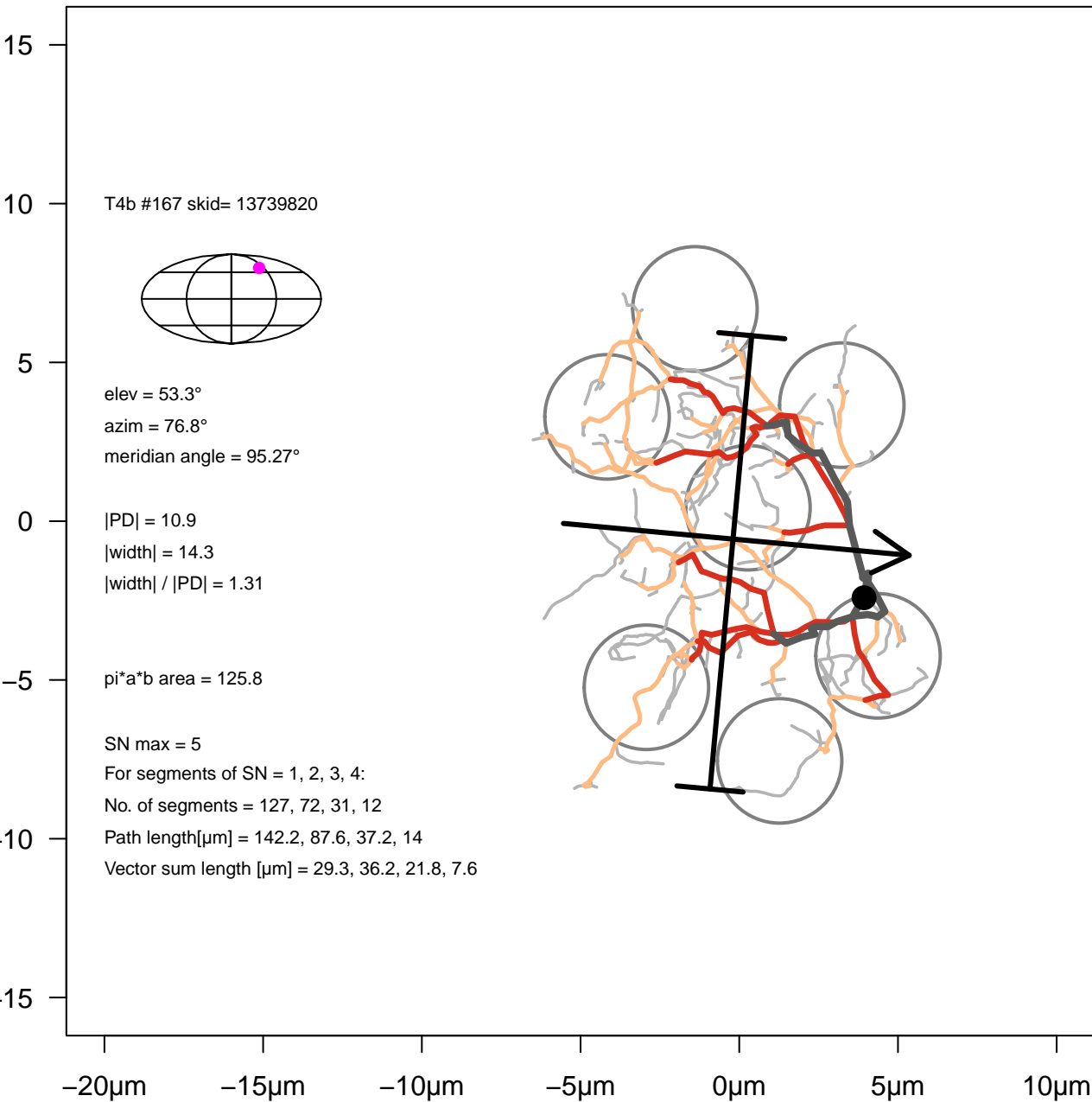

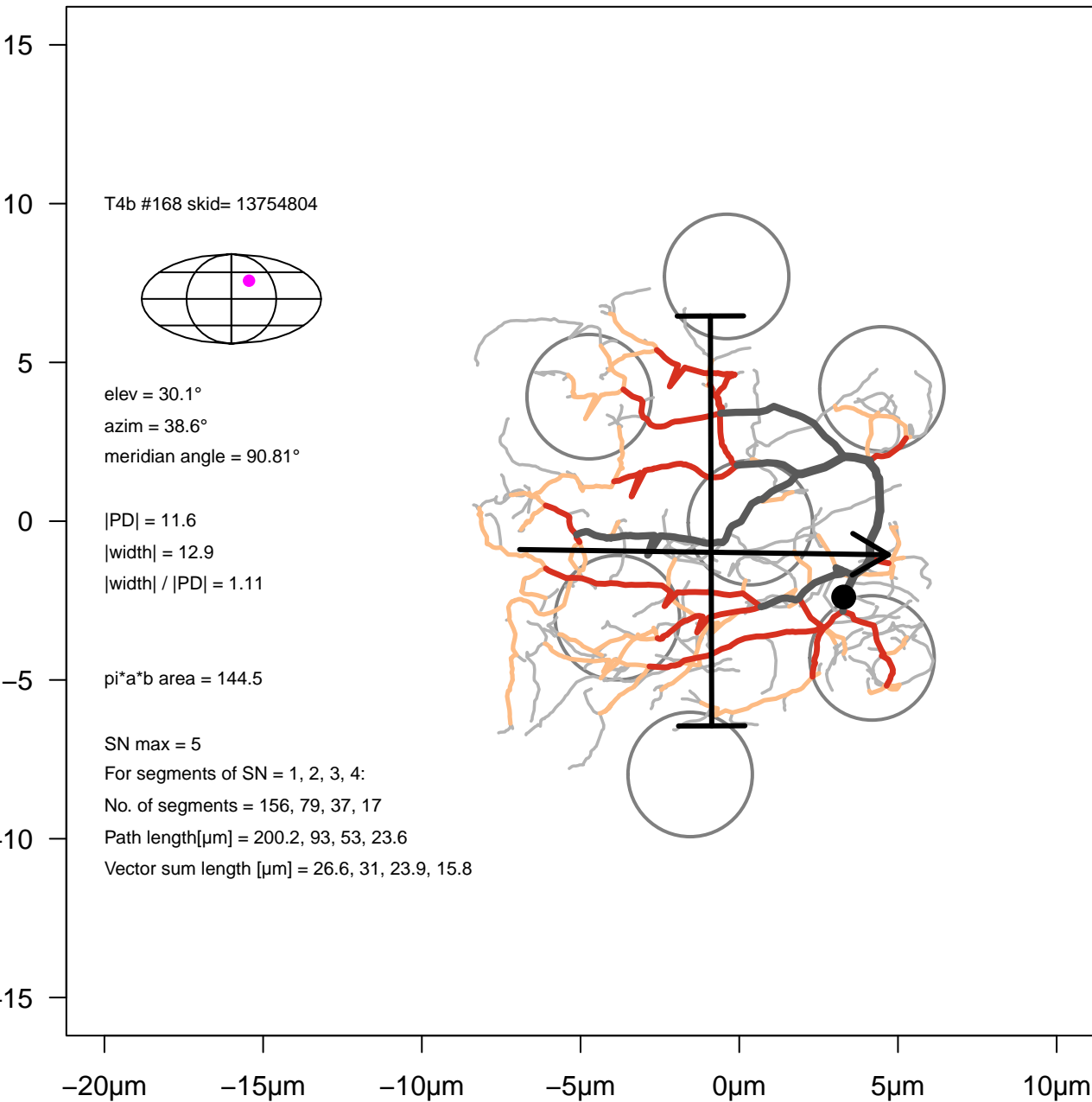

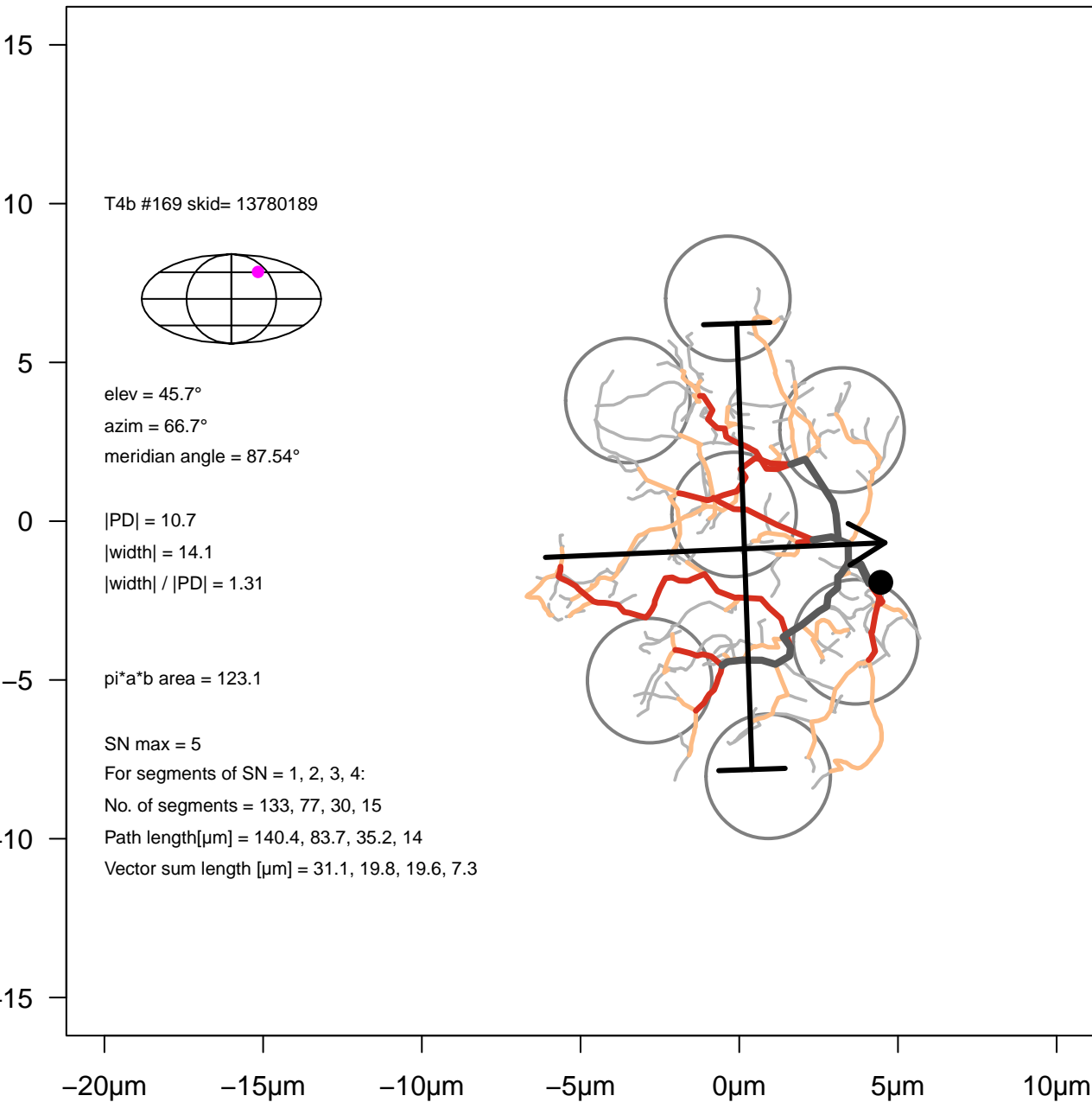

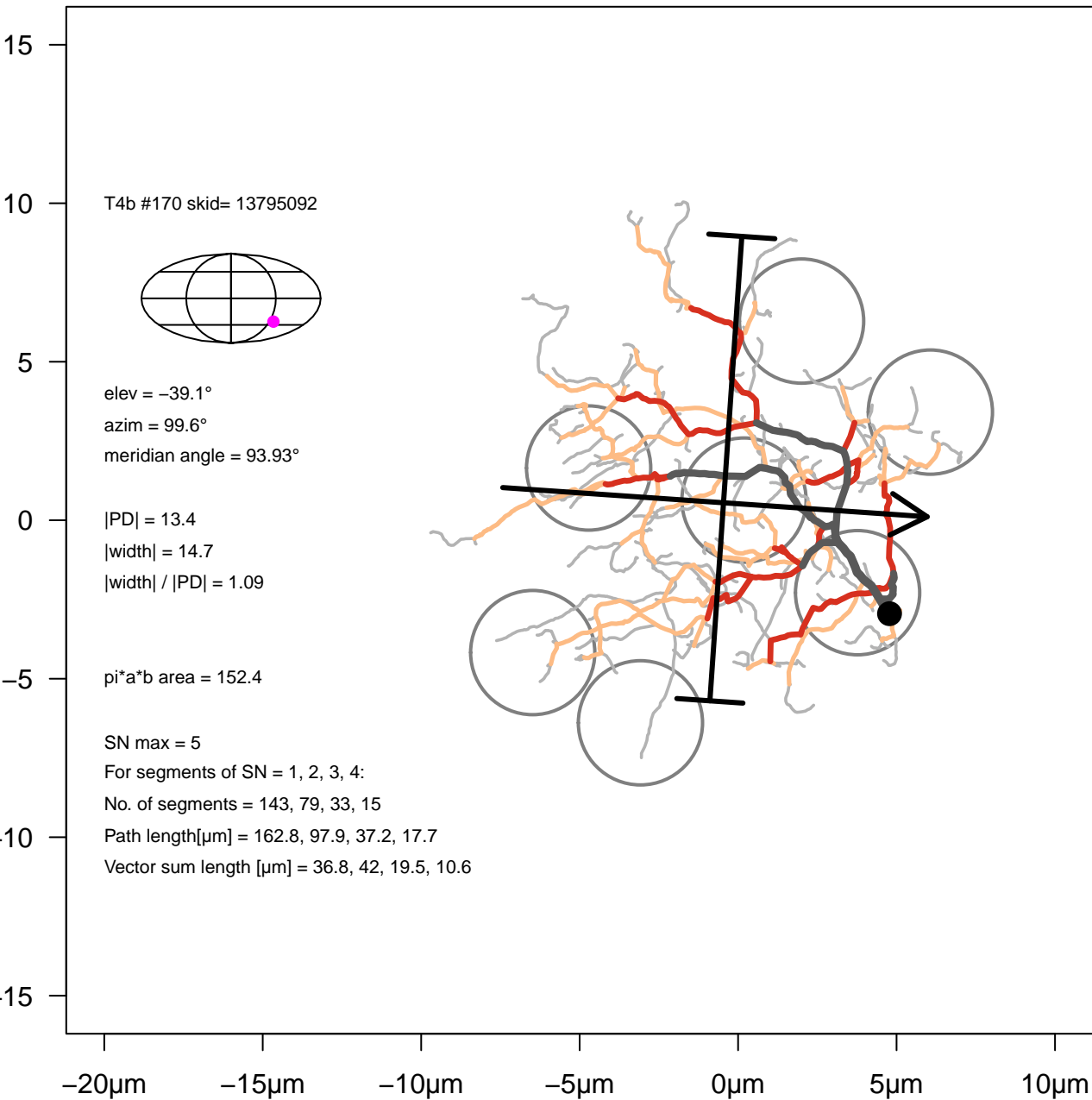

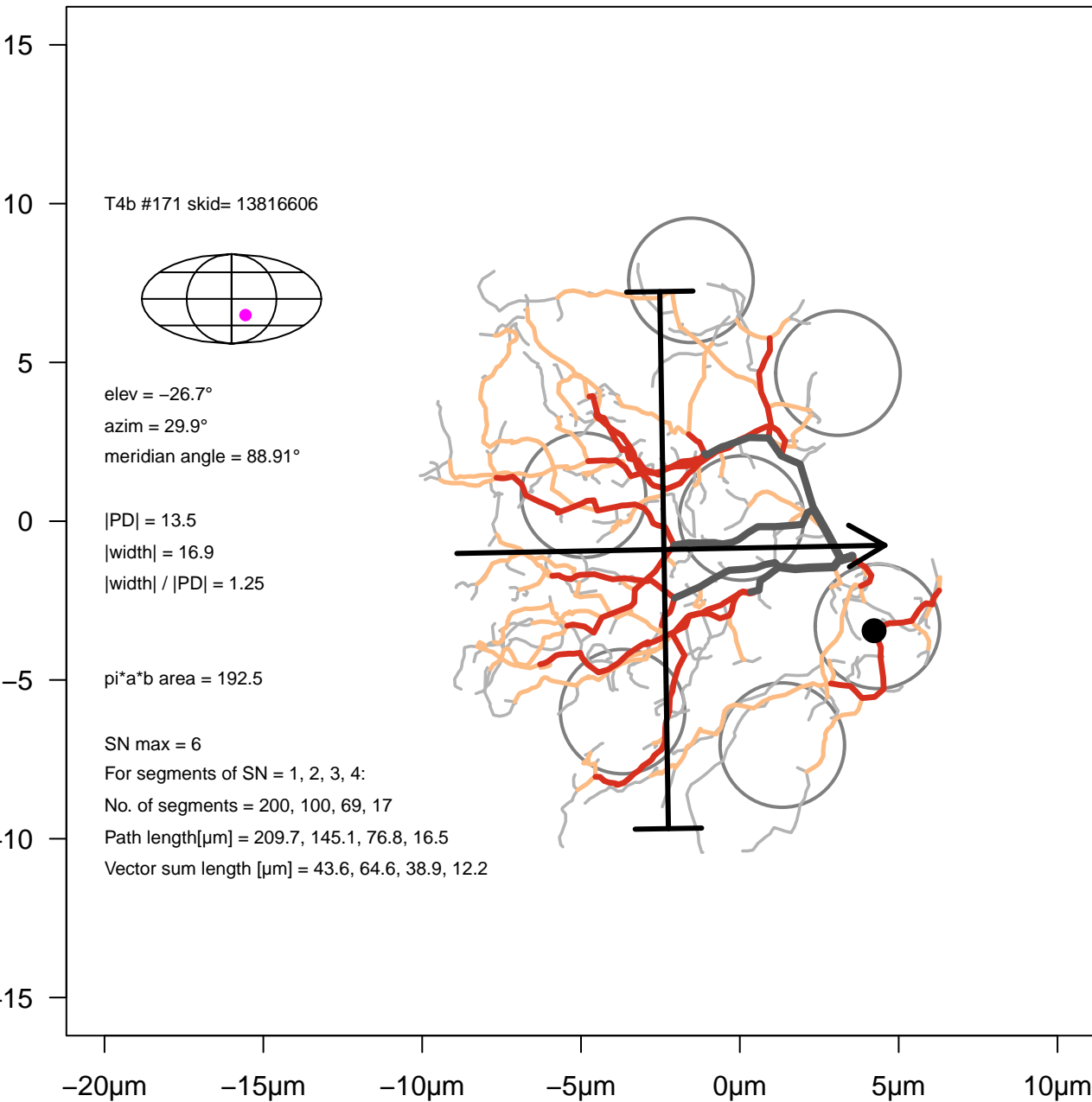

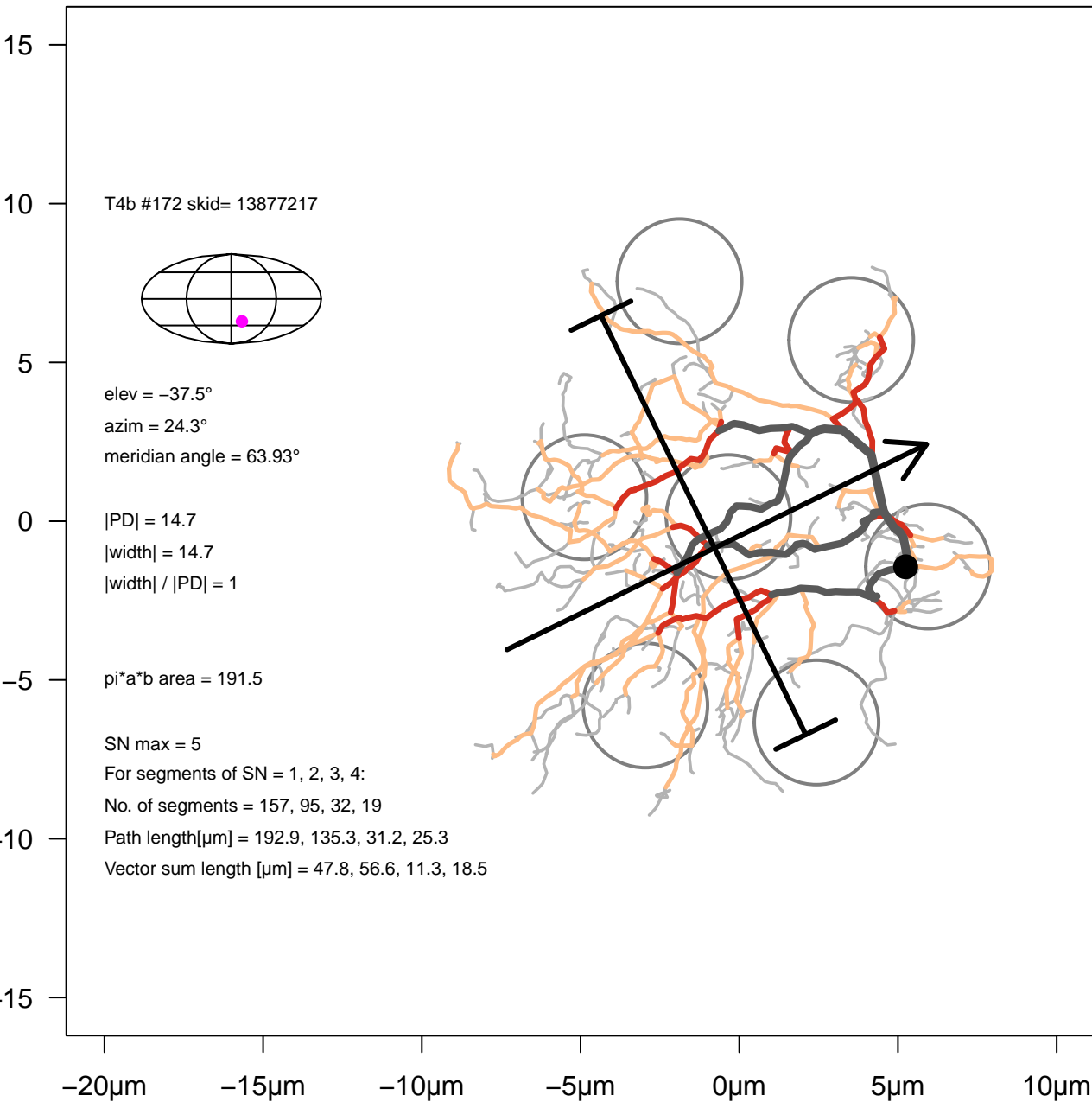

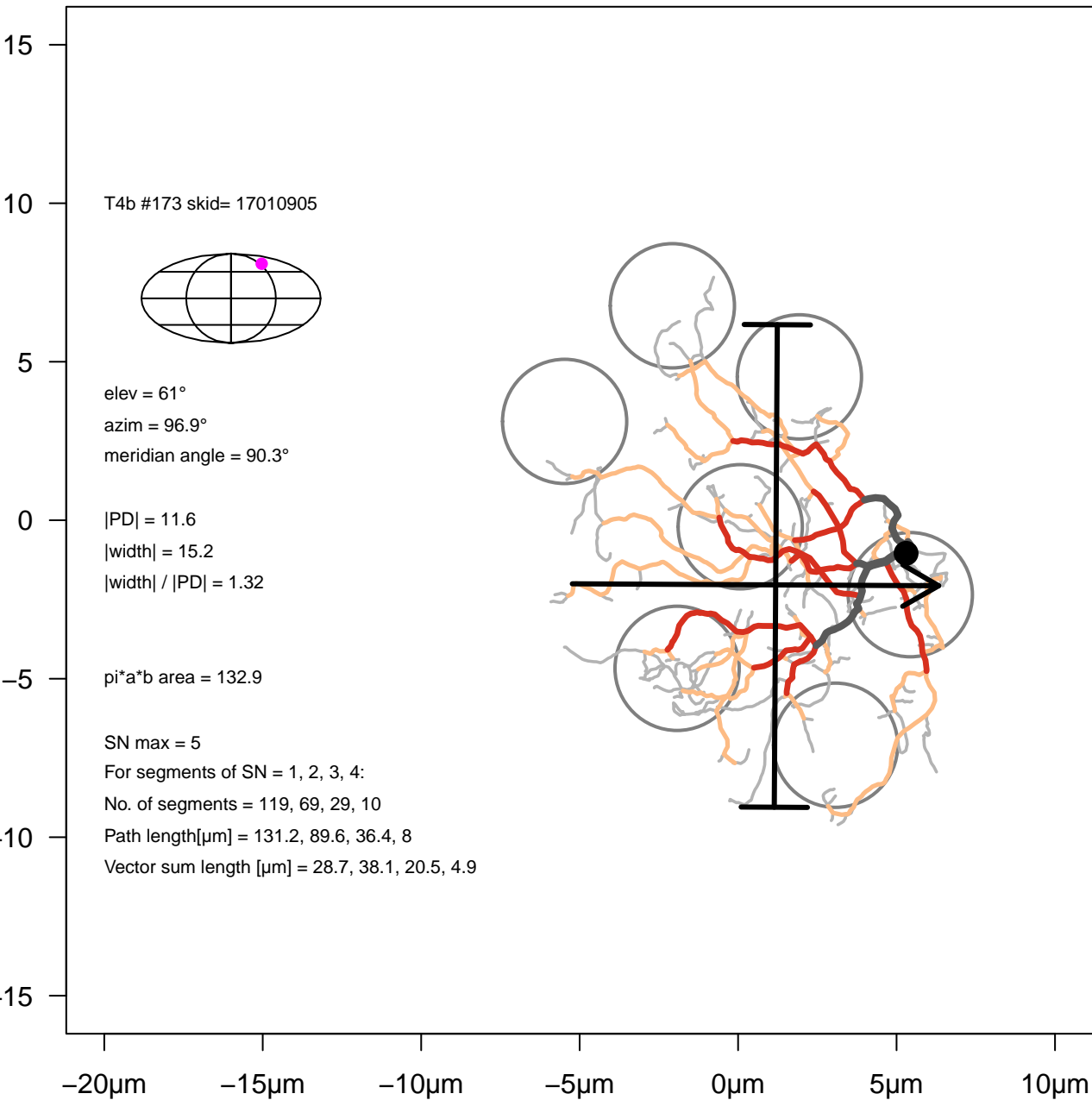

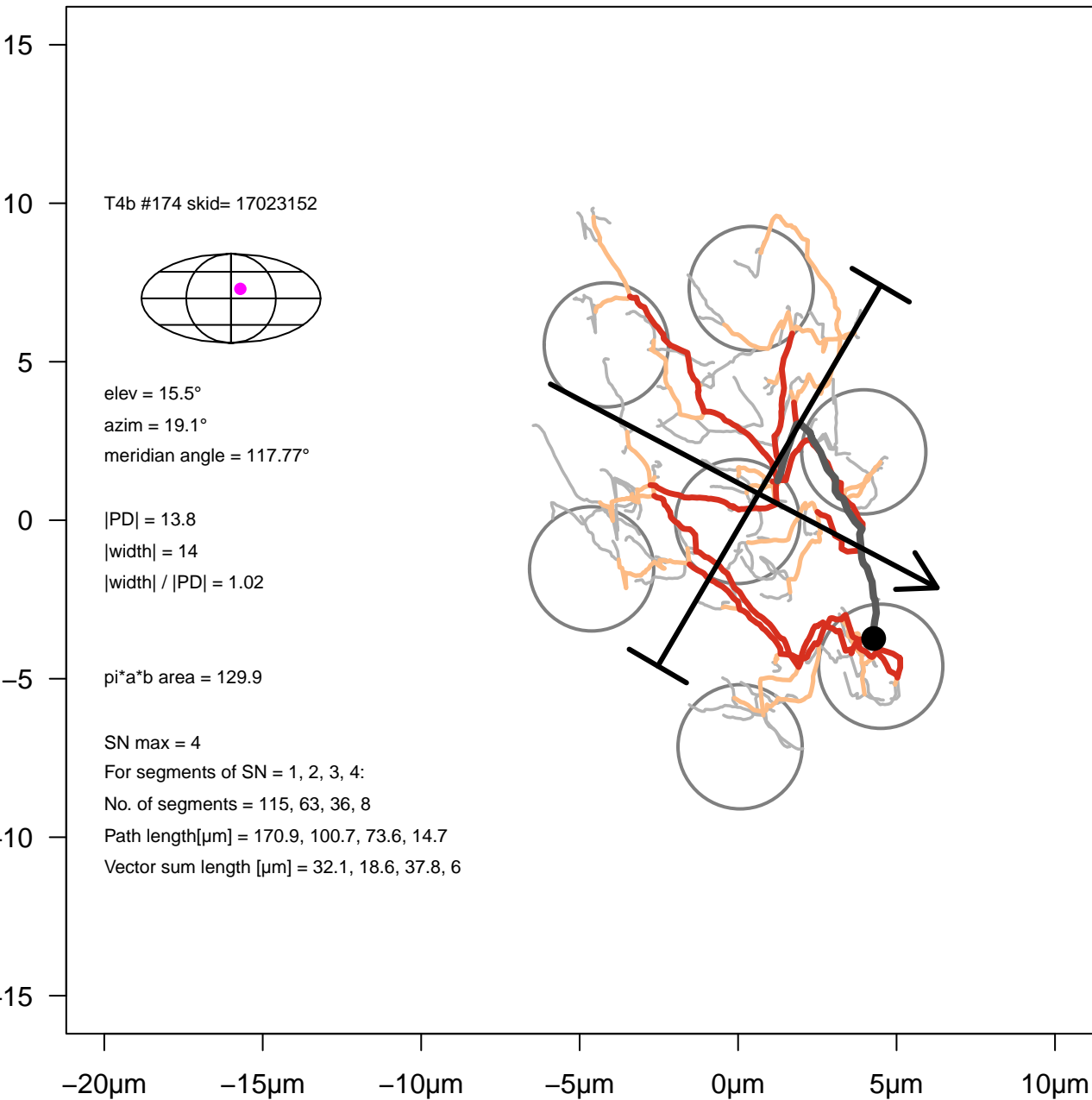

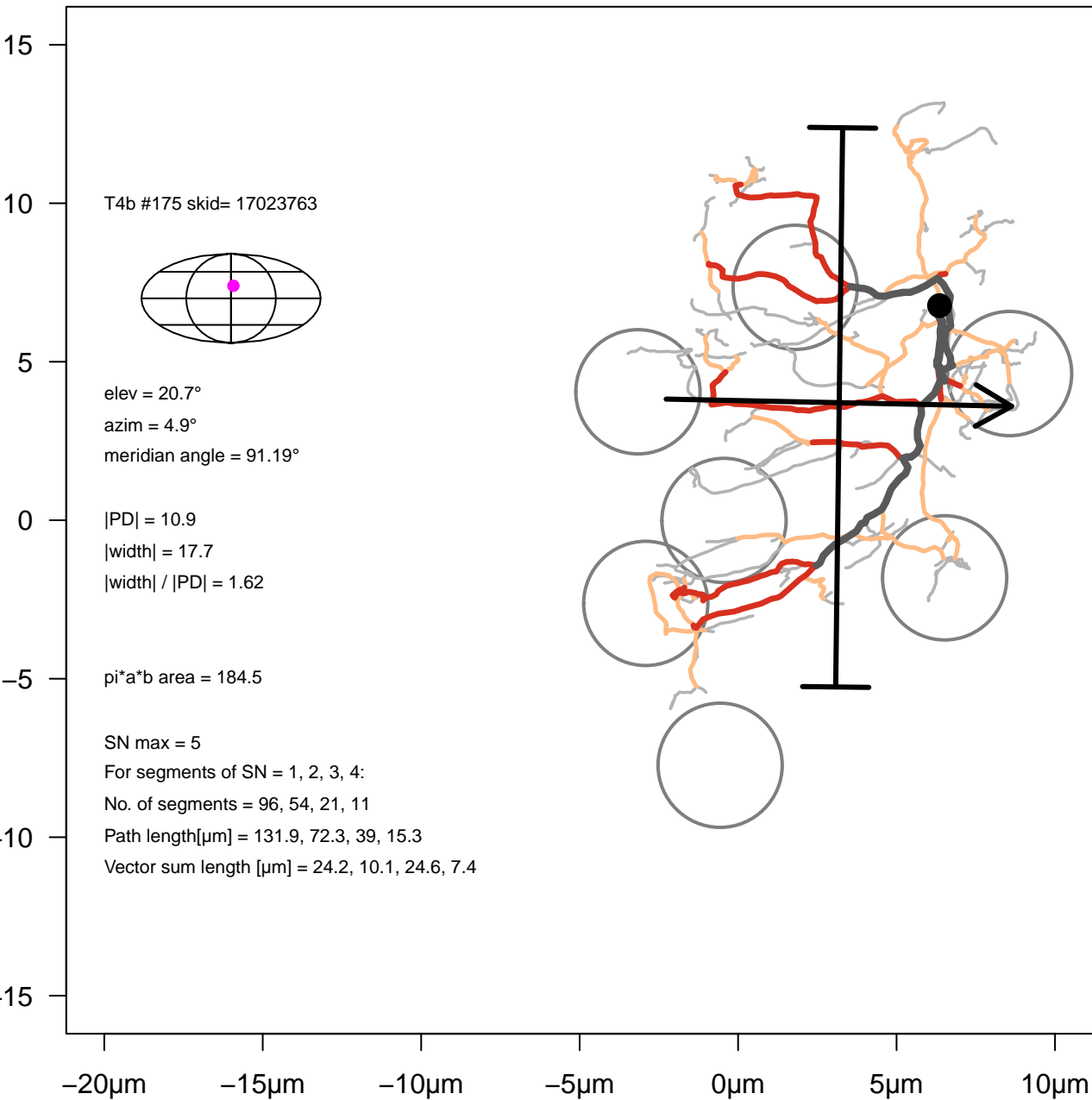

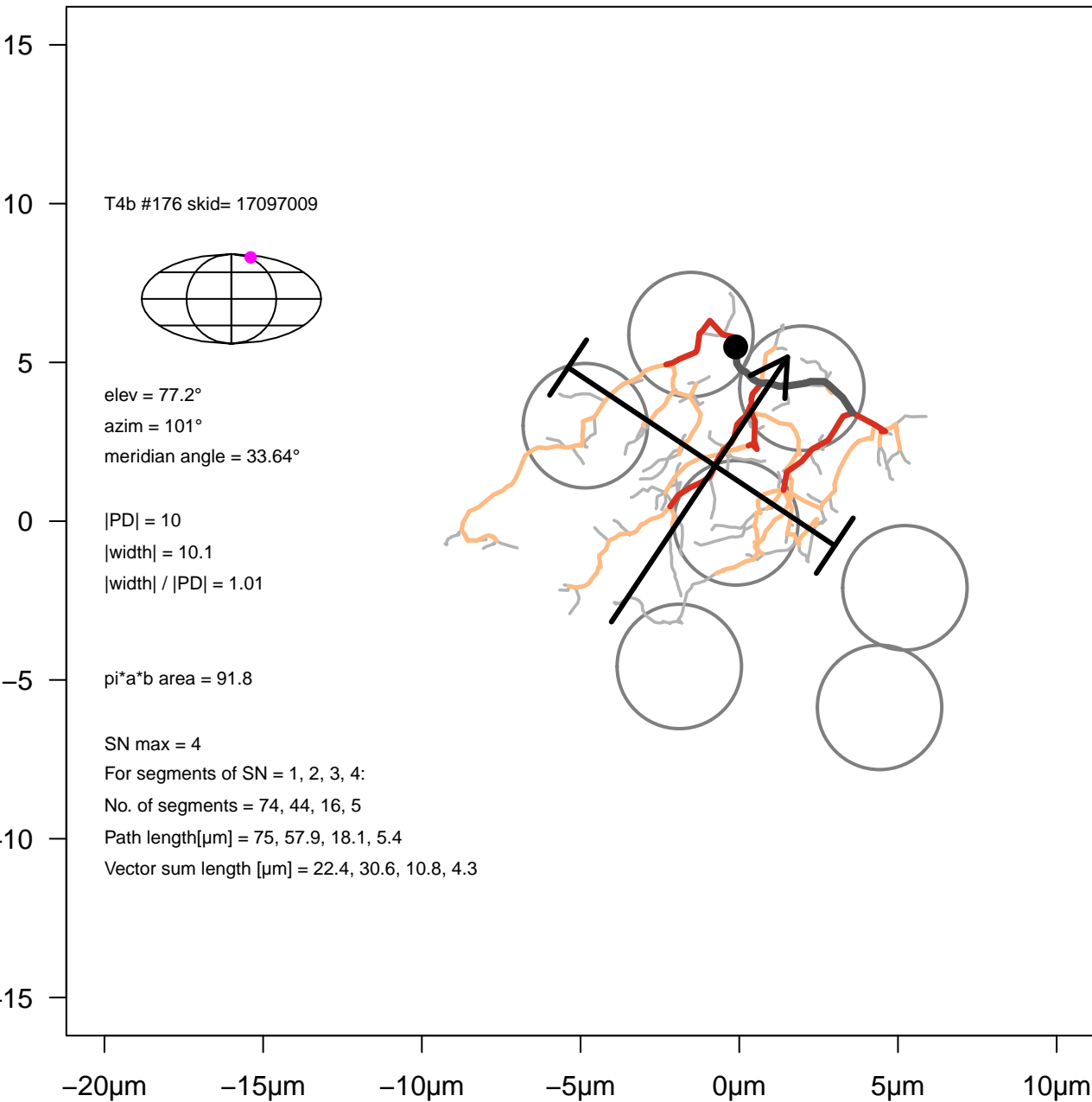

Supplement: Supplementary file 3 — Galleries of T4 neurons with PDs. All T4 neurons reconstructed in the FAFB dataset: 38 T4a, 176 T4b, 22 T4c, 114 T4d, are plotted similarly as in Fig. 2g. Using the eye map established in Fig. 4a, we include the position (elevation and azimuth angles) in the eye coordinate. The angle between T4’s PD and the local meridian line is computed instead of using the +v-axis as the reference, as in Fig. 2g. The meridian line is defined as the direction line going from the south pole to the north pole in the eye reference frame (often close to the +v-axis). The cell and surrounding columns are also aligned such that the vertical direction in the plot coincides with the meridian direction. A summary of the Strahler number analysis for each cell is included. [file 41586_2025_9276_MOESM3_ESM.zip › gallery_T4b.pdf]

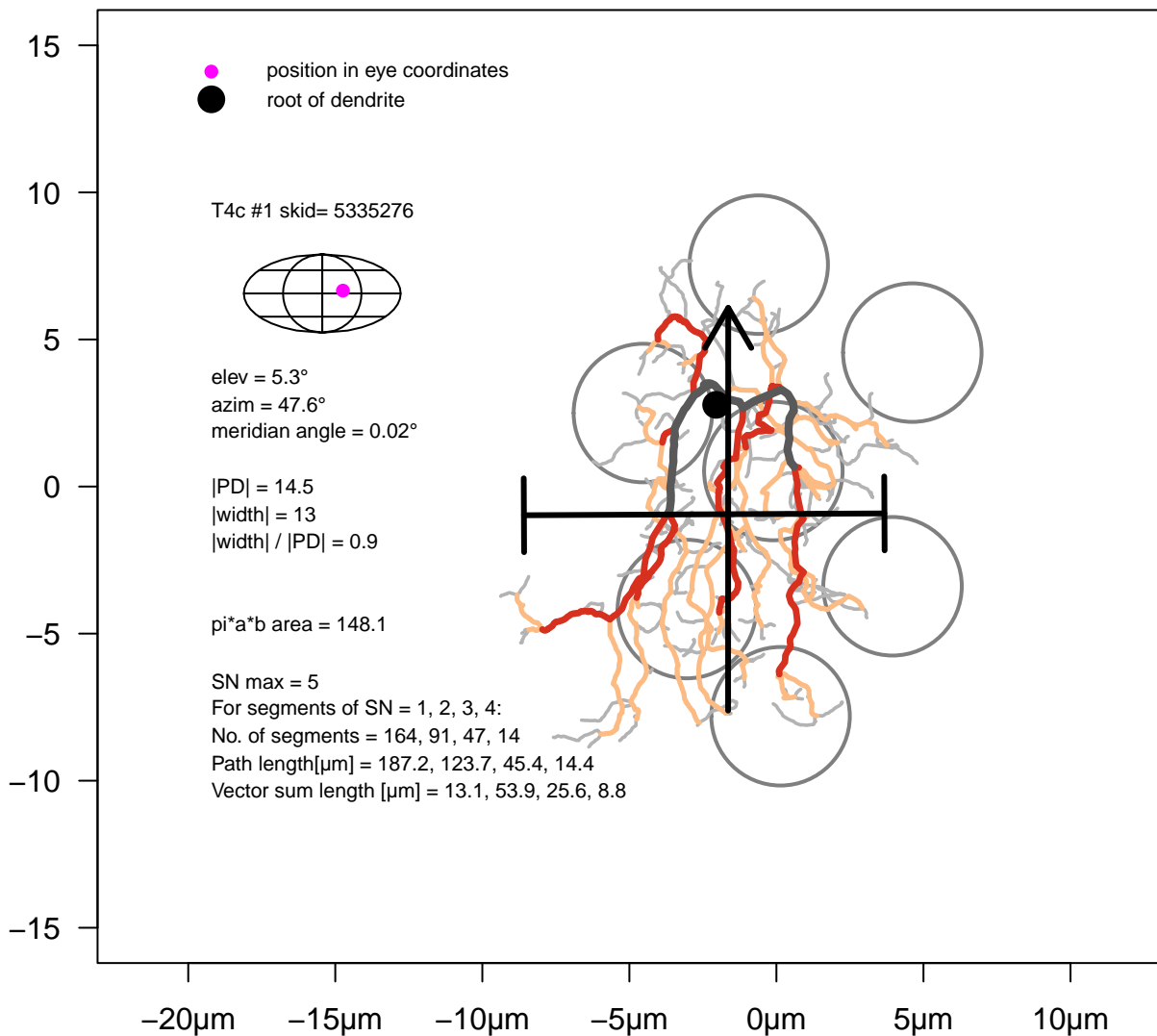

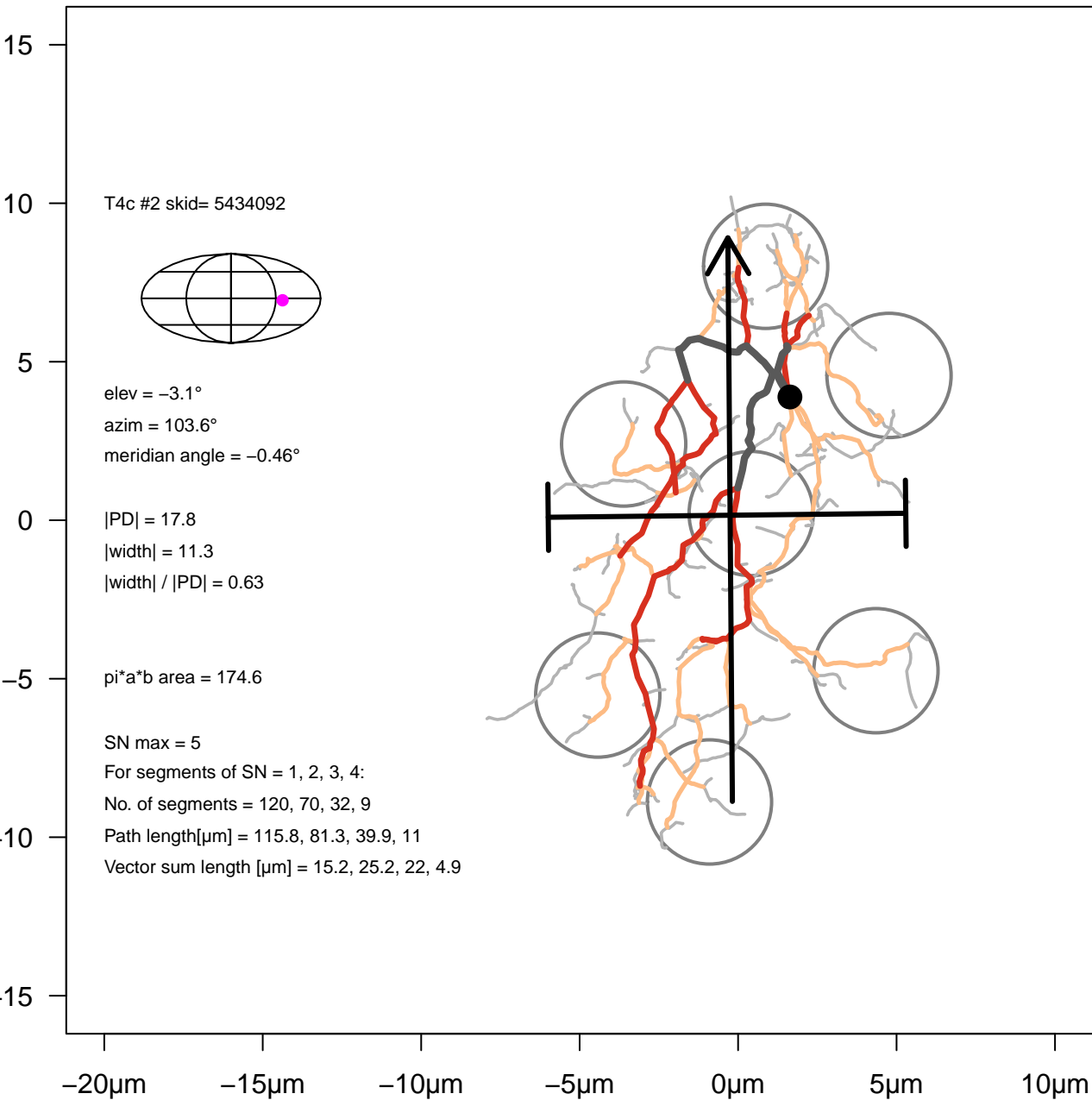

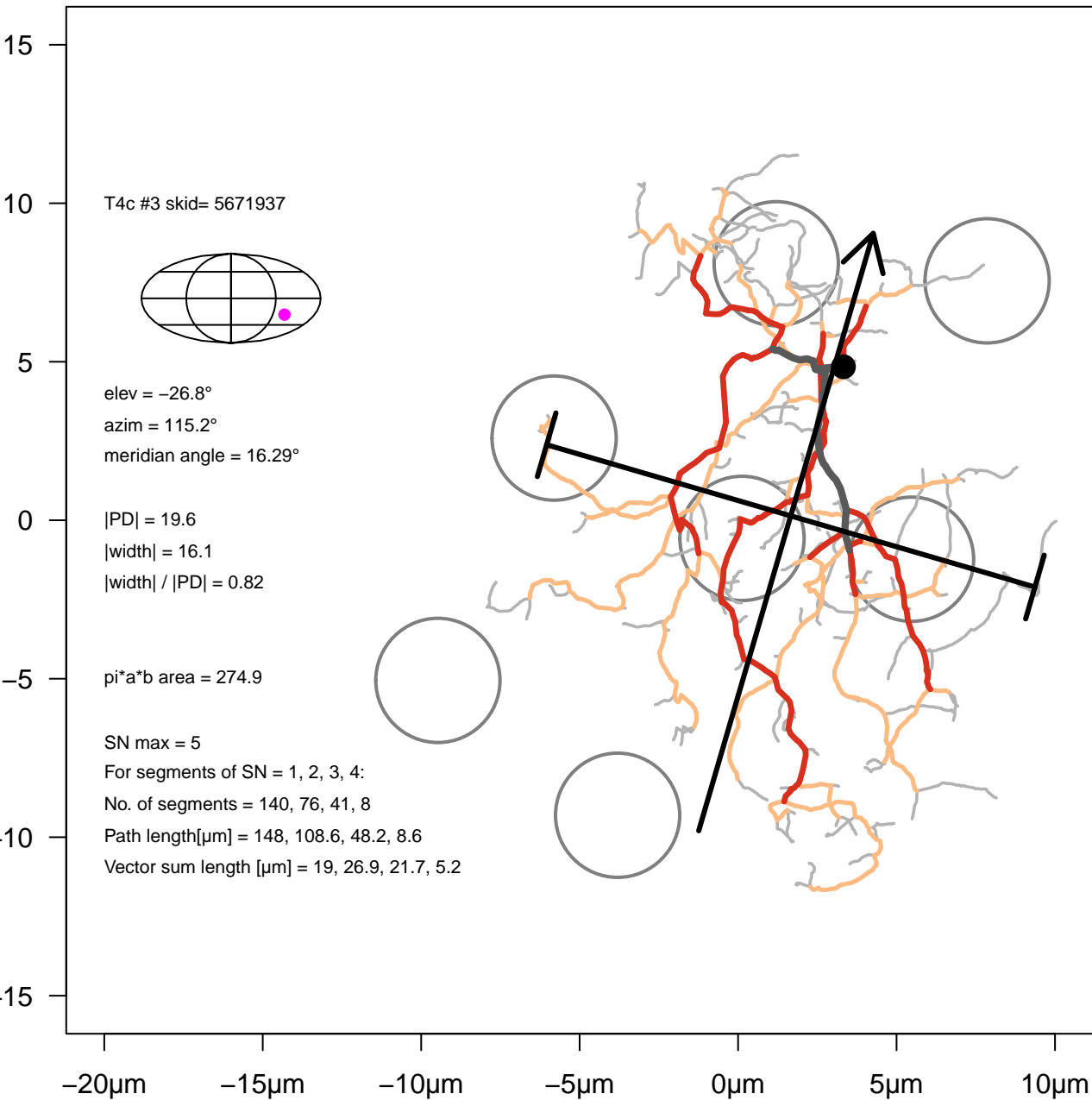

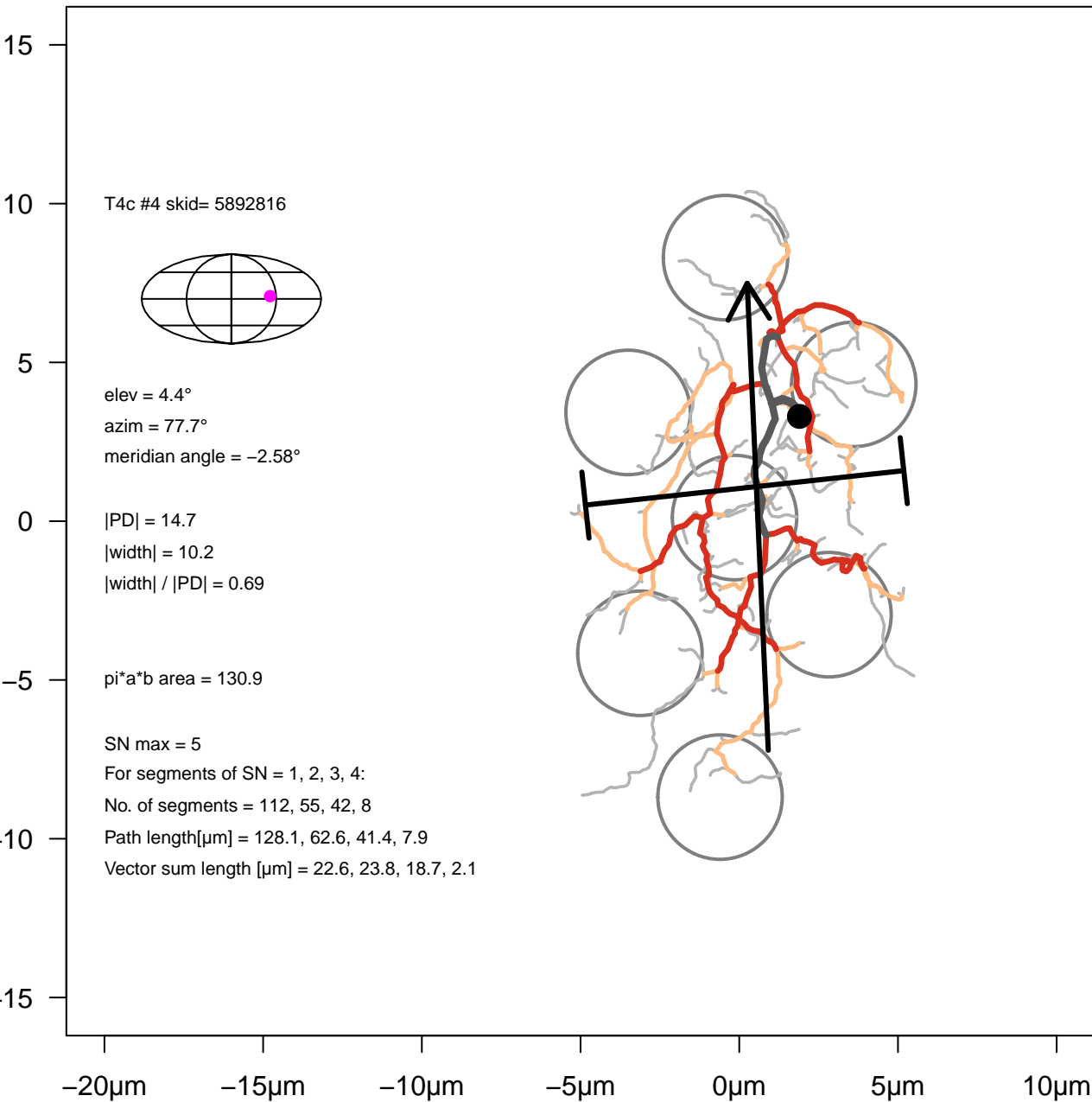

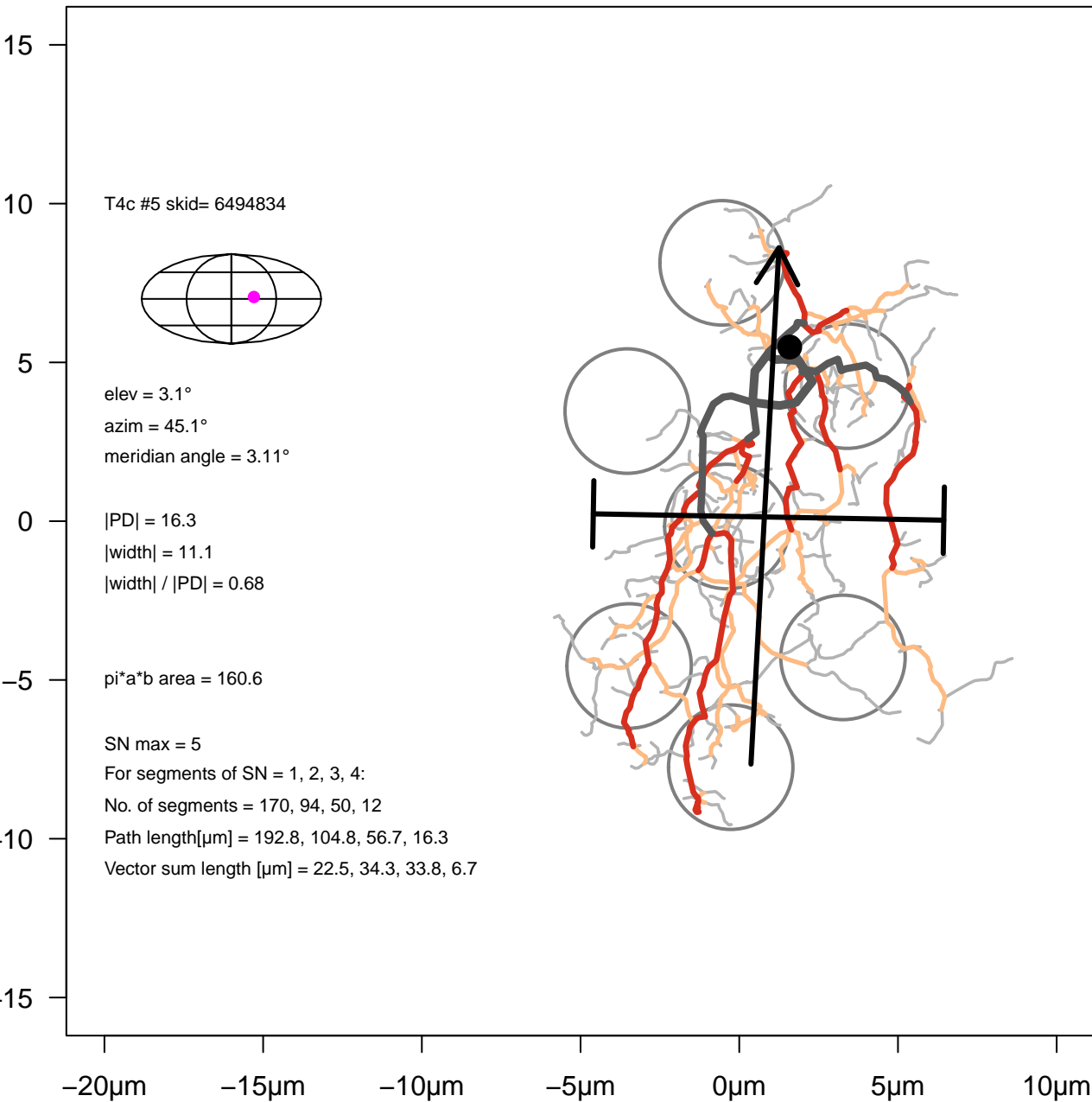

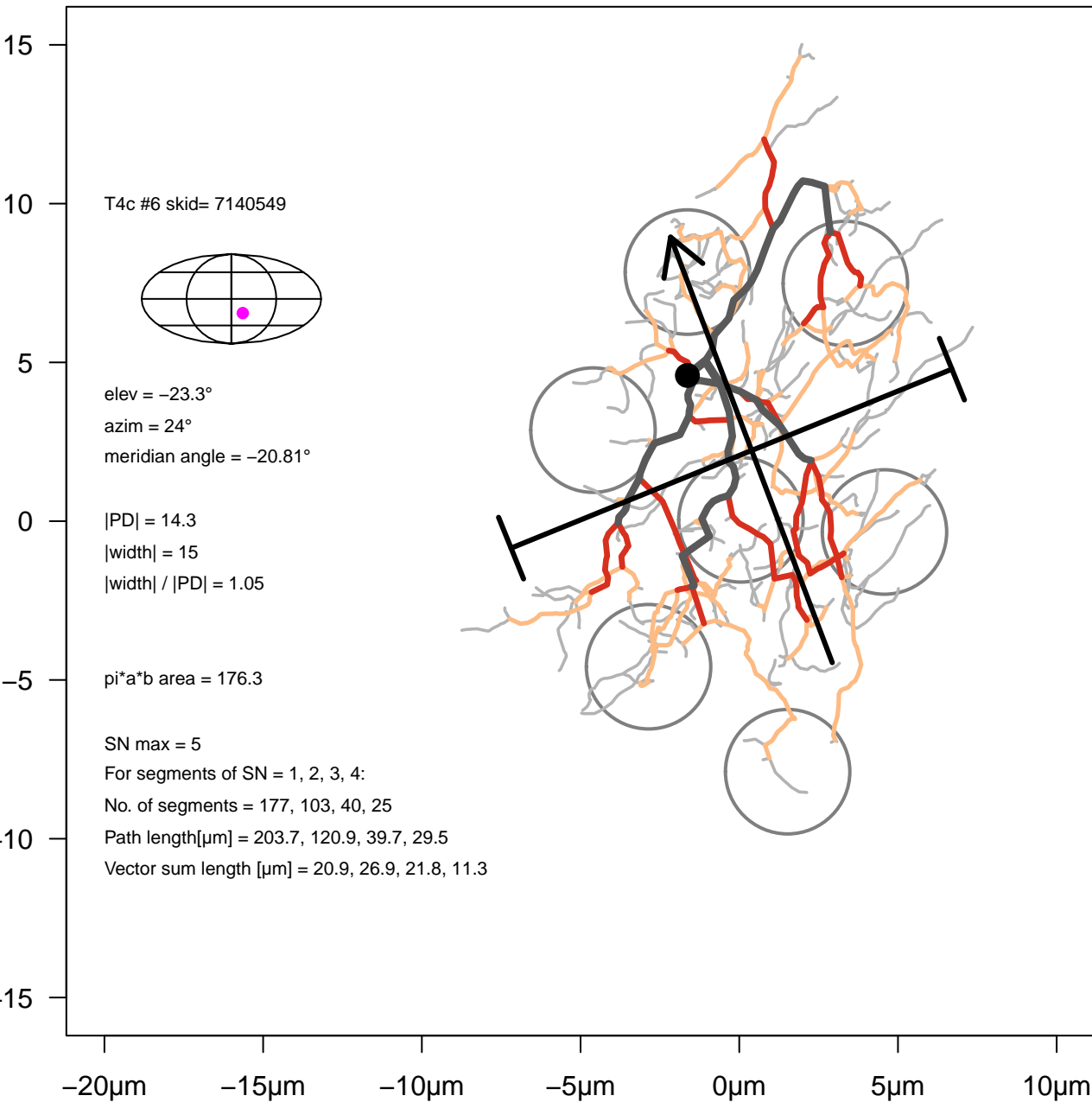

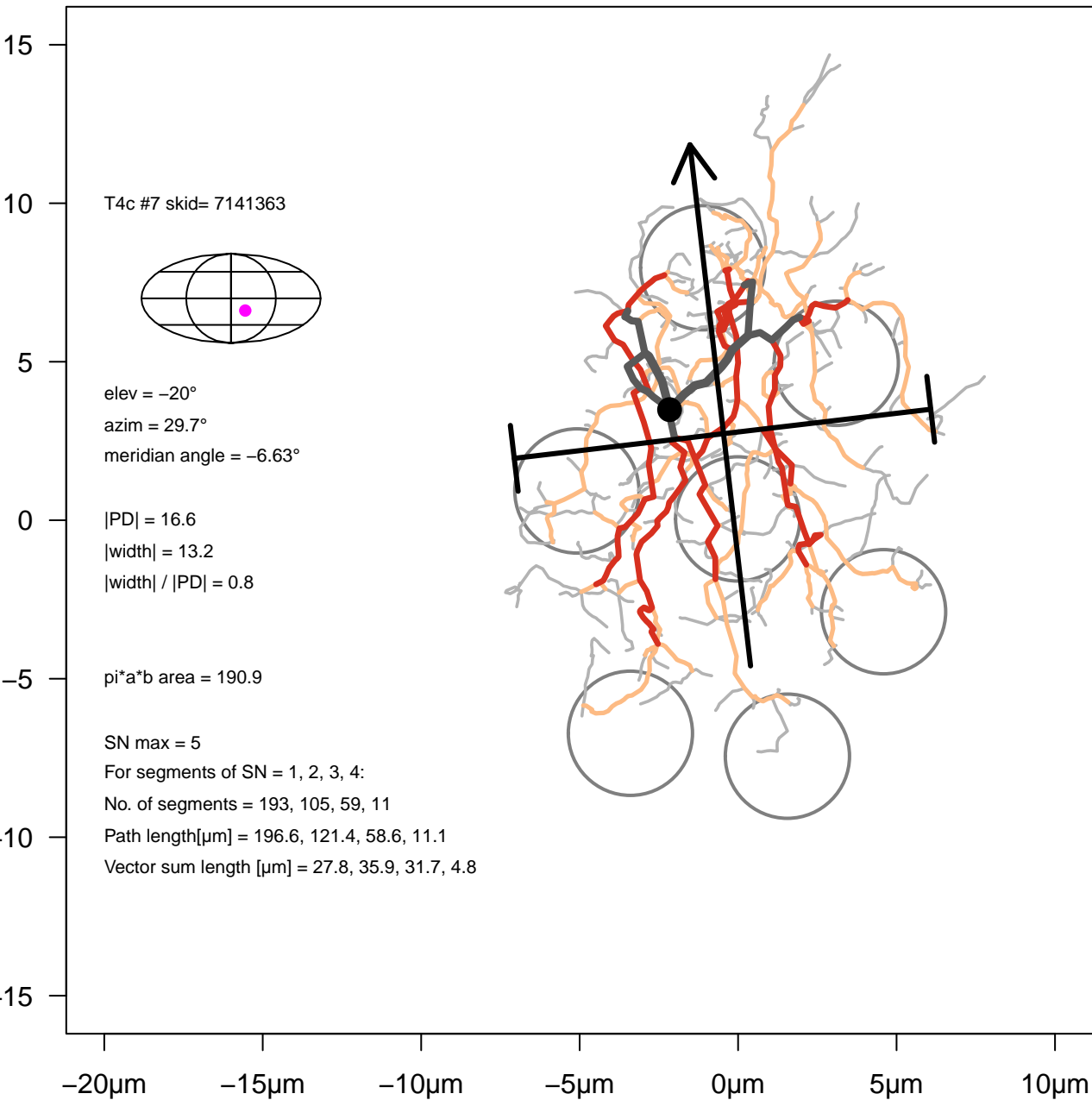

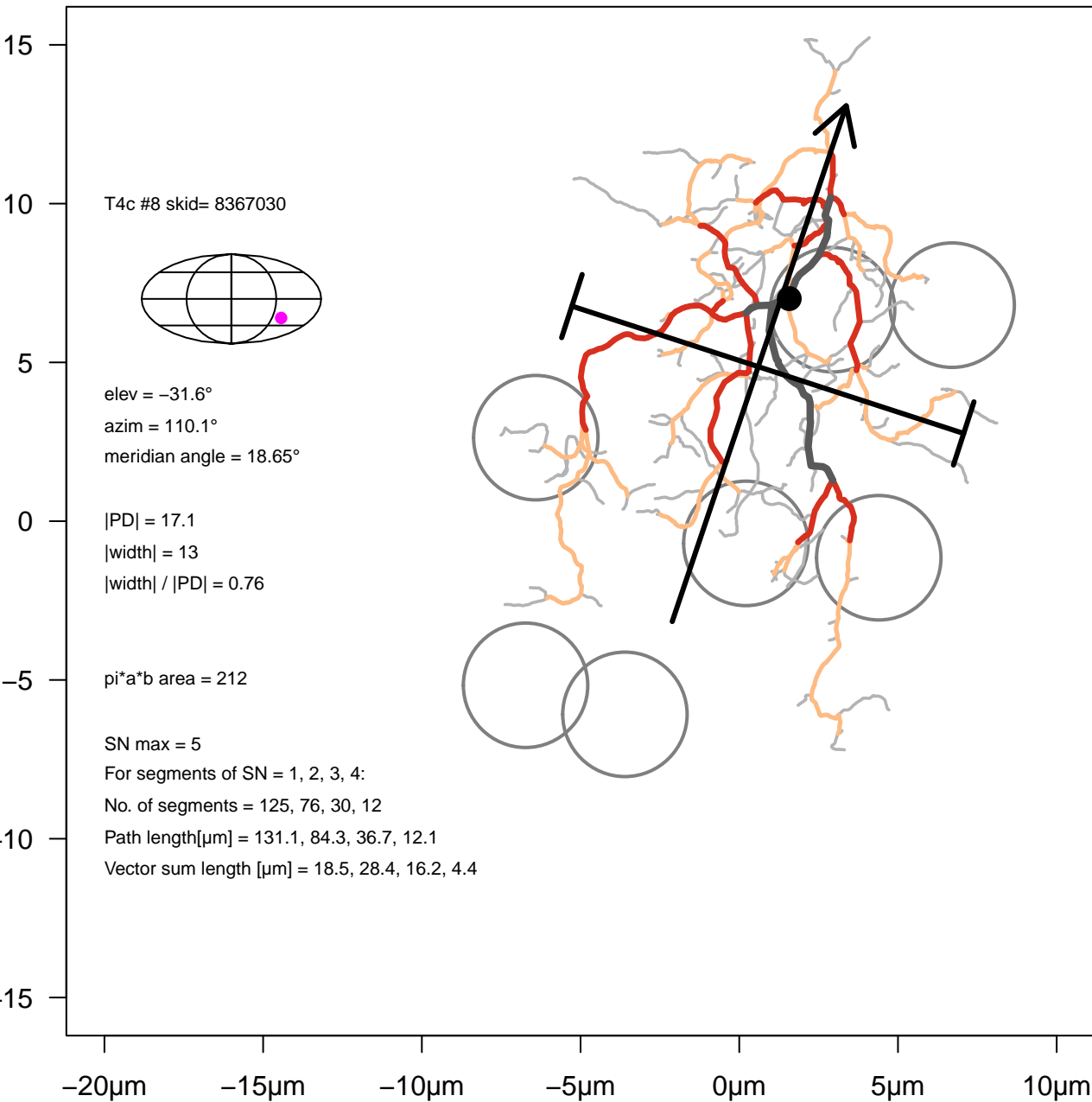

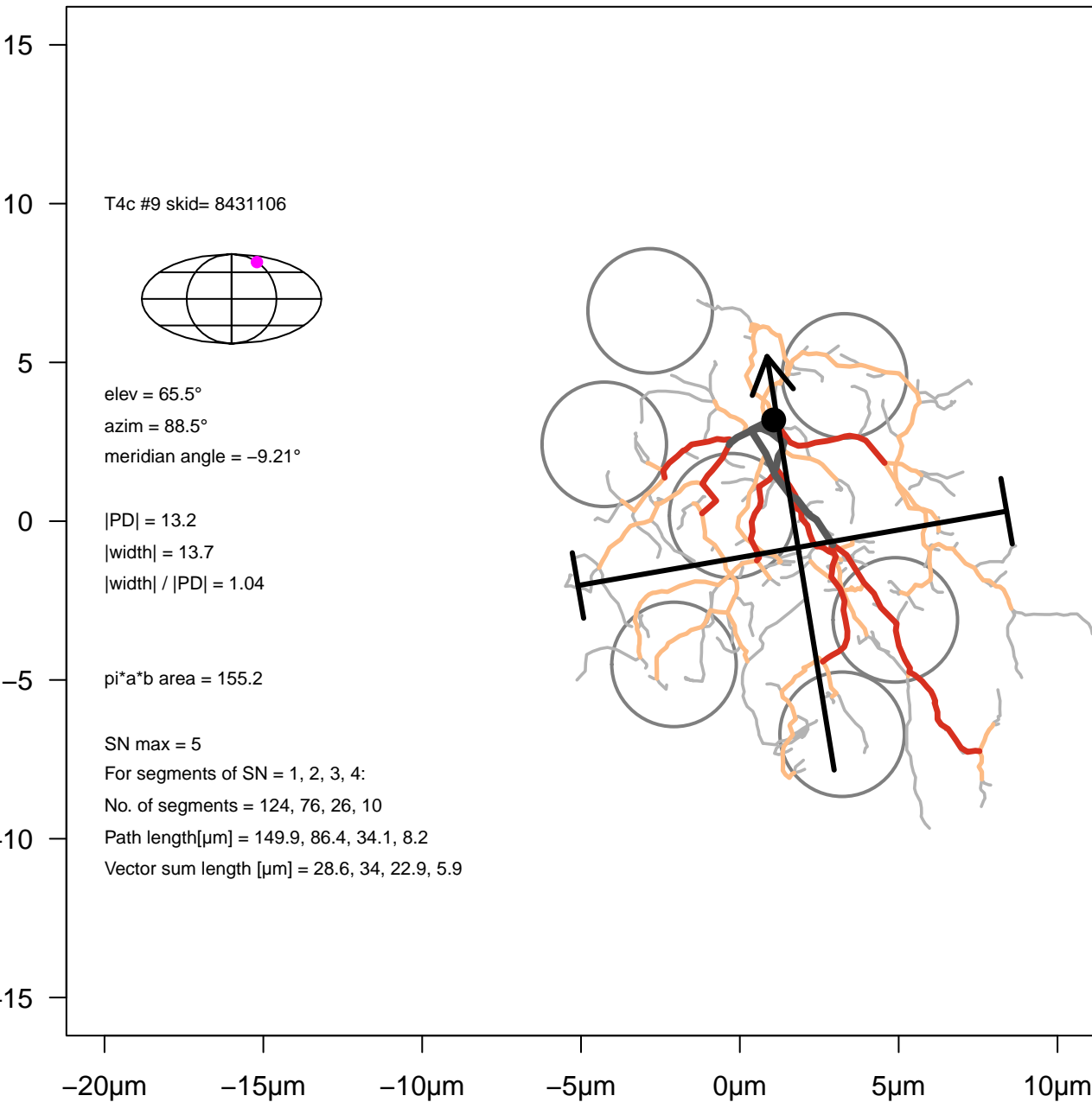

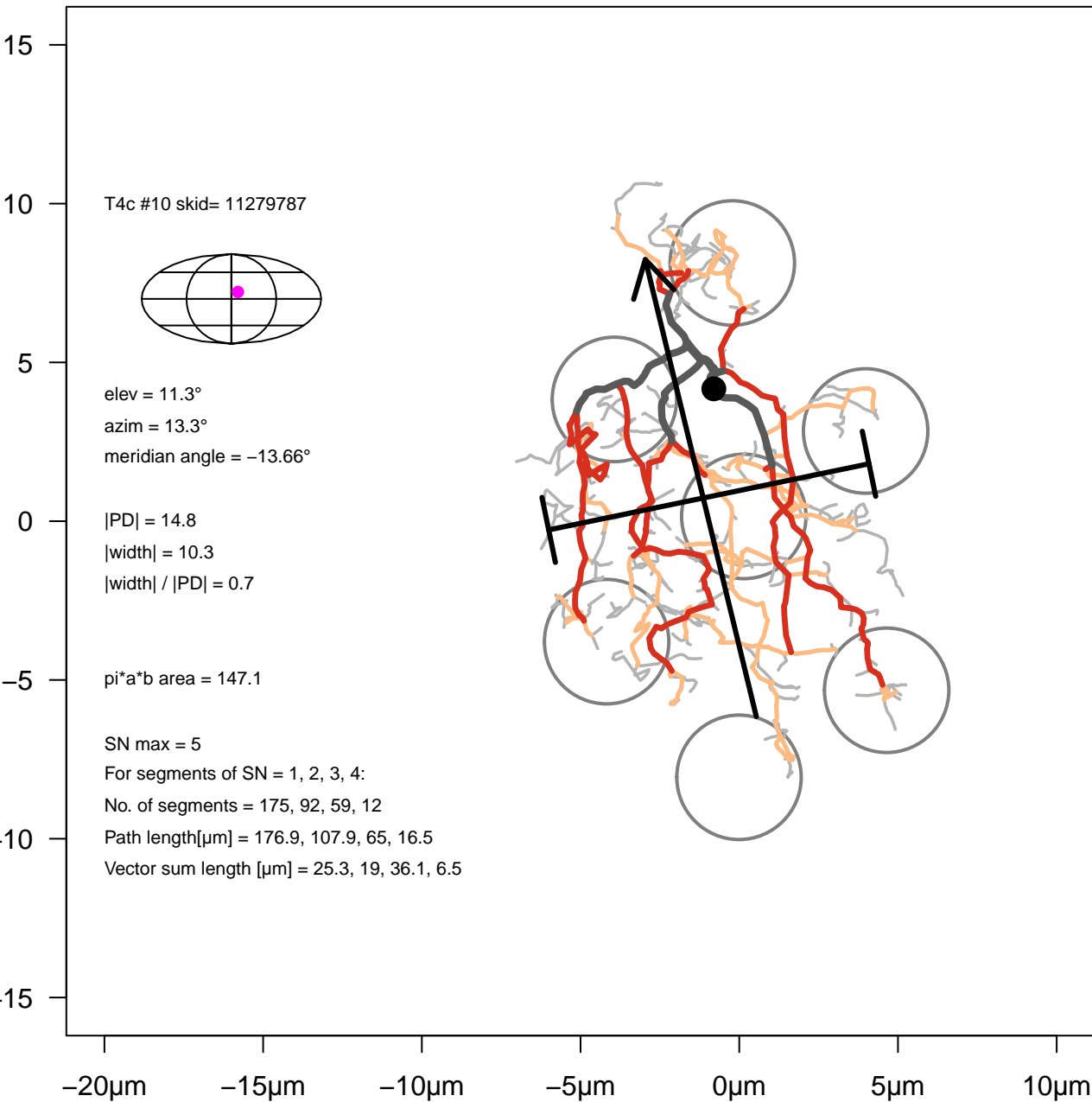

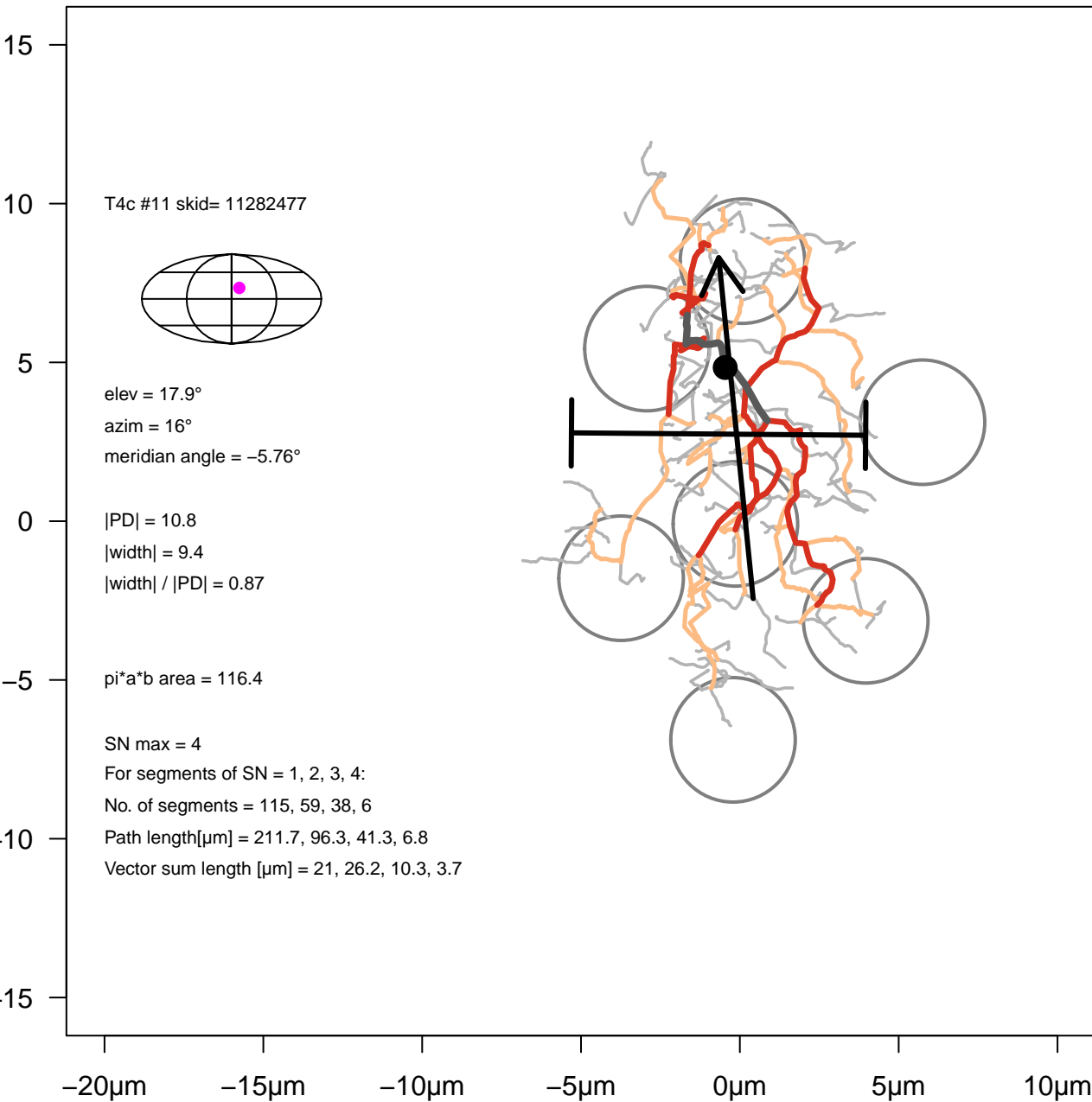

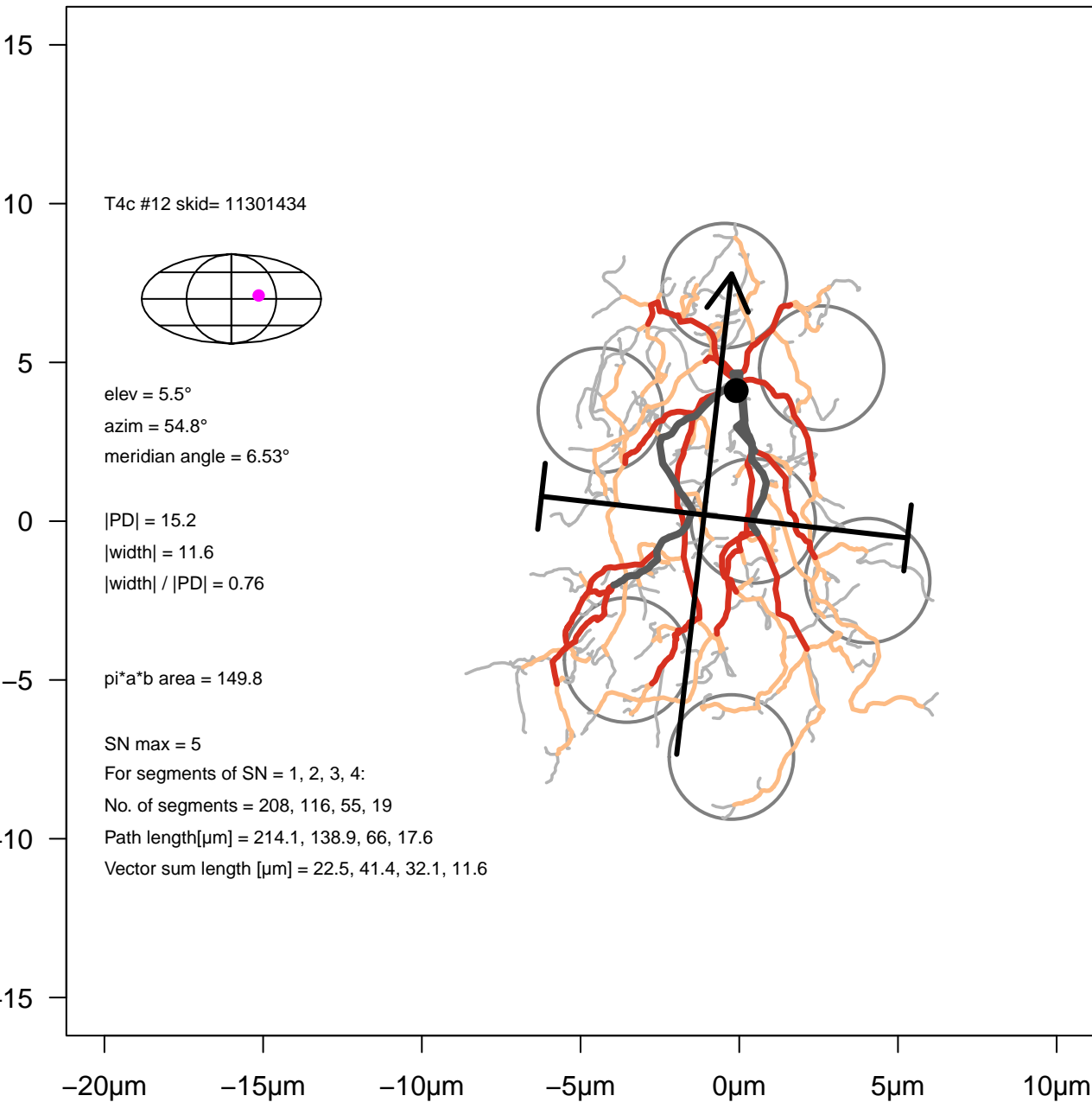

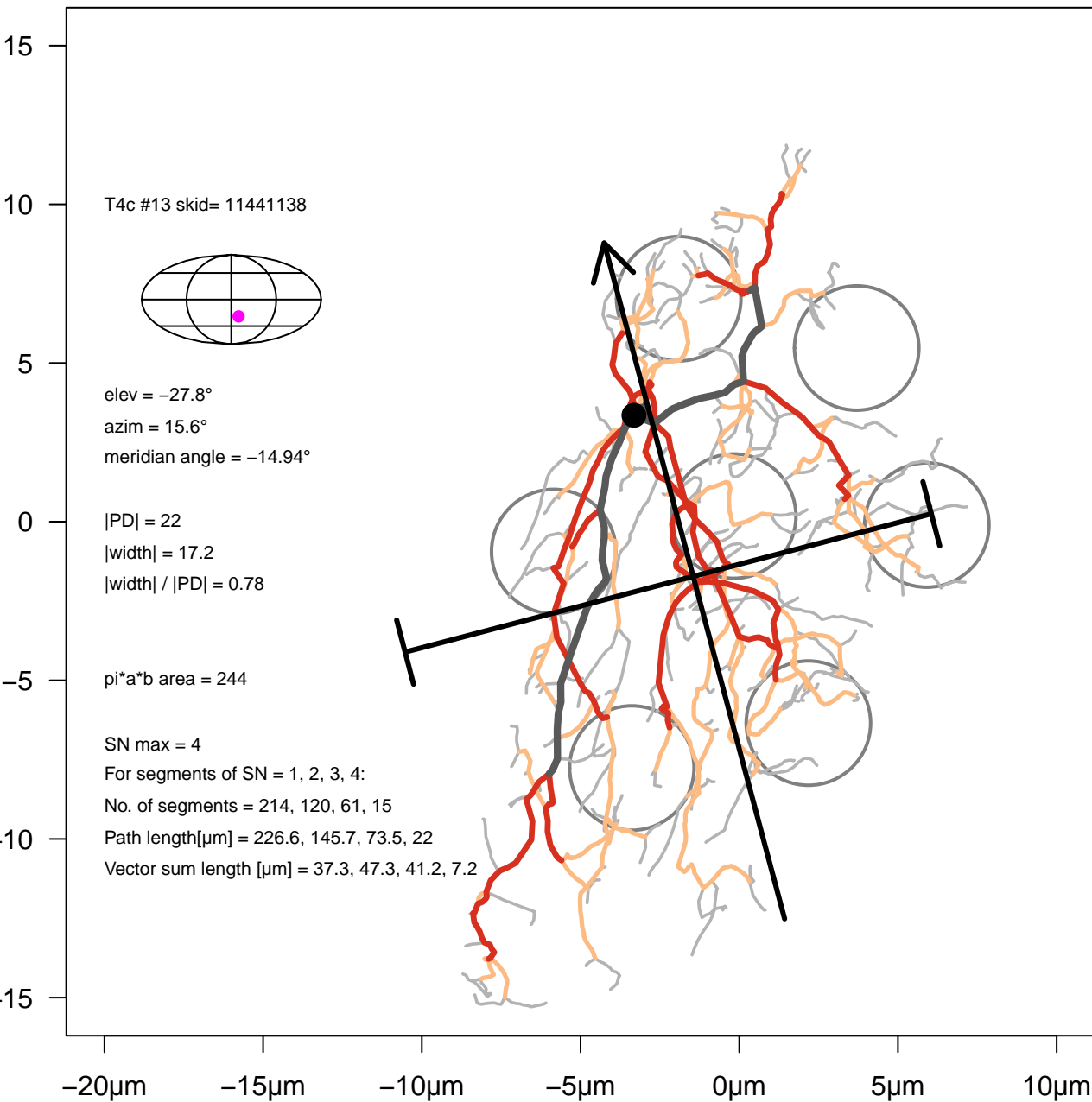

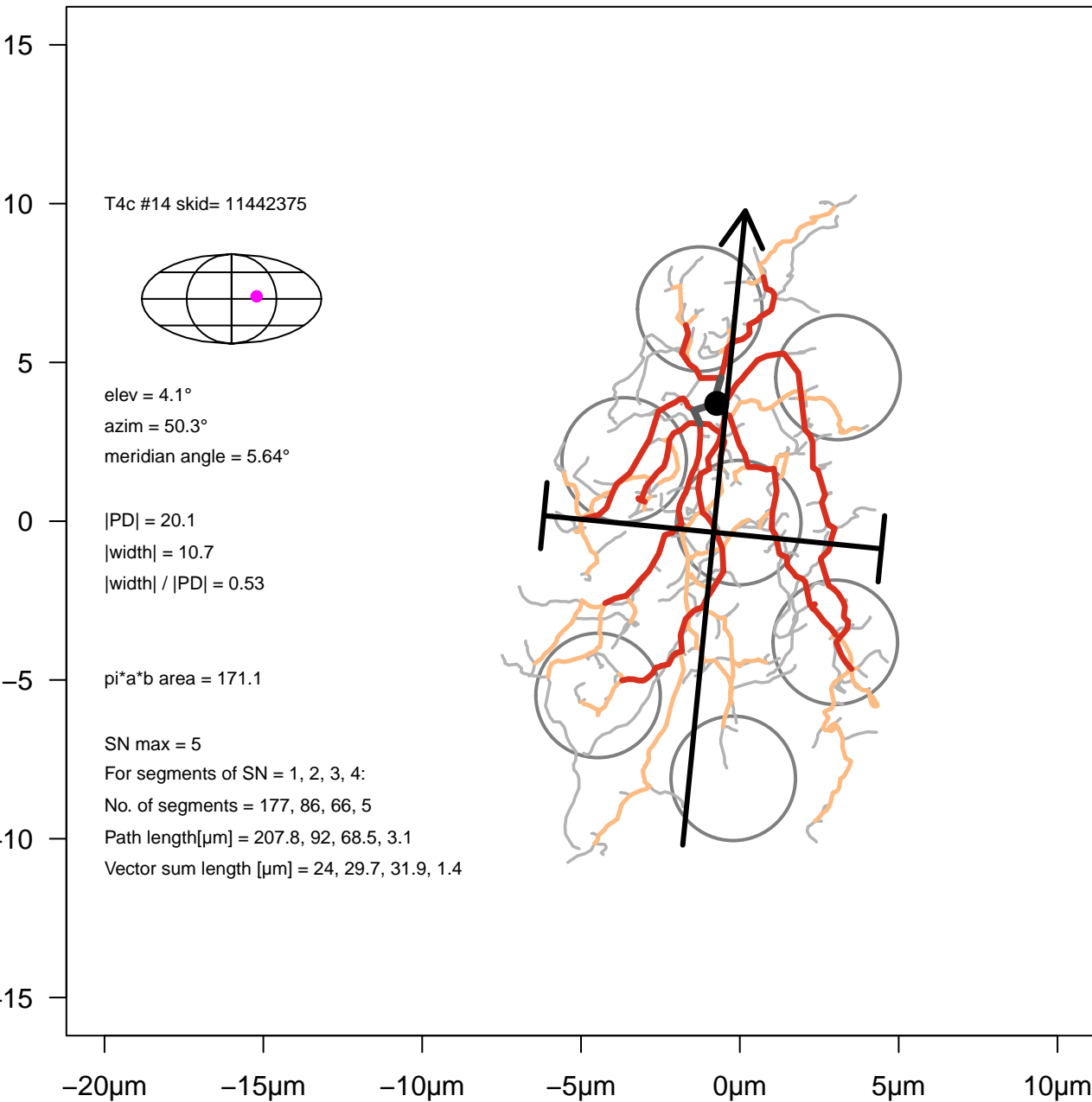

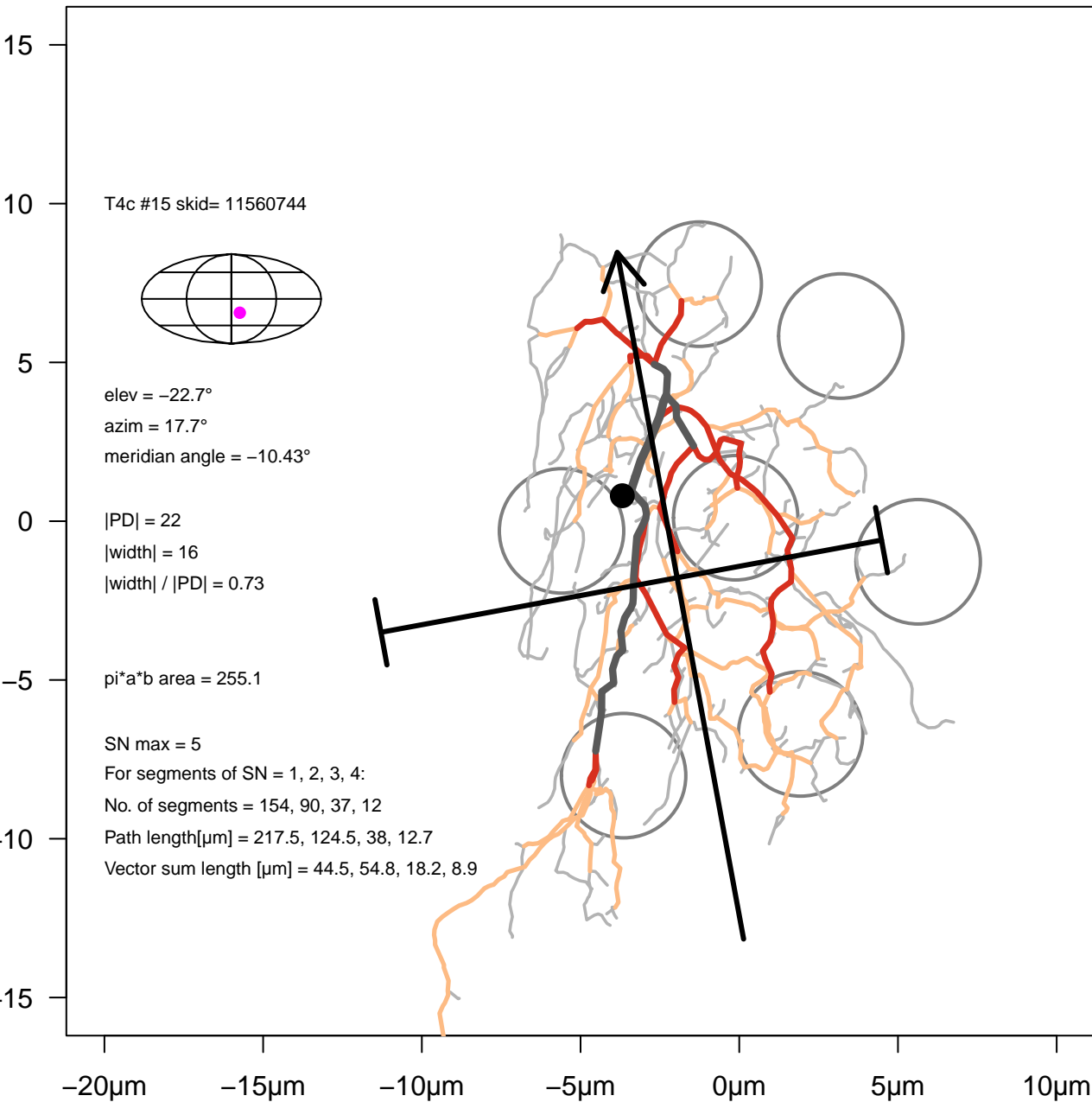

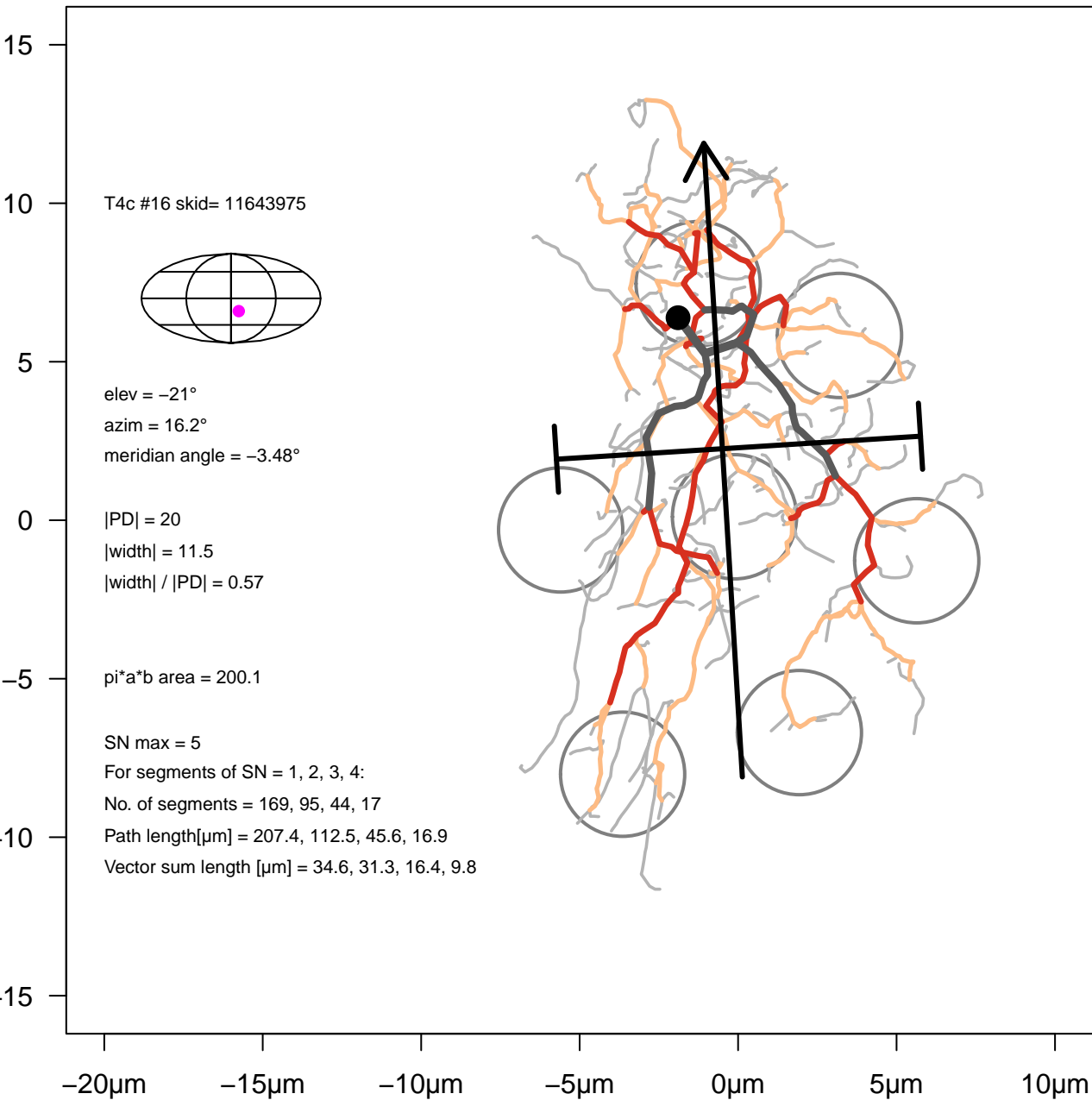

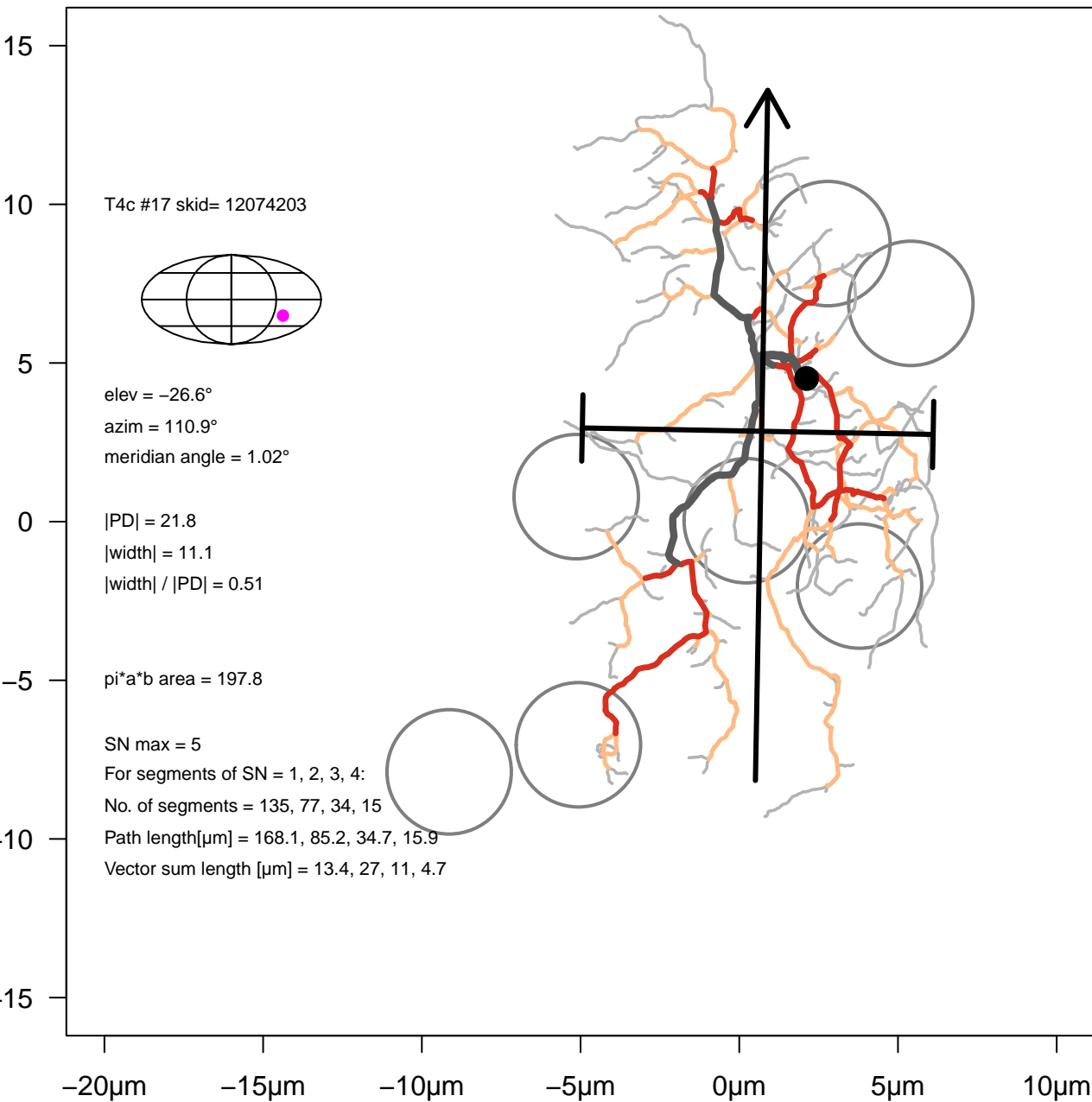

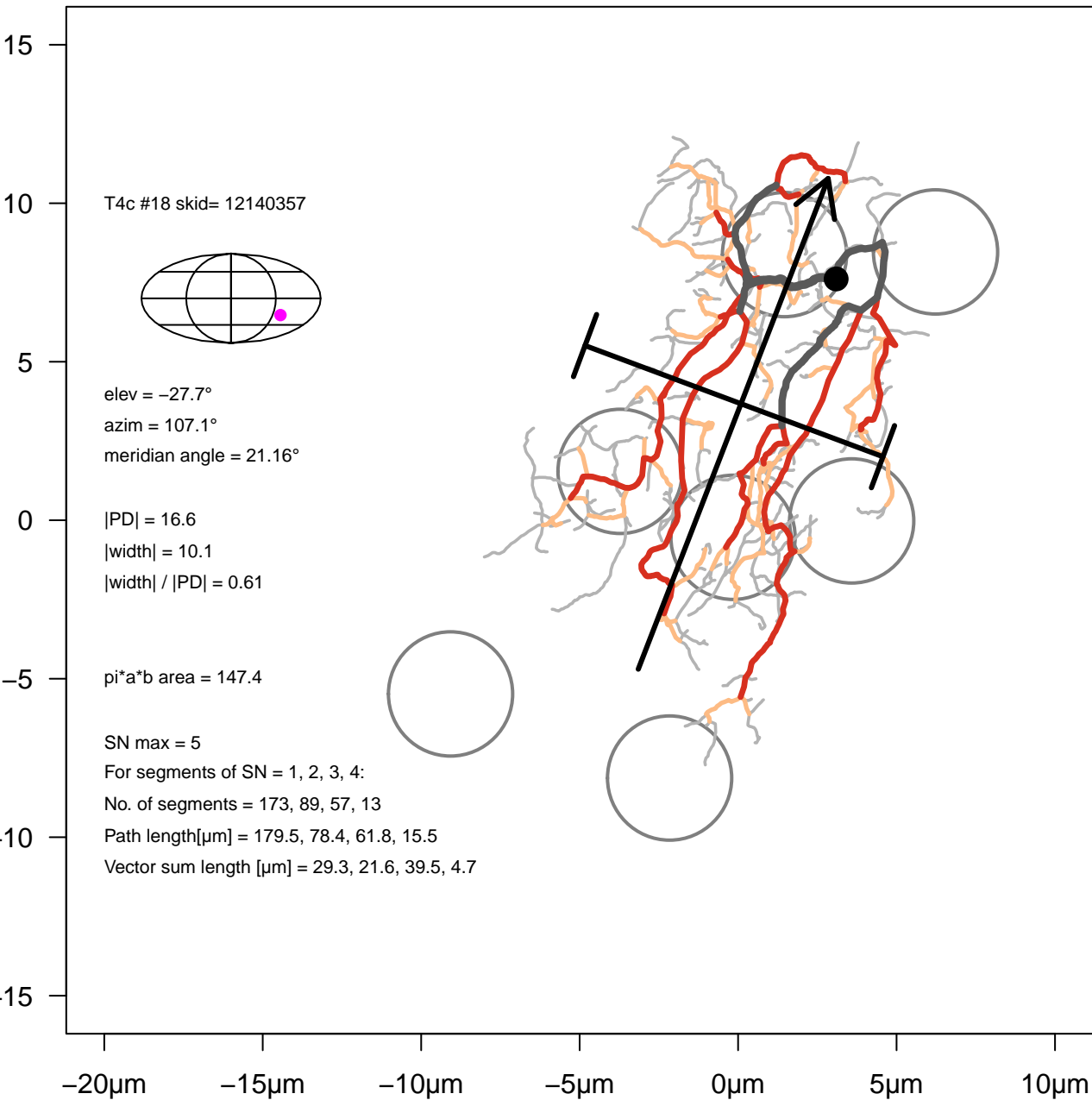

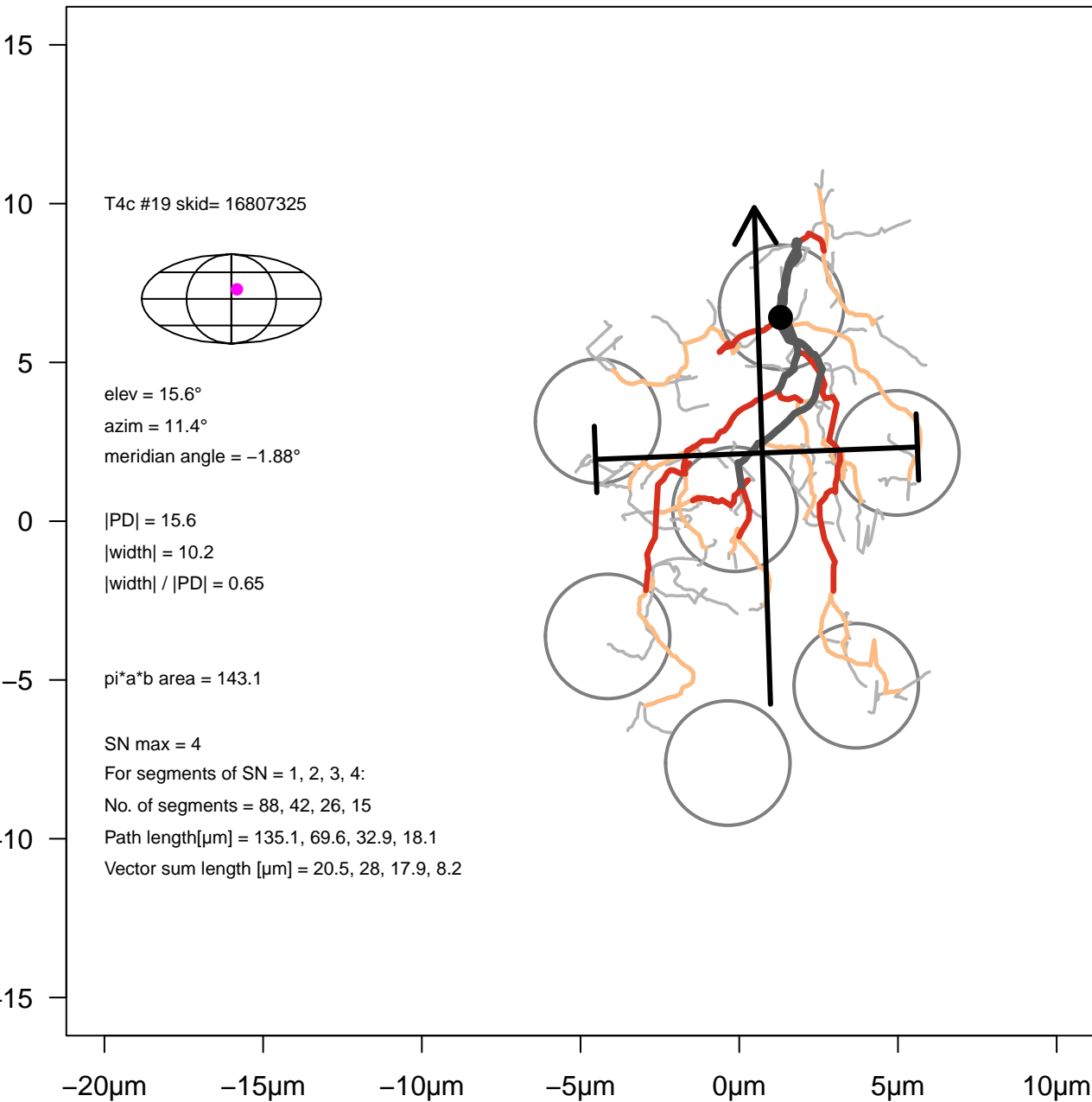

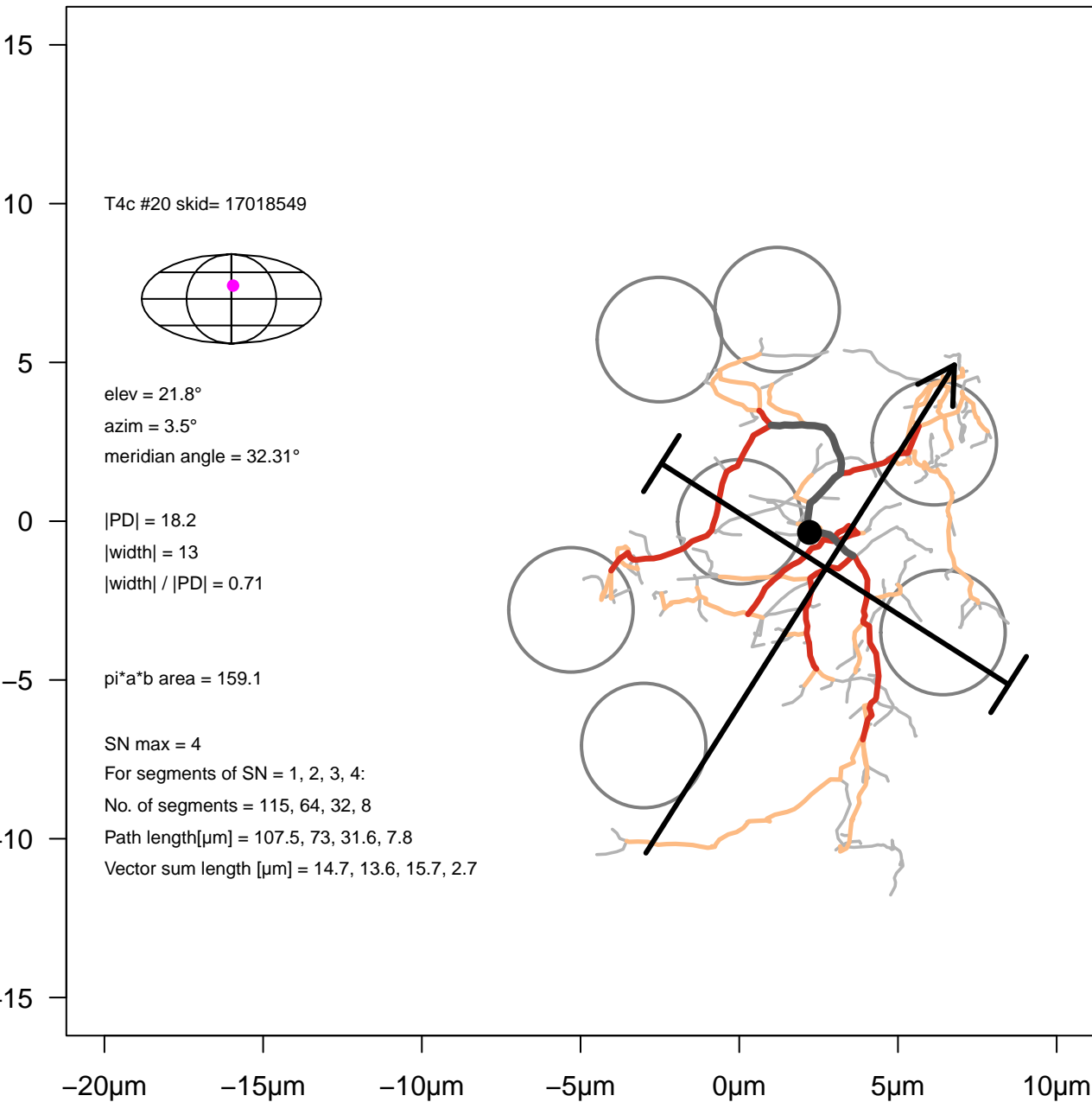

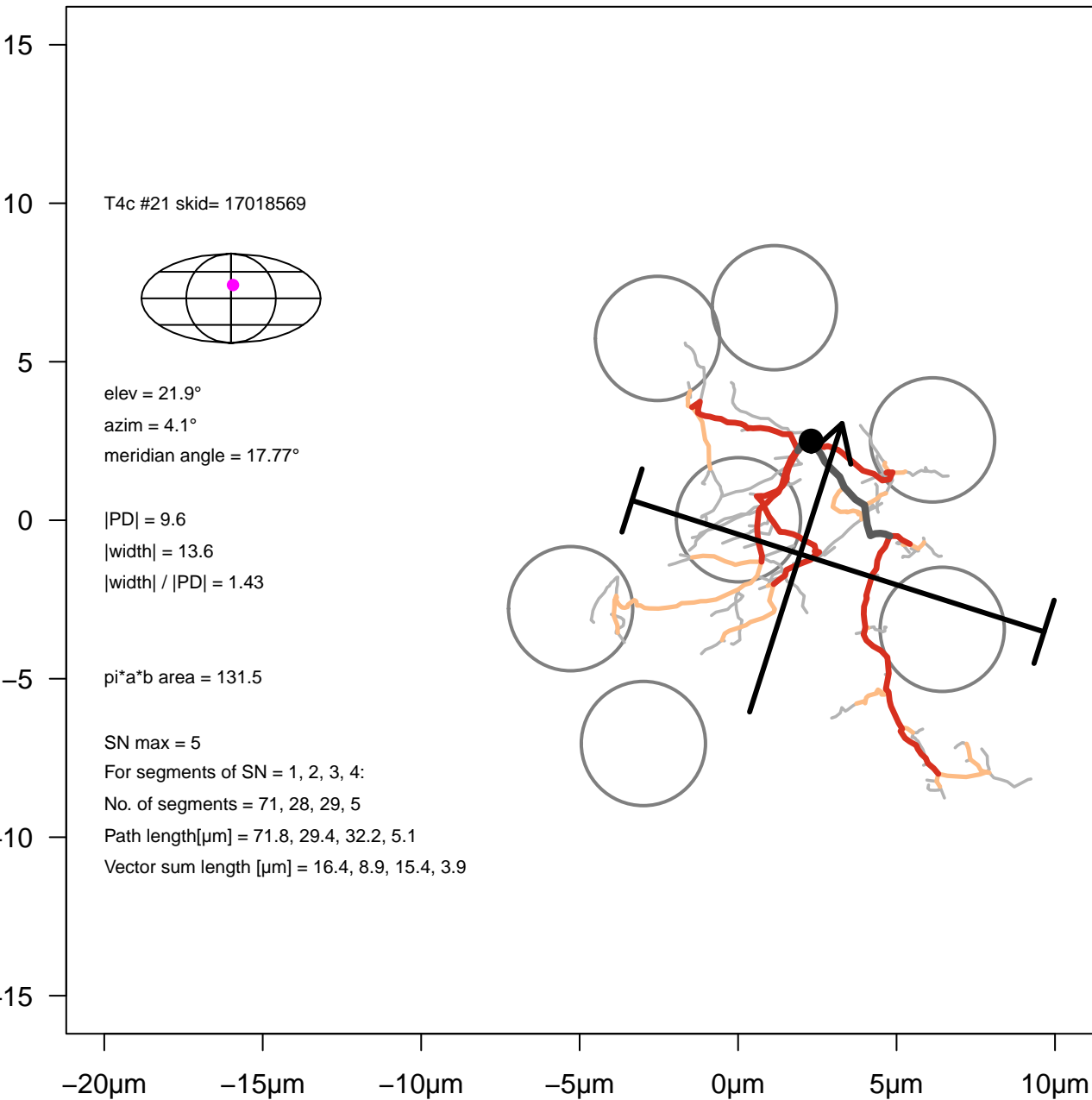

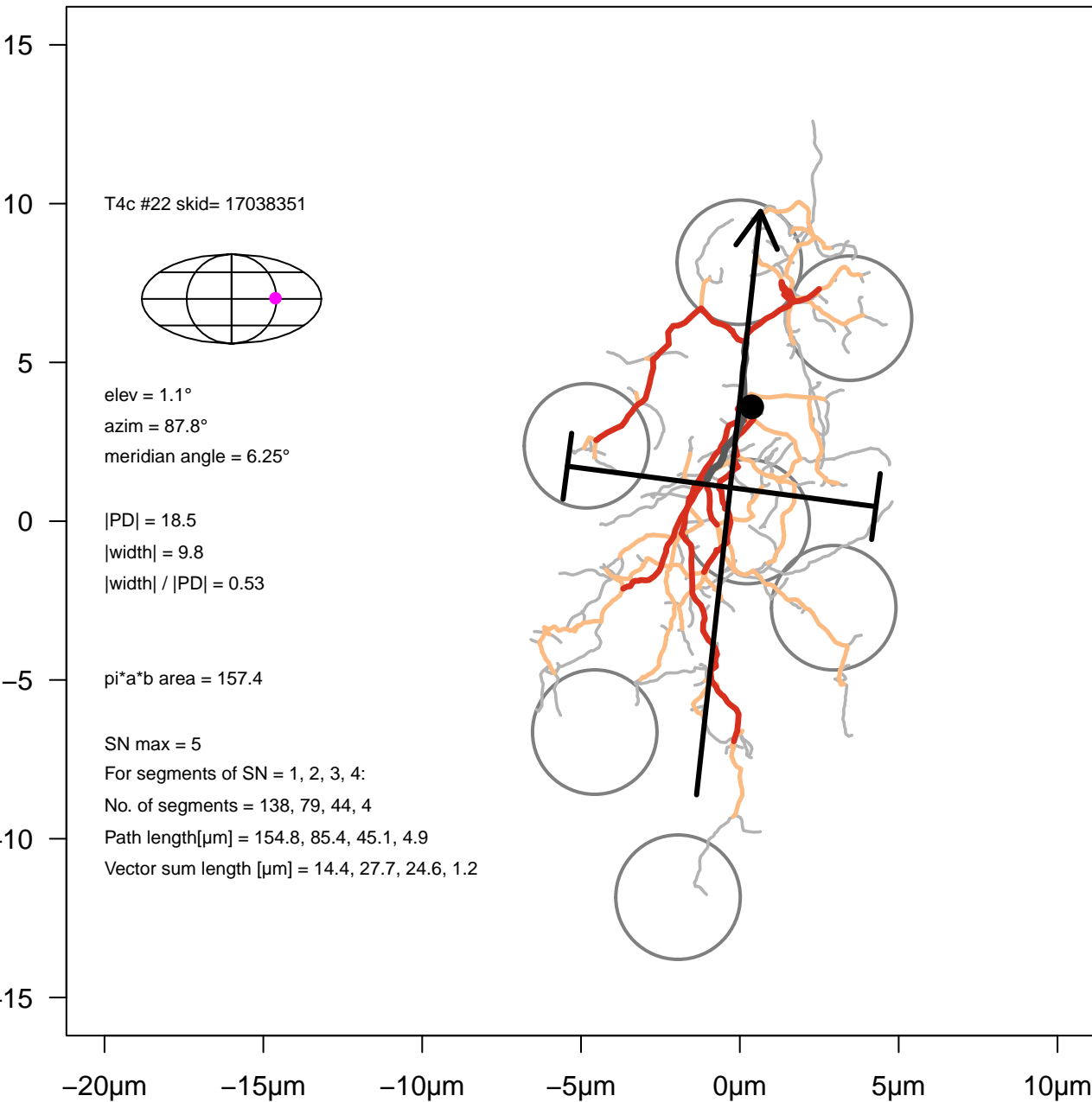

Supplement: Supplementary file 3 — Galleries of T4 neurons with PDs. All T4 neurons reconstructed in the FAFB dataset: 38 T4a, 176 T4b, 22 T4c, 114 T4d, are plotted similarly as in Fig. 2g. Using the eye map established in Fig. 4a, we include the position (elevation and azimuth angles) in the eye coordinate. The angle between T4’s PD and the local meridian line is computed instead of using the +v-axis as the reference, as in Fig. 2g. The meridian line is defined as the direction line going from the south pole to the north pole in the eye reference frame (often close to the +v-axis). The cell and surrounding columns are also aligned such that the vertical direction in the plot coincides with the meridian direction. A summary of the Strahler number analysis for each cell is included. [file 41586_2025_9276_MOESM3_ESM.zip › gallery_T4c.pdf]
